# Positive Selection of Transcription Factors Is a Prominent Feature of the Evolution of a Plant Pathogenic Genus Originating in the Miocene

**DOI:** 10.1093/gbe/evab167

**Published:** 2021-07-20

**Authors:** Mark C Derbyshire, Lincoln A Harper, Francisco J Lopez-Ruiz

**Affiliations:** Centre for Crop and Disease Management, Curtin University, Perth, Western Australia, Australia

**Keywords:** *d*_N_/*d*_S_, nonsynonymous, *Botrytis*, divergence time, Markov chain Monte Carlo, PAML

## Abstract

Tests based on the *d*_N_*/d*_S_ statistic are used to identify positive selection of nonsynonymous polymorphisms. Using these tests on alignments of all orthologs from related species can provide insights into which gene categories have been most frequently positively selected. However, longer alignments have more power to detect positive selection, creating a detection bias that could create misleading results from functional enrichment tests. Most studies of positive selection in plant pathogens focus on genes with specific virulence functions, with little emphasis on broader molecular processes. Furthermore, no studies in plant pathogens have accounted for detection bias due to alignment length when performing functional enrichment tests. To address these research gaps, we analyze 12 genomes of the phytopathogenic fungal genus *Botrytis*, including two sequenced in this study. To establish a temporal context, we estimated fossil-calibrated divergence times for the genus. We find that *Botrytis* likely originated 16–18 Ma in the Miocene and underwent continuous radiation ending in the Pliocene. An untargeted scan of *Botrytis* single-copy orthologs for positive selection with three different statistical tests uncovered evidence for positive selection among proteases, signaling proteins, CAZymes, and secreted proteins. There was also a strong overrepresentation of transcription factors among positively selected genes. This overrepresentation was still apparent after two complementary controls for detection bias due to sequence length. Positively selected sites were depleted within DNA-binding domains, suggesting changes in transcriptional responses to internal and external cues or protein–protein interactions have undergone positive selection more frequently than changes in promoter fidelity.

Significance

The genes most important to adaptation are often elusive in eukaryotes, including plant pathogenic fungi. One way to identify them is the use of tests of positive selection followed by tests for statistical enrichment of gene categories among positively selected genes. However, these tests are inherently biased as longer genes have more power to detect positive selection. In this study, we use tests that correct this length bias to uncover a significant overrepresentation of transcription factors among positively selected genes in the plant pathogenic fungal genus *Botrytis*, which is notorious for its pathogenesis of numerous plant species worldwide. We also dated the origin of this genus to around 16–18 Ma. Thus, we elucidate the importance of transcription factor evolution to adaptation of *Botrytis* fungi over the last few million years.

## Introduction

Positive selection is a process involving spread of alleles through a population as a result of their association with favorable phenotypes. Past positive selection can be identified in coding DNA sequences by comparing the rates of fixation of nonsynonymous and synonymous substitutions across alignments of orthologous codons from isolated lineages (Miyata and Yasunaga 1980; Nielsen and Yang 1998; Yang 1998, 2000; Yang and Nielsen 2002). Because nonsynonymous substitutions directly affect protein sequence, an increase in their fixation rate relative to that of synonymous substitutions could indicate positive selection on changes in protein structure.

By testing alignments of all orthologous genes among a group of species for positive selection, it is possible, via functional domain analysis, to assess what molecular processes have been most frequently involved in adaptation through coding sequence modification. This approach has been applied to numerous different groups of species across the tree of life (Kosiol et al. 2008; Lefébure and Stanhope 2009; Roux et al. 2014; Shultz and Sackton 2019; Yang et al. 2019).

Plant pathogenic fungi constitute an interesting group of organisms that experiences strong positive selective pressure from various factors such as host plants, dissemination to novel environments, and exposure to fungicides. They may thus be useful models for investigation of adaptation at the coding sequence level. A number of studies have investigated positive selection of coding sequences in plant pathogenic fungi. These studies have often focused on secreted proteins and especially candidate “effectors,” which are a group of proteins whose main function is alteration of host immune responses (Wicker et al. 2013; Guyon et al. 2014; Sperschneider et al. 2014; Schweizer et al. 2018; Stam et al. 2018; Beckerson et al. 2019; Derbyshire 2020). For example, Stukenbrock et al. (2011) investigated positive selection among a group of closely related pathogen species, including the wheat pathogen *Zymoseptoria tritici*. This study suggested that the recent emergence of *Z. tritici* as a pathogen of domesticated wheat coincided with enhanced adaptive fixations of nonsynonymous polymorphisms, especially those in effector proteins. Furthermore, Richards et al. (2019) found evidence for differential levels of positive selection among 17 effectors in two different North American populations of the wheat pathogen *Parastagonospora nodorum*.

Others have focused on specific functional classes of genes such as peptidases (Krishnan et al. 2018). However, relatively few studies of plant pathogenic fungi have involved an untargeted genome-wide assessment of which gene functions are most frequently under positive selection in the genome. Those that have done have found evidence of elevated positive selection among genes involved in processes such as mitochondrial function (Aguileta et al. 2010; Sillo et al. 2015), nutrient uptake (Aguileta et al. 2010), and responding to external stimuli (Schweizer et al. 2018).

When performing tests of functional enrichment among positively selected genes, it may be important to account for detection bias due to sequence length. This bias occurs because statistical power increases for site-wise models of positive selection when more codons are present in the alignment (Smith et al. 2015). However, to our knowledge, only a few studies (although none on plant pathogens) have controlled for this phenomenon. For example, a recent study by Shultz and Sackton (2019) used logistic regression to account for alignment length when determining enrichment of KEGG pathways among positively selected genes in birds and mammals.

We aimed to explore the kinds of functional domains that are enriched among positively selected genes in a plant pathogenic fungal taxon and to place this exploration into a temporal context. We performed our analyses on the genus *Botrytis*, which contains several species of significance to agriculture, including the host generalist *Botrytis cinerea*, which infects more than 200 species worldwide (Williamson et al. 2007).

The *Botrytis* genus has been subjected to a number of phylogenetic studies that have consistently identified two main clades (Staats et al. 2005; Walker et al. 2011; Zhou et al. 2014; Saito et al. 2016; Harper et al. 2019). Clade 1 contains the species *B. cinerea, Botrytis calthae, Botrytis pseudocinerea, Botrytis medusae, Botrytis fabae*, *Botrytis pelargonii, Botrytis sinoviticola*, and *Botrytis californica* and Clade 2 contains the rest of the known *Botrytis* species. The species in Clade 1 that have been most extensively studied, such as *B. cinerea, B. fabae, B. pelargonii*, and *B. calthae* only infect eudicot plants; Clade 2 contains both species that infect monocots and species that infect dicots. Using a molecular clock analysis without fossil calibration, it has been estimated that the host generalists *B. cinerea* and *B. pseudocinerea* were separated between seven and 18 Ma (Walker et al. 2011); however, divergence dates for the rest of the *Botrytis* phylogeny are unknown.

A previous study found evidence for positive selection in two *Botrytis* homologs of a phytotoxic necrosis and ethylene-inducing protein from the plant pathogen *Fusarium graminearum* (Staats et al. 2007). A further study on 642 expressed sequence tags suggested positive selection in 21 genes possibly involved in signal transduction and metabolism (Aguileta et al. 2012). A more recent study describes the genome sequences of ten *Botrytis* species (including nine new genomes and the reference genome of *B. cinerea*). Comparison of orthologous sequences between these species showed a complex pattern of presence/absence polymorphisms among secondary metabolite clusters with general conservation of function among secreted proteins (Valero-Jiménez et al. 2019). However, positive selection has not been investigated at the whole-genome scale in *Botrytis*. In our study, we sequence two new genomes, including those of *B. pseudocinerea* and *B. medusae* and analyze them in conjunction with ten existing genomes. We find that the two *Botrytis* clades likely share a common ancestor about 16–18 Ma in the mid-Miocene (23.03–5.333 Ma). We find evidence for continuing diversification of both clades throughout the Miocene and into the Pliocene (5.333–2.58 Ma), with the most recent divergences being between *B. pseudocinerea* and *B. cinerea*, and *Botrytis narcissicola* and *Botrytis tulipae* (both ∼4 Ma). Similar to previous studies, we found that positive selection was more likely to be detected in longer sequences.

With and without accounting for this bias, transcription factors were significantly enriched among positively selected genes. Positively selected codons in transcription factors were significantly depleted within DNA-binding domains, suggesting that promoter fidelity is generally conserved. We did not find any differences between branches in the numbers of transcription factors under positive selection that fit with known lifestyle attributes, suggesting that transcription factors evolve in response to a variety of different selective pressures. We hypothesize that diversification of gene regulatory networks through changes in transcription factor coding sequences has been a particularly important component of adaptive evolution in the *Botrytis* genus over the last 16–18 Myr.

## Results

### Two New Genome Sequences of *Botrytis* Species

In this study, we sequenced the genomes of *B. medusae* (Harper et al. 2019) and *B. pseudocinerea* (Walker et al. 2011). The error-corrected PacBio contigs and reads used to generate them are available in the NCBI data bases under the BioProject number PRJNA692037. Assembly stats are in supplementary table 4, Supplementary Material online. On the whole, the PacBio assemblies seemed better than the Illumina assemblies, although improvements were fairly marginal. PacBio assembly size was 40.6 Mb for *B. pseudocinerea* and 40.3 Mb for *B. medusae*, whereas Illumina genomes were 37.9 and 37.1 Mb, respectively. The number of contigs in PacBio sequences was 205 versus 319 in the Illumina assembly for *B. pseudocinerea* and 138 versus 380 for the *B. medusae* assembly.

### Confirming the Placement of *Botrytis* Species in Clades 1 and 2

To build an initial phylogeny on which to estimate fossil-calibrated divergence times, we used both maximum likelihood and Bayesian techniques. There were 107 fungal taxa in this alignment, including the *Botrytis* species sequenced by Valero-Jiménez et al. (2019) and the two newly sequenced species *B. pseudocinerea* and *B. medusae* from our study. Other fungal taxa in this phylogeny belonged to the Basidiomycota classes Agaricomycetes, Microbotryomycetes, Tremellomycetes, Ustilaginomycetes, and Xylonomycetes, and the Ascomycota classes Dothideomycetes, Eurotiomycetes, Lecanoromycetes, Leotiomycetes, Orbiliomycetes, Pezizomycetes, Saccharomycetes, Sordariomycetes, Taphrinomycetes, Tremellomycetes, Ustilaginomycetes, and Xylonomycetes. The super-alignment was a concatenation of 61 single-copy orthologs with a total of 37,627 sites.

**Fig. 1.**
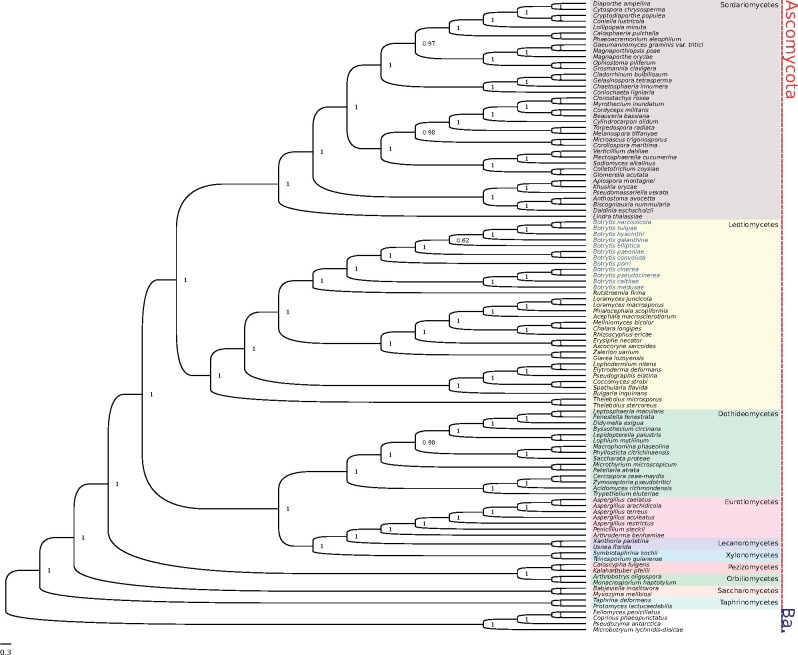
Bayesian phylogeny built from a partitioned protein alignment of 61 single-copy orthologs from 107 fungal species. The two major fungal phyla Ascomycota (in red) and Basidiomycota (in blue—“Ba.”) are marked to the right of the tree. Major fungal classes are represented in different color blocks. The 12 *Botrytis* species, which sit within the Leotiomycetes class, are highlighted in blue. Node labels indicate posterior support.

**Fig. 2.**
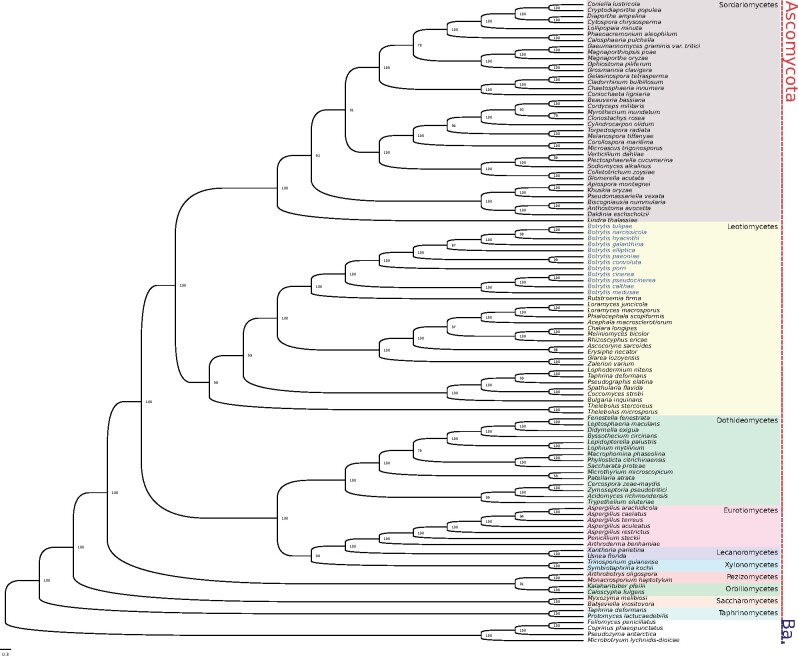
Maximum likelihood phylogeny built from a partitioned protein alignment of 61 single-copy orthologs from 107 fungal species. The two major fungal phyla Ascomycota (in red) and Basidiomycota (in blue—“Ba.”) are marked to the right of the tree. Major fungal classes are represented in different color blocks. The 12 *Botrytis* species, which sit within the Leotiomycetes class, are highlighted in blue. Node labels indicate bootstrap support.

Maximum likelihood and Bayesian topologies were identical (figs. 1 and 2). In the maximum likelihood tree, most nodes had a bootstrap (1,000 replicates) support of 100%. The only nodes with less than 90% support were the node ancestral to *Botrytis elliptica* and four other species in *Botrytis* clade 2, which had 67% support, a node separating two Sordariomycete clades, which had 78% support, and the node ancestral to *Myrothecium inundatum* and *Clonostachys rosea*, which had 79% support. The Bayesian tree was similarly well supported as posterior probabilities for most nodes were 1. The only node with lower posterior probability was the same node ancestral to *B. elliptica* and four other *Botrytis* Clade 2 species, which had a posterior probability of 0.62. The *Botrytis* portion of the tree was in agreeance with previous topologies (Staats et al. 2005; Zhou et al. 2014; Saito et al. 2016; Harper et al. 2019).

### The *Botrytis* Genus Likely Radiated in the Miocene and Pliocene Epochs

To understand the recent evolutionary history of the *Botrytis* genus, we estimated species divergence times using Bayesian MCMC. Since there are no fossilized *Botrytis* species specimens, we performed the analyses on a selection of 107 diverse fungal taxa and calibrated the tree using three of the five fossils used by Beimforde et al. (2014). As mentioned, initial topology estimates from both maximum likelihood and Bayesian approaches were identical (figs. 1 and 2). Therefore, node ages were estimated arbitrarily on the Bayesian topology. We performed the analysis on both an unpartitioned super-alignment with nucleotide sites and an alignment split into 20 partitions based on substitution rate similarity (supplementary table 3, Supplementary Material online). Partitioned alignments have the potential to reduce the variance of divergence date estimates, but the choice of partitioning scheme may cause biased estimates if partitions are not independent realizations of the rate-drift process. Therefore, the partitioned analysis is considered in the context of the unpartitioned analysis, which is likely to be unbiased but also less precise. Trace plots of the model log likelihood and ages of the first ten nodes are shown in supplementary figure 1, Supplementary Material online. Overall, there were scant differences between the two independent runs of the MCMC (all outputs are in supplementary file 5, Supplementary Material online) and, based on the observation that there was no movement of mean values through parameter space for all 10,000 generations, Markov Chains appeared to have converged sufficiently.

**Fig. 3.**
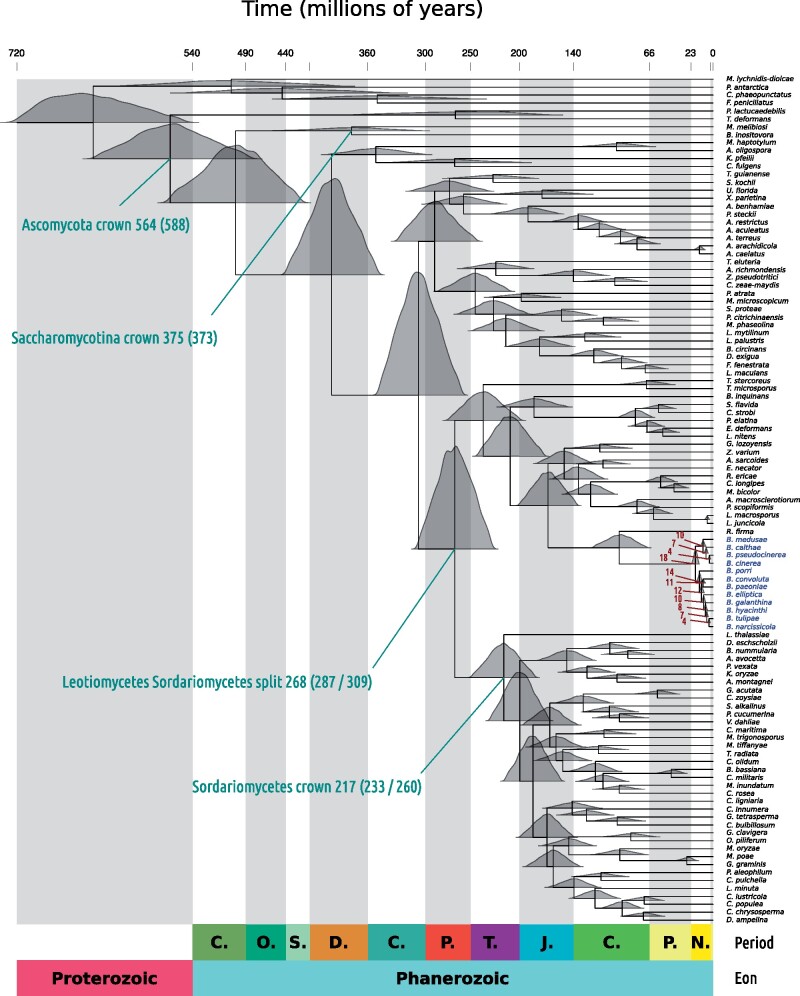
Time tree of 107 fungal species generated through Markov chain Monte Carlo using fossil calibrations in three nodes. The time scale on the *x* axis is in millions of years. The different colored blocks at the bottom of the figure represent different geological periods, Cambrian (C.), Ordovician (O.), Silurian (S.), Devonian (D.), Carboniferous (C.), Permian (P.), Triassic (T.), Jurassic (J.), Cretaceous (C.), Paleogene (P.), and Neogene (N.), and the geological eons Proterozoic and Phanerozoic. The gray blocks emanating from the base of the plot divide the tree into different geological periods. Density plots above nodes in the tree are the full posterior distributions of node ages from the Markov Chain. Four major events are labeled in turquoise. In brackets are the dates estimated by Beimforde et al. (2014) using five fossil calibrations. *Botrytis* species names are highlighted in blue and node ages in the *Botrytis* clade are labeled in dark red. This tree is one of two trees built using unpartitioned nucleotide data. Both runs of the MCMC are in supplementary file 5, Supplementary Material online. Supplementary figure 1, Supplementary Material online, shows trace plots of tree log likelihood and the first ten node ages for this tree. The same plot based on the tree built from the partitioned alignment is in supplementary figure 2, Supplementary Material online. All node age estimates were consistent with radiation of *Botrytis* starting in the Pliocene and ending in the Miocene.

Several node ages on this topology agreed with Beimforde et al. (fig. 3). For instance, using the unpartitioned data set, we estimated the divergence between Ascomycota and Basidiomycota to have occured 643 Ma (95% HPD = 546–719), whereas Beimforde et al. estimated a divergence date of 642 Ma (95% HPD = 504–859); using the partitioned data set, the estimate was a little earlier at 670 Ma (95% HPD = 599–727). Similarly, Beimforde et al. estimated a date of 588 Ma (95% HPD = 487–773) for the common ancestor of the Ascomycota, whereas we estimated slightly more recent dates of 564 Ma (95% HPD = 488–644) with the unpartitioned data set and 556 Ma (95% HPD = 497–605) with the partitioned data set. Our estimates for the common ancestor of the Saccharomycotina, at 375 Ma (303–450) and 389 Ma (345–427), for the partitioned and unpartitioned data sets, respectively, were also similar to Beimforde et al.’s 373 Ma (95% HPD = 276–514).

Divergence dates for some taxa were considerably more recent from our estimates compared with those of Beimforde et al. For example, the partitioned and unpartitioned data sets both estimated an age of 247 Ma (95% HPD = 221–269 and 211–282, respectively) for the common ancestor of the Dothideomycetes, whereas Beimforde et al. estimated 350 Ma (95% HPD = 273–459). The Leotiomycete Sordariomycete divergence was dated by Beimforde et al. at 309 Ma (95% HPD = 267–430), whereas we estimated dates of 268 Ma (95% HPD = 179–374) and 257 Ma (95% HPD = 230–280), using the unpartitioned and partitioned data sets, respectively.

Overall, the partitioned data set estimated slightly more recent divergence times with substantially reduced 95% HPD intervals. Using either the partitioned or unpartitioned data set did not significantly affect divergence time estimates for the *Botrytis* genus. The common ancestor of *Botrytis* was dated to 18 Ma (95% HPD = 15–22) using the unpartitioned data set and 16 Ma (95% HPD = 14–17) using the partitioned data set. The common ancestor of Clade 1 (including *B. cinerea*, *B. pseudocinerea*, *B. medusae*, and *B. calthae*) was dated to 10 Ma (95% HPD = 8–13) with the unpartitioned data set and 9 Ma (95% HPD = 8–10 Ma) with the partitioned data set. The common ancestor of *Botrytis* Clade 2 (including the other eight species) was dated to 14 Ma (95% HPD = 12–17) using the unpartitioned data set and 12 Ma (95% HPD = 11–13) using the partitioned data set. The most recently diverging *Botrytis* species were *B. pseudocinerea* and *B. cinerea* at 4 Ma and *B. tulipae* and *B. narcissicola*, also at 2 Ma. These data overall suggest that the two main clades in the *Botrytis* genus diverged in the mid-Miocene epoch (5.333–23.03 Ma) and continued diversifying through the Miocene into the Pliocene (2.588–5.333 Ma).

### Positive Selection Is More Likely to Be Identified in Longer Alignments

Positive selection may be inferred from sequence alignments by determining the ratio of the rate of fixation of nonsynonymous substitutions per nonsynonymous site (*d*_N_) to the rate of fixation of synonymous substitutions per synonymous site (*d*_S_). If this ratio, referred to as *ω*, is above 1, it is possible that the gene in question has undergone positive selection of nonsynonymous substitutions. The null hypothesis in this case is a composite of neutral evolution, where *d*_N_ = *d*_S_ and *ω* = 1 and purifying selection where *d*_N_ < *d*_S_ and *ω* < 1.

We used three different tests to assess positive selection in *Botrytis* single-copy orthologs, the site models described in Nielsen and Yang (1998) and Yang (2000) and the branch-site model of Murrell et al. (2015). For the site models, we compared the null model M1a with the positive selection model M2a (described as “M1a vs. M2a”) and the null model M7 with the positive selection model M8 (described as “M7 vs. M8”); the test from Murrell et al. is described as “BUSTED.”

**Fig. 4.**
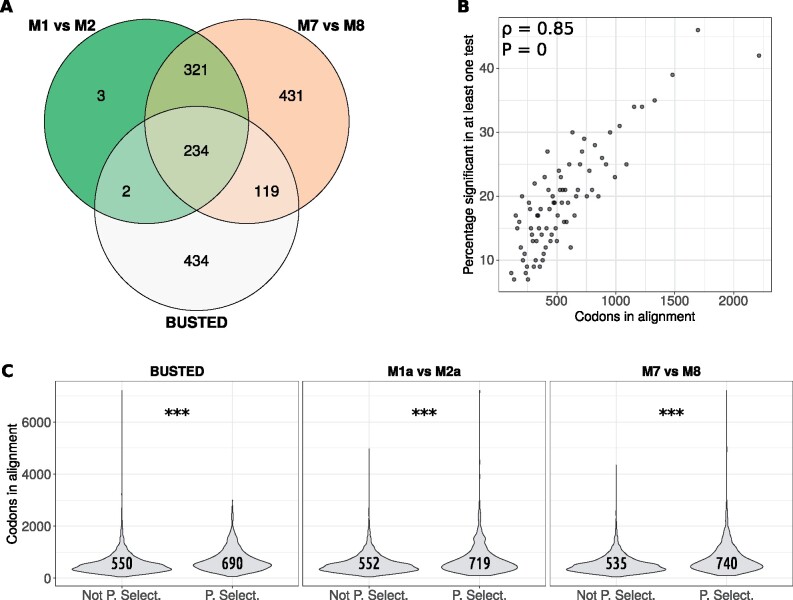
Overview of results of whole-genome scans for positive selection. (*A*) A Venn diagram showing the number of genes found to be under positive selection in the three tests and how many overlapped between tests. (*B*) An assessment of the correlation between alignment length and detection of positive selection. Alignments were binned into groups of 500 from shortest to longest and the mean number of codons in alignment for each bin is on the *x* axis. The *y* axis shows the percentage of genes in each bin that were found to be under positive selection in at least one test. (*C*) Violin plot showing the difference in alignment length between nonselected and positively selected genes for each of the three tests. The *y* axis shows alignment length in codons and status of “not positively selected” (Not P. Select.) and “positively selected” (P. Select.) are on the *x* axis. Violins show kernel density and asterisks indicate significance from a Student’s *t-*test. “Three asterisks” indicates significance at an *α* of 0.001. The numbers inside violins show the mean lengths for each class for each test. Test names are given above each plot.

We identified 7,848 single-copy orthogroups in total. We found that M1a versus M2a predicted positive selection in 840 (10.7%) of these single-copy orthologs, M7 versus M8 in 1,767 (22.52%), and BUSTED in 1,377 (17.55%) at alpha = 0.05. After false discovery rate correction using the Benjamini–Hochberg method, M1a versus M2a predicted positive selection in 560 (7.14%) single-copy orthologs, M7 versus M8 in 1,105 (14.08%), and BUSTED in 789 (10.05%) at alpha = 0.05. Following false discovery rate correction, positive selection was predicted by all three approaches in 234 (2.98%) single-copy orthologs, by only M1 versus M2 in 3 (0.04%) by only M7 versus M8 in 431 (5.49%) and by only BUSTED in 434 (5.53%). Both M1a versus M2a and M7 versus M8 but not BUSTED predicted positive selection in 321 (4.09%) single-copy orthologs; both BUSTED and M7 versus M8 but not M1a versus M2a predicted positive selection in 119 (1.52%) single-copy orthologs; and both BUSTED and M1a versus M2a but not M7 versus M8 predicted positive selection in 2 (0.03%) single-copy orthologs (fig. 4*A*).

There was a clear impact of alignment length on the ability of the different models to detect positive selection (fig. 4*B* and *C*). In bins of from shortest to longest alignment, there was a correlation between median bin alignment length in codons and percentage of significant bin alignments (Pearson’s ρ = 0.86, *P* = 0) (fig. 4*B*). For each individual test, there was a significant difference in the mean number of codons in the alignment between positively selected and nonpositively selected orthogroups (fig. 4*C*).

Since sample sizes were large (7,848 single-copy orthogroups divided into two groups for each test), the Wilcoxon and *t*-tests that were used had a large amount of power, which could possibly be interpreted as an overpowering of the tests. Therefore, we also investigated effect sizes using Cohen’s *d*, which, in our context, is the mean length of nonselected orthogroups minus the mean length of positively selected orthogroups divided by the total SD. We interpret a Cohen’s *d* of 0.2 (the means are 0.2 SDs apart) as a small effect, 0.3–0.7 as a medium effect and >0.7 as a large effect (Lakens 2013). In the following, negative values would occur if the mean of the nonselected groups were smaller and positive values would occur if the mean of the positively selected groups were smaller. Thus, being positively selected according to M1 versus M2 had a medium effect of *d* = −0.43 (CI = −0.51/−0.34) on alignment length; being positively selected according to M7a versus M8a had a medium effect of *d* = −0.53 (CI = −0.6/−0.47); and, being positively selected according to BUSTED also had a medium effect of *d* = −0.36 (−0.43/−0.29) on alignment length.

Overall, there was some agreement between the three tests of positive selection, although BUSTED was more different to M1a versus M2a and M7 versus M8 than they were to each other. For all tests, there was a significant impact of alignment length on ability to detect positive selection. In the next section on functional enrichment, we refer to orthogroups predicted to be under positive selection by at least one set as the “low confidence set” and those predicted to be under positive selection by all tests as the “high confidence set.”

### Functions Associated with Signaling/Transcriptional Regulation and Carbohydrate Metabolism Are Enriched among Positively Selected Single-Copy Orthogroups

To assess what functional categories of genes may most frequently undergo positive selection in *Botrytis*, we performed a Fisher’s exact test for enrichment of InterPro domains among positively selected single-copy orthogroups. Each orthogroup was assigned a single InterPro domain if it was present in any of its members.

We were able to assign at least one InterPro domain to 6,324 single-copy orthogroups. Of these domains, 31 were significantly enriched (*α* < 0.05) among the 1,544 orthogroups predicted to be positively selected by at least one test. Among the higher confidence set of 234 orthogroups predicted to be under positive selection by all three tests, 14 domains were significantly enriched (*α* < 0.05).

**Fig. 5.**
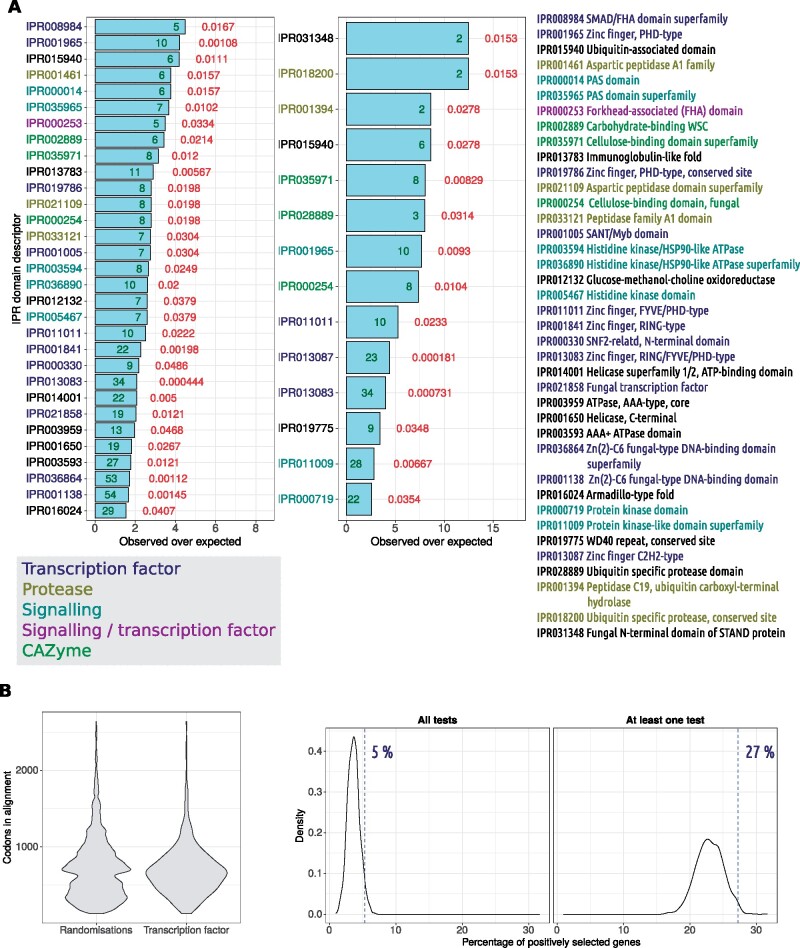
Enrichment of InterPro terms among positively selected genes in the *Botrytis* genus. (*A*) Barplots showing results of a Fisher’s exact test for enrichment among positively selected orthogroups. The *x* axis displays the observed number of orthogroups with the InterPro term (on the *y* axis) over the expected number based on its overall abundance. Green numbers show the number of orthogroups containing the term and red numbers show *P* values associated with the enrichment test. InterPro terms are colored depending on the category to which they belong, which is one of “transcription factor,” “protease,” “signaling,” “signaling/transcription factor,” and “CAZyme” (which stands for “carbohydrate active enzyme”). Full InterPro term descriptors colored in the same way are given to the right of the plot. (*B*) The difference in alignment length in codons between all orthogroups in the full set of randomizations and orthogroups flagged as “transcription factors” based on the InterPro terms identified by Shelest (2017). The aim of this randomization test was to determine the proportion of orthogroups of an equivalent average alignment length to transcription factors that were found to be under positive selection. (*C*) Shows the result of the randomization test. The *x* axis plots the percentage of positively selected genes and the *y* axis shows the kernel density based on all randomizations. The vertical dashed lines show the percentage of transcription factors shown to be under positive selection, which is compared with the distribution of percentages from the randomizations. Results are plotted for either all three tests (left) or at least one test (right). In both instances, less than 5% of randomizations had a percentage of positively selected orthogroups more than or equal to the percentage of positively selected transcription factor orthogroups.

After inspecting InterPro domains, we manually grouped the overrepresented domains into likely transcription factors, proteases, signaling proteins, signaling/transcription factor proteins or CAZymes. We find that these different categories make up the majority of enriched domains in both sets (fig. 5*A*). Among likely transcription factors, Zinc finger domains were particularly prominent as they made up eight out of the 12 likely transcription factor domains that were significantly enriched. Two likely transcription factor InterPro domains with particularly good evidence of enrichment among positively selected orthogroups were IPR013087 “Zinc finger C2H2-type,” which was present in 23 positively selected orthogroups in the high confidence set (Fisher’s exact test *P* = 0.000181, enrichment = 4.40 times expected), and IPR013083 “Zinc finger, RING/FYVE/PHD-type,” which was present in 34 orthogroups in the high confidence set (Fisher’s exact test *P* = 0.000731, enrichment = 3.98 times expected). A likely signaling protein InterPro domain with particularly good evidence of enrichment among positively selected orthogroups was IPR011009 “Protein kinase-like domain superfamily,” which was present in 28 orthogroups in the high confidence set (Fisher’s exact test *P* = 0.00667, enrichment = 2.81 times expected).

Since domains appeared to be members of distinct broad functional classes, we grouped genes into either CAZymes, using dbCAN, or transcription factors using the InterPro domains identified by Shelest (2017). We also predicted whether proteins contained secretion signals as we a priori expect secreted proteins to undergo enhanced positive selection. If at least one member of the alignment had a secretion signal, the orthogroup was flagged as secreted and likewise for status as “CAZyme.” All statistics quoted are from Fisher’s exact tests with a Benjamini–Hochberg false discovery rate correction. Contrary to our expectations, we found that CAZymes were not enriched among orthogroups predicted as positively selected by at least one test (*P* = 0.12; odds ratio = 1.20) or the high confidence set (*P* = 0.58; odds ratio = 0.73). However, secreted CAZymes showed some evidence of enrichment among the low confidence set (*P* = 0.026; odds ratio = 1.53) but not the high confidence set (*P* = 0.83; odds ratio = 1.05). Secreted proteins in general also showed some evidence of enrichment in the low confidence set (*P* = 0.079; odds ratio = 1.19) but not in the high confidence set (*P* = 0.64; odds ratio = 1.09). In contrast to the other categories, transcription factors showed good evidence of being overrepresented among positively selected orthogroups in both the low confidence (*P* = 0.0005; odds ratio = 1.57) and high confidence (*P* = 0.027; odds ratio = 1.93) sets.

Since detection of positive selection was strongly linked to protein length, we tested whether orthogroup alignments in these functional categories were significantly longer or shorter than others. We found that orthogroups in two categories had alignments significantly longer than average (*α* = 0.05). All following *P* values are adjusted for false discovery rate using the Benjamini–Hochberg procedure. CAZyme alignments had a mean length of 643.82 codons, whereas non-CAZyme alignments had a mean length of 559.28 codons (Welch’s *t P* = 7.50e^−06^; Wilcoxon’s test *P* = 8.49e^−12^); transcription factor alignments had a mean length of 693.58 codons, whereas nontranscription factor alignments had a mean length of 556.95 codons (Welch’s *t P* = 2.35e^−14^; Wilcoxon’s test *P* = 1.75e^−28^). However, secreted protein orthogroup alignments were on average significantly shorter than nonsecreted protein orthogroup alignments, with a mean of 488.27 codons versus a mean of 571.65 codons (Welch’s *t P* = 6.63e^−10^; Wilcoxon’s test *P* = 8.07e^−08^). Secreted CAZymes were not significantly different in length to the average alignment (Welch’s *t P* = 0.89; Wilcoxon’s test *P* = 0.087). Similarly to the previous section, we also assessed effect size for these comparisons using Cohen’s *d*. Thus, assignment to the category “CAZyme” had a small effect of *d* = −0.21 (CI = −0.31/−0.12) on alignment length; assignment to the category “transcription factor” had a medium effect of *d* = −0.35 (CI = −0.45/−0.25) on alignment length; and, assignment to the category “secreted” had a small effect of *d *=* *0.21 (CI = 0.14/0.29). Note, the positive Cohen’s *d* estimate for secreted protein orthogroups is reflective of the fact that they had a “shorter” alignment length than nonsecreted proteins.

Since transcription factors were significantly longer than the average alignment and showed evidence of enrichment in positively selected orthogroups, we performed tests to determine whether this enrichment was still evident after correction for alignment length. Since secreted proteins showed possible evidence for enrichment among the positively selected set of orthogroups but had a shorter alignment length than average, we also performed tests to determine whether there was, in fact, an enrichment when this shorter alignment length was taken into account. Our first approach was to fit two logistic regression models with the binary status of “positively selected” or “not positively selected” as the dependent variable. The null model contained only alignment length as the predictor and the alternative model contained alignment length and membership of each of the categories; for example, the binary classification of “transcription factor” or “not transcription factor.” We compared the null and full models with a likelihood ratio test assuming a χ^2^ distribution with df 1. All statistics quoted are *P* values adjusted using the Benjamini–Hochberg false discovery rate correction procedure. We found that, for both low and high confidence sets, transcription factors maintained their association with status as “positively selected” even when correcting for length (*P* = 0.005 for both sets). For the high confidence set, the Akaike Information Criterion (AIC) decreased from 2,072.53 to 2,069.06; for the low confidence set, AIC decreased from 7,558.72 to 7,552.89. Secreted proteins also improved model fit for the low confidence set over alignment length alone (*P* = 0.0034) but not for the high confidence set (*P* = 0.45). For the high confidence set, AIC increased from 2,072.53 to 2,073.97. For the low confidence set, AIC decreased rom 7,558.72 to 7,552.15. Despite this weaker evidence for positive selection in secreted proteins, it should be noted that, on average, *Botrytis* species contained 1,069 (SD of ±41) secreted proteins (supplementary fig. 3, Supplementary Material online). Since there were only 710 secreted proteins in the set of single-copy orthogroups, the tests we performed are limited in scope to proteins that have not been lost, gained, or duplicated.

As there was evidence of enrichment of transcription factors among positively selected orthogroups in spite of the length bias, we sought further confirmatory evidence of this phenomenon. We therefore conducted a randomization test. This test took 1,000 random gene samples, without replacement, of the same size as the number of transcription factors (411 orthogroup alignments). These samples were taken from between the lengths of the shortest and longest transcription factor alignments to make their average length similar to that of the transcription factors; sampling was weighted to mimic the overall distribution of transcription factor lengths (see methods). The proportion of each sample that was positively selected was recorded for each randomization. The *P* values quoted are the number of random samples for which the proportion of positively selected random orthogroup alignments was greater than the proportion of positively selected transcription factors. We found that the overall mean length of all 1,000 x 411 random alignments was not significantly different to that of the transcription factor alignments (Welch’s *t P* = 0.18, Wilcoxon’s test *P* = 0.87). We found that for both the low confidence and high confidence sets, a significantly high proportion of transcription factors was positively selected, compared with an arbitrary orthogroup set of equivalent length (*P* = 0.009 and 0.029, respectively) (fig. 5*B*). In total, we annotated 411 orthogroups as transcription factors based on the domains identified by Shelest (2017). Of these 411 orthogroups, 112 (27.5%) were identified as being under positive selection by at least one test. This is a 39.81% increase in proportion over the 19.67% of all orthogroups identified as being under positive selection in at least one test. A total of 22 (5.35%) of the 411 transcription factor orthogroups were identified as being under positive selection in all three tests. This is a 79.53% increase in proportion over the 2.98% of all orthogroups identified as being under positive selection in at least one test.

### Positively Selected Sites Are Depleted in Transcription Factor DNA-Binding Domains

**Fig. 6.**
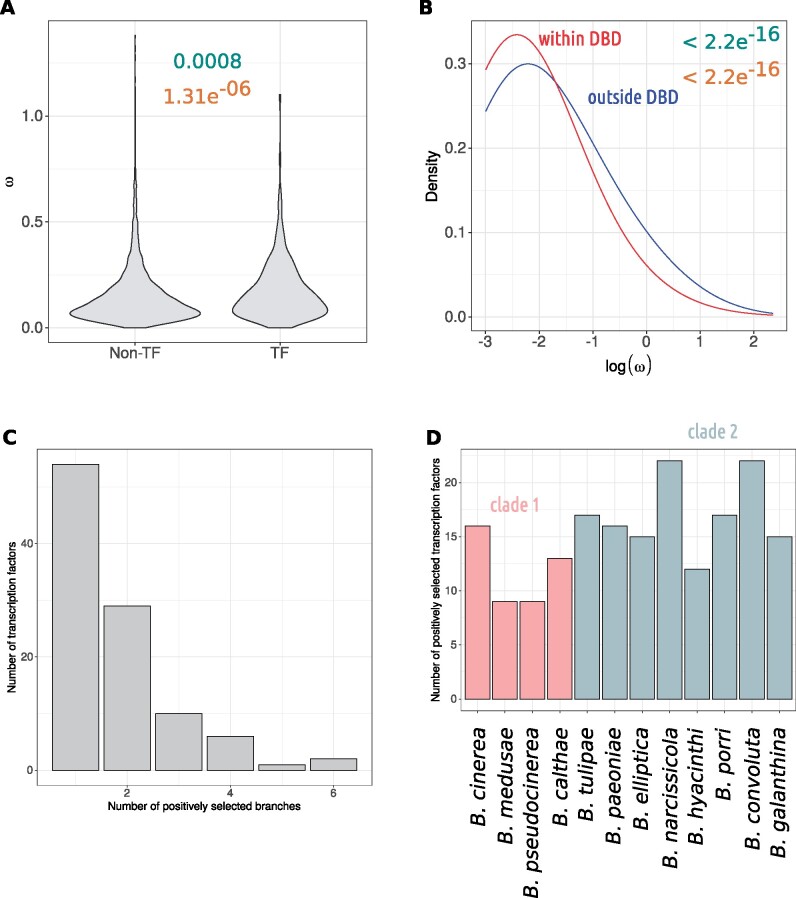
Analysis of the sites of positive selection within transcription factors. (*A*) Shows *d*_N_*/d*_S_ from the base model (*y* axis) for transcription factor (TF) or nontranscription factor (non-TF) (*x* axis) orthogroups. Violins show kernel density. The numbers above show *P* values from Student’s *t* and Wilcoxon’s tests in turquoise and orange, respectively. (*B*) The average *d*_N_*/d*_S_ within or outside of DNA-binding domains of transcription factors. The *x* axis shows the logarithm of site-wise *d*_N_*/d*_S_ Bayes empirical Bayes estimates and the *y* axis shows kernel density. The different lines show density for sites within DNA-binding domains (red) and outside DNA-binding domains (blue). The average site-wise *d*_N_*/d*_S_ value was significantly lower for sites within DNA-binding domains. The *P* values in turquoise and orange (top right) are from a Student’s *t-*test and a Wilcoxon test, respectively. (*C*) Analysis based on the aBSREL algorithm. Shows the number of transcription factor orthogroups (*y* axis) that were positively selected on one or more branches of the phylogeny. The *x* axis shows the number of branches. (*D*) Each *Botrytis* species is on the *x* axis and the *y* axis shows the corresponding number of positively selected transcription factor orthogroups for that lineage. There were no obvious differences between groups of *Botrytis* species that we could link with known lifestyle attributes. The two clades are colored in pink and gray.

To further investigate the impacts of positive selection on transcription factors, we analyzed whole sequence *ω* values and Bayes empirical Bayes (BEB) estimates of site-wise *ω* from the M1a versus M2a test. We found that, in accordance with our observation that transcription factors were more likely than other types of genes to have undergone positive selection, alignment-wide omegas for transcription factors were higher (mean *ω* = 0.18) than nontranscription factor (mean *ω* = 0.15) orthogroups (Welch’s *t P* = 0.0008; Wilcoxon’s test *P* = 1.31e^−06^) (fig. 6*A*). However, this moderate increase coincided with only a small Cohen’s *d* of −0.17 (CI = −0.26/−0.08). It is thus likely that, although transcription factors were more likely to contain at least one site under positive selection, most sites in transcription factors were not under positive selection.

On average, according to the BEB procedure, 4.15% (±6.04%) of single-copy orthogroup coding sites were under positive selection. A similar mean proportion of 4.03% (±4.89%) was found among the 411 transcription factor orthogroups. We hypothesized that positive selection may have acted on DNA-binding domains as this could have impacts on transcription factor promoter site binding and thus transcriptional regulation. However, we reject this hypothesis as sites within DNA-binding domains exhibited lower BEB estimates of *ω* than sites outside (mean *ω* = 0.25 vs. 0.17; Welch’s *t* and Wilcoxon’s test *P* < 2.2e^−16^) (fig. 6*B*). Positively selected sites were also significantly depleted within DNA-binding domains than outside (Fisher’s exact test *P* < 2.2e^−16^; odds ratio = 0.48 times expected). Furthermore, the mean proportion of positively selected transcription factor orthogroup sites outside of DNA-binding domains was 5.36% (±6.47%). In contrast, the mean proportion of positively selected sites within DNA-binding domains was 2.22% (±3.78%). The difference in proportion of coding sites under positive selection between sites within DNA-binding domains and outside was significant according to a Wilcoxon’s test (*P* < 2.2e^−16^, *W* = 38,146).

We then further investigated the number of different lineages in which the transcription factors were positively selected using the results of the aBSREL test for branch- and site-specific positive selection. We found that in most cases, transcription factors were only selected in one lineage (fig. 6*C*). We then considered the inverse of this metric, that is, how many transcription factors were under positive selection in each branch. We hypothesized that there may be a difference between how many transcription factors were positively selected in different branches depending on ecological factors such as, for example, host range. However, we did not find any obvious differences overall. Although the host generalist species *B. pseudocinerea* had the joint fewest (9) transcription factors with evidence of positive selection, this was not true of the generalist species *B. cinerea*, which had the third most (16).

Our results are consistent with enhanced positive selection in transcription factors overall but not within DNA-binding domains, suggesting conservation of promoter fidelity. The same transcription factor was seldom positively selected in multiple lineages, suggesting that selection on gene regulatory networks occurs in response to a large variety of different environmental factors on different evolutionary trajectories. Placing this into the context of our time-tree, the widespread adaptive fixations in transcription factors is likely to have occurred within the last 20 Myr, during the radiation of the *Botrytis* genus.

## Discussion

A central issue in molecular phylogenetics is accurate calibration of phylogenetic trees using fossil data. In our study, we chose three fossils that likely represent extant Ascomycete taxa, thus providing suitable minimum age constraints for different nodes in the tree. However, there is still likely to be a great deal of uncertainty in the analysis as this is the nature of molecular dating studies on poorly preserved taxa such as fungi (Beimforde et al. 2014). Therefore, the divergence times we estimated must be provided with the caveat that they are likely quite broad approximations.

The first major phylogenetic study of *Botrytis* identified two deeply diverging clades, dubbed “clade 1” and “clade 2” (Staats et al. 2005). Clade 1 contained *B. calthae*, *B. cinerea*, *B. pelargonii*, and *B. fabae*. Of these three species, only *B. calthae* and *B. cinerea* were present in our study. Our topology agrees with that of Staats et al. and several other more recent studies (Zhou et al. 2014; Saito et al. 2016; Harper et al. 2019) in that it places these two species, alongside *B. pseudocinerea* and *B. medusae*, in clade 1, and the rest of the species in clade 2. We also identified similar relationships within Clade 2 as Staats et al. For instance, *Botrytis hyacinthi* was basal to the branch leading to *B. tulipae* and *B. narcissicola* and *Botrytis galanthina* was basal to all three; the *B. elliptica* branch was then basal to this clade. We also identified the same relationship between *Botrytis porri* and *Botrytis convoluta*, which were placed in their own separate clades, and the rest of clade 2. Since these relationships have now been identified in multiple studies using different approaches, and in our study a different and more expansive set of sequences, we can be quite confident of their validity.

Our study dates the divergence between these two clades to approximately 16–18 Ma in the Miocene epoch. The middle Miocene was an age of major climatic and oceanic change. The *Botrytis* genus appears to have begun diverging around the time of the Miocene climatic optimum 15–17 Ma. This period interrupted long-term Cenozoic cooling and is markedly different from the period immediately following, which was characterized by cool temperatures and much lower sea levels (Methner et al. 2020). In our study, we were not able to estimate the ancestral geographical distribution of *Botrytis* as its members co-exist with agricultural crops all around the world (see multiple reports of various *Botrytis* species in different countries; Zhang et al. 2009; Saito et al. 2014; Li et al. 2015; Erper et al. 2019; Chen et al. 2020; Jeon et al. 2020; Mansouripour and Holmes 2020; Meeboon and Shinohara 2020; Moparthi et al. 2020). Therefore, it is not possible to precisely pinpoint ancient species radiations with respect to particular geographical events as was done, for instance, for the Chinese medicinal fungus *Sanghuangporus* (Zhu et al. 2019). To do so, it would be necessary first to estimate the centers of origin for all the *Botrytis* species, such as has been done for *Magnaporthe oryzae* (Saleh et al. 2014).

Our study shows that, in the 16–18 Myr since the most recent common ancestor of *Botrytis*, transcription factors have often been under positive selection in different lineages. In fact, as a class, transcription factors were highly enriched among positively selected genes, even after their greater than average length was accounted for. It has long been hypothesized that differences in gene regulatory networks are major drivers of phenotypic diversity (Gallego Romero et al. 2012). Early studies showed marked differences in expression profiles of a large number of genes between species, which provided empirical support for this hypothesis. For example, developmental timing of expression of a key set of genes differs between the brains of humans, rhesus macaques, and chimpanzees, possibly leading to the cognitive differences observed between these species (Somel et al. 2009). In fungi, a number of genes with different expression levels between the genera *Fusarium* and *Neurospora* were found to have significant roles in morphological development during sexual reproduction through gene knockout experiments (Trail et al. 2017).

Understanding the genetic basis of differences in gene expression between species and the role positive selection plays in their development is an important goal of molecular ecological research. There is an important distinction between polymorphisms with *cis-* and *trans*-regulatory impacts. Mutations in regulatory regions such as promoters have *cis* impacts on gene regulation as they impact adjacent genes. Mutations in transcription factors themselves likely have impacts in *trans*, as they likely affect genes in different parts of the genome. Mutations with *trans* regulatory impacts may have wider reaching effects on gene expression than *cis* effective mutations as they change the expression profiles of a greater number of genes (Tirosh et al. 2009).

In our study, we did not directly investigate *cis-* or *trans-*acting polymorphisms. However, enhanced positive selection in transcription factors is suggestive of pervasive selection of *trans* acting polymorphisms. The overall low *d*_N_*/d*_S_ ratios in transcription factors with evidence for some positively selected sites in some lineages is indicative of a longer term trend of such episodic selection. We also found that purifying selection in positively selected transcription factors was most apparent within their DNA-binding domains. This could suggest longer term maintenance of transcription factor target gene association with adaptation mostly coming from changes in how transcription factors respond external or internal stimuli. Our additional observation that signaling proteins such as protein kinases were also enriched among positively selected genes further supports this hypothesis. These observations together suggest frequent positive selection in cellular responses to stimuli impacting adaptive gene expression responses. This is in line with the previous observation that *trans* acting adaptive regulatory polymorphisms in yeast were most frequently associated with genes only expressed under certain conditions (Tirosh et al. 2009). Studies in yeast and mice have also shown that segregating expression polymorphisms are predominantly *trans* acting in natural populations (Yvert et al. 2003). Extrapolating from this, it is conceivable that among *Botrytis* populations over the last 16 Myr, standing genetic variation in *trans* acting expression polymorphisms in transcription factors was likely available for positive selection. However, follow-up studies involving comparative transcriptomics of *Botrytis* species are necessary to assess the functional significance of the polymorphisms in transcription factors we observed.

Positive selection pressure on transcription factors and signaling proteins is not a newly identified phenomenon in plant pathogenic fungi. For example, a simple estimate of *d*_N_/*d*_S_ ratios across all genes conserved between *Colletotrichum kahawae* and nonpathogenic sibling taxa turned up several protein kinases and transcription factors (Vieira et al. 2019). Echoing this study, Schweizer et al. (2018) found that the GO terms “Cellular response to extracellular stimulus,” “Response to external stimulus,” and “Cellular response to starvation” were enriched among positively selected genes in the smut fungal genus *Sporisorium*. Together with our study on *Botrytis*, these studies may indicate that cellular responses to the environment are often under positive selection in plant pathogenic fungi. By rigorously correcting for detection bias caused by alignment length, our study strengthens these observations and provides a future avenue for further study.

A final word should be said on the limitations of this study vis-à-vis genes that are not single-copy orthologs. Using conserved single-copy orthologs has the advantage that a robust species tree can be used to detect positive selection in alignments. However, it has the disadvantage that in our analyses we are likely missing approximately 30% of genes in any given species. For example, we found 7,848 single-copy orthogroups conserved in all *Botrytis* species, which is only 68% of the 11,498 genes we identified in *B. cinerea*. These kinds of genes are likely to have undergone losses, gains, or duplications in different lineages, which could be indicative of quite rapid evolutionary changes. Often proteins that are strongly involved in the arms race with plant host species, such as secreted effectors, are among this group (Brown and Tellier 2011). Thus, despite only finding limited evidence of enrichment of secreted proteins among positively selected orthogroups, we cannot draw firm conclusions on this particular class in *Botrytis* as our study lacks the power to detect positive selection among genes that have diversified through losses, gains, or duplications.

In summary, we find evidence for diversification of the *Botrytis* genus throughout the Miocene and Pliocene. During this fairly recent evolutionary period, species in this genus may have diversified gene regulatory networks through positive selection of point mutations in transcription factors and signaling proteins. This could reflect adaptation during radiation of species onto different host plants, which may present quite different environments to the pathogen.

## Materials and Methods

### DNA Extraction and Sequencing

The isolates of *B. medusae* and *B. pseudocinera* described in Harper et al. (2019), *B. medusae* B-555 and *B. pseudocinerea* Bp-362, were used in this study. The two strains were initially grown on potato dextrose agar (PDA) for two days at room temperature. To stimulate sporulation, strains were incubated for 14 days at room temperature with cycles of 12 h near-UV light and 12 h darkness. To extract DNA, spores were scraped off each plate using a sterile scalpel and placed in a 1.5 ml microcentrifuge tube. Each sample was frozen in liquid nitrogen and then ground using a sterile pestle. Genomic DNA was isolated using a BioSprint 15 instrument and BioSprint DNA Plant kit (Qiagen) according to the manufacturer’s instructions. DNA samples were subjected to both Pacific Biosciences single molecule real-time (PacBio) and Illumina sequencing at Macrogen, South Korea. PacBio sequencing was conducted on one SMRT cell 8Pac V3 per species and libraries were prepared with the DNA polymerase binding kit P6 v2. Paired end Illumina sequencing was conducted on the HiSeq 2500 system using the TruSeq Nano DNA kit library preparation method; read length was 101 base pairs.

### Genome Assembly and Annotation

In this study, we sequenced the genomes of two additional *Botrytis* taxa, *B. medusae* (Harper et al. 2019) and *B. pseudocinerea* (Walker et al. 2011) using both Pacific Biosciences Single Molecule Real-time (PacBio) and paired-end Illumina sequencing. PacBio reads were assembled using CANU version 1.4 (Koren et al. 2017). Illumina reads were aligned to PacBio genomes with Bowtie version 2.3.4.1-1 (Langmead and Salzberg 2012) and SNPs were called using the Genome Analysis Toolkit (GATK) version 4.0.10.1 (McKenna et al. 2010). The settings and submodules of GATK used are in supplementary file 1, Supplementary Material online. Insertions or deletions (InDels) called between Illumina reads and PacBio assemblies were used to generate an error-corrected reference sequence for each species. We also assembled Illumina reads separately using the A5 pipeline version 20160825 (Coil et al. 2015). We also used this pipeline to assemble Illumina reads of *B. cinerea* strain “Rasp” (Atwell et al. 2018) downloaded from the National Center for Biotechnology Information (NCBI) Sequence Read Archive (accession number SRR8835268). PacBio reads, Illumina reads, and error-corrected PacBio assemblies generated in this study are deposited in GenBank under BioProject number PRJNA692037.

We used the Illumina assemblies for comparison of orthogroups as we initially intended to assess gene duplication rates, which could be biased by assembly completeness. However, these analyses did not come to fruition and we are left with only the positive selection analyses. The results of these analyses can be considered equivalent to results based on the same data set with two Illumina sequences substituted with PacBio ones. The PacBio sequences are thus provided purely as a community resource.

Illumina assemblies of the genomes of 12 *Botrytis* spp. (the nine species described in Valero-Jiménez et al. [2019]), the two new Illumina genomes of *B. medusae* and *B. pseudocinerea*, and the *B. cinerea* strain “Rasp” (Atwell et al. 2018) were annotated using GeneMark-ES version 4 (Lomsadze et al. 2005) and Augustus version 3.3.3 (Stanke and Morgenstern 2005) trained on *B. cinerea*. Repeats were predicted using repeat masker version 4.1.0 (Smit et al. 2015). Protein sequences of the high-quality annotations of the gapless *B. cinerea* reference genome were aligned to each genome using Exonerate version 2.2.0 (Slater and Birney 2005). Evidence modeler version 1.1.1 was run on softmasked genome sequences with the following weights: GeneMark ES = 1, Augustus = 2, Exonerate alignments = 5.

### Identification of Single-Copy Orthologs and Preparation of Alignments for Phylogenetic Inference

We used Orthofinder version 2.3.3 (Emms and Kelly 2015) with default settings to identify orthologous groups of proteins among the proteomes listed in supplementary table 1, Supplementary Material online. We included proteomes of at least two representatives of each fungal class, including the nine *Botrytis* spp. described by Valero-Jiménez et al. (2019), the *B. cinerea* reference isolate B05.10 (van Kan et al. 2017) (Bioproject PRJNA264284) and new annotations of the two genome assemblies of *B. medusae* and *B. pseudocinerea*. Single-copy orthologs were derived from this analysis and aligned using Multiple Alignment using Fast Fourier Transform (MAFFT) version 7.407 (Katoh and Standley 2013). These alignments were then trimmed using Gblocks version 0.91b (Castresana 2000) with the setting “-b5 = h” to allow for half the sequences in a position to be gapped. For each single-copy ortholog, we constructed a maximum likelihood tree using Iqtree version 1.6.12 (Minh et al. 2020) with the option “-m TESTMERGE” to assess substitution model fit and partitioning scheme, possibly merging partitions, to reduce overparameterization and increase model fit. Each of the trees was manually inspected for congruence with accepted fungal taxonomy at the class level; all maximum likelihood trees for individual single-copy orthologs are in supplementary file 2, Supplementary Material online. Based on this inspection, a final set of 61 single-copy orthologs, detailed in supplementary table 2, Supplementary Material online, was retained. Fasta files containing all members of each of these 61 single-copy orthogroups are in supplementary file 3, Supplementary Material online. The individual protein alignments were concatenated into a super-alignment and used as input for Bayesian and maximum likelihood phylogenetic tree inference. The super-alignment is in supplementary file 4, Supplementary Material online.

### Inference of Fungal Phylogenetic Trees

We used both Bayesian and maximum likelihood methods to infer phylogenetic trees on which to estimate fungal divergence times. To construct a Bayesian tree, we used the software package Exabayes version 1.5 (Aberer et al. 2014) with the previously mentioned super-alignment of single-copy orthologs. We used default settings in Exabayes for this analysis and treated the alignment as partitioned. Thus, all parameters were unlinked for all partitions; amino acid state frequencies were empirically derived from the amino acid substitution matrix; branch lengths were given exponential priors with *λ* = 10; topology was given a uniform prior, that is, all topologies were equally likely; a general time reversible model of amino acid substitution was used with a uniform Dirichlet process prior over all 190 substitution rates (*ɑ* = {*i*_1_ = 1, …, *i*_190_ = 1}); rate heterogeneity was given a uniform prior across a range from zero to 200; and, a uniform Dirichlet process prior was used for the 20 state frequencies across the GTR matrix. The analysis was run for at least 100,000 generations until the average SD of split frequencies was less than 5%. To construct a maximum likelihood tree, we used Iqtree. For this, we used the options “-m TESTMERGE” and “-bsam GENESITE.” The second option specifies bootstrap resampling of partitions, then sites within partitions; this procedure was used to generate 1,000 bootstrap topologies.

### Estimation of Divergence Times Using Fossil Calibrations

We used the program MCMCtree from the software package phylogenetic analysis by maximum likelihood (PAML) version 4.9j (Yang 1997) to estimate divergence times on a fixed topology.

We performed this analysis on a super-alignment of coding sequences corresponding to the amino acid sequences used for initial tree inference. Partitioning of nucleotide sequences in divergence time dating may reduce the variance of estimates of node ages compared with unpartitioned data. However, partitioning has the potential to introduce bias into estimates if partitions are not independent realizations of the rate-drift process (Zhu et al. 2015). Therefore, we analyzed both partitioned and unpartitioned data and present the results from both analyses.

For both partitioned and unpartitioned data sets, mutation rates were estimated with the PAML package baseml under the strict clock general time reversible model. For the unpartitioned data set, this estimated rate was used as the prior for mean mutation rate across branches under a relaxed clock model for MCMC divergence time dating. For the partitioned data set, initial partitions were merged into partitions with similar mutation rates based on the scheme in supplementary table 3, Supplementary Material online. The rate prior for the partitioned MCMC divergence time estimate was the average of all the partition rates initially estimated using baseml.

We used a relaxed-clock model to estimate rates across lineages and the K80 (Kimura 1980) nucleotide substitution model. We used a gamma Dirichlet prior for locus rates with the parameters *ɑ*_μ_ = 4.8, *β*_μ_ = 10, and *ɑ* = 1 for the unpartitioned data set. For the partitioned data set, *ɑ*_μ_ was 4.4. The mean mutation rate is specified by *ɑ*_μ_/*β*_μ_ and the lower the value of *ɑ*_μ_, the less informative the prior. Thus, we chose a prior based on the average rates calculated using baseml but gave it quite a diffuse distribution. We also used a gamma Dirichlet prior on the variability of rates across branches. For this prior, we set *ɑ* = 10 and *β* = 100 to specify the shape and scale, respectively.

We used three fossil calibrations to estimate divergence times, based on an analysis by Beimforde et al. (2014). These included the fossil taxa *Aspergillus collembolorum* (Dörfelt and Schmidt 2005), *Petropus brachyphylli* (van der Ham and Dortangs 2005), and *Paleopyrenomycites devonicus* (Taylor et al. 1999, 2005). All distributions on node ages were skew normal with scale and shape parameters (*ɑ* and *β*) of 0.5 and 2, respectively.

Similarly to Beimforde et al. (2014), we assigned the following calibrations based on the estimated ages of these fossils: the common ancestor of the Pezizomycotina was given an offset of 400 Myr based on the estimated age of *Paleopyrenomycites devonicus*; the common ancestor of the *Aspergillus* was given an offset of 35 Myr based on the estimated age of *A. collembolorum*; and, the common ancestor of the Capnodiales was given an offset of 100 Myr based on the estimated age of *Petropus brachyphylli*. We used the approximate likelihood method to estimate divergence times (dos Reis and Yang 2011). We ran the analysis for 10,000 generations after 5,000 generations of burn-in, with a sampling frequency of two. For partitioned and unpartitioned data sets, we ran two independent Markov Chains each and inspected parameter estimates for convergence.

### Genome-Wide Analysis of Positive Selection

We performed tests of positive selection on all single-copy orthologs present across all species in the *Botrytis* genus. We derived these from a second run of Orthofinder using only the *Botrytis* species, including the new genome sequences and the *B. cinerea* isolate “rasp” (see previous section on assemblies). We used PAML and tools from the HyPhy suite (available at https://www.hyphy.org/ at the time of writing) to fit different models of sequence evolution to single-copy orthologs. Before fitting these models, we used the program Sliding Window Alignment Masker for PAML version 31-03-14 (Harrison et al. 2014) to mask low-quality alignment regions with a sliding window size of 15 and a threshold of five nonsynonymous substitutions. We did this because spurious gene predictions may lead to spurious alignments, which is a known cause of false-positives in maximum likelihood tests of positive selection.

The PAML site model comparisons M1a versus M2a and M7 versus M8 were performed using codeml. Models M1a and M7 are null models restricting site-wise *ω* to one or less. Their corresponding alternate models, M2a and M8, estimate *ω* with free parameters, thus allowing inference of positive selection across sites. To determine whether the alternate models fit significantly better than their null models, we used a χ^2^ test of the likelihood ratio between the alternate and null models with two degrees of freedom. The degrees of freedom correspond to the difference in free parameters between the null and alternate models. The Bayes empirical Bayes procedure was used to determine which sites had undergone positive selection.

The BUSTED algorithm (Murrell et al. 2015) was used to identify episodic positive selection in specific lineages. This algorithm fits a model that varies stochastically across branches and sites and compares it with a restricted null model. Again, a likelihood ratio test determines whether positive selection has acted on a codon sequence. It is theoretically more sensitive than the PAML site models as it allows *ω* to vary between branches and sites.

Finally, to test branch-specific positive selection, we used the aBSREL algorithm (Smith et al. 2015). This algorithm uses a model selection procedure for each branch to determine the proportion of sites per branch that have undergone positive selection. Again, comparing a more complex model with a null model, it is able to test for significance of positive selection in specific branches.

### InterPro Annotation of Orthogroups and Functional Enrichment Analyses

All *Botrytis* proteomes were annotated using InterPro scan version 5.46-81 (Jones et al. 2014) with default settings. If any members of an orthogroup contained an InterPro domain, that domain was added to the list of domains present in that orthogroup. Each unique InterPro domain in the set of all InterPro domains annotated to single-copy orthogroups was tested for functional enrichment among positively selected orthogroups. This was done using a Fisher’s exact test.

### Identification of Specific Orthogroup Categories

Orthogroups were annotated as transcription factors using the InterPro DNA-binding domains outlined in Shelest (2017). If an orthogroup was annotated with one of these InterPro domains, it was designated as a transcription factor orthogroup. Carbohydrate active enzymes (CAZymes) were identified in all *Botrytis* proteomes using dbCAN version 2 (Yin et al. 2012). Secreted proteins were identified in all *Botrytis* proteomes using signalP version 5 (Almagro Armenteros et al. 2019). As with the InterPro domains, if any members of an orthogroup had a secretion signal, that orthogroup was designated as “secreted” and if any members of an orthogroup was annotated as a CAZyme, that orthogroup was designated as a “CAZyme” orthogroup.

### Other Statistical Tests

To assess whether the classes “CAZyme,” “transcription factor,” or “secreted protein” were enriched among positively selected genes, we used Fisher’s exact test. To adjust this estimate for protein length, we used logistic regression. The dependent variable was the binary status of “positively selected” or “not positively selected” and the predictors were protein length and membership of one of the three classes. We fit a null model that included only protein length and an alternate model for each class that included protein length and membership of the class. We then used a χ^2^ test, with one degree of freedom, of the likelihood ratio between alternate and null models to determine whether alternate models fit the data better. The null hypothesis here is that detection of positive selection is dependent upon only alignment length. If membership of a particular class significantly improves model fit, it suggests that protein length alone is not sufficient to predict whether an alignment was classed as positively selected. Thus, even if a particular class has a longer alignment length than average, significant improvement of fit in the null model suggests that its enrichment among positively selected orthogroups is biologically relevant and not just an artifact.

We further controlled the effects on protein length for our transcription factor estimates using the following randomization test. For each randomization, we selected a random set of orthogroup alignments, without replacement, with lengths between the lowest and highest for transcription factor orthogroup alignments. This sampling was weighted in the following way: 2/3 of the sample was drawn from between the lowest and highest transcription factor orthogroup alignment lengths and 1/3 of the sample was drawn from above the mean and below the highest transcription factor alignment length. This approximated the skewed distribution of alignment lengths of transcription factor orthogroups. We then recorded the proportion of these orthogroups that were positively selected according to the likelihood ratio tests from PAML and HyPhy. We made 1,000 such randomizations and compared the actual proportion of transcription factor alignments that were positively selected with the distribution of proportions from the randomizations.

To test for differences in length and *ω* from the base PAML model between sets of orthogroups, we used both Welch’s *t*-tests and Wilcoxon’s tests. Wherever we conducted the same tests for the same metrics more than once, we corrected for false discovery rate using the Benjamini–Hochberg procedure. This was also the case for the logistic regression models mentioned in the previous paragraph. For some comparisons between means, we also assessed Cohen’s *d* in the R package effsize version 0.1.8. As well as performing likelihood ratio tests between different models, we also compared AIC values using the base R function “AIC.” All statistical tests were performed in R version 3.6.3.

## Supplementary Material

Supplementary data are available at *Genome Biology and Evolution* online.

## Supplementary Material

evab167_Supplementary_Data

## Data Availability

The Pacific Biosciences and Illumina sequencing reads to generate assemblies of the species *Botrytis medusae* and *Botrytis pseudocinerea* and the assemblies themselves are available in NCBI under BioProject Number PRJNA692037. All other data are publicly available and accessible from NCBI or JGI using the accession numbers mentioned in supplementary table 1, Supplementary Material online.
