# Supplementary material for: Positive Selection of Transcription Factors Is a Prominent Feature of the Evolution of a Plant Pathogenic Genus Originating in the Miocene
Source: Genome Biol Evol. 2021 Jul 20;13(8):evab167. doi: 10.1093/gbe/evab167 (PMC8379374; doi:10.1093/gbe/evab167)
Supplement: evab167_Supplementary_Data [file evab167_supplementary_data.zip › Supplementary Information legends_210221.docx]

**Supplementary Table 1 Species used to construct fungal phylogenies, including accession numbers and database source; protein sequences were used to build the phylogenies.**

**Supplementary Table 2 A list of single copy orthogroups identified from the OrthoFinder analysis and an indication of whether or not they were used for the phylogeny (see supplementary File 2 for individual phylogenies from each orthogroup for the basis of these decisions).**

**Supplementary Table 3 Partition merging scheme for gene sequences in the super-alignment that was used for running the partitioned divergence dating analysis.**

**Supplementary Table 4 Assembly statistics for the Illumina and Illumina + PacBio assemblies of *Botrytis pseudocinerea* and *Botrytis medusae*.**

**Supplementary Figure 1 Convergence of MCMC chains from the divergence dating analysis from two independent runs. A** The x axis shows the MCMC generation and the y axis the log likelihood of the joint posterior distribution. **B** The x axis shows MCMC generation and the y axis the node age in hundreds of millions of years; each colour represents a different node and the first five nodes are presented showing adequate MCMC convergence.

**Supplementary Figure 2 Time tree based on the partitioned data set.** The time scale on the x axis is in millions of years. The different coloured blocks at the bottom of the figure represent different geological periods, Cambrian (C.), Ordovician (O.), Silurian (S.), Devonian (D.), Carboniferous (C.), Permian (P.), Triassic (T.), Jurassic (J.), Cretaceous (C.), Paleogene (P.) and Neogene (N.), and the geological eons Proterozoic and Phanerozoic. The grey blocks emanating from the base of the plot divide the tree into different geological periods. Density plots above nodes in the tree are the full posterior distributions of node ages from the Markov Chain. Four major events are labelled in turquoise. In brackets are the dates estimated by Beimforde et al. (2014) using five fossil calibrations. *Botrytis* species names are highlighted in blue and node ages in the *Botrytis* clade are labelled in dark red. This tree is one of two trees built using unpartitioned nucleotide data. Both runs of the MCMC are in Supplementary File 5. Supplementary Figure 1 shows trace plots of tree log likelihood and the first 10 node ages for this tree. The same plot based on the tree built from the unpartitioned alignment is in Figure 3. All node age estimates were consistent with radiation of *Botrytis* starting in the Pliocene and ending in the Miocene.

**Supplementary Figure 3 The number of secreted proteins in predicted in each of the *Botrytis* genomes.** Species name is no the x axis and number of secreted proteins predicted using signal P is on the Y axis.
