# Supplementary material for: Positive Selection of Transcription Factors Is a Prominent Feature of the Evolution of a Plant Pathogenic Genus Originating in the Miocene
Source: Genome Biol Evol. 2021 Jul 20;13(8):evab167. doi: 10.1093/gbe/evab167 (PMC8379374; doi:10.1093/gbe/evab167)
Supplement: evab167_Supplementary_Data [file evab167_supplementary_data.zip › FileS1.rtf]

#!/usr/bin/env bash

# The following commands were used to run GATK and Picard to correct PacBio contig errors using SNPs. 
# To use these commands, paths should be changed to reflect the local file system.
# These commands were used for both new genome assemblies. 

PICARDPATH=/usr/local/src
GATKPATH=/usr/local/src
BAMDIR=../data/
REFERENCEDIR=../data/Bpseudocinerea-auto/

# First, clean the bam file.
java -jar $PICARDPATH/picard.jar \
	CleanSam \
	I=$BAMDIR/Bp-vs-Bp.bam \
	OUTPUT=$BAMDIR/Bp-vs-Bp.map.clean.bam

# Create a sequence dictionary for the BAM.
java -jar $PICARDPATH/picard.jar \
	CreateSequenceDictionary \
	R=$REFERENCEDIR/Bpseudocinerea.contigs.fasta \
	O=$REFERENCEDIR/Bpseudocinerea.contigs.dict

# Sort the BAM.
java -jar $PICARDPATH/picard.jar \
	SortSam \
	I=$BAMDIR/Bp-vs-Bp.map.clean.bam \
	O=$BAMDIR/Bp-vs-Bp.map.clean.sorted.bam \
	SORT_ORDER=coordinate

# Mark duplicate reads.
java -jar $PICARDPATH/picard.jar \
	MarkDuplicates \
	I=$BAMDIR/Bp-vs-Bp.map.clean.sorted.bam \
	O=$BAMDIR/Bp-vs-Bp.map.clean.sorted.md.bam \
	M=$BAMDIR/metrics.txt

# Add read groups to the file.
java -jar $PICARDPATH/picard.jar \
	AddOrReplaceReadGroups \
	I=$BAMDIR/Bp-vs-Bp.map.clean.sorted.md.bam \
	O=$BAMDIR/Bp-vs-Bp.map.clean.sorted.md.rg.bam \
	RGID=Bp-Illumina \
	RGLB=Bp-Illumina-lib \
	RGPL=Illumina \
	RGPU=Bp-unit \
	RGSM=Bp-sample

# Index the cleaned and sorted BAM.
samtools \
	index \
	$BAMDIR/Bp-vs-Bp.map.clean.sorted.md.rg.bam

# Index the Canu-assembled PacBio contigs.
samtools \
	faidx \
	$REFERENCEDIR/Bpseudocinerea.contigs.fasta

# Call haplotypes between the PacBio assembly and Illumina reads.
java -jar $GATKPATH/GenomeAnalysisTK.jar \
	-R $REFERENCEDIR/Bpseudocinerea.contigs.fasta \
	-T HaplotypeCaller \
	-I $BAMDIR/Bp-vs-Bp.map.clean.sorted.md.rg.bam \
	--genotyping_mode DISCOVERY \
	-stand_call_conf 30 \
	-o Bp-vs-Bp.vcf

# Select SNP variants.
java -jar $GATKPATH/GenomeAnalysisTK.jar \
	-T SelectVariants \
	-R $REFERENCEDIR/Bpseudocinerea.contigs.fasta \
	-V Bp-vs-Bp.vcf \
	-selectType SNP \
	-o Bp-vs-Bp.raw.vcf

# Filter SNP variants using the '--filterExpression' below.
java -jar $GATKPATH/GenomeAnalysisTK.jar \
	-T VariantFiltration \
	-R $REFERENCEDIR/Bpseudocinerea.contigs.fasta \
	-V Bp-vs-Bp.raw.vcf \
	--filterExpression "QD < 2.0 || AF < 1.0 || FS > 60.0 || MQ < 40.0 || MQRankSum < -12.5 || ReadPosRankSum < -8.0" \
	--filterName "snp_filter" \
	-o Bp-vs-Bp.filt.vcf

# Select InDels.
java -jar $GATKPATH/GenomeAnalysisTK.jar \
	-T SelectVariants \
	-R $REFERENCEDIR/Bpseudocinerea.contigs.fasta \
	-V Bp-vs-Bp.vcf \
	-selectType INDEL \
	-o Bp-vs-Bp.indels.raw.vcf

# Filter the InDels using the '--filterExpression' below.
java -jar $GATKPATH/GenomeAnalysisTK.jar \
	-T VariantFiltration \
	-R $REFERENCEDIR/Bpseudocinerea.contigs.fasta \
	-V Bp-vs-Bp.indels.raw.vcf \
	--filterExpression "QD < 2.0 || AF < 1.0 || FS > 200.0 || ReadPosRankSum < -20.0" \
	--filterName "indel_filter" \
	-o Bp-vs-Bp.indels.filt.vcf

# Combine the SNP and InDel variants.
java -jar $GATKPATH/GenomeAnalysisTK.jar \
	-T CombineVariants \
	-R $REFERENCEDIR/Bpseudocinerea.contigs.fasta \
	--variant:a Bp-vs-Bp.indels.filt.vcf \
	--variant:b Bp-vs-Bp.filt.vcf \
	-o Bp-vs-Bp.highqual.vcf \
	-genotypeMergeOptions PRIORITIZE \
	-priority a,b 

# Recalibrate base scores based on the high quality variants filtered from above.
java -jar $GATKPATH/GenomeAnalysisTK.jar \
	-T BaseRecalibrator \
	-R $REFERENCEDIR/Bpseudocinerea.contigs.fasta \
	-I $BAMDIR/Bp-vs-Bp.map.clean.sorted.md.rg.bam \
	-knownSites Bp-vs-Bp.highqual.vcf \
	-o Bp-vs-Bp.recal.txt

# Recalibrate the scores in the BAM file.
java -jar $GATKPATH/GenomeAnalysisTK.jar \
	-T PrintReads \
	-R $REFERENCEDIR/Bpseudocinerea.contigs.fasta \
	-I $BAMDIR/Bp-vs-Bp.map.clean.sorted.md.rg.bam \
	-BQSR Bp-vs-Bp.recal.txt \
	-o $BAMDIR/Bp-vs-Bp.recal.bam

# Call haplotypes with recalibrated BAM.
java -jar $GATKPATH/GenomeAnalysisTK.jar \
	-R $REFERENCEDIR/Bpseudocinerea.contigs.fasta \
	-T HaplotypeCaller \
	-I $BAMDIR/Bp-vs-Bp.recal.bam \
	--genotyping_mode DISCOVERY \
	-stand_call_conf 30 \
	-o Bp-vs-Bp.recal.vcf

# Select InDels from the recalibrated calls.
java -jar $GATKPATH/GenomeAnalysisTK.jar \
	-T SelectVariants \
	-R $REFERENCEDIR/Bpseudocinerea.contigs.fasta \
	-V Bp-vs-Bp.recal.vcf \
	-selectType INDEL \
	-o Bp-vs-Bp.indels.recal.vcf

# Make an alternate reference from the InDel calls against the PacBio contigs.
java -jar $GATKPATH/GenomeAnalysisTK.jar \
	-T FastaAlternateReferenceMaker \
	-R $REFERENCEDIR/Bpseudocinerea.contigs.fasta \
	-V Bp-vs-Bp.indels.recal.vcf \
	-o Bpseudocinerea.corrected.contigs.fasta
