# Supplementary material for: Positive Selection of Transcription Factors Is a Prominent Feature of the Evolution of a Plant Pathogenic Genus Originating in the Miocene
Source: Genome Biol Evol. 2021 Jul 20;13(8):evab167. doi: 10.1093/gbe/evab167 (PMC8379374; doi:10.1093/gbe/evab167)
Supplement: evab167_Supplementary_Data [file evab167_supplementary_data.zip › FileS2.pdf]

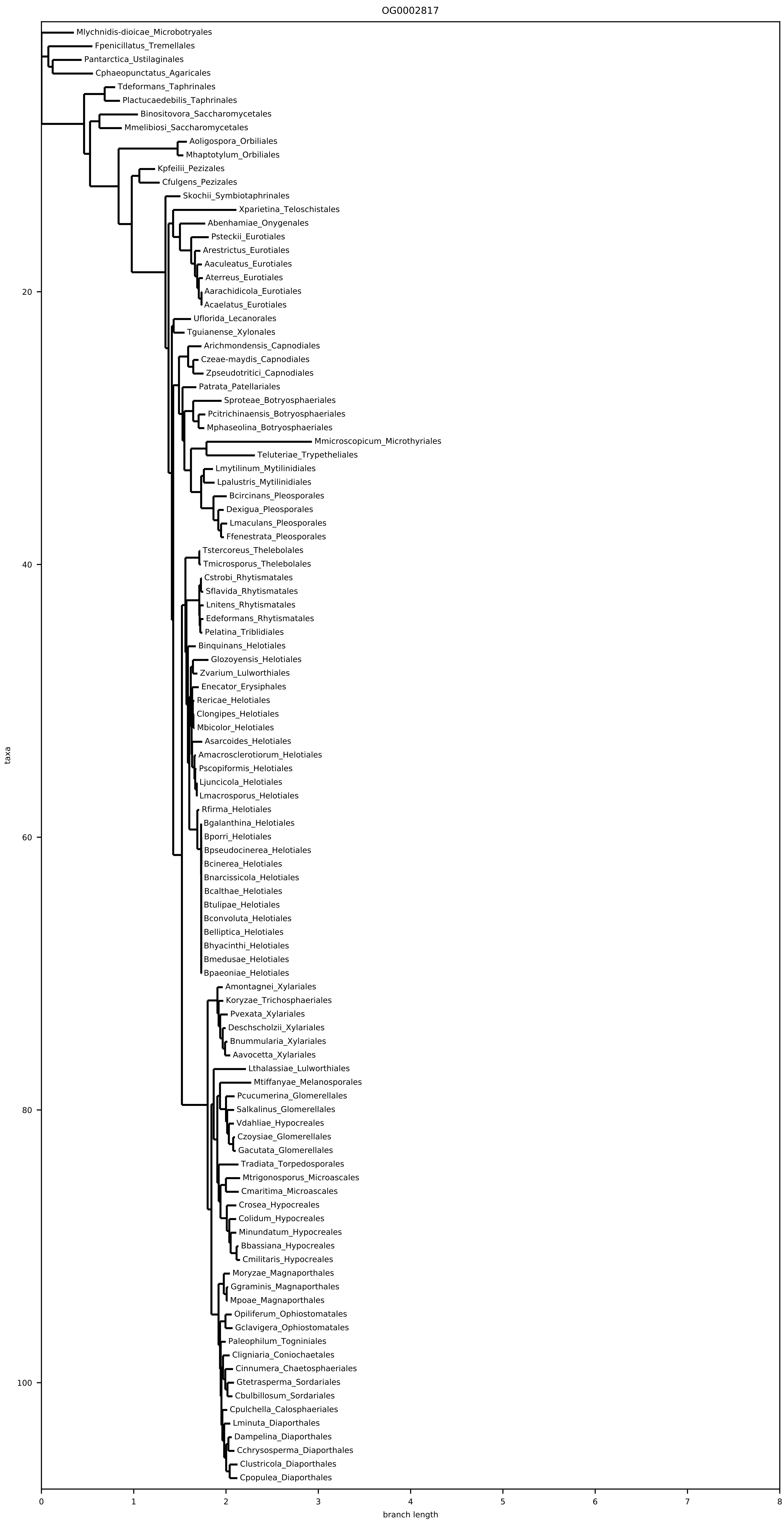
OG0002817
Mlychnidis-dioicae_Microbotryales
Fpenicillatus_Tremellales
Pantarctica_Ustilaginales
Cphaeopunctatus_Agaricales
Tdeformans_Taphrinales
Plactucaedebilis_Taphrinales
Binositovora_Saccharomycetales
Mmelibiosi_Saccharomycetales
Aoligospora_Orbiliales
Mhaptotylum_Orbiliales
Kpfeilii_Pezizales
Cfulgens_Pezizales
Skochii_Symbiotaphrinales
Xparietina_Teloschistales
Abenhamiae_Onygenales
Psteckii_Eurotiales
Arestrictus_Eurotiales
Aaculeatus_Eurotiales
Aterreus_Eurotiales
Aarachidicola_Eurotiales
Acaelatus_Eurotiales
Uflorida_Lecanorales
Tguianense_Xylonales
Arichmondensis_Capnodiales
Czeae-maydis_Capnodiales
Zpseudotritici_Capnodiales
Patrata_Patellariales
Sproteae_Botryosphaeriales
Pcitrichinaensis_Botryosphaeriales
Mphaseolina_Botryosphaeriales
Mmicroscopicum_Microthyriales
Teluteriae_Trypetheliales
Lmytilinum_Mytilinidiales
Lpalustris_Mytilinidiales
Bcircinans_Pleosporales
Dexigua_Pleosporales
Lmaculans_Pleosporales
Ffenestrata_Pleosporales
Tstercoreus_Thelebolales
Tmicrosporus_Thelebolales
Cstrobi_Rhytismatales
Sflavida_Rhytismatales
Lnitens_Rhytismatales
Edeformans_Rhytismatales
Pelatina_Triblidiales
Binquinans_Helotiales
Glozoyensis_Helotiales
Zvarium_Lulworthiales
Enecator_Erysiphales
Rericae_Helotiales
Clongipes_Helotiales
Mbicolor_Helotiales
Asarcoides_Helotiales
Amacrosclerotiorum_Helotiales
Pscopiformis_Helotiales
Ljuncicola_Helotiales
Lmacrosporus_Helotiales
Rfirma_Helotiales
Bgalanthina_Helotiales
Bporri_Helotiales
Bpseudocinerea_Helotiales
Bcinerea_Helotiales
Bnarcissicola_Helotiales
Bcalthae_Helotiales
Btulipae_Helotiales
Bconvoluta_Helotiales
Belliptica_Helotiales
Bhyacinthi_Helotiales
Bmedusae_Helotiales
Bpaeoniae_Helotiales
Amontagnei_Xylariales
Koryzae_Trichosphaeriales
Pvexata_Xylariales
Deschscholzii_Xylariales
Bnummularia_Xylariales
Aavocetta_Xylariales
Lthalassiae_Lulworthiales
Mtiffanyae_Melanosporales
Pcucumerina_Glomerellales
Salkalinus_Glomerellales
Vdahliae_Hypocreales
Czoysiae_Glomerellales
Gacutata_Glomerellales
Tradiata_Torpedosporales
Mtrigonosporus_Microascales
Cmaritima_Microascales
Crosea_Hypocreales
Colidum_Hypocreales
Minundatum_Hypocreales
Bbassiana_Hypocreales
Cmilitaris_Hypocreales
Moryzae_Magnaporthales
Ggraminis_Magnaporthales
Mpoae_Magnaporthales
Opiliferum_Ophiostomatales
Gclavigera_Ophiostomatales
Paleophilum_Togniniales
Cligniaria_Coniochaetales
Cinnumera_Chaetosphaeriales
Gtetrasperma_Sordariales
Cbulbillosum_Sordariales
Cpulchella_Calosphaeriales
Lminuta_Diaporthales
Dampelina_Diaporthales
Cchrysosperma_Diaporthales
Clustricola_Diaporthales
Cpopulea_Diaporthales
taxa
20
40
60
80
100
0
1
2
3
4
5
6
7
8
branch length

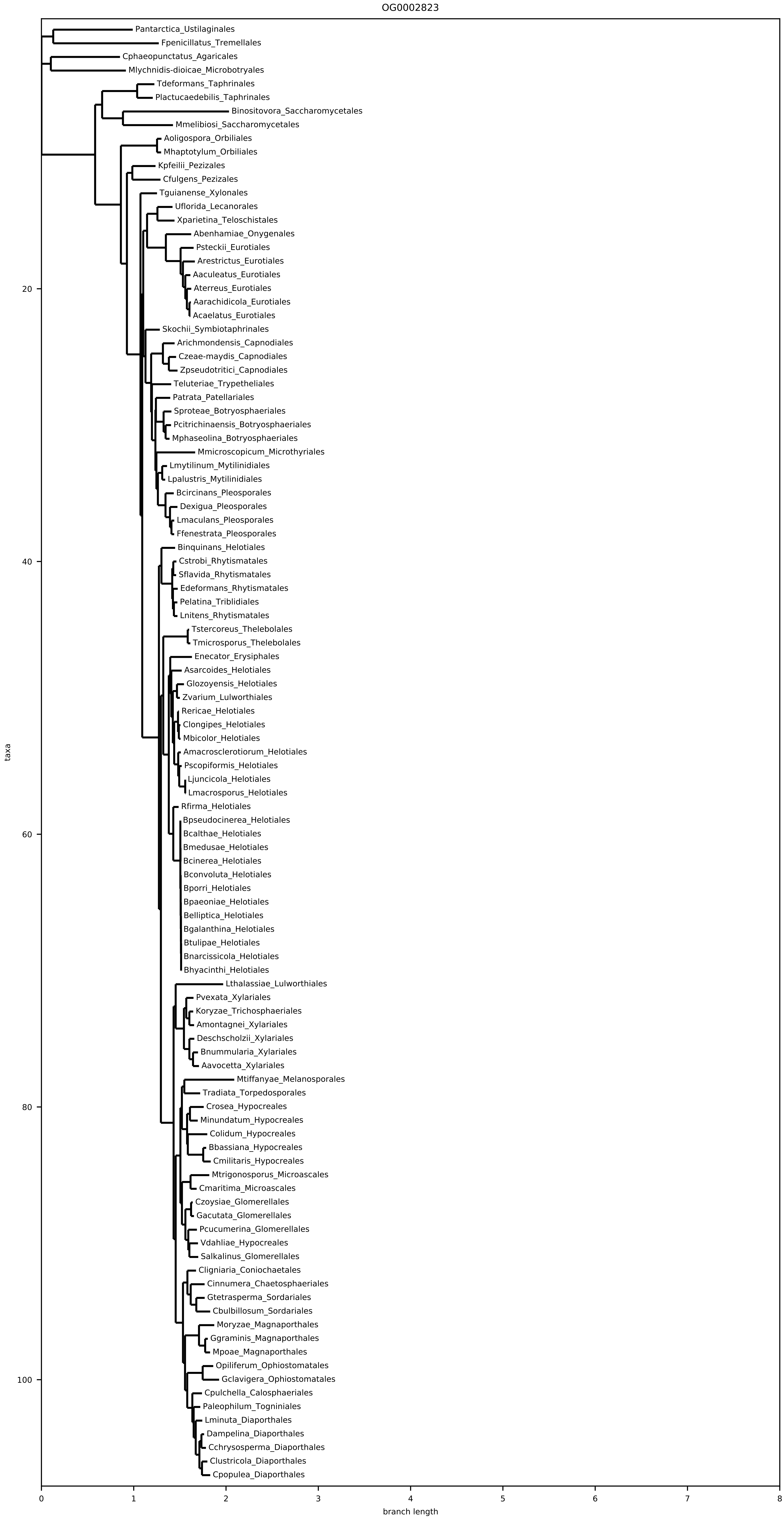

OG0002823
Pantarctica_Ustilaginales
Fpenicillatus_Tremellales
Cphaeopunctatus_Agaricales
Mlychnidis-dioicae_Microbotryales
Tdeformans_Taphrinales
Plactucaedebilis_Taphrinales
Binositovora_Saccharomycetales
Mmelibiosi_Saccharomycetales
Aoligospora_Orbiliales
Mhaptotylum_Orbiliales
Kpfeilii_Pezizales
Cfulgens_Pezizales
Tguianense_Xylonales
Uflorida_Lecanorales
Xparietina_Teloschistales
Abenhamiae_Onygenales
Psteckii_Eurotiales
Arestrictus_Eurotiales
Aaculeatus_Eurotiales
Aterreus_Eurotiales
Aarachidicola_Eurotiales
Acaelatus_Eurotiales
Skochii_Symbiotaphrinales
Arichmondensis_Capnodiales
Czeae-maydis_Capnodiales
Zpseudotritici_Capnodiales
Teluteriae_Trypetheliales
Patrata_Patellariales
Sproteae_Botryosphaeriales
Pcitrichinaensis_Botryosphaeriales
Mphaseolina_Botryosphaeriales
Mmicroscopicum_Microthyriales
Lmytilinum_Mytilinidiales
Lpalustris_Mytilinidiales
Bcircinans_Pleosporales
Dexigua_Pleosporales
Lmaculans_Pleosporales
Ffenestrata_Pleosporales
Binquinans_Helotiales
Cstrobi_Rhytismatales
Sflavida_Rhytismatales
Edeformans_Rhytismatales
Pelatina_Triblidiales
Lnitens_Rhytismatales
Tstercoreus_Thelebolales
Tmicrosporus_Thelebolales
Enecator_Erysiphales
Asarcoides_Helotiales
Glozoyensis_Helotiales
Zvarium_Lulworthiales
Rericae_Helotiales
Clongipes_Helotiales
Mbicolor_Helotiales
Amacrosclerotiorum_Helotiales
Pscopiformis_Helotiales
Ljuncicola_Helotiales
Lmacrosporus_Helotiales
Rfirma_Helotiales
Bpseudocinerea_Helotiales
Bcalthae_Helotiales
Bmedusae_Helotiales
Bcinerea_Helotiales
Bconvoluta_Helotiales
Bporri_Helotiales
Bpaeoniae_Helotiales
Belliptica_Helotiales
Bgalanthina_Helotiales
Btulipae_Helotiales
Bnarcissicola_Helotiales
Bhyacinthi_Helotiales
Lthalassiae_Lulworthiales
Pvexata_Xylariales
Koryzae_Trichosphaeriales
Amontagnei_Xylariales
Deschscholzii_Xylariales
Bnummularia_Xylariales
Aavocetta_Xylariales
Mtiffanyae_Melanosporales
Tradiata_Torpedosporales
Crosea_Hypocreales
Minundatum_Hypocreales
Colidum_Hypocreales
Bbassiana_Hypocreales
Cmilitaris_Hypocreales
Mtrigonosporus_Microascales
Cmaritima_Microascales
Czoysiae_Glomerellales
Gacutata_Glomerellales
Pcucumerina_Glomerellales
Vdahliae_Hypocreales
Salkalinus_Glomerellales
Cligniaria_Coniochaetales
Cinnumera_Chaetosphaeriales
Gtetrasperma_Sordariales
Cbulbillosum_Sordariales
Moryzae_Magnaporthales
Ggraminis_Magnaporthales
Mpoae_Magnaporthales
Opiliferum_Ophiostomatales
Gclavigera_Ophiostomatales
Cpulchella_Calosphaeriales
Paleophilum_Togniniales
Lminuta_Diaporthales
Dampelina_Diaporthales
Cchrysosperma_Diaporthales
Clustricola_Diaporthales
Cpopulea_Diaporthales
taxa
20
40
60
80
100
0
1
2
3
4
5
6
7
8
branch length

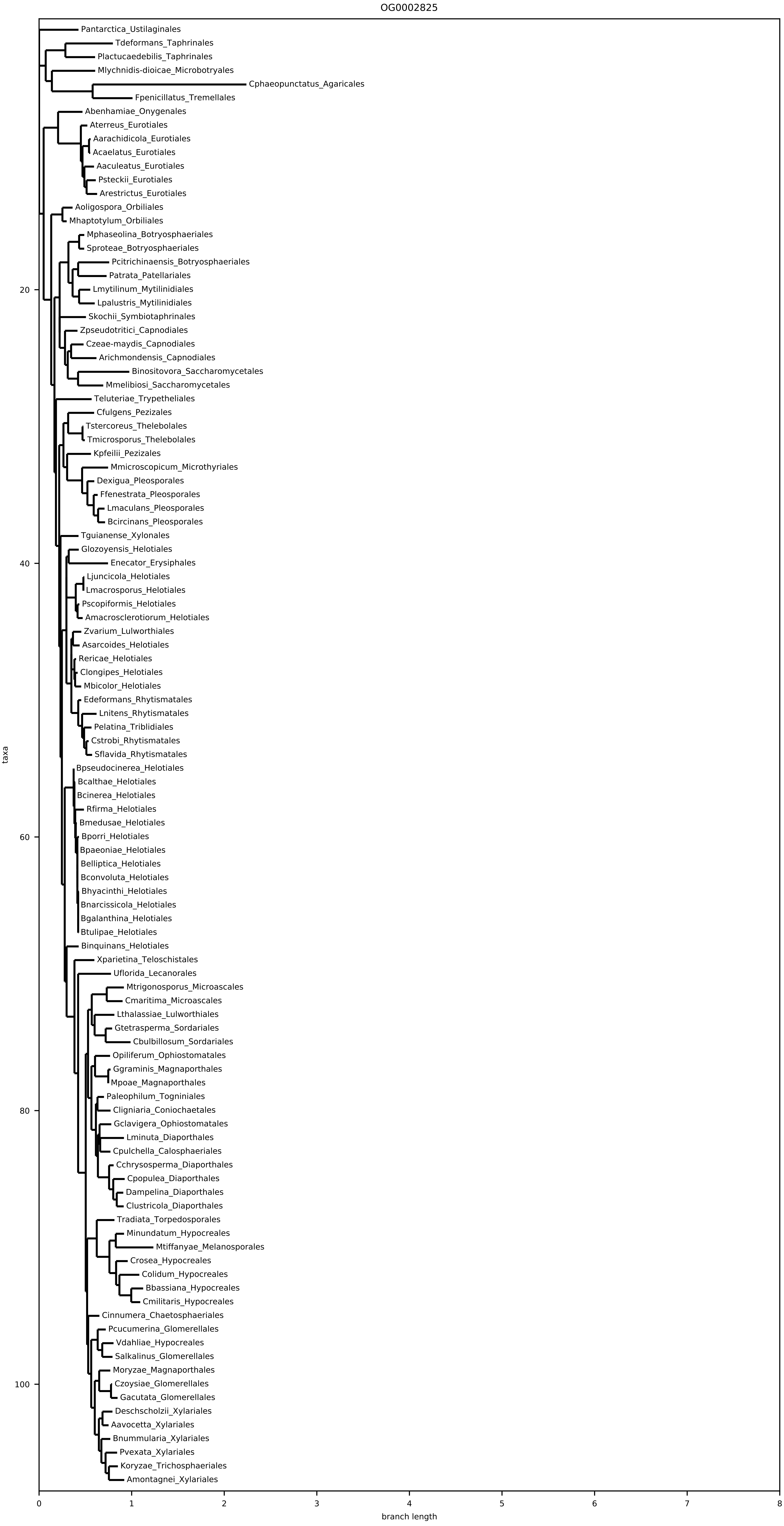
OG0002825
Pantarctica_Ustilaginales
Tdeformans_Taphrinales
Plactucaedebilis_Taphrinales
Mlychnidis-dioicae_Microbotryales
Cphaeopunctatus_Agaricales
Fpenicillatus_Tremellales
Abenhamiae_Onygenales
Aterreus_Eurotiales
Aarachidicola_Eurotiales
Acaelatus_Eurotiales
Aaculeatus_Eurotiales
Psteckii_Eurotiales
Arestrictus_Eurotiales
Aoligospora_Orbiliales
Mhaptotylum_Orbiliales
Mphaseolina_Botryosphaeriales
Sproteae_Botryosphaeriales
Pcitrichinaensis_Botryosphaeriales
Patrata_Patellariales
Lmytilinum_Mytilinidiales
Lpalustris_Mytilinidiales
Skochii_Symbiotaphrinales
Zpseudotritici_Capnodiales
Czeae-maydis_Capnodiales
Arichmondensis_Capnodiales
Binositovora_Saccharomycetales
Mmelibiosi_Saccharomycetales
Teluteriae_Trypetheliales
Cfulgens_Pezizales
Tstercoreus_Thelebolales
Tmicrosporus_Thelebolales
Kpfeilii_Pezizales
Mmicroscopicum_Microthyriales
Dexigua_Pleosporales
Ffenestrata_Pleosporales
Lmaculans_Pleosporales
Bcircinans_Pleosporales
Tguianense_Xylonales
Glozoyensis_Helotiales
Enecator_Erysiphales
Ljuncicola_Helotiales
Lmacrosporus_Helotiales
Pscopiformis_Helotiales
Amacrosclerotiorum_Helotiales
Zvarium_Lulworthiales
Asarcoides_Helotiales
Rericae_Helotiales
Clongipes_Helotiales
Mbicolor_Helotiales
Edeformans_Rhytismatales
Lnitens_Rhytismatales
Pelatina_Triblidiales
Cstrobi_Rhytismatales
Sflavida_Rhytismatales
Bpseudocinerea_Helotiales
Bcalthae_Helotiales
Bcinerea_Helotiales
Rfirma_Helotiales
Bmedusae_Helotiales
Bporri_Helotiales
Bpaeoniae_Helotiales
Belliptica_Helotiales
Bconvoluta_Helotiales
Bhyacinthi_Helotiales
Bnarcissicola_Helotiales
Bgalanthina_Helotiales
Btulipae_Helotiales
Binquinans_Helotiales
Xparietina_Teloschistales
Uflorida_Lecanorales
Mtrigonosporus_Microascales
Cmaritima_Microascales
Lthalassiae_Lulworthiales
Gtetrasperma_Sordariales
Cbulbillosum_Sordariales
Opiliferum_Ophiostomatales
Ggraminis_Magnaporthales
Mpoae_Magnaporthales
Paleophilum_Togniniales
Cligniaria_Coniochaetales
Gclavigera_Ophiostomatales
Lminuta_Diaporthales
Cpulchella_Calosphaeriales
Cchrysosperma_Diaporthales
Cpopulea_Diaporthales
Dampelina_Diaporthales
Clustricola_Diaporthales
Tradiata_Torpedosporales
Minundatum_Hypocreales
Mtiffanyae_Melanosporales
Crosea_Hypocreales
Colidum_Hypocreales
Bbassiana_Hypocreales
Cmilitaris_Hypocreales
Cinnumera_Chaetosphaeriales
Pcucumerina_Glomerellales
Vdahliae_Hypocreales
Salkalinus_Glomerellales
Moryzae_Magnaporthales
Czoysiae_Glomerellales
Gacutata_Glomerellales
Deschscholzii_Xylariales
Aavocetta_Xylariales
Bnummularia_Xylariales
Pvexata_Xylariales
Koryzae_Trichosphaeriales
Amontagnei_Xylariales
taxa
20
40
60
80
100
0
1
2
3
4
5
6
7
8
branch length

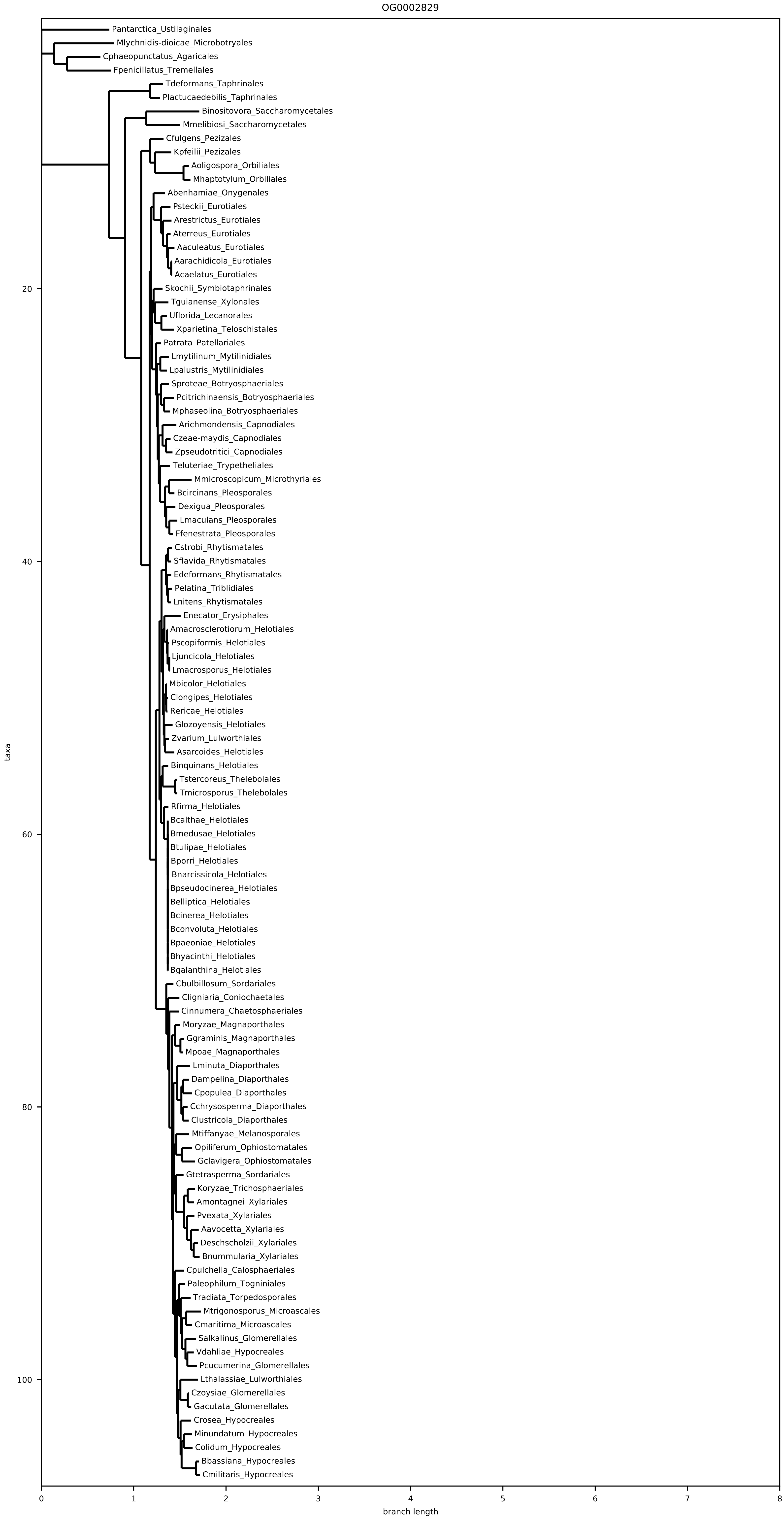
OG0002829
Pantarctica_Ustilaginales
Mlychnidis-dioicae_Microbotryales
Cphaeopunctatus_Agaricales
Fpenicillatus_Tremellales
Tdeformans_Taphrinales
Plactucaedebilis_Taphrinales
Binositovora_Saccharomycetales
Mmelibiosi_Saccharomycetales
Cfulgens_Pezizales
Kpfeilii_Pezizales
Aoligospora_Orbiliales
Mhaptotylum_Orbiliales
Abenhamiae_Onygenales
Psteckii_Eurotiales
Arestrictus_Eurotiales
Aterreus_Eurotiales
Aaculeatus_Eurotiales
Aarachidicola_Eurotiales
Acaelatus_Eurotiales
Skochii_Symbiotaphrinales
Tguianense_Xylonales
Uflorida_Lecanorales
Xparietina_Teloschistales
Patrata_Patellariales
Lmytilinum_Mytilinidiales
Lpalustris_Mytilinidiales
Sproteae_Botryosphaeriales
Pcitrichinaensis_Botryosphaeriales
Mphaseolina_Botryosphaeriales
Arichmondensis_Capnodiales
Czeae-maydis_Capnodiales
Zpseudotritici_Capnodiales
Teluteriae_Trypetheliales
Mmicroscopicum_Microthyriales
Bcircinans_Pleosporales
Dexigua_Pleosporales
Lmaculans_Pleosporales
Ffenestrata_Pleosporales
Cstrobi_Rhytismatales
Sflavida_Rhytismatales
Edeformans_Rhytismatales
Pelatina_Triblidiales
Lnitens_Rhytismatales
Enecator_Erysiphales
Amacrosclerotiorum_Helotiales
Pscopiformis_Helotiales
Ljuncicola_Helotiales
Lmacrosporus_Helotiales
Mbicolor_Helotiales
Clongipes_Helotiales
Rericae_Helotiales
Glozoyensis_Helotiales
Zvarium_Lulworthiales
Asarcoides_Helotiales
Binquinans_Helotiales
Tstercoreus_Thelebolales
Tmicrosporus_Thelebolales
Rfirma_Helotiales
Bcalthae_Helotiales
Bmedusae_Helotiales
Btulipae_Helotiales
Bporri_Helotiales
Bnarcissicola_Helotiales
Bpseudocinerea_Helotiales
Belliptica_Helotiales
Bcinerea_Helotiales
Bconvoluta_Helotiales
Bpaeoniae_Helotiales
Bhyacinthi_Helotiales
Bgalanthina_Helotiales
Cbulbillosum_Sordariales
Cligniaria_Coniochaetales
Cinnumera_Chaetosphaeriales
Moryzae_Magnaporthales
Ggraminis_Magnaporthales
Mpoae_Magnaporthales
Lminuta_Diaporthales
Dampelina_Diaporthales
Cpopulea_Diaporthales
Cchrysosperma_Diaporthales
Clustricola_Diaporthales
Mtiffanyae_Melanosporales
Opiliferum_Ophiostomatales
Gclavigera_Ophiostomatales
Gtetrasperma_Sordariales
Koryzae_Trichosphaeriales
Amontagnei_Xylariales
Pvexata_Xylariales
Aavocetta_Xylariales
Deschscholzii_Xylariales
Bnummularia_Xylariales
Cpulchella_Calosphaeriales
Paleophilum_Togniniales
Tradiata_Torpedosporales
Mtrigonosporus_Microascales
Cmaritima_Microascales
Salkalinus_Glomerellales
Vdahliae_Hypocreales
Pcucumerina_Glomerellales
Lthalassiae_Lulworthiales
Czoysiae_Glomerellales
Gacutata_Glomerellales
Crosea_Hypocreales
Minundatum_Hypocreales
Colidum_Hypocreales
Bbassiana_Hypocreales
Cmilitaris_Hypocreales
taxa
20
40
60
80
100
0
1
2
3
4
5
6
7
8
branch length

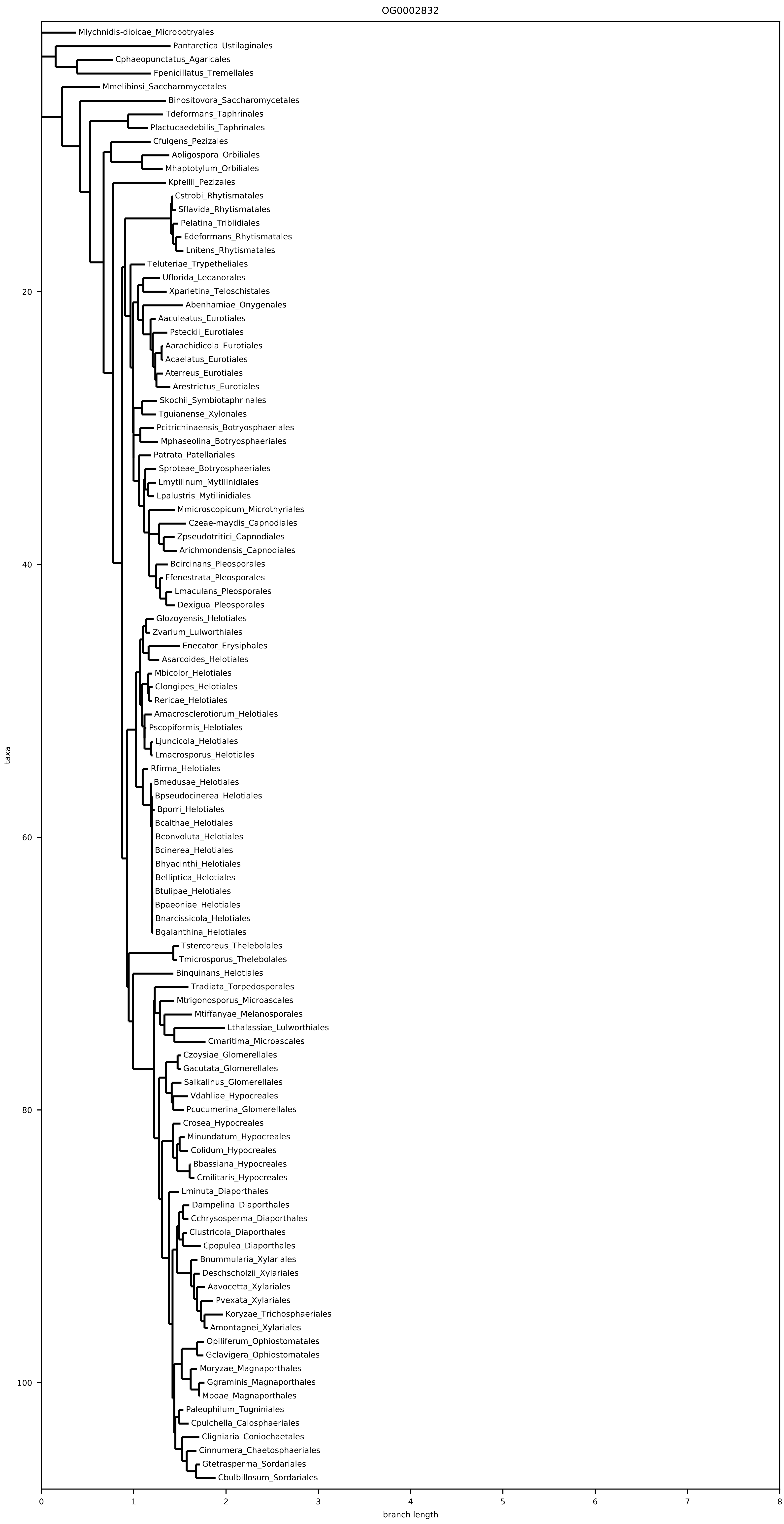

OG0002832
Mlychnidis-dioicae_Microbotryales
Pantarctica_Ustilaginales
Cphaeopunctatus_Agaricales
Fpenicillatus_Tremellales
Mmelibiosi_Saccharomycetales
Binositovora_Saccharomycetales
Tdeformans_Taphrinales
Plactucaedebilis_Taphrinales
Cfulgens_Pezizales
Aoligospora_Orbiliales
Mhaptotylum_Orbiliales
Kpfeilii_Pezizales
Cstrobi_Rhytismatales
Sflavida_Rhytismatales
Pelatina_Triblidiales
Edeformans_Rhytismatales
Lnitens_Rhytismatales
Teluteriae_Trypetheliales
Uflorida_Lecanorales
Xparietina_Teloschistales
Abenhamiae_Onygenales
Aaculeatus_Eurotiales
Psteckii_Eurotiales
Aarachidicola_Eurotiales
Acaelatus_Eurotiales
Aterreus_Eurotiales
Arestrictus_Eurotiales
Skochii_Symbiotaphrinales
Tguianense_Xylonales
Pcitrichinaensis_Botryosphaeriales
Mphaseolina_Botryosphaeriales
Patrata_Patellariales
Sproteae_Botryosphaeriales
Lmytilinum_Mytilinidiales
Lpalustris_Mytilinidiales
Mmicroscopicum_Microthyriales
Czeae-maydis_Capnodiales
Zpseudotritici_Capnodiales
Arichmondensis_Capnodiales
Bcircinans_Pleosporales
Ffenestrata_Pleosporales
Lmaculans_Pleosporales
Dexigua_Pleosporales
Glozoyensis_Helotiales
Zvarium_Lulworthiales
Enecator_Erysiphales
Asarcoides_Helotiales
Mbicolor_Helotiales
Clongipes_Helotiales
Rericae_Helotiales
Amacrosclerotiorum_Helotiales
Pscopiformis_Helotiales
Ljuncicola_Helotiales
Lmacrosporus_Helotiales
Rfirma_Helotiales
Bmedusae_Helotiales
Bpseudocinerea_Helotiales
Bporri_Helotiales
Bcalthae_Helotiales
Bconvoluta_Helotiales
Bcinerea_Helotiales
Bhyacinthi_Helotiales
Belliptica_Helotiales
Btulipae_Helotiales
Bpaeoniae_Helotiales
Bnarcissicola_Helotiales
Bgalanthina_Helotiales
Tstercoreus_Thelebolales
Tmicrosporus_Thelebolales
Binquinans_Helotiales
Tradiata_Torpedosporales
Mtrigonosporus_Microascales
Mtiffanyae_Melanosporales
Lthalassiae_Lulworthiales
Cmaritima_Microascales
Czoysiae_Glomerellales
Gacutata_Glomerellales
Salkalinus_Glomerellales
Vdahliae_Hypocreales
Pcucumerina_Glomerellales
Crosea_Hypocreales
Minundatum_Hypocreales
Colidum_Hypocreales
Bbassiana_Hypocreales
Cmilitaris_Hypocreales
Lminuta_Diaporthales
Dampelina_Diaporthales
Cchrysosperma_Diaporthales
Clustricola_Diaporthales
Cpopulea_Diaporthales
Bnummularia_Xylariales
Deschscholzii_Xylariales
Aavocetta_Xylariales
Pvexata_Xylariales
Koryzae_Trichosphaeriales
Amontagnei_Xylariales
Opiliferum_Ophiostomatales
Gclavigera_Ophiostomatales
Moryzae_Magnaporthales
Ggraminis_Magnaporthales
Mpoae_Magnaporthales
Paleophilum_Togniniales
Cpulchella_Calosphaeriales
Cligniaria_Coniochaetales
Cinnumera_Chaetosphaeriales
Gtetrasperma_Sordariales
Cbulbillosum_Sordariales
taxa
20
40
60
80
100
0
1
2
3
4
5
6
7
8
branch length

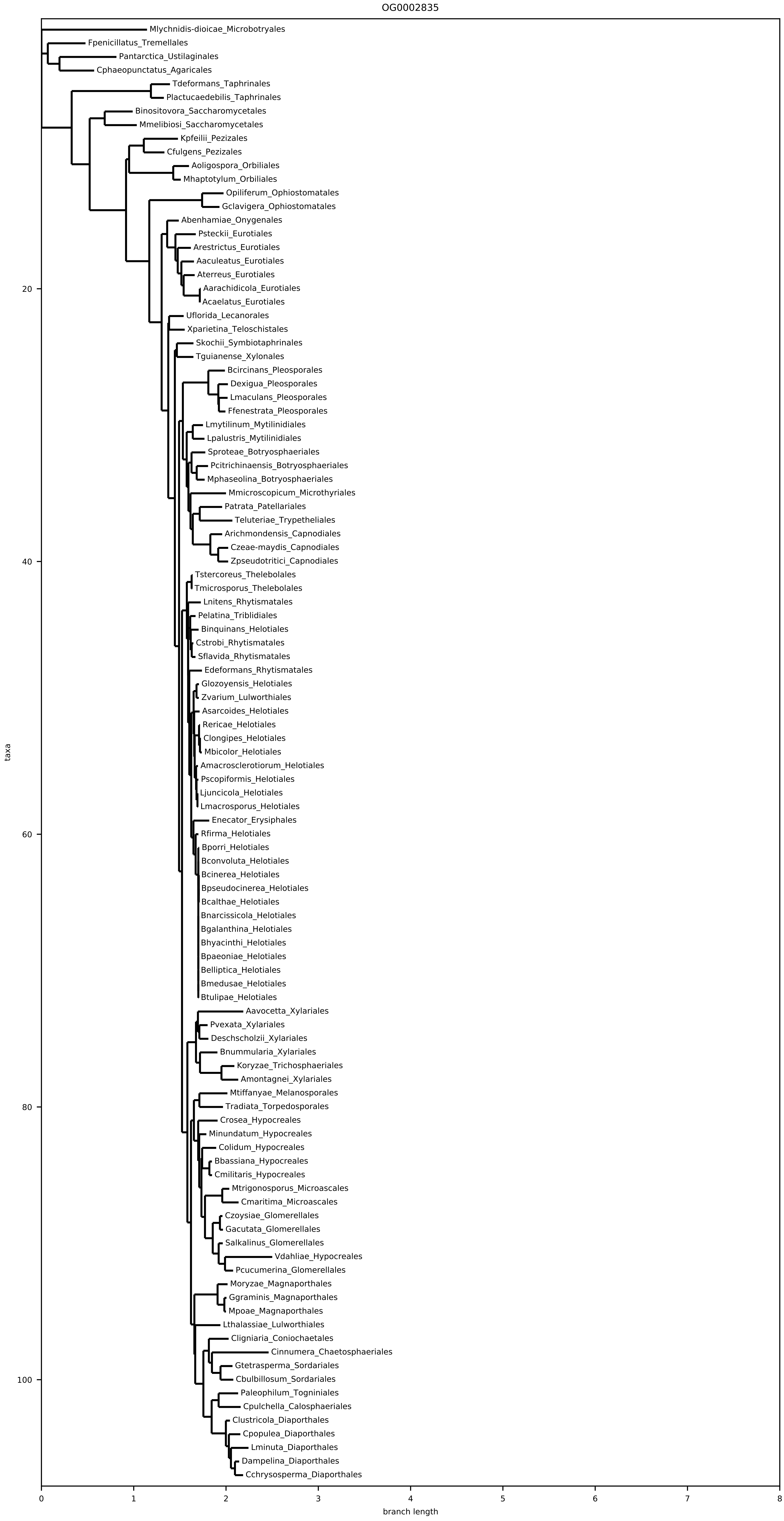

OG0002835
Mlychnidis-dioicae_Microbotryales
Fpenicillatus_Tremellales
Pantarctica_Ustilaginales
Cphaeopunctatus_Agaricales
Tdeformans_Taphrinales
Plactucaedebilis_Taphrinales
Binositovora_Saccharomycetales
Mmelibiosi_Saccharomycetales
Kpfeilii_Pezizales
Cfulgens_Pezizales
Aoligospora_Orbiliales
Mhaptotylum_Orbiliales
Opiliferum_Ophiostomatales
Gclavigera_Ophiostomatales
Abenhamiae_Onygenales
Psteckii_Eurotiales
Arestrictus_Eurotiales
Aaculeatus_Eurotiales
Aterreus_Eurotiales
Aarachidicola_Eurotiales
Acaelatus_Eurotiales
Uflorida_Lecanorales
Xparietina_Teloschistales
Skochii_Symbiotaphrinales
Tguianense_Xylonales
Bcircinans_Pleosporales
Dexigua_Pleosporales
Lmaculans_Pleosporales
Ffenestrata_Pleosporales
Lmytilinum_Mytilinidiales
Lpalustris_Mytilinidiales
Sproteae_Botryosphaeriales
Pcitrichinaensis_Botryosphaeriales
Mphaseolina_Botryosphaeriales
Mmicroscopicum_Microthyriales
Patrata_Patellariales
Teluteriae_Trypetheliales
Arichmondensis_Capnodiales
Czeae-maydis_Capnodiales
Zpseudotritici_Capnodiales
Tstercoreus_Thelebolales
Tmicrosporus_Thelebolales
Lnitens_Rhytismatales
Pelatina_Triblidiales
Binquinans_Helotiales
Cstrobi_Rhytismatales
Sflavida_Rhytismatales
Edeformans_Rhytismatales
Glozoyensis_Helotiales
Zvarium_Lulworthiales
Asarcoides_Helotiales
Rericae_Helotiales
Clongipes_Helotiales
Mbicolor_Helotiales
Amacrosclerotiorum_Helotiales
Pscopiformis_Helotiales
Ljuncicola_Helotiales
Lmacrosporus_Helotiales
Enecator_Erysiphales
Rfirma_Helotiales
Bporri_Helotiales
Bconvoluta_Helotiales
Bcinerea_Helotiales
Bpseudocinerea_Helotiales
Bcalthae_Helotiales
Bnarcissicola_Helotiales
Bgalanthina_Helotiales
Bhyacinthi_Helotiales
Bpaeoniae_Helotiales
Belliptica_Helotiales
Bmedusae_Helotiales
Btulipae_Helotiales
Aavocetta_Xylariales
Pvexata_Xylariales
Deschscholzii_Xylariales
Bnummularia_Xylariales
Koryzae_Trichosphaeriales
Amontagnei_Xylariales
Mtiffanyae_Melanosporales
Tradiata_Torpedosporales
Crosea_Hypocreales
Minundatum_Hypocreales
Colidum_Hypocreales
Bbassiana_Hypocreales
Cmilitaris_Hypocreales
Mtrigonosporus_Microascales
Cmaritima_Microascales
Czoysiae_Glomerellales
Gacutata_Glomerellales
Salkalinus_Glomerellales
Vdahliae_Hypocreales
Pcucumerina_Glomerellales
Moryzae_Magnaporthales
Ggraminis_Magnaporthales
Mpoae_Magnaporthales
Lthalassiae_Lulworthiales
Cligniaria_Coniochaetales
Cinnumera_Chaetosphaeriales
Gtetrasperma_Sordariales
Cbulbillosum_Sordariales
Paleophilum_Togniniales
Cpulchella_Calosphaeriales
Clustricola_Diaporthales
Cpopulea_Diaporthales
Lminuta_Diaporthales
Dampelina_Diaporthales
Cchrysosperma_Diaporthales
taxa
20
40
60
80
100
0
1
2
3
4
5
6
7
8
branch length

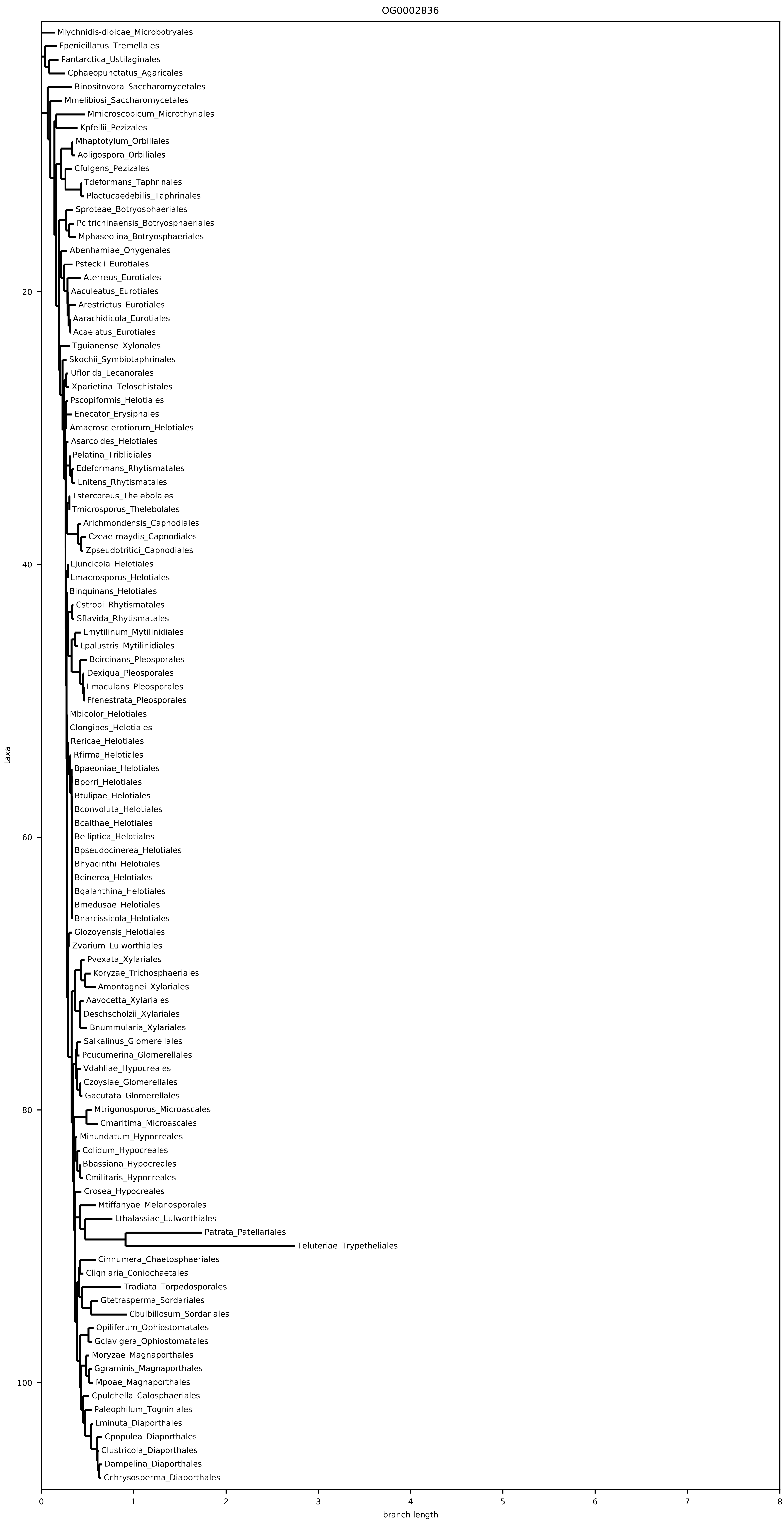
OG0002836
Mlychnidis-dioicae_Microbotryales
Fpenicillatus_Tremellales
Pantarctica_Ustilaginales
Cphaeopunctatus_Agaricales
Binositovora_Saccharomycetales
Mmelibiosi_Saccharomycetales
Mmicroscopicum_Microthyriales
Kpfeilii_Pezizales
Mhaptotylum_Orbiliales
Aoligospora_Orbiliales
Cfulgens_Pezizales
Tdeformans_Taphrinales
Plactucaedebilis_Taphrinales
Sproteae_Botryosphaeriales
Pcitrichinaensis_Botryosphaeriales
Mphaseolina_Botryosphaeriales
Abenhamiae_Onygenales
Psteckii_Eurotiales
Aterreus_Eurotiales
Aaculeatus_Eurotiales
Arestrictus_Eurotiales
Aarachidicola_Eurotiales
Acaelatus_Eurotiales
Tguianense_Xylonales
Skochii_Symbiotaphrinales
Uflorida_Lecanorales
Xparietina_Teloschistales
Pscopiformis_Helotiales
Enecator_Erysiphales
Amacrosclerotiorum_Helotiales
Asarcoides_Helotiales
Pelatina_Triblidiales
Edeformans_Rhytismatales
Lnitens_Rhytismatales
Tstercoreus_Thelebolales
Tmicrosporus_Thelebolales
Arichmondensis_Capnodiales
Czeae-maydis_Capnodiales
Zpseudotritici_Capnodiales
Ljuncicola_Helotiales
Lmacrosporus_Helotiales
Binquinans_Helotiales
Cstrobi_Rhytismatales
Sflavida_Rhytismatales
Lmytilinum_Mytilinidiales
Lpalustris_Mytilinidiales
Bcircinans_Pleosporales
Dexigua_Pleosporales
Lmaculans_Pleosporales
Ffenestrata_Pleosporales
Mbicolor_Helotiales
Clongipes_Helotiales
Rericae_Helotiales
Rfirma_Helotiales
Bpaeoniae_Helotiales
Bporri_Helotiales
Btulipae_Helotiales
Bconvoluta_Helotiales
Bcalthae_Helotiales
Belliptica_Helotiales
Bpseudocinerea_Helotiales
Bhyacinthi_Helotiales
Bcinerea_Helotiales
Bgalanthina_Helotiales
Bmedusae_Helotiales
Bnarcissicola_Helotiales
Glozoyensis_Helotiales
Zvarium_Lulworthiales
Pvexata_Xylariales
Koryzae_Trichosphaeriales
Amontagnei_Xylariales
Aavocetta_Xylariales
Deschscholzii_Xylariales
Bnummularia_Xylariales
Salkalinus_Glomerellales
Pcucumerina_Glomerellales
Vdahliae_Hypocreales
Czoysiae_Glomerellales
Gacutata_Glomerellales
Mtrigonosporus_Microascales
Cmaritima_Microascales
Minundatum_Hypocreales
Colidum_Hypocreales
Bbassiana_Hypocreales
Cmilitaris_Hypocreales
Crosea_Hypocreales
Mtiffanyae_Melanosporales
Lthalassiae_Lulworthiales
Patrata_Patellariales
Teluteriae_Trypetheliales
Cinnumera_Chaetosphaeriales
Cligniaria_Coniochaetales
Tradiata_Torpedosporales
Gtetrasperma_Sordariales
Cbulbillosum_Sordariales
Opiliferum_Ophiostomatales
Gclavigera_Ophiostomatales
Moryzae_Magnaporthales
Ggraminis_Magnaporthales
Mpoae_Magnaporthales
Cpulchella_Calosphaeriales
Paleophilum_Togniniales
Lminuta_Diaporthales
Cpopulea_Diaporthales
Clustricola_Diaporthales
Dampelina_Diaporthales
Cchrysosperma_Diaporthales
taxa
20
40
60
80
100
0
1
2
3
4
5
6
7
8
branch length

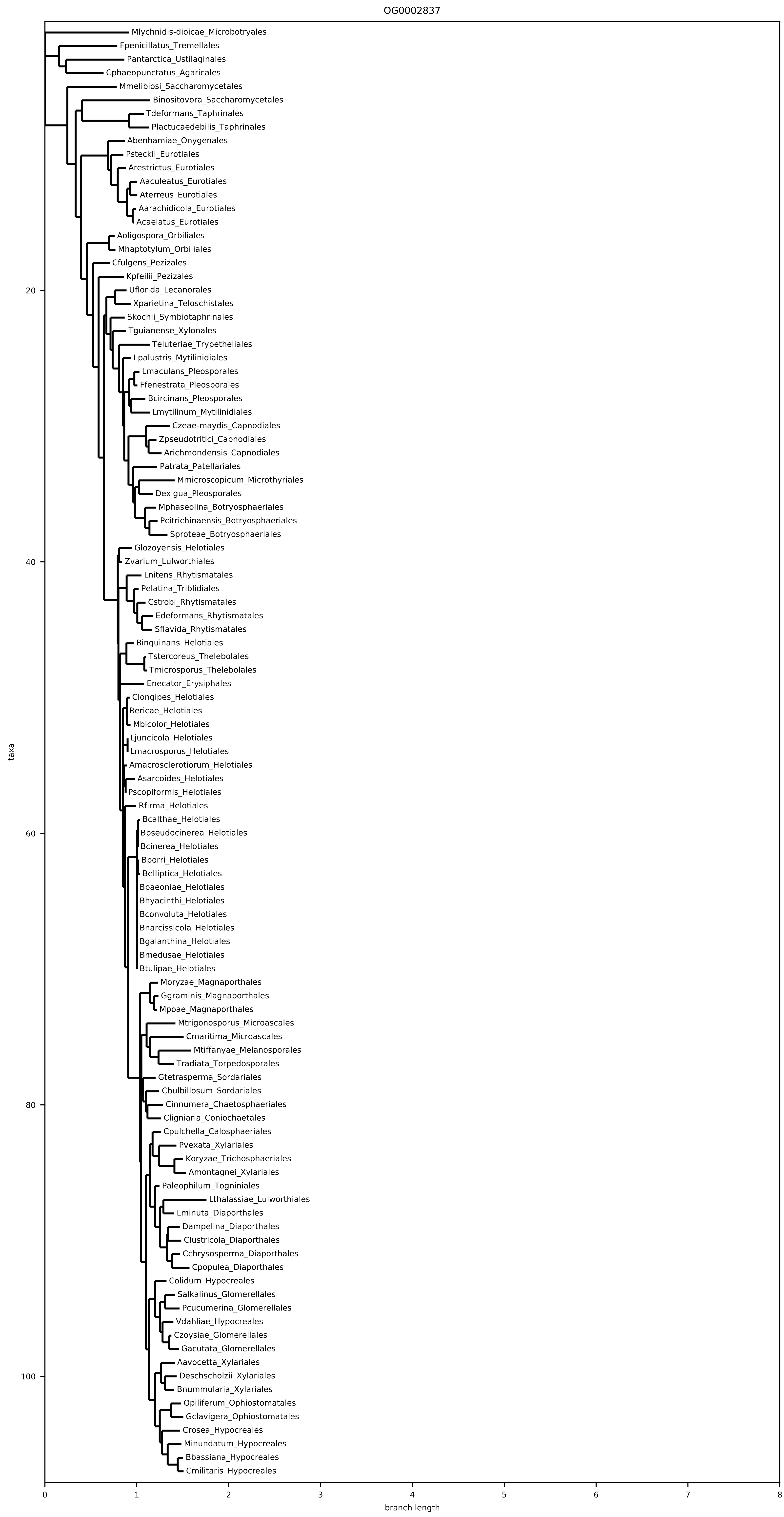

OG0002837
Mlychnidis-dioicae_Microbotryales
Fpenicillatus_Tremellales
Pantarctica_Ustilaginales
Cphaeopunctatus_Agaricales
Mmelibiosi_Saccharomycetales
Binositovora_Saccharomycetales
Tdeformans_Taphrinales
Plactucaedebilis_Taphrinales
Abenhamiae_Onygenales
Psteckii_Eurotiales
Arestrictus_Eurotiales
Aaculeatus_Eurotiales
Aterreus_Eurotiales
Aarachidicola_Eurotiales
Acaelatus_Eurotiales
Aoligospora_Orbiliales
Mhaptotylum_Orbiliales
Cfulgens_Pezizales
Kpfeilii_Pezizales
Uflorida_Lecanorales
Xparietina_Teloschistales
Skochii_Symbiotaphrinales
Tguianense_Xylonales
Teluteriae_Trypetheliales
Lpalustris_Mytilinidiales
Lmaculans_Pleosporales
Ffenestrata_Pleosporales
Bcircinans_Pleosporales
Lmytilinum_Mytilinidiales
Czeae-maydis_Capnodiales
Zpseudotritici_Capnodiales
Arichmondensis_Capnodiales
Patrata_Patellariales
Mmicroscopicum_Microthyriales
Dexigua_Pleosporales
Mphaseolina_Botryosphaeriales
Pcitrichinaensis_Botryosphaeriales
Sproteae_Botryosphaeriales
Glozoyensis_Helotiales
Zvarium_Lulworthiales
Lnitens_Rhytismatales
Pelatina_Triblidiales
Cstrobi_Rhytismatales
Edeformans_Rhytismatales
Sflavida_Rhytismatales
Binquinans_Helotiales
Tstercoreus_Thelebolales
Tmicrosporus_Thelebolales
Enecator_Erysiphales
Clongipes_Helotiales
Rericae_Helotiales
Mbicolor_Helotiales
Ljuncicola_Helotiales
Lmacrosporus_Helotiales
Amacrosclerotiorum_Helotiales
Asarcoides_Helotiales
Pscopiformis_Helotiales
Rfirma_Helotiales
Bcalthae_Helotiales
Bpseudocinerea_Helotiales
Bcinerea_Helotiales
Bporri_Helotiales
Belliptica_Helotiales
Bpaeoniae_Helotiales
Bhyacinthi_Helotiales
Bconvoluta_Helotiales
Bnarcissicola_Helotiales
Bgalanthina_Helotiales
Bmedusae_Helotiales
Btulipae_Helotiales
Moryzae_Magnaporthales
Ggraminis_Magnaporthales
Mpoae_Magnaporthales
Mtrigonosporus_Microascales
Cmaritima_Microascales
Mtiffanyae_Melanosporales
Tradiata_Torpedosporales
Gtetrasperma_Sordariales
Cbulbillosum_Sordariales
Cinnumera_Chaetosphaeriales
Cligniaria_Coniochaetales
Cpulchella_Calosphaeriales
Pvexata_Xylariales
Koryzae_Trichosphaeriales
Amontagnei_Xylariales
Paleophilum_Togniniales
Lthalassiae_Lulworthiales
Lminuta_Diaporthales
Dampelina_Diaporthales
Clustricola_Diaporthales
Cchrysosperma_Diaporthales
Cpopulea_Diaporthales
Colidum_Hypocreales
Salkalinus_Glomerellales
Pcucumerina_Glomerellales
Vdahliae_Hypocreales
Czoysiae_Glomerellales
Gacutata_Glomerellales
Aavocetta_Xylariales
Deschscholzii_Xylariales
Bnummularia_Xylariales
Opiliferum_Ophiostomatales
Gclavigera_Ophiostomatales
Crosea_Hypocreales
Minundatum_Hypocreales
Bbassiana_Hypocreales
Cmilitaris_Hypocreales
taxa
20
40
60
80
100
0
1
2
3
4
5
6
7
8
branch length

OG0002838

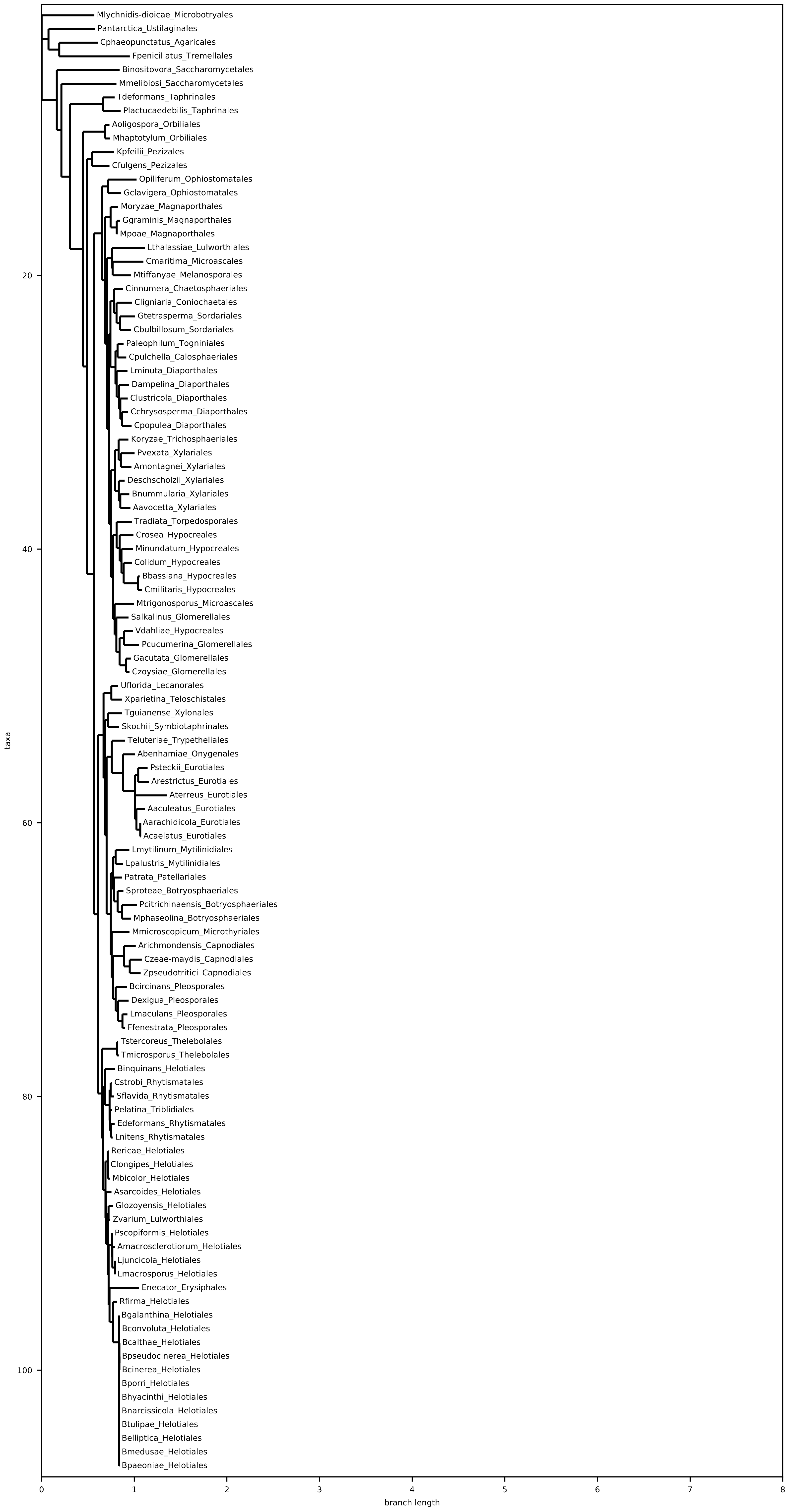
Mlychnidis-dioicae_Microbotryales
Pantarctica_Ustilaginales
Cphaeopunctatus_Agaricales
Fpenicillatus_Tremellales
Binositovora_Saccharomycetales
Mmelibiosi_Saccharomycetales
Tdeformans_Taphrinales
Plactucaedebilis_Taphrinales
Aoligospora_Orbiliales
Mhaptotylum_Orbiliales
Kpfeilii_Pezizales
Cfulgens_Pezizales
Opiliferum_Ophiostomatales
Gclavigera_Ophiostomatales
Moryzae_Magnaporthales
Ggraminis_Magnaporthales
Mpoae_Magnaporthales
Lthalassiae_Lulworthiales
Cmaritima_Microascales
Mtiffanyae_Melanosporales
Cinnumera_Chaetosphaeriales
Cligniaria_Coniochaetales
Gtetrasperma_Sordariales
Cbulbillosum_Sordariales
Paleophilum_Togniniales
Cpulchella_Calosphaeriales
Lminuta_Diaporthales
Dampelina_Diaporthales
Clustricola_Diaporthales
Cchrysosperma_Diaporthales
Cpopulea_Diaporthales
Koryzae_Trichosphaeriales
Pvexata_Xylariales
Amontagnei_Xylariales
Deschscholzii_Xylariales
Bnummularia_Xylariales
Aavocetta_Xylariales
Tradiata_Torpedosporales
Crosea_Hypocreales
Minundatum_Hypocreales
Colidum_Hypocreales
Bbassiana_Hypocreales
Cmilitaris_Hypocreales
Mtrigonosporus_Microascales
Salkalinus_Glomerellales
Vdahliae_Hypocreales
Pcucumerina_Glomerellales
Gacutata_Glomerellales
Czoysiae_Glomerellales
Uflorida_Lecanorales
Xparietina_Teloschistales
Tguianense_Xylonales
Skochii_Symbiotaphrinales
Teluteriae_Trypetheliales
Abenhamiae_Onygenales
Psteckii_Eurotiales
Arestrictus_Eurotiales
Aterreus_Eurotiales
Aaculeatus_Eurotiales
Aarachidicola_Eurotiales
Acaelatus_Eurotiales
Lmytilinum_Mytilinidiales
Lpalustris_Mytilinidiales
Patrata_Patellariales
Sproteae_Botryosphaeriales
Pcitrichinaensis_Botryosphaeriales
Mphaseolina_Botryosphaeriales
Mmicroscopicum_Microthyriales
Arichmondensis_Capnodiales
Czeae-maydis_Capnodiales
Zpseudotritici_Capnodiales
Bcircinans_Pleosporales
Dexigua_Pleosporales
Lmaculans_Pleosporales
Ffenestrata_Pleosporales
Tstercoreus_Thelebolales
Tmicrosporus_Thelebolales
Binquinans_Helotiales
Cstrobi_Rhytismatales
Sflavida_Rhytismatales
Pelatina_Triblidiales
Edeformans_Rhytismatales
Lnitens_Rhytismatales
Rericae_Helotiales
Clongipes_Helotiales
Mbicolor_Helotiales
Asarcoides_Helotiales
Glozoyensis_Helotiales
Zvarium_Lulworthiales
Pscopiformis_Helotiales
Amacrosclerotiorum_Helotiales
Ljuncicola_Helotiales
Lmacrosporus_Helotiales
Enecator_Erysiphales
Rfirma_Helotiales
Bgalanthina_Helotiales
Bconvoluta_Helotiales
Bcalthae_Helotiales
Bpseudocinerea_Helotiales
Bcinerea_Helotiales
Bporri_Helotiales
Bhyacinthi_Helotiales
Bnarcissicola_Helotiales
Btulipae_Helotiales
Belliptica_Helotiales
Bmedusae_Helotiales
Bpaeoniae_Helotiales
taxa
20
40
60
80
100
0
1
2
3
4
5
6
7
8
branch length

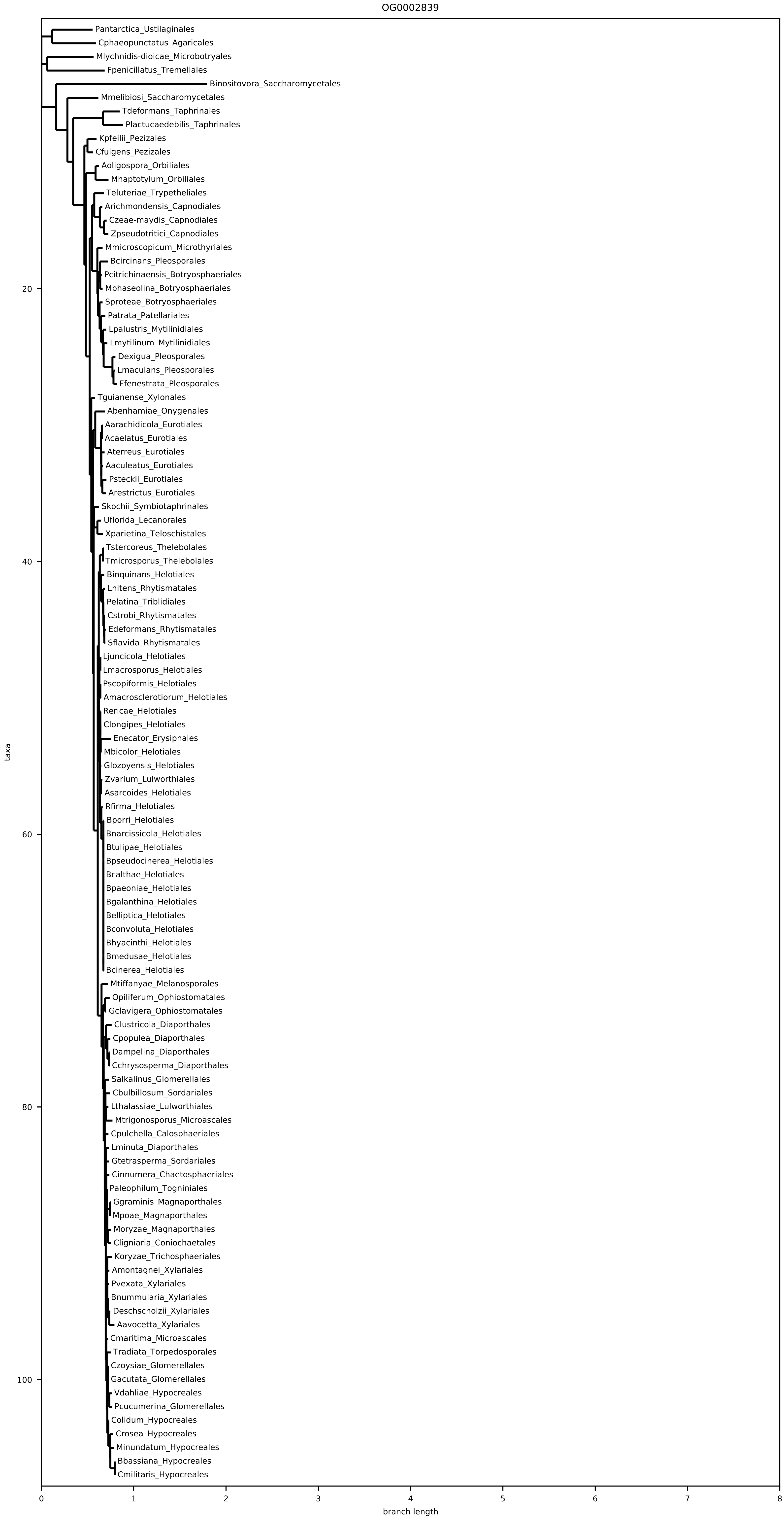
OG0002839
Pantarctica_Ustilaginales
Cphaeopunctatus_Agaricales
Mlychnidis-dioicae_Microbotryales
Fpenicillatus_Tremellales
Binositovora_Saccharomycetales
Mmelibiosi_Saccharomycetales
Tdeformans_Taphrinales
Plactucaedebilis_Taphrinales
Kpfeilii_Pezizales
Cfulgens_Pezizales
Aoligospora_Orbiliales
Mhaptotylum_Orbiliales
Teluteriae_Trypetheliales
Arichmondensis_Capnodiales
Czeae-maydis_Capnodiales
Zpseudotritici_Capnodiales
Mmicroscopicum_Microthyriales
Bcircinans_Pleosporales
Pcitrichinaensis_Botryosphaeriales
Mphaseolina_Botryosphaeriales
Sproteae_Botryosphaeriales
Patrata_Patellariales
Lpalustris_Mytilinidiales
Lmytilinum_Mytilinidiales
Dexigua_Pleosporales
Lmaculans_Pleosporales
Ffenestrata_Pleosporales
Tguianense_Xylonales
Abenhamiae_Onygenales
Aarachidicola_Eurotiales
Acaelatus_Eurotiales
Aterreus_Eurotiales
Aaculeatus_Eurotiales
Psteckii_Eurotiales
Arestrictus_Eurotiales
Skochii_Symbiotaphrinales
Uflorida_Lecanorales
Xparietina_Teloschistales
Tstercoreus_Thelebolales
Tmicrosporus_Thelebolales
Binquinans_Helotiales
Lnitens_Rhytismatales
Pelatina_Triblidiales
Cstrobi_Rhytismatales
Edeformans_Rhytismatales
Sflavida_Rhytismatales
Ljuncicola_Helotiales
Lmacrosporus_Helotiales
Pscopiformis_Helotiales
Amacrosclerotiorum_Helotiales
Rericae_Helotiales
Clongipes_Helotiales
Enecator_Erysiphales
Mbicolor_Helotiales
Glozoyensis_Helotiales
Zvarium_Lulworthiales
Asarcoides_Helotiales
Rfirma_Helotiales
Bporri_Helotiales
Bnarcissicola_Helotiales
Btulipae_Helotiales
Bpseudocinerea_Helotiales
Bcalthae_Helotiales
Bpaeoniae_Helotiales
Bgalanthina_Helotiales
Belliptica_Helotiales
Bconvoluta_Helotiales
Bhyacinthi_Helotiales
Bmedusae_Helotiales
Bcinerea_Helotiales
Mtiffanyae_Melanosporales
Opiliferum_Ophiostomatales
Gclavigera_Ophiostomatales
Clustricola_Diaporthales
Cpopulea_Diaporthales
Dampelina_Diaporthales
Cchrysosperma_Diaporthales
Salkalinus_Glomerellales
Cbulbillosum_Sordariales
Lthalassiae_Lulworthiales
Mtrigonosporus_Microascales
Cpulchella_Calosphaeriales
Lminuta_Diaporthales
Gtetrasperma_Sordariales
Cinnumera_Chaetosphaeriales
Paleophilum_Togniniales
Ggraminis_Magnaporthales
Mpoae_Magnaporthales
Moryzae_Magnaporthales
Cligniaria_Coniochaetales
Koryzae_Trichosphaeriales
Amontagnei_Xylariales
Pvexata_Xylariales
Bnummularia_Xylariales
Deschscholzii_Xylariales
Aavocetta_Xylariales
Cmaritima_Microascales
Tradiata_Torpedosporales
Czoysiae_Glomerellales
Gacutata_Glomerellales
Vdahliae_Hypocreales
Pcucumerina_Glomerellales
Colidum_Hypocreales
Crosea_Hypocreales
Minundatum_Hypocreales
Bbassiana_Hypocreales
Cmilitaris_Hypocreales
taxa
20
40
60
80
100
0
1
2
3
4
5
6
7
8
branch length

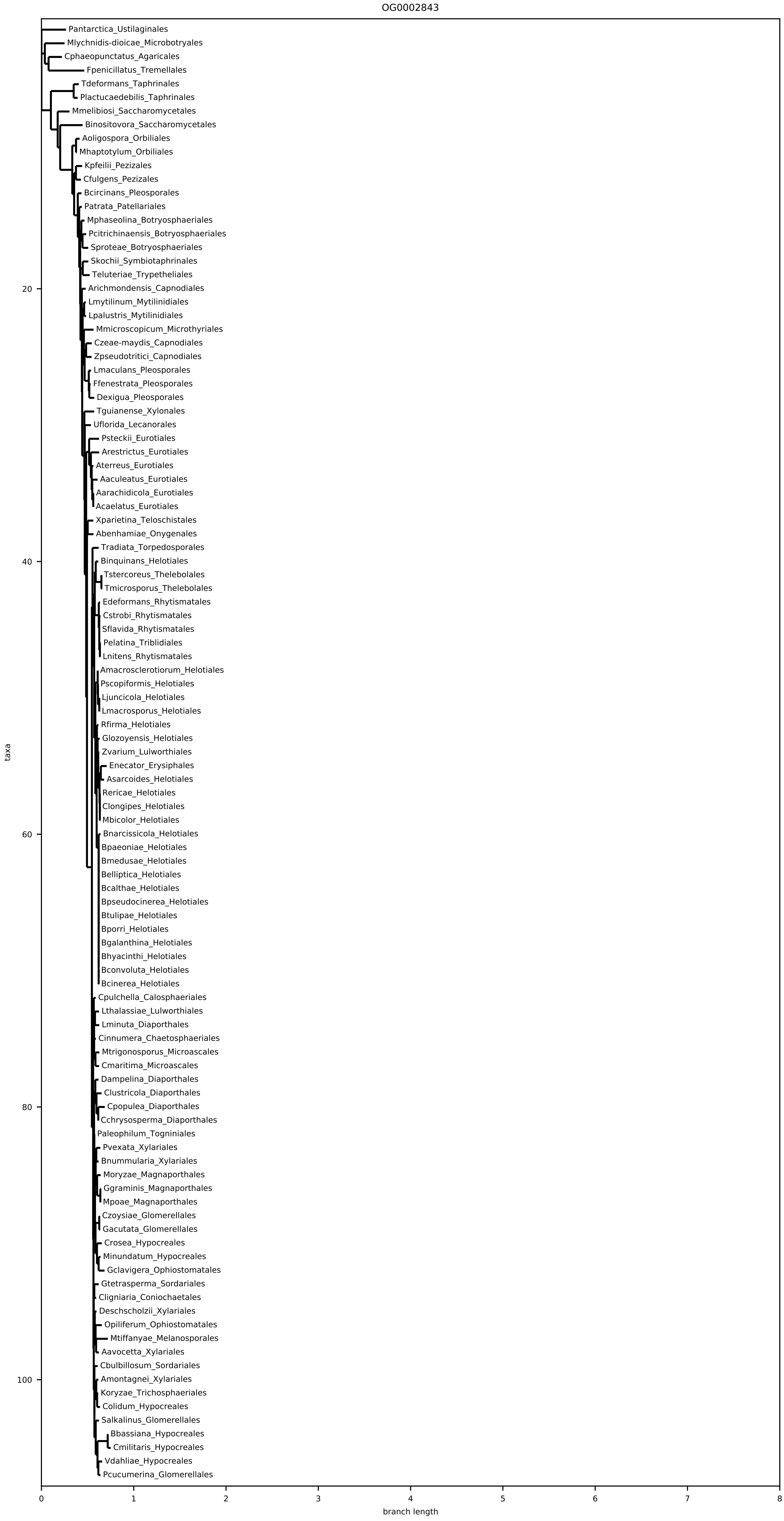

OG0002843
Pantarctica_Ustilaginales
Mlychnidis-dioicae_Microbotryales
Cphaeopunctatus_Agaricales
Fpenicillatus_Tremellales
Tdeformans_Taphrinales
Plactucaedebilis_Taphrinales
Mmelibiosi_Saccharomycetales
Binositovora_Saccharomycetales
Aoligospora_Orbiliales
Mhaptotylum_Orbiliales
Kpfeilii_Pezizales
Cfulgens_Pezizales
Bcircinans_Pleosporales
Patrata_Patellariales
Mphaseolina_Botryosphaeriales
Pcitrichinaensis_Botryosphaeriales
Sproteae_Botryosphaeriales
Skochii_Symbiotaphrinales
Teluteriae_Trypetheliales
Arichmondensis_Capnodiales
Lmytilinum_Mytilinidiales
Lpalustris_Mytilinidiales
Mmicroscopicum_Microthyriales
Czeae-maydis_Capnodiales
Zpseudotritici_Capnodiales
Lmaculans_Pleosporales
Ffenestrata_Pleosporales
Dexigua_Pleosporales
Tguianense_Xylonales
Uflorida_Lecanorales
Psteckii_Eurotiales
Arestrictus_Eurotiales
Aterreus_Eurotiales
Aaculeatus_Eurotiales
Aarachidicola_Eurotiales
Acaelatus_Eurotiales
Xparietina_Teloschistales
Abenhamiae_Onygenales
Tradiata_Torpedosporales
Binquinans_Helotiales
Tstercoreus_Thelebolales
Tmicrosporus_Thelebolales
Edeformans_Rhytismatales
Cstrobi_Rhytismatales
Sflavida_Rhytismatales
Pelatina_Triblidiales
Lnitens_Rhytismatales
Amacrosclerotiorum_Helotiales
Pscopiformis_Helotiales
Ljuncicola_Helotiales
Lmacrosporus_Helotiales
Rfirma_Helotiales
Glozoyensis_Helotiales
Zvarium_Lulworthiales
Enecator_Erysiphales
Asarcoides_Helotiales
Rericae_Helotiales
Clongipes_Helotiales
Mbicolor_Helotiales
Bnarcissicola_Helotiales
Bpaeoniae_Helotiales
Bmedusae_Helotiales
Belliptica_Helotiales
Bcalthae_Helotiales
Bpseudocinerea_Helotiales
Btulipae_Helotiales
Bporri_Helotiales
Bgalanthina_Helotiales
Bhyacinthi_Helotiales
Bconvoluta_Helotiales
Bcinerea_Helotiales
Cpulchella_Calosphaeriales
Lthalassiae_Lulworthiales
Lminuta_Diaporthales
Cinnumera_Chaetosphaeriales
Mtrigonosporus_Microascales
Cmaritima_Microascales
Dampelina_Diaporthales
Clustricola_Diaporthales
Cpopulea_Diaporthales
Cchrysosperma_Diaporthales
Paleophilum_Togniniales
Pvexata_Xylariales
Bnummularia_Xylariales
Moryzae_Magnaporthales
Ggraminis_Magnaporthales
Mpoae_Magnaporthales
Czoysiae_Glomerellales
Gacutata_Glomerellales
Crosea_Hypocreales
Minundatum_Hypocreales
Gclavigera_Ophiostomatales
Gtetrasperma_Sordariales
Cligniaria_Coniochaetales
Deschscholzii_Xylariales
Opiliferum_Ophiostomatales
Mtiffanyae_Melanosporales
Aavocetta_Xylariales
Cbulbillosum_Sordariales
Amontagnei_Xylariales
Koryzae_Trichosphaeriales
Colidum_Hypocreales
Salkalinus_Glomerellales
Bbassiana_Hypocreales
Cmilitaris_Hypocreales
Vdahliae_Hypocreales
Pcucumerina_Glomerellales
20
40
60
80
100
taxa
0
1
2
3
4
5
6
7
8
branch length

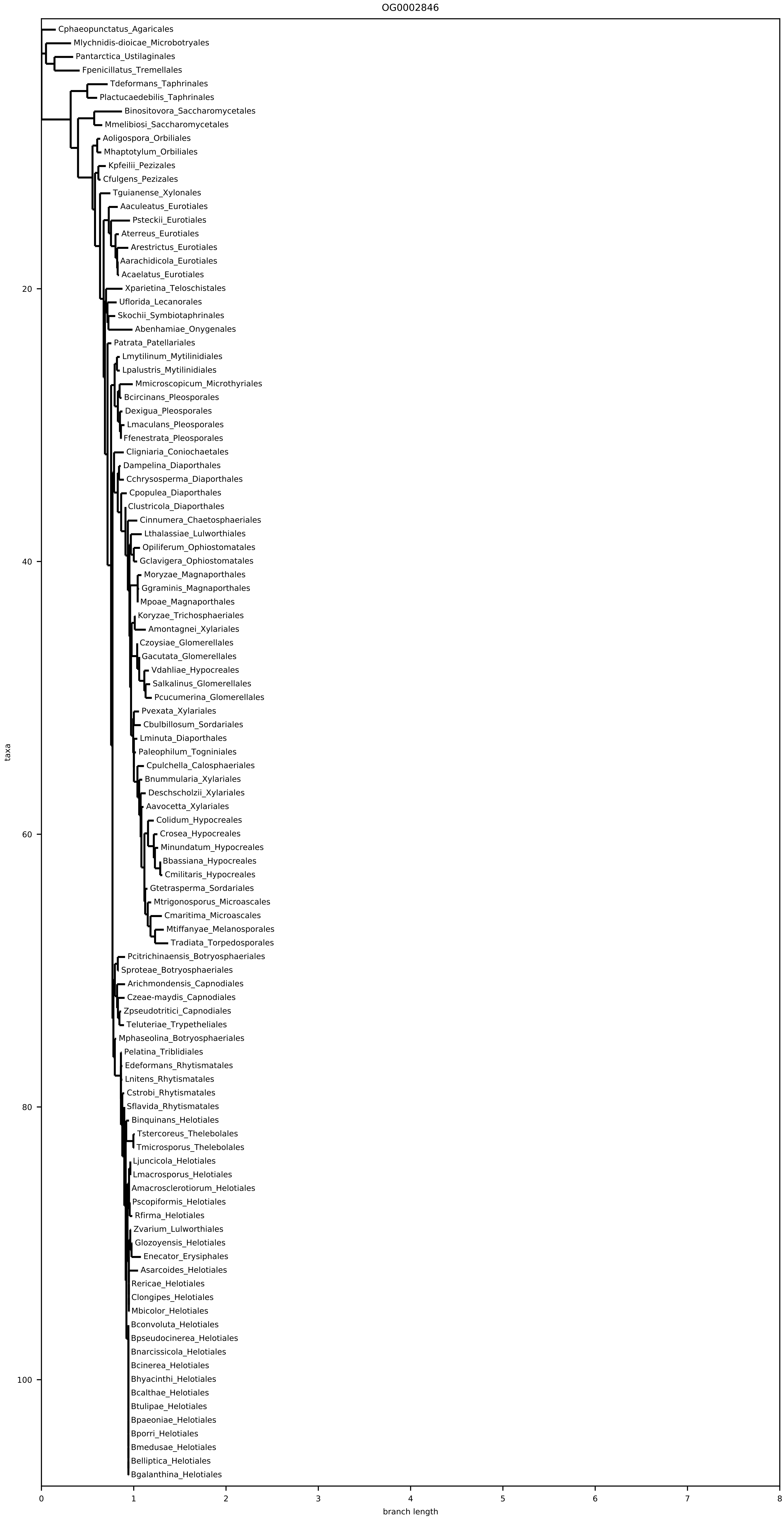
OG0002846
taxa
branch length
Cphaeopunctatus_Agaricales
Mlychnidis-dioicae_Microbotryales
Pantarctica_Ustilaginales
Fpenicillatus_Tremellales
Tdeformans_Taphrinales
Plactucaedebilis_Taphrinales
Binositovora_Saccharomycetales
Mmelibiosi_Saccharomycetales
Aoligospora_Orbiliales
Mhaptotylum_Orbiliales
Kpfeilii_Pezizales
Cfulgens_Pezizales
Tguianense_Xylonales
Aaculeatus_Eurotiales
Psteckii_Eurotiales
Aterreus_Eurotiales
Arestrictus_Eurotiales
Aarachidicola_Eurotiales
Acaelatus_Eurotiales
Xparietina_Teloschistales
Uflorida_Lecanorales
Skochii_Symbiotaphrinales
Abenhamiae_Onygenales
Patrata_Patellariales
Lmytilinum_Mytilinidiales
Lpalustris_Mytilinidiales
Mmicroscopicum_Microthyriales
Bcircinans_Pleosporales
Dexigua_Pleosporales
Lmaculans_Pleosporales
Ffenestrata_Pleosporales
Cligniaria_Coniochaetales
Dampelina_Diaporthales
Cchrysosperma_Diaporthales
Cpopulea_Diaporthales
Clustricola_Diaporthales
Cinnumera_Chaetosphaeriales
Lthalassiae_Lulworthiales
Opiliferum_Ophiostomatales
Gclavigera_Ophiostomatales
Moryzae_Magnaporthales
Ggraminis_Magnaporthales
Mpoae_Magnaporthales
Koryzae_Trichosphaeriales
Amontagnei_Xylariales
Czoysiae_Glomerellales
Gacutata_Glomerellales
Vdahliae_Hypocreales
Salkalinus_Glomerellales
Pcucumerina_Glomerellales
Pvexata_Xylariales
Cbulbillosum_Sordariales
Lminuta_Diaporthales
Paleophilum_Togniniales
Cpulchella_Calosphaeriales
Bnummularia_Xylariales
Deschscholzii_Xylariales
Aavocetta_Xylariales
Colidum_Hypocreales
Crosea_Hypocreales
Minundatum_Hypocreales
Bbassiana_Hypocreales
Cmilitaris_Hypocreales
Gtetrasperma_Sordariales
Mtrigonosporus_Microascales
Cmaritima_Microascales
Mtiffanyae_Melanosporales
Tradiata_Torpedosporales
Pcitrichinaensis_Botryosphaeriales
Sproteae_Botryosphaeriales
Arichmondensis_Capnodiales
Czeae-maydis_Capnodiales
Zpseudotritici_Capnodiales
Teluteriae_Trypetheliales
Mphaseolina_Botryosphaeriales
Pelatina_Triblidiales
Edeformans_Rhytismatales
Lnitens_Rhytismatales
Cstrobi_Rhytismatales
Sflavida_Rhytismatales
Binquinans_Helotiales
Tstercoreus_Thelebolales
Tmicrosporus_Thelebolales
Ljuncicola_Helotiales
Lmacrosporus_Helotiales
Amacrosclerotiorum_Helotiales
Pscopiformis_Helotiales
Rfirma_Helotiales
Zvarium_Lulworthiales
Glozoyensis_Helotiales
Enecator_Erysiphales
Asarcoides_Helotiales
Rericae_Helotiales
Clongipes_Helotiales
Mbicolor_Helotiales
Bconvoluta_Helotiales
Bpseudocinerea_Helotiales
Bnarcissicola_Helotiales
Bcinerea_Helotiales
Bhyacinthi_Helotiales
Bcalthae_Helotiales
Btulipae_Helotiales
Bpaeoniae_Helotiales
Bporri_Helotiales
Bmedusae_Helotiales
Belliptica_Helotiales
Bgalanthina_Helotiales
20
40
60
80
100
0
1
2
3
4
5
6
7
8

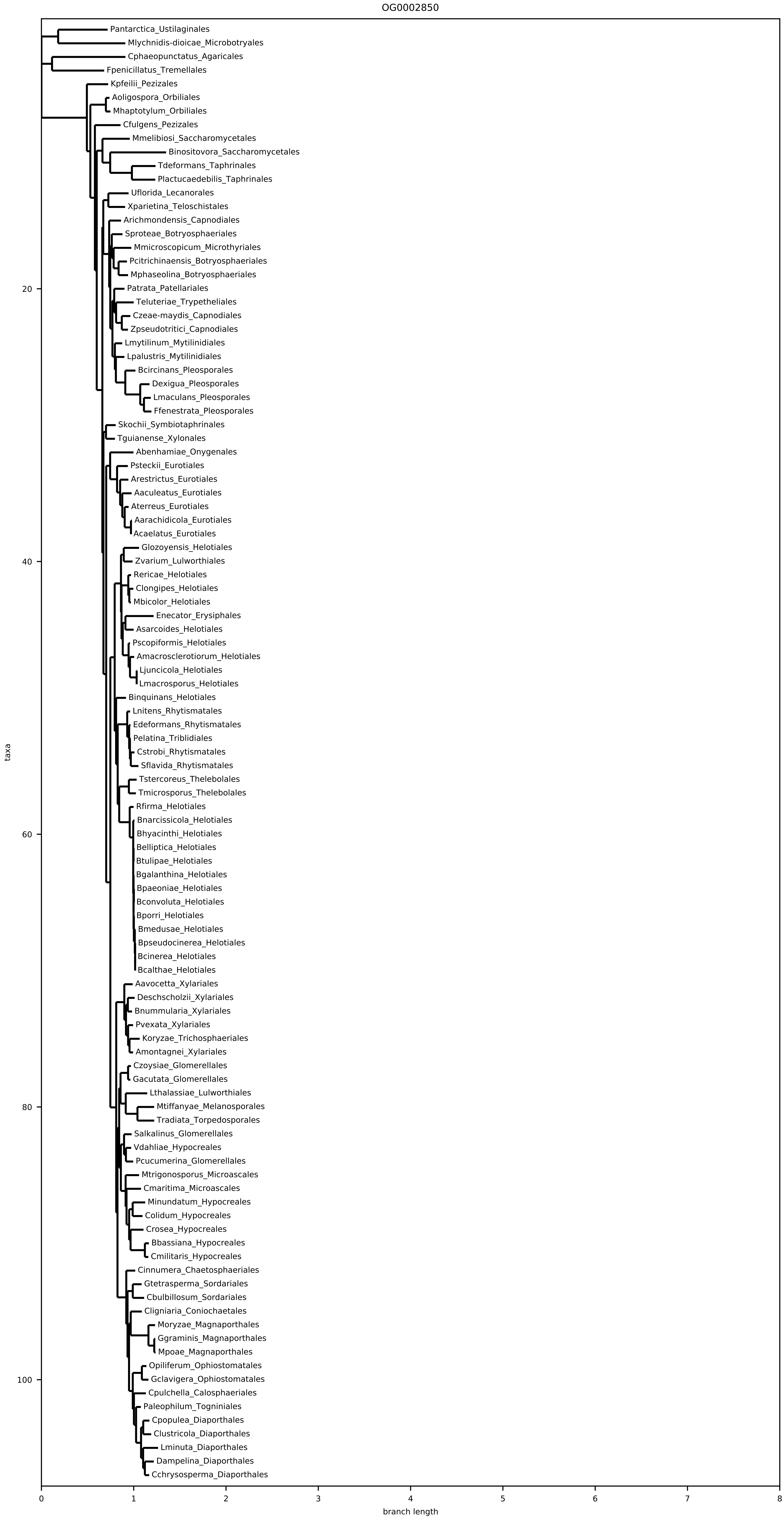

OG0002850
Pantarctica_Ustilaginales
Mlychnidis-dioicae_Microbotryales
Cphaeopunctatus_Agaricales
Fpenicillatus_Tremellales
Kpfeilii_Pezizales
Aoligospora_Orbiliales
Mhaptotylum_Orbiliales
Cfulgens_Pezizales
Mmelibiosi_Saccharomycetales
Binositovora_Saccharomycetales
Tdeformans_Taphrinales
Plactucaedebilis_Taphrinales
Uflorida_Lecanorales
Xparietina_Teloschistales
Arichmondensis_Capnodiales
Sproteae_Botryosphaeriales
Mmicroscopicum_Microthyriales
Pcitrichinaensis_Botryosphaeriales
Mphaseolina_Botryosphaeriales
Patrata_Patellariales
Teluteriae_Trypetheliales
Czeae-maydis_Capnodiales
Zpseudotritici_Capnodiales
Lmytilinum_Mytilinidiales
Lpalustris_Mytilinidiales
Bcircinans_Pleosporales
Dexigua_Pleosporales
Lmaculans_Pleosporales
Ffenestrata_Pleosporales
Skochii_Symbiotaphrinales
Tguianense_Xylonales
Abenhamiae_Onygenales
Psteckii_Eurotiales
Arestrictus_Eurotiales
Aaculeatus_Eurotiales
Aterreus_Eurotiales
Aarachidicola_Eurotiales
Acaelatus_Eurotiales
Glozoyensis_Helotiales
Zvarium_Lulworthiales
Rericae_Helotiales
Clongipes_Helotiales
Mbicolor_Helotiales
Enecator_Erysiphales
Asarcoides_Helotiales
Pscopiformis_Helotiales
Amacrosclerotiorum_Helotiales
Ljuncicola_Helotiales
Lmacrosporus_Helotiales
Binquinans_Helotiales
Lnitens_Rhytismatales
Edeformans_Rhytismatales
Pelatina_Triblidiales
Cstrobi_Rhytismatales
Sflavida_Rhytismatales
Tstercoreus_Thelebolales
Tmicrosporus_Thelebolales
Rfirma_Helotiales
Bnarcissicola_Helotiales
Bhyacinthi_Helotiales
Belliptica_Helotiales
Btulipae_Helotiales
Bgalanthina_Helotiales
Bpaeoniae_Helotiales
Bconvoluta_Helotiales
Bporri_Helotiales
Bmedusae_Helotiales
Bpseudocinerea_Helotiales
Bcinerea_Helotiales
Bcalthae_Helotiales
Aavocetta_Xylariales
Deschscholzii_Xylariales
Bnummularia_Xylariales
Pvexata_Xylariales
Koryzae_Trichosphaeriales
Amontagnei_Xylariales
Czoysiae_Glomerellales
Gacutata_Glomerellales
Lthalassiae_Lulworthiales
Mtiffanyae_Melanosporales
Tradiata_Torpedosporales
Salkalinus_Glomerellales
Vdahliae_Hypocreales
Pcucumerina_Glomerellales
Mtrigonosporus_Microascales
Cmaritima_Microascales
Minundatum_Hypocreales
Colidum_Hypocreales
Crosea_Hypocreales
Bbassiana_Hypocreales
Cmilitaris_Hypocreales
Cinnumera_Chaetosphaeriales
Gtetrasperma_Sordariales
Cbulbillosum_Sordariales
Cligniaria_Coniochaetales
Moryzae_Magnaporthales
Ggraminis_Magnaporthales
Mpoae_Magnaporthales
Opiliferum_Ophiostomatales
Gclavigera_Ophiostomatales
Cpulchella_Calosphaeriales
Paleophilum_Togniniales
Cpopulea_Diaporthales
Clustricola_Diaporthales
Lminuta_Diaporthales
Dampelina_Diaporthales
Cchrysosperma_Diaporthales
taxa
20
40
60
80
100
0
1
2
3
4
5
6
7
8
branch length

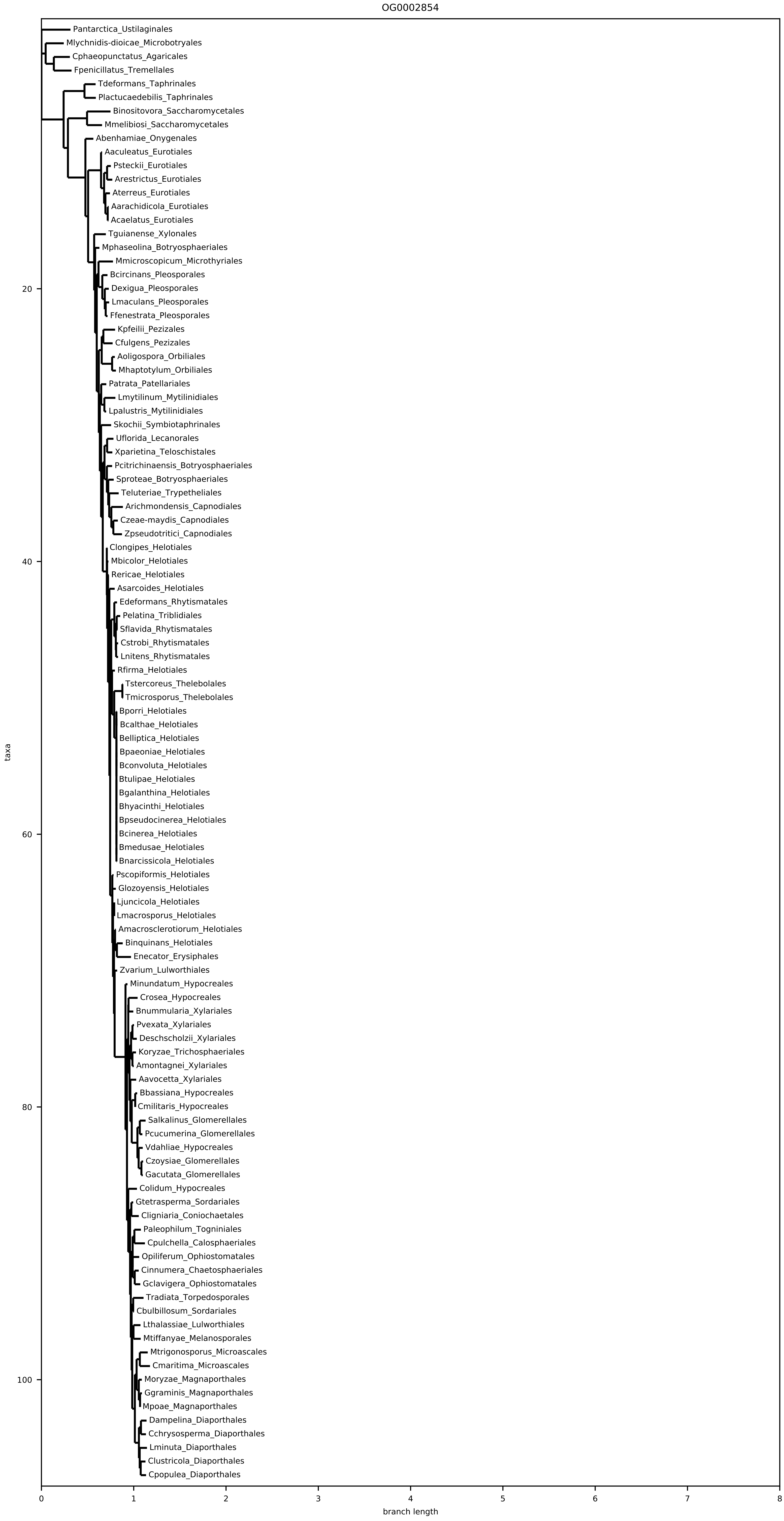

OG0002854
Pantarctica_Ustilaginales
Mlychnidis-dioicae_Microbotryales
Cphaeopunctatus_Agaricales
Fpenicillatus_Tremellales
Tdeformans_Taphrinales
Plactucaedebilis_Taphrinales
Binositovora_Saccharomycetales
Mmelibiosi_Saccharomycetales
Abenhamiae_Onygenales
Aaculeatus_Eurotiales
Psteckii_Eurotiales
Arestrictus_Eurotiales
Aterreus_Eurotiales
Aarachidicola_Eurotiales
Acaelatus_Eurotiales
Tguianense_Xylonales
Mphaseolina_Botryosphaeriales
Mmicroscopicum_Microthyriales
Bcircinans_Pleosporales
Dexigua_Pleosporales
Lmaculans_Pleosporales
Ffenestrata_Pleosporales
Kpfeilii_Pezizales
Cfulgens_Pezizales
Aoligospora_Orbiliales
Mhaptotylum_Orbiliales
Patrata_Patellariales
Lmytilinum_Mytilinidiales
Lpalustris_Mytilinidiales
Skochii_Symbiotaphrinales
Uflorida_Lecanorales
Xparietina_Teloschistales
Pcitrichinaensis_Botryosphaeriales
Sproteae_Botryosphaeriales
Teluteriae_Trypetheliales
Arichmondensis_Capnodiales
Czeae-maydis_Capnodiales
Zpseudotritici_Capnodiales
Clongipes_Helotiales
Mbicolor_Helotiales
Rericae_Helotiales
Asarcoides_Helotiales
Edeformans_Rhytismatales
Pelatina_Triblidiales
Sflavida_Rhytismatales
Cstrobi_Rhytismatales
Lnitens_Rhytismatales
Rfirma_Helotiales
Tstercoreus_Thelebolales
Tmicrosporus_Thelebolales
Bporri_Helotiales
Bcalthae_Helotiales
Belliptica_Helotiales
Bpaeoniae_Helotiales
Bconvoluta_Helotiales
Btulipae_Helotiales
Bgalanthina_Helotiales
Bhyacinthi_Helotiales
Bpseudocinerea_Helotiales
Bcinerea_Helotiales
Bmedusae_Helotiales
Bnarcissicola_Helotiales
Pscopiformis_Helotiales
Glozoyensis_Helotiales
Ljuncicola_Helotiales
Lmacrosporus_Helotiales
Amacrosclerotiorum_Helotiales
Binquinans_Helotiales
Enecator_Erysiphales
Zvarium_Lulworthiales
Minundatum_Hypocreales
Crosea_Hypocreales
Bnummularia_Xylariales
Pvexata_Xylariales
Deschscholzii_Xylariales
Koryzae_Trichosphaeriales
Amontagnei_Xylariales
Aavocetta_Xylariales
Bbassiana_Hypocreales
Cmilitaris_Hypocreales
Salkalinus_Glomerellales
Pcucumerina_Glomerellales
Vdahliae_Hypocreales
Czoysiae_Glomerellales
Gacutata_Glomerellales
Colidum_Hypocreales
Gtetrasperma_Sordariales
Cligniaria_Coniochaetales
Paleophilum_Togniniales
Cpulchella_Calosphaeriales
Opiliferum_Ophiostomatales
Cinnumera_Chaetosphaeriales
Gclavigera_Ophiostomatales
Tradiata_Torpedosporales
Cbulbillosum_Sordariales
Lthalassiae_Lulworthiales
Mtiffanyae_Melanosporales
Mtrigonosporus_Microascales
Cmaritima_Microascales
Moryzae_Magnaporthales
Ggraminis_Magnaporthales
Mpoae_Magnaporthales
Dampelina_Diaporthales
Cchrysosperma_Diaporthales
Lminuta_Diaporthales
Clustricola_Diaporthales
Cpopulea_Diaporthales
taxa
20
40
60
80
100
0
1
2
3
4
5
6
7
8
branch length

OG0002856

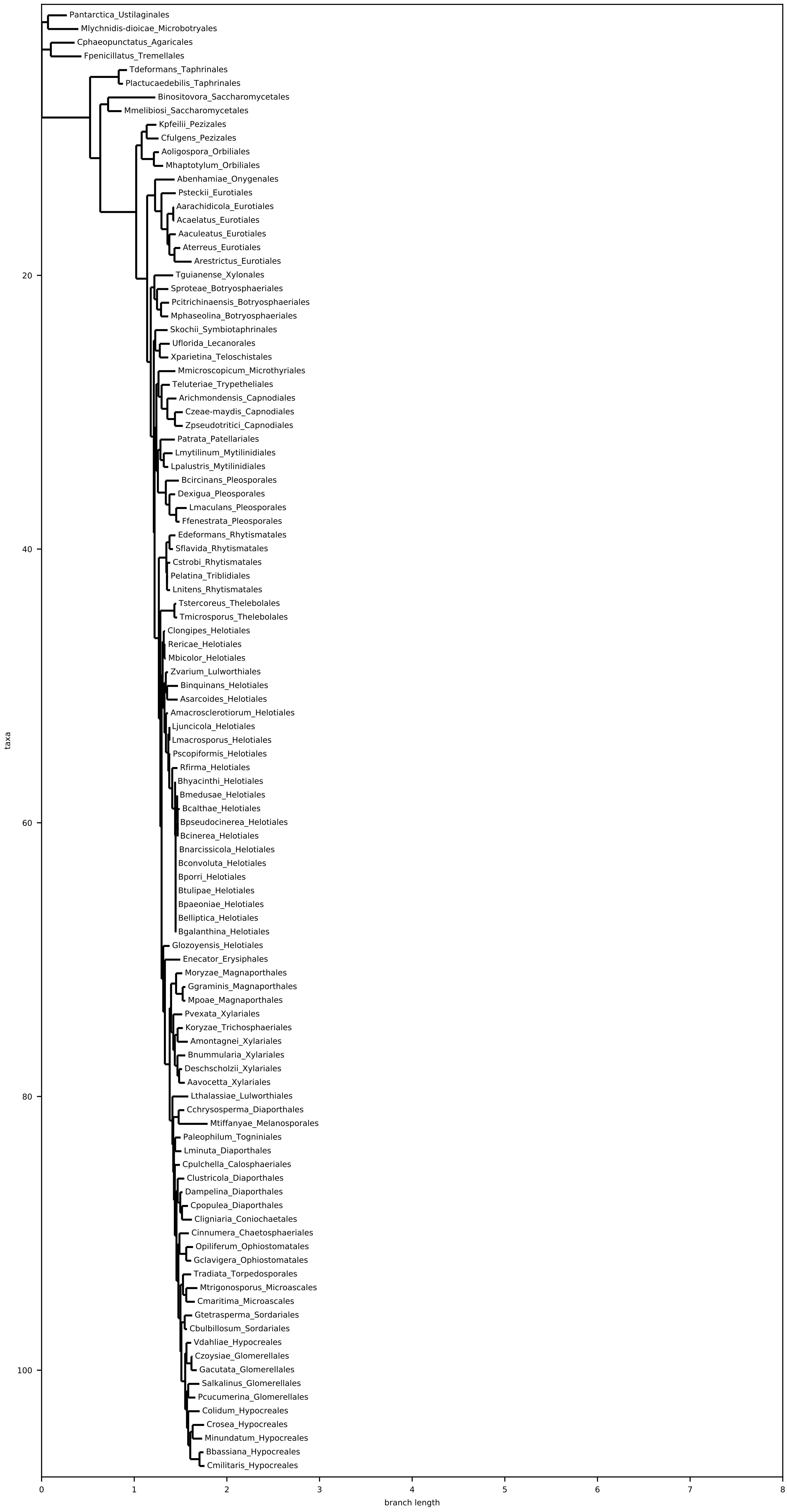

Pantarctica_Ustilaginales
Mlychnidis-dioicae_Microbotryales
Cphaeopunctatus_Agaricales
Fpenicillatus_Tremellales
Tdeformans_Taphrinales
Plactucaedebilis_Taphrinales
Binositovora_Saccharomycetales
Mmelibiosi_Saccharomycetales
Kpfeilii_Pezizales
Cfulgens_Pezizales
Aoligospora_Orbiliales
Mhaptotylum_Orbiliales
Abenhamiae_Onygenales
Psteckii_Eurotiales
Aarachidicola_Eurotiales
Acaelatus_Eurotiales
Aaculeatus_Eurotiales
Aterreus_Eurotiales
Arestrictus_Eurotiales
Tguianense_Xylonales
Sproteae_Botryosphaeriales
Pcitrichinaensis_Botryosphaeriales
Mphaseolina_Botryosphaeriales
Skochii_Symbiotaphrinales
Uflorida_Lecanorales
Xparietina_Teloschistales
Mmicroscopicum_Microthyriales
Teluteriae_Trypetheliales
Arichmondensis_Capnodiales
Czeae-maydis_Capnodiales
Zpseudotritici_Capnodiales
Patrata_Patellariales
Lmytilinum_Mytilinidiales
Lpalustris_Mytilinidiales
Bcircinans_Pleosporales
Dexigua_Pleosporales
Lmaculans_Pleosporales
Ffenestrata_Pleosporales
Edeformans_Rhytismatales
Sflavida_Rhytismatales
Cstrobi_Rhytismatales
Pelatina_Triblidiales
Lnitens_Rhytismatales
Tstercoreus_Thelebolales
Tmicrosporus_Thelebolales
Clongipes_Helotiales
Rericae_Helotiales
Mbicolor_Helotiales
Zvarium_Lulworthiales
Binquinans_Helotiales
Asarcoides_Helotiales
Amacrosclerotiorum_Helotiales
Ljuncicola_Helotiales
Lmacrosporus_Helotiales
Pscopiformis_Helotiales
Rfirma_Helotiales
Bhyacinthi_Helotiales
Bmedusae_Helotiales
Bcalthae_Helotiales
Bpseudocinerea_Helotiales
Bcinerea_Helotiales
Bnarcissicola_Helotiales
Bconvoluta_Helotiales
Bporri_Helotiales
Btulipae_Helotiales
Bpaeoniae_Helotiales
Belliptica_Helotiales
Bgalanthina_Helotiales
Glozoyensis_Helotiales
Enecator_Erysiphales
Moryzae_Magnaporthales
Ggraminis_Magnaporthales
Mpoae_Magnaporthales
Pvexata_Xylariales
Koryzae_Trichosphaeriales
Amontagnei_Xylariales
Bnummularia_Xylariales
Deschscholzii_Xylariales
Aavocetta_Xylariales
Lthalassiae_Lulworthiales
Cchrysosperma_Diaporthales
Mtiffanyae_Melanosporales
Paleophilum_Togniniales
Lminuta_Diaporthales
Cpulchella_Calosphaeriales
Clustricola_Diaporthales
Dampelina_Diaporthales
Cpopulea_Diaporthales
Cligniaria_Coniochaetales
Cinnumera_Chaetosphaeriales
Opiliferum_Ophiostomatales
Gclavigera_Ophiostomatales
Tradiata_Torpedosporales
Mtrigonosporus_Microascales
Cmaritima_Microascales
Gtetrasperma_Sordariales
Cbulbillosum_Sordariales
Vdahliae_Hypocreales
Czoysiae_Glomerellales
Gacutata_Glomerellales
Salkalinus_Glomerellales
Pcucumerina_Glomerellales
Colidum_Hypocreales
Crosea_Hypocreales
Minundatum_Hypocreales
Bbassiana_Hypocreales
Cmilitaris_Hypocreales
taxa
20
40
60
80
100
0
1
2
3
4
5
6
7
8
branch length

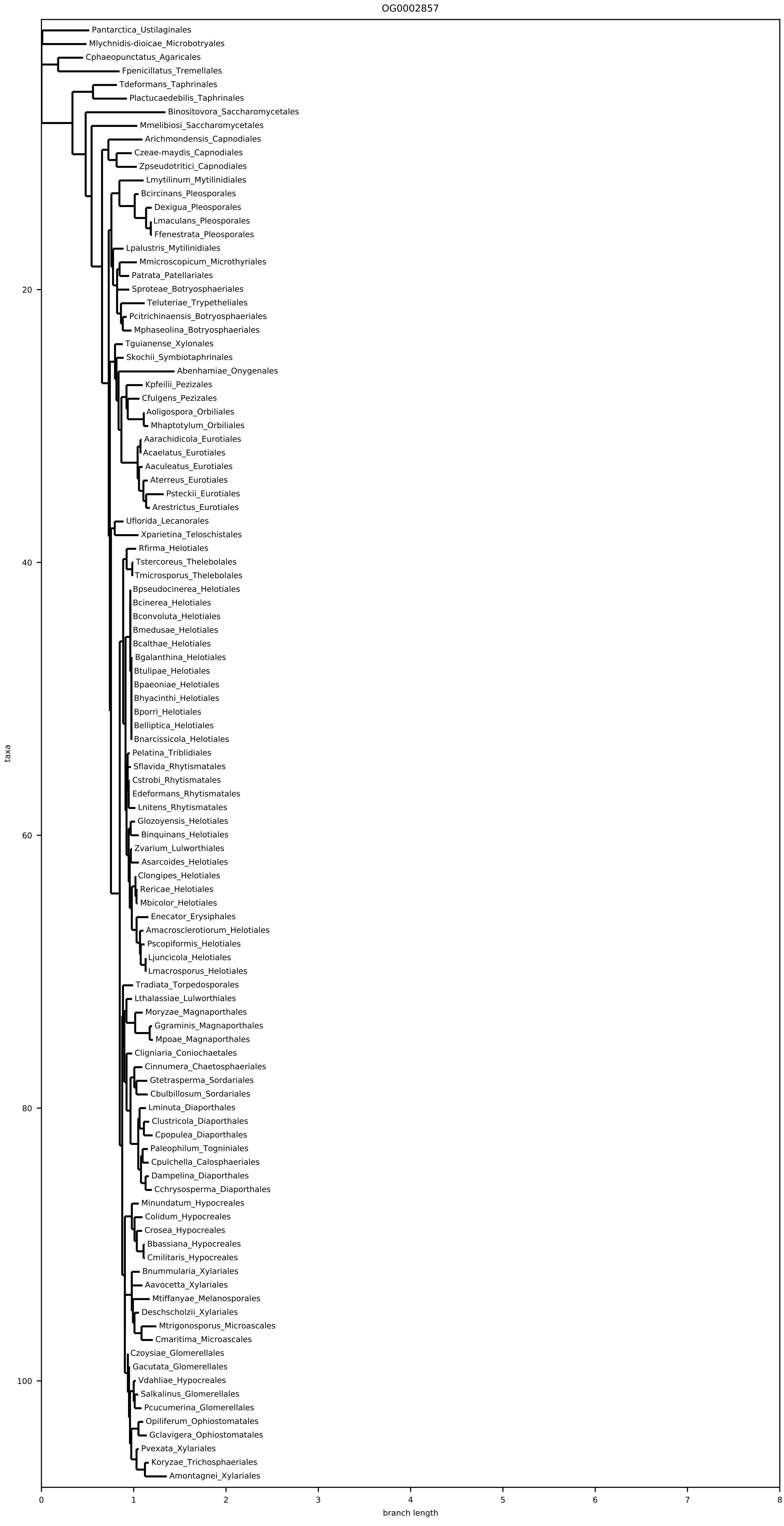
OG0002857
Pantarctica_Ustilaginales
Mlychnidis-dioicae_Microbotryales
Cphaeopunctatus_Agaricales
Fpenicillatus_Tremellales
Tdeformans_Taphrinales
Plactucaedebilis_Taphrinales
Binositovora_Saccharomycetales
Mmelibiosi_Saccharomycetales
Arichmondensis_Capnodiales
Czeae-maydis_Capnodiales
Zpseudotritici_Capnodiales
Lmytilinum_Mytilinidiales
Bcircinans_Pleosporales
Dexigua_Pleosporales
Lmaculans_Pleosporales
Ffenestrata_Pleosporales
Lpalustris_Mytilinidiales
Mmicroscopicum_Microthyriales
Patrata_Patellariales
Sproteae_Botryosphaeriales
Teluteriae_Trypetheliales
Pcitrichinaensis_Botryosphaeriales
Mphaseolina_Botryosphaeriales
Tguianense_Xylonales
Skochii_Symbiotaphrinales
Abenhamiae_Onygenales
Kpfeilii_Pezizales
Cfulgens_Pezizales
Aoligospora_Orbiliales
Mhaptotylum_Orbiliales
Aarachidicola_Eurotiales
Acaelatus_Eurotiales
Aaculeatus_Eurotiales
Aterreus_Eurotiales
Psteckii_Eurotiales
Arestrictus_Eurotiales
Uflorida_Lecanorales
Xparietina_Teloschistales
Rfirma_Helotiales
Tstercoreus_Thelebolales
Tmicrosporus_Thelebolales
Bpseudocinerea_Helotiales
Bcinerea_Helotiales
Bconvoluta_Helotiales
Bmedusae_Helotiales
Bcalthae_Helotiales
Bgalanthina_Helotiales
Btulipae_Helotiales
Bpaeoniae_Helotiales
Bhyacinthi_Helotiales
Bporri_Helotiales
Belliptica_Helotiales
Bnarcissicola_Helotiales
Pelatina_Triblidiales
Sflavida_Rhytismatales
Cstrobi_Rhytismatales
Edeformans_Rhytismatales
Lnitens_Rhytismatales
Glozoyensis_Helotiales
Binquinans_Helotiales
Zvarium_Lulworthiales
Asarcoides_Helotiales
Clongipes_Helotiales
Rericae_Helotiales
Mbicolor_Helotiales
Enecator_Erysiphales
Amacrosclerotiorum_Helotiales
Pscopiformis_Helotiales
Ljuncicola_Helotiales
Lmacrosporus_Helotiales
Tradiata_Torpedosporales
Lthalassiae_Lulworthiales
Moryzae_Magnaporthales
Ggraminis_Magnaporthales
Mpoae_Magnaporthales
Cligniaria_Coniochaetales
Cinnumera_Chaetosphaeriales
Gtetrasperma_Sordariales
Cbulbillosum_Sordariales
Lminuta_Diaporthales
Clustricola_Diaporthales
Cpopulea_Diaporthales
Paleophilum_Togniniales
Cpulchella_Calosphaeriales
Dampelina_Diaporthales
Cchrysosperma_Diaporthales
Minundatum_Hypocreales
Colidum_Hypocreales
Crosea_Hypocreales
Bbassiana_Hypocreales
Cmilitaris_Hypocreales
Bnummularia_Xylariales
Aavocetta_Xylariales
Mtiffanyae_Melanosporales
Deschscholzii_Xylariales
Mtrigonosporus_Microascales
Cmaritima_Microascales
Czoysiae_Glomerellales
Gacutata_Glomerellales
Vdahliae_Hypocreales
Salkalinus_Glomerellales
Pcucumerina_Glomerellales
Opiliferum_Ophiostomatales
Gclavigera_Ophiostomatales
Pvexata_Xylariales
Koryzae_Trichosphaeriales
Amontagnei_Xylariales
20
40
60
80
100
taxa
0
1
2
3
4
5
6
7
8
branch length

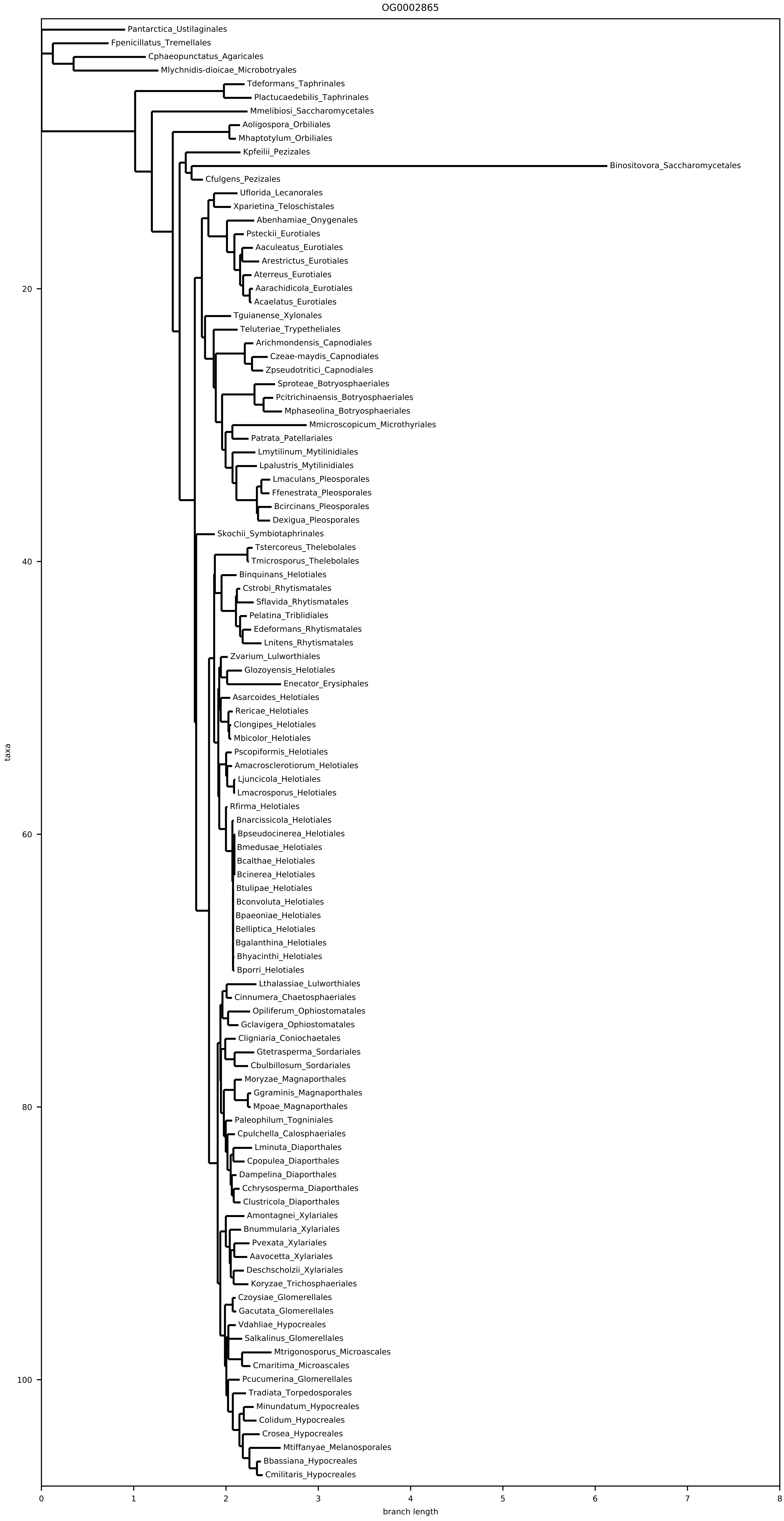

OG0002865
Pantarctica_Ustilaginales
Fpenicillatus_Tremellales
Cphaeopunctatus_Agaricales
Mlychnidis-dioicae_Microbotryales
Tdeformans_Taphrinales
Plactucaedebilis_Taphrinales
Mmelibiosi_Saccharomycetales
Aoligospora_Orbiliales
Mhaptotylum_Orbiliales
Kpfeilii_Pezizales
Binositovora_Saccharomycetales
Cfulgens_Pezizales
Uflorida_Lecanorales
Xparietina_Teloschistales
Abenhamiae_Onygenales
Psteckii_Eurotiales
Aaculeatus_Eurotiales
Arestrictus_Eurotiales
Aterreus_Eurotiales
Aarachidicola_Eurotiales
Acaelatus_Eurotiales
Tguianense_Xylonales
Teluteriae_Trypetheliales
Arichmondensis_Capnodiales
Czeae-maydis_Capnodiales
Zpseudotritici_Capnodiales
Sproteae_Botryosphaeriales
Pcitrichinaensis_Botryosphaeriales
Mphaseolina_Botryosphaeriales
Mmicroscopicum_Microthyriales
Patrata_Patellariales
Lmytilinum_Mytilinidiales
Lpalustris_Mytilinidiales
Lmaculans_Pleosporales
Ffenestrata_Pleosporales
Bcircinans_Pleosporales
Dexigua_Pleosporales
Skochii_Symbiotaphrinales
Tstercoreus_Thelebolales
Tmicrosporus_Thelebolales
Binquinans_Helotiales
Cstrobi_Rhytismatales
Sflavida_Rhytismatales
Pelatina_Triblidiales
Edeformans_Rhytismatales
Lnitens_Rhytismatales
Zvarium_Lulworthiales
Glozoyensis_Helotiales
Enecator_Erysiphales
Asarcoides_Helotiales
Rericae_Helotiales
Clongipes_Helotiales
Mbicolor_Helotiales
Pscopiformis_Helotiales
Amacrosclerotiorum_Helotiales
Ljuncicola_Helotiales
Lmacrosporus_Helotiales
Rfirma_Helotiales
Bnarcissicola_Helotiales
Bpseudocinerea_Helotiales
Bmedusae_Helotiales
Bcalthae_Helotiales
Bcinerea_Helotiales
Btulipae_Helotiales
Bconvoluta_Helotiales
Bpaeoniae_Helotiales
Belliptica_Helotiales
Bgalanthina_Helotiales
Bhyacinthi_Helotiales
Bporri_Helotiales
Lthalassiae_Lulworthiales
Cinnumera_Chaetosphaeriales
Opiliferum_Ophiostomatales
Gclavigera_Ophiostomatales
Cligniaria_Coniochaetales
Gtetrasperma_Sordariales
Cbulbillosum_Sordariales
Moryzae_Magnaporthales
Ggraminis_Magnaporthales
Mpoae_Magnaporthales
Paleophilum_Togniniales
Cpulchella_Calosphaeriales
Lminuta_Diaporthales
Cpopulea_Diaporthales
Dampelina_Diaporthales
Cchrysosperma_Diaporthales
Clustricola_Diaporthales
Amontagnei_Xylariales
Bnummularia_Xylariales
Pvexata_Xylariales
Aavocetta_Xylariales
Deschscholzii_Xylariales
Koryzae_Trichosphaeriales
Czoysiae_Glomerellales
Gacutata_Glomerellales
Vdahliae_Hypocreales
Salkalinus_Glomerellales
Mtrigonosporus_Microascales
Cmaritima_Microascales
Pcucumerina_Glomerellales
Tradiata_Torpedosporales
Minundatum_Hypocreales
Colidum_Hypocreales
Crosea_Hypocreales
Mtiffanyae_Melanosporales
Bbassiana_Hypocreales
Cmilitaris_Hypocreales
taxa
20
40
60
80
100
0
1
2
3
4
5
6
7
8
branch length

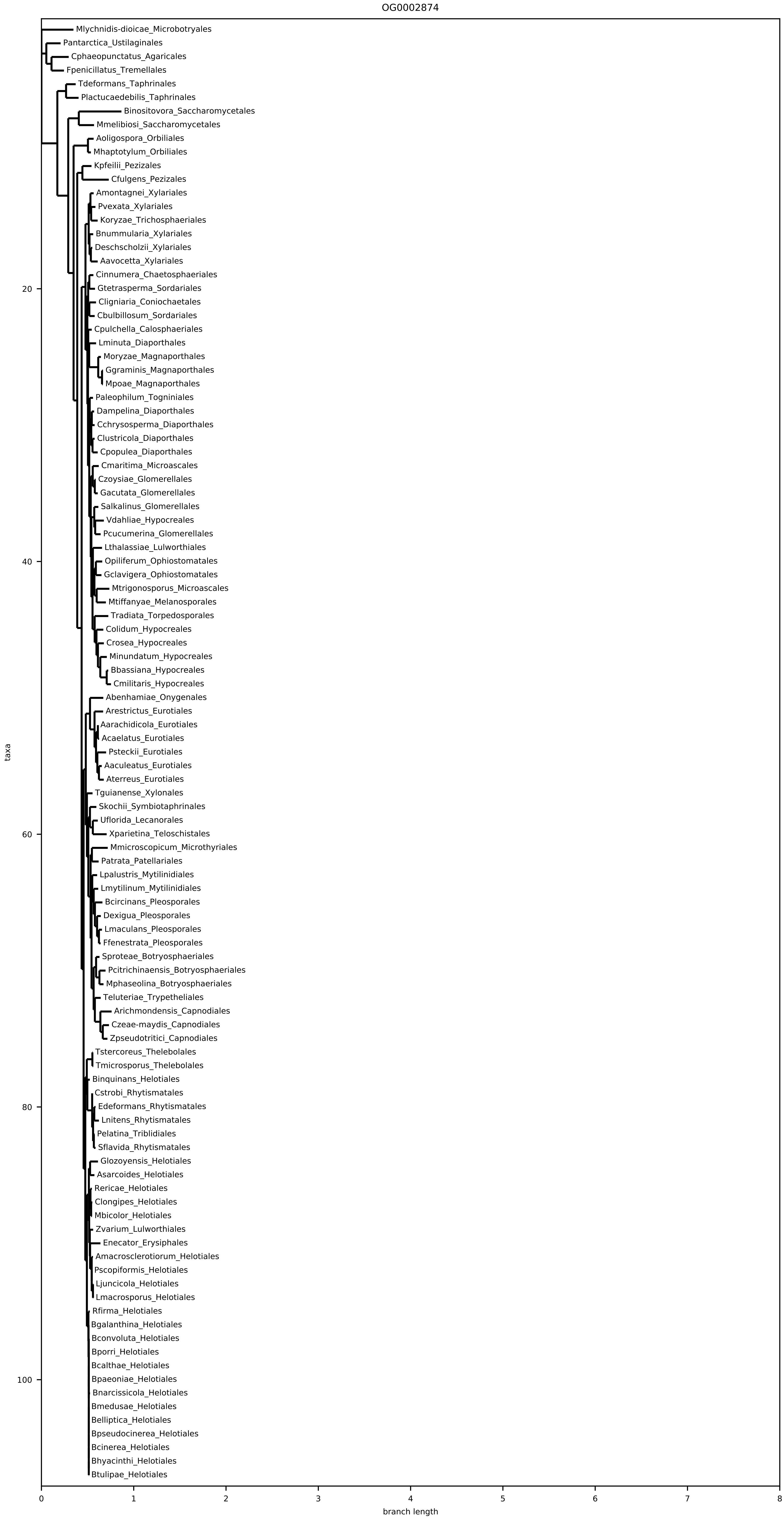

OG0002874
Mlychnidis-dioicae_Microbotryales
Pantarctica_Ustilaginales
Cphaeopunctatus_Agaricales
Fpenicillatus_Tremellales
Tdeformans_Taphrinales
Plactucaedebilis_Taphrinales
Binositovora_Saccharomycetales
Mmelibiosi_Saccharomycetales
Aoligospora_Orbiliales
Mhaptotylum_Orbiliales
Kpfeilii_Pezizales
Cfulgens_Pezizales
Amontagnei_Xylariales
Pvexata_Xylariales
Koryzae_Trichosphaeriales
Bnummularia_Xylariales
Deschscholzii_Xylariales
Aavocetta_Xylariales
Cinnumera_Chaetosphaeriales
Gtetrasperma_Sordariales
Cligniaria_Coniochaetales
Cbulbillosum_Sordariales
Cpulchella_Calosphaeriales
Lminuta_Diaporthales
Moryzae_Magnaporthales
Ggraminis_Magnaporthales
Mpoae_Magnaporthales
Paleophilum_Togniniales
Dampelina_Diaporthales
Cchrysosperma_Diaporthales
Clustricola_Diaporthales
Cpopulea_Diaporthales
Cmaritima_Microascales
Czoysiae_Glomerellales
Gacutata_Glomerellales
Salkalinus_Glomerellales
Vdahliae_Hypocreales
Pcucumerina_Glomerellales
Lthalassiae_Lulworthiales
Opiliferum_Ophiostomatales
Gclavigera_Ophiostomatales
Mtrigonosporus_Microascales
Mtiffanyae_Melanosporales
Tradiata_Torpedosporales
Colidum_Hypocreales
Crosea_Hypocreales
Minundatum_Hypocreales
Bbassiana_Hypocreales
Cmilitaris_Hypocreales
Abenhamiae_Onygenales
Arestrictus_Eurotiales
Aarachidicola_Eurotiales
Acaelatus_Eurotiales
Psteckii_Eurotiales
Aaculeatus_Eurotiales
Aterreus_Eurotiales
Tguianense_Xylonales
Skochii_Symbiotaphrinales
Uflorida_Lecanorales
Xparietina_Teloschistales
Mmicroscopicum_Microthyriales
Patrata_Patellariales
Lpalustris_Mytilinidiales
Lmytilinum_Mytilinidiales
Bcircinans_Pleosporales
Dexigua_Pleosporales
Lmaculans_Pleosporales
Ffenestrata_Pleosporales
Sproteae_Botryosphaeriales
Pcitrichinaensis_Botryosphaeriales
Mphaseolina_Botryosphaeriales
Teluteriae_Trypetheliales
Arichmondensis_Capnodiales
Czeae-maydis_Capnodiales
Zpseudotritici_Capnodiales
Tstercoreus_Thelebolales
Tmicrosporus_Thelebolales
Binquinans_Helotiales
Cstrobi_Rhytismatales
Edeformans_Rhytismatales
Lnitens_Rhytismatales
Pelatina_Triblidiales
Sflavida_Rhytismatales
Glozoyensis_Helotiales
Asarcoides_Helotiales
Rericae_Helotiales
Clongipes_Helotiales
Mbicolor_Helotiales
Zvarium_Lulworthiales
Enecator_Erysiphales
Amacrosclerotiorum_Helotiales
Pscopiformis_Helotiales
Ljuncicola_Helotiales
Lmacrosporus_Helotiales
Rfirma_Helotiales
Bgalanthina_Helotiales
Bconvoluta_Helotiales
Bporri_Helotiales
Bcalthae_Helotiales
Bpaeoniae_Helotiales
Bnarcissicola_Helotiales
Bmedusae_Helotiales
Belliptica_Helotiales
Bpseudocinerea_Helotiales
Bcinerea_Helotiales
Bhyacinthi_Helotiales
Btulipae_Helotiales
taxa
20
40
60
80
100
0
1
2
3
4
5
6
7
8
branch length

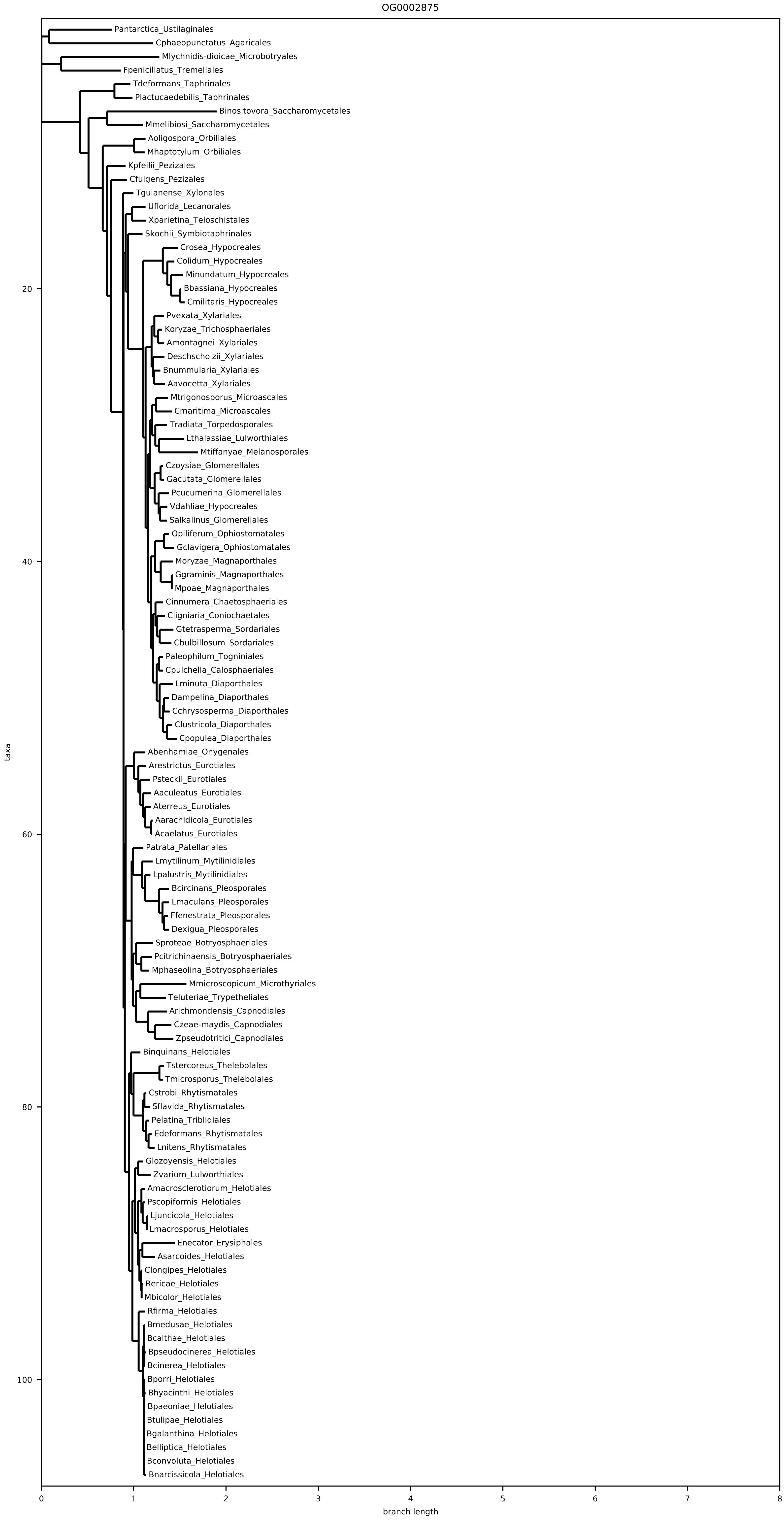
OG0002875
Pantarctica_Ustilaginales
Cphaeopunctatus_Agaricales
Mlychnidis-dioicae_Microbotryales
Fpenicillatus_Tremellales
Tdeformans_Taphrinales
Plactucaedebilis_Taphrinales
Binositovora_Saccharomycetales
Mmelibiosi_Saccharomycetales
Aoligospora_Orbiliales
Mhaptotylum_Orbiliales
Kpfeilii_Pezizales
Cfulgens_Pezizales
Tguianense_Xylonales
Uflorida_Lecanorales
Xparietina_Teloschistales
Skochii_Symbiotaphrinales
Crosea_Hypocreales
Colidum_Hypocreales
Minundatum_Hypocreales
Bbassiana_Hypocreales
Cmilitaris_Hypocreales
Pvexata_Xylariales
Koryzae_Trichosphaeriales
Amontagnei_Xylariales
Deschscholzii_Xylariales
Bnummularia_Xylariales
Aavocetta_Xylariales
Mtrigonosporus_Microascales
Cmaritima_Microascales
Tradiata_Torpedosporales
Lthalassiae_Lulworthiales
Mtiffanyae_Melanosporales
Czoysiae_Glomerellales
Gacutata_Glomerellales
Pcucumerina_Glomerellales
Vdahliae_Hypocreales
Salkalinus_Glomerellales
Opiliferum_Ophiostomatales
Gclavigera_Ophiostomatales
Moryzae_Magnaporthales
Ggraminis_Magnaporthales
Mpoae_Magnaporthales
Cinnumera_Chaetosphaeriales
Cligniaria_Coniochaetales
Gtetrasperma_Sordariales
Cbulbillosum_Sordariales
Paleophilum_Togniniales
Cpulchella_Calosphaeriales
Lminuta_Diaporthales
Dampelina_Diaporthales
Cchrysosperma_Diaporthales
Clustricola_Diaporthales
Cpopulea_Diaporthales
Abenhamiae_Onygenales
Arestrictus_Eurotiales
Psteckii_Eurotiales
Aaculeatus_Eurotiales
Aterreus_Eurotiales
Aarachidicola_Eurotiales
Acaelatus_Eurotiales
Patrata_Patellariales
Lmytilinum_Mytilinidiales
Lpalustris_Mytilinidiales
Bcircinans_Pleosporales
Lmaculans_Pleosporales
Ffenestrata_Pleosporales
Dexigua_Pleosporales
Sproteae_Botryosphaeriales
Pcitrichinaensis_Botryosphaeriales
Mphaseolina_Botryosphaeriales
Mmicroscopicum_Microthyriales
Teluteriae_Trypetheliales
Arichmondensis_Capnodiales
Czeae-maydis_Capnodiales
Zpseudotritici_Capnodiales
Binquinans_Helotiales
Tstercoreus_Thelebolales
Tmicrosporus_Thelebolales
Cstrobi_Rhytismatales
Sflavida_Rhytismatales
Pelatina_Triblidiales
Edeformans_Rhytismatales
Lnitens_Rhytismatales
Glozoyensis_Helotiales
Zvarium_Lulworthiales
Amacrosclerotiorum_Helotiales
Pscopiformis_Helotiales
Ljuncicola_Helotiales
Lmacrosporus_Helotiales
Enecator_Erysiphales
Asarcoides_Helotiales
Clongipes_Helotiales
Rericae_Helotiales
Mbicolor_Helotiales
Rfirma_Helotiales
Bmedusae_Helotiales
Bcalthae_Helotiales
Bpseudocinerea_Helotiales
Bcinerea_Helotiales
Bporri_Helotiales
Bhyacinthi_Helotiales
Bpaeoniae_Helotiales
Btulipae_Helotiales
Bgalanthina_Helotiales
Belliptica_Helotiales
Bconvoluta_Helotiales
Bnarcissicola_Helotiales
taxa
20
40
60
80
100
0
1
2
3
4
5
6
7
8
branch length

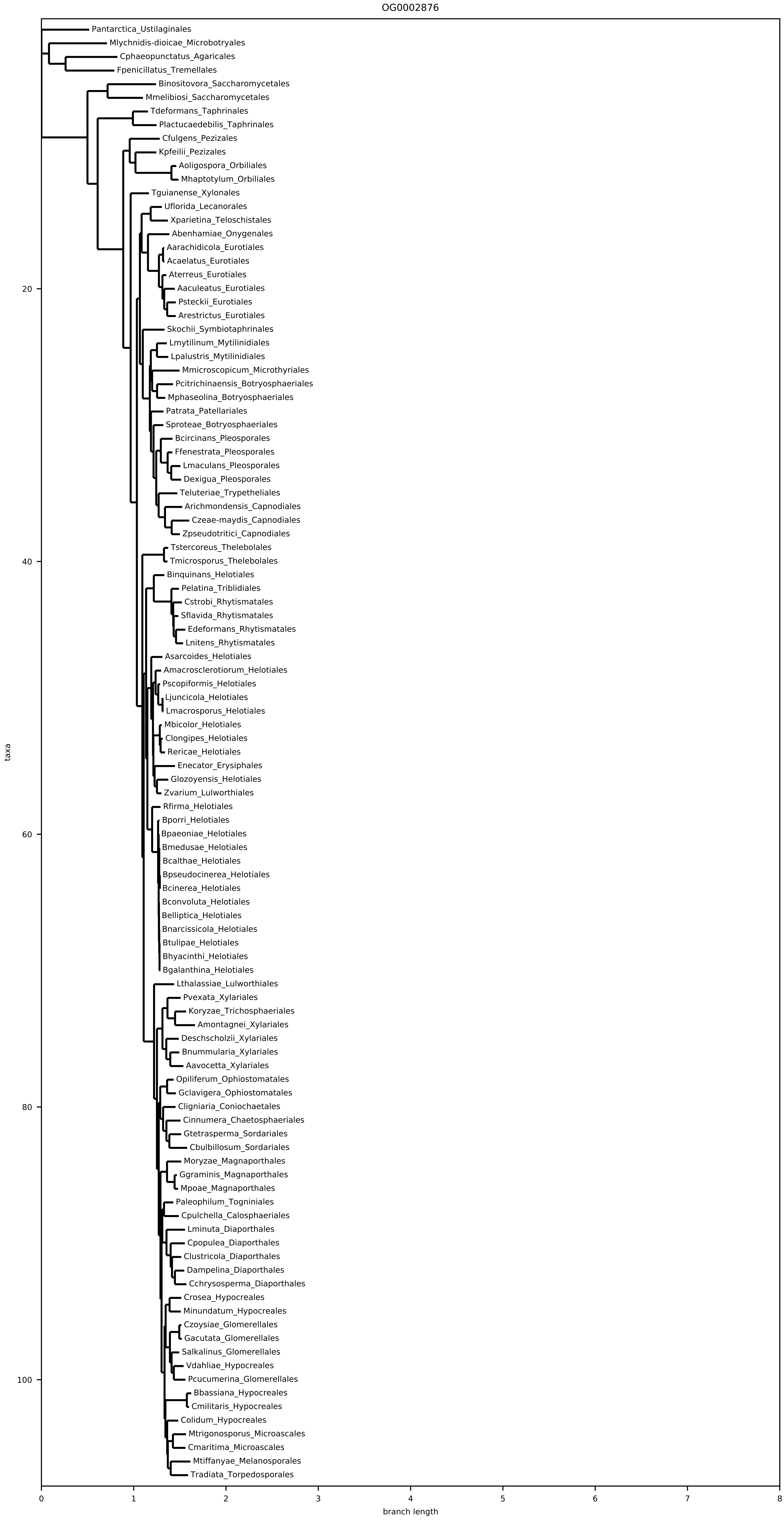

OG0002876
Pantarctica_Ustilaginales
Mlychnidis-dioicae_Microbotryales
Cphaeopunctatus_Agaricales
Fpenicillatus_Tremellales
Binositovora_Saccharomycetales
Mmelibiosi_Saccharomycetales
Tdeformans_Taphrinales
Plactucaedebilis_Taphrinales
Cfulgens_Pezizales
Kpfeilii_Pezizales
Aoligospora_Orbiliales
Mhaptotylum_Orbiliales
Tguianense_Xylonales
Uflorida_Lecanorales
Xparietina_Teloschistales
Abenhamiae_Onygenales
Aarachidicola_Eurotiales
Acaelatus_Eurotiales
Aterreus_Eurotiales
Aaculeatus_Eurotiales
Psteckii_Eurotiales
Arestrictus_Eurotiales
Skochii_Symbiotaphrinales
Lmytilinum_Mytilinidiales
Lpalustris_Mytilinidiales
Mmicroscopicum_Microthyriales
Pcitrichinaensis_Botryosphaeriales
Mphaseolina_Botryosphaeriales
Patrata_Patellariales
Sproteae_Botryosphaeriales
Bcircinans_Pleosporales
Ffenestrata_Pleosporales
Lmaculans_Pleosporales
Dexigua_Pleosporales
Teluteriae_Trypetheliales
Arichmondensis_Capnodiales
Czeae-maydis_Capnodiales
Zpseudotritici_Capnodiales
Tstercoreus_Thelebolales
Tmicrosporus_Thelebolales
Binquinans_Helotiales
Pelatina_Triblidiales
Cstrobi_Rhytismatales
Sflavida_Rhytismatales
Edeformans_Rhytismatales
Lnitens_Rhytismatales
Asarcoides_Helotiales
Amacrosclerotiorum_Helotiales
Pscopiformis_Helotiales
Ljuncicola_Helotiales
Lmacrosporus_Helotiales
Mbicolor_Helotiales
Clongipes_Helotiales
Rericae_Helotiales
Enecator_Erysiphales
Glozoyensis_Helotiales
Zvarium_Lulworthiales
Rfirma_Helotiales
Bporri_Helotiales
Bpaeoniae_Helotiales
Bmedusae_Helotiales
Bcalthae_Helotiales
Bpseudocinerea_Helotiales
Bcinerea_Helotiales
Bconvoluta_Helotiales
Belliptica_Helotiales
Bnarcissicola_Helotiales
Btulipae_Helotiales
Bhyacinthi_Helotiales
Bgalanthina_Helotiales
Lthalassiae_Lulworthiales
Pvexata_Xylariales
Koryzae_Trichosphaeriales
Amontagnei_Xylariales
Deschscholzii_Xylariales
Bnummularia_Xylariales
Aavocetta_Xylariales
Opiliferum_Ophiostomatales
Gclavigera_Ophiostomatales
Cligniaria_Coniochaetales
Cinnumera_Chaetosphaeriales
Gtetrasperma_Sordariales
Cbulbillosum_Sordariales
Moryzae_Magnaporthales
Ggraminis_Magnaporthales
Mpoae_Magnaporthales
Paleophilum_Togniniales
Cpulchella_Calosphaeriales
Lminuta_Diaporthales
Cpopulea_Diaporthales
Clustricola_Diaporthales
Dampelina_Diaporthales
Cchrysosperma_Diaporthales
Crosea_Hypocreales
Minundatum_Hypocreales
Czoysiae_Glomerellales
Gacutata_Glomerellales
Salkalinus_Glomerellales
Vdahliae_Hypocreales
Pcucumerina_Glomerellales
Bbassiana_Hypocreales
Cmilitaris_Hypocreales
Colidum_Hypocreales
Mtrigonosporus_Microascales
Cmaritima_Microascales
Mtiffanyae_Melanosporales
Tradiata_Torpedosporales
taxa
20
40
60
80
100
0
1
2
3
4
5
6
7
8
branch length

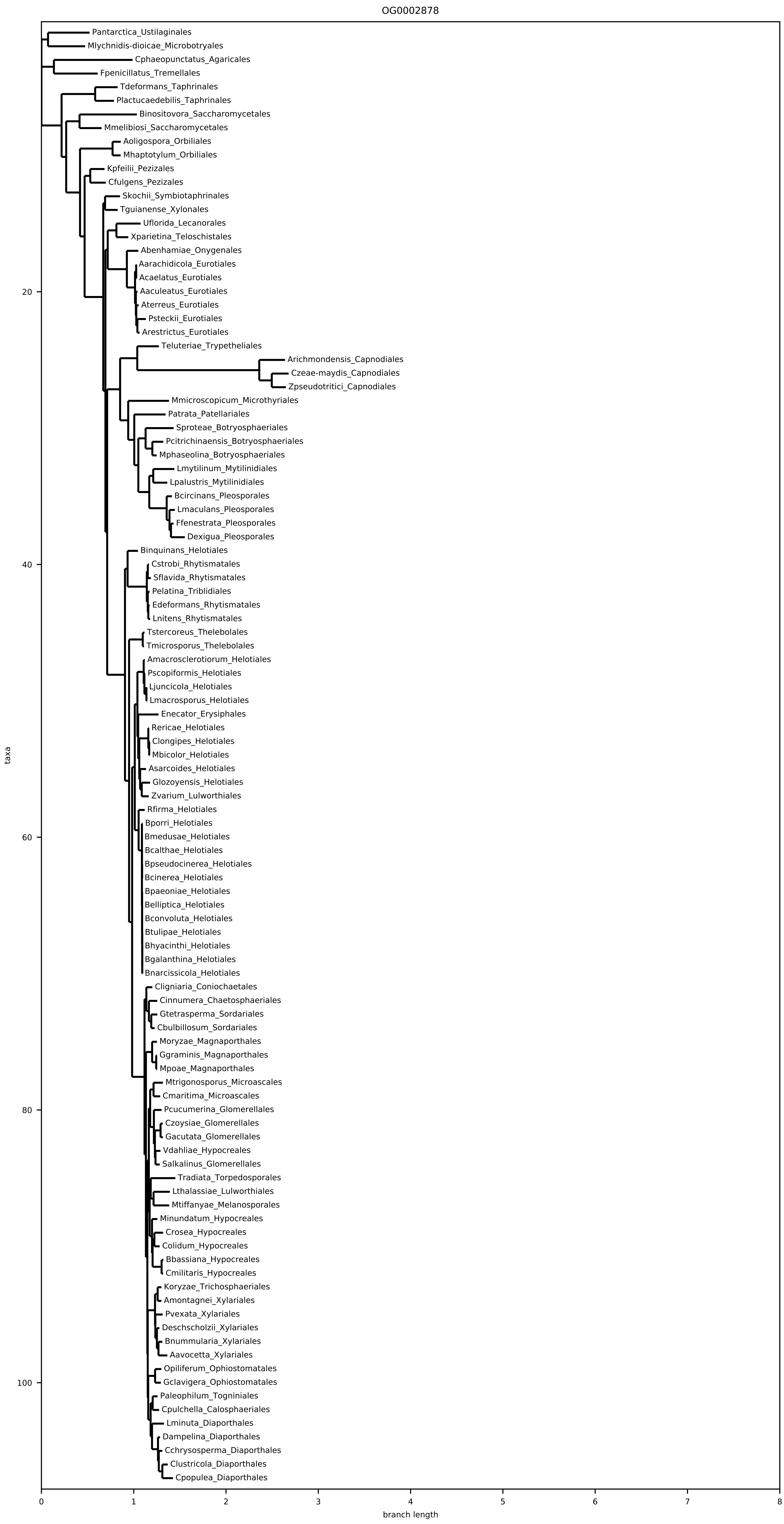
OG0002878
Pantarctica_Ustilaginales
Mlychnidis-dioicae_Microbotryales
Cphaeopunctatus_Agaricales
Fpenicillatus_Tremellales
Tdeformans_Taphrinales
Plactucaedebilis_Taphrinales
Binositovora_Saccharomycetales
Mmelibiosi_Saccharomycetales
Aoligospora_Orbiliales
Mhaptotylum_Orbiliales
Kpfeilii_Pezizales
Cfulgens_Pezizales
Skochii_Symbiotaphrinales
Tguianense_Xylonales
Uflorida_Lecanorales
Xparietina_Teloschistales
Abenhamiae_Onygenales
Aarachidicola_Eurotiales
Acaelatus_Eurotiales
Aaculeatus_Eurotiales
Aterreus_Eurotiales
Psteckii_Eurotiales
Arestrictus_Eurotiales
Teluteriae_Trypetheliales
Arichmondensis_Capnodiales
Czeae-maydis_Capnodiales
Zpseudotritici_Capnodiales
Mmicroscopicum_Microthyriales
Patrata_Patellariales
Sproteae_Botryosphaeriales
Pcitrichinaensis_Botryosphaeriales
Mphaseolina_Botryosphaeriales
Lmytilinum_Mytilinidiales
Lpalustris_Mytilinidiales
Bcircinans_Pleosporales
Lmaculans_Pleosporales
Ffenestrata_Pleosporales
Dexigua_Pleosporales
Binquinans_Helotiales
Cstrobi_Rhytismatales
Sflavida_Rhytismatales
Pelatina_Triblidiales
Edeformans_Rhytismatales
Lnitens_Rhytismatales
Tstercoreus_Thelebolales
Tmicrosporus_Thelebolales
Amacrosclerotiorum_Helotiales
Pscopiformis_Helotiales
Ljuncicola_Helotiales
Lmacrosporus_Helotiales
Enecator_Erysiphales
Rericae_Helotiales
Clongipes_Helotiales
Mbicolor_Helotiales
Asarcoides_Helotiales
Glozoyensis_Helotiales
Zvarium_Lulworthiales
Rfirma_Helotiales
Bporri_Helotiales
Bmedusae_Helotiales
Bcalthae_Helotiales
Bpseudocinerea_Helotiales
Bcinerea_Helotiales
Bpaeoniae_Helotiales
Belliptica_Helotiales
Bconvoluta_Helotiales
Btulipae_Helotiales
Bhyacinthi_Helotiales
Bgalanthina_Helotiales
Bnarcissicola_Helotiales
Cligniaria_Coniochaetales
Cinnumera_Chaetosphaeriales
Gtetrasperma_Sordariales
Cbulbillosum_Sordariales
Moryzae_Magnaporthales
Ggraminis_Magnaporthales
Mpoae_Magnaporthales
Mtrigonosporus_Microascales
Cmaritima_Microascales
Pcucumerina_Glomerellales
Czoysiae_Glomerellales
Gacutata_Glomerellales
Vdahliae_Hypocreales
Salkalinus_Glomerellales
Tradiata_Torpedosporales
Lthalassiae_Lulworthiales
Mtiffanyae_Melanosporales
Minundatum_Hypocreales
Crosea_Hypocreales
Colidum_Hypocreales
Bbassiana_Hypocreales
Cmilitaris_Hypocreales
Koryzae_Trichosphaeriales
Amontagnei_Xylariales
Pvexata_Xylariales
Deschscholzii_Xylariales
Bnummularia_Xylariales
Aavocetta_Xylariales
Opiliferum_Ophiostomatales
Gclavigera_Ophiostomatales
Paleophilum_Togniniales
Cpulchella_Calosphaeriales
Lminuta_Diaporthales
Dampelina_Diaporthales
Cchrysosperma_Diaporthales
Clustricola_Diaporthales
Cpopulea_Diaporthales
taxa
20
40
60
80
100
branch length
0
1
2
3
4
5
6
7
8

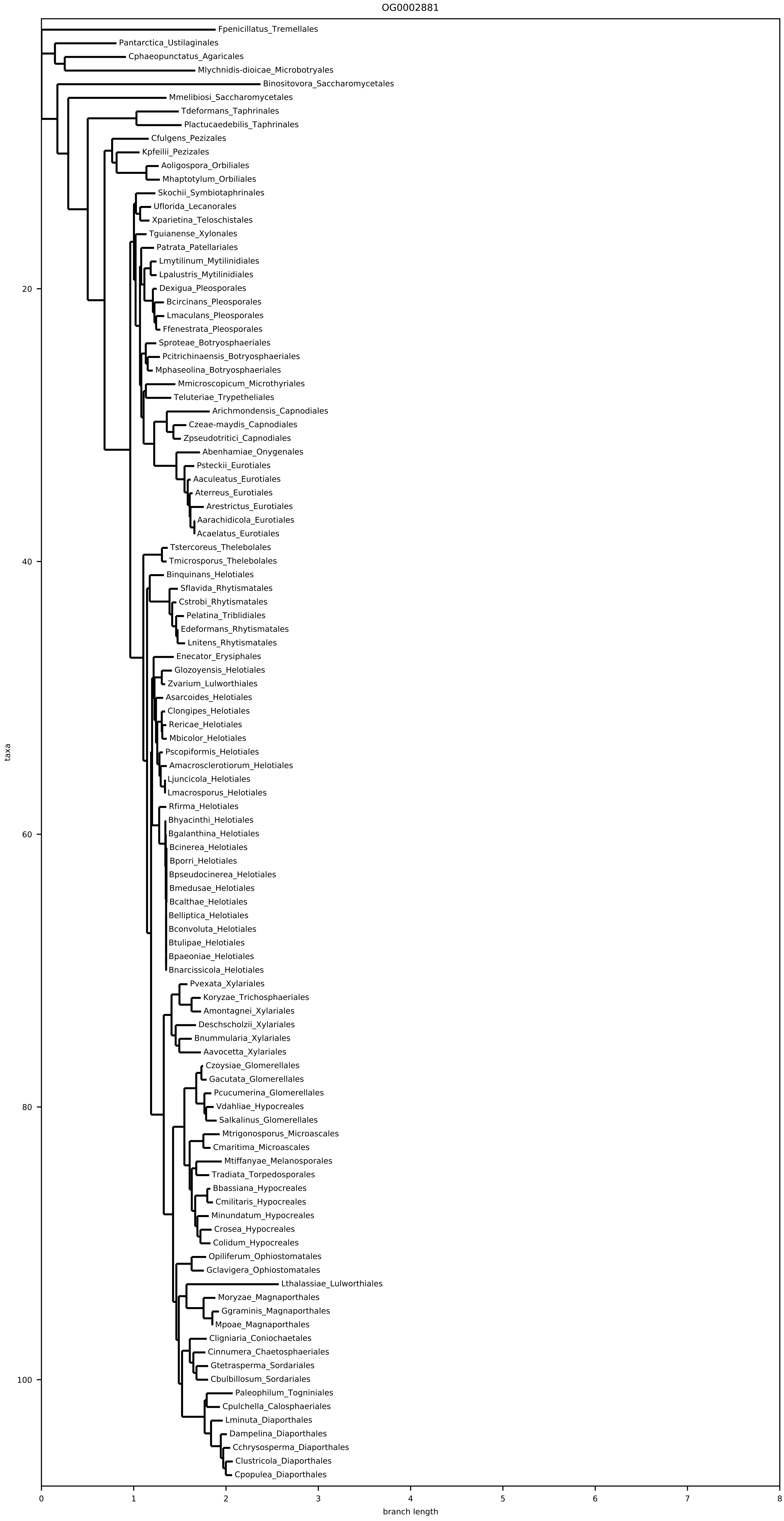

OG0002881
Fpenicillatus_Tremellales
Pantarctica_Ustilaginales
Cphaeopunctatus_Agaricales
Mlychnidis-dioicae_Microbotryales
Binositovora_Saccharomycetales
Mmelibiosi_Saccharomycetales
Tdeformans_Taphrinales
Plactucaedebilis_Taphrinales
Cfulgens_Pezizales
Kpfeilii_Pezizales
Aoligospora_Orbiliales
Mhaptotylum_Orbiliales
Skochii_Symbiotaphrinales
Uflorida_Lecanorales
Xparietina_Teloschistales
Tguianense_Xylonales
Patrata_Patellariales
Lmytilinum_Mytilinidiales
Lpalustris_Mytilinidiales
Dexigua_Pleosporales
Bcircinans_Pleosporales
Lmaculans_Pleosporales
Ffenestrata_Pleosporales
Sproteae_Botryosphaeriales
Pcitrichinaensis_Botryosphaeriales
Mphaseolina_Botryosphaeriales
Mmicroscopicum_Microthyriales
Teluteriae_Trypetheliales
Arichmondensis_Capnodiales
Czeae-maydis_Capnodiales
Zpseudotritici_Capnodiales
Abenhamiae_Onygenales
Psteckii_Eurotiales
Aaculeatus_Eurotiales
Aterreus_Eurotiales
Arestrictus_Eurotiales
Aarachidicola_Eurotiales
Acaelatus_Eurotiales
Tstercoreus_Thelebolales
Tmicrosporus_Thelebolales
Binquinans_Helotiales
Sflavida_Rhytismatales
Cstrobi_Rhytismatales
Pelatina_Triblidiales
Edeformans_Rhytismatales
Lnitens_Rhytismatales
Enecator_Erysiphales
Glozoyensis_Helotiales
Zvarium_Lulworthiales
Asarcoides_Helotiales
Clongipes_Helotiales
Rericae_Helotiales
Mbicolor_Helotiales
Pscopiformis_Helotiales
Amacrosclerotiorum_Helotiales
Ljuncicola_Helotiales
Lmacrosporus_Helotiales
Rfirma_Helotiales
Bhyacinthi_Helotiales
Bgalanthina_Helotiales
Bcinerea_Helotiales
Bporri_Helotiales
Bpseudocinerea_Helotiales
Bmedusae_Helotiales
Bcalthae_Helotiales
Belliptica_Helotiales
Bconvoluta_Helotiales
Btulipae_Helotiales
Bpaeoniae_Helotiales
Bnarcissicola_Helotiales
Pvexata_Xylariales
Koryzae_Trichosphaeriales
Amontagnei_Xylariales
Deschscholzii_Xylariales
Bnummularia_Xylariales
Aavocetta_Xylariales
Czoysiae_Glomerellales
Gacutata_Glomerellales
Pcucumerina_Glomerellales
Vdahliae_Hypocreales
Salkalinus_Glomerellales
Mtrigonosporus_Microascales
Cmaritima_Microascales
Mtiffanyae_Melanosporales
Tradiata_Torpedosporales
Bbassiana_Hypocreales
Cmilitaris_Hypocreales
Minundatum_Hypocreales
Crosea_Hypocreales
Colidum_Hypocreales
Opiliferum_Ophiostomatales
Gclavigera_Ophiostomatales
Lthalassiae_Lulworthiales
Moryzae_Magnaporthales
Ggraminis_Magnaporthales
Mpoae_Magnaporthales
Cligniaria_Coniochaetales
Cinnumera_Chaetosphaeriales
Gtetrasperma_Sordariales
Cbulbillosum_Sordariales
Paleophilum_Togniniales
Cpulchella_Calosphaeriales
Lminuta_Diaporthales
Dampelina_Diaporthales
Cchrysosperma_Diaporthales
Clustricola_Diaporthales
Cpopulea_Diaporthales
taxa
20
40
60
80
100
0
1
2
3
4
5
6
7
8
branch length

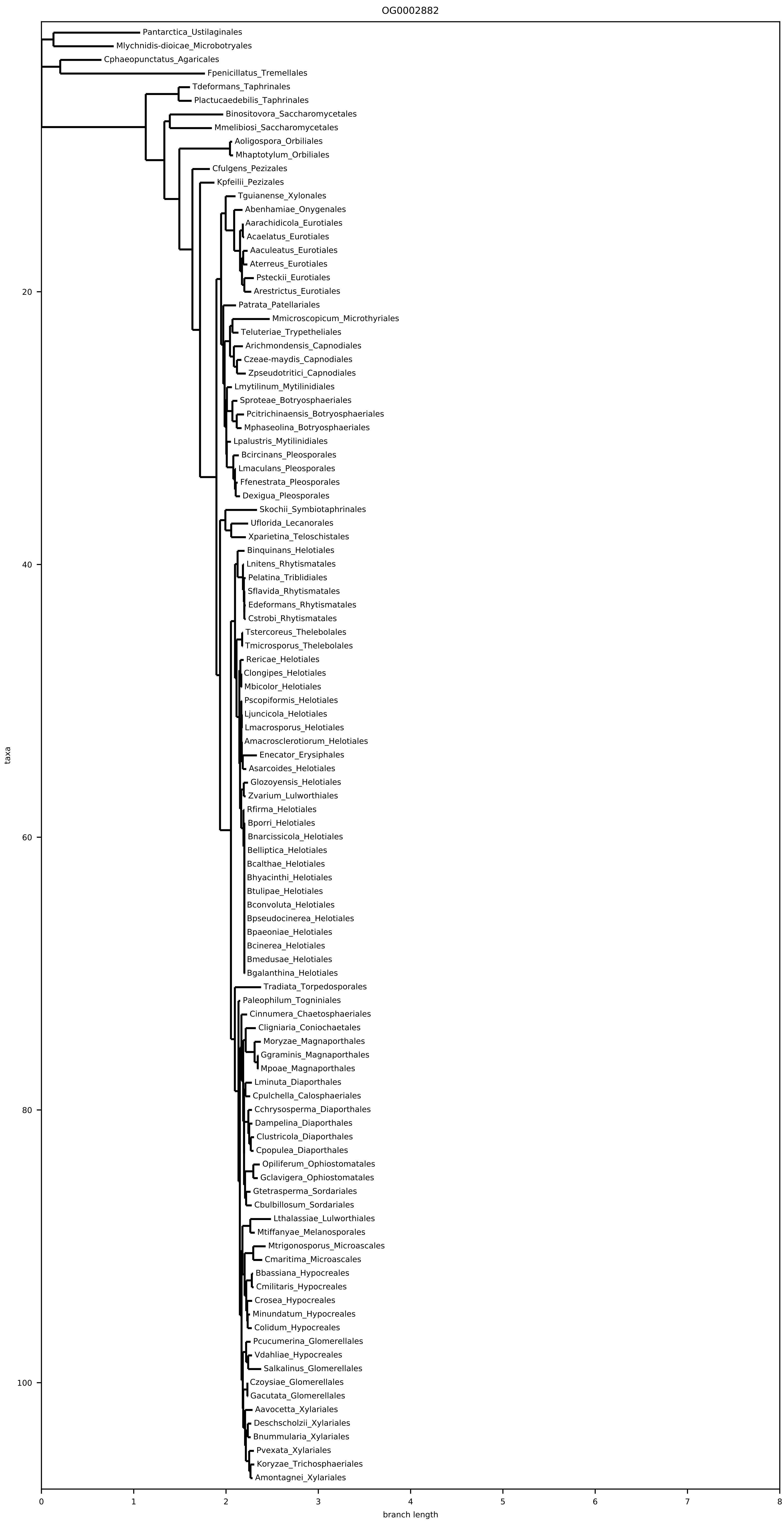
OG0002882
Pantarctica_Ustilaginales
Mlychnidis-dioicae_Microbotryales
Cphaeopunctatus_Agaricales
Fpenicillatus_Tremellales
Tdeformans_Taphrinales
Plactucaedebilis_Taphrinales
Binositovora_Saccharomycetales
Mmelibiosi_Saccharomycetales
Aoligospora_Orbiliales
Mhaptotylum_Orbiliales
Cfulgens_Pezizales
Kpfeilii_Pezizales
Tguianense_Xylonales
Abenhamiae_Onygenales
Aarachidicola_Eurotiales
Acaelatus_Eurotiales
Aaculeatus_Eurotiales
Aterreus_Eurotiales
Psteckii_Eurotiales
Arestrictus_Eurotiales
Patrata_Patellariales
Mmicroscopicum_Microthyriales
Teluteriae_Trypetheliales
Arichmondensis_Capnodiales
Czeae-maydis_Capnodiales
Zpseudotritici_Capnodiales
Lmytilinum_Mytilinidiales
Sproteae_Botryosphaeriales
Pcitrichinaensis_Botryosphaeriales
Mphaseolina_Botryosphaeriales
Lpalustris_Mytilinidiales
Bcircinans_Pleosporales
Lmaculans_Pleosporales
Ffenestrata_Pleosporales
Dexigua_Pleosporales
Skochii_Symbiotaphrinales
Uflorida_Lecanorales
Xparietina_Teloschistales
Binquinans_Helotiales
Lnitens_Rhytismatales
Pelatina_Triblidiales
Sflavida_Rhytismatales
Edeformans_Rhytismatales
Cstrobi_Rhytismatales
Tstercoreus_Thelebolales
Tmicrosporus_Thelebolales
Rericae_Helotiales
Clongipes_Helotiales
Mbicolor_Helotiales
Pscopiformis_Helotiales
Ljuncicola_Helotiales
Lmacrosporus_Helotiales
Amacrosclerotiorum_Helotiales
Enecator_Erysiphales
Asarcoides_Helotiales
Glozoyensis_Helotiales
Zvarium_Lulworthiales
Rfirma_Helotiales
Bporri_Helotiales
Bnarcissicola_Helotiales
Belliptica_Helotiales
Bcalthae_Helotiales
Bhyacinthi_Helotiales
Btulipae_Helotiales
Bconvoluta_Helotiales
Bpseudocinerea_Helotiales
Bpaeoniae_Helotiales
Bcinerea_Helotiales
Bmedusae_Helotiales
Bgalanthina_Helotiales
Tradiata_Torpedosporales
Paleophilum_Togniniales
Cinnumera_Chaetosphaeriales
Cligniaria_Coniochaetales
Moryzae_Magnaporthales
Ggraminis_Magnaporthales
Mpoae_Magnaporthales
Lminuta_Diaporthales
Cpulchella_Calosphaeriales
Cchrysosperma_Diaporthales
Dampelina_Diaporthales
Clustricola_Diaporthales
Cpopulea_Diaporthales
Opiliferum_Ophiostomatales
Gclavigera_Ophiostomatales
Gtetrasperma_Sordariales
Cbulbillosum_Sordariales
Lthalassiae_Lulworthiales
Mtiffanyae_Melanosporales
Mtrigonosporus_Microascales
Cmaritima_Microascales
Bbassiana_Hypocreales
Cmilitaris_Hypocreales
Crosea_Hypocreales
Minundatum_Hypocreales
Colidum_Hypocreales
Pcucumerina_Glomerellales
Vdahliae_Hypocreales
Salkalinus_Glomerellales
Czoysiae_Glomerellales
Gacutata_Glomerellales
Aavocetta_Xylariales
Deschscholzii_Xylariales
Bnummularia_Xylariales
Pvexata_Xylariales
Koryzae_Trichosphaeriales
Amontagnei_Xylariales
taxa
20
40
60
80
100
0
1
2
3
4
5
6
7
8
branch length

OG0002885

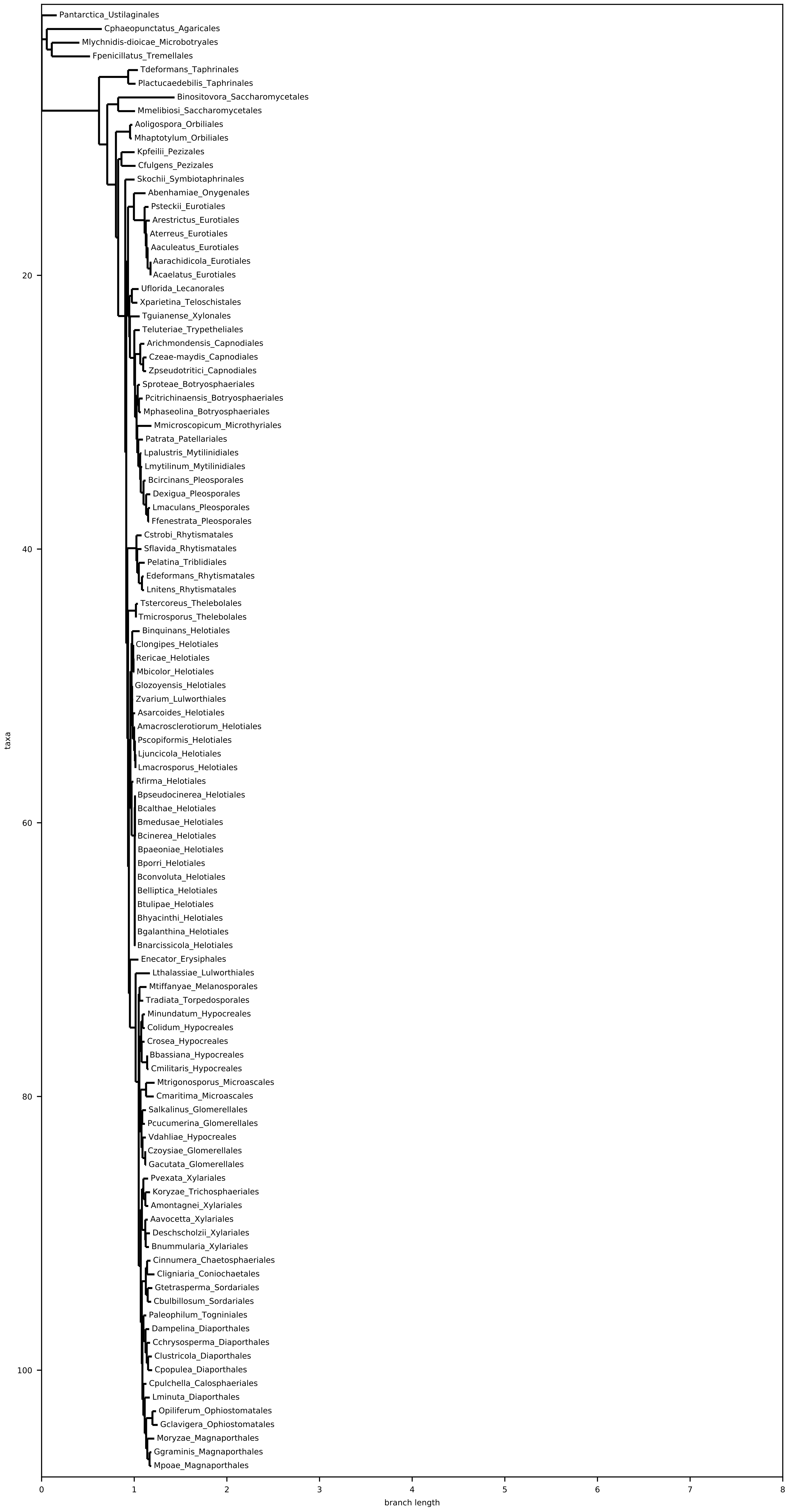

Pantarctica_Ustilaginales
Cphaeopunctatus_Agaricales
Mlychnidis-dioicae_Microbotryales
Fpenicillatus_Tremellales
Tdeformans_Taphrinales
Plactucaedebilis_Taphrinales
Binositovora_Saccharomycetales
Mmelibiosi_Saccharomycetales
Aoligospora_Orbiliales
Mhaptotylum_Orbiliales
Kpfeilii_Pezizales
Cfulgens_Pezizales
Skochii_Symbiotaphrinales
Abenhamiae_Onygenales
Psteckii_Eurotiales
Arestrictus_Eurotiales
Aterreus_Eurotiales
Aaculeatus_Eurotiales
Aarachidicola_Eurotiales
Acaelatus_Eurotiales
Uflorida_Lecanorales
Xparietina_Teloschistales
Tguianense_Xylonales
Teluteriae_Trypetheliales
Arichmondensis_Capnodiales
Czeae-maydis_Capnodiales
Zpseudotritici_Capnodiales
Sproteae_Botryosphaeriales
Pcitrichinaensis_Botryosphaeriales
Mphaseolina_Botryosphaeriales
Mmicroscopicum_Microthyriales
Patrata_Patellariales
Lpalustris_Mytilinidiales
Lmytilinum_Mytilinidiales
Bcircinans_Pleosporales
Dexigua_Pleosporales
Lmaculans_Pleosporales
Ffenestrata_Pleosporales
Cstrobi_Rhytismatales
Sflavida_Rhytismatales
Pelatina_Triblidiales
Edeformans_Rhytismatales
Lnitens_Rhytismatales
Tstercoreus_Thelebolales
Tmicrosporus_Thelebolales
Binquinans_Helotiales
Clongipes_Helotiales
Rericae_Helotiales
Mbicolor_Helotiales
Glozoyensis_Helotiales
Zvarium_Lulworthiales
Asarcoides_Helotiales
Amacrosclerotiorum_Helotiales
Pscopiformis_Helotiales
Ljuncicola_Helotiales
Lmacrosporus_Helotiales
Rfirma_Helotiales
Bpseudocinerea_Helotiales
Bcalthae_Helotiales
Bmedusae_Helotiales
Bcinerea_Helotiales
Bpaeoniae_Helotiales
Bporri_Helotiales
Bconvoluta_Helotiales
Belliptica_Helotiales
Btulipae_Helotiales
Bhyacinthi_Helotiales
Bgalanthina_Helotiales
Bnarcissicola_Helotiales
Enecator_Erysiphales
Lthalassiae_Lulworthiales
Mtiffanyae_Melanosporales
Tradiata_Torpedosporales
Minundatum_Hypocreales
Colidum_Hypocreales
Crosea_Hypocreales
Bbassiana_Hypocreales
Cmilitaris_Hypocreales
Mtrigonosporus_Microascales
Cmaritima_Microascales
Salkalinus_Glomerellales
Pcucumerina_Glomerellales
Vdahliae_Hypocreales
Czoysiae_Glomerellales
Gacutata_Glomerellales
Pvexata_Xylariales
Koryzae_Trichosphaeriales
Amontagnei_Xylariales
Aavocetta_Xylariales
Deschscholzii_Xylariales
Bnummularia_Xylariales
Cinnumera_Chaetosphaeriales
Cligniaria_Coniochaetales
Gtetrasperma_Sordariales
Cbulbillosum_Sordariales
Paleophilum_Togniniales
Dampelina_Diaporthales
Cchrysosperma_Diaporthales
Clustricola_Diaporthales
Cpopulea_Diaporthales
Cpulchella_Calosphaeriales
Lminuta_Diaporthales
Opiliferum_Ophiostomatales
Gclavigera_Ophiostomatales
Moryzae_Magnaporthales
Ggraminis_Magnaporthales
Mpoae_Magnaporthales
taxa
20
40
60
80
100
0
1
2
3
4
5
6
7
8
branch length

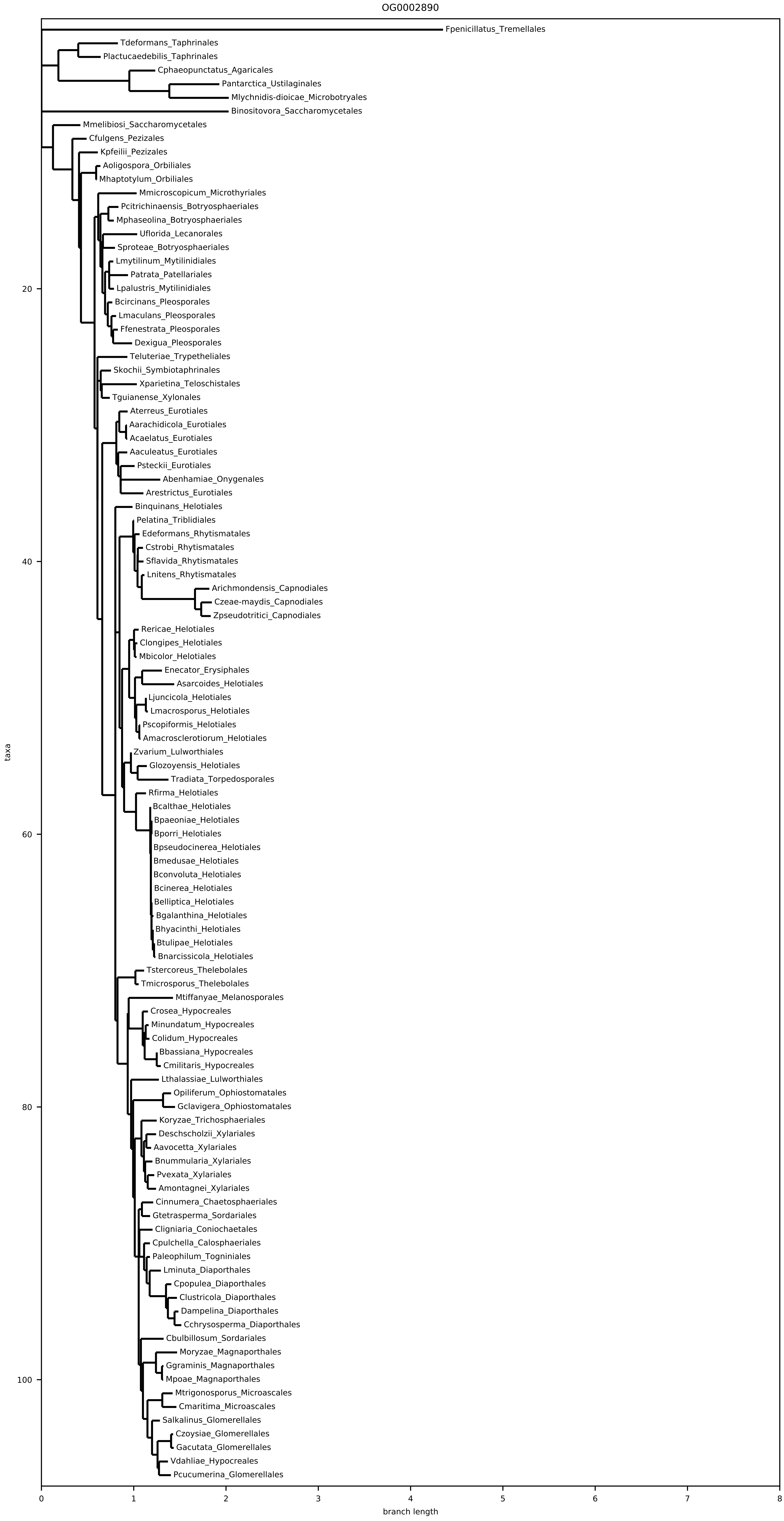

OG0002890
Fpenicillatus_Tremellales
Tdeformans_Taphrinales
Plactucaedebilis_Taphrinales
Cphaeopunctatus_Agaricales
Pantarctica_Ustilaginales
Mlychnidis-dioicae_Microbotryales
Binositovora_Saccharomycetales
Mmelibiosi_Saccharomycetales
Cfulgens_Pezizales
Kpfeilii_Pezizales
Aoligospora_Orbiliales
Mhaptotylum_Orbiliales
Mmicroscopicum_Microthyriales
Pcitrichinaensis_Botryosphaeriales
Mphaseolina_Botryosphaeriales
Uflorida_Lecanorales
Sproteae_Botryosphaeriales
Lmytilinum_Mytilinidiales
Patrata_Patellariales
Lpalustris_Mytilinidiales
Bcircinans_Pleosporales
Lmaculans_Pleosporales
Ffenestrata_Pleosporales
Dexigua_Pleosporales
Teluteriae_Trypetheliales
Skochii_Symbiotaphrinales
Xparietina_Teloschistales
Tguianense_Xylonales
Aterreus_Eurotiales
Aarachidicola_Eurotiales
Acaelatus_Eurotiales
Aaculeatus_Eurotiales
Psteckii_Eurotiales
Abenhamiae_Onygenales
Arestrictus_Eurotiales
Binquinans_Helotiales
Pelatina_Triblidiales
Edeformans_Rhytismatales
Cstrobi_Rhytismatales
Sflavida_Rhytismatales
Lnitens_Rhytismatales
Arichmondensis_Capnodiales
Czeae-maydis_Capnodiales
Zpseudotritici_Capnodiales
Rericae_Helotiales
Clongipes_Helotiales
Mbicolor_Helotiales
Enecator_Erysiphales
Asarcoides_Helotiales
Ljuncicola_Helotiales
Lmacrosporus_Helotiales
Pscopiformis_Helotiales
Amacrosclerotiorum_Helotiales
Zvarium_Lulworthiales
Glozoyensis_Helotiales
Tradiata_Torpedosporales
Rfirma_Helotiales
Bcalthae_Helotiales
Bpaeoniae_Helotiales
Bporri_Helotiales
Bpseudocinerea_Helotiales
Bmedusae_Helotiales
Bconvoluta_Helotiales
Bcinerea_Helotiales
Belliptica_Helotiales
Bgalanthina_Helotiales
Bhyacinthi_Helotiales
Btulipae_Helotiales
Bnarcissicola_Helotiales
Tstercoreus_Thelebolales
Tmicrosporus_Thelebolales
Mtiffanyae_Melanosporales
Crosea_Hypocreales
Minundatum_Hypocreales
Colidum_Hypocreales
Bbassiana_Hypocreales
Cmilitaris_Hypocreales
Lthalassiae_Lulworthiales
Opiliferum_Ophiostomatales
Gclavigera_Ophiostomatales
Koryzae_Trichosphaeriales
Deschscholzii_Xylariales
Aavocetta_Xylariales
Bnummularia_Xylariales
Pvexata_Xylariales
Amontagnei_Xylariales
Cinnumera_Chaetosphaeriales
Gtetrasperma_Sordariales
Cligniaria_Coniochaetales
Cpulchella_Calosphaeriales
Paleophilum_Togniniales
Lminuta_Diaporthales
Cpopulea_Diaporthales
Clustricola_Diaporthales
Dampelina_Diaporthales
Cchrysosperma_Diaporthales
Cbulbillosum_Sordariales
Moryzae_Magnaporthales
Ggraminis_Magnaporthales
Mpoae_Magnaporthales
Mtrigonosporus_Microascales
Cmaritima_Microascales
Salkalinus_Glomerellales
Czoysiae_Glomerellales
Gacutata_Glomerellales
Vdahliae_Hypocreales
Pcucumerina_Glomerellales
taxa
20
40
60
80
100
0
1
2
3
4
5
6
7
8
branch length

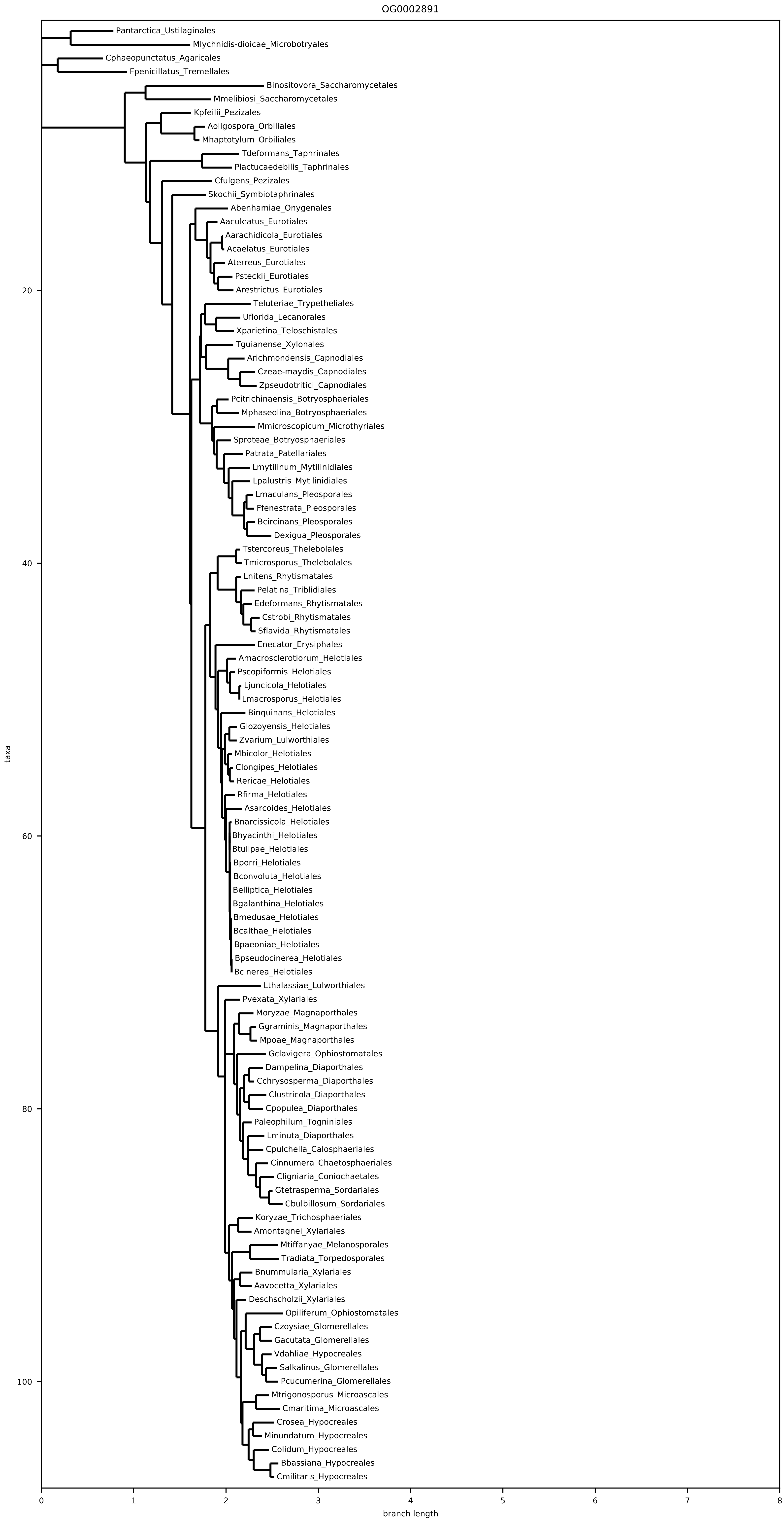
OG0002891
Pantarctica_Ustilaginales
Mlychnidis-dioicae_Microbotryales
Cphaeopunctatus_Agaricales
Fpenicillatus_Tremellales
Binositovora_Saccharomycetales
Mmelibiosi_Saccharomycetales
Kpfeilii_Pezizales
Aoligospora_Orbiliales
Mhaptotylum_Orbiliales
Tdeformans_Taphrinales
Plactucaedebilis_Taphrinales
Cfulgens_Pezizales
Skochii_Symbiotaphrinales
Abenhamiae_Onygenales
Aaculeatus_Eurotiales
Aarachidicola_Eurotiales
Acaelatus_Eurotiales
Aterreus_Eurotiales
Psteckii_Eurotiales
Arestrictus_Eurotiales
Teluteriae_Trypetheliales
Uflorida_Lecanorales
Xparietina_Teloschistales
Tguianense_Xylonales
Arichmondensis_Capnodiales
Czeae-maydis_Capnodiales
Zpseudotritici_Capnodiales
Pcitrichinaensis_Botryosphaeriales
Mphaseolina_Botryosphaeriales
Mmicroscopicum_Microthyriales
Sproteae_Botryosphaeriales
Patrata_Patellariales
Lmytilinum_Mytilinidiales
Lpalustris_Mytilinidiales
Lmaculans_Pleosporales
Ffenestrata_Pleosporales
Bcircinans_Pleosporales
Dexigua_Pleosporales
Tstercoreus_Thelebolales
Tmicrosporus_Thelebolales
Lnitens_Rhytismatales
Pelatina_Triblidiales
Edeformans_Rhytismatales
Cstrobi_Rhytismatales
Sflavida_Rhytismatales
Enecator_Erysiphales
Amacrosclerotiorum_Helotiales
Pscopiformis_Helotiales
Ljuncicola_Helotiales
Lmacrosporus_Helotiales
Binquinans_Helotiales
Glozoyensis_Helotiales
Zvarium_Lulworthiales
Mbicolor_Helotiales
Clongipes_Helotiales
Rericae_Helotiales
Rfirma_Helotiales
Asarcoides_Helotiales
Bnarcissicola_Helotiales
Bhyacinthi_Helotiales
Btulipae_Helotiales
Bporri_Helotiales
Bconvoluta_Helotiales
Belliptica_Helotiales
Bgalanthina_Helotiales
Bmedusae_Helotiales
Bcalthae_Helotiales
Bpaeoniae_Helotiales
Bpseudocinerea_Helotiales
Bcinerea_Helotiales
Lthalassiae_Lulworthiales
Pvexata_Xylariales
Moryzae_Magnaporthales
Ggraminis_Magnaporthales
Mpoae_Magnaporthales
Gclavigera_Ophiostomatales
Dampelina_Diaporthales
Cchrysosperma_Diaporthales
Clustricola_Diaporthales
Cpopulea_Diaporthales
Paleophilum_Togniniales
Lminuta_Diaporthales
Cpulchella_Calosphaeriales
Cinnumera_Chaetosphaeriales
Cligniaria_Coniochaetales
Gtetrasperma_Sordariales
Cbulbillosum_Sordariales
Koryzae_Trichosphaeriales
Amontagnei_Xylariales
Mtiffanyae_Melanosporales
Tradiata_Torpedosporales
Bnummularia_Xylariales
Aavocetta_Xylariales
Deschscholzii_Xylariales
Opiliferum_Ophiostomatales
Czoysiae_Glomerellales
Gacutata_Glomerellales
Vdahliae_Hypocreales
Salkalinus_Glomerellales
Pcucumerina_Glomerellales
Mtrigonosporus_Microascales
Cmaritima_Microascales
Crosea_Hypocreales
Minundatum_Hypocreales
Colidum_Hypocreales
Bbassiana_Hypocreales
Cmilitaris_Hypocreales
taxa
20
40
60
80
100
0
1
2
3
4
5
6
7
8
branch length

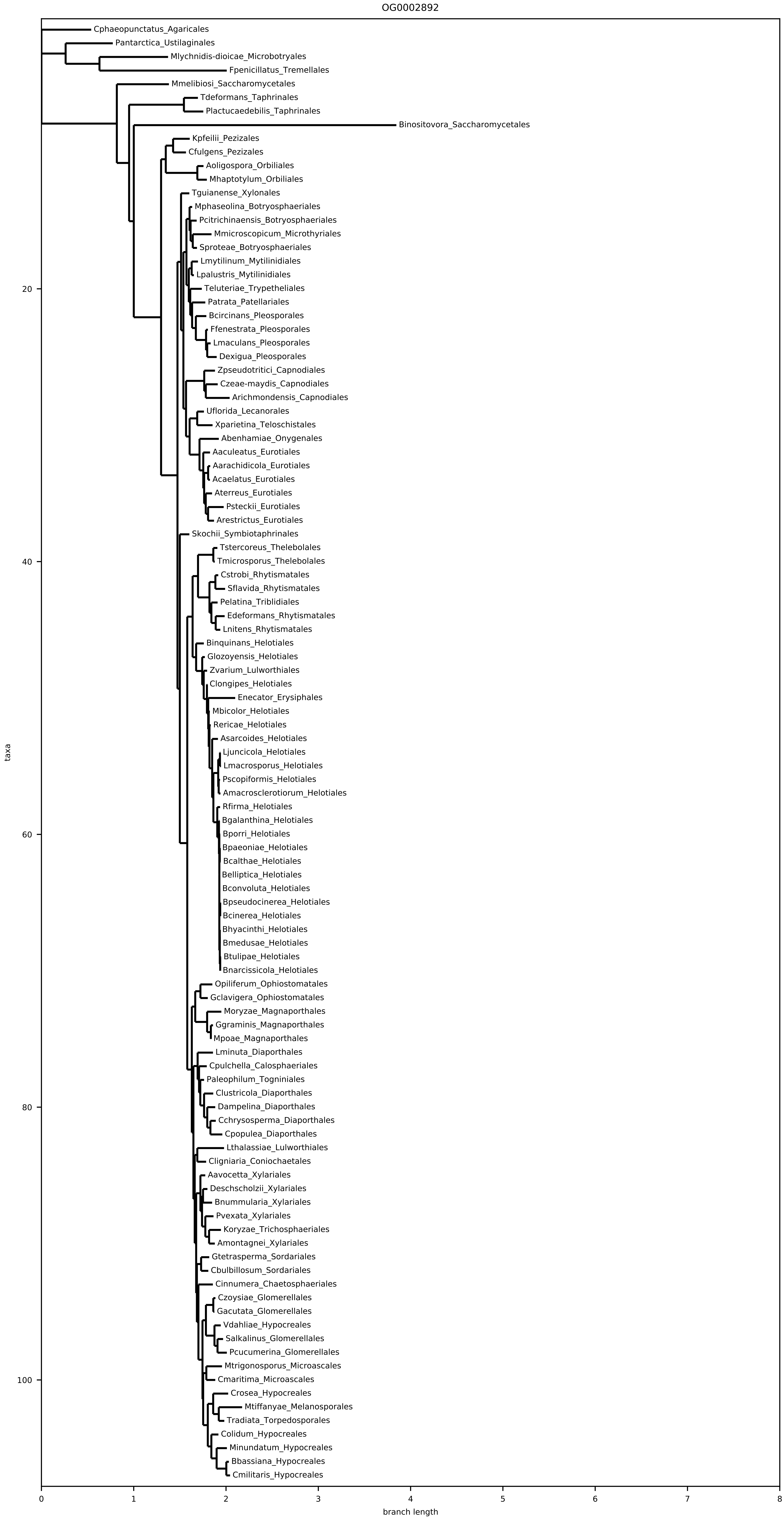

OG0002892
Cphaeopunctatus_Agaricales
Pantarctica_Ustilaginales
Mlychnidis-dioicae_Microbotryales
Fpenicillatus_Tremellales
Mmelibiosi_Saccharomycetales
Tdeformans_Taphrinales
Plactucaedebilis_Taphrinales
Binositovora_Saccharomycetales
Kpfeilii_Pezizales
Cfulgens_Pezizales
Aoligospora_Orbiliales
Mhaptotylum_Orbiliales
Tguianense_Xylonales
Mphaseolina_Botryosphaeriales
Pcitrichinaensis_Botryosphaeriales
Mmicroscopicum_Microthyriales
Sproteae_Botryosphaeriales
Lmytilinum_Mytilinidiales
Lpalustris_Mytilinidiales
Teluteriae_Trypetheliales
Patrata_Patellariales
Bcircinans_Pleosporales
Ffenestrata_Pleosporales
Lmaculans_Pleosporales
Dexigua_Pleosporales
Zpseudotritici_Capnodiales
Czeae-maydis_Capnodiales
Arichmondensis_Capnodiales
Uflorida_Lecanorales
Xparietina_Teloschistales
Abenhamiae_Onygenales
Aaculeatus_Eurotiales
Aarachidicola_Eurotiales
Acaelatus_Eurotiales
Aterreus_Eurotiales
Psteckii_Eurotiales
Arestrictus_Eurotiales
Skochii_Symbiotaphrinales
Tstercoreus_Thelebolales
Tmicrosporus_Thelebolales
Cstrobi_Rhytismatales
Sflavida_Rhytismatales
Pelatina_Triblidiales
Edeformans_Rhytismatales
Lnitens_Rhytismatales
Binquinans_Helotiales
Glozoyensis_Helotiales
Zvarium_Lulworthiales
Clongipes_Helotiales
Enecator_Erysiphales
Mbicolor_Helotiales
Rericae_Helotiales
Asarcoides_Helotiales
Ljuncicola_Helotiales
Lmacrosporus_Helotiales
Pscopiformis_Helotiales
Amacrosclerotiorum_Helotiales
Rfirma_Helotiales
Bgalanthina_Helotiales
Bporri_Helotiales
Bpaeoniae_Helotiales
Bcalthae_Helotiales
Belliptica_Helotiales
Bconvoluta_Helotiales
Bpseudocinerea_Helotiales
Bcinerea_Helotiales
Bhyacinthi_Helotiales
Bmedusae_Helotiales
Btulipae_Helotiales
Bnarcissicola_Helotiales
Opiliferum_Ophiostomatales
Gclavigera_Ophiostomatales
Moryzae_Magnaporthales
Ggraminis_Magnaporthales
Mpoae_Magnaporthales
Lminuta_Diaporthales
Cpulchella_Calosphaeriales
Paleophilum_Togniniales
Clustricola_Diaporthales
Dampelina_Diaporthales
Cchrysosperma_Diaporthales
Cpopulea_Diaporthales
Lthalassiae_Lulworthiales
Cligniaria_Coniochaetales
Aavocetta_Xylariales
Deschscholzii_Xylariales
Bnummularia_Xylariales
Pvexata_Xylariales
Koryzae_Trichosphaeriales
Amontagnei_Xylariales
Gtetrasperma_Sordariales
Cbulbillosum_Sordariales
Cinnumera_Chaetosphaeriales
Czoysiae_Glomerellales
Gacutata_Glomerellales
Vdahliae_Hypocreales
Salkalinus_Glomerellales
Pcucumerina_Glomerellales
Mtrigonosporus_Microascales
Cmaritima_Microascales
Crosea_Hypocreales
Mtiffanyae_Melanosporales
Tradiata_Torpedosporales
Colidum_Hypocreales
Minundatum_Hypocreales
Bbassiana_Hypocreales
Cmilitaris_Hypocreales
taxa
20
40
60
80
100
0
1
2
3
4
5
6
7
8
branch length

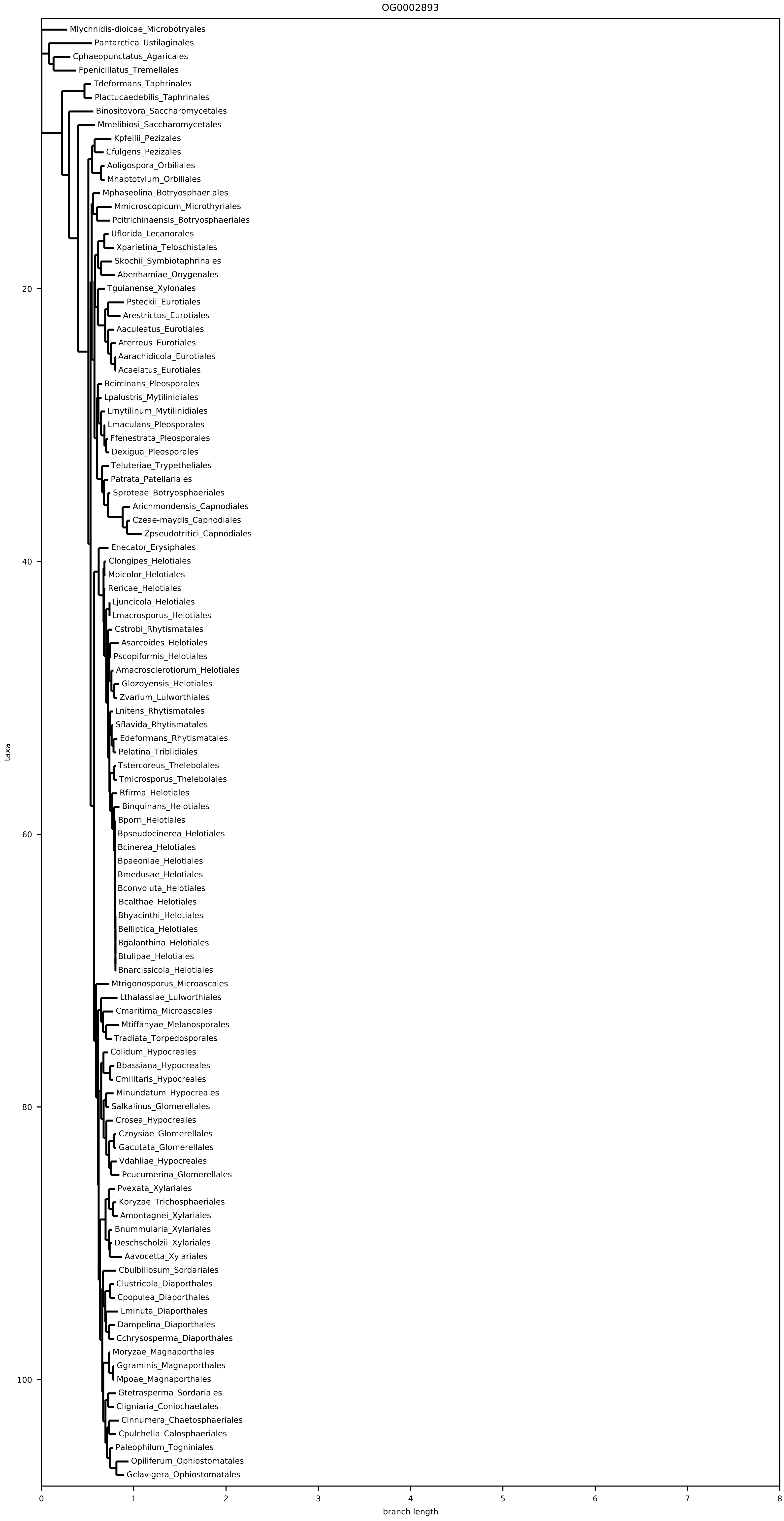

OG0002893
Mlychnidis-dioicae_Microbotryales
Pantarctica_Ustilaginales
Cphaeopunctatus_Agaricales
Fpenicillatus_Tremellales
Tdeformans_Taphrinales
Plactucaedebilis_Taphrinales
Binositovora_Saccharomycetales
Mmelibiosi_Saccharomycetales
Kpfeilii_Pezizales
Cfulgens_Pezizales
Aoligospora_Orbiliales
Mhaptotylum_Orbiliales
Mphaseolina_Botryosphaeriales
Mmicroscopicum_Microthyriales
Pcitrichinaensis_Botryosphaeriales
Uflorida_Lecanorales
Xparietina_Teloschistales
Skochii_Symbiotaphrinales
Abenhamiae_Onygenales
Tguianense_Xylonales
Psteckii_Eurotiales
Arestrictus_Eurotiales
Aaculeatus_Eurotiales
Aterreus_Eurotiales
Aarachidicola_Eurotiales
Acaelatus_Eurotiales
Bcircinans_Pleosporales
Lpalustris_Mytilinidiales
Lmytilinum_Mytilinidiales
Lmaculans_Pleosporales
Ffenestrata_Pleosporales
Dexigua_Pleosporales
Teluteriae_Trypetheliales
Patrata_Patellariales
Sproteae_Botryosphaeriales
Arichmondensis_Capnodiales
Czeae-maydis_Capnodiales
Zpseudotritici_Capnodiales
Enecator_Erysiphales
Clongipes_Helotiales
Mbicolor_Helotiales
Rericae_Helotiales
Ljuncicola_Helotiales
Lmacrosporus_Helotiales
Cstrobi_Rhytismatales
Asarcoides_Helotiales
Pscopiformis_Helotiales
Amacrosclerotiorum_Helotiales
Glozoyensis_Helotiales
Zvarium_Lulworthiales
Lnitens_Rhytismatales
Sflavida_Rhytismatales
Edeformans_Rhytismatales
Pelatina_Triblidiales
Tstercoreus_Thelebolales
Tmicrosporus_Thelebolales
Rfirma_Helotiales
Binquinans_Helotiales
Bporri_Helotiales
Bpseudocinerea_Helotiales
Bcinerea_Helotiales
Bpaeoniae_Helotiales
Bmedusae_Helotiales
Bconvoluta_Helotiales
Bcalthae_Helotiales
Bhyacinthi_Helotiales
Belliptica_Helotiales
Bgalanthina_Helotiales
Btulipae_Helotiales
Bnarcissicola_Helotiales
Mtrigonosporus_Microascales
Lthalassiae_Lulworthiales
Cmaritima_Microascales
Mtiffanyae_Melanosporales
Tradiata_Torpedosporales
Colidum_Hypocreales
Bbassiana_Hypocreales
Cmilitaris_Hypocreales
Minundatum_Hypocreales
Salkalinus_Glomerellales
Crosea_Hypocreales
Czoysiae_Glomerellales
Gacutata_Glomerellales
Vdahliae_Hypocreales
Pcucumerina_Glomerellales
Pvexata_Xylariales
Koryzae_Trichosphaeriales
Amontagnei_Xylariales
Bnummularia_Xylariales
Deschscholzii_Xylariales
Aavocetta_Xylariales
Cbulbillosum_Sordariales
Clustricola_Diaporthales
Cpopulea_Diaporthales
Lminuta_Diaporthales
Dampelina_Diaporthales
Cchrysosperma_Diaporthales
Moryzae_Magnaporthales
Ggraminis_Magnaporthales
Mpoae_Magnaporthales
Gtetrasperma_Sordariales
Cligniaria_Coniochaetales
Cinnumera_Chaetosphaeriales
Cpulchella_Calosphaeriales
Paleophilum_Togniniales
Opiliferum_Ophiostomatales
Gclavigera_Ophiostomatales
taxa
20
40
60
80
100
0
1
2
3
4
5
6
7
8
branch length

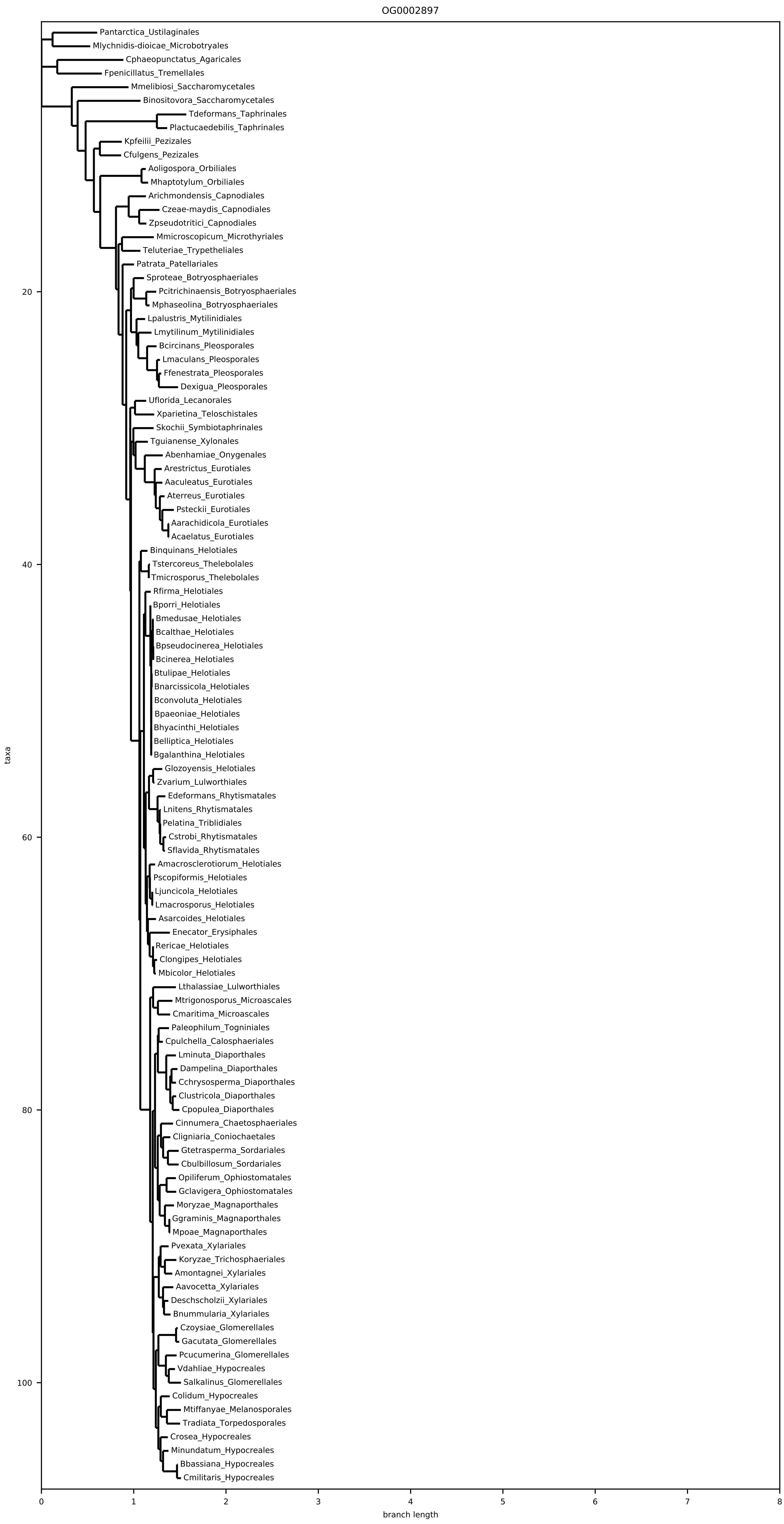

OG0002897
Pantarctica_Ustilaginales
Mlychnidis-dioicae_Microbotryales
Cphaeopunctatus_Agaricales
Fpenicillatus_Tremellales
Mmelibiosi_Saccharomycetales
Binositovora_Saccharomycetales
Tdeformans_Taphrinales
Plactucaedebilis_Taphrinales
Kpfeilii_Pezizales
Cfulgens_Pezizales
Aoligospora_Orbiliales
Mhaptotylum_Orbiliales
Arichmondensis_Capnodiales
Czeae-maydis_Capnodiales
Zpseudotritici_Capnodiales
Mmicroscopicum_Microthyriales
Teluteriae_Trypetheliales
Patrata_Patellariales
Sproteae_Botryosphaeriales
Pcitrichinaensis_Botryosphaeriales
Mphaseolina_Botryosphaeriales
Lpalustris_Mytilinidiales
Lmytilinum_Mytilinidiales
Bcircinans_Pleosporales
Lmaculans_Pleosporales
Ffenestrata_Pleosporales
Dexigua_Pleosporales
Uflorida_Lecanorales
Xparietina_Teloschistales
Skochii_Symbiotaphrinales
Tguianense_Xylonales
Abenhamiae_Onygenales
Arestrictus_Eurotiales
Aaculeatus_Eurotiales
Aterreus_Eurotiales
Psteckii_Eurotiales
Aarachidicola_Eurotiales
Acaelatus_Eurotiales
Binquinans_Helotiales
Tstercoreus_Thelebolales
Tmicrosporus_Thelebolales
Rfirma_Helotiales
Bporri_Helotiales
Bmedusae_Helotiales
Bcalthae_Helotiales
Bpseudocinerea_Helotiales
Bcinerea_Helotiales
Btulipae_Helotiales
Bnarcissicola_Helotiales
Bconvoluta_Helotiales
Bpaeoniae_Helotiales
Bhyacinthi_Helotiales
Belliptica_Helotiales
Bgalanthina_Helotiales
Glozoyensis_Helotiales
Zvarium_Lulworthiales
Edeformans_Rhytismatales
Lnitens_Rhytismatales
Pelatina_Triblidiales
Cstrobi_Rhytismatales
Sflavida_Rhytismatales
Amacrosclerotiorum_Helotiales
Pscopiformis_Helotiales
Ljuncicola_Helotiales
Lmacrosporus_Helotiales
Asarcoides_Helotiales
Enecator_Erysiphales
Rericae_Helotiales
Clongipes_Helotiales
Mbicolor_Helotiales
Lthalassiae_Lulworthiales
Mtrigonosporus_Microascales
Cmaritima_Microascales
Paleophilum_Togniniales
Cpulchella_Calosphaeriales
Lminuta_Diaporthales
Dampelina_Diaporthales
Cchrysosperma_Diaporthales
Clustricola_Diaporthales
Cpopulea_Diaporthales
Cinnumera_Chaetosphaeriales
Cligniaria_Coniochaetales
Gtetrasperma_Sordariales
Cbulbillosum_Sordariales
Opiliferum_Ophiostomatales
Gclavigera_Ophiostomatales
Moryzae_Magnaporthales
Ggraminis_Magnaporthales
Mpoae_Magnaporthales
Pvexata_Xylariales
Koryzae_Trichosphaeriales
Amontagnei_Xylariales
Aavocetta_Xylariales
Deschscholzii_Xylariales
Bnummularia_Xylariales
Czoysiae_Glomerellales
Gacutata_Glomerellales
Pcucumerina_Glomerellales
Vdahliae_Hypocreales
Salkalinus_Glomerellales
Colidum_Hypocreales
Mtiffanyae_Melanosporales
Tradiata_Torpedosporales
Crosea_Hypocreales
Minundatum_Hypocreales
Bbassiana_Hypocreales
Cmilitaris_Hypocreales
taxa
20
40
60
80
100
0
1
2
3
4
5
6
7
8
branch length

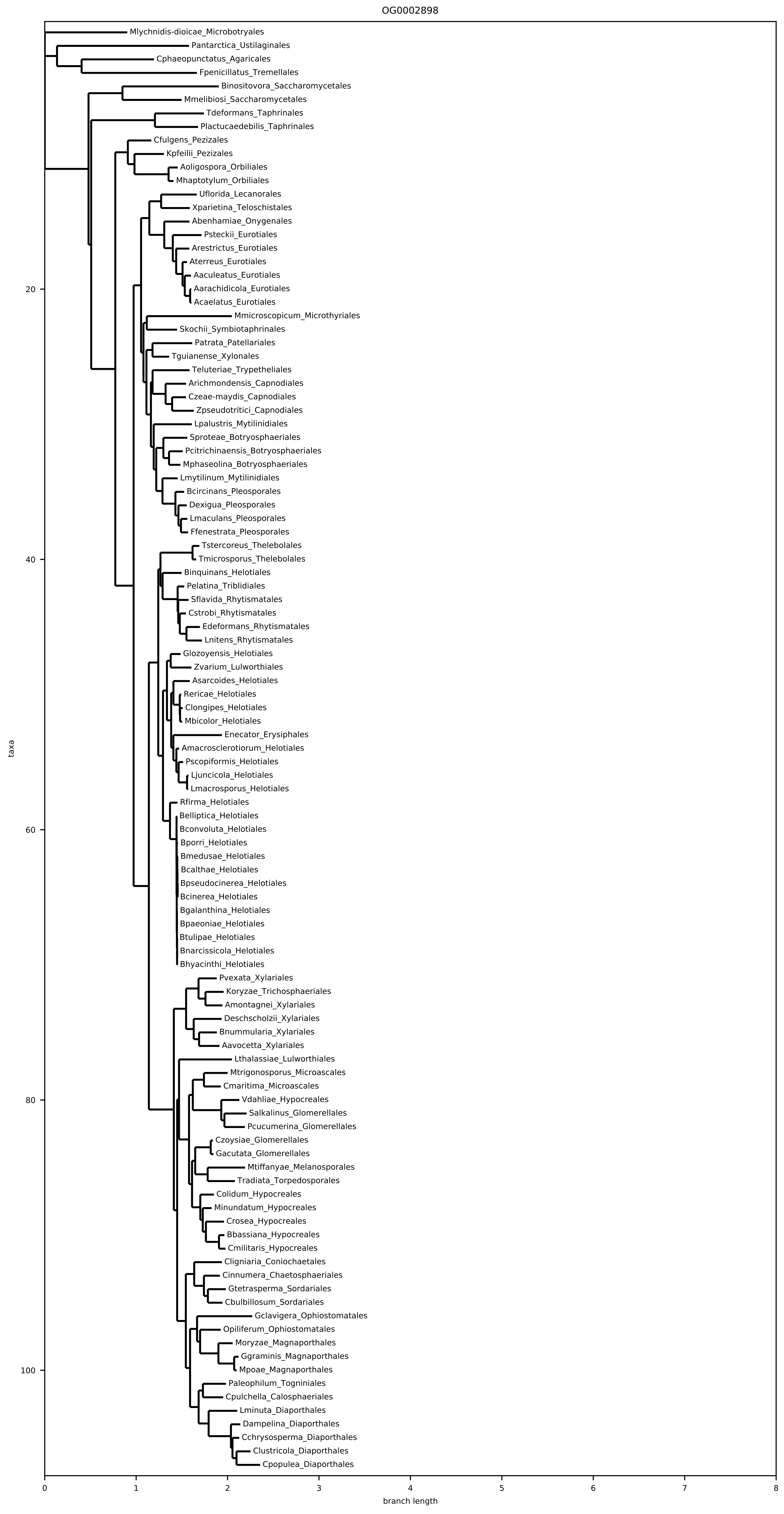

OG0002898
Mlychnidis-dioicae_Microbotryales
Pantarctica_Ustilaginales
Cphaeopunctatus_Agaricales
Fpenicillatus_Tremellales
Binositovora_Saccharomycetales
Mmelibiosi_Saccharomycetales
Tdeformans_Taphrinales
Plactucaedebilis_Taphrinales
Cfulgens_Pezizales
Kpfeilii_Pezizales
Aoligospora_Orbiliales
Mhaptotylum_Orbiliales
Uflorida_Lecanorales
Xparietina_Teloschistales
Abenhamiae_Onygenales
Psteckii_Eurotiales
Arestrictus_Eurotiales
Aterreus_Eurotiales
Aaculeatus_Eurotiales
Aarachidicola_Eurotiales
Acaelatus_Eurotiales
Mmicroscopicum_Microthyriales
Skochii_Symbiotaphrinales
Patrata_Patellariales
Tguianense_Xylonales
Teluteriae_Trypetheliales
Arichmondensis_Capnodiales
Czeae-maydis_Capnodiales
Zpseudotritici_Capnodiales
Lpalustris_Mytilinidiales
Sproteae_Botryosphaeriales
Pcitrichinaensis_Botryosphaeriales
Mphaseolina_Botryosphaeriales
Lmytilinum_Mytilinidiales
Bcircinans_Pleosporales
Dexigua_Pleosporales
Lmaculans_Pleosporales
Ffenestrata_Pleosporales
Tstercoreus_Thelebolales
Tmicrosporus_Thelebolales
Binquinans_Helotiales
Pelatina_Triblidiales
Sflavida_Rhytismatales
Cstrobi_Rhytismatales
Edeformans_Rhytismatales
Lnitens_Rhytismatales
Glozoyensis_Helotiales
Zvarium_Lulworthiales
Asarcoides_Helotiales
Rericae_Helotiales
Clongipes_Helotiales
Mbicolor_Helotiales
Enecator_Erysiphales
Amacrosclerotiorum_Helotiales
Pscopiformis_Helotiales
Ljuncicola_Helotiales
Lmacrosporus_Helotiales
Rfirma_Helotiales
Belliptica_Helotiales
Bconvoluta_Helotiales
Bporri_Helotiales
Bmedusae_Helotiales
Bcalthae_Helotiales
Bpseudocinerea_Helotiales
Bcinerea_Helotiales
Bgalanthina_Helotiales
Bpaeoniae_Helotiales
Btulipae_Helotiales
Bnarcissicola_Helotiales
Bhyacinthi_Helotiales
Pvexata_Xylariales
Koryzae_Trichosphaeriales
Amontagnei_Xylariales
Deschscholzii_Xylariales
Bnummularia_Xylariales
Aavocetta_Xylariales
Lthalassiae_Lulworthiales
Mtrigonosporus_Microascales
Cmaritima_Microascales
Vdahliae_Hypocreales
Salkalinus_Glomerellales
Pcucumerina_Glomerellales
Czoysiae_Glomerellales
Gacutata_Glomerellales
Mtiffanyae_Melanosporales
Tradiata_Torpedosporales
Colidum_Hypocreales
Minundatum_Hypocreales
Crosea_Hypocreales
Bbassiana_Hypocreales
Cmilitaris_Hypocreales
Cligniaria_Coniochaetales
Cinnumera_Chaetosphaeriales
Gtetrasperma_Sordariales
Cbulbillosum_Sordariales
Gclavigera_Ophiostomatales
Opiliferum_Ophiostomatales
Moryzae_Magnaporthales
Ggraminis_Magnaporthales
Mpoae_Magnaporthales
Paleophilum_Togniniales
Cpulchella_Calosphaeriales
Lminuta_Diaporthales
Dampelina_Diaporthales
Cchrysosperma_Diaporthales
Clustricola_Diaporthales
Cpopulea_Diaporthales
taxa
20
40
60
80
100
0
1
2
3
4
5
6
7
8
branch length

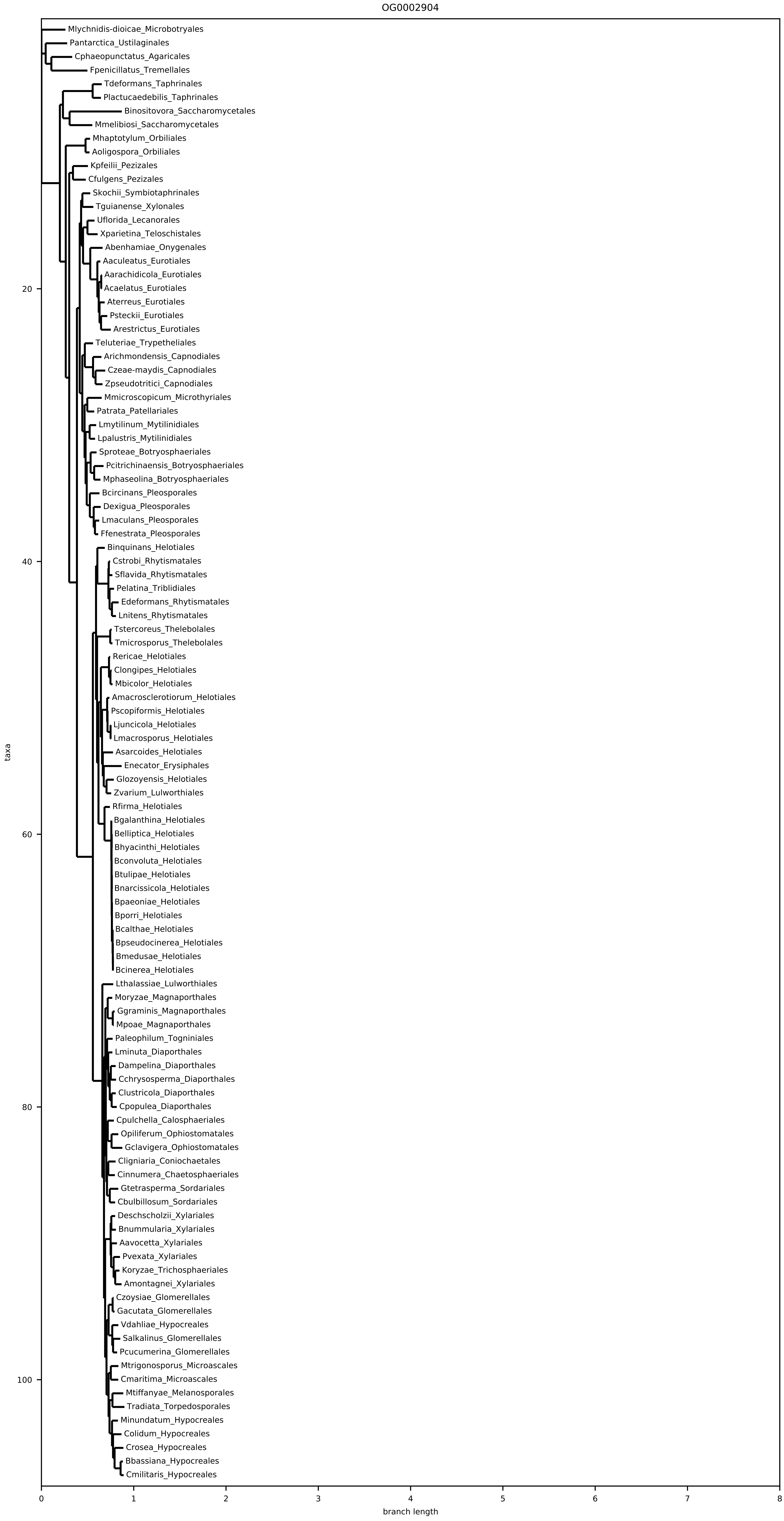
OG0002904
Mlychnidis-dioicae_Microbotryales
Pantarctica_Ustilaginales
Cphaeopunctatus_Agaricales
Fpenicillatus_Tremellales
Tdeformans_Taphrinales
Plactucaedebilis_Taphrinales
Binositovora_Saccharomycetales
Mmelibiosi_Saccharomycetales
Mhaptotylum_Orbiliales
Aoligospora_Orbiliales
Kpfeilii_Pezizales
Cfulgens_Pezizales
Skochii_Symbiotaphrinales
Tguianense_Xylonales
Uflorida_Lecanorales
Xparietina_Teloschistales
Abenhamiae_Onygenales
Aaculeatus_Eurotiales
Aarachidicola_Eurotiales
Acaelatus_Eurotiales
Aterreus_Eurotiales
Psteckii_Eurotiales
Arestrictus_Eurotiales
Teluteriae_Trypetheliales
Arichmondensis_Capnodiales
Czeae-maydis_Capnodiales
Zpseudotritici_Capnodiales
Mmicroscopicum_Microthyriales
Patrata_Patellariales
Lmytilinum_Mytilinidiales
Lpalustris_Mytilinidiales
Sproteae_Botryosphaeriales
Pcitrichinaensis_Botryosphaeriales
Mphaseolina_Botryosphaeriales
Bcircinans_Pleosporales
Dexigua_Pleosporales
Lmaculans_Pleosporales
Ffenestrata_Pleosporales
Binquinans_Helotiales
Cstrobi_Rhytismatales
Sflavida_Rhytismatales
Pelatina_Triblidiales
Edeformans_Rhytismatales
Lnitens_Rhytismatales
Tstercoreus_Thelebolales
Tmicrosporus_Thelebolales
Rericae_Helotiales
Clongipes_Helotiales
Mbicolor_Helotiales
Amacrosclerotiorum_Helotiales
Pscopiformis_Helotiales
Ljuncicola_Helotiales
Lmacrosporus_Helotiales
Asarcoides_Helotiales
Enecator_Erysiphales
Glozoyensis_Helotiales
Zvarium_Lulworthiales
Rfirma_Helotiales
Bgalanthina_Helotiales
Belliptica_Helotiales
Bhyacinthi_Helotiales
Bconvoluta_Helotiales
Btulipae_Helotiales
Bnarcissicola_Helotiales
Bpaeoniae_Helotiales
Bporri_Helotiales
Bcalthae_Helotiales
Bpseudocinerea_Helotiales
Bmedusae_Helotiales
Bcinerea_Helotiales
Lthalassiae_Lulworthiales
Moryzae_Magnaporthales
Ggraminis_Magnaporthales
Mpoae_Magnaporthales
Paleophilum_Togniniales
Lminuta_Diaporthales
Dampelina_Diaporthales
Cchrysosperma_Diaporthales
Clustricola_Diaporthales
Cpopulea_Diaporthales
Cpulchella_Calosphaeriales
Opiliferum_Ophiostomatales
Gclavigera_Ophiostomatales
Cligniaria_Coniochaetales
Cinnumera_Chaetosphaeriales
Gtetrasperma_Sordariales
Cbulbillosum_Sordariales
Deschscholzii_Xylariales
Bnummularia_Xylariales
Aavocetta_Xylariales
Pvexata_Xylariales
Koryzae_Trichosphaeriales
Amontagnei_Xylariales
Czoysiae_Glomerellales
Gacutata_Glomerellales
Vdahliae_Hypocreales
Salkalinus_Glomerellales
Pcucumerina_Glomerellales
Mtrigonosporus_Microascales
Cmaritima_Microascales
Mtiffanyae_Melanosporales
Tradiata_Torpedosporales
Minundatum_Hypocreales
Colidum_Hypocreales
Crosea_Hypocreales
Bbassiana_Hypocreales
Cmilitaris_Hypocreales
taxa
20
40
60
80
100
0
1
2
3
4
5
6
7
8
branch length

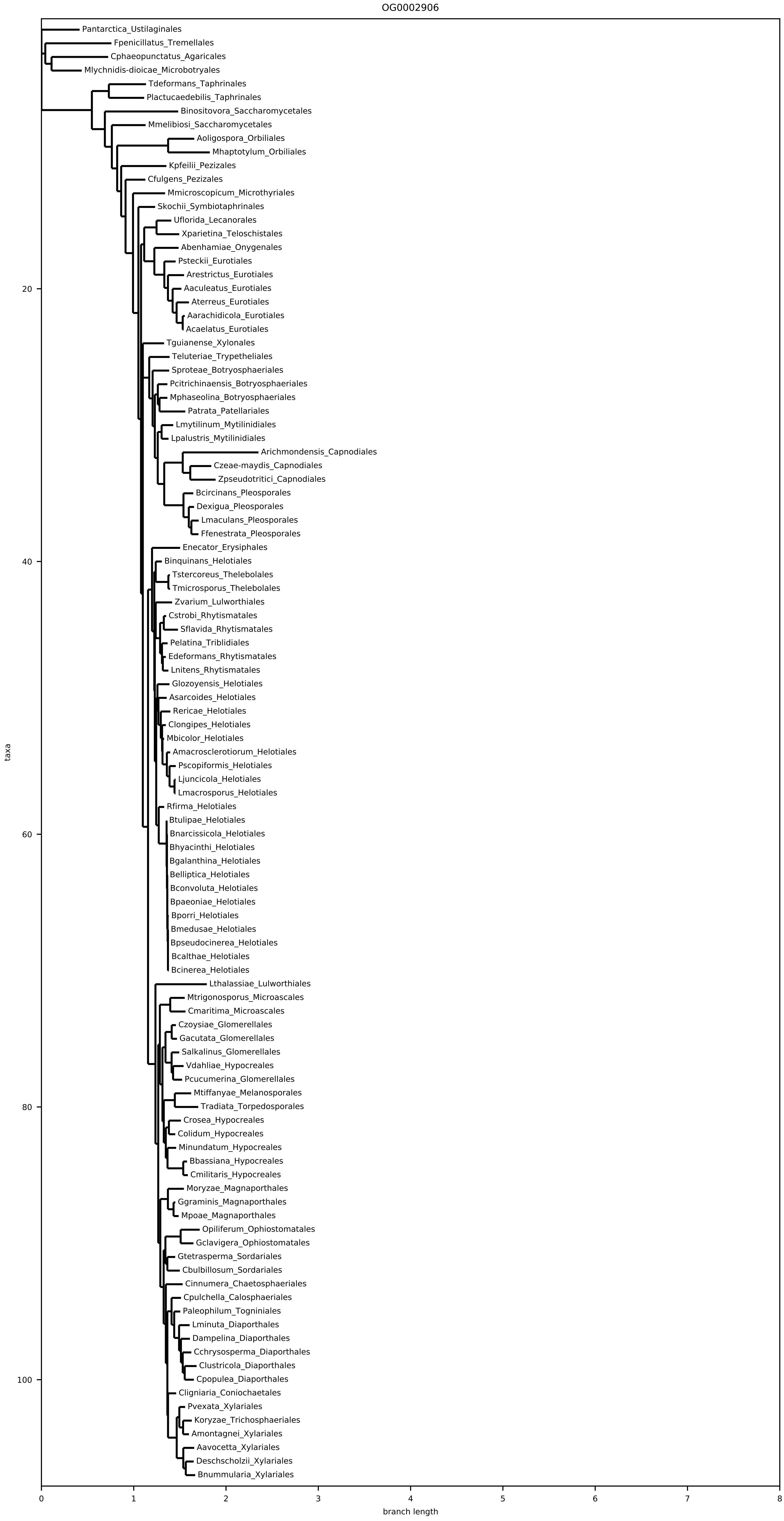

OG0002906
Pantarctica_Ustilaginales
Fpenicillatus_Tremellales
Cphaeopunctatus_Agaricales
Mlychnidis-dioicae_Microbotryales
Tdeformans_Taphrinales
Plactucaedebilis_Taphrinales
Binositovora_Saccharomycetales
Mmelibiosi_Saccharomycetales
Aoligospora_Orbiliales
Mhaptotylum_Orbiliales
Kpfeilii_Pezizales
Cfulgens_Pezizales
Mmicroscopicum_Microthyriales
Skochii_Symbiotaphrinales
Uflorida_Lecanorales
Xparietina_Teloschistales
Abenhamiae_Onygenales
Psteckii_Eurotiales
Arestrictus_Eurotiales
Aaculeatus_Eurotiales
Aterreus_Eurotiales
Aarachidicola_Eurotiales
Acaelatus_Eurotiales
Tguianense_Xylonales
Teluteriae_Trypetheliales
Sproteae_Botryosphaeriales
Pcitrichinaensis_Botryosphaeriales
Mphaseolina_Botryosphaeriales
Patrata_Patellariales
Lmytilinum_Mytilinidiales
Lpalustris_Mytilinidiales
Arichmondensis_Capnodiales
Czeae-maydis_Capnodiales
Zpseudotritici_Capnodiales
Bcircinans_Pleosporales
Dexigua_Pleosporales
Lmaculans_Pleosporales
Ffenestrata_Pleosporales
Enecator_Erysiphales
Binquinans_Helotiales
Tstercoreus_Thelebolales
Tmicrosporus_Thelebolales
Zvarium_Lulworthiales
Cstrobi_Rhytismatales
Sflavida_Rhytismatales
Pelatina_Triblidiales
Edeformans_Rhytismatales
Lnitens_Rhytismatales
Glozoyensis_Helotiales
Asarcoides_Helotiales
Rericae_Helotiales
Clongipes_Helotiales
Mbicolor_Helotiales
Amacrosclerotiorum_Helotiales
Pscopiformis_Helotiales
Ljuncicola_Helotiales
Lmacrosporus_Helotiales
Rfirma_Helotiales
Btulipae_Helotiales
Bnarcissicola_Helotiales
Bhyacinthi_Helotiales
Bgalanthina_Helotiales
Belliptica_Helotiales
Bconvoluta_Helotiales
Bpaeoniae_Helotiales
Bporri_Helotiales
Bmedusae_Helotiales
Bpseudocinerea_Helotiales
Bcalthae_Helotiales
Bcinerea_Helotiales
Lthalassiae_Lulworthiales
Mtrigonosporus_Microascales
Cmaritima_Microascales
Czoysiae_Glomerellales
Gacutata_Glomerellales
Salkalinus_Glomerellales
Vdahliae_Hypocreales
Pcucumerina_Glomerellales
Mtiffanyae_Melanosporales
Tradiata_Torpedosporales
Crosea_Hypocreales
Colidum_Hypocreales
Minundatum_Hypocreales
Bbassiana_Hypocreales
Cmilitaris_Hypocreales
Moryzae_Magnaporthales
Ggraminis_Magnaporthales
Mpoae_Magnaporthales
Opiliferum_Ophiostomatales
Gclavigera_Ophiostomatales
Gtetrasperma_Sordariales
Cbulbillosum_Sordariales
Cinnumera_Chaetosphaeriales
Cpulchella_Calosphaeriales
Paleophilum_Togniniales
Lminuta_Diaporthales
Dampelina_Diaporthales
Cchrysosperma_Diaporthales
Clustricola_Diaporthales
Cpopulea_Diaporthales
Cligniaria_Coniochaetales
Pvexata_Xylariales
Koryzae_Trichosphaeriales
Amontagnei_Xylariales
Aavocetta_Xylariales
Deschscholzii_Xylariales
Bnummularia_Xylariales
taxa
20
40
60
80
100
0
1
2
3
4
5
6
7
8
branch length

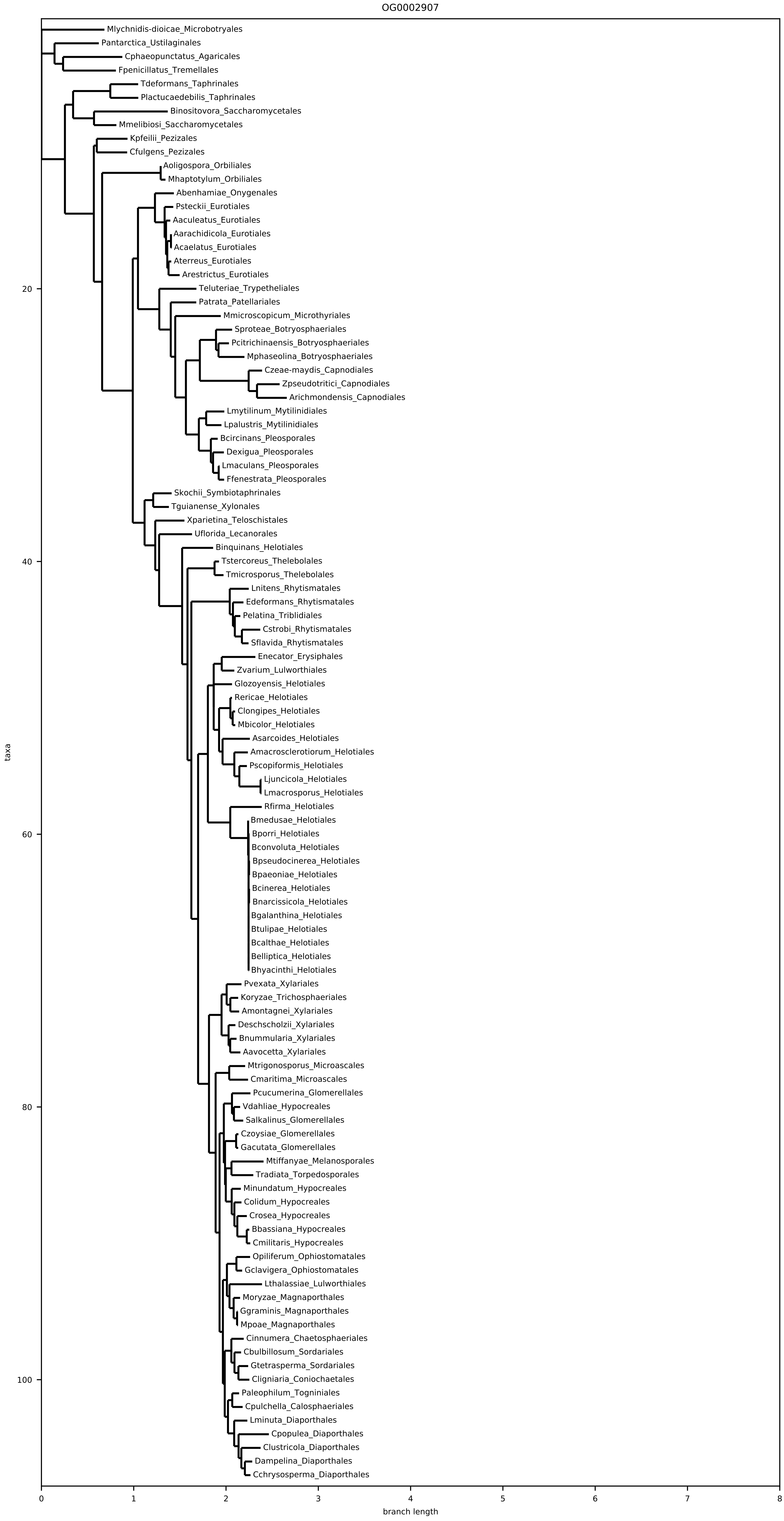

OG0002907
Mlychnidis-dioicae_Microbotryales
Pantarctica_Ustilaginales
Cphaeopunctatus_Agaricales
Fpenicillatus_Tremellales
Tdeformans_Taphrinales
Plactucaedebilis_Taphrinales
Binositovora_Saccharomycetales
Mmelibiosi_Saccharomycetales
Kpfeilii_Pezizales
Cfulgens_Pezizales
Aoligospora_Orbiliales
Mhaptotylum_Orbiliales
Abenhamiae_Onygenales
Psteckii_Eurotiales
Aaculeatus_Eurotiales
Aarachidicola_Eurotiales
Acaelatus_Eurotiales
Aterreus_Eurotiales
Arestrictus_Eurotiales
Teluteriae_Trypetheliales
Patrata_Patellariales
Mmicroscopicum_Microthyriales
Sproteae_Botryosphaeriales
Pcitrichinaensis_Botryosphaeriales
Mphaseolina_Botryosphaeriales
Czeae-maydis_Capnodiales
Zpseudotritici_Capnodiales
Arichmondensis_Capnodiales
Lmytilinum_Mytilinidiales
Lpalustris_Mytilinidiales
Bcircinans_Pleosporales
Dexigua_Pleosporales
Lmaculans_Pleosporales
Ffenestrata_Pleosporales
Skochii_Symbiotaphrinales
Tguianense_Xylonales
Xparietina_Teloschistales
Uflorida_Lecanorales
Binquinans_Helotiales
Tstercoreus_Thelebolales
Tmicrosporus_Thelebolales
Lnitens_Rhytismatales
Edeformans_Rhytismatales
Pelatina_Triblidiales
Cstrobi_Rhytismatales
Sflavida_Rhytismatales
Enecator_Erysiphales
Zvarium_Lulworthiales
Glozoyensis_Helotiales
Rericae_Helotiales
Clongipes_Helotiales
Mbicolor_Helotiales
Asarcoides_Helotiales
Amacrosclerotiorum_Helotiales
Pscopiformis_Helotiales
Ljuncicola_Helotiales
Lmacrosporus_Helotiales
Rfirma_Helotiales
Bmedusae_Helotiales
Bporri_Helotiales
Bconvoluta_Helotiales
Bpseudocinerea_Helotiales
Bpaeoniae_Helotiales
Bcinerea_Helotiales
Bnarcissicola_Helotiales
Bgalanthina_Helotiales
Btulipae_Helotiales
Bcalthae_Helotiales
Belliptica_Helotiales
Bhyacinthi_Helotiales
Pvexata_Xylariales
Koryzae_Trichosphaeriales
Amontagnei_Xylariales
Deschscholzii_Xylariales
Bnummularia_Xylariales
Aavocetta_Xylariales
Mtrigonosporus_Microascales
Cmaritima_Microascales
Pcucumerina_Glomerellales
Vdahliae_Hypocreales
Salkalinus_Glomerellales
Czoysiae_Glomerellales
Gacutata_Glomerellales
Mtiffanyae_Melanosporales
Tradiata_Torpedosporales
Minundatum_Hypocreales
Colidum_Hypocreales
Crosea_Hypocreales
Bbassiana_Hypocreales
Cmilitaris_Hypocreales
Opiliferum_Ophiostomatales
Gclavigera_Ophiostomatales
Lthalassiae_Lulworthiales
Moryzae_Magnaporthales
Ggraminis_Magnaporthales
Mpoae_Magnaporthales
Cinnumera_Chaetosphaeriales
Cbulbillosum_Sordariales
Gtetrasperma_Sordariales
Cligniaria_Coniochaetales
Paleophilum_Togniniales
Cpulchella_Calosphaeriales
Lminuta_Diaporthales
Cpopulea_Diaporthales
Clustricola_Diaporthales
Dampelina_Diaporthales
Cchrysosperma_Diaporthales
taxa
20
40
60
80
100
0
1
2
3
4
5
6
7
8
branch length

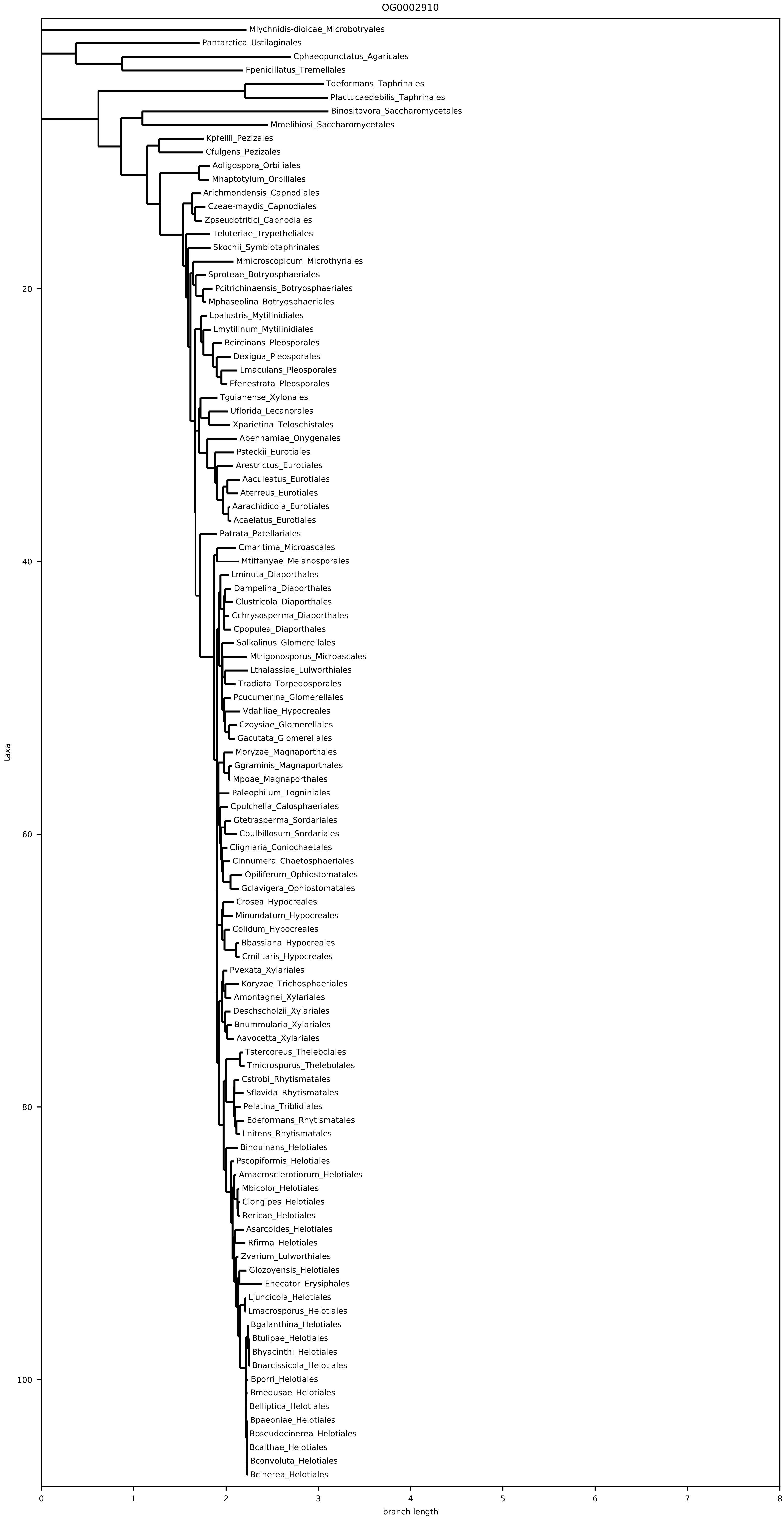

OG0002910
Mlychnidis-dioicae_Microbotryales
Pantarctica_Ustilaginales
Cphaeopunctatus_Agaricales
Fpenicillatus_Tremellales
Tdeformans_Taphrinales
Plactucaedebilis_Taphrinales
Binositovora_Saccharomycetales
Mmelibiosi_Saccharomycetales
Kpfeilii_Pezizales
Cfulgens_Pezizales
Aoligospora_Orbiliales
Mhaptotylum_Orbiliales
Arichmondensis_Capnodiales
Czeae-maydis_Capnodiales
Zpseudotritici_Capnodiales
Teluteriae_Trypetheliales
Skochii_Symbiotaphrinales
Mmicroscopicum_Microthyriales
Sproteae_Botryosphaeriales
Pcitrichinaensis_Botryosphaeriales
Mphaseolina_Botryosphaeriales
Lpalustris_Mytilinidiales
Lmytilinum_Mytilinidiales
Bcircinans_Pleosporales
Dexigua_Pleosporales
Lmaculans_Pleosporales
Ffenestrata_Pleosporales
Tguianense_Xylonales
Uflorida_Lecanorales
Xparietina_Teloschistales
Abenhamiae_Onygenales
Psteckii_Eurotiales
Arestrictus_Eurotiales
Aaculeatus_Eurotiales
Aterreus_Eurotiales
Aarachidicola_Eurotiales
Acaelatus_Eurotiales
Patrata_Patellariales
Cmaritima_Microascales
Mtiffanyae_Melanosporales
Lminuta_Diaporthales
Dampelina_Diaporthales
Clustricola_Diaporthales
Cchrysosperma_Diaporthales
Cpopulea_Diaporthales
Salkalinus_Glomerellales
Mtrigonosporus_Microascales
Lthalassiae_Lulworthiales
Tradiata_Torpedosporales
Pcucumerina_Glomerellales
Vdahliae_Hypocreales
Czoysiae_Glomerellales
Gacutata_Glomerellales
Moryzae_Magnaporthales
Ggraminis_Magnaporthales
Mpoae_Magnaporthales
Paleophilum_Togniniales
Cpulchella_Calosphaeriales
Gtetrasperma_Sordariales
Cbulbillosum_Sordariales
Cligniaria_Coniochaetales
Cinnumera_Chaetosphaeriales
Opiliferum_Ophiostomatales
Gclavigera_Ophiostomatales
Crosea_Hypocreales
Minundatum_Hypocreales
Colidum_Hypocreales
Bbassiana_Hypocreales
Cmilitaris_Hypocreales
Pvexata_Xylariales
Koryzae_Trichosphaeriales
Amontagnei_Xylariales
Deschscholzii_Xylariales
Bnummularia_Xylariales
Aavocetta_Xylariales
Tstercoreus_Thelebolales
Tmicrosporus_Thelebolales
Cstrobi_Rhytismatales
Sflavida_Rhytismatales
Pelatina_Triblidiales
Edeformans_Rhytismatales
Lnitens_Rhytismatales
Binquinans_Helotiales
Pscopiformis_Helotiales
Amacrosclerotiorum_Helotiales
Mbicolor_Helotiales
Clongipes_Helotiales
Rericae_Helotiales
Asarcoides_Helotiales
Rfirma_Helotiales
Zvarium_Lulworthiales
Glozoyensis_Helotiales
Enecator_Erysiphales
Ljuncicola_Helotiales
Lmacrosporus_Helotiales
Bgalanthina_Helotiales
Btulipae_Helotiales
Bhyacinthi_Helotiales
Bnarcissicola_Helotiales
Bporri_Helotiales
Bmedusae_Helotiales
Belliptica_Helotiales
Bpaeoniae_Helotiales
Bpseudocinerea_Helotiales
Bcalthae_Helotiales
Bconvoluta_Helotiales
Bcinerea_Helotiales
taxa
20
40
60
80
100
0
1
2
3
4
5
6
7
8
branch length

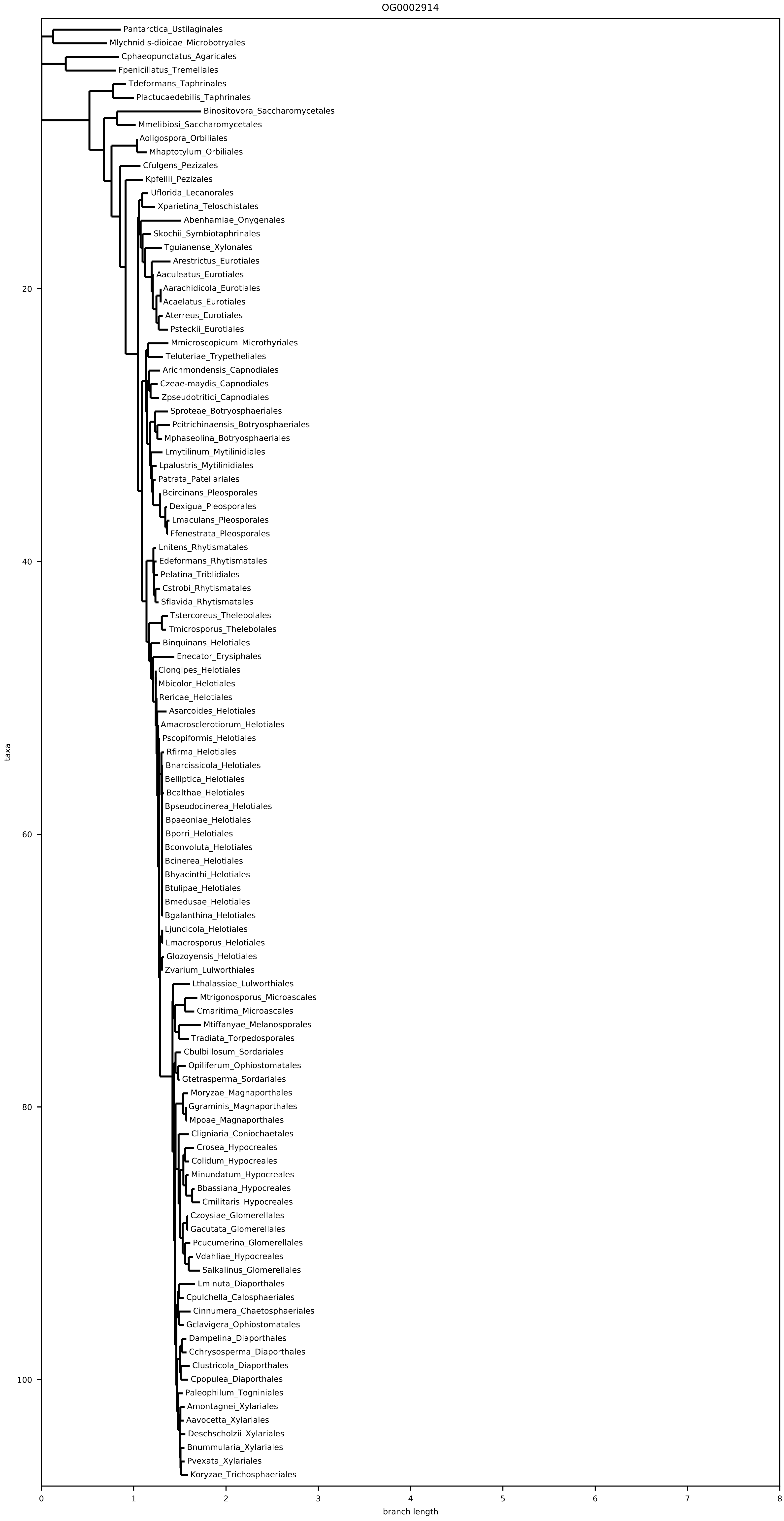
OG0002914
Pantarctica_Ustilaginales
Mlychnidis-dioicae_Microbotryales
Cphaeopunctatus_Agaricales
Fpenicillatus_Tremellales
Tdeformans_Taphrinales
Plactucaedebilis_Taphrinales
Binositovora_Saccharomycetales
Mmelibiosi_Saccharomycetales
Aoligospora_Orbiliales
Mhaptotylum_Orbiliales
Cfulgens_Pezizales
Kpfeilii_Pezizales
Uflorida_Lecanorales
Xparietina_Teloschistales
Abenhamiae_Onygenales
Skochii_Symbiotaphrinales
Tguianense_Xylonales
Arestrictus_Eurotiales
Aaculeatus_Eurotiales
Aarachidicola_Eurotiales
Acaelatus_Eurotiales
Aterreus_Eurotiales
Psteckii_Eurotiales
Mmicroscopicum_Microthyriales
Teluteriae_Trypetheliales
Arichmondensis_Capnodiales
Czeae-maydis_Capnodiales
Zpseudotritici_Capnodiales
Sproteae_Botryosphaeriales
Pcitrichinaensis_Botryosphaeriales
Mphaseolina_Botryosphaeriales
Lmytilinum_Mytilinidiales
Lpalustris_Mytilinidiales
Patrata_Patellariales
Bcircinans_Pleosporales
Dexigua_Pleosporales
Lmaculans_Pleosporales
Ffenestrata_Pleosporales
Lnitens_Rhytismatales
Edeformans_Rhytismatales
Pelatina_Triblidiales
Cstrobi_Rhytismatales
Sflavida_Rhytismatales
Tstercoreus_Thelebolales
Tmicrosporus_Thelebolales
Binquinans_Helotiales
Enecator_Erysiphales
Clongipes_Helotiales
Mbicolor_Helotiales
Rericae_Helotiales
Asarcoides_Helotiales
Amacrosclerotiorum_Helotiales
Pscopiformis_Helotiales
Rfirma_Helotiales
Bnarcissicola_Helotiales
Belliptica_Helotiales
Bcalthae_Helotiales
Bpseudocinerea_Helotiales
Bpaeoniae_Helotiales
Bporri_Helotiales
Bconvoluta_Helotiales
Bcinerea_Helotiales
Bhyacinthi_Helotiales
Btulipae_Helotiales
Bmedusae_Helotiales
Bgalanthina_Helotiales
Ljuncicola_Helotiales
Lmacrosporus_Helotiales
Glozoyensis_Helotiales
Zvarium_Lulworthiales
Lthalassiae_Lulworthiales
Mtrigonosporus_Microascales
Cmaritima_Microascales
Mtiffanyae_Melanosporales
Tradiata_Torpedosporales
Cbulbillosum_Sordariales
Opiliferum_Ophiostomatales
Gtetrasperma_Sordariales
Moryzae_Magnaporthales
Ggraminis_Magnaporthales
Mpoae_Magnaporthales
Cligniaria_Coniochaetales
Crosea_Hypocreales
Colidum_Hypocreales
Minundatum_Hypocreales
Bbassiana_Hypocreales
Cmilitaris_Hypocreales
Czoysiae_Glomerellales
Gacutata_Glomerellales
Pcucumerina_Glomerellales
Vdahliae_Hypocreales
Salkalinus_Glomerellales
Lminuta_Diaporthales
Cpulchella_Calosphaeriales
Cinnumera_Chaetosphaeriales
Gclavigera_Ophiostomatales
Dampelina_Diaporthales
Cchrysosperma_Diaporthales
Clustricola_Diaporthales
Cpopulea_Diaporthales
Paleophilum_Togniniales
Amontagnei_Xylariales
Aavocetta_Xylariales
Deschscholzii_Xylariales
Bnummularia_Xylariales
Pvexata_Xylariales
Koryzae_Trichosphaeriales
taxa
20
40
60
80
100
0
1
2
3
4
5
6
7
8
branch length

OG0002919

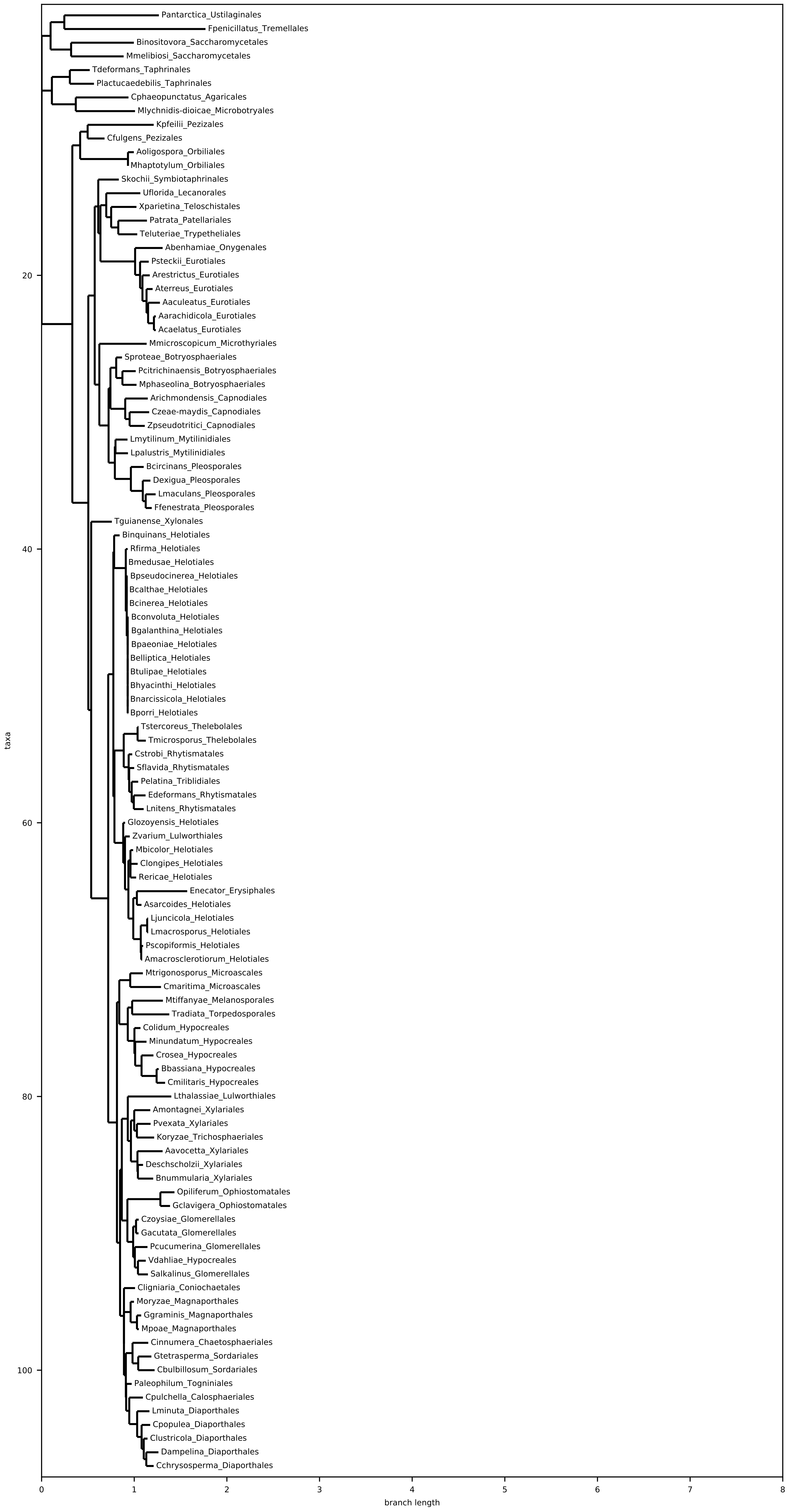

Pantarctica_Ustilaginales
Fpenicillatus_Tremellales
Binositovora_Saccharomycetales
Mmelibiosi_Saccharomycetales
Tdeformans_Taphrinales
Plactucaedebilis_Taphrinales
Cphaeopunctatus_Agaricales
Mlychnidis-dioicae_Microbotryales
Kpfeilii_Pezizales
Cfulgens_Pezizales
Aoligospora_Orbiliales
Mhaptotylum_Orbiliales
Skochii_Symbiotaphrinales
Uflorida_Lecanorales
Xparietina_Teloschistales
Patrata_Patellariales
Teluteriae_Trypetheliales
Abenhamiae_Onygenales
Psteckii_Eurotiales
Arestrictus_Eurotiales
Aterreus_Eurotiales
Aaculeatus_Eurotiales
Aarachidicola_Eurotiales
Acaelatus_Eurotiales
Mmicroscopicum_Microthyriales
Sproteae_Botryosphaeriales
Pcitrichinaensis_Botryosphaeriales
Mphaseolina_Botryosphaeriales
Arichmondensis_Capnodiales
Czeae-maydis_Capnodiales
Zpseudotritici_Capnodiales
Lmytilinum_Mytilinidiales
Lpalustris_Mytilinidiales
Bcircinans_Pleosporales
Dexigua_Pleosporales
Lmaculans_Pleosporales
Ffenestrata_Pleosporales
Tguianense_Xylonales
Binquinans_Helotiales
Rfirma_Helotiales
Bmedusae_Helotiales
Bpseudocinerea_Helotiales
Bcalthae_Helotiales
Bcinerea_Helotiales
Bconvoluta_Helotiales
Bgalanthina_Helotiales
Bpaeoniae_Helotiales
Belliptica_Helotiales
Btulipae_Helotiales
Bhyacinthi_Helotiales
Bnarcissicola_Helotiales
Bporri_Helotiales
Tstercoreus_Thelebolales
Tmicrosporus_Thelebolales
Cstrobi_Rhytismatales
Sflavida_Rhytismatales
Pelatina_Triblidiales
Edeformans_Rhytismatales
Lnitens_Rhytismatales
Glozoyensis_Helotiales
Zvarium_Lulworthiales
Mbicolor_Helotiales
Clongipes_Helotiales
Rericae_Helotiales
Enecator_Erysiphales
Asarcoides_Helotiales
Ljuncicola_Helotiales
Lmacrosporus_Helotiales
Pscopiformis_Helotiales
Amacrosclerotiorum_Helotiales
Mtrigonosporus_Microascales
Cmaritima_Microascales
Mtiffanyae_Melanosporales
Tradiata_Torpedosporales
Colidum_Hypocreales
Minundatum_Hypocreales
Crosea_Hypocreales
Bbassiana_Hypocreales
Cmilitaris_Hypocreales
Lthalassiae_Lulworthiales
Amontagnei_Xylariales
Pvexata_Xylariales
Koryzae_Trichosphaeriales
Aavocetta_Xylariales
Deschscholzii_Xylariales
Bnummularia_Xylariales
Opiliferum_Ophiostomatales
Gclavigera_Ophiostomatales
Czoysiae_Glomerellales
Gacutata_Glomerellales
Pcucumerina_Glomerellales
Vdahliae_Hypocreales
Salkalinus_Glomerellales
Cligniaria_Coniochaetales
Moryzae_Magnaporthales
Ggraminis_Magnaporthales
Mpoae_Magnaporthales
Cinnumera_Chaetosphaeriales
Gtetrasperma_Sordariales
Cbulbillosum_Sordariales
Paleophilum_Togniniales
Cpulchella_Calosphaeriales
Lminuta_Diaporthales
Cpopulea_Diaporthales
Clustricola_Diaporthales
Dampelina_Diaporthales
Cchrysosperma_Diaporthales
taxa
20
40
60
80
100
0
1
2
3
4
5
6
7
8
branch length

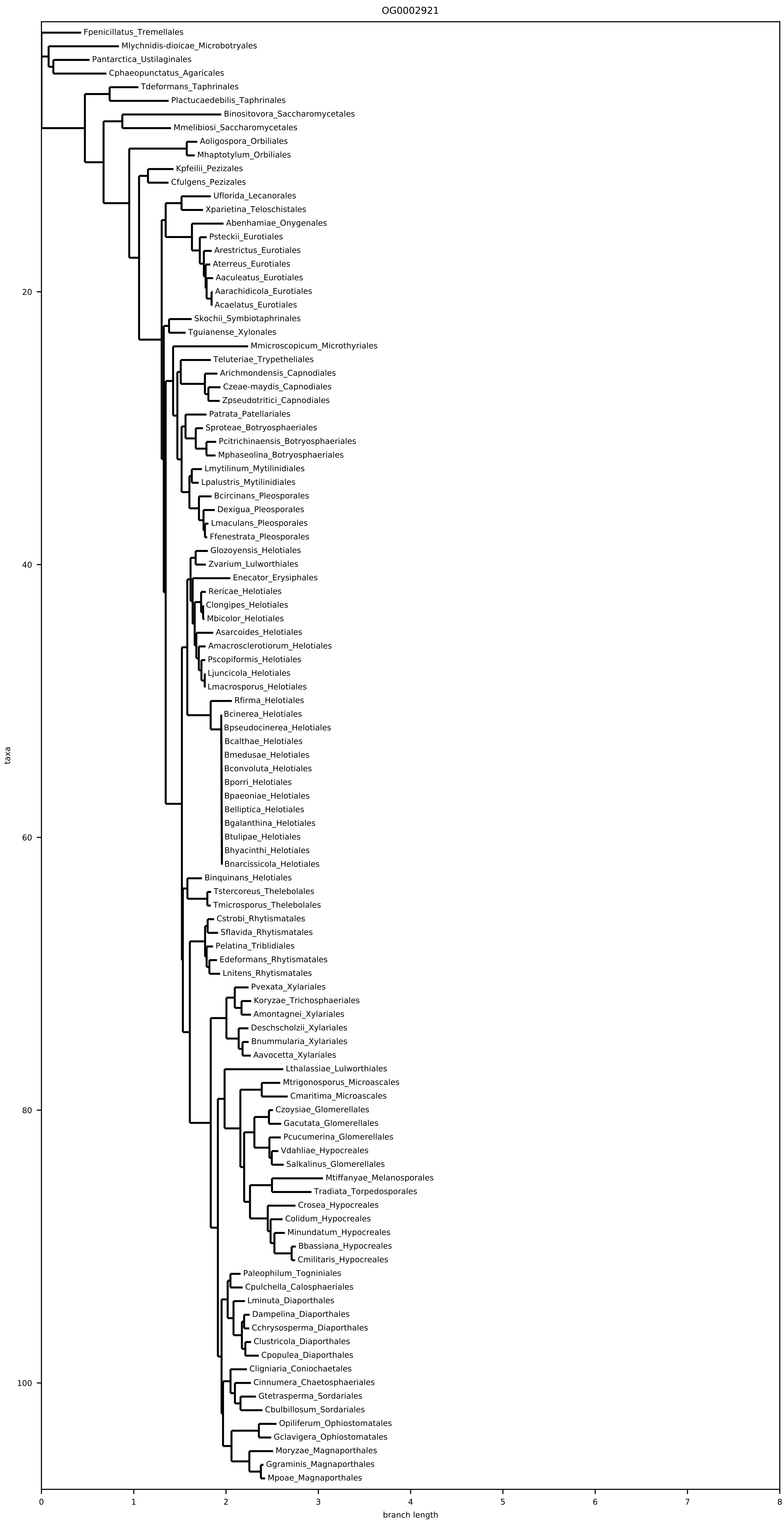
OG0002921
Fpenicillatus_Tremellales
Mlychnidis-dioicae_Microbotryales
Pantarctica_Ustilaginales
Cphaeopunctatus_Agaricales
Tdeformans_Taphrinales
Plactucaedebilis_Taphrinales
Binositovora_Saccharomycetales
Mmelibiosi_Saccharomycetales
Aoligospora_Orbiliales
Mhaptotylum_Orbiliales
Kpfeilii_Pezizales
Cfulgens_Pezizales
Uflorida_Lecanorales
Xparietina_Teloschistales
Abenhamiae_Onygenales
Psteckii_Eurotiales
Arestrictus_Eurotiales
Aterreus_Eurotiales
Aaculeatus_Eurotiales
Aarachidicola_Eurotiales
Acaelatus_Eurotiales
Skochii_Symbiotaphrinales
Tguianense_Xylonales
Mmicroscopicum_Microthyriales
Teluteriae_Trypetheliales
Arichmondensis_Capnodiales
Czeae-maydis_Capnodiales
Zpseudotritici_Capnodiales
Patrata_Patellariales
Sproteae_Botryosphaeriales
Pcitrichinaensis_Botryosphaeriales
Mphaseolina_Botryosphaeriales
Lmytilinum_Mytilinidiales
Lpalustris_Mytilinidiales
Bcircinans_Pleosporales
Dexigua_Pleosporales
Lmaculans_Pleosporales
Ffenestrata_Pleosporales
Glozoyensis_Helotiales
Zvarium_Lulworthiales
Enecator_Erysiphales
Rericae_Helotiales
Clongipes_Helotiales
Mbicolor_Helotiales
Asarcoides_Helotiales
Amacrosclerotiorum_Helotiales
Pscopiformis_Helotiales
Ljuncicola_Helotiales
Lmacrosporus_Helotiales
Rfirma_Helotiales
Bcinerea_Helotiales
Bpseudocinerea_Helotiales
Bcalthae_Helotiales
Bmedusae_Helotiales
Bconvoluta_Helotiales
Bporri_Helotiales
Bpaeoniae_Helotiales
Belliptica_Helotiales
Bgalanthina_Helotiales
Btulipae_Helotiales
Bhyacinthi_Helotiales
Bnarcissicola_Helotiales
Binquinans_Helotiales
Tstercoreus_Thelebolales
Tmicrosporus_Thelebolales
Cstrobi_Rhytismatales
Sflavida_Rhytismatales
Pelatina_Triblidiales
Edeformans_Rhytismatales
Lnitens_Rhytismatales
Pvexata_Xylariales
Koryzae_Trichosphaeriales
Amontagnei_Xylariales
Deschscholzii_Xylariales
Bnummularia_Xylariales
Aavocetta_Xylariales
Lthalassiae_Lulworthiales
Mtrigonosporus_Microascales
Cmaritima_Microascales
Czoysiae_Glomerellales
Gacutata_Glomerellales
Pcucumerina_Glomerellales
Vdahliae_Hypocreales
Salkalinus_Glomerellales
Mtiffanyae_Melanosporales
Tradiata_Torpedosporales
Crosea_Hypocreales
Colidum_Hypocreales
Minundatum_Hypocreales
Bbassiana_Hypocreales
Cmilitaris_Hypocreales
Paleophilum_Togniniales
Cpulchella_Calosphaeriales
Lminuta_Diaporthales
Dampelina_Diaporthales
Cchrysosperma_Diaporthales
Clustricola_Diaporthales
Cpopulea_Diaporthales
Cligniaria_Coniochaetales
Cinnumera_Chaetosphaeriales
Gtetrasperma_Sordariales
Cbulbillosum_Sordariales
Opiliferum_Ophiostomatales
Gclavigera_Ophiostomatales
Moryzae_Magnaporthales
Ggraminis_Magnaporthales
Mpoae_Magnaporthales
taxa
20
40
60
80
100
0
1
2
3
4
5
6
7
8
branch length

OG0002924

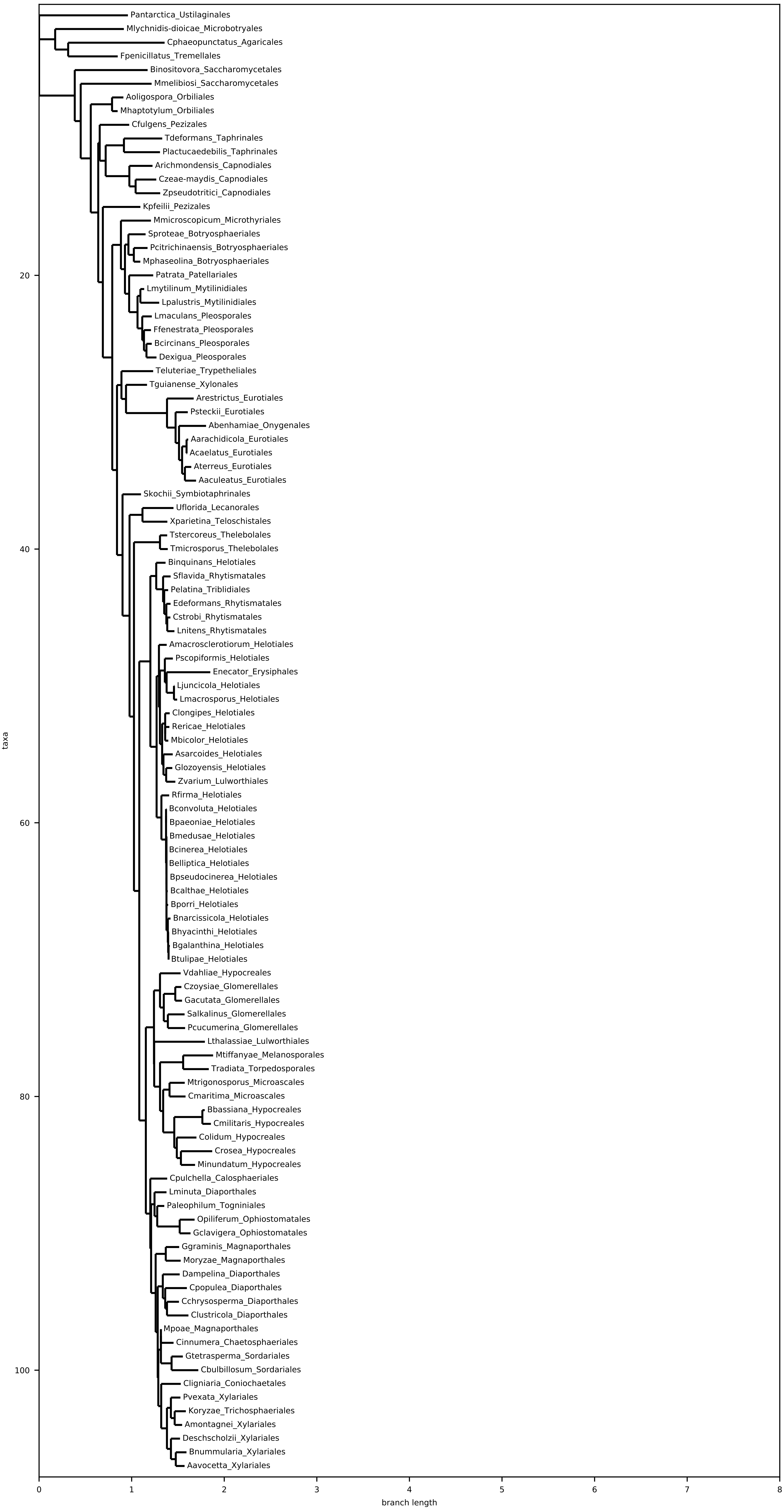
Pantarctica_Ustilaginales
Mlychnidis-dioicae_Microbotryales
Cphaeopunctatus_Agaricales
Fpenicillatus_Tremellales
Binositovora_Saccharomycetales
Mmelibiosi_Saccharomycetales
Aoligospora_Orbiliales
Mhaptotylum_Orbiliales
Cfulgens_Pezizales
Tdeformans_Taphrinales
Plactucaedebilis_Taphrinales
Arichmondensis_Capnodiales
Czeae-maydis_Capnodiales
Zpseudotritici_Capnodiales
Kpfeilii_Pezizales
Mmicroscopicum_Microthyriales
Sproteae_Botryosphaeriales
Pcitrichinaensis_Botryosphaeriales
Mphaseolina_Botryosphaeriales
Patrata_Patellariales
Lmytilinum_Mytilinidiales
Lpalustris_Mytilinidiales
Lmaculans_Pleosporales
Ffenestrata_Pleosporales
Bcircinans_Pleosporales
Dexigua_Pleosporales
Teluteriae_Trypetheliales
Tguianense_Xylonales
Arestrictus_Eurotiales
Psteckii_Eurotiales
Abenhamiae_Onygenales
Aarachidicola_Eurotiales
Acaelatus_Eurotiales
Aterreus_Eurotiales
Aaculeatus_Eurotiales
Skochii_Symbiotaphrinales
Uflorida_Lecanorales
Xparietina_Teloschistales
Tstercoreus_Thelebolales
Tmicrosporus_Thelebolales
Binquinans_Helotiales
Sflavida_Rhytismatales
Pelatina_Triblidiales
Edeformans_Rhytismatales
Cstrobi_Rhytismatales
Lnitens_Rhytismatales
Amacrosclerotiorum_Helotiales
Pscopiformis_Helotiales
Enecator_Erysiphales
Ljuncicola_Helotiales
Lmacrosporus_Helotiales
Clongipes_Helotiales
Rericae_Helotiales
Mbicolor_Helotiales
Asarcoides_Helotiales
Glozoyensis_Helotiales
Zvarium_Lulworthiales
Rfirma_Helotiales
Bconvoluta_Helotiales
Bpaeoniae_Helotiales
Bmedusae_Helotiales
Bcinerea_Helotiales
Belliptica_Helotiales
Bpseudocinerea_Helotiales
Bcalthae_Helotiales
Bporri_Helotiales
Bnarcissicola_Helotiales
Bhyacinthi_Helotiales
Bgalanthina_Helotiales
Btulipae_Helotiales
Vdahliae_Hypocreales
Czoysiae_Glomerellales
Gacutata_Glomerellales
Salkalinus_Glomerellales
Pcucumerina_Glomerellales
Lthalassiae_Lulworthiales
Mtiffanyae_Melanosporales
Tradiata_Torpedosporales
Mtrigonosporus_Microascales
Cmaritima_Microascales
Bbassiana_Hypocreales
Cmilitaris_Hypocreales
Colidum_Hypocreales
Crosea_Hypocreales
Minundatum_Hypocreales
Cpulchella_Calosphaeriales
Lminuta_Diaporthales
Paleophilum_Togniniales
Opiliferum_Ophiostomatales
Gclavigera_Ophiostomatales
Ggraminis_Magnaporthales
Moryzae_Magnaporthales
Dampelina_Diaporthales
Cpopulea_Diaporthales
Cchrysosperma_Diaporthales
Clustricola_Diaporthales
Mpoae_Magnaporthales
Cinnumera_Chaetosphaeriales
Gtetrasperma_Sordariales
Cbulbillosum_Sordariales
Cligniaria_Coniochaetales
Pvexata_Xylariales
Koryzae_Trichosphaeriales
Amontagnei_Xylariales
Deschscholzii_Xylariales
Bnummularia_Xylariales
Aavocetta_Xylariales
taxa
20
40
60
80
100
0
1
2
3
4
5
6
7
8
branch length

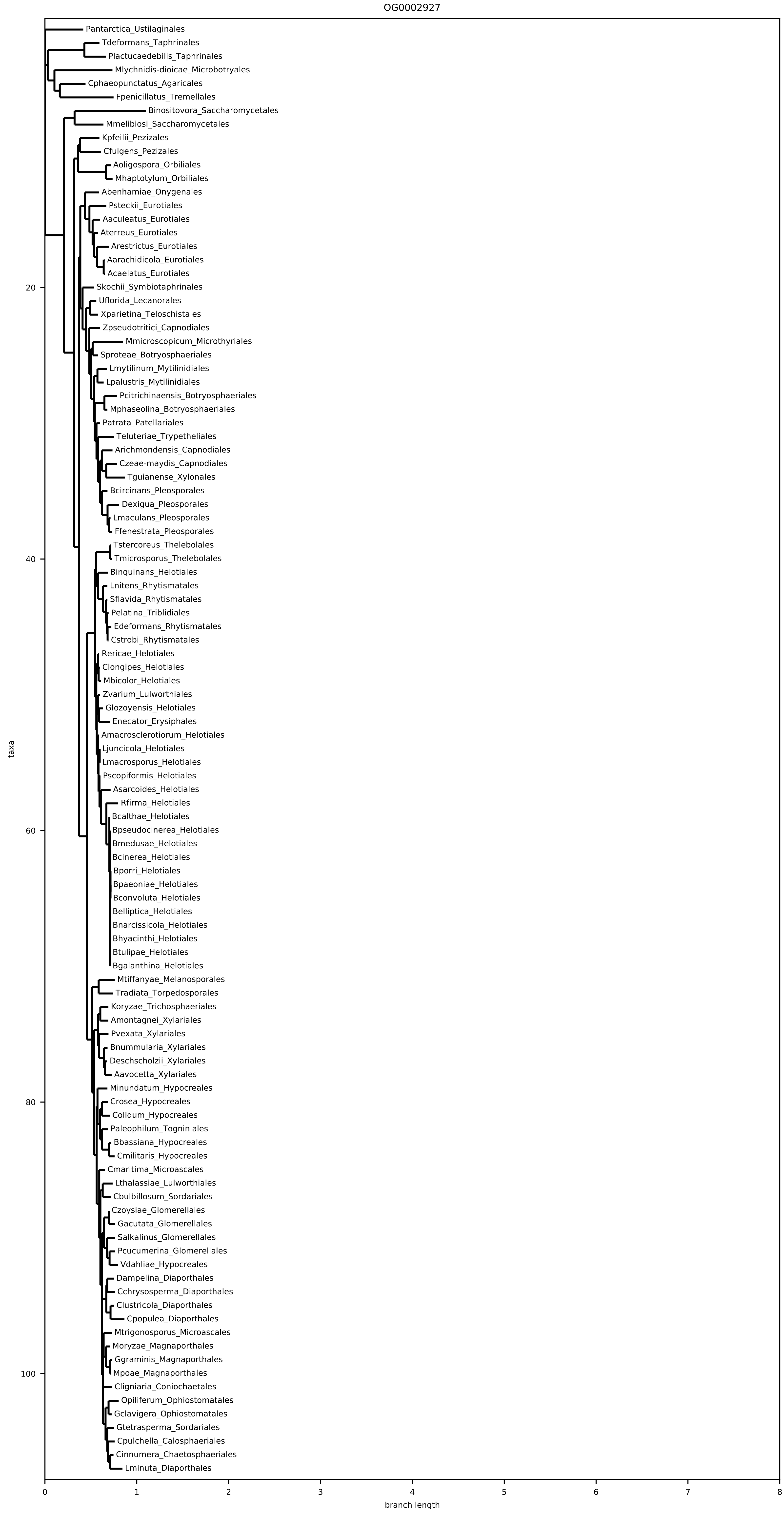
OG0002927
Pantarctica_Ustilaginales
Tdeformans_Taphrinales
Plactucaedebilis_Taphrinales
Mlychnidis-dioicae_Microbotryales
Cphaeopunctatus_Agaricales
Fpenicillatus_Tremellales
Binositovora_Saccharomycetales
Mmelibiosi_Saccharomycetales
Kpfeilii_Pezizales
Cfulgens_Pezizales
Aoligospora_Orbiliales
Mhaptotylum_Orbiliales
Abenhamiae_Onygenales
Psteckii_Eurotiales
Aaculeatus_Eurotiales
Aterreus_Eurotiales
Arestrictus_Eurotiales
Aarachidicola_Eurotiales
Acaelatus_Eurotiales
Skochii_Symbiotaphrinales
Uflorida_Lecanorales
Xparietina_Teloschistales
Zpseudotritici_Capnodiales
Mmicroscopicum_Microthyriales
Sproteae_Botryosphaeriales
Lmytilinum_Mytilinidiales
Lpalustris_Mytilinidiales
Pcitrichinaensis_Botryosphaeriales
Mphaseolina_Botryosphaeriales
Patrata_Patellariales
Teluteriae_Trypetheliales
Arichmondensis_Capnodiales
Czeae-maydis_Capnodiales
Tguianense_Xylonales
Bcircinans_Pleosporales
Dexigua_Pleosporales
Lmaculans_Pleosporales
Ffenestrata_Pleosporales
Tstercoreus_Thelebolales
Tmicrosporus_Thelebolales
Binquinans_Helotiales
Lnitens_Rhytismatales
Sflavida_Rhytismatales
Pelatina_Triblidiales
Edeformans_Rhytismatales
Cstrobi_Rhytismatales
Rericae_Helotiales
Clongipes_Helotiales
Mbicolor_Helotiales
Zvarium_Lulworthiales
Glozoyensis_Helotiales
Enecator_Erysiphales
Amacrosclerotiorum_Helotiales
Ljuncicola_Helotiales
Lmacrosporus_Helotiales
Pscopiformis_Helotiales
Asarcoides_Helotiales
Rfirma_Helotiales
Bcalthae_Helotiales
Bpseudocinerea_Helotiales
Bmedusae_Helotiales
Bcinerea_Helotiales
Bporri_Helotiales
Bpaeoniae_Helotiales
Bconvoluta_Helotiales
Belliptica_Helotiales
Bnarcissicola_Helotiales
Bhyacinthi_Helotiales
Btulipae_Helotiales
Bgalanthina_Helotiales
Mtiffanyae_Melanosporales
Tradiata_Torpedosporales
Koryzae_Trichosphaeriales
Amontagnei_Xylariales
Pvexata_Xylariales
Bnummularia_Xylariales
Deschscholzii_Xylariales
Aavocetta_Xylariales
Minundatum_Hypocreales
Crosea_Hypocreales
Colidum_Hypocreales
Paleophilum_Togniniales
Bbassiana_Hypocreales
Cmilitaris_Hypocreales
Cmaritima_Microascales
Lthalassiae_Lulworthiales
Cbulbillosum_Sordariales
Czoysiae_Glomerellales
Gacutata_Glomerellales
Salkalinus_Glomerellales
Pcucumerina_Glomerellales
Vdahliae_Hypocreales
Dampelina_Diaporthales
Cchrysosperma_Diaporthales
Clustricola_Diaporthales
Cpopulea_Diaporthales
Mtrigonosporus_Microascales
Moryzae_Magnaporthales
Ggraminis_Magnaporthales
Mpoae_Magnaporthales
Cligniaria_Coniochaetales
Opiliferum_Ophiostomatales
Gclavigera_Ophiostomatales
Gtetrasperma_Sordariales
Cpulchella_Calosphaeriales
Cinnumera_Chaetosphaeriales
Lminuta_Diaporthales
taxa
20
40
60
80
100
0
1
2
3
4
5
6
7
8
branch length

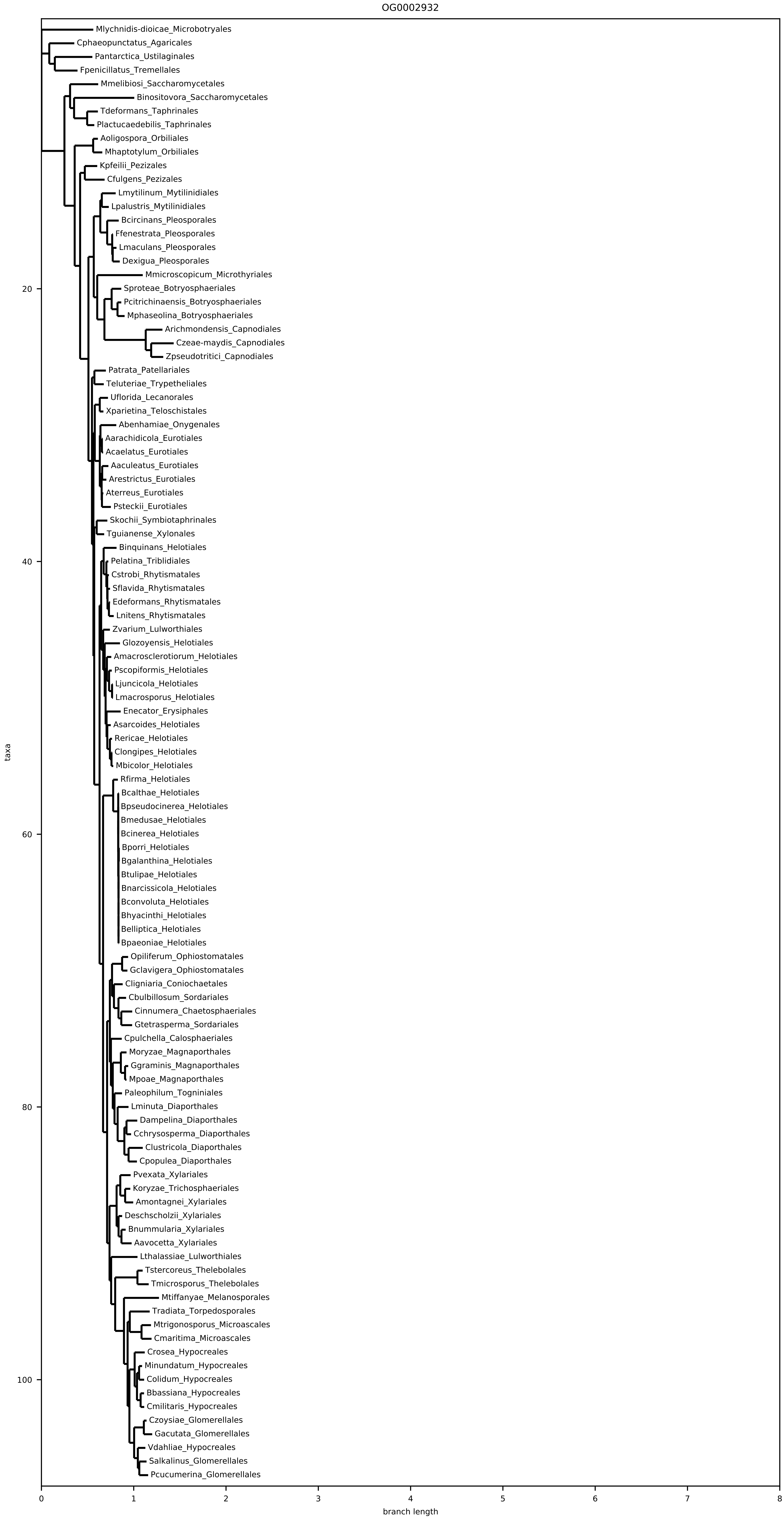

OG0002932
Mlychnidis-dioicae_Microbotryales
Cphaeopunctatus_Agaricales
Pantarctica_Ustilaginales
Fpenicillatus_Tremellales
Mmelibiosi_Saccharomycetales
Binositovora_Saccharomycetales
Tdeformans_Taphrinales
Plactucaedebilis_Taphrinales
Aoligospora_Orbiliales
Mhaptotylum_Orbiliales
Kpfeilii_Pezizales
Cfulgens_Pezizales
Lmytilinum_Mytilinidiales
Lpalustris_Mytilinidiales
Bcircinans_Pleosporales
Ffenestrata_Pleosporales
Lmaculans_Pleosporales
Dexigua_Pleosporales
Mmicroscopicum_Microthyriales
Sproteae_Botryosphaeriales
Pcitrichinaensis_Botryosphaeriales
Mphaseolina_Botryosphaeriales
Arichmondensis_Capnodiales
Czeae-maydis_Capnodiales
Zpseudotritici_Capnodiales
Patrata_Patellariales
Teluteriae_Trypetheliales
Uflorida_Lecanorales
Xparietina_Teloschistales
Abenhamiae_Onygenales
Aarachidicola_Eurotiales
Acaelatus_Eurotiales
Aaculeatus_Eurotiales
Arestrictus_Eurotiales
Aterreus_Eurotiales
Psteckii_Eurotiales
Skochii_Symbiotaphrinales
Tguianense_Xylonales
Binquinans_Helotiales
Pelatina_Triblidiales
Cstrobi_Rhytismatales
Sflavida_Rhytismatales
Edeformans_Rhytismatales
Lnitens_Rhytismatales
Zvarium_Lulworthiales
Glozoyensis_Helotiales
Amacrosclerotiorum_Helotiales
Pscopiformis_Helotiales
Ljuncicola_Helotiales
Lmacrosporus_Helotiales
Enecator_Erysiphales
Asarcoides_Helotiales
Rericae_Helotiales
Clongipes_Helotiales
Mbicolor_Helotiales
Rfirma_Helotiales
Bcalthae_Helotiales
Bpseudocinerea_Helotiales
Bmedusae_Helotiales
Bcinerea_Helotiales
Bporri_Helotiales
Bgalanthina_Helotiales
Btulipae_Helotiales
Bnarcissicola_Helotiales
Bconvoluta_Helotiales
Bhyacinthi_Helotiales
Belliptica_Helotiales
Bpaeoniae_Helotiales
Opiliferum_Ophiostomatales
Gclavigera_Ophiostomatales
Cligniaria_Coniochaetales
Cbulbillosum_Sordariales
Cinnumera_Chaetosphaeriales
Gtetrasperma_Sordariales
Cpulchella_Calosphaeriales
Moryzae_Magnaporthales
Ggraminis_Magnaporthales
Mpoae_Magnaporthales
Paleophilum_Togniniales
Lminuta_Diaporthales
Dampelina_Diaporthales
Cchrysosperma_Diaporthales
Clustricola_Diaporthales
Cpopulea_Diaporthales
Pvexata_Xylariales
Koryzae_Trichosphaeriales
Amontagnei_Xylariales
Deschscholzii_Xylariales
Bnummularia_Xylariales
Aavocetta_Xylariales
Lthalassiae_Lulworthiales
Tstercoreus_Thelebolales
Tmicrosporus_Thelebolales
Mtiffanyae_Melanosporales
Tradiata_Torpedosporales
Mtrigonosporus_Microascales
Cmaritima_Microascales
Crosea_Hypocreales
Minundatum_Hypocreales
Colidum_Hypocreales
Bbassiana_Hypocreales
Cmilitaris_Hypocreales
Czoysiae_Glomerellales
Gacutata_Glomerellales
Vdahliae_Hypocreales
Salkalinus_Glomerellales
Pcucumerina_Glomerellales
taxa
20
40
60
80
100
0
1
2
3
4
5
6
7
8
branch length

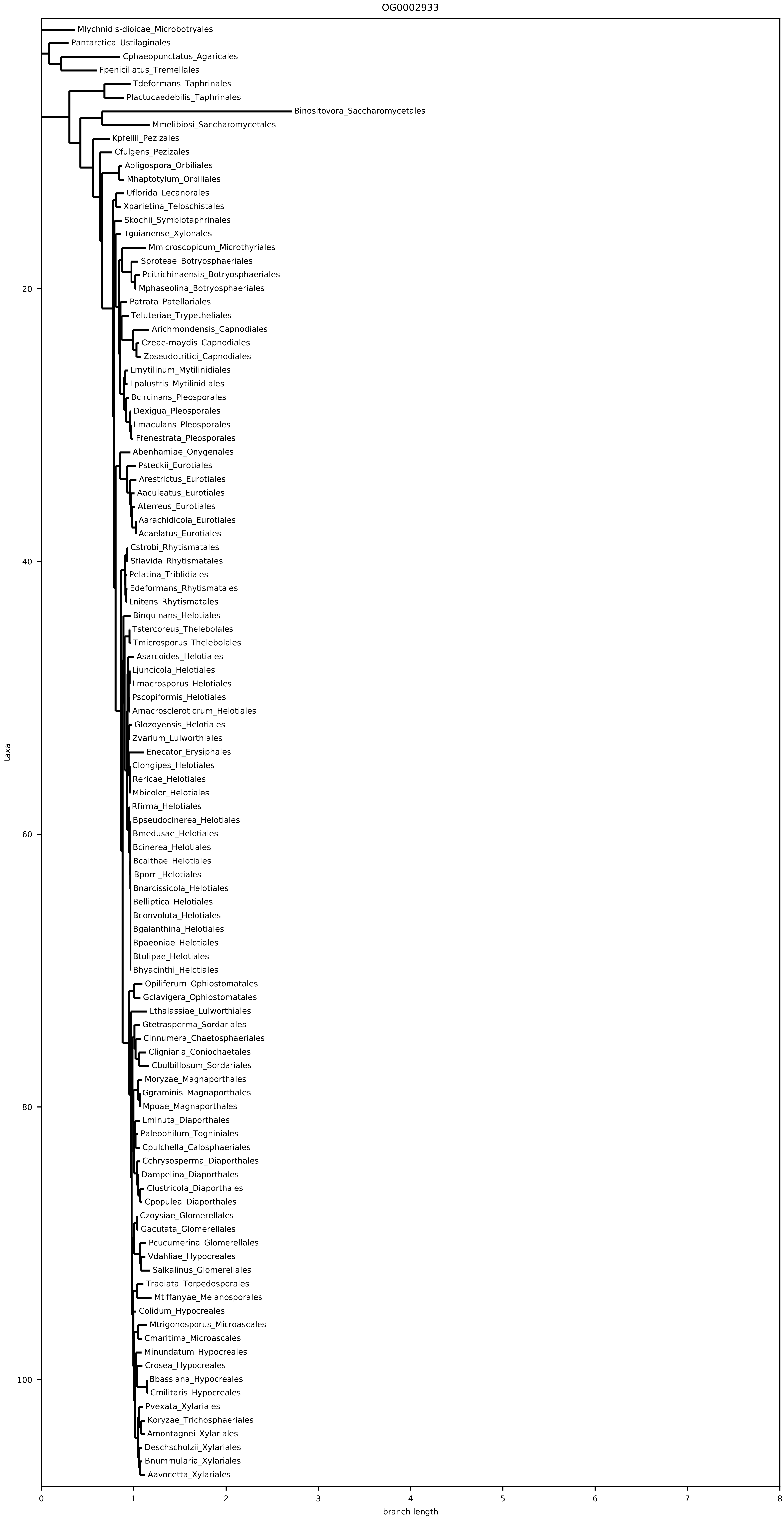

OG0002933
Mlychnidis-dioicae_Microbotryales
Pantarctica_Ustilaginales
Cphaeopunctatus_Agaricales
Fpenicillatus_Tremellales
Tdeformans_Taphrinales
Plactucaedebilis_Taphrinales
Binositovora_Saccharomycetales
Mmelibiosi_Saccharomycetales
Kpfeilii_Pezizales
Cfulgens_Pezizales
Aoligospora_Orbiliales
Mhaptotylum_Orbiliales
Uflorida_Lecanorales
Xparietina_Teloschistales
Skochii_Symbiotaphrinales
Tguianense_Xylonales
Mmicroscopicum_Microthyriales
Sproteae_Botryosphaeriales
Pcitrichinaensis_Botryosphaeriales
Mphaseolina_Botryosphaeriales
Patrata_Patellariales
Teluteriae_Trypetheliales
Arichmondensis_Capnodiales
Czeae-maydis_Capnodiales
Zpseudotritici_Capnodiales
Lmytilinum_Mytilinidiales
Lpalustris_Mytilinidiales
Bcircinans_Pleosporales
Dexigua_Pleosporales
Lmaculans_Pleosporales
Ffenestrata_Pleosporales
Abenhamiae_Onygenales
Psteckii_Eurotiales
Arestrictus_Eurotiales
Aaculeatus_Eurotiales
Aterreus_Eurotiales
Aarachidicola_Eurotiales
Acaelatus_Eurotiales
Cstrobi_Rhytismatales
Sflavida_Rhytismatales
Pelatina_Triblidiales
Edeformans_Rhytismatales
Lnitens_Rhytismatales
Binquinans_Helotiales
Tstercoreus_Thelebolales
Tmicrosporus_Thelebolales
Asarcoides_Helotiales
Ljuncicola_Helotiales
Lmacrosporus_Helotiales
Pscopiformis_Helotiales
Amacrosclerotiorum_Helotiales
Glozoyensis_Helotiales
Zvarium_Lulworthiales
Enecator_Erysiphales
Clongipes_Helotiales
Rericae_Helotiales
Mbicolor_Helotiales
Rfirma_Helotiales
Bpseudocinerea_Helotiales
Bmedusae_Helotiales
Bcinerea_Helotiales
Bcalthae_Helotiales
Bporri_Helotiales
Bnarcissicola_Helotiales
Belliptica_Helotiales
Bconvoluta_Helotiales
Bgalanthina_Helotiales
Bpaeoniae_Helotiales
Btulipae_Helotiales
Bhyacinthi_Helotiales
Opiliferum_Ophiostomatales
Gclavigera_Ophiostomatales
Lthalassiae_Lulworthiales
Gtetrasperma_Sordariales
Cinnumera_Chaetosphaeriales
Cligniaria_Coniochaetales
Cbulbillosum_Sordariales
Moryzae_Magnaporthales
Ggraminis_Magnaporthales
Mpoae_Magnaporthales
Lminuta_Diaporthales
Paleophilum_Togniniales
Cpulchella_Calosphaeriales
Cchrysosperma_Diaporthales
Dampelina_Diaporthales
Clustricola_Diaporthales
Cpopulea_Diaporthales
Czoysiae_Glomerellales
Gacutata_Glomerellales
Pcucumerina_Glomerellales
Vdahliae_Hypocreales
Salkalinus_Glomerellales
Tradiata_Torpedosporales
Mtiffanyae_Melanosporales
Colidum_Hypocreales
Mtrigonosporus_Microascales
Cmaritima_Microascales
Minundatum_Hypocreales
Crosea_Hypocreales
Bbassiana_Hypocreales
Cmilitaris_Hypocreales
Pvexata_Xylariales
Koryzae_Trichosphaeriales
Amontagnei_Xylariales
Deschscholzii_Xylariales
Bnummularia_Xylariales
Aavocetta_Xylariales
taxa
20
40
60
80
100
0
1
2
3
4
5
6
7
8
branch length

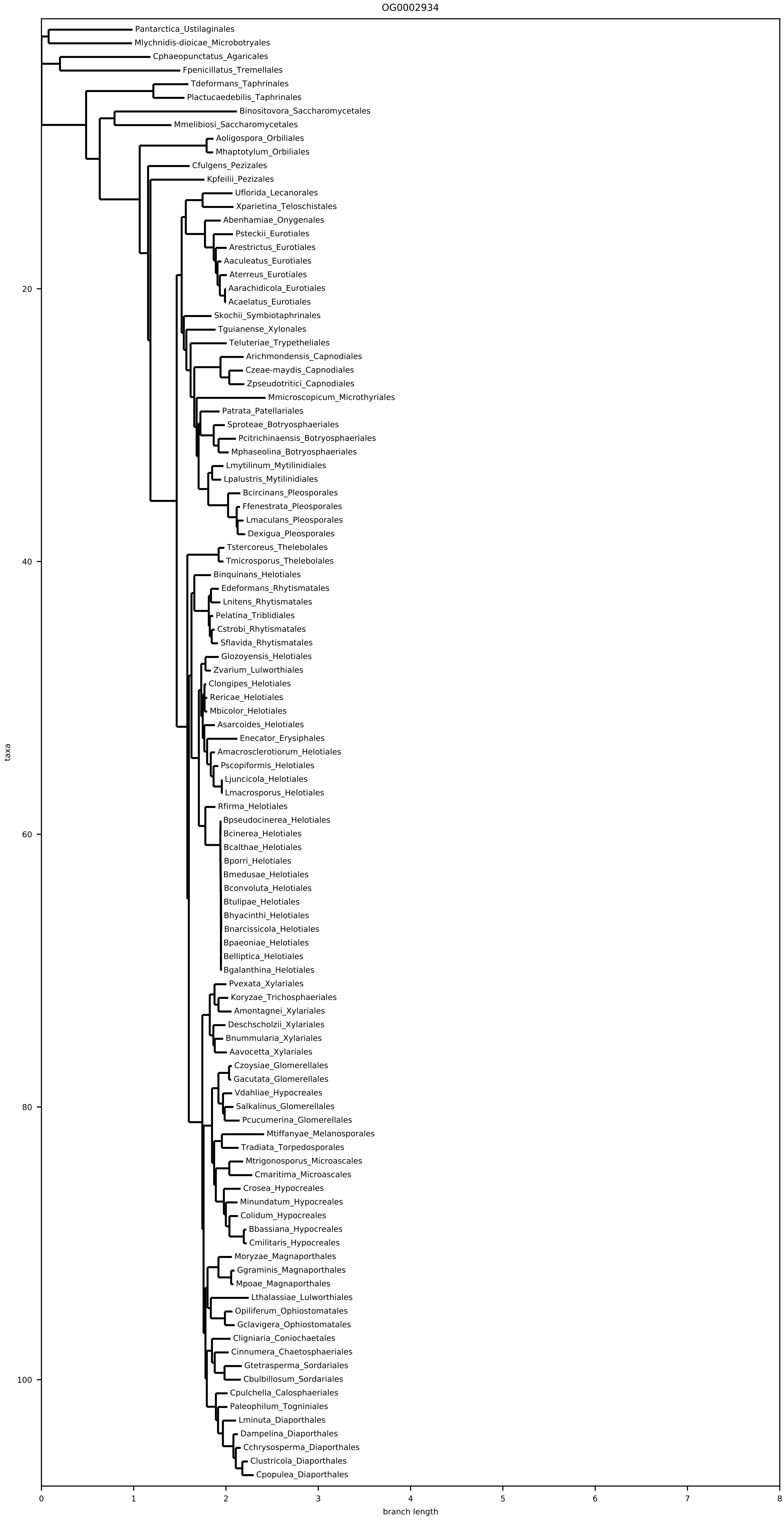
OG0002934
Pantarctica_Ustilaginales
Mlychnidis-dioicae_Microbotryales
Cphaeopunctatus_Agaricales
Fpenicillatus_Tremellales
Tdeformans_Taphrinales
Plactucaedebilis_Taphrinales
Binositovora_Saccharomycetales
Mmelibiosi_Saccharomycetales
Aoligospora_Orbiliales
Mhaptotylum_Orbiliales
Cfulgens_Pezizales
Kpfeilii_Pezizales
Uflorida_Lecanorales
Xparietina_Teloschistales
Abenhamiae_Onygenales
Psteckii_Eurotiales
Arestrictus_Eurotiales
Aaculeatus_Eurotiales
Aterreus_Eurotiales
Aarachidicola_Eurotiales
Acaelatus_Eurotiales
Skochii_Symbiotaphrinales
Tguianense_Xylonales
Teluteriae_Trypetheliales
Arichmondensis_Capnodiales
Czeae-maydis_Capnodiales
Zpseudotritici_Capnodiales
Mmicroscopicum_Microthyriales
Patrata_Patellariales
Sproteae_Botryosphaeriales
Pcitrichinaensis_Botryosphaeriales
Mphaseolina_Botryosphaeriales
Lmytilinum_Mytilinidiales
Lpalustris_Mytilinidiales
Bcircinans_Pleosporales
Ffenestrata_Pleosporales
Lmaculans_Pleosporales
Dexigua_Pleosporales
Tstercoreus_Thelebolales
Tmicrosporus_Thelebolales
Binquinans_Helotiales
Edeformans_Rhytismatales
Lnitens_Rhytismatales
Pelatina_Triblidiales
Cstrobi_Rhytismatales
Sflavida_Rhytismatales
Glozoyensis_Helotiales
Zvarium_Lulworthiales
Clongipes_Helotiales
Rericae_Helotiales
Mbicolor_Helotiales
Asarcoides_Helotiales
Enecator_Erysiphales
Amacrosclerotiorum_Helotiales
Pscopiformis_Helotiales
Ljuncicola_Helotiales
Lmacrosporus_Helotiales
Rfirma_Helotiales
Bpseudocinerea_Helotiales
Bcinerea_Helotiales
Bcalthae_Helotiales
Bporri_Helotiales
Bmedusae_Helotiales
Bconvoluta_Helotiales
Btulipae_Helotiales
Bhyacinthi_Helotiales
Bnarcissicola_Helotiales
Bpaeoniae_Helotiales
Belliptica_Helotiales
Bgalanthina_Helotiales
Pvexata_Xylariales
Koryzae_Trichosphaeriales
Amontagnei_Xylariales
Deschscholzii_Xylariales
Bnummularia_Xylariales
Aavocetta_Xylariales
Czoysiae_Glomerellales
Gacutata_Glomerellales
Vdahliae_Hypocreales
Salkalinus_Glomerellales
Pcucumerina_Glomerellales
Mtiffanyae_Melanosporales
Tradiata_Torpedosporales
Mtrigonosporus_Microascales
Cmaritima_Microascales
Crosea_Hypocreales
Minundatum_Hypocreales
Colidum_Hypocreales
Bbassiana_Hypocreales
Cmilitaris_Hypocreales
Moryzae_Magnaporthales
Ggraminis_Magnaporthales
Mpoae_Magnaporthales
Lthalassiae_Lulworthiales
Opiliferum_Ophiostomatales
Gclavigera_Ophiostomatales
Cligniaria_Coniochaetales
Cinnumera_Chaetosphaeriales
Gtetrasperma_Sordariales
Cbulbillosum_Sordariales
Cpulchella_Calosphaeriales
Paleophilum_Togniniales
Lminuta_Diaporthales
Dampelina_Diaporthales
Cchrysosperma_Diaporthales
Clustricola_Diaporthales
Cpopulea_Diaporthales
taxa
20
40
60
80
100
0
1
2
3
4
5
6
7
8
branch length

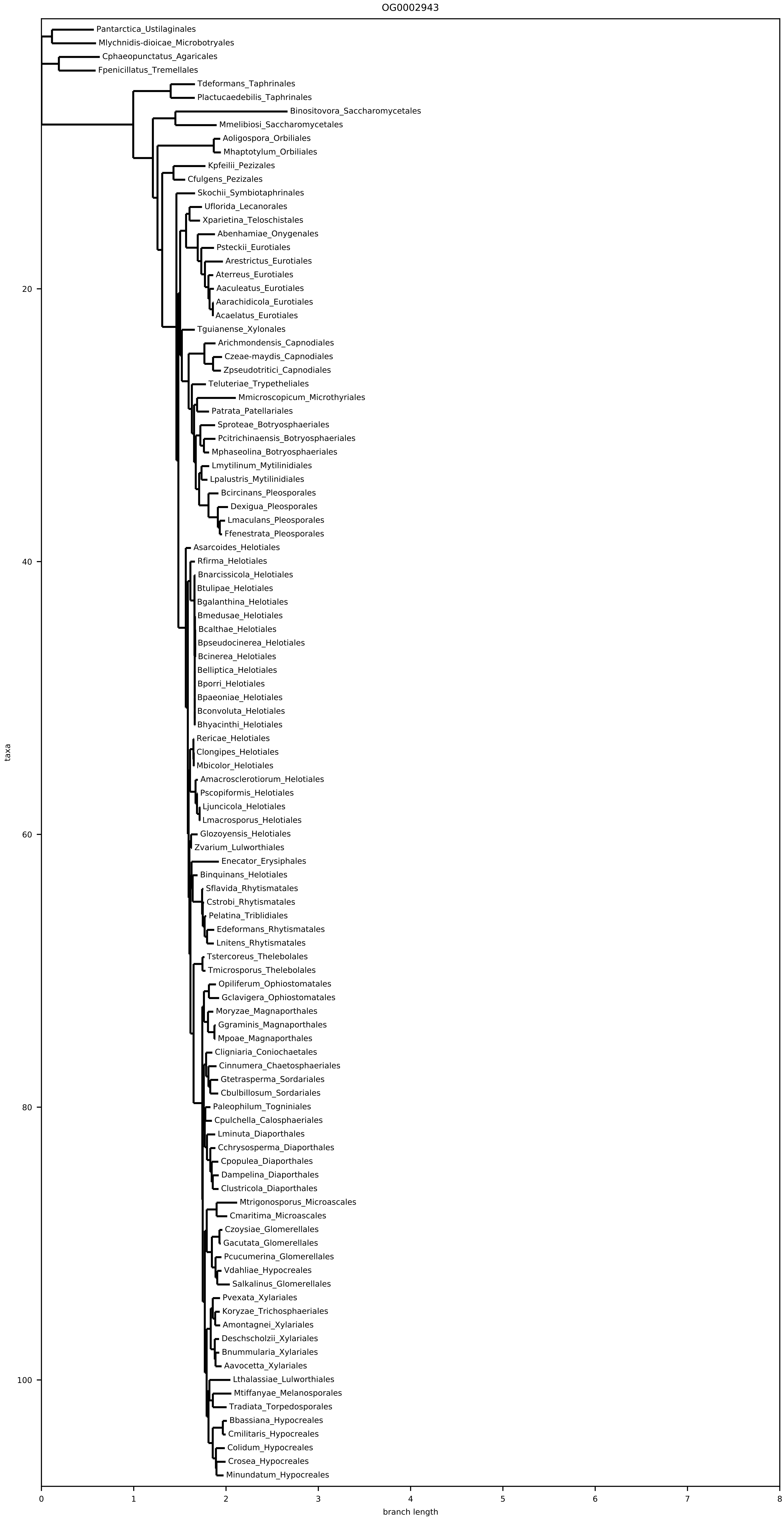

OG0002943
Pantarctica_Ustilaginales
Mlychnidis-dioicae_Microbotryales
Cphaeopunctatus_Agaricales
Fpenicillatus_Tremellales
Tdeformans_Taphrinales
Plactucaedebilis_Taphrinales
Binositovora_Saccharomycetales
Mmelibiosi_Saccharomycetales
Aoligospora_Orbiliales
Mhaptotylum_Orbiliales
Kpfeilii_Pezizales
Cfulgens_Pezizales
Skochii_Symbiotaphrinales
Uflorida_Lecanorales
Xparietina_Teloschistales
Abenhamiae_Onygenales
Psteckii_Eurotiales
Arestrictus_Eurotiales
Aterreus_Eurotiales
Aaculeatus_Eurotiales
Aarachidicola_Eurotiales
Acaelatus_Eurotiales
Tguianense_Xylonales
Arichmondensis_Capnodiales
Czeae-maydis_Capnodiales
Zpseudotritici_Capnodiales
Teluteriae_Trypetheliales
Mmicroscopicum_Microthyriales
Patrata_Patellariales
Sproteae_Botryosphaeriales
Pcitrichinaensis_Botryosphaeriales
Mphaseolina_Botryosphaeriales
Lmytilinum_Mytilinidiales
Lpalustris_Mytilinidiales
Bcircinans_Pleosporales
Dexigua_Pleosporales
Lmaculans_Pleosporales
Ffenestrata_Pleosporales
Asarcoides_Helotiales
Rfirma_Helotiales
Bnarcissicola_Helotiales
Btulipae_Helotiales
Bgalanthina_Helotiales
Bmedusae_Helotiales
Bcalthae_Helotiales
Bpseudocinerea_Helotiales
Bcinerea_Helotiales
Belliptica_Helotiales
Bporri_Helotiales
Bpaeoniae_Helotiales
Bconvoluta_Helotiales
Bhyacinthi_Helotiales
Rericae_Helotiales
Clongipes_Helotiales
Mbicolor_Helotiales
Amacrosclerotiorum_Helotiales
Pscopiformis_Helotiales
Ljuncicola_Helotiales
Lmacrosporus_Helotiales
Glozoyensis_Helotiales
Zvarium_Lulworthiales
Enecator_Erysiphales
Binquinans_Helotiales
Sflavida_Rhytismatales
Cstrobi_Rhytismatales
Pelatina_Triblidiales
Edeformans_Rhytismatales
Lnitens_Rhytismatales
Tstercoreus_Thelebolales
Tmicrosporus_Thelebolales
Opiliferum_Ophiostomatales
Gclavigera_Ophiostomatales
Moryzae_Magnaporthales
Ggraminis_Magnaporthales
Mpoae_Magnaporthales
Cligniaria_Coniochaetales
Cinnumera_Chaetosphaeriales
Gtetrasperma_Sordariales
Cbulbillosum_Sordariales
Paleophilum_Togniniales
Cpulchella_Calosphaeriales
Lminuta_Diaporthales
Cchrysosperma_Diaporthales
Cpopulea_Diaporthales
Dampelina_Diaporthales
Clustricola_Diaporthales
Mtrigonosporus_Microascales
Cmaritima_Microascales
Czoysiae_Glomerellales
Gacutata_Glomerellales
Pcucumerina_Glomerellales
Vdahliae_Hypocreales
Salkalinus_Glomerellales
Pvexata_Xylariales
Koryzae_Trichosphaeriales
Amontagnei_Xylariales
Deschscholzii_Xylariales
Bnummularia_Xylariales
Aavocetta_Xylariales
Lthalassiae_Lulworthiales
Mtiffanyae_Melanosporales
Tradiata_Torpedosporales
Bbassiana_Hypocreales
Cmilitaris_Hypocreales
Colidum_Hypocreales
Crosea_Hypocreales
Minundatum_Hypocreales
taxa
20
40
60
80
100
0
1
2
3
4
5
6
7
8
branch length

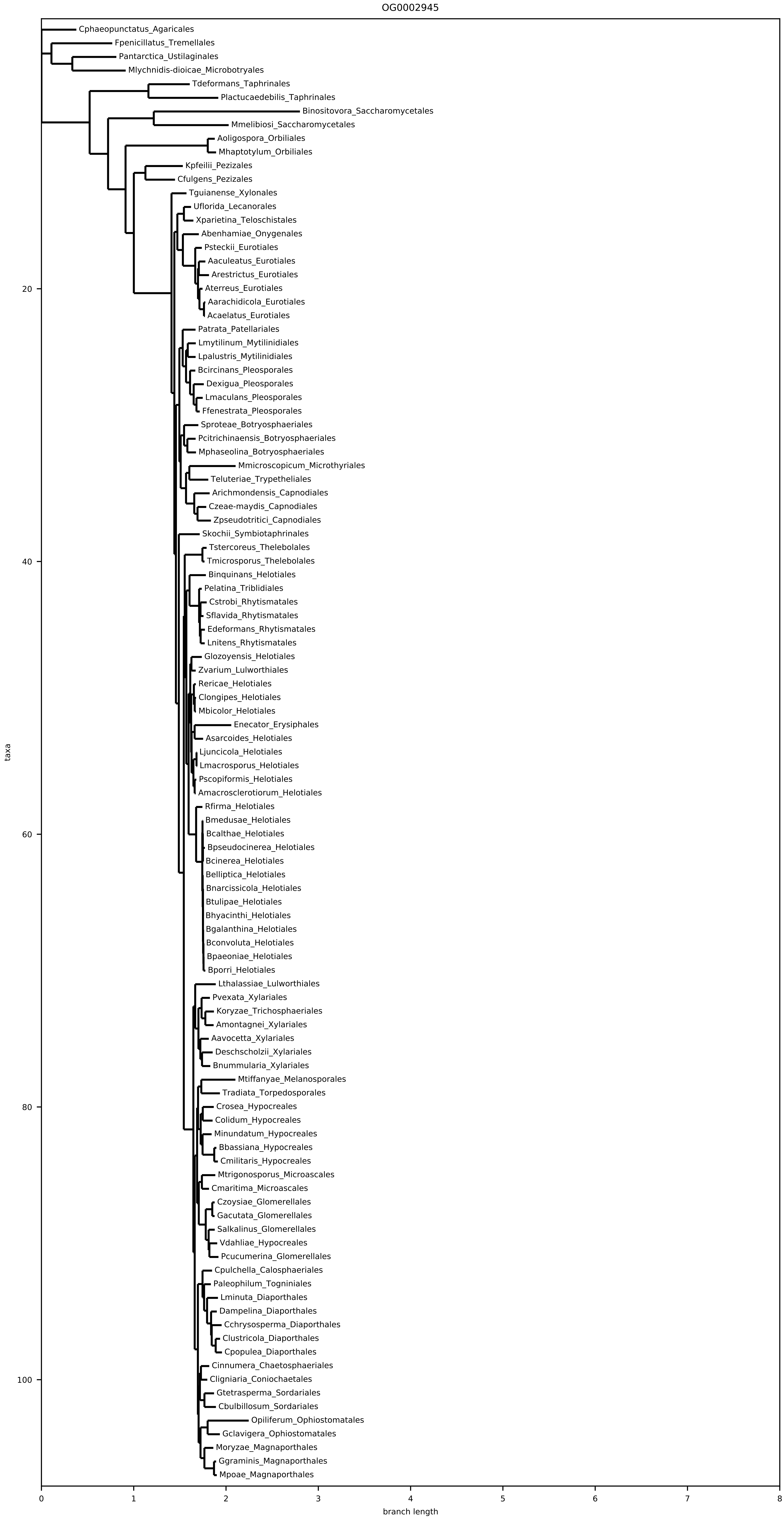

OG0002945
Cphaeopunctatus_Agaricales
Fpenicillatus_Tremellales
Pantarctica_Ustilaginales
Mlychnidis-dioicae_Microbotryales
Tdeformans_Taphrinales
Plactucaedebilis_Taphrinales
Binositovora_Saccharomycetales
Mmelibiosi_Saccharomycetales
Aoligospora_Orbiliales
Mhaptotylum_Orbiliales
Kpfeilii_Pezizales
Cfulgens_Pezizales
Tguianense_Xylonales
Uflorida_Lecanorales
Xparietina_Teloschistales
Abenhamiae_Onygenales
Psteckii_Eurotiales
Aaculeatus_Eurotiales
Arestrictus_Eurotiales
Aterreus_Eurotiales
Aarachidicola_Eurotiales
Acaelatus_Eurotiales
Patrata_Patellariales
Lmytilinum_Mytilinidiales
Lpalustris_Mytilinidiales
Bcircinans_Pleosporales
Dexigua_Pleosporales
Lmaculans_Pleosporales
Ffenestrata_Pleosporales
Sproteae_Botryosphaeriales
Pcitrichinaensis_Botryosphaeriales
Mphaseolina_Botryosphaeriales
Mmicroscopicum_Microthyriales
Teluteriae_Trypetheliales
Arichmondensis_Capnodiales
Czeae-maydis_Capnodiales
Zpseudotritici_Capnodiales
Skochii_Symbiotaphrinales
Tstercoreus_Thelebolales
Tmicrosporus_Thelebolales
Binquinans_Helotiales
Pelatina_Triblidiales
Cstrobi_Rhytismatales
Sflavida_Rhytismatales
Edeformans_Rhytismatales
Lnitens_Rhytismatales
Glozoyensis_Helotiales
Zvarium_Lulworthiales
Rericae_Helotiales
Clongipes_Helotiales
Mbicolor_Helotiales
Enecator_Erysiphales
Asarcoides_Helotiales
Ljuncicola_Helotiales
Lmacrosporus_Helotiales
Pscopiformis_Helotiales
Amacrosclerotiorum_Helotiales
Rfirma_Helotiales
Bmedusae_Helotiales
Bcalthae_Helotiales
Bpseudocinerea_Helotiales
Bcinerea_Helotiales
Belliptica_Helotiales
Bnarcissicola_Helotiales
Btulipae_Helotiales
Bhyacinthi_Helotiales
Bgalanthina_Helotiales
Bconvoluta_Helotiales
Bpaeoniae_Helotiales
Bporri_Helotiales
Lthalassiae_Lulworthiales
Pvexata_Xylariales
Koryzae_Trichosphaeriales
Amontagnei_Xylariales
Aavocetta_Xylariales
Deschscholzii_Xylariales
Bnummularia_Xylariales
Mtiffanyae_Melanosporales
Tradiata_Torpedosporales
Crosea_Hypocreales
Colidum_Hypocreales
Minundatum_Hypocreales
Bbassiana_Hypocreales
Cmilitaris_Hypocreales
Mtrigonosporus_Microascales
Cmaritima_Microascales
Czoysiae_Glomerellales
Gacutata_Glomerellales
Salkalinus_Glomerellales
Vdahliae_Hypocreales
Pcucumerina_Glomerellales
Cpulchella_Calosphaeriales
Paleophilum_Togniniales
Lminuta_Diaporthales
Dampelina_Diaporthales
Cchrysosperma_Diaporthales
Clustricola_Diaporthales
Cpopulea_Diaporthales
Cinnumera_Chaetosphaeriales
Cligniaria_Coniochaetales
Gtetrasperma_Sordariales
Cbulbillosum_Sordariales
Opiliferum_Ophiostomatales
Gclavigera_Ophiostomatales
Moryzae_Magnaporthales
Ggraminis_Magnaporthales
Mpoae_Magnaporthales
taxa
20
40
60
80
100
0
1
2
3
4
5
6
7
8
branch length

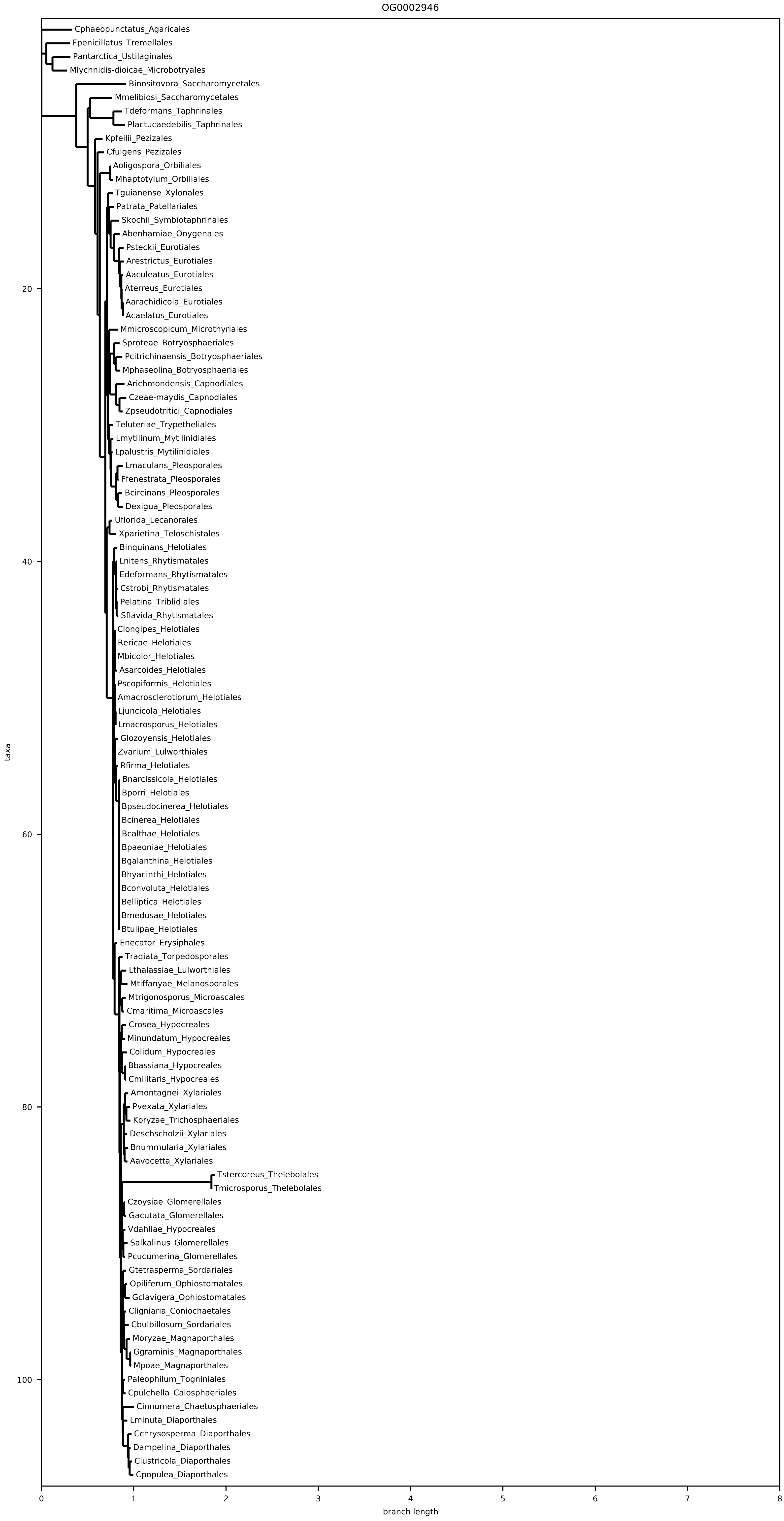
OG0002946
Cphaeopunctatus_Agaricales
Fpenicillatus_Tremellales
Pantarctica_Ustilaginales
Mlychnidis-dioicae_Microbotryales
Binositovora_Saccharomycetales
Mmelibiosi_Saccharomycetales
Tdeformans_Taphrinales
Plactucaedebilis_Taphrinales
Kpfeilii_Pezizales
Cfulgens_Pezizales
Aoligospora_Orbiliales
Mhaptotylum_Orbiliales
Tguianense_Xylonales
Patrata_Patellariales
Skochii_Symbiotaphrinales
Abenhamiae_Onygenales
Psteckii_Eurotiales
Arestrictus_Eurotiales
Aaculeatus_Eurotiales
Aterreus_Eurotiales
Aarachidicola_Eurotiales
Acaelatus_Eurotiales
Mmicroscopicum_Microthyriales
Sproteae_Botryosphaeriales
Pcitrichinaensis_Botryosphaeriales
Mphaseolina_Botryosphaeriales
Arichmondensis_Capnodiales
Czeae-maydis_Capnodiales
Zpseudotritici_Capnodiales
Teluteriae_Trypetheliales
Lmytilinum_Mytilinidiales
Lpalustris_Mytilinidiales
Lmaculans_Pleosporales
Ffenestrata_Pleosporales
Bcircinans_Pleosporales
Dexigua_Pleosporales
Uflorida_Lecanorales
Xparietina_Teloschistales
Binquinans_Helotiales
Lnitens_Rhytismatales
Edeformans_Rhytismatales
Cstrobi_Rhytismatales
Pelatina_Triblidiales
Sflavida_Rhytismatales
Clongipes_Helotiales
Rericae_Helotiales
Mbicolor_Helotiales
Asarcoides_Helotiales
Pscopiformis_Helotiales
Amacrosclerotiorum_Helotiales
Ljuncicola_Helotiales
Lmacrosporus_Helotiales
Glozoyensis_Helotiales
Zvarium_Lulworthiales
Rfirma_Helotiales
Bnarcissicola_Helotiales
Bporri_Helotiales
Bpseudocinerea_Helotiales
Bcinerea_Helotiales
Bcalthae_Helotiales
Bpaeoniae_Helotiales
Bgalanthina_Helotiales
Bhyacinthi_Helotiales
Bconvoluta_Helotiales
Belliptica_Helotiales
Bmedusae_Helotiales
Btulipae_Helotiales
Enecator_Erysiphales
Tradiata_Torpedosporales
Lthalassiae_Lulworthiales
Mtiffanyae_Melanosporales
Mtrigonosporus_Microascales
Cmaritima_Microascales
Crosea_Hypocreales
Minundatum_Hypocreales
Colidum_Hypocreales
Bbassiana_Hypocreales
Cmilitaris_Hypocreales
Amontagnei_Xylariales
Pvexata_Xylariales
Koryzae_Trichosphaeriales
Deschscholzii_Xylariales
Bnummularia_Xylariales
Aavocetta_Xylariales
Tstercoreus_Thelebolales
Tmicrosporus_Thelebolales
Czoysiae_Glomerellales
Gacutata_Glomerellales
Vdahliae_Hypocreales
Salkalinus_Glomerellales
Pcucumerina_Glomerellales
Gtetrasperma_Sordariales
Opiliferum_Ophiostomatales
Gclavigera_Ophiostomatales
Cligniaria_Coniochaetales
Cbulbillosum_Sordariales
Moryzae_Magnaporthales
Ggraminis_Magnaporthales
Mpoae_Magnaporthales
Paleophilum_Togniniales
Cpulchella_Calosphaeriales
Cinnumera_Chaetosphaeriales
Lminuta_Diaporthales
Cchrysosperma_Diaporthales
Dampelina_Diaporthales
Clustricola_Diaporthales
Cpopulea_Diaporthales
taxa
20
40
60
80
100
0
1
2
3
4
5
6
7
8
branch length

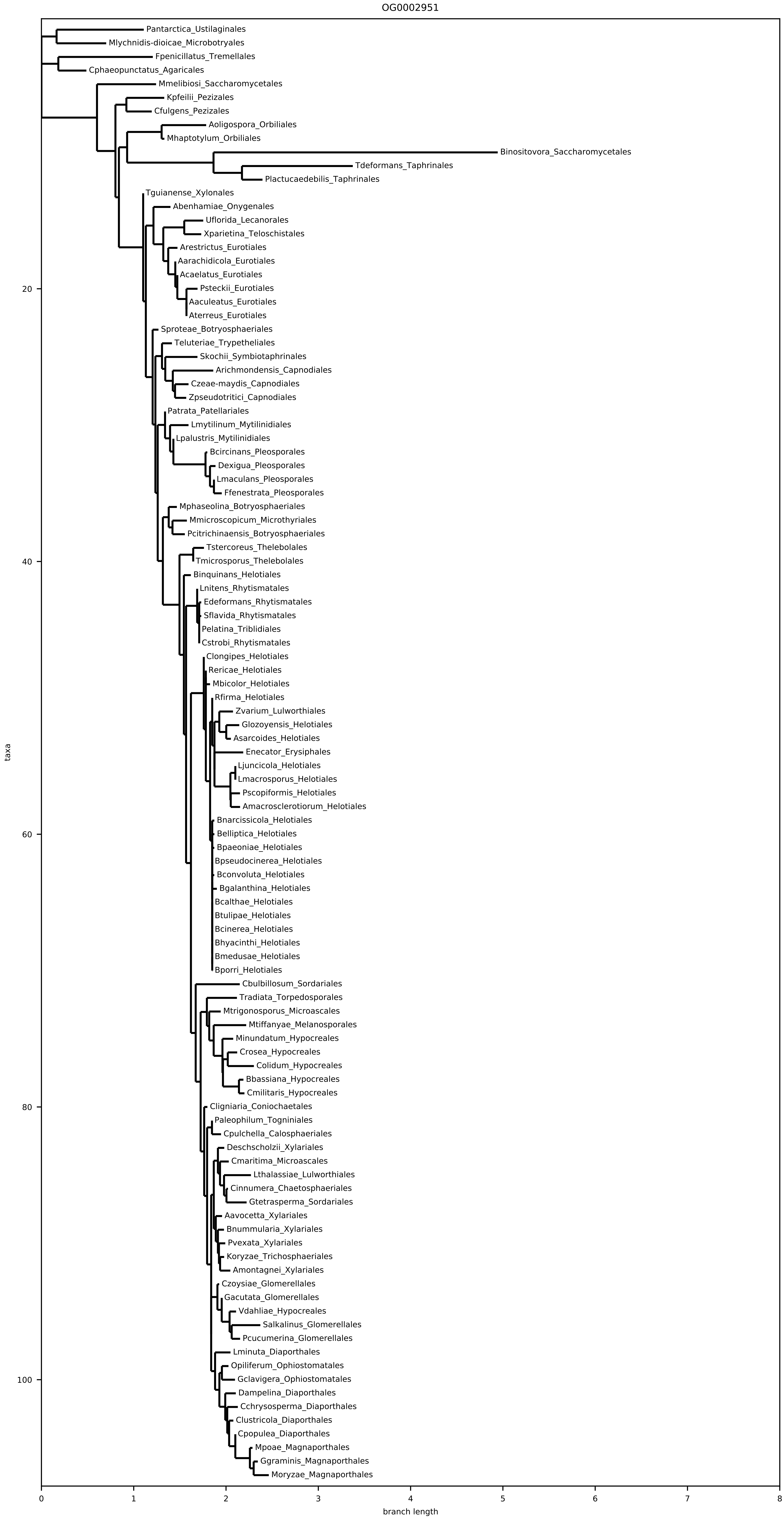
OG0002951
Pantarctica_Ustilaginales
Mlychnidis-dioicae_Microbotryales
Fpenicillatus_Tremellales
Cphaeopunctatus_Agaricales
Mmelibiosi_Saccharomycetales
Kpfeilii_Pezizales
Cfulgens_Pezizales
Aoligospora_Orbiliales
Mhaptotylum_Orbiliales
Binositovora_Saccharomycetales
Tdeformans_Taphrinales
Plactucaedebilis_Taphrinales
Tguianense_Xylonales
Abenhamiae_Onygenales
Uflorida_Lecanorales
Xparietina_Teloschistales
Arestrictus_Eurotiales
Aarachidicola_Eurotiales
Acaelatus_Eurotiales
Psteckii_Eurotiales
Aaculeatus_Eurotiales
Aterreus_Eurotiales
Sproteae_Botryosphaeriales
Teluteriae_Trypetheliales
Skochii_Symbiotaphrinales
Arichmondensis_Capnodiales
Czeae-maydis_Capnodiales
Zpseudotritici_Capnodiales
Patrata_Patellariales
Lmytilinum_Mytilinidiales
Lpalustris_Mytilinidiales
Bcircinans_Pleosporales
Dexigua_Pleosporales
Lmaculans_Pleosporales
Ffenestrata_Pleosporales
Mphaseolina_Botryosphaeriales
Mmicroscopicum_Microthyriales
Pcitrichinaensis_Botryosphaeriales
Tstercoreus_Thelebolales
Tmicrosporus_Thelebolales
Binquinans_Helotiales
Lnitens_Rhytismatales
Edeformans_Rhytismatales
Sflavida_Rhytismatales
Pelatina_Triblidiales
Cstrobi_Rhytismatales
Clongipes_Helotiales
Rericae_Helotiales
Mbicolor_Helotiales
Rfirma_Helotiales
Zvarium_Lulworthiales
Glozoyensis_Helotiales
Asarcoides_Helotiales
Enecator_Erysiphales
Ljuncicola_Helotiales
Lmacrosporus_Helotiales
Pscopiformis_Helotiales
Amacrosclerotiorum_Helotiales
Bnarcissicola_Helotiales
Belliptica_Helotiales
Bpaeoniae_Helotiales
Bpseudocinerea_Helotiales
Bconvoluta_Helotiales
Bgalanthina_Helotiales
Bcalthae_Helotiales
Btulipae_Helotiales
Bcinerea_Helotiales
Bhyacinthi_Helotiales
Bmedusae_Helotiales
Bporri_Helotiales
Cbulbillosum_Sordariales
Tradiata_Torpedosporales
Mtrigonosporus_Microascales
Mtiffanyae_Melanosporales
Minundatum_Hypocreales
Crosea_Hypocreales
Colidum_Hypocreales
Bbassiana_Hypocreales
Cmilitaris_Hypocreales
Cligniaria_Coniochaetales
Paleophilum_Togniniales
Cpulchella_Calosphaeriales
Deschscholzii_Xylariales
Cmaritima_Microascales
Lthalassiae_Lulworthiales
Cinnumera_Chaetosphaeriales
Gtetrasperma_Sordariales
Aavocetta_Xylariales
Bnummularia_Xylariales
Pvexata_Xylariales
Koryzae_Trichosphaeriales
Amontagnei_Xylariales
Czoysiae_Glomerellales
Gacutata_Glomerellales
Vdahliae_Hypocreales
Salkalinus_Glomerellales
Pcucumerina_Glomerellales
Lminuta_Diaporthales
Opiliferum_Ophiostomatales
Gclavigera_Ophiostomatales
Dampelina_Diaporthales
Cchrysosperma_Diaporthales
Clustricola_Diaporthales
Cpopulea_Diaporthales
Mpoae_Magnaporthales
Ggraminis_Magnaporthales
Moryzae_Magnaporthales
taxa
20
40
60
80
100
0
1
2
3
4
5
6
7
8
branch length

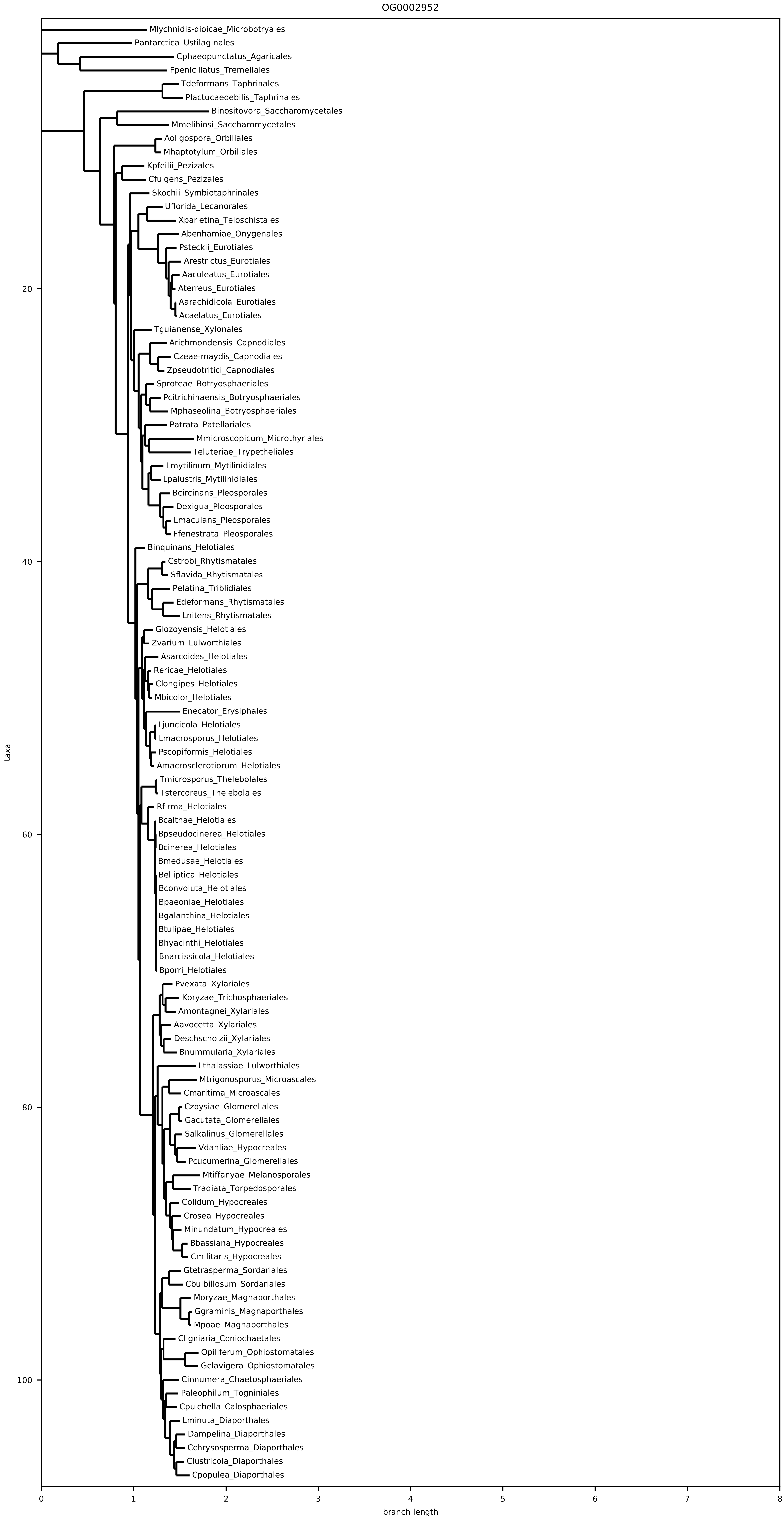
OG0002952
Mlychnidis-dioicae_Microbotryales
Pantarctica_Ustilaginales
Cphaeopunctatus_Agaricales
Fpenicillatus_Tremellales
Tdeformans_Taphrinales
Plactucaedebilis_Taphrinales
Binositovora_Saccharomycetales
Mmelibiosi_Saccharomycetales
Aoligospora_Orbiliales
Mhaptotylum_Orbiliales
Kpfeilii_Pezizales
Cfulgens_Pezizales
Skochii_Symbiotaphrinales
Uflorida_Lecanorales
Xparietina_Teloschistales
Abenhamiae_Onygenales
Psteckii_Eurotiales
Arestrictus_Eurotiales
Aaculeatus_Eurotiales
Aterreus_Eurotiales
Aarachidicola_Eurotiales
Acaelatus_Eurotiales
Tguianense_Xylonales
Arichmondensis_Capnodiales
Czeae-maydis_Capnodiales
Zpseudotritici_Capnodiales
Sproteae_Botryosphaeriales
Pcitrichinaensis_Botryosphaeriales
Mphaseolina_Botryosphaeriales
Patrata_Patellariales
Mmicroscopicum_Microthyriales
Teluteriae_Trypetheliales
Lmytilinum_Mytilinidiales
Lpalustris_Mytilinidiales
Bcircinans_Pleosporales
Dexigua_Pleosporales
Lmaculans_Pleosporales
Ffenestrata_Pleosporales
Binquinans_Helotiales
Cstrobi_Rhytismatales
Sflavida_Rhytismatales
Pelatina_Triblidiales
Edeformans_Rhytismatales
Lnitens_Rhytismatales
Glozoyensis_Helotiales
Zvarium_Lulworthiales
Asarcoides_Helotiales
Rericae_Helotiales
Clongipes_Helotiales
Mbicolor_Helotiales
Enecator_Erysiphales
Ljuncicola_Helotiales
Lmacrosporus_Helotiales
Pscopiformis_Helotiales
Amacrosclerotiorum_Helotiales
Tmicrosporus_Thelebolales
Tstercoreus_Thelebolales
Rfirma_Helotiales
Bcalthae_Helotiales
Bpseudocinerea_Helotiales
Bcinerea_Helotiales
Bmedusae_Helotiales
Belliptica_Helotiales
Bconvoluta_Helotiales
Bpaeoniae_Helotiales
Bgalanthina_Helotiales
Btulipae_Helotiales
Bhyacinthi_Helotiales
Bnarcissicola_Helotiales
Bporri_Helotiales
Pvexata_Xylariales
Koryzae_Trichosphaeriales
Amontagnei_Xylariales
Aavocetta_Xylariales
Deschscholzii_Xylariales
Bnummularia_Xylariales
Lthalassiae_Lulworthiales
Mtrigonosporus_Microascales
Cmaritima_Microascales
Czoysiae_Glomerellales
Gacutata_Glomerellales
Salkalinus_Glomerellales
Vdahliae_Hypocreales
Pcucumerina_Glomerellales
Mtiffanyae_Melanosporales
Tradiata_Torpedosporales
Colidum_Hypocreales
Crosea_Hypocreales
Minundatum_Hypocreales
Bbassiana_Hypocreales
Cmilitaris_Hypocreales
Gtetrasperma_Sordariales
Cbulbillosum_Sordariales
Moryzae_Magnaporthales
Ggraminis_Magnaporthales
Mpoae_Magnaporthales
Cligniaria_Coniochaetales
Opiliferum_Ophiostomatales
Gclavigera_Ophiostomatales
Cinnumera_Chaetosphaeriales
Paleophilum_Togniniales
Cpulchella_Calosphaeriales
Lminuta_Diaporthales
Dampelina_Diaporthales
Cchrysosperma_Diaporthales
Clustricola_Diaporthales
Cpopulea_Diaporthales
taxa
20
40
60
80
100
0
1
2
3
4
5
6
7
8
branch length

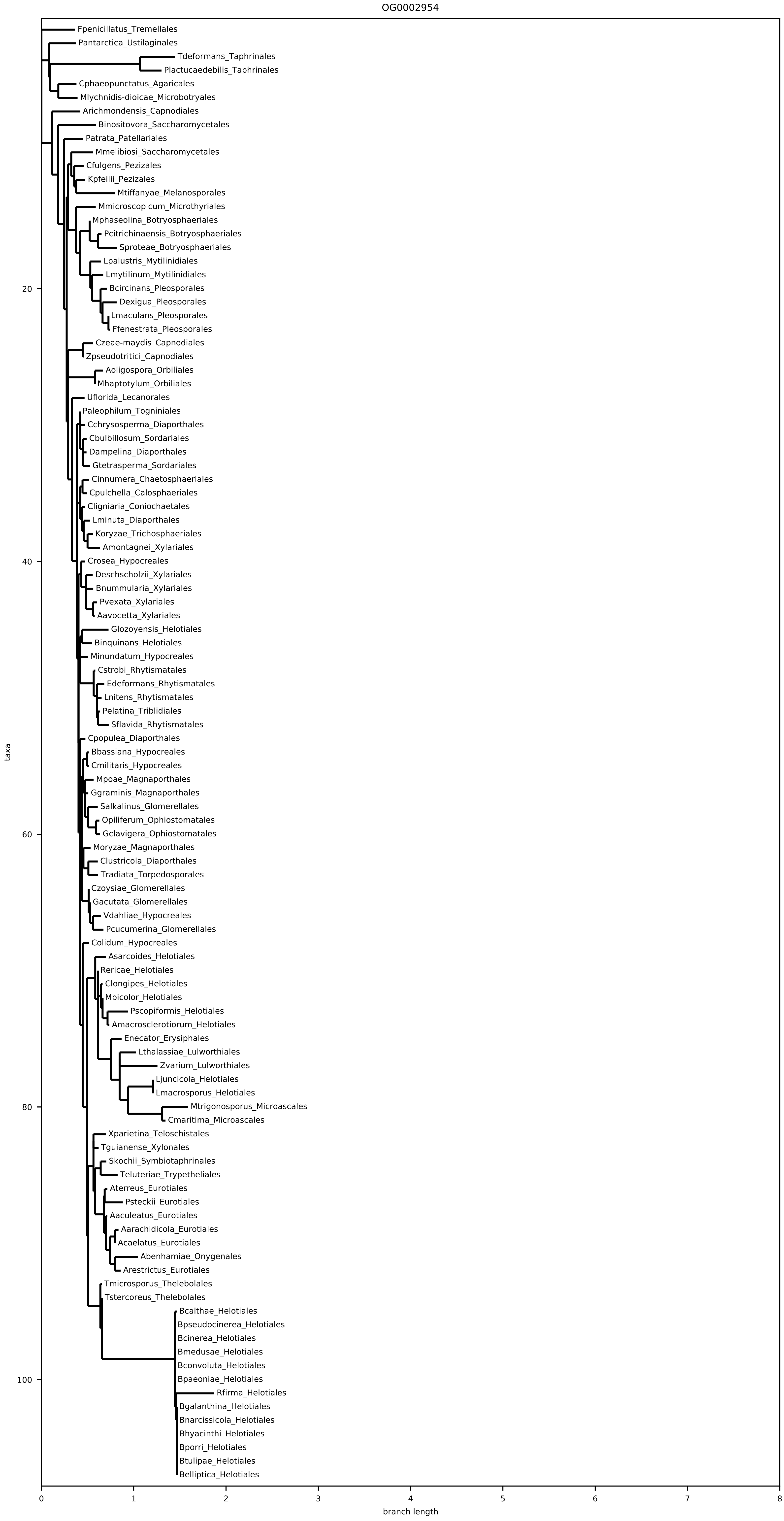

OG0002954
Fpenicillatus_Tremellales
Pantarctica_Ustilaginales
Tdeformans_Taphrinales
Plactucaedebilis_Taphrinales
Cphaeopunctatus_Agaricales
Mlychnidis-dioicae_Microbotryales
Arichmondensis_Capnodiales
Binositovora_Saccharomycetales
Patrata_Patellariales
Mmelibiosi_Saccharomycetales
Cfulgens_Pezizales
Kpfeilii_Pezizales
Mtiffanyae_Melanosporales
Mmicroscopicum_Microthyriales
Mphaseolina_Botryosphaeriales
Pcitrichinaensis_Botryosphaeriales
Sproteae_Botryosphaeriales
Lpalustris_Mytilinidiales
Lmytilinum_Mytilinidiales
Bcircinans_Pleosporales
Dexigua_Pleosporales
Lmaculans_Pleosporales
Ffenestrata_Pleosporales
Czeae-maydis_Capnodiales
Zpseudotritici_Capnodiales
Aoligospora_Orbiliales
Mhaptotylum_Orbiliales
Uflorida_Lecanorales
Paleophilum_Togniniales
Cchrysosperma_Diaporthales
Cbulbillosum_Sordariales
Dampelina_Diaporthales
Gtetrasperma_Sordariales
Cinnumera_Chaetosphaeriales
Cpulchella_Calosphaeriales
Cligniaria_Coniochaetales
Lminuta_Diaporthales
Koryzae_Trichosphaeriales
Amontagnei_Xylariales
Crosea_Hypocreales
Deschscholzii_Xylariales
Bnummularia_Xylariales
Pvexata_Xylariales
Aavocetta_Xylariales
Glozoyensis_Helotiales
Binquinans_Helotiales
Minundatum_Hypocreales
Cstrobi_Rhytismatales
Edeformans_Rhytismatales
Lnitens_Rhytismatales
Pelatina_Triblidiales
Sflavida_Rhytismatales
Cpopulea_Diaporthales
Bbassiana_Hypocreales
Cmilitaris_Hypocreales
Mpoae_Magnaporthales
Ggraminis_Magnaporthales
Salkalinus_Glomerellales
Opiliferum_Ophiostomatales
Gclavigera_Ophiostomatales
Moryzae_Magnaporthales
Clustricola_Diaporthales
Tradiata_Torpedosporales
Czoysiae_Glomerellales
Gacutata_Glomerellales
Vdahliae_Hypocreales
Pcucumerina_Glomerellales
Colidum_Hypocreales
Asarcoides_Helotiales
Rericae_Helotiales
Clongipes_Helotiales
Mbicolor_Helotiales
Pscopiformis_Helotiales
Amacrosclerotiorum_Helotiales
Enecator_Erysiphales
Lthalassiae_Lulworthiales
Zvarium_Lulworthiales
Ljuncicola_Helotiales
Lmacrosporus_Helotiales
Mtrigonosporus_Microascales
Cmaritima_Microascales
Xparietina_Teloschistales
Tguianense_Xylonales
Skochii_Symbiotaphrinales
Teluteriae_Trypetheliales
Aterreus_Eurotiales
Psteckii_Eurotiales
Aaculeatus_Eurotiales
Aarachidicola_Eurotiales
Acaelatus_Eurotiales
Abenhamiae_Onygenales
Arestrictus_Eurotiales
Tmicrosporus_Thelebolales
Tstercoreus_Thelebolales
Bcalthae_Helotiales
Bpseudocinerea_Helotiales
Bcinerea_Helotiales
Bmedusae_Helotiales
Bconvoluta_Helotiales
Bpaeoniae_Helotiales
Rfirma_Helotiales
Bgalanthina_Helotiales
Bnarcissicola_Helotiales
Bhyacinthi_Helotiales
Bporri_Helotiales
Btulipae_Helotiales
Belliptica_Helotiales
taxa
20
40
60
80
100
0
1
2
3
4
5
6
7
8
branch length

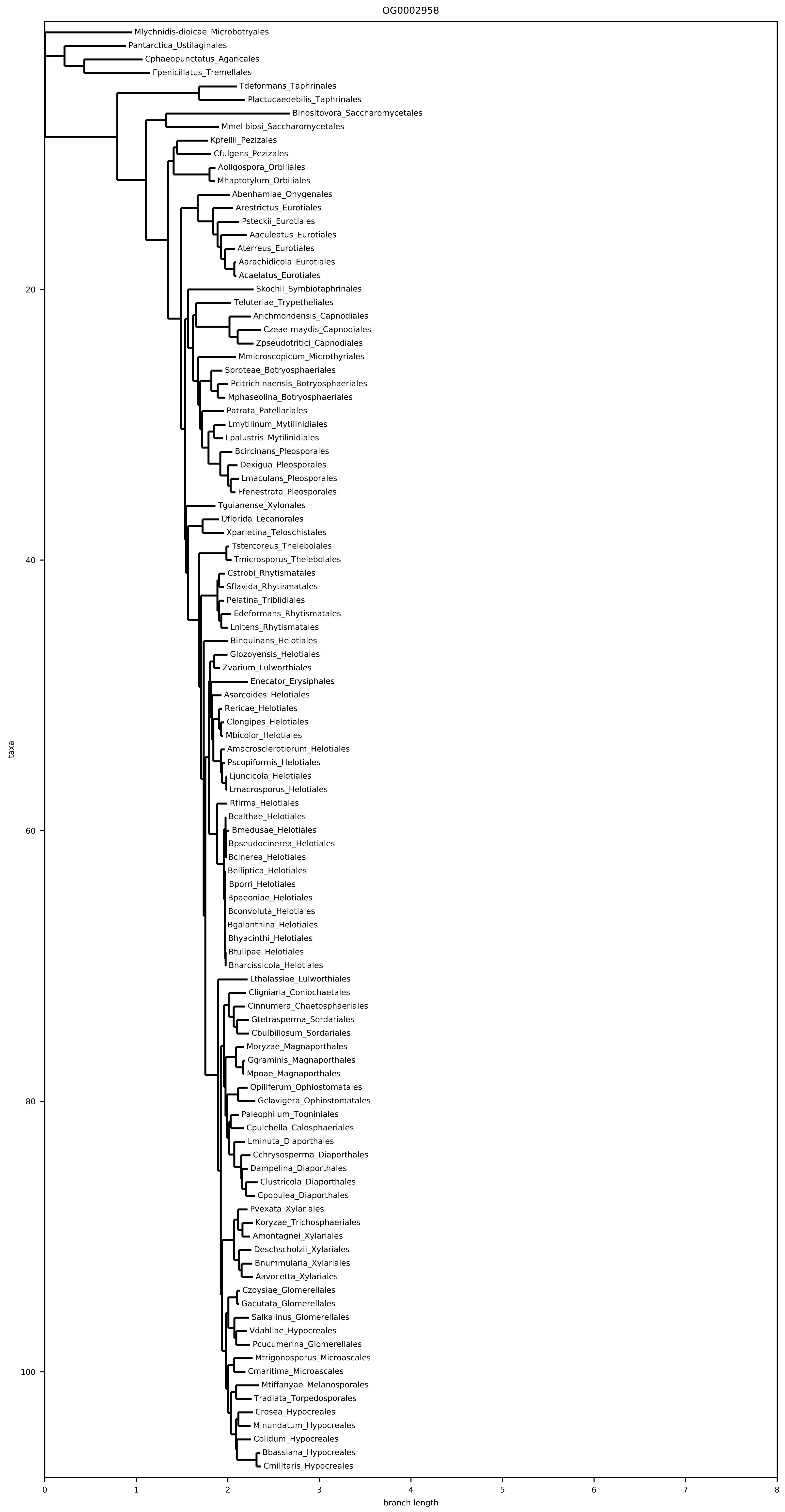
OG0002958
Mlychnidis-dioicae_Microbotryales
Pantarctica_Ustilaginales
Cphaeopunctatus_Agaricales
Fpenicillatus_Tremellales
Tdeformans_Taphrinales
Plactucaedebilis_Taphrinales
Binositovora_Saccharomycetales
Mmelibiosi_Saccharomycetales
Kpfeilii_Pezizales
Cfulgens_Pezizales
Aoligospora_Orbiliales
Mhaptotylum_Orbiliales
Abenhamiae_Onygenales
Arestrictus_Eurotiales
Psteckii_Eurotiales
Aaculeatus_Eurotiales
Aterreus_Eurotiales
Aarachidicola_Eurotiales
Acaelatus_Eurotiales
Skochii_Symbiotaphrinales
Teluteriae_Trypetheliales
Arichmondensis_Capnodiales
Czeae-maydis_Capnodiales
Zpseudotritici_Capnodiales
Mmicroscopicum_Microthyriales
Sproteae_Botryosphaeriales
Pcitrichinaensis_Botryosphaeriales
Mphaseolina_Botryosphaeriales
Patrata_Patellariales
Lmytilinum_Mytilinidiales
Lpalustris_Mytilinidiales
Bcircinans_Pleosporales
Dexigua_Pleosporales
Lmaculans_Pleosporales
Ffenestrata_Pleosporales
Tguianense_Xylonales
Uflorida_Lecanorales
Xparietina_Teloschistales
Tstercoreus_Thelebolales
Tmicrosporus_Thelebolales
Cstrobi_Rhytismatales
Sflavida_Rhytismatales
Pelatina_Triblidiales
Edeformans_Rhytismatales
Lnitens_Rhytismatales
Binquinans_Helotiales
Glozoyensis_Helotiales
Zvarium_Lulworthiales
Enecator_Erysiphales
Asarcoides_Helotiales
Rericae_Helotiales
Clongipes_Helotiales
Mbicolor_Helotiales
Amacrosclerotiorum_Helotiales
Pscopiformis_Helotiales
Ljuncicola_Helotiales
Lmacrosporus_Helotiales
Rfirma_Helotiales
Bcalthae_Helotiales
Bmedusae_Helotiales
Bpseudocinerea_Helotiales
Bcinerea_Helotiales
Belliptica_Helotiales
Bporri_Helotiales
Bpaeoniae_Helotiales
Bconvoluta_Helotiales
Bgalanthina_Helotiales
Bhyacinthi_Helotiales
Btulipae_Helotiales
Bnarcissicola_Helotiales
Lthalassiae_Lulworthiales
Cligniaria_Coniochaetales
Cinnumera_Chaetosphaeriales
Gtetrasperma_Sordariales
Cbulbillosum_Sordariales
Moryzae_Magnaporthales
Ggraminis_Magnaporthales
Mpoae_Magnaporthales
Opiliferum_Ophiostomatales
Gclavigera_Ophiostomatales
Paleophilum_Togniniales
Cpulchella_Calosphaeriales
Lminuta_Diaporthales
Cchrysosperma_Diaporthales
Dampelina_Diaporthales
Clustricola_Diaporthales
Cpopulea_Diaporthales
Pvexata_Xylariales
Koryzae_Trichosphaeriales
Amontagnei_Xylariales
Deschscholzii_Xylariales
Bnummularia_Xylariales
Aavocetta_Xylariales
Czoysiae_Glomerellales
Gacutata_Glomerellales
Salkalinus_Glomerellales
Vdahliae_Hypocreales
Pcucumerina_Glomerellales
Mtrigonosporus_Microascales
Cmaritima_Microascales
Mtiffanyae_Melanosporales
Tradiata_Torpedosporales
Crosea_Hypocreales
Minundatum_Hypocreales
Colidum_Hypocreales
Bbassiana_Hypocreales
Cmilitaris_Hypocreales
taxa
20
40
60
80
100
0
1
2
3
4
5
6
7
8
branch length

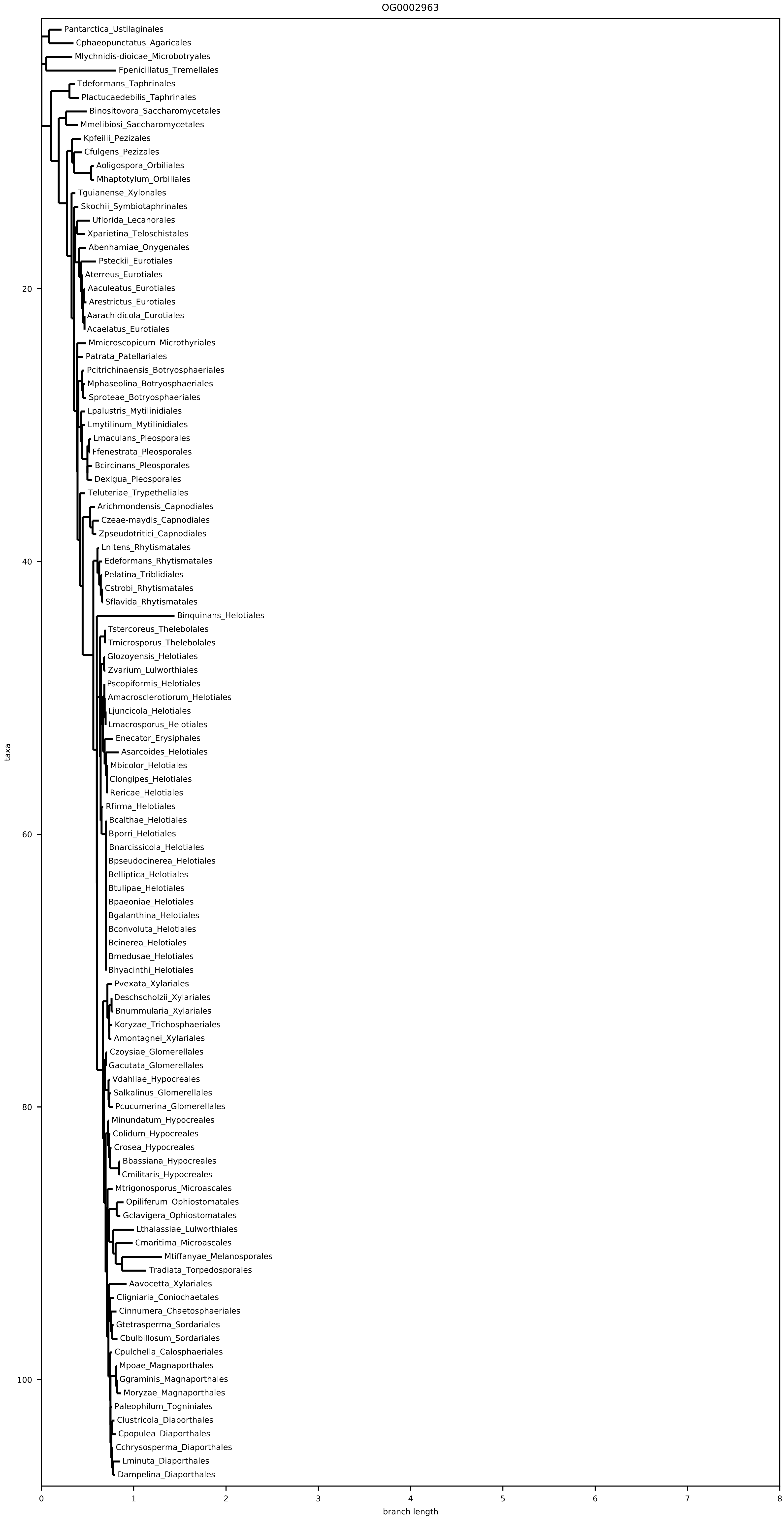

OG0002963
Pantarctica_Ustilaginales
Cphaeopunctatus_Agaricales
Mlychnidis-dioicae_Microbotryales
Fpenicillatus_Tremellales
Tdeformans_Taphrinales
Plactucaedebilis_Taphrinales
Binositovora_Saccharomycetales
Mmelibiosi_Saccharomycetales
Kpfeilii_Pezizales
Cfulgens_Pezizales
Aoligospora_Orbiliales
Mhaptotylum_Orbiliales
Tguianense_Xylonales
Skochii_Symbiotaphrinales
Uflorida_Lecanorales
Xparietina_Teloschistales
Abenhamiae_Onygenales
Psteckii_Eurotiales
Aterreus_Eurotiales
Aaculeatus_Eurotiales
Arestrictus_Eurotiales
Aarachidicola_Eurotiales
Acaelatus_Eurotiales
Mmicroscopicum_Microthyriales
Patrata_Patellariales
Pcitrichinaensis_Botryosphaeriales
Mphaseolina_Botryosphaeriales
Sproteae_Botryosphaeriales
Lpalustris_Mytilinidiales
Lmytilinum_Mytilinidiales
Lmaculans_Pleosporales
Ffenestrata_Pleosporales
Bcircinans_Pleosporales
Dexigua_Pleosporales
Teluteriae_Trypetheliales
Arichmondensis_Capnodiales
Czeae-maydis_Capnodiales
Zpseudotritici_Capnodiales
Lnitens_Rhytismatales
Edeformans_Rhytismatales
Pelatina_Triblidiales
Cstrobi_Rhytismatales
Sflavida_Rhytismatales
Binquinans_Helotiales
Tstercoreus_Thelebolales
Tmicrosporus_Thelebolales
Glozoyensis_Helotiales
Zvarium_Lulworthiales
Pscopiformis_Helotiales
Amacrosclerotiorum_Helotiales
Ljuncicola_Helotiales
Lmacrosporus_Helotiales
Enecator_Erysiphales
Asarcoides_Helotiales
Mbicolor_Helotiales
Clongipes_Helotiales
Rericae_Helotiales
Rfirma_Helotiales
Bcalthae_Helotiales
Bporri_Helotiales
Bnarcissicola_Helotiales
Bpseudocinerea_Helotiales
Belliptica_Helotiales
Btulipae_Helotiales
Bpaeoniae_Helotiales
Bgalanthina_Helotiales
Bconvoluta_Helotiales
Bcinerea_Helotiales
Bmedusae_Helotiales
Bhyacinthi_Helotiales
Pvexata_Xylariales
Deschscholzii_Xylariales
Bnummularia_Xylariales
Koryzae_Trichosphaeriales
Amontagnei_Xylariales
Czoysiae_Glomerellales
Gacutata_Glomerellales
Vdahliae_Hypocreales
Salkalinus_Glomerellales
Pcucumerina_Glomerellales
Minundatum_Hypocreales
Colidum_Hypocreales
Crosea_Hypocreales
Bbassiana_Hypocreales
Cmilitaris_Hypocreales
Mtrigonosporus_Microascales
Opiliferum_Ophiostomatales
Gclavigera_Ophiostomatales
Lthalassiae_Lulworthiales
Cmaritima_Microascales
Mtiffanyae_Melanosporales
Tradiata_Torpedosporales
Aavocetta_Xylariales
Cligniaria_Coniochaetales
Cinnumera_Chaetosphaeriales
Gtetrasperma_Sordariales
Cbulbillosum_Sordariales
Cpulchella_Calosphaeriales
Mpoae_Magnaporthales
Ggraminis_Magnaporthales
Moryzae_Magnaporthales
Paleophilum_Togniniales
Clustricola_Diaporthales
Cpopulea_Diaporthales
Cchrysosperma_Diaporthales
Lminuta_Diaporthales
Dampelina_Diaporthales
taxa
20
40
60
80
100
0
1
2
3
4
5
6
7
8
branch length

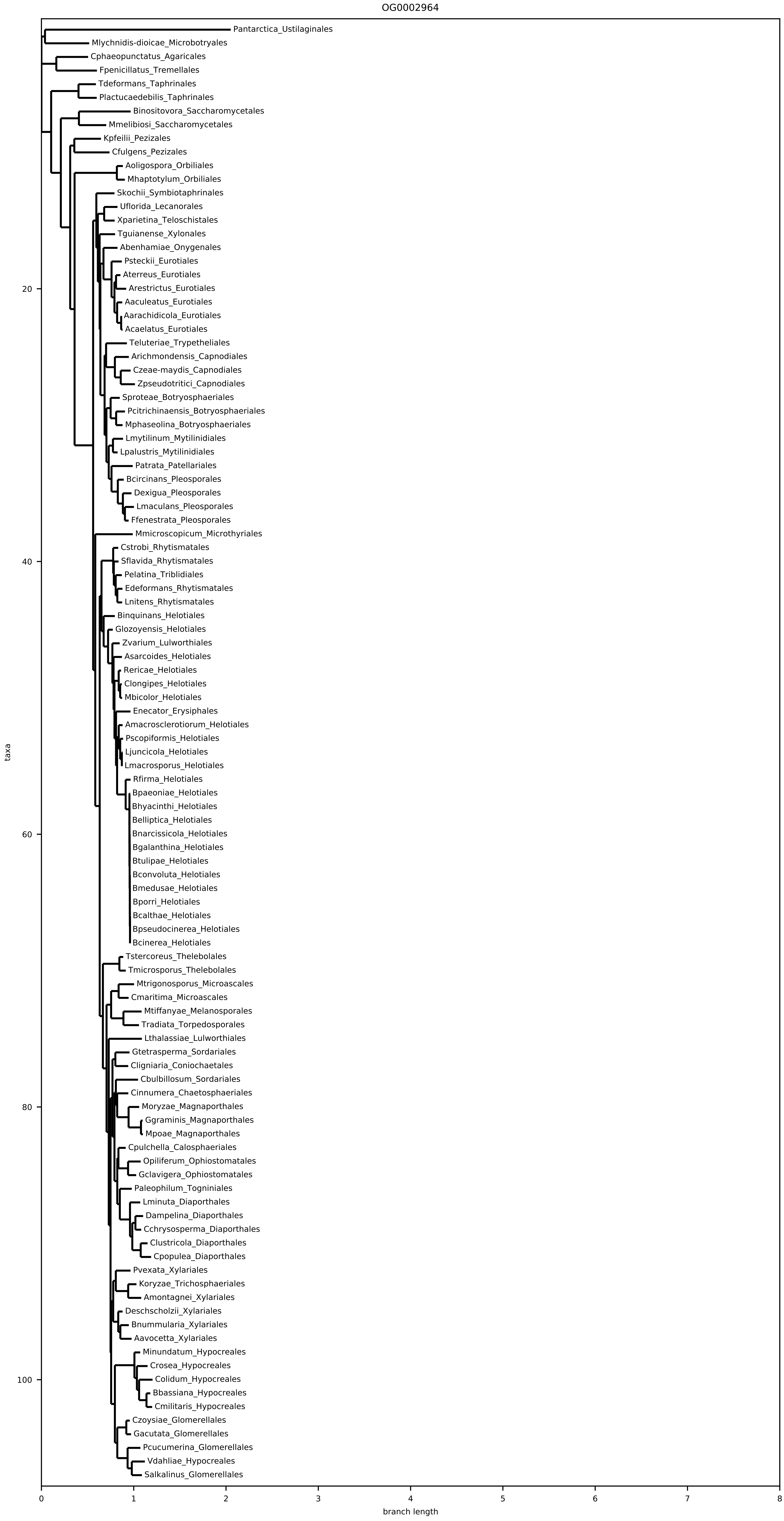
OG0002964
Pantarctica_Ustilaginales
Mlychnidis-dioicae_Microbotryales
Cphaeopunctatus_Agaricales
Fpenicillatus_Tremellales
Tdeformans_Taphrinales
Plactucaedebilis_Taphrinales
Binositovora_Saccharomycetales
Mmelibiosi_Saccharomycetales
Kpfeilii_Pezizales
Cfulgens_Pezizales
Aoligospora_Orbiliales
Mhaptotylum_Orbiliales
Skochii_Symbiotaphrinales
Uflorida_Lecanorales
Xparietina_Teloschistales
Tguianense_Xylonales
Abenhamiae_Onygenales
Psteckii_Eurotiales
Aterreus_Eurotiales
Arestrictus_Eurotiales
Aaculeatus_Eurotiales
Aarachidicola_Eurotiales
Acaelatus_Eurotiales
Teluteriae_Trypetheliales
Arichmondensis_Capnodiales
Czeae-maydis_Capnodiales
Zpseudotritici_Capnodiales
Sproteae_Botryosphaeriales
Pcitrichinaensis_Botryosphaeriales
Mphaseolina_Botryosphaeriales
Lmytilinum_Mytilinidiales
Lpalustris_Mytilinidiales
Patrata_Patellariales
Bcircinans_Pleosporales
Dexigua_Pleosporales
Lmaculans_Pleosporales
Ffenestrata_Pleosporales
Mmicroscopicum_Microthyriales
Cstrobi_Rhytismatales
Sflavida_Rhytismatales
Pelatina_Triblidiales
Edeformans_Rhytismatales
Lnitens_Rhytismatales
Binquinans_Helotiales
Glozoyensis_Helotiales
Zvarium_Lulworthiales
Asarcoides_Helotiales
Rericae_Helotiales
Clongipes_Helotiales
Mbicolor_Helotiales
Enecator_Erysiphales
Amacrosclerotiorum_Helotiales
Pscopiformis_Helotiales
Ljuncicola_Helotiales
Lmacrosporus_Helotiales
Rfirma_Helotiales
Bpaeoniae_Helotiales
Bhyacinthi_Helotiales
Belliptica_Helotiales
Bnarcissicola_Helotiales
Bgalanthina_Helotiales
Btulipae_Helotiales
Bconvoluta_Helotiales
Bmedusae_Helotiales
Bporri_Helotiales
Bcalthae_Helotiales
Bpseudocinerea_Helotiales
Bcinerea_Helotiales
Tstercoreus_Thelebolales
Tmicrosporus_Thelebolales
Mtrigonosporus_Microascales
Cmaritima_Microascales
Mtiffanyae_Melanosporales
Tradiata_Torpedosporales
Lthalassiae_Lulworthiales
Gtetrasperma_Sordariales
Cligniaria_Coniochaetales
Cbulbillosum_Sordariales
Cinnumera_Chaetosphaeriales
Moryzae_Magnaporthales
Ggraminis_Magnaporthales
Mpoae_Magnaporthales
Cpulchella_Calosphaeriales
Opiliferum_Ophiostomatales
Gclavigera_Ophiostomatales
Paleophilum_Togniniales
Lminuta_Diaporthales
Dampelina_Diaporthales
Cchrysosperma_Diaporthales
Clustricola_Diaporthales
Cpopulea_Diaporthales
Pvexata_Xylariales
Koryzae_Trichosphaeriales
Amontagnei_Xylariales
Deschscholzii_Xylariales
Bnummularia_Xylariales
Aavocetta_Xylariales
Minundatum_Hypocreales
Crosea_Hypocreales
Colidum_Hypocreales
Bbassiana_Hypocreales
Cmilitaris_Hypocreales
Czoysiae_Glomerellales
Gacutata_Glomerellales
Pcucumerina_Glomerellales
Vdahliae_Hypocreales
Salkalinus_Glomerellales
taxa
20
40
60
80
100
0
1
2
3
4
5
6
7
8
branch length

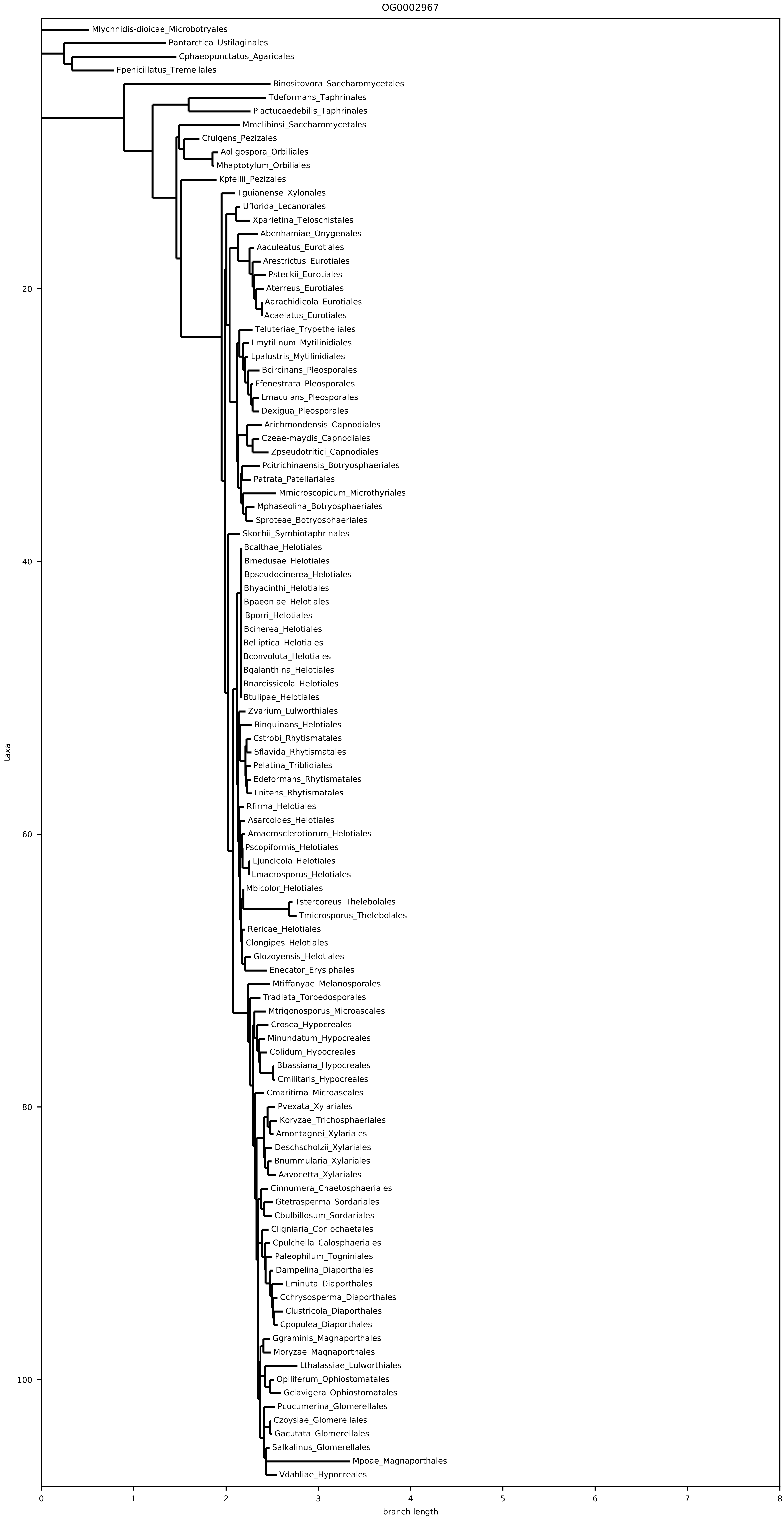
OG0002967
Mlychnidis-dioicae_Microbotryales
Pantarctica_Ustilaginales
Cphaeopunctatus_Agaricales
Fpenicillatus_Tremellales
Binositovora_Saccharomycetales
Tdeformans_Taphrinales
Plactucaedebilis_Taphrinales
Mmelibiosi_Saccharomycetales
Cfulgens_Pezizales
Aoligospora_Orbiliales
Mhaptotylum_Orbiliales
Kpfeilii_Pezizales
Tguianense_Xylonales
Uflorida_Lecanorales
Xparietina_Teloschistales
Abenhamiae_Onygenales
Aaculeatus_Eurotiales
Arestrictus_Eurotiales
Psteckii_Eurotiales
Aterreus_Eurotiales
Aarachidicola_Eurotiales
Acaelatus_Eurotiales
Teluteriae_Trypetheliales
Lmytilinum_Mytilinidiales
Lpalustris_Mytilinidiales
Bcircinans_Pleosporales
Ffenestrata_Pleosporales
Lmaculans_Pleosporales
Dexigua_Pleosporales
Arichmondensis_Capnodiales
Czeae-maydis_Capnodiales
Zpseudotritici_Capnodiales
Pcitrichinaensis_Botryosphaeriales
Patrata_Patellariales
Mmicroscopicum_Microthyriales
Mphaseolina_Botryosphaeriales
Sproteae_Botryosphaeriales
Skochii_Symbiotaphrinales
Bcalthae_Helotiales
Bmedusae_Helotiales
Bpseudocinerea_Helotiales
Bhyacinthi_Helotiales
Bpaeoniae_Helotiales
Bporri_Helotiales
Bcinerea_Helotiales
Belliptica_Helotiales
Bconvoluta_Helotiales
Bgalanthina_Helotiales
Bnarcissicola_Helotiales
Btulipae_Helotiales
Zvarium_Lulworthiales
Binquinans_Helotiales
Cstrobi_Rhytismatales
Sflavida_Rhytismatales
Pelatina_Triblidiales
Edeformans_Rhytismatales
Lnitens_Rhytismatales
Rfirma_Helotiales
Asarcoides_Helotiales
Amacrosclerotiorum_Helotiales
Pscopiformis_Helotiales
Ljuncicola_Helotiales
Lmacrosporus_Helotiales
Mbicolor_Helotiales
Tstercoreus_Thelebolales
Tmicrosporus_Thelebolales
Rericae_Helotiales
Clongipes_Helotiales
Glozoyensis_Helotiales
Enecator_Erysiphales
Mtiffanyae_Melanosporales
Tradiata_Torpedosporales
Mtrigonosporus_Microascales
Crosea_Hypocreales
Minundatum_Hypocreales
Colidum_Hypocreales
Bbassiana_Hypocreales
Cmilitaris_Hypocreales
Cmaritima_Microascales
Pvexata_Xylariales
Koryzae_Trichosphaeriales
Amontagnei_Xylariales
Deschscholzii_Xylariales
Bnummularia_Xylariales
Aavocetta_Xylariales
Cinnumera_Chaetosphaeriales
Gtetrasperma_Sordariales
Cbulbillosum_Sordariales
Cligniaria_Coniochaetales
Cpulchella_Calosphaeriales
Paleophilum_Togniniales
Dampelina_Diaporthales
Lminuta_Diaporthales
Cchrysosperma_Diaporthales
Clustricola_Diaporthales
Cpopulea_Diaporthales
Ggraminis_Magnaporthales
Moryzae_Magnaporthales
Lthalassiae_Lulworthiales
Opiliferum_Ophiostomatales
Gclavigera_Ophiostomatales
Pcucumerina_Glomerellales
Czoysiae_Glomerellales
Gacutata_Glomerellales
Salkalinus_Glomerellales
Mpoae_Magnaporthales
Vdahliae_Hypocreales
taxa
20
40
60
80
100
0
1
2
3
4
5
6
7
8
branch length

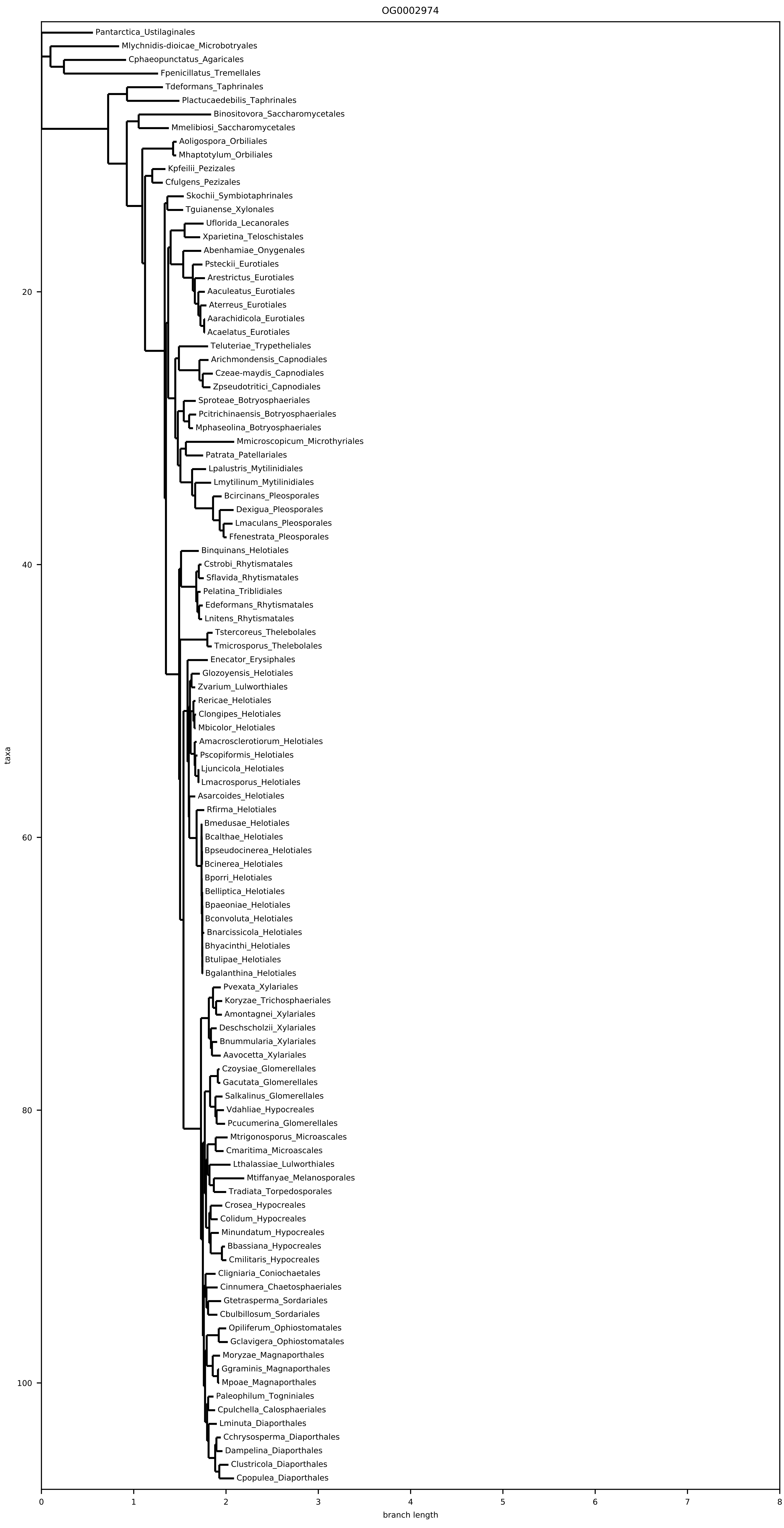
OG0002974
Pantarctica_Ustilaginales
Mlychnidis-dioicae_Microbotryales
Cphaeopunctatus_Agaricales
Fpenicillatus_Tremellales
Tdeformans_Taphrinales
Plactucaedebilis_Taphrinales
Binositovora_Saccharomycetales
Mmelibiosi_Saccharomycetales
Aoligospora_Orbiliales
Mhaptotylum_Orbiliales
Kpfeilii_Pezizales
Cfulgens_Pezizales
Skochii_Symbiotaphrinales
Tguianense_Xylonales
Uflorida_Lecanorales
Xparietina_Teloschistales
Abenhamiae_Onygenales
Psteckii_Eurotiales
Arestrictus_Eurotiales
Aaculeatus_Eurotiales
Aterreus_Eurotiales
Aarachidicola_Eurotiales
Acaelatus_Eurotiales
Teluteriae_Trypetheliales
Arichmondensis_Capnodiales
Czeae-maydis_Capnodiales
Zpseudotritici_Capnodiales
Sproteae_Botryosphaeriales
Pcitrichinaensis_Botryosphaeriales
Mphaseolina_Botryosphaeriales
Mmicroscopicum_Microthyriales
Patrata_Patellariales
Lpalustris_Mytilinidiales
Lmytilinum_Mytilinidiales
Bcircinans_Pleosporales
Dexigua_Pleosporales
Lmaculans_Pleosporales
Ffenestrata_Pleosporales
Binquinans_Helotiales
Cstrobi_Rhytismatales
Sflavida_Rhytismatales
Pelatina_Triblidiales
Edeformans_Rhytismatales
Lnitens_Rhytismatales
Tstercoreus_Thelebolales
Tmicrosporus_Thelebolales
Enecator_Erysiphales
Glozoyensis_Helotiales
Zvarium_Lulworthiales
Rericae_Helotiales
Clongipes_Helotiales
Mbicolor_Helotiales
Amacrosclerotiorum_Helotiales
Pscopiformis_Helotiales
Ljuncicola_Helotiales
Lmacrosporus_Helotiales
Asarcoides_Helotiales
Rfirma_Helotiales
Bmedusae_Helotiales
Bcalthae_Helotiales
Bpseudocinerea_Helotiales
Bcinerea_Helotiales
Bporri_Helotiales
Belliptica_Helotiales
Bpaeoniae_Helotiales
Bconvoluta_Helotiales
Bnarcissicola_Helotiales
Bhyacinthi_Helotiales
Btulipae_Helotiales
Bgalanthina_Helotiales
Pvexata_Xylariales
Koryzae_Trichosphaeriales
Amontagnei_Xylariales
Deschscholzii_Xylariales
Bnummularia_Xylariales
Aavocetta_Xylariales
Czoysiae_Glomerellales
Gacutata_Glomerellales
Salkalinus_Glomerellales
Vdahliae_Hypocreales
Pcucumerina_Glomerellales
Mtrigonosporus_Microascales
Cmaritima_Microascales
Lthalassiae_Lulworthiales
Mtiffanyae_Melanosporales
Tradiata_Torpedosporales
Crosea_Hypocreales
Colidum_Hypocreales
Minundatum_Hypocreales
Bbassiana_Hypocreales
Cmilitaris_Hypocreales
Cligniaria_Coniochaetales
Cinnumera_Chaetosphaeriales
Gtetrasperma_Sordariales
Cbulbillosum_Sordariales
Opiliferum_Ophiostomatales
Gclavigera_Ophiostomatales
Moryzae_Magnaporthales
Ggraminis_Magnaporthales
Mpoae_Magnaporthales
Paleophilum_Togniniales
Cpulchella_Calosphaeriales
Lminuta_Diaporthales
Cchrysosperma_Diaporthales
Dampelina_Diaporthales
Clustricola_Diaporthales
Cpopulea_Diaporthales
taxa
20
40
60
80
100
0
1
2
3
4
5
6
7
8
branch length

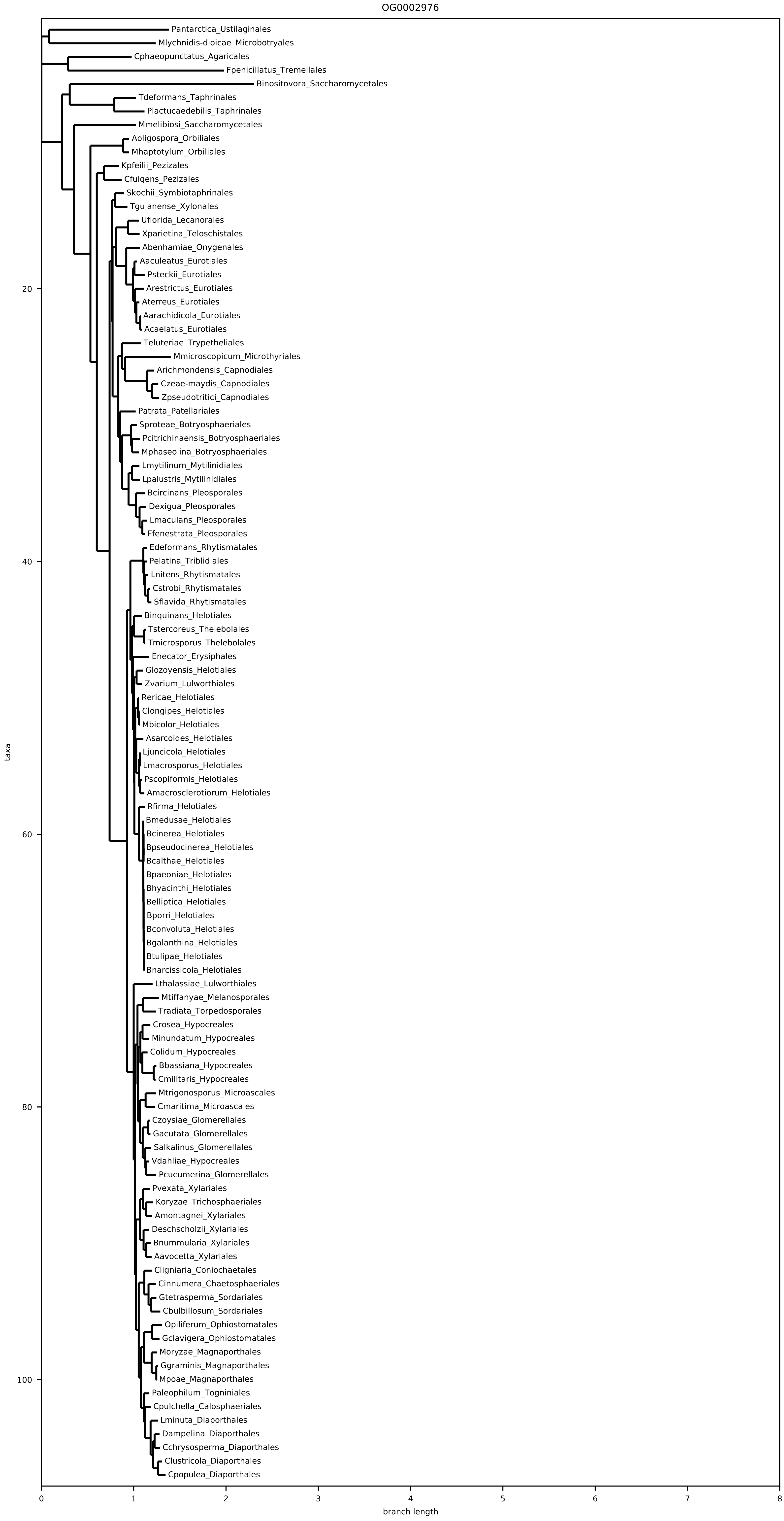

OG0002976
Pantarctica_Ustilaginales
Mlychnidis-dioicae_Microbotryales
Cphaeopunctatus_Agaricales
Fpenicillatus_Tremellales
Binositovora_Saccharomycetales
Tdeformans_Taphrinales
Plactucaedebilis_Taphrinales
Mmelibiosi_Saccharomycetales
Aoligospora_Orbiliales
Mhaptotylum_Orbiliales
Kpfeilii_Pezizales
Cfulgens_Pezizales
Skochii_Symbiotaphrinales
Tguianense_Xylonales
Uflorida_Lecanorales
Xparietina_Teloschistales
Abenhamiae_Onygenales
Aaculeatus_Eurotiales
Psteckii_Eurotiales
Arestrictus_Eurotiales
Aterreus_Eurotiales
Aarachidicola_Eurotiales
Acaelatus_Eurotiales
Teluteriae_Trypetheliales
Mmicroscopicum_Microthyriales
Arichmondensis_Capnodiales
Czeae-maydis_Capnodiales
Zpseudotritici_Capnodiales
Patrata_Patellariales
Sproteae_Botryosphaeriales
Pcitrichinaensis_Botryosphaeriales
Mphaseolina_Botryosphaeriales
Lmytilinum_Mytilinidiales
Lpalustris_Mytilinidiales
Bcircinans_Pleosporales
Dexigua_Pleosporales
Lmaculans_Pleosporales
Ffenestrata_Pleosporales
Edeformans_Rhytismatales
Pelatina_Triblidiales
Lnitens_Rhytismatales
Cstrobi_Rhytismatales
Sflavida_Rhytismatales
Binquinans_Helotiales
Tstercoreus_Thelebolales
Tmicrosporus_Thelebolales
Enecator_Erysiphales
Glozoyensis_Helotiales
Zvarium_Lulworthiales
Rericae_Helotiales
Clongipes_Helotiales
Mbicolor_Helotiales
Asarcoides_Helotiales
Ljuncicola_Helotiales
Lmacrosporus_Helotiales
Pscopiformis_Helotiales
Amacrosclerotiorum_Helotiales
Rfirma_Helotiales
Bmedusae_Helotiales
Bcinerea_Helotiales
Bpseudocinerea_Helotiales
Bcalthae_Helotiales
Bpaeoniae_Helotiales
Bhyacinthi_Helotiales
Belliptica_Helotiales
Bporri_Helotiales
Bconvoluta_Helotiales
Bgalanthina_Helotiales
Btulipae_Helotiales
Bnarcissicola_Helotiales
Lthalassiae_Lulworthiales
Mtiffanyae_Melanosporales
Tradiata_Torpedosporales
Crosea_Hypocreales
Minundatum_Hypocreales
Colidum_Hypocreales
Bbassiana_Hypocreales
Cmilitaris_Hypocreales
Mtrigonosporus_Microascales
Cmaritima_Microascales
Czoysiae_Glomerellales
Gacutata_Glomerellales
Salkalinus_Glomerellales
Vdahliae_Hypocreales
Pcucumerina_Glomerellales
Pvexata_Xylariales
Koryzae_Trichosphaeriales
Amontagnei_Xylariales
Deschscholzii_Xylariales
Bnummularia_Xylariales
Aavocetta_Xylariales
Cligniaria_Coniochaetales
Cinnumera_Chaetosphaeriales
Gtetrasperma_Sordariales
Cbulbillosum_Sordariales
Opiliferum_Ophiostomatales
Gclavigera_Ophiostomatales
Moryzae_Magnaporthales
Ggraminis_Magnaporthales
Mpoae_Magnaporthales
Paleophilum_Togniniales
Cpulchella_Calosphaeriales
Lminuta_Diaporthales
Dampelina_Diaporthales
Cchrysosperma_Diaporthales
Clustricola_Diaporthales
Cpopulea_Diaporthales
taxa
20
40
60
80
100
0
1
2
3
4
5
6
7
8
branch length

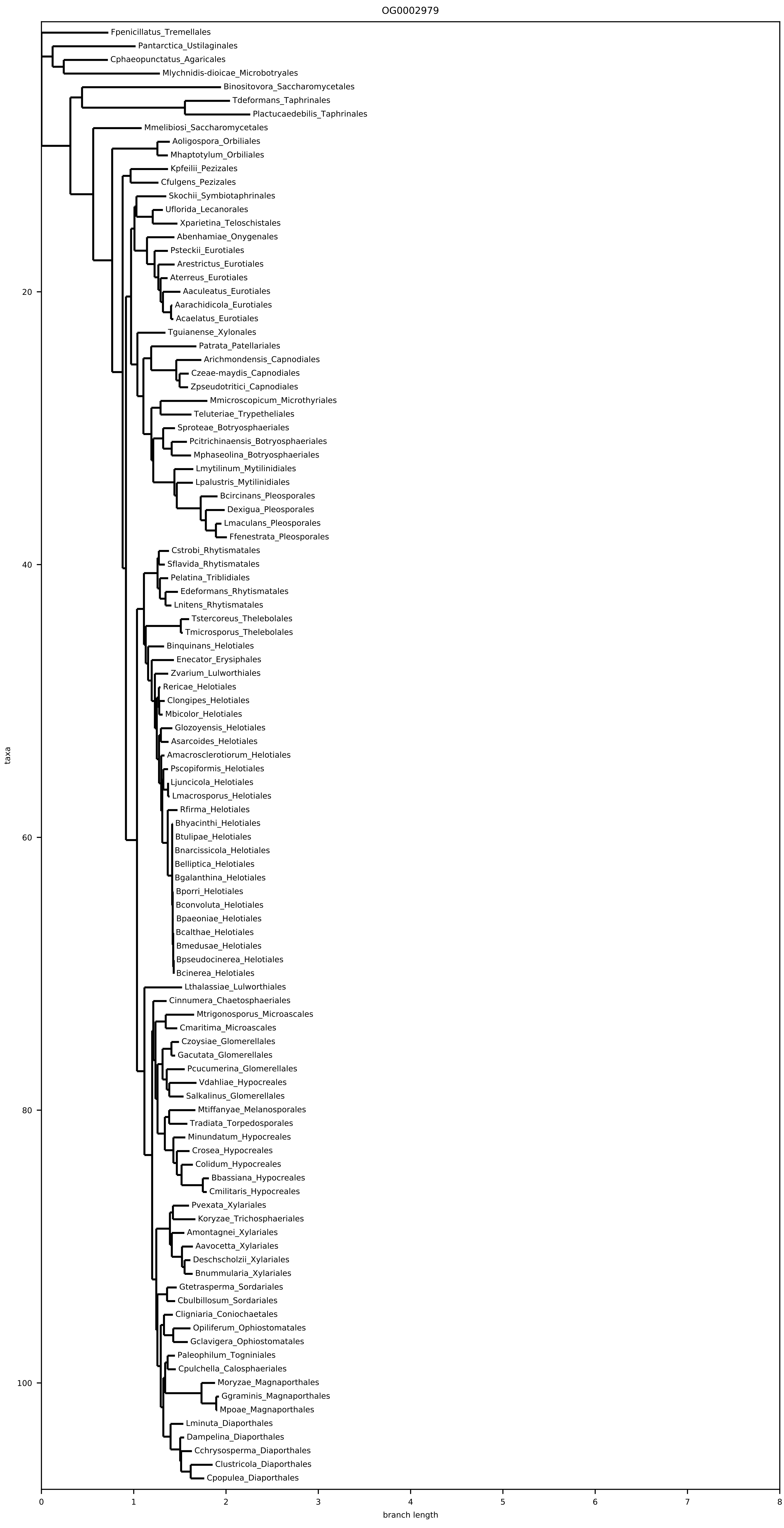

OG0002979
Fpenicillatus_Tremellales
Pantarctica_Ustilaginales
Cphaeopunctatus_Agaricales
Mlychnidis-dioicae_Microbotryales
Binositovora_Saccharomycetales
Tdeformans_Taphrinales
Plactucaedebilis_Taphrinales
Mmelibiosi_Saccharomycetales
Aoligospora_Orbiliales
Mhaptotylum_Orbiliales
Kpfeilii_Pezizales
Cfulgens_Pezizales
Skochii_Symbiotaphrinales
Uflorida_Lecanorales
Xparietina_Teloschistales
Abenhamiae_Onygenales
Psteckii_Eurotiales
Arestrictus_Eurotiales
Aterreus_Eurotiales
Aaculeatus_Eurotiales
Aarachidicola_Eurotiales
Acaelatus_Eurotiales
Tguianense_Xylonales
Patrata_Patellariales
Arichmondensis_Capnodiales
Czeae-maydis_Capnodiales
Zpseudotritici_Capnodiales
Mmicroscopicum_Microthyriales
Teluteriae_Trypetheliales
Sproteae_Botryosphaeriales
Pcitrichinaensis_Botryosphaeriales
Mphaseolina_Botryosphaeriales
Lmytilinum_Mytilinidiales
Lpalustris_Mytilinidiales
Bcircinans_Pleosporales
Dexigua_Pleosporales
Lmaculans_Pleosporales
Ffenestrata_Pleosporales
Cstrobi_Rhytismatales
Sflavida_Rhytismatales
Pelatina_Triblidiales
Edeformans_Rhytismatales
Lnitens_Rhytismatales
Tstercoreus_Thelebolales
Tmicrosporus_Thelebolales
Binquinans_Helotiales
Enecator_Erysiphales
Zvarium_Lulworthiales
Rericae_Helotiales
Clongipes_Helotiales
Mbicolor_Helotiales
Glozoyensis_Helotiales
Asarcoides_Helotiales
Amacrosclerotiorum_Helotiales
Pscopiformis_Helotiales
Ljuncicola_Helotiales
Lmacrosporus_Helotiales
Rfirma_Helotiales
Bhyacinthi_Helotiales
Btulipae_Helotiales
Bnarcissicola_Helotiales
Belliptica_Helotiales
Bgalanthina_Helotiales
Bporri_Helotiales
Bconvoluta_Helotiales
Bpaeoniae_Helotiales
Bcalthae_Helotiales
Bmedusae_Helotiales
Bpseudocinerea_Helotiales
Bcinerea_Helotiales
Lthalassiae_Lulworthiales
Cinnumera_Chaetosphaeriales
Mtrigonosporus_Microascales
Cmaritima_Microascales
Czoysiae_Glomerellales
Gacutata_Glomerellales
Pcucumerina_Glomerellales
Vdahliae_Hypocreales
Salkalinus_Glomerellales
Mtiffanyae_Melanosporales
Tradiata_Torpedosporales
Minundatum_Hypocreales
Crosea_Hypocreales
Colidum_Hypocreales
Bbassiana_Hypocreales
Cmilitaris_Hypocreales
Pvexata_Xylariales
Koryzae_Trichosphaeriales
Amontagnei_Xylariales
Aavocetta_Xylariales
Deschscholzii_Xylariales
Bnummularia_Xylariales
Gtetrasperma_Sordariales
Cbulbillosum_Sordariales
Cligniaria_Coniochaetales
Opiliferum_Ophiostomatales
Gclavigera_Ophiostomatales
Paleophilum_Togniniales
Cpulchella_Calosphaeriales
Moryzae_Magnaporthales
Ggraminis_Magnaporthales
Mpoae_Magnaporthales
Lminuta_Diaporthales
Dampelina_Diaporthales
Cchrysosperma_Diaporthales
Clustricola_Diaporthales
Cpopulea_Diaporthales
taxa
20
40
60
80
100
0
1
2
3
4
5
6
7
8
branch length

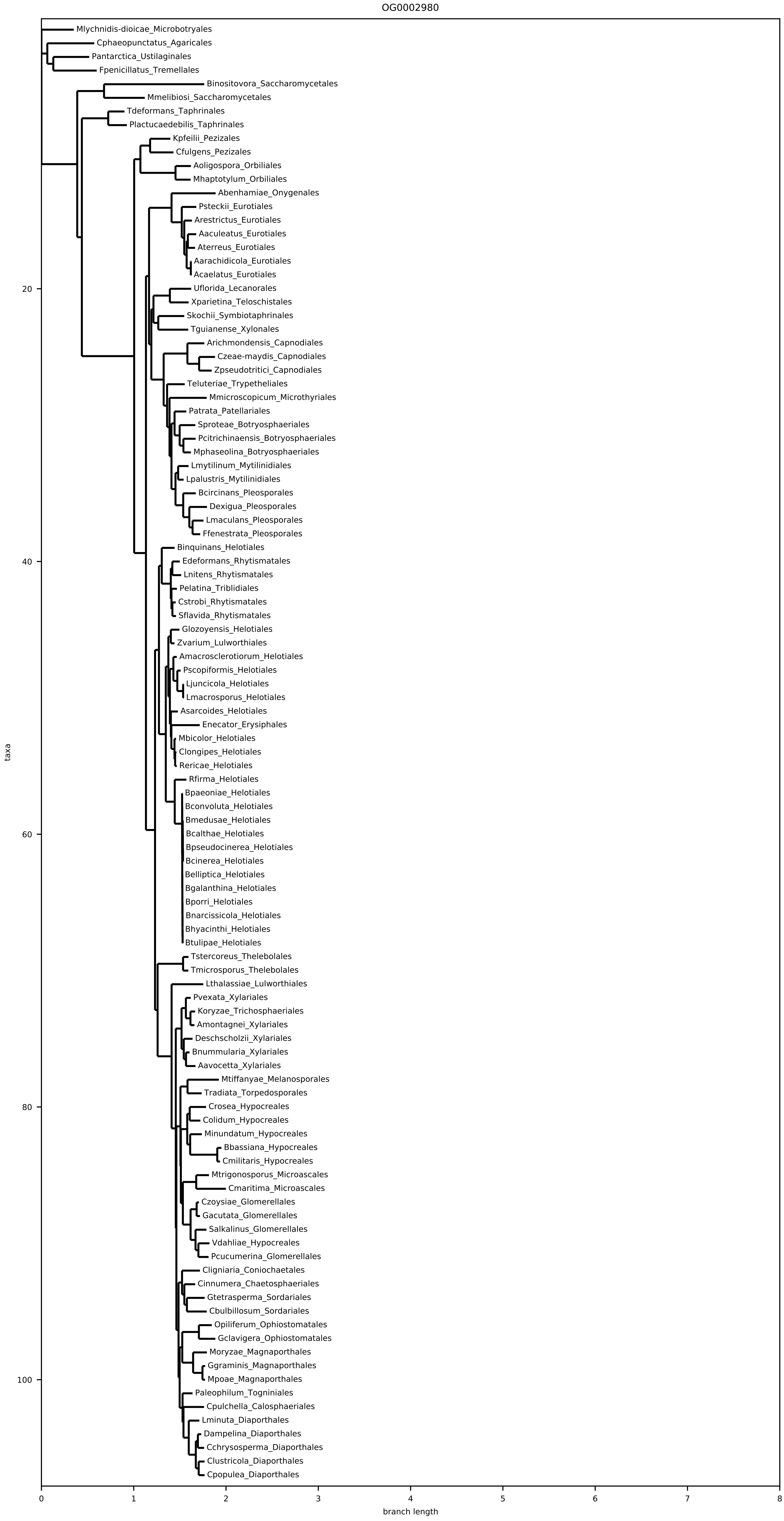
OG0002980
Mlychnidis-dioicae_Microbotryales
Cphaeopunctatus_Agaricales
Pantarctica_Ustilaginales
Fpenicillatus_Tremellales
Binositovora_Saccharomycetales
Mmelibiosi_Saccharomycetales
Tdeformans_Taphrinales
Plactucaedebilis_Taphrinales
Kpfeilii_Pezizales
Cfulgens_Pezizales
Aoligospora_Orbiliales
Mhaptotylum_Orbiliales
Abenhamiae_Onygenales
Psteckii_Eurotiales
Arestrictus_Eurotiales
Aaculeatus_Eurotiales
Aterreus_Eurotiales
Aarachidicola_Eurotiales
Acaelatus_Eurotiales
Uflorida_Lecanorales
Xparietina_Teloschistales
Skochii_Symbiotaphrinales
Tguianense_Xylonales
Arichmondensis_Capnodiales
Czeae-maydis_Capnodiales
Zpseudotritici_Capnodiales
Teluteriae_Trypetheliales
Mmicroscopicum_Microthyriales
Patrata_Patellariales
Sproteae_Botryosphaeriales
Pcitrichinaensis_Botryosphaeriales
Mphaseolina_Botryosphaeriales
Lmytilinum_Mytilinidiales
Lpalustris_Mytilinidiales
Bcircinans_Pleosporales
Dexigua_Pleosporales
Lmaculans_Pleosporales
Ffenestrata_Pleosporales
Binquinans_Helotiales
Edeformans_Rhytismatales
Lnitens_Rhytismatales
Pelatina_Triblidiales
Cstrobi_Rhytismatales
Sflavida_Rhytismatales
Glozoyensis_Helotiales
Zvarium_Lulworthiales
Amacrosclerotiorum_Helotiales
Pscopiformis_Helotiales
Ljuncicola_Helotiales
Lmacrosporus_Helotiales
Asarcoides_Helotiales
Enecator_Erysiphales
Mbicolor_Helotiales
Clongipes_Helotiales
Rericae_Helotiales
Rfirma_Helotiales
Bpaeoniae_Helotiales
Bconvoluta_Helotiales
Bmedusae_Helotiales
Bcalthae_Helotiales
Bpseudocinerea_Helotiales
Bcinerea_Helotiales
Belliptica_Helotiales
Bgalanthina_Helotiales
Bporri_Helotiales
Bnarcissicola_Helotiales
Bhyacinthi_Helotiales
Btulipae_Helotiales
Tstercoreus_Thelebolales
Tmicrosporus_Thelebolales
Lthalassiae_Lulworthiales
Pvexata_Xylariales
Koryzae_Trichosphaeriales
Amontagnei_Xylariales
Deschscholzii_Xylariales
Bnummularia_Xylariales
Aavocetta_Xylariales
Mtiffanyae_Melanosporales
Tradiata_Torpedosporales
Crosea_Hypocreales
Colidum_Hypocreales
Minundatum_Hypocreales
Bbassiana_Hypocreales
Cmilitaris_Hypocreales
Mtrigonosporus_Microascales
Cmaritima_Microascales
Czoysiae_Glomerellales
Gacutata_Glomerellales
Salkalinus_Glomerellales
Vdahliae_Hypocreales
Pcucumerina_Glomerellales
Cligniaria_Coniochaetales
Cinnumera_Chaetosphaeriales
Gtetrasperma_Sordariales
Cbulbillosum_Sordariales
Opiliferum_Ophiostomatales
Gclavigera_Ophiostomatales
Moryzae_Magnaporthales
Ggraminis_Magnaporthales
Mpoae_Magnaporthales
Paleophilum_Togniniales
Cpulchella_Calosphaeriales
Lminuta_Diaporthales
Dampelina_Diaporthales
Cchrysosperma_Diaporthales
Clustricola_Diaporthales
Cpopulea_Diaporthales
taxa
20
40
60
80
100
0
1
2
3
4
5
6
7
8
branch length

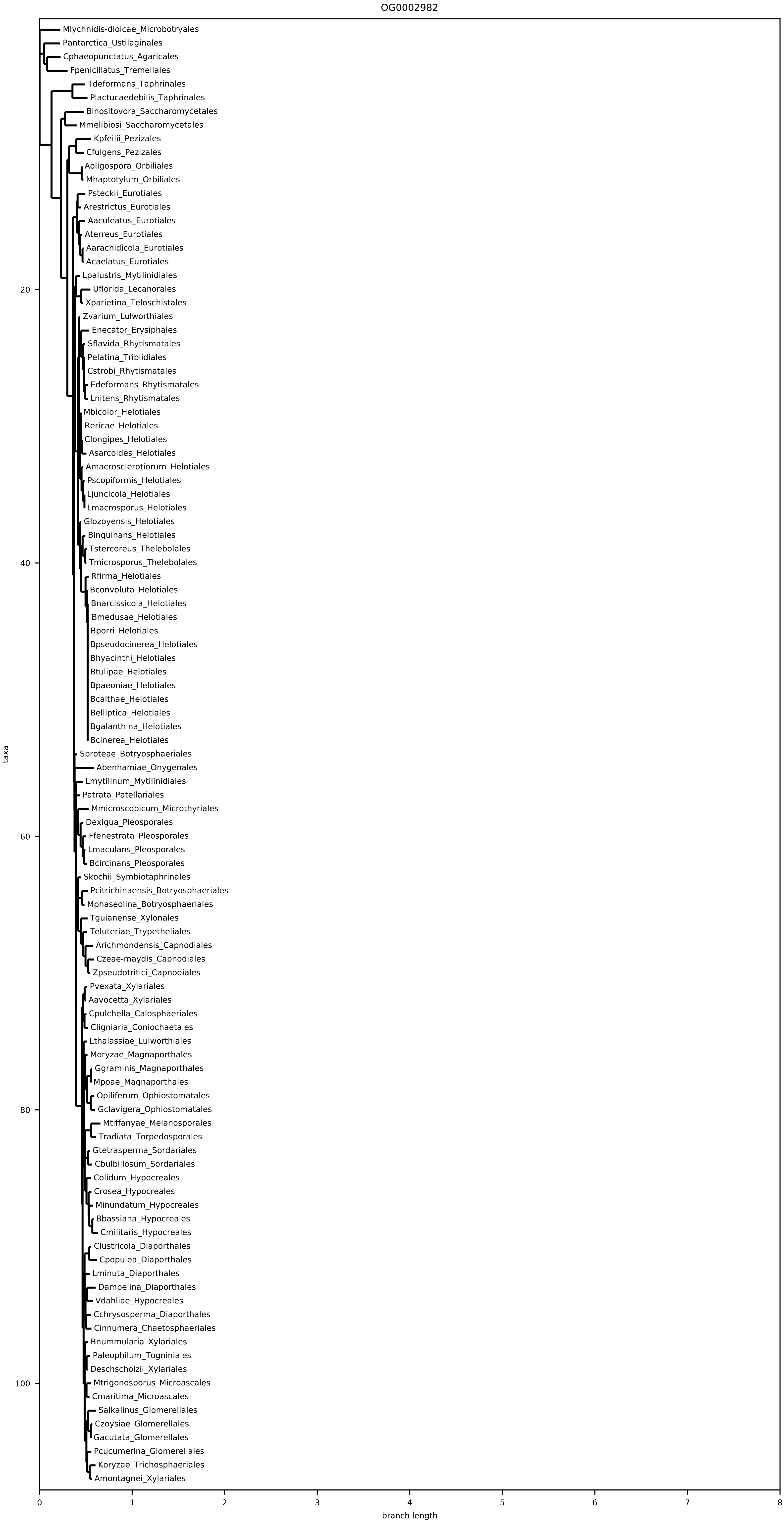

OG0002982
Mlychnidis-dioicae_Microbotryales
Pantarctica_Ustilaginales
Cphaeopunctatus_Agaricales
Fpenicillatus_Tremellales
Tdeformans_Taphrinales
Plactucaedebilis_Taphrinales
Binositovora_Saccharomycetales
Mmelibiosi_Saccharomycetales
Kpfeilii_Pezizales
Cfulgens_Pezizales
Aoligospora_Orbiliales
Mhaptotylum_Orbiliales
Psteckii_Eurotiales
Arestrictus_Eurotiales
Aaculeatus_Eurotiales
Aterreus_Eurotiales
Aarachidicola_Eurotiales
Acaelatus_Eurotiales
Lpalustris_Mytilinidiales
Uflorida_Lecanorales
Xparietina_Teloschistales
Zvarium_Lulworthiales
Enecator_Erysiphales
Sflavida_Rhytismatales
Pelatina_Triblidiales
Cstrobi_Rhytismatales
Edeformans_Rhytismatales
Lnitens_Rhytismatales
Mbicolor_Helotiales
Rericae_Helotiales
Clongipes_Helotiales
Asarcoides_Helotiales
Amacrosclerotiorum_Helotiales
Pscopiformis_Helotiales
Ljuncicola_Helotiales
Lmacrosporus_Helotiales
Glozoyensis_Helotiales
Binquinans_Helotiales
Tstercoreus_Thelebolales
Tmicrosporus_Thelebolales
Rfirma_Helotiales
Bconvoluta_Helotiales
Bnarcissicola_Helotiales
Bmedusae_Helotiales
Bporri_Helotiales
Bpseudocinerea_Helotiales
Bhyacinthi_Helotiales
Btulipae_Helotiales
Bpaeoniae_Helotiales
Bcalthae_Helotiales
Belliptica_Helotiales
Bgalanthina_Helotiales
Bcinerea_Helotiales
Sproteae_Botryosphaeriales
Abenhamiae_Onygenales
Lmytilinum_Mytilinidiales
Patrata_Patellariales
Mmicroscopicum_Microthyriales
Dexigua_Pleosporales
Ffenestrata_Pleosporales
Lmaculans_Pleosporales
Bcircinans_Pleosporales
Skochii_Symbiotaphrinales
Pcitrichinaensis_Botryosphaeriales
Mphaseolina_Botryosphaeriales
Tguianense_Xylonales
Teluteriae_Trypetheliales
Arichmondensis_Capnodiales
Czeae-maydis_Capnodiales
Zpseudotritici_Capnodiales
Pvexata_Xylariales
Aavocetta_Xylariales
Cpulchella_Calosphaeriales
Cligniaria_Coniochaetales
Lthalassiae_Lulworthiales
Moryzae_Magnaporthales
Ggraminis_Magnaporthales
Mpoae_Magnaporthales
Opiliferum_Ophiostomatales
Gclavigera_Ophiostomatales
Mtiffanyae_Melanosporales
Tradiata_Torpedosporales
Gtetrasperma_Sordariales
Cbulbillosum_Sordariales
Colidum_Hypocreales
Crosea_Hypocreales
Minundatum_Hypocreales
Bbassiana_Hypocreales
Cmilitaris_Hypocreales
Clustricola_Diaporthales
Cpopulea_Diaporthales
Lminuta_Diaporthales
Dampelina_Diaporthales
Vdahliae_Hypocreales
Cchrysosperma_Diaporthales
Cinnumera_Chaetosphaeriales
Bnummularia_Xylariales
Paleophilum_Togniniales
Deschscholzii_Xylariales
Mtrigonosporus_Microascales
Cmaritima_Microascales
Salkalinus_Glomerellales
Czoysiae_Glomerellales
Gacutata_Glomerellales
Pcucumerina_Glomerellales
Koryzae_Trichosphaeriales
Amontagnei_Xylariales
taxa
20
40
60
80
100
0
1
2
3
4
5
6
7
8
branch length

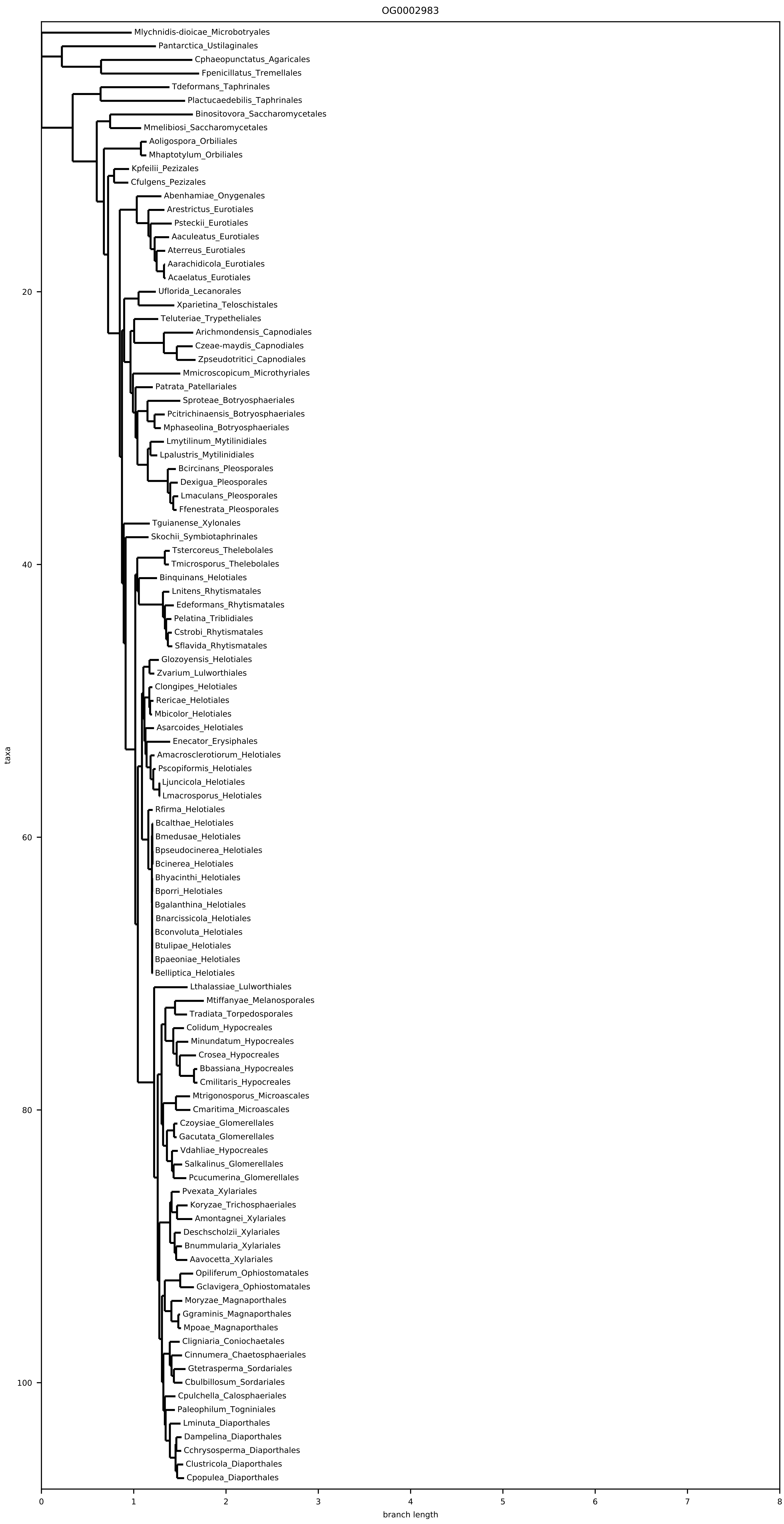

OG0002983
Mlychnidis-dioicae_Microbotryales
Pantarctica_Ustilaginales
Cphaeopunctatus_Agaricales
Fpenicillatus_Tremellales
Tdeformans_Taphrinales
Plactucaedebilis_Taphrinales
Binositovora_Saccharomycetales
Mmelibiosi_Saccharomycetales
Aoligospora_Orbiliales
Mhaptotylum_Orbiliales
Kpfeilii_Pezizales
Cfulgens_Pezizales
Abenhamiae_Onygenales
Arestrictus_Eurotiales
Psteckii_Eurotiales
Aaculeatus_Eurotiales
Aterreus_Eurotiales
Aarachidicola_Eurotiales
Acaelatus_Eurotiales
Uflorida_Lecanorales
Xparietina_Teloschistales
Teluteriae_Trypetheliales
Arichmondensis_Capnodiales
Czeae-maydis_Capnodiales
Zpseudotritici_Capnodiales
Mmicroscopicum_Microthyriales
Patrata_Patellariales
Sproteae_Botryosphaeriales
Pcitrichinaensis_Botryosphaeriales
Mphaseolina_Botryosphaeriales
Lmytilinum_Mytilinidiales
Lpalustris_Mytilinidiales
Bcircinans_Pleosporales
Dexigua_Pleosporales
Lmaculans_Pleosporales
Ffenestrata_Pleosporales
Tguianense_Xylonales
Skochii_Symbiotaphrinales
Tstercoreus_Thelebolales
Tmicrosporus_Thelebolales
Binquinans_Helotiales
Lnitens_Rhytismatales
Edeformans_Rhytismatales
Pelatina_Triblidiales
Cstrobi_Rhytismatales
Sflavida_Rhytismatales
Glozoyensis_Helotiales
Zvarium_Lulworthiales
Clongipes_Helotiales
Rericae_Helotiales
Mbicolor_Helotiales
Asarcoides_Helotiales
Enecator_Erysiphales
Amacrosclerotiorum_Helotiales
Pscopiformis_Helotiales
Ljuncicola_Helotiales
Lmacrosporus_Helotiales
Rfirma_Helotiales
Bcalthae_Helotiales
Bmedusae_Helotiales
Bpseudocinerea_Helotiales
Bcinerea_Helotiales
Bhyacinthi_Helotiales
Bporri_Helotiales
Bgalanthina_Helotiales
Bnarcissicola_Helotiales
Bconvoluta_Helotiales
Btulipae_Helotiales
Bpaeoniae_Helotiales
Belliptica_Helotiales
Lthalassiae_Lulworthiales
Mtiffanyae_Melanosporales
Tradiata_Torpedosporales
Colidum_Hypocreales
Minundatum_Hypocreales
Crosea_Hypocreales
Bbassiana_Hypocreales
Cmilitaris_Hypocreales
Mtrigonosporus_Microascales
Cmaritima_Microascales
Czoysiae_Glomerellales
Gacutata_Glomerellales
Vdahliae_Hypocreales
Salkalinus_Glomerellales
Pcucumerina_Glomerellales
Pvexata_Xylariales
Koryzae_Trichosphaeriales
Amontagnei_Xylariales
Deschscholzii_Xylariales
Bnummularia_Xylariales
Aavocetta_Xylariales
Opiliferum_Ophiostomatales
Gclavigera_Ophiostomatales
Moryzae_Magnaporthales
Ggraminis_Magnaporthales
Mpoae_Magnaporthales
Cligniaria_Coniochaetales
Cinnumera_Chaetosphaeriales
Gtetrasperma_Sordariales
Cbulbillosum_Sordariales
Cpulchella_Calosphaeriales
Paleophilum_Togniniales
Lminuta_Diaporthales
Dampelina_Diaporthales
Cchrysosperma_Diaporthales
Clustricola_Diaporthales
Cpopulea_Diaporthales
taxa
20
40
60
80
100
0
1
2
3
4
5
6
7
8
branch length

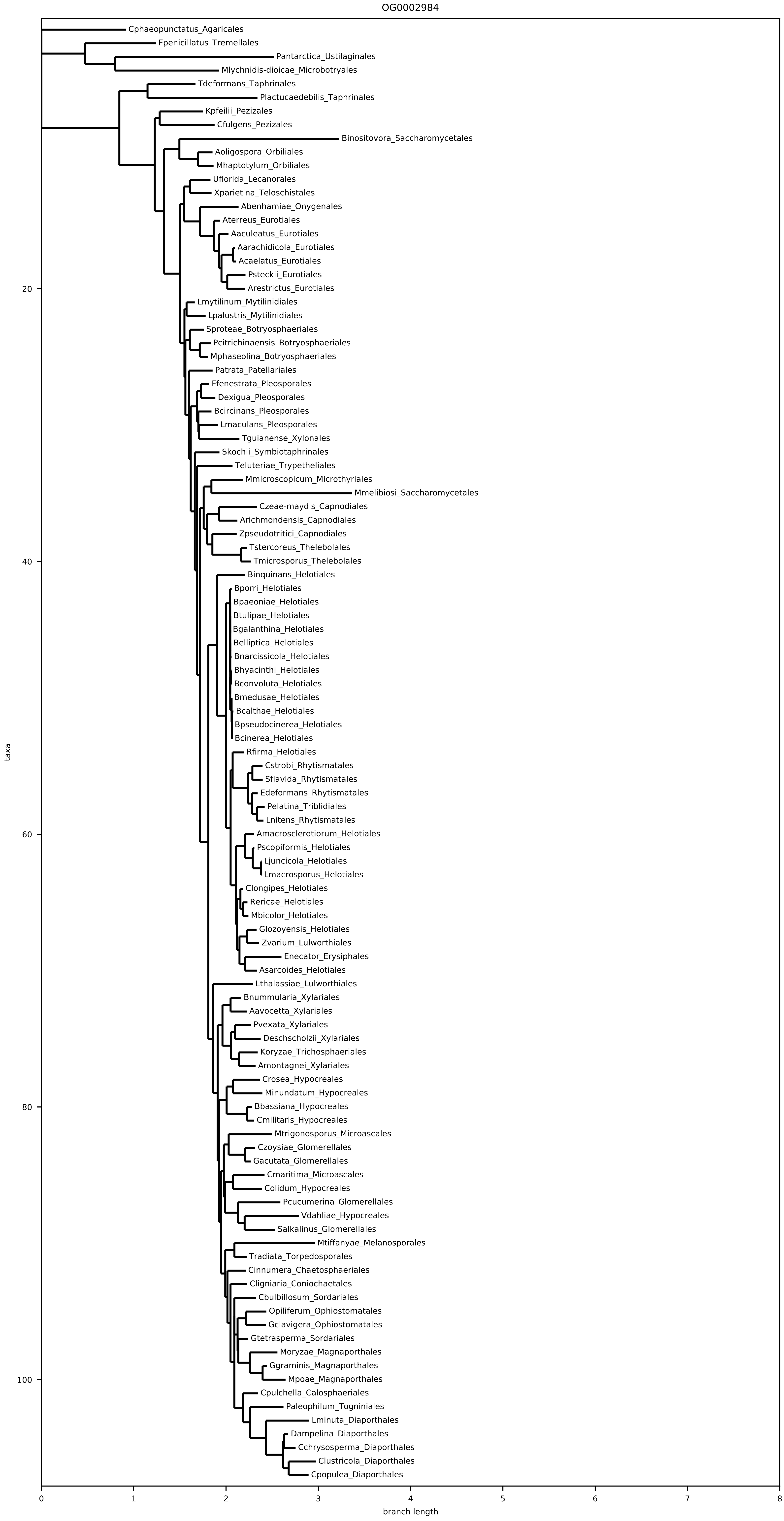

OG0002984
Cphaeopunctatus_Agaricales
Fpenicillatus_Tremellales
Pantarctica_Ustilaginales
Mlychnidis-dioicae_Microbotryales
Tdeformans_Taphrinales
Plactucaedebilis_Taphrinales
Kpfeilii_Pezizales
Cfulgens_Pezizales
Binositovora_Saccharomycetales
Aoligospora_Orbiliales
Mhaptotylum_Orbiliales
Uflorida_Lecanorales
Xparietina_Teloschistales
Abenhamiae_Onygenales
Aterreus_Eurotiales
Aaculeatus_Eurotiales
Aarachidicola_Eurotiales
Acaelatus_Eurotiales
Psteckii_Eurotiales
Arestrictus_Eurotiales
Lmytilinum_Mytilinidiales
Lpalustris_Mytilinidiales
Sproteae_Botryosphaeriales
Pcitrichinaensis_Botryosphaeriales
Mphaseolina_Botryosphaeriales
Patrata_Patellariales
Ffenestrata_Pleosporales
Dexigua_Pleosporales
Bcircinans_Pleosporales
Lmaculans_Pleosporales
Tguianense_Xylonales
Skochii_Symbiotaphrinales
Teluteriae_Trypetheliales
Mmicroscopicum_Microthyriales
Mmelibiosi_Saccharomycetales
Czeae-maydis_Capnodiales
Arichmondensis_Capnodiales
Zpseudotritici_Capnodiales
Tstercoreus_Thelebolales
Tmicrosporus_Thelebolales
Binquinans_Helotiales
Bporri_Helotiales
Bpaeoniae_Helotiales
Btulipae_Helotiales
Bgalanthina_Helotiales
Belliptica_Helotiales
Bnarcissicola_Helotiales
Bhyacinthi_Helotiales
Bconvoluta_Helotiales
Bmedusae_Helotiales
Bcalthae_Helotiales
Bpseudocinerea_Helotiales
Bcinerea_Helotiales
Rfirma_Helotiales
Cstrobi_Rhytismatales
Sflavida_Rhytismatales
Edeformans_Rhytismatales
Pelatina_Triblidiales
Lnitens_Rhytismatales
Amacrosclerotiorum_Helotiales
Pscopiformis_Helotiales
Ljuncicola_Helotiales
Lmacrosporus_Helotiales
Clongipes_Helotiales
Rericae_Helotiales
Mbicolor_Helotiales
Glozoyensis_Helotiales
Zvarium_Lulworthiales
Enecator_Erysiphales
Asarcoides_Helotiales
Lthalassiae_Lulworthiales
Bnummularia_Xylariales
Aavocetta_Xylariales
Pvexata_Xylariales
Deschscholzii_Xylariales
Koryzae_Trichosphaeriales
Amontagnei_Xylariales
Crosea_Hypocreales
Minundatum_Hypocreales
Bbassiana_Hypocreales
Cmilitaris_Hypocreales
Mtrigonosporus_Microascales
Czoysiae_Glomerellales
Gacutata_Glomerellales
Cmaritima_Microascales
Colidum_Hypocreales
Pcucumerina_Glomerellales
Vdahliae_Hypocreales
Salkalinus_Glomerellales
Mtiffanyae_Melanosporales
Tradiata_Torpedosporales
Cinnumera_Chaetosphaeriales
Cligniaria_Coniochaetales
Cbulbillosum_Sordariales
Opiliferum_Ophiostomatales
Gclavigera_Ophiostomatales
Gtetrasperma_Sordariales
Moryzae_Magnaporthales
Ggraminis_Magnaporthales
Mpoae_Magnaporthales
Cpulchella_Calosphaeriales
Paleophilum_Togniniales
Lminuta_Diaporthales
Dampelina_Diaporthales
Cchrysosperma_Diaporthales
Clustricola_Diaporthales
Cpopulea_Diaporthales
taxa
20
40
60
80
100
0
1
2
3
4
5
6
7
8
branch length

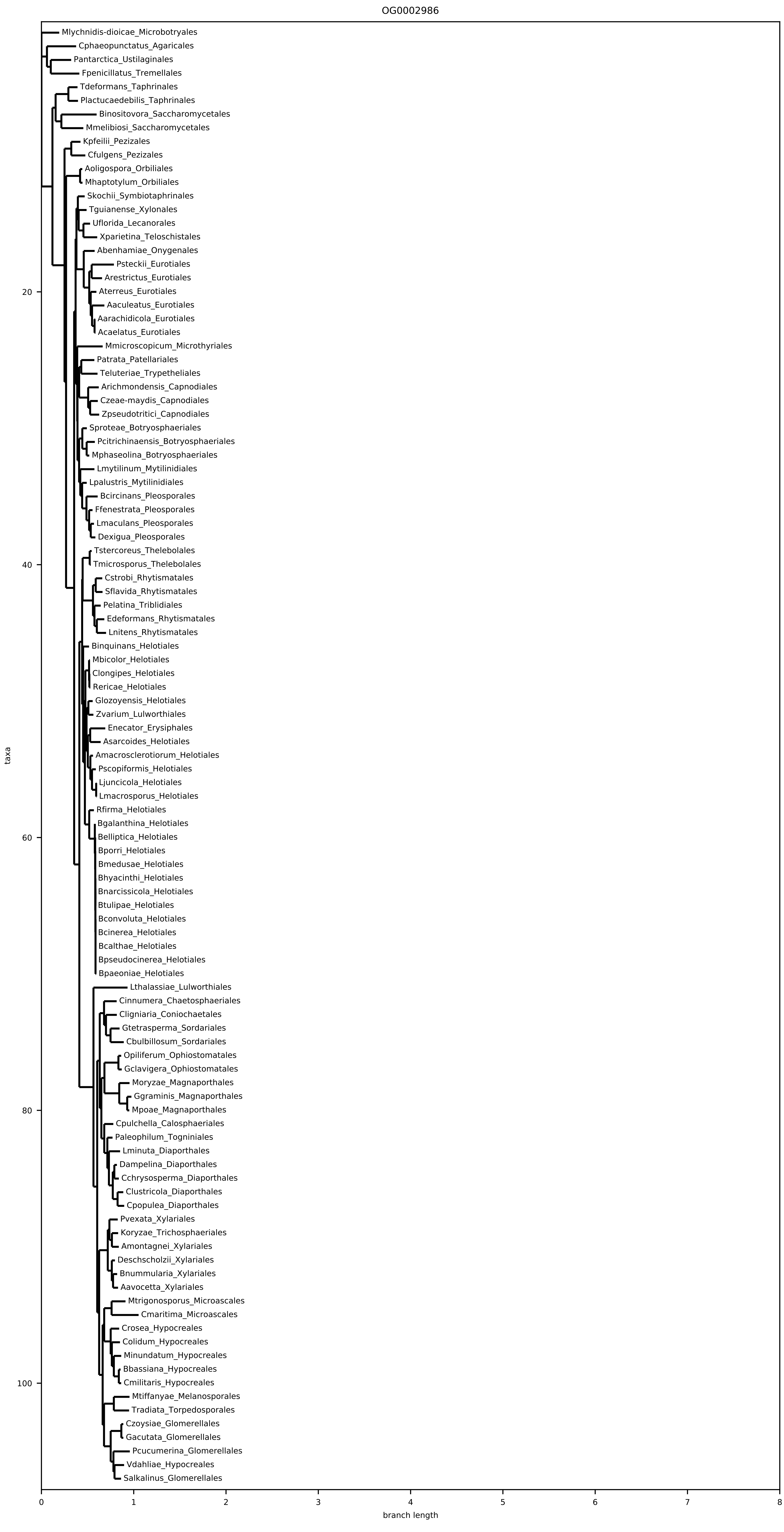

OG0002986
Mlychnidis-dioicae_Microbotryales
Cphaeopunctatus_Agaricales
Pantarctica_Ustilaginales
Fpenicillatus_Tremellales
Tdeformans_Taphrinales
Plactucaedebilis_Taphrinales
Binositovora_Saccharomycetales
Mmelibiosi_Saccharomycetales
Kpfeilii_Pezizales
Cfulgens_Pezizales
Aoligospora_Orbiliales
Mhaptotylum_Orbiliales
Skochii_Symbiotaphrinales
Tguianense_Xylonales
Uflorida_Lecanorales
Xparietina_Teloschistales
Abenhamiae_Onygenales
Psteckii_Eurotiales
Arestrictus_Eurotiales
Aterreus_Eurotiales
Aaculeatus_Eurotiales
Aarachidicola_Eurotiales
Acaelatus_Eurotiales
Mmicroscopicum_Microthyriales
Patrata_Patellariales
Teluteriae_Trypetheliales
Arichmondensis_Capnodiales
Czeae-maydis_Capnodiales
Zpseudotritici_Capnodiales
Sproteae_Botryosphaeriales
Pcitrichinaensis_Botryosphaeriales
Mphaseolina_Botryosphaeriales
Lmytilinum_Mytilinidiales
Lpalustris_Mytilinidiales
Bcircinans_Pleosporales
Ffenestrata_Pleosporales
Lmaculans_Pleosporales
Dexigua_Pleosporales
Tstercoreus_Thelebolales
Tmicrosporus_Thelebolales
Cstrobi_Rhytismatales
Sflavida_Rhytismatales
Pelatina_Triblidiales
Edeformans_Rhytismatales
Lnitens_Rhytismatales
Binquinans_Helotiales
Mbicolor_Helotiales
Clongipes_Helotiales
Rericae_Helotiales
Glozoyensis_Helotiales
Zvarium_Lulworthiales
Enecator_Erysiphales
Asarcoides_Helotiales
Amacrosclerotiorum_Helotiales
Pscopiformis_Helotiales
Ljuncicola_Helotiales
Lmacrosporus_Helotiales
Rfirma_Helotiales
Bgalanthina_Helotiales
Belliptica_Helotiales
Bporri_Helotiales
Bmedusae_Helotiales
Bhyacinthi_Helotiales
Bnarcissicola_Helotiales
Btulipae_Helotiales
Bconvoluta_Helotiales
Bcinerea_Helotiales
Bcalthae_Helotiales
Bpseudocinerea_Helotiales
Bpaeoniae_Helotiales
Lthalassiae_Lulworthiales
Cinnumera_Chaetosphaeriales
Cligniaria_Coniochaetales
Gtetrasperma_Sordariales
Cbulbillosum_Sordariales
Opiliferum_Ophiostomatales
Gclavigera_Ophiostomatales
Moryzae_Magnaporthales
Ggraminis_Magnaporthales
Mpoae_Magnaporthales
Cpulchella_Calosphaeriales
Paleophilum_Togniniales
Lminuta_Diaporthales
Dampelina_Diaporthales
Cchrysosperma_Diaporthales
Clustricola_Diaporthales
Cpopulea_Diaporthales
Pvexata_Xylariales
Koryzae_Trichosphaeriales
Amontagnei_Xylariales
Deschscholzii_Xylariales
Bnummularia_Xylariales
Aavocetta_Xylariales
Mtrigonosporus_Microascales
Cmaritima_Microascales
Crosea_Hypocreales
Colidum_Hypocreales
Minundatum_Hypocreales
Bbassiana_Hypocreales
Cmilitaris_Hypocreales
Mtiffanyae_Melanosporales
Tradiata_Torpedosporales
Czoysiae_Glomerellales
Gacutata_Glomerellales
Pcucumerina_Glomerellales
Vdahliae_Hypocreales
Salkalinus_Glomerellales
taxa
20
40
60
80
100
0
1
2
3
4
5
6
7
8
branch length

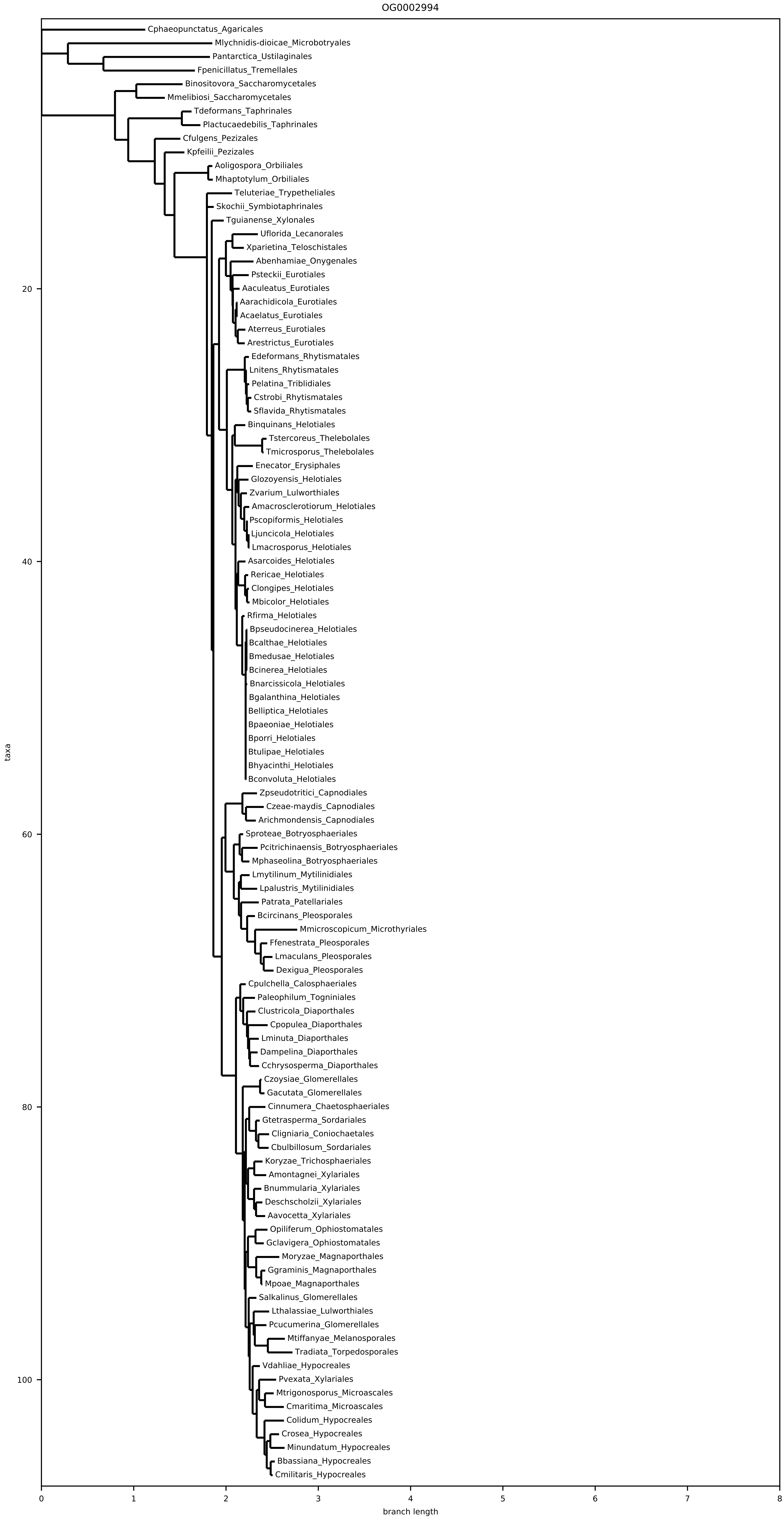

OG0002994
Cphaeopunctatus_Agaricales
Mlychnidis-dioicae_Microbotryales
Pantarctica_Ustilaginales
Fpenicillatus_Tremellales
Binositovora_Saccharomycetales
Mmelibiosi_Saccharomycetales
Tdeformans_Taphrinales
Plactucaedebilis_Taphrinales
Cfulgens_Pezizales
Kpfeilii_Pezizales
Aoligospora_Orbiliales
Mhaptotylum_Orbiliales
Teluteriae_Trypetheliales
Skochii_Symbiotaphrinales
Tguianense_Xylonales
Uflorida_Lecanorales
Xparietina_Teloschistales
Abenhamiae_Onygenales
Psteckii_Eurotiales
Aaculeatus_Eurotiales
Aarachidicola_Eurotiales
Acaelatus_Eurotiales
Aterreus_Eurotiales
Arestrictus_Eurotiales
Edeformans_Rhytismatales
Lnitens_Rhytismatales
Pelatina_Triblidiales
Cstrobi_Rhytismatales
Sflavida_Rhytismatales
Binquinans_Helotiales
Tstercoreus_Thelebolales
Tmicrosporus_Thelebolales
Enecator_Erysiphales
Glozoyensis_Helotiales
Zvarium_Lulworthiales
Amacrosclerotiorum_Helotiales
Pscopiformis_Helotiales
Ljuncicola_Helotiales
Lmacrosporus_Helotiales
Asarcoides_Helotiales
Rericae_Helotiales
Clongipes_Helotiales
Mbicolor_Helotiales
Rfirma_Helotiales
Bpseudocinerea_Helotiales
Bcalthae_Helotiales
Bmedusae_Helotiales
Bcinerea_Helotiales
Bnarcissicola_Helotiales
Bgalanthina_Helotiales
Belliptica_Helotiales
Bpaeoniae_Helotiales
Bporri_Helotiales
Btulipae_Helotiales
Bhyacinthi_Helotiales
Bconvoluta_Helotiales
Zpseudotritici_Capnodiales
Czeae-maydis_Capnodiales
Arichmondensis_Capnodiales
Sproteae_Botryosphaeriales
Pcitrichinaensis_Botryosphaeriales
Mphaseolina_Botryosphaeriales
Lmytilinum_Mytilinidiales
Lpalustris_Mytilinidiales
Patrata_Patellariales
Bcircinans_Pleosporales
Mmicroscopicum_Microthyriales
Ffenestrata_Pleosporales
Lmaculans_Pleosporales
Dexigua_Pleosporales
Cpulchella_Calosphaeriales
Paleophilum_Togniniales
Clustricola_Diaporthales
Cpopulea_Diaporthales
Lminuta_Diaporthales
Dampelina_Diaporthales
Cchrysosperma_Diaporthales
Czoysiae_Glomerellales
Gacutata_Glomerellales
Cinnumera_Chaetosphaeriales
Gtetrasperma_Sordariales
Cligniaria_Coniochaetales
Cbulbillosum_Sordariales
Koryzae_Trichosphaeriales
Amontagnei_Xylariales
Bnummularia_Xylariales
Deschscholzii_Xylariales
Aavocetta_Xylariales
Opiliferum_Ophiostomatales
Gclavigera_Ophiostomatales
Moryzae_Magnaporthales
Ggraminis_Magnaporthales
Mpoae_Magnaporthales
Salkalinus_Glomerellales
Lthalassiae_Lulworthiales
Pcucumerina_Glomerellales
Mtiffanyae_Melanosporales
Tradiata_Torpedosporales
Vdahliae_Hypocreales
Pvexata_Xylariales
Mtrigonosporus_Microascales
Cmaritima_Microascales
Colidum_Hypocreales
Crosea_Hypocreales
Minundatum_Hypocreales
Bbassiana_Hypocreales
Cmilitaris_Hypocreales
taxa
20
40
60
80
100
0
1
2
3
4
5
6
7
8
branch length

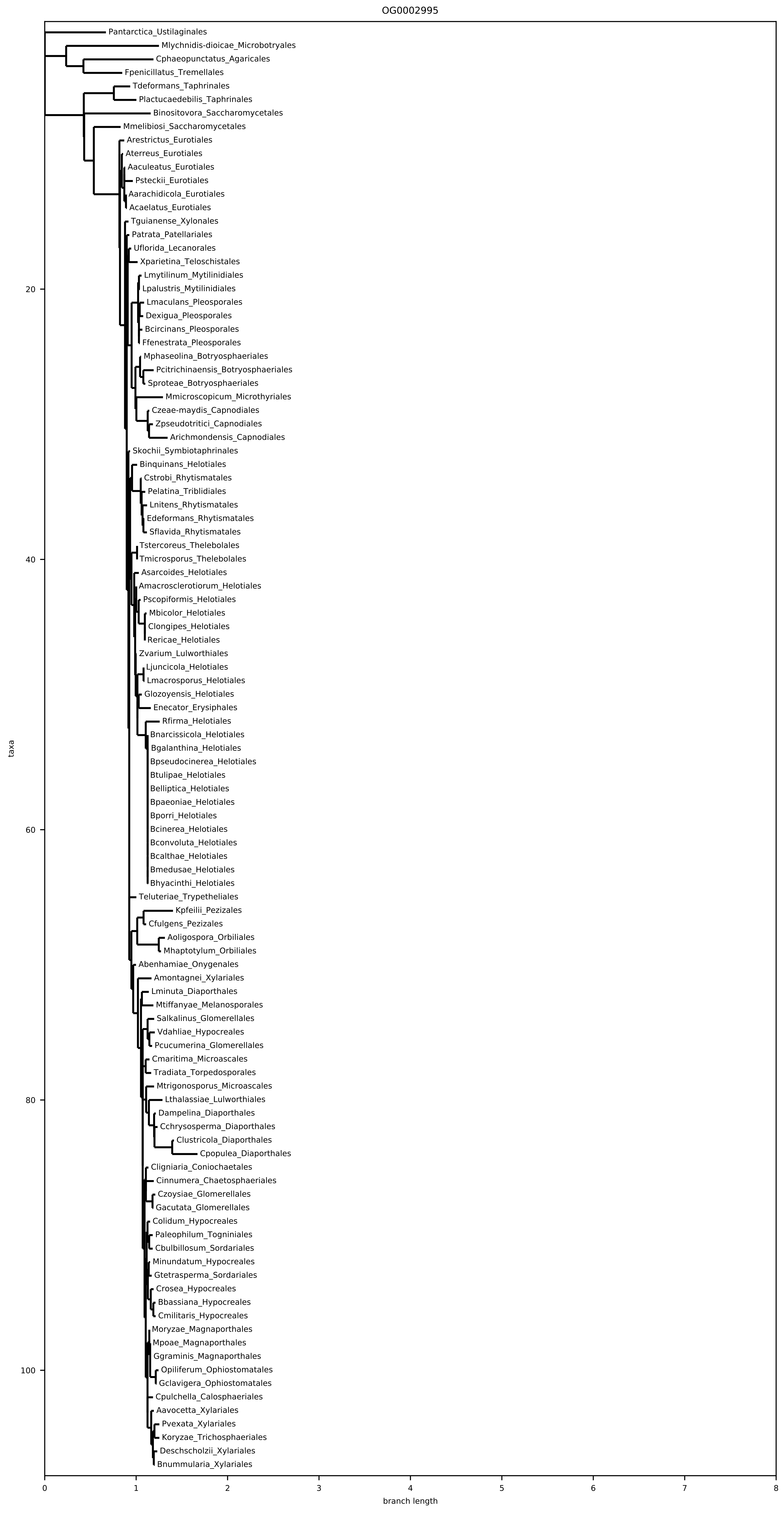

OG0002995
Pantarctica_Ustilaginales
Mlychnidis-dioicae_Microbotryales
Cphaeopunctatus_Agaricales
Fpenicillatus_Tremellales
Tdeformans_Taphrinales
Plactucaedebilis_Taphrinales
Binositovora_Saccharomycetales
Mmelibiosi_Saccharomycetales
Arestrictus_Eurotiales
Aterreus_Eurotiales
Aaculeatus_Eurotiales
Psteckii_Eurotiales
Aarachidicola_Eurotiales
Acaelatus_Eurotiales
Tguianense_Xylonales
Patrata_Patellariales
Uflorida_Lecanorales
Xparietina_Teloschistales
Lmytilinum_Mytilinidiales
Lpalustris_Mytilinidiales
Lmaculans_Pleosporales
Dexigua_Pleosporales
Bcircinans_Pleosporales
Ffenestrata_Pleosporales
Mphaseolina_Botryosphaeriales
Pcitrichinaensis_Botryosphaeriales
Sproteae_Botryosphaeriales
Mmicroscopicum_Microthyriales
Czeae-maydis_Capnodiales
Zpseudotritici_Capnodiales
Arichmondensis_Capnodiales
Skochii_Symbiotaphrinales
Binquinans_Helotiales
Cstrobi_Rhytismatales
Pelatina_Triblidiales
Lnitens_Rhytismatales
Edeformans_Rhytismatales
Sflavida_Rhytismatales
Tstercoreus_Thelebolales
Tmicrosporus_Thelebolales
Asarcoides_Helotiales
Amacrosclerotiorum_Helotiales
Pscopiformis_Helotiales
Mbicolor_Helotiales
Clongipes_Helotiales
Rericae_Helotiales
Zvarium_Lulworthiales
Ljuncicola_Helotiales
Lmacrosporus_Helotiales
Glozoyensis_Helotiales
Enecator_Erysiphales
Rfirma_Helotiales
Bnarcissicola_Helotiales
Bgalanthina_Helotiales
Bpseudocinerea_Helotiales
Btulipae_Helotiales
Belliptica_Helotiales
Bpaeoniae_Helotiales
Bporri_Helotiales
Bcinerea_Helotiales
Bconvoluta_Helotiales
Bcalthae_Helotiales
Bmedusae_Helotiales
Bhyacinthi_Helotiales
Teluteriae_Trypetheliales
Kpfeilii_Pezizales
Cfulgens_Pezizales
Aoligospora_Orbiliales
Mhaptotylum_Orbiliales
Abenhamiae_Onygenales
Amontagnei_Xylariales
Lminuta_Diaporthales
Mtiffanyae_Melanosporales
Salkalinus_Glomerellales
Vdahliae_Hypocreales
Pcucumerina_Glomerellales
Cmaritima_Microascales
Tradiata_Torpedosporales
Mtrigonosporus_Microascales
Lthalassiae_Lulworthiales
Dampelina_Diaporthales
Cchrysosperma_Diaporthales
Clustricola_Diaporthales
Cpopulea_Diaporthales
Cligniaria_Coniochaetales
Cinnumera_Chaetosphaeriales
Czoysiae_Glomerellales
Gacutata_Glomerellales
Colidum_Hypocreales
Paleophilum_Togniniales
Cbulbillosum_Sordariales
Minundatum_Hypocreales
Gtetrasperma_Sordariales
Crosea_Hypocreales
Bbassiana_Hypocreales
Cmilitaris_Hypocreales
Moryzae_Magnaporthales
Mpoae_Magnaporthales
Ggraminis_Magnaporthales
Opiliferum_Ophiostomatales
Gclavigera_Ophiostomatales
Cpulchella_Calosphaeriales
Aavocetta_Xylariales
Pvexata_Xylariales
Koryzae_Trichosphaeriales
Deschscholzii_Xylariales
Bnummularia_Xylariales
taxa
20
40
60
80
100
0
1
2
3
4
5
6
7
8
branch length

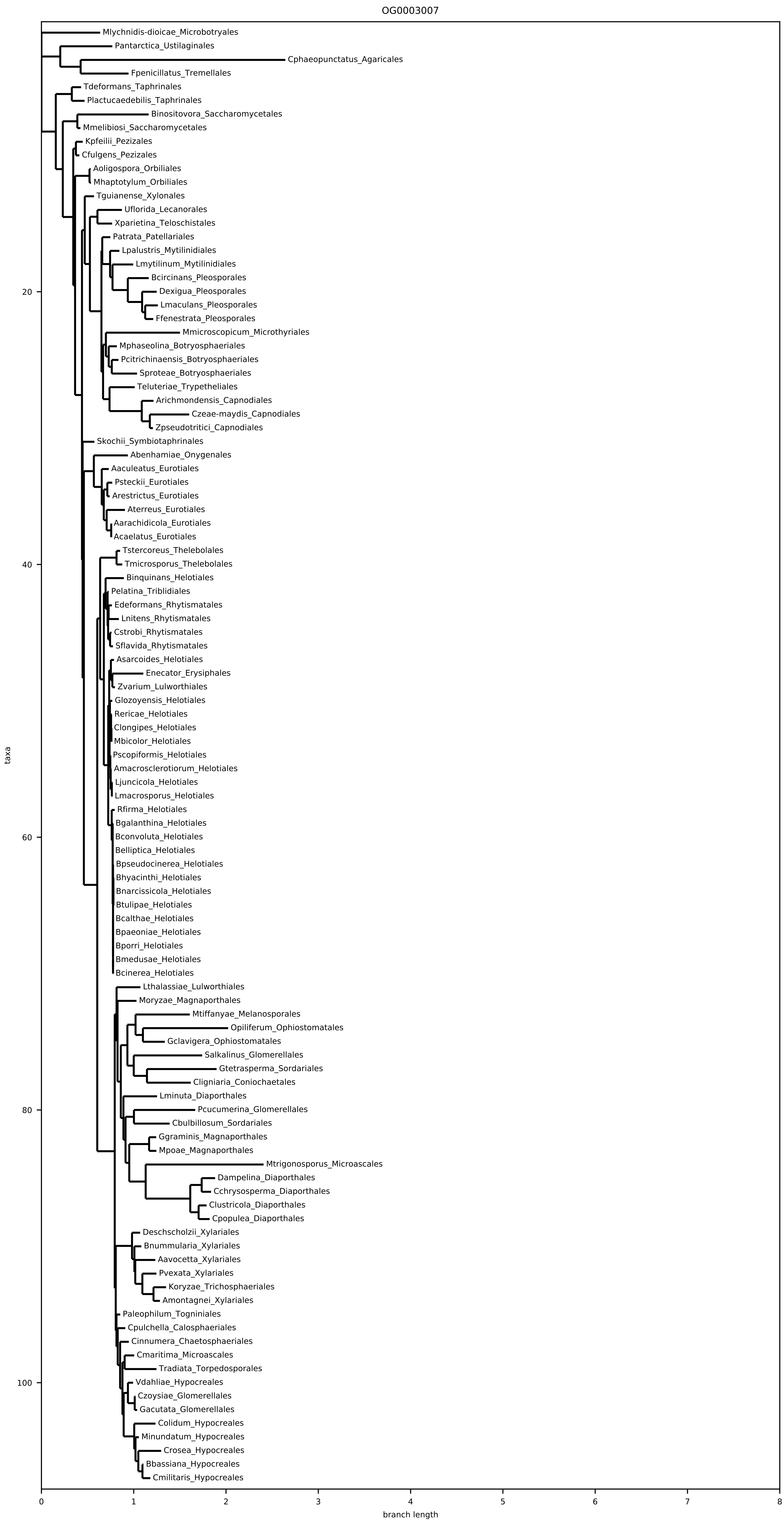

OG0003007
Mlychnidis-dioicae_Microbotryales
Pantarctica_Ustilaginales
Cphaeopunctatus_Agaricales
Fpenicillatus_Tremellales
Tdeformans_Taphrinales
Plactucaedebilis_Taphrinales
Binositovora_Saccharomycetales
Mmelibiosi_Saccharomycetales
Kpfeilii_Pezizales
Cfulgens_Pezizales
Aoligospora_Orbiliales
Mhaptotylum_Orbiliales
Tguianense_Xylonales
Uflorida_Lecanorales
Xparietina_Teloschistales
Patrata_Patellariales
Lpalustris_Mytilinidiales
Lmytilinum_Mytilinidiales
Bcircinans_Pleosporales
Dexigua_Pleosporales
Lmaculans_Pleosporales
Ffenestrata_Pleosporales
Mmicroscopicum_Microthyriales
Mphaseolina_Botryosphaeriales
Pcitrichinaensis_Botryosphaeriales
Sproteae_Botryosphaeriales
Teluteriae_Trypetheliales
Arichmondensis_Capnodiales
Czeae-maydis_Capnodiales
Zpseudotritici_Capnodiales
Skochii_Symbiotaphrinales
Abenhamiae_Onygenales
Aaculeatus_Eurotiales
Psteckii_Eurotiales
Arestrictus_Eurotiales
Aterreus_Eurotiales
Aarachidicola_Eurotiales
Acaelatus_Eurotiales
Tstercoreus_Thelebolales
Tmicrosporus_Thelebolales
Binquinans_Helotiales
Pelatina_Triblidiales
Edeformans_Rhytismatales
Lnitens_Rhytismatales
Cstrobi_Rhytismatales
Sflavida_Rhytismatales
Asarcoides_Helotiales
Enecator_Erysiphales
Zvarium_Lulworthiales
Glozoyensis_Helotiales
Rericae_Helotiales
Clongipes_Helotiales
Mbicolor_Helotiales
Pscopiformis_Helotiales
Amacrosclerotiorum_Helotiales
Ljuncicola_Helotiales
Lmacrosporus_Helotiales
Rfirma_Helotiales
Bgalanthina_Helotiales
Bconvoluta_Helotiales
Belliptica_Helotiales
Bpseudocinerea_Helotiales
Bhyacinthi_Helotiales
Bnarcissicola_Helotiales
Btulipae_Helotiales
Bcalthae_Helotiales
Bpaeoniae_Helotiales
Bporri_Helotiales
Bmedusae_Helotiales
Bcinerea_Helotiales
Lthalassiae_Lulworthiales
Moryzae_Magnaporthales
Mtiffanyae_Melanosporales
Opiliferum_Ophiostomatales
Gclavigera_Ophiostomatales
Salkalinus_Glomerellales
Gtetrasperma_Sordariales
Cligniaria_Coniochaetales
Lminuta_Diaporthales
Pcucumerina_Glomerellales
Cbulbillosum_Sordariales
Ggraminis_Magnaporthales
Mpoae_Magnaporthales
Mtrigonosporus_Microascales
Dampelina_Diaporthales
Cchrysosperma_Diaporthales
Clustricola_Diaporthales
Cpopulea_Diaporthales
Deschscholzii_Xylariales
Bnummularia_Xylariales
Aavocetta_Xylariales
Pvexata_Xylariales
Koryzae_Trichosphaeriales
Amontagnei_Xylariales
Paleophilum_Togniniales
Cpulchella_Calosphaeriales
Cinnumera_Chaetosphaeriales
Cmaritima_Microascales
Tradiata_Torpedosporales
Vdahliae_Hypocreales
Czoysiae_Glomerellales
Gacutata_Glomerellales
Colidum_Hypocreales
Minundatum_Hypocreales
Crosea_Hypocreales
Bbassiana_Hypocreales
Cmilitaris_Hypocreales
taxa
20
40
60
80
100
0
1
2
3
4
5
6
7
8
branch length

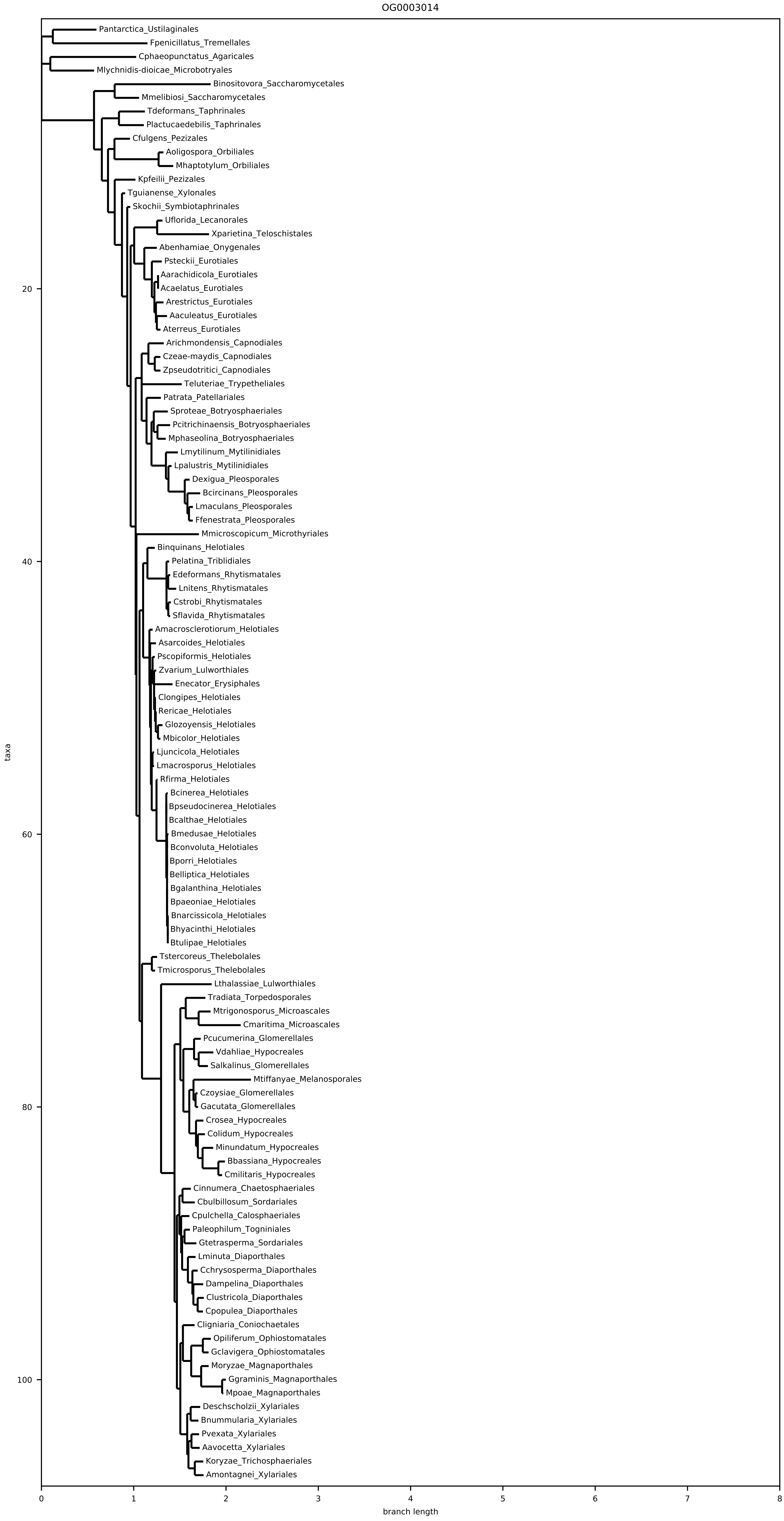
OG0003014
Pantarctica_Ustilaginales
Fpenicillatus_Tremellales
Cphaeopunctatus_Agaricales
Mlychnidis-dioicae_Microbotryales
Binositovora_Saccharomycetales
Mmelibiosi_Saccharomycetales
Tdeformans_Taphrinales
Plactucaedebilis_Taphrinales
Cfulgens_Pezizales
Aoligospora_Orbiliales
Mhaptotylum_Orbiliales
Kpfeilii_Pezizales
Tguianense_Xylonales
Skochii_Symbiotaphrinales
Uflorida_Lecanorales
Xparietina_Teloschistales
Abenhamiae_Onygenales
Psteckii_Eurotiales
Aarachidicola_Eurotiales
Acaelatus_Eurotiales
Arestrictus_Eurotiales
Aaculeatus_Eurotiales
Aterreus_Eurotiales
Arichmondensis_Capnodiales
Czeae-maydis_Capnodiales
Zpseudotritici_Capnodiales
Teluteriae_Trypetheliales
Patrata_Patellariales
Sproteae_Botryosphaeriales
Pcitrichinaensis_Botryosphaeriales
Mphaseolina_Botryosphaeriales
Lmytilinum_Mytilinidiales
Lpalustris_Mytilinidiales
Dexigua_Pleosporales
Bcircinans_Pleosporales
Lmaculans_Pleosporales
Ffenestrata_Pleosporales
Mmicroscopicum_Microthyriales
Binquinans_Helotiales
Pelatina_Triblidiales
Edeformans_Rhytismatales
Lnitens_Rhytismatales
Cstrobi_Rhytismatales
Sflavida_Rhytismatales
Amacrosclerotiorum_Helotiales
Asarcoides_Helotiales
Pscopiformis_Helotiales
Zvarium_Lulworthiales
Enecator_Erysiphales
Clongipes_Helotiales
Rericae_Helotiales
Glozoyensis_Helotiales
Mbicolor_Helotiales
Ljuncicola_Helotiales
Lmacrosporus_Helotiales
Rfirma_Helotiales
Bcinerea_Helotiales
Bpseudocinerea_Helotiales
Bcalthae_Helotiales
Bmedusae_Helotiales
Bconvoluta_Helotiales
Bporri_Helotiales
Belliptica_Helotiales
Bgalanthina_Helotiales
Bpaeoniae_Helotiales
Bnarcissicola_Helotiales
Bhyacinthi_Helotiales
Btulipae_Helotiales
Tstercoreus_Thelebolales
Tmicrosporus_Thelebolales
Lthalassiae_Lulworthiales
Tradiata_Torpedosporales
Mtrigonosporus_Microascales
Cmaritima_Microascales
Pcucumerina_Glomerellales
Vdahliae_Hypocreales
Salkalinus_Glomerellales
Mtiffanyae_Melanosporales
Czoysiae_Glomerellales
Gacutata_Glomerellales
Crosea_Hypocreales
Colidum_Hypocreales
Minundatum_Hypocreales
Bbassiana_Hypocreales
Cmilitaris_Hypocreales
Cinnumera_Chaetosphaeriales
Cbulbillosum_Sordariales
Cpulchella_Calosphaeriales
Paleophilum_Togniniales
Gtetrasperma_Sordariales
Lminuta_Diaporthales
Cchrysosperma_Diaporthales
Dampelina_Diaporthales
Clustricola_Diaporthales
Cpopulea_Diaporthales
Cligniaria_Coniochaetales
Opiliferum_Ophiostomatales
Gclavigera_Ophiostomatales
Moryzae_Magnaporthales
Ggraminis_Magnaporthales
Mpoae_Magnaporthales
Deschscholzii_Xylariales
Bnummularia_Xylariales
Pvexata_Xylariales
Aavocetta_Xylariales
Koryzae_Trichosphaeriales
Amontagnei_Xylariales
taxa
20
40
60
80
100
0
1
2
3
4
5
6
7
8
branch length

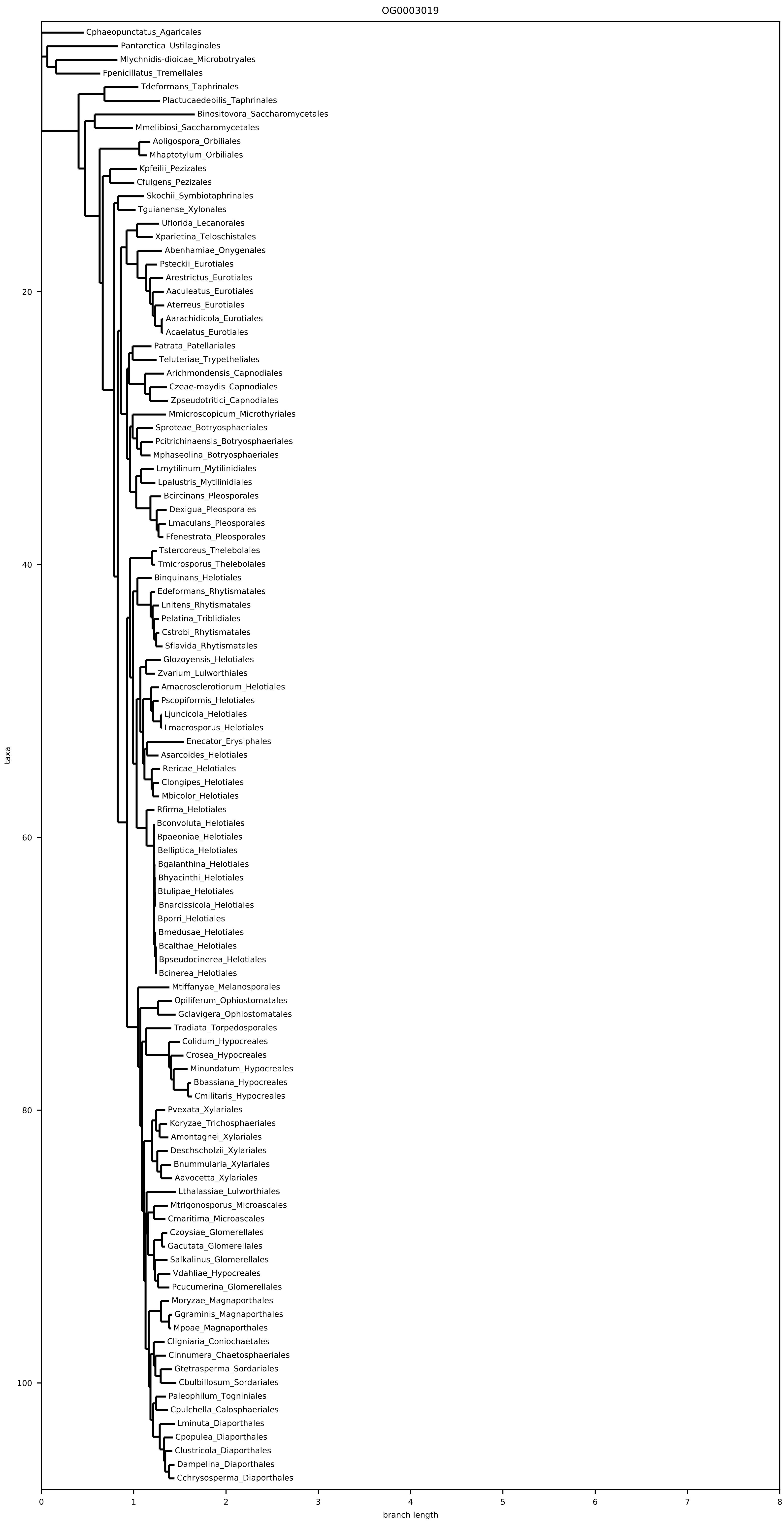
OG0003019
Cphaeopunctatus_Agaricales
Pantarctica_Ustilaginales
Mlychnidis-dioicae_Microbotryales
Fpenicillatus_Tremellales
Tdeformans_Taphrinales
Plactucaedebilis_Taphrinales
Binositovora_Saccharomycetales
Mmelibiosi_Saccharomycetales
Aoligospora_Orbiliales
Mhaptotylum_Orbiliales
Kpfeilii_Pezizales
Cfulgens_Pezizales
Skochii_Symbiotaphrinales
Tguianense_Xylonales
Uflorida_Lecanorales
Xparietina_Teloschistales
Abenhamiae_Onygenales
Psteckii_Eurotiales
Arestrictus_Eurotiales
Aaculeatus_Eurotiales
Aterreus_Eurotiales
Aarachidicola_Eurotiales
Acaelatus_Eurotiales
Patrata_Patellariales
Teluteriae_Trypetheliales
Arichmondensis_Capnodiales
Czeae-maydis_Capnodiales
Zpseudotritici_Capnodiales
Mmicroscopicum_Microthyriales
Sproteae_Botryosphaeriales
Pcitrichinaensis_Botryosphaeriales
Mphaseolina_Botryosphaeriales
Lmytilinum_Mytilinidiales
Lpalustris_Mytilinidiales
Bcircinans_Pleosporales
Dexigua_Pleosporales
Lmaculans_Pleosporales
Ffenestrata_Pleosporales
Tstercoreus_Thelebolales
Tmicrosporus_Thelebolales
Binquinans_Helotiales
Edeformans_Rhytismatales
Lnitens_Rhytismatales
Pelatina_Triblidiales
Cstrobi_Rhytismatales
Sflavida_Rhytismatales
Glozoyensis_Helotiales
Zvarium_Lulworthiales
Amacrosclerotiorum_Helotiales
Pscopiformis_Helotiales
Ljuncicola_Helotiales
Lmacrosporus_Helotiales
Enecator_Erysiphales
Asarcoides_Helotiales
Rericae_Helotiales
Clongipes_Helotiales
Mbicolor_Helotiales
Rfirma_Helotiales
Bconvoluta_Helotiales
Bpaeoniae_Helotiales
Belliptica_Helotiales
Bgalanthina_Helotiales
Bhyacinthi_Helotiales
Btulipae_Helotiales
Bnarcissicola_Helotiales
Bporri_Helotiales
Bmedusae_Helotiales
Bcalthae_Helotiales
Bpseudocinerea_Helotiales
Bcinerea_Helotiales
Mtiffanyae_Melanosporales
Opiliferum_Ophiostomatales
Gclavigera_Ophiostomatales
Tradiata_Torpedosporales
Colidum_Hypocreales
Crosea_Hypocreales
Minundatum_Hypocreales
Bbassiana_Hypocreales
Cmilitaris_Hypocreales
Pvexata_Xylariales
Koryzae_Trichosphaeriales
Amontagnei_Xylariales
Deschscholzii_Xylariales
Bnummularia_Xylariales
Aavocetta_Xylariales
Lthalassiae_Lulworthiales
Mtrigonosporus_Microascales
Cmaritima_Microascales
Czoysiae_Glomerellales
Gacutata_Glomerellales
Salkalinus_Glomerellales
Vdahliae_Hypocreales
Pcucumerina_Glomerellales
Moryzae_Magnaporthales
Ggraminis_Magnaporthales
Mpoae_Magnaporthales
Cligniaria_Coniochaetales
Cinnumera_Chaetosphaeriales
Gtetrasperma_Sordariales
Cbulbillosum_Sordariales
Paleophilum_Togniniales
Cpulchella_Calosphaeriales
Lminuta_Diaporthales
Cpopulea_Diaporthales
Clustricola_Diaporthales
Dampelina_Diaporthales
Cchrysosperma_Diaporthales
taxa
20
40
60
80
100
0
1
2
3
4
5
6
7
8
branch length

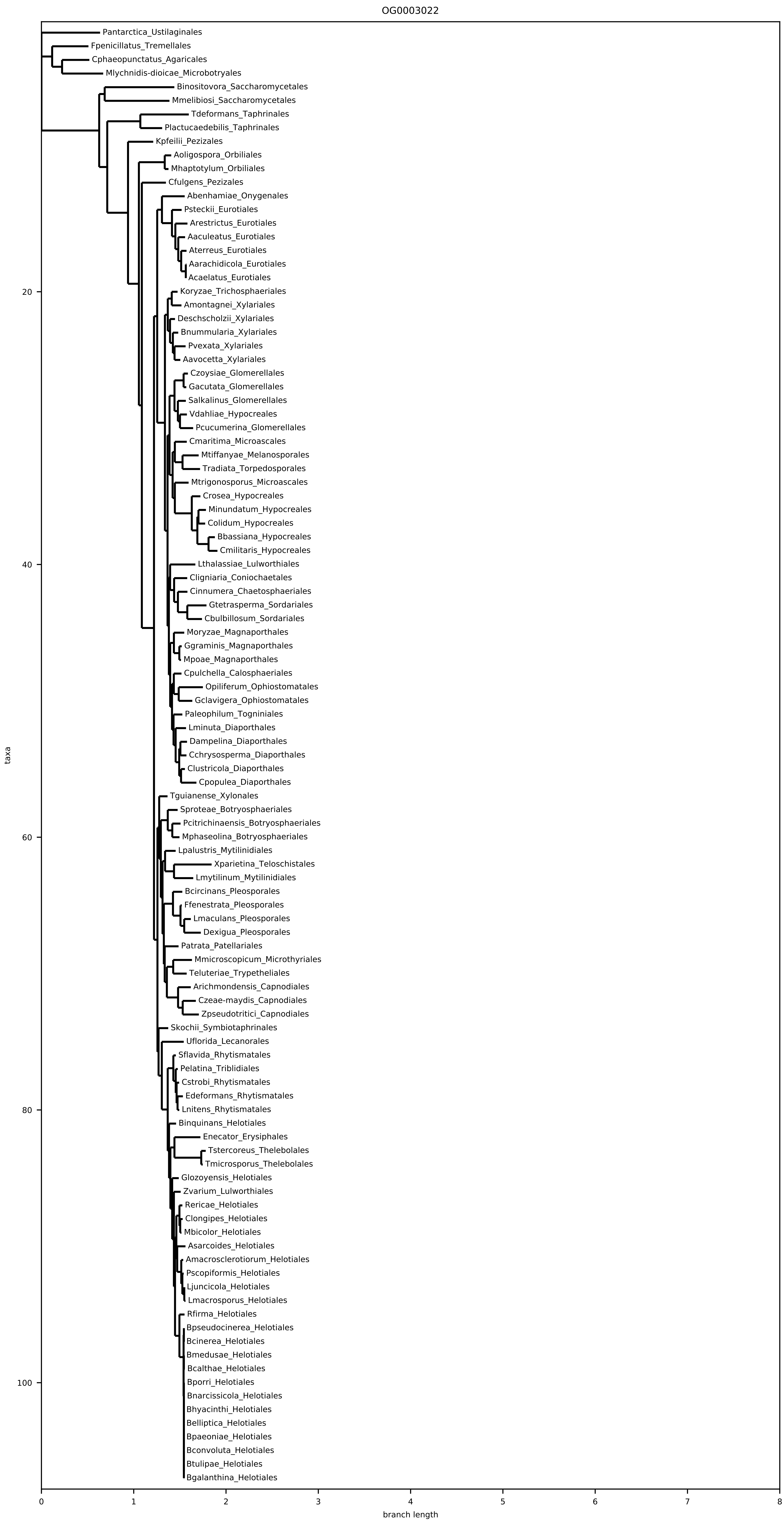
OG0003022
Pantarctica_Ustilaginales
Fpenicillatus_Tremellales
Cphaeopunctatus_Agaricales
Mlychnidis-dioicae_Microbotryales
Binositovora_Saccharomycetales
Mmelibiosi_Saccharomycetales
Tdeformans_Taphrinales
Plactucaedebilis_Taphrinales
Kpfeilii_Pezizales
Aoligospora_Orbiliales
Mhaptotylum_Orbiliales
Cfulgens_Pezizales
Abenhamiae_Onygenales
Psteckii_Eurotiales
Arestrictus_Eurotiales
Aaculeatus_Eurotiales
Aterreus_Eurotiales
Aarachidicola_Eurotiales
Acaelatus_Eurotiales
Koryzae_Trichosphaeriales
Amontagnei_Xylariales
Deschscholzii_Xylariales
Bnummularia_Xylariales
Pvexata_Xylariales
Aavocetta_Xylariales
Czoysiae_Glomerellales
Gacutata_Glomerellales
Salkalinus_Glomerellales
Vdahliae_Hypocreales
Pcucumerina_Glomerellales
Cmaritima_Microascales
Mtiffanyae_Melanosporales
Tradiata_Torpedosporales
Mtrigonosporus_Microascales
Crosea_Hypocreales
Minundatum_Hypocreales
Colidum_Hypocreales
Bbassiana_Hypocreales
Cmilitaris_Hypocreales
Lthalassiae_Lulworthiales
Cligniaria_Coniochaetales
Cinnumera_Chaetosphaeriales
Gtetrasperma_Sordariales
Cbulbillosum_Sordariales
Moryzae_Magnaporthales
Ggraminis_Magnaporthales
Mpoae_Magnaporthales
Cpulchella_Calosphaeriales
Opiliferum_Ophiostomatales
Gclavigera_Ophiostomatales
Paleophilum_Togniniales
Lminuta_Diaporthales
Dampelina_Diaporthales
Cchrysosperma_Diaporthales
Clustricola_Diaporthales
Cpopulea_Diaporthales
Tguianense_Xylonales
Sproteae_Botryosphaeriales
Pcitrichinaensis_Botryosphaeriales
Mphaseolina_Botryosphaeriales
Lpalustris_Mytilinidiales
Xparietina_Teloschistales
Lmytilinum_Mytilinidiales
Bcircinans_Pleosporales
Ffenestrata_Pleosporales
Lmaculans_Pleosporales
Dexigua_Pleosporales
Patrata_Patellariales
Mmicroscopicum_Microthyriales
Teluteriae_Trypetheliales
Arichmondensis_Capnodiales
Czeae-maydis_Capnodiales
Zpseudotritici_Capnodiales
Skochii_Symbiotaphrinales
Uflorida_Lecanorales
Sflavida_Rhytismatales
Pelatina_Triblidiales
Cstrobi_Rhytismatales
Edeformans_Rhytismatales
Lnitens_Rhytismatales
Binquinans_Helotiales
Enecator_Erysiphales
Tstercoreus_Thelebolales
Tmicrosporus_Thelebolales
Glozoyensis_Helotiales
Zvarium_Lulworthiales
Rericae_Helotiales
Clongipes_Helotiales
Mbicolor_Helotiales
Asarcoides_Helotiales
Amacrosclerotiorum_Helotiales
Pscopiformis_Helotiales
Ljuncicola_Helotiales
Lmacrosporus_Helotiales
Rfirma_Helotiales
Bpseudocinerea_Helotiales
Bcinerea_Helotiales
Bmedusae_Helotiales
Bcalthae_Helotiales
Bporri_Helotiales
Bnarcissicola_Helotiales
Bhyacinthi_Helotiales
Belliptica_Helotiales
Bpaeoniae_Helotiales
Bconvoluta_Helotiales
Btulipae_Helotiales
Bgalanthina_Helotiales
taxa
20
40
60
80
100
0
1
2
3
4
5
6
7
8
branch length

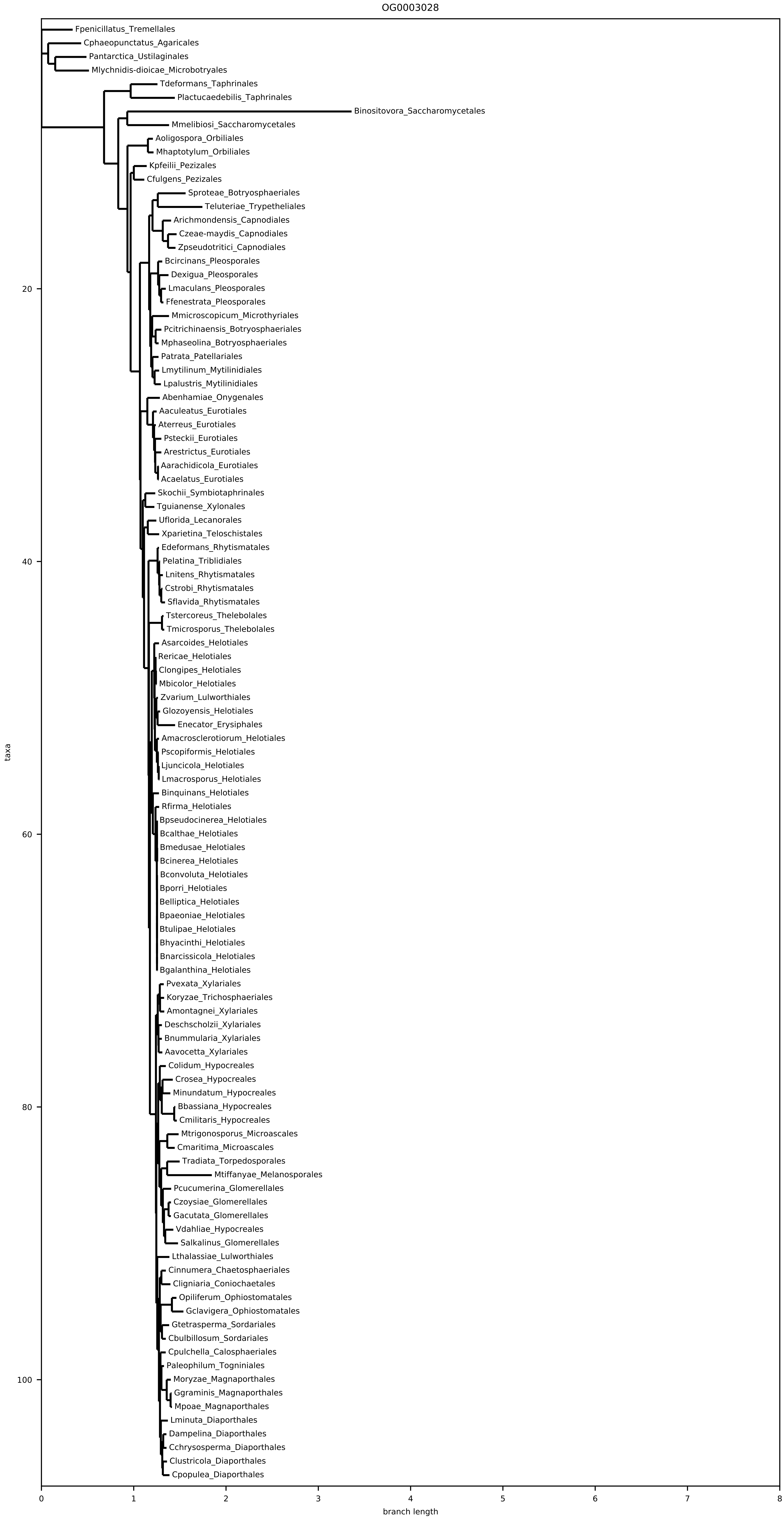
OG0003028
Fpenicillatus_Tremellales
Cphaeopunctatus_Agaricales
Pantarctica_Ustilaginales
Mlychnidis-dioicae_Microbotryales
Tdeformans_Taphrinales
Plactucaedebilis_Taphrinales
Binositovora_Saccharomycetales
Mmelibiosi_Saccharomycetales
Aoligospora_Orbiliales
Mhaptotylum_Orbiliales
Kpfeilii_Pezizales
Cfulgens_Pezizales
Sproteae_Botryosphaeriales
Teluteriae_Trypetheliales
Arichmondensis_Capnodiales
Czeae-maydis_Capnodiales
Zpseudotritici_Capnodiales
Bcircinans_Pleosporales
Dexigua_Pleosporales
Lmaculans_Pleosporales
Ffenestrata_Pleosporales
Mmicroscopicum_Microthyriales
Pcitrichinaensis_Botryosphaeriales
Mphaseolina_Botryosphaeriales
Patrata_Patellariales
Lmytilinum_Mytilinidiales
Lpalustris_Mytilinidiales
Abenhamiae_Onygenales
Aaculeatus_Eurotiales
Aterreus_Eurotiales
Psteckii_Eurotiales
Arestrictus_Eurotiales
Aarachidicola_Eurotiales
Acaelatus_Eurotiales
Skochii_Symbiotaphrinales
Tguianense_Xylonales
Uflorida_Lecanorales
Xparietina_Teloschistales
Edeformans_Rhytismatales
Pelatina_Triblidiales
Lnitens_Rhytismatales
Cstrobi_Rhytismatales
Sflavida_Rhytismatales
Tstercoreus_Thelebolales
Tmicrosporus_Thelebolales
Asarcoides_Helotiales
Rericae_Helotiales
Clongipes_Helotiales
Mbicolor_Helotiales
Zvarium_Lulworthiales
Glozoyensis_Helotiales
Enecator_Erysiphales
Amacrosclerotiorum_Helotiales
Pscopiformis_Helotiales
Ljuncicola_Helotiales
Lmacrosporus_Helotiales
Binquinans_Helotiales
Rfirma_Helotiales
Bpseudocinerea_Helotiales
Bcalthae_Helotiales
Bmedusae_Helotiales
Bcinerea_Helotiales
Bconvoluta_Helotiales
Bporri_Helotiales
Belliptica_Helotiales
Bpaeoniae_Helotiales
Btulipae_Helotiales
Bhyacinthi_Helotiales
Bnarcissicola_Helotiales
Bgalanthina_Helotiales
Pvexata_Xylariales
Koryzae_Trichosphaeriales
Amontagnei_Xylariales
Deschscholzii_Xylariales
Bnummularia_Xylariales
Aavocetta_Xylariales
Colidum_Hypocreales
Crosea_Hypocreales
Minundatum_Hypocreales
Bbassiana_Hypocreales
Cmilitaris_Hypocreales
Mtrigonosporus_Microascales
Cmaritima_Microascales
Tradiata_Torpedosporales
Mtiffanyae_Melanosporales
Pcucumerina_Glomerellales
Czoysiae_Glomerellales
Gacutata_Glomerellales
Vdahliae_Hypocreales
Salkalinus_Glomerellales
Lthalassiae_Lulworthiales
Cinnumera_Chaetosphaeriales
Cligniaria_Coniochaetales
Opiliferum_Ophiostomatales
Gclavigera_Ophiostomatales
Gtetrasperma_Sordariales
Cbulbillosum_Sordariales
Cpulchella_Calosphaeriales
Paleophilum_Togniniales
Moryzae_Magnaporthales
Ggraminis_Magnaporthales
Mpoae_Magnaporthales
Lminuta_Diaporthales
Dampelina_Diaporthales
Cchrysosperma_Diaporthales
Clustricola_Diaporthales
Cpopulea_Diaporthales
taxa
20
40
60
80
100
0
1
2
3
4
5
6
7
8
branch length

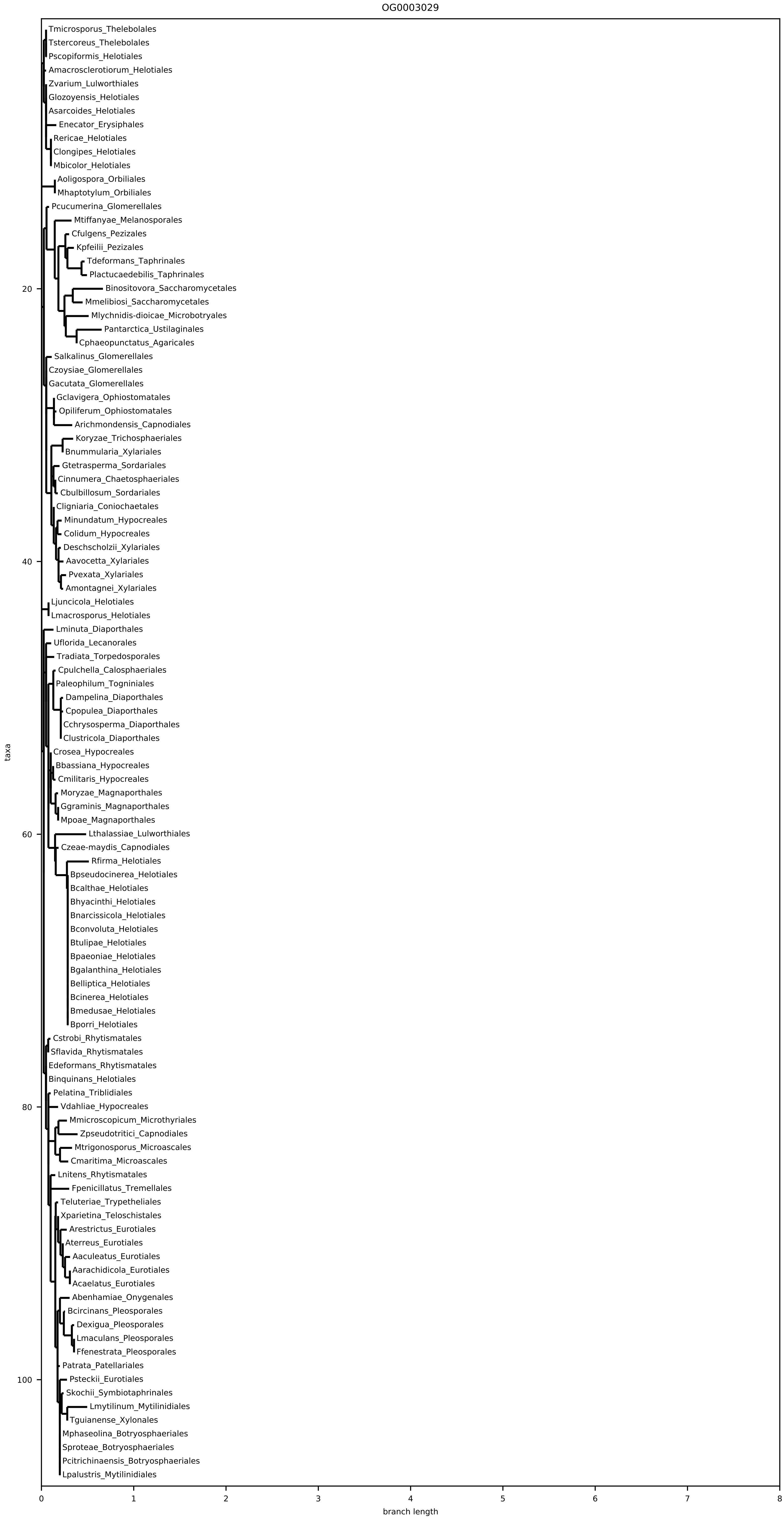
OG0003029
Tmicrosporus_Thelebolales
Tstercoreus_Thelebolales
Pscopiformis_Helotiales
Amacrosclerotiorum_Helotiales
Zvarium_Lulworthiales
Glozoyensis_Helotiales
Asarcoides_Helotiales
Enecator_Erysiphales
Rericae_Helotiales
Clongipes_Helotiales
Mbicolor_Helotiales
Aoligospora_Orbiliales
Mhaptotylum_Orbiliales
Pcucumerina_Glomerellales
Mtiffanyae_Melanosporales
Cfulgens_Pezizales
Kpfeilii_Pezizales
Tdeformans_Taphrinales
Plactucaedebilis_Taphrinales
Binositovora_Saccharomycetales
Mmelibiosi_Saccharomycetales
Mlychnidis-dioicae_Microbotryales
Pantarctica_Ustilaginales
Cphaeopunctatus_Agaricales
Salkalinus_Glomerellales
Czoysiae_Glomerellales
Gacutata_Glomerellales
Gclavigera_Ophiostomatales
Opiliferum_Ophiostomatales
Arichmondensis_Capnodiales
Koryzae_Trichosphaeriales
Bnummularia_Xylariales
Gtetrasperma_Sordariales
Cinnumera_Chaetosphaeriales
Cbulbillosum_Sordariales
Cligniaria_Coniochaetales
Minundatum_Hypocreales
Colidum_Hypocreales
Deschscholzii_Xylariales
Aavocetta_Xylariales
Pvexata_Xylariales
Amontagnei_Xylariales
Ljuncicola_Helotiales
Lmacrosporus_Helotiales
Lminuta_Diaporthales
Uflorida_Lecanorales
Tradiata_Torpedosporales
Cpulchella_Calosphaeriales
Paleophilum_Togniniales
Dampelina_Diaporthales
Cpopulea_Diaporthales
Cchrysosperma_Diaporthales
Clustricola_Diaporthales
Crosea_Hypocreales
Bbassiana_Hypocreales
Cmilitaris_Hypocreales
Moryzae_Magnaporthales
Ggraminis_Magnaporthales
Mpoae_Magnaporthales
Lthalassiae_Lulworthiales
Czeae-maydis_Capnodiales
Rfirma_Helotiales
Bpseudocinerea_Helotiales
Bcalthae_Helotiales
Bhyacinthi_Helotiales
Bnarcissicola_Helotiales
Bconvoluta_Helotiales
Btulipae_Helotiales
Bpaeoniae_Helotiales
Bgalanthina_Helotiales
Belliptica_Helotiales
Bcinerea_Helotiales
Bmedusae_Helotiales
Bporri_Helotiales
Cstrobi_Rhytismatales
Sflavida_Rhytismatales
Edeformans_Rhytismatales
Binquinans_Helotiales
Pelatina_Triblidiales
Vdahliae_Hypocreales
Mmicroscopicum_Microthyriales
Zpseudotritici_Capnodiales
Mtrigonosporus_Microascales
Cmaritima_Microascales
Lnitens_Rhytismatales
Fpenicillatus_Tremellales
Teluteriae_Trypetheliales
Xparietina_Teloschistales
Arestrictus_Eurotiales
Aterreus_Eurotiales
Aaculeatus_Eurotiales
Aarachidicola_Eurotiales
Acaelatus_Eurotiales
Abenhamiae_Onygenales
Bcircinans_Pleosporales
Dexigua_Pleosporales
Lmaculans_Pleosporales
Ffenestrata_Pleosporales
Patrata_Patellariales
Psteckii_Eurotiales
Skochii_Symbiotaphrinales
Lmytilinum_Mytilinidiales
Tguianense_Xylonales
Mphaseolina_Botryosphaeriales
Sproteae_Botryosphaeriales
Pcitrichinaensis_Botryosphaeriales
Lpalustris_Mytilinidiales
taxa
20
40
60
80
100
0
1
2
3
4
5
6
7
8
branch length

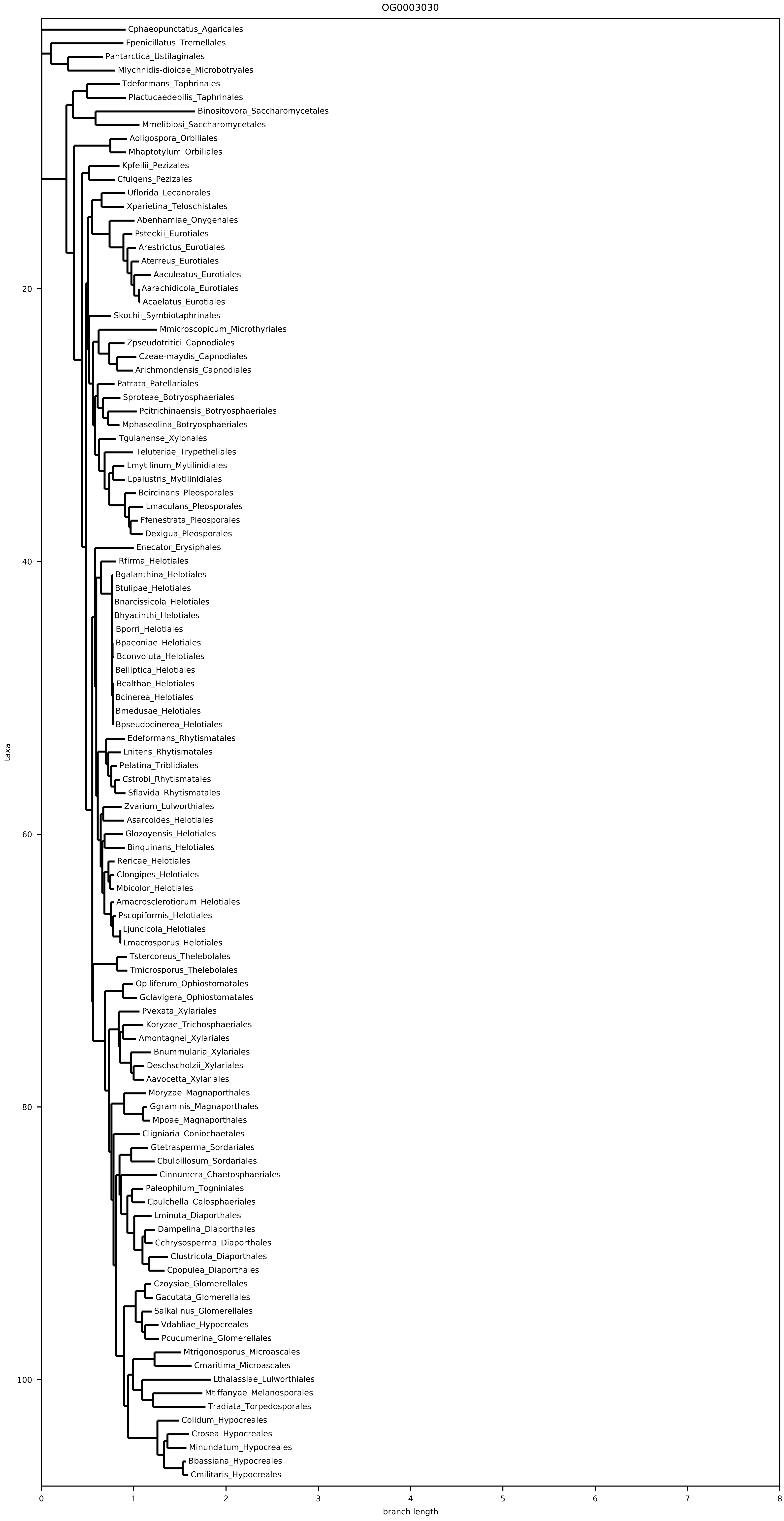

OG0003030
Cphaeopunctatus_Agaricales
Fpenicillatus_Tremellales
Pantarctica_Ustilaginales
Mlychnidis-dioicae_Microbotryales
Tdeformans_Taphrinales
Plactucaedebilis_Taphrinales
Binositovora_Saccharomycetales
Mmelibiosi_Saccharomycetales
Aoligospora_Orbiliales
Mhaptotylum_Orbiliales
Kpfeilii_Pezizales
Cfulgens_Pezizales
Uflorida_Lecanorales
Xparietina_Teloschistales
Abenhamiae_Onygenales
Psteckii_Eurotiales
Arestrictus_Eurotiales
Aterreus_Eurotiales
Aaculeatus_Eurotiales
Aarachidicola_Eurotiales
Acaelatus_Eurotiales
Skochii_Symbiotaphrinales
Mmicroscopicum_Microthyriales
Zpseudotritici_Capnodiales
Czeae-maydis_Capnodiales
Arichmondensis_Capnodiales
Patrata_Patellariales
Sproteae_Botryosphaeriales
Pcitrichinaensis_Botryosphaeriales
Mphaseolina_Botryosphaeriales
Tguianense_Xylonales
Teluteriae_Trypetheliales
Lmytilinum_Mytilinidiales
Lpalustris_Mytilinidiales
Bcircinans_Pleosporales
Lmaculans_Pleosporales
Ffenestrata_Pleosporales
Dexigua_Pleosporales
Enecator_Erysiphales
Rfirma_Helotiales
Bgalanthina_Helotiales
Btulipae_Helotiales
Bnarcissicola_Helotiales
Bhyacinthi_Helotiales
Bporri_Helotiales
Bpaeoniae_Helotiales
Bconvoluta_Helotiales
Belliptica_Helotiales
Bcalthae_Helotiales
Bcinerea_Helotiales
Bmedusae_Helotiales
Bpseudocinerea_Helotiales
Edeformans_Rhytismatales
Lnitens_Rhytismatales
Pelatina_Triblidiales
Cstrobi_Rhytismatales
Sflavida_Rhytismatales
Zvarium_Lulworthiales
Asarcoides_Helotiales
Glozoyensis_Helotiales
Binquinans_Helotiales
Rericae_Helotiales
Clongipes_Helotiales
Mbicolor_Helotiales
Amacrosclerotiorum_Helotiales
Pscopiformis_Helotiales
Ljuncicola_Helotiales
Lmacrosporus_Helotiales
Tstercoreus_Thelebolales
Tmicrosporus_Thelebolales
Opiliferum_Ophiostomatales
Gclavigera_Ophiostomatales
Pvexata_Xylariales
Koryzae_Trichosphaeriales
Amontagnei_Xylariales
Bnummularia_Xylariales
Deschscholzii_Xylariales
Aavocetta_Xylariales
Moryzae_Magnaporthales
Ggraminis_Magnaporthales
Mpoae_Magnaporthales
Cligniaria_Coniochaetales
Gtetrasperma_Sordariales
Cbulbillosum_Sordariales
Cinnumera_Chaetosphaeriales
Paleophilum_Togniniales
Cpulchella_Calosphaeriales
Lminuta_Diaporthales
Dampelina_Diaporthales
Cchrysosperma_Diaporthales
Clustricola_Diaporthales
Cpopulea_Diaporthales
Czoysiae_Glomerellales
Gacutata_Glomerellales
Salkalinus_Glomerellales
Vdahliae_Hypocreales
Pcucumerina_Glomerellales
Mtrigonosporus_Microascales
Cmaritima_Microascales
Lthalassiae_Lulworthiales
Mtiffanyae_Melanosporales
Tradiata_Torpedosporales
Colidum_Hypocreales
Crosea_Hypocreales
Minundatum_Hypocreales
Bbassiana_Hypocreales
Cmilitaris_Hypocreales
taxa
20
40
60
80
100
0
1
2
3
4
5
6
7
8
branch length

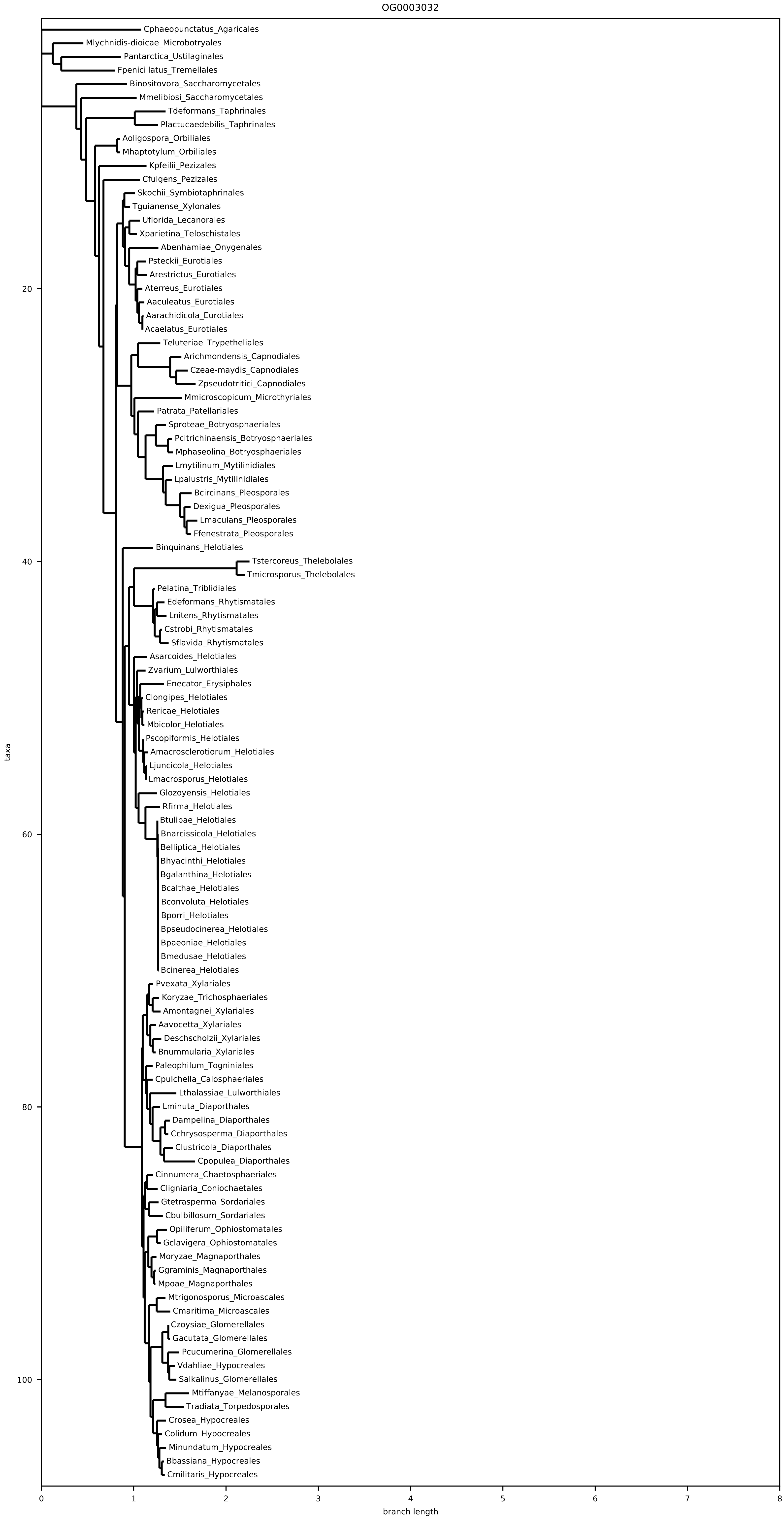
OG0003032
Cphaeopunctatus_Agaricales
Mlychnidis-dioicae_Microbotryales
Pantarctica_Ustilaginales
Fpenicillatus_Tremellales
Binositovora_Saccharomycetales
Mmelibiosi_Saccharomycetales
Tdeformans_Taphrinales
Plactucaedebilis_Taphrinales
Aoligospora_Orbiliales
Mhaptotylum_Orbiliales
Kpfeilii_Pezizales
Cfulgens_Pezizales
Skochii_Symbiotaphrinales
Tguianense_Xylonales
Uflorida_Lecanorales
Xparietina_Teloschistales
Abenhamiae_Onygenales
Psteckii_Eurotiales
Arestrictus_Eurotiales
Aterreus_Eurotiales
Aaculeatus_Eurotiales
Aarachidicola_Eurotiales
Acaelatus_Eurotiales
Teluteriae_Trypetheliales
Arichmondensis_Capnodiales
Czeae-maydis_Capnodiales
Zpseudotritici_Capnodiales
Mmicroscopicum_Microthyriales
Patrata_Patellariales
Sproteae_Botryosphaeriales
Pcitrichinaensis_Botryosphaeriales
Mphaseolina_Botryosphaeriales
Lmytilinum_Mytilinidiales
Lpalustris_Mytilinidiales
Bcircinans_Pleosporales
Dexigua_Pleosporales
Lmaculans_Pleosporales
Ffenestrata_Pleosporales
Binquinans_Helotiales
Tstercoreus_Thelebolales
Tmicrosporus_Thelebolales
Pelatina_Triblidiales
Edeformans_Rhytismatales
Lnitens_Rhytismatales
Cstrobi_Rhytismatales
Sflavida_Rhytismatales
Asarcoides_Helotiales
Zvarium_Lulworthiales
Enecator_Erysiphales
Clongipes_Helotiales
Rericae_Helotiales
Mbicolor_Helotiales
Pscopiformis_Helotiales
Amacrosclerotiorum_Helotiales
Ljuncicola_Helotiales
Lmacrosporus_Helotiales
Glozoyensis_Helotiales
Rfirma_Helotiales
Btulipae_Helotiales
Bnarcissicola_Helotiales
Belliptica_Helotiales
Bhyacinthi_Helotiales
Bgalanthina_Helotiales
Bcalthae_Helotiales
Bconvoluta_Helotiales
Bporri_Helotiales
Bpseudocinerea_Helotiales
Bpaeoniae_Helotiales
Bmedusae_Helotiales
Bcinerea_Helotiales
Pvexata_Xylariales
Koryzae_Trichosphaeriales
Amontagnei_Xylariales
Aavocetta_Xylariales
Deschscholzii_Xylariales
Bnummularia_Xylariales
Paleophilum_Togniniales
Cpulchella_Calosphaeriales
Lthalassiae_Lulworthiales
Lminuta_Diaporthales
Dampelina_Diaporthales
Cchrysosperma_Diaporthales
Clustricola_Diaporthales
Cpopulea_Diaporthales
Cinnumera_Chaetosphaeriales
Cligniaria_Coniochaetales
Gtetrasperma_Sordariales
Cbulbillosum_Sordariales
Opiliferum_Ophiostomatales
Gclavigera_Ophiostomatales
Moryzae_Magnaporthales
Ggraminis_Magnaporthales
Mpoae_Magnaporthales
Mtrigonosporus_Microascales
Cmaritima_Microascales
Czoysiae_Glomerellales
Gacutata_Glomerellales
Pcucumerina_Glomerellales
Vdahliae_Hypocreales
Salkalinus_Glomerellales
Mtiffanyae_Melanosporales
Tradiata_Torpedosporales
Crosea_Hypocreales
Colidum_Hypocreales
Minundatum_Hypocreales
Bbassiana_Hypocreales
Cmilitaris_Hypocreales
taxa
20
40
60
80
100
0
1
2
3
4
5
6
7
8
branch length

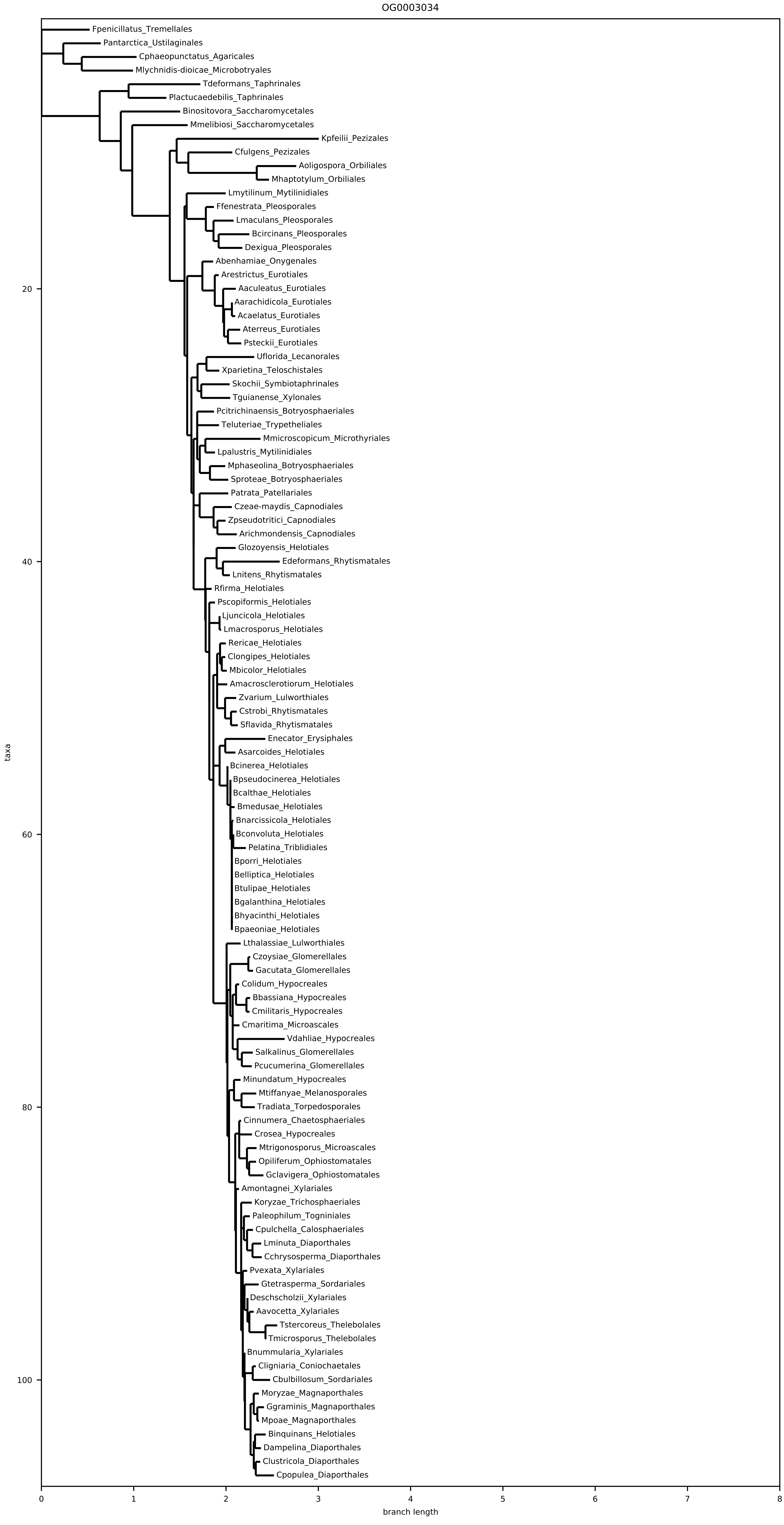
OG0003034
Fpenicillatus_Tremellales
Pantarctica_Ustilaginales
Cphaeopunctatus_Agaricales
Mlychnidis-dioicae_Microbotryales
Tdeformans_Taphrinales
Plactucaedebilis_Taphrinales
Binositovora_Saccharomycetales
Mmelibiosi_Saccharomycetales
Kpfeilii_Pezizales
Cfulgens_Pezizales
Aoligospora_Orbiliales
Mhaptotylum_Orbiliales
Lmytilinum_Mytilinidiales
Ffenestrata_Pleosporales
Lmaculans_Pleosporales
Bcircinans_Pleosporales
Dexigua_Pleosporales
Abenhamiae_Onygenales
Arestrictus_Eurotiales
Aaculeatus_Eurotiales
Aarachidicola_Eurotiales
Acaelatus_Eurotiales
Aterreus_Eurotiales
Psteckii_Eurotiales
Uflorida_Lecanorales
Xparietina_Teloschistales
Skochii_Symbiotaphrinales
Tguianense_Xylonales
Pcitrichinaensis_Botryosphaeriales
Teluteriae_Trypetheliales
Mmicroscopicum_Microthyriales
Lpalustris_Mytilinidiales
Mphaseolina_Botryosphaeriales
Sproteae_Botryosphaeriales
Patrata_Patellariales
Czeae-maydis_Capnodiales
Zpseudotritici_Capnodiales
Arichmondensis_Capnodiales
Glozoyensis_Helotiales
Edeformans_Rhytismatales
Lnitens_Rhytismatales
Rfirma_Helotiales
Pscopiformis_Helotiales
Ljuncicola_Helotiales
Lmacrosporus_Helotiales
Rericae_Helotiales
Clongipes_Helotiales
Mbicolor_Helotiales
Amacrosclerotiorum_Helotiales
Zvarium_Lulworthiales
Cstrobi_Rhytismatales
Sflavida_Rhytismatales
Enecator_Erysiphales
Asarcoides_Helotiales
Bcinerea_Helotiales
Bpseudocinerea_Helotiales
Bcalthae_Helotiales
Bmedusae_Helotiales
Bnarcissicola_Helotiales
Bconvoluta_Helotiales
Pelatina_Triblidiales
Bporri_Helotiales
Belliptica_Helotiales
Btulipae_Helotiales
Bgalanthina_Helotiales
Bhyacinthi_Helotiales
Bpaeoniae_Helotiales
Lthalassiae_Lulworthiales
Czoysiae_Glomerellales
Gacutata_Glomerellales
Colidum_Hypocreales
Bbassiana_Hypocreales
Cmilitaris_Hypocreales
Cmaritima_Microascales
Vdahliae_Hypocreales
Salkalinus_Glomerellales
Pcucumerina_Glomerellales
Minundatum_Hypocreales
Mtiffanyae_Melanosporales
Tradiata_Torpedosporales
Cinnumera_Chaetosphaeriales
Crosea_Hypocreales
Mtrigonosporus_Microascales
Opiliferum_Ophiostomatales
Gclavigera_Ophiostomatales
Amontagnei_Xylariales
Koryzae_Trichosphaeriales
Paleophilum_Togniniales
Cpulchella_Calosphaeriales
Lminuta_Diaporthales
Cchrysosperma_Diaporthales
Pvexata_Xylariales
Gtetrasperma_Sordariales
Deschscholzii_Xylariales
Aavocetta_Xylariales
Tstercoreus_Thelebolales
Tmicrosporus_Thelebolales
Bnummularia_Xylariales
Cligniaria_Coniochaetales
Cbulbillosum_Sordariales
Moryzae_Magnaporthales
Ggraminis_Magnaporthales
Mpoae_Magnaporthales
Binquinans_Helotiales
Dampelina_Diaporthales
Clustricola_Diaporthales
Cpopulea_Diaporthales
taxa
20
40
60
80
100
0
1
2
3
4
5
6
7
8
branch length

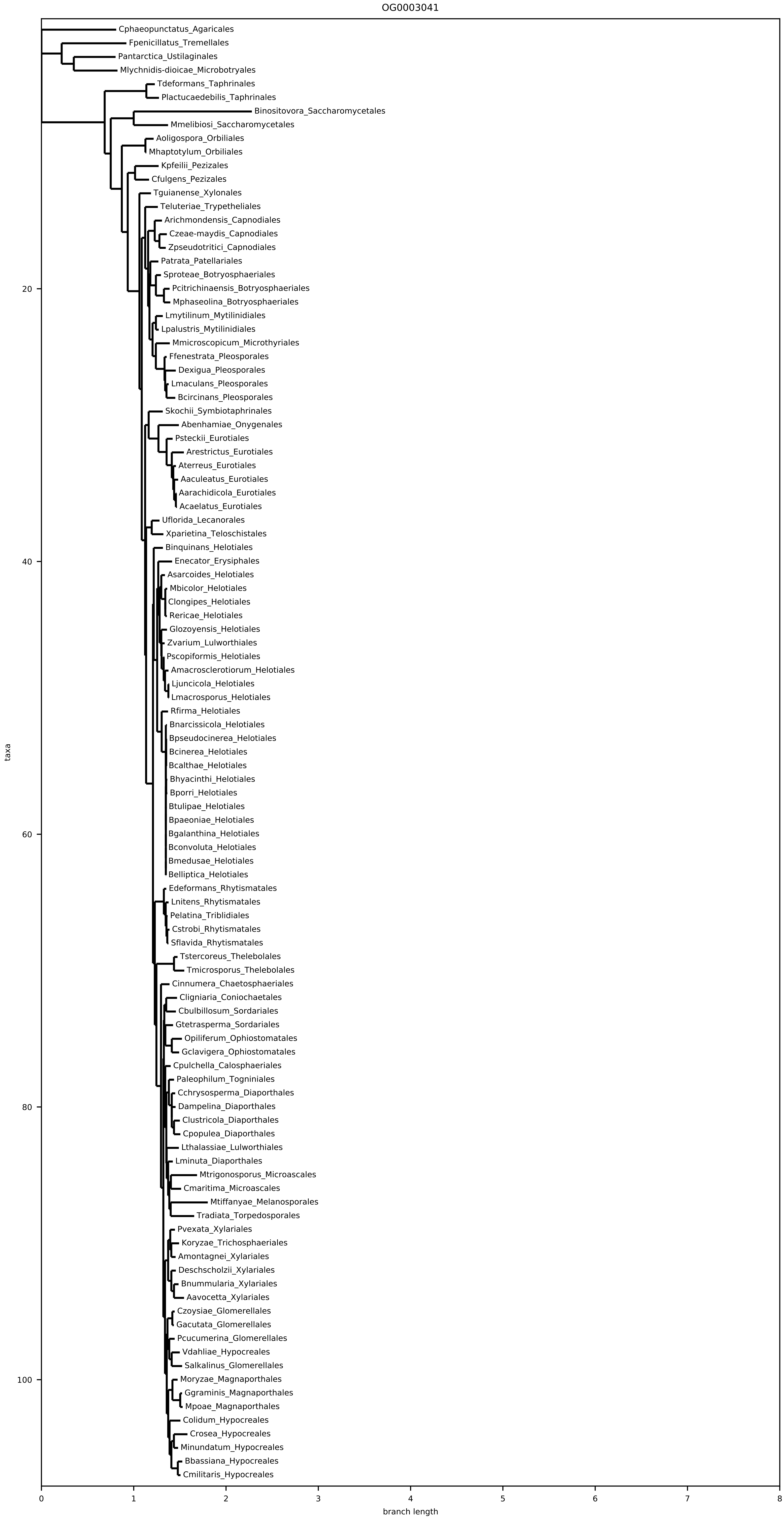

OG0003041
Cphaeopunctatus_Agaricales
Fpenicillatus_Tremellales
Pantarctica_Ustilaginales
Mlychnidis-dioicae_Microbotryales
Tdeformans_Taphrinales
Plactucaedebilis_Taphrinales
Binositovora_Saccharomycetales
Mmelibiosi_Saccharomycetales
Aoligospora_Orbiliales
Mhaptotylum_Orbiliales
Kpfeilii_Pezizales
Cfulgens_Pezizales
Tguianense_Xylonales
Teluteriae_Trypetheliales
Arichmondensis_Capnodiales
Czeae-maydis_Capnodiales
Zpseudotritici_Capnodiales
Patrata_Patellariales
Sproteae_Botryosphaeriales
Pcitrichinaensis_Botryosphaeriales
Mphaseolina_Botryosphaeriales
Lmytilinum_Mytilinidiales
Lpalustris_Mytilinidiales
Mmicroscopicum_Microthyriales
Ffenestrata_Pleosporales
Dexigua_Pleosporales
Lmaculans_Pleosporales
Bcircinans_Pleosporales
Skochii_Symbiotaphrinales
Abenhamiae_Onygenales
Psteckii_Eurotiales
Arestrictus_Eurotiales
Aterreus_Eurotiales
Aaculeatus_Eurotiales
Aarachidicola_Eurotiales
Acaelatus_Eurotiales
Uflorida_Lecanorales
Xparietina_Teloschistales
Binquinans_Helotiales
Enecator_Erysiphales
Asarcoides_Helotiales
Mbicolor_Helotiales
Clongipes_Helotiales
Rericae_Helotiales
Glozoyensis_Helotiales
Zvarium_Lulworthiales
Pscopiformis_Helotiales
Amacrosclerotiorum_Helotiales
Ljuncicola_Helotiales
Lmacrosporus_Helotiales
Rfirma_Helotiales
Bnarcissicola_Helotiales
Bpseudocinerea_Helotiales
Bcinerea_Helotiales
Bcalthae_Helotiales
Bhyacinthi_Helotiales
Bporri_Helotiales
Btulipae_Helotiales
Bpaeoniae_Helotiales
Bgalanthina_Helotiales
Bconvoluta_Helotiales
Bmedusae_Helotiales
Belliptica_Helotiales
Edeformans_Rhytismatales
Lnitens_Rhytismatales
Pelatina_Triblidiales
Cstrobi_Rhytismatales
Sflavida_Rhytismatales
Tstercoreus_Thelebolales
Tmicrosporus_Thelebolales
Cinnumera_Chaetosphaeriales
Cligniaria_Coniochaetales
Cbulbillosum_Sordariales
Gtetrasperma_Sordariales
Opiliferum_Ophiostomatales
Gclavigera_Ophiostomatales
Cpulchella_Calosphaeriales
Paleophilum_Togniniales
Cchrysosperma_Diaporthales
Dampelina_Diaporthales
Clustricola_Diaporthales
Cpopulea_Diaporthales
Lthalassiae_Lulworthiales
Lminuta_Diaporthales
Mtrigonosporus_Microascales
Cmaritima_Microascales
Mtiffanyae_Melanosporales
Tradiata_Torpedosporales
Pvexata_Xylariales
Koryzae_Trichosphaeriales
Amontagnei_Xylariales
Deschscholzii_Xylariales
Bnummularia_Xylariales
Aavocetta_Xylariales
Czoysiae_Glomerellales
Gacutata_Glomerellales
Pcucumerina_Glomerellales
Vdahliae_Hypocreales
Salkalinus_Glomerellales
Moryzae_Magnaporthales
Ggraminis_Magnaporthales
Mpoae_Magnaporthales
Colidum_Hypocreales
Crosea_Hypocreales
Minundatum_Hypocreales
Bbassiana_Hypocreales
Cmilitaris_Hypocreales
taxa
20
40
60
80
100
0
1
2
3
4
5
6
7
8
branch length

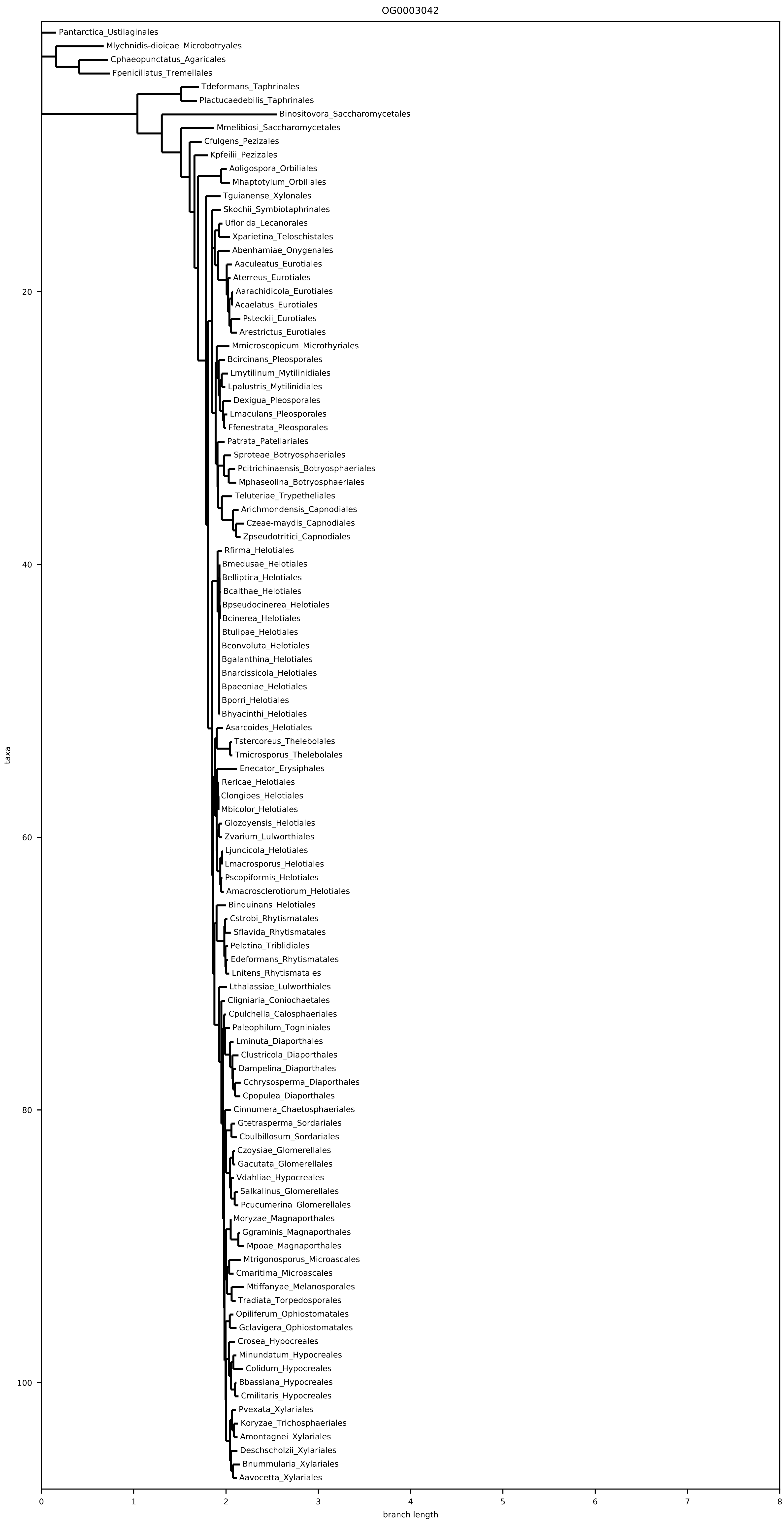
OG0003042
taxa
branch length
0
1
2
3
4
5
6
7
8
20
40
60
80
100
Pantarctica_Ustilaginales
Mlychnidis-dioicae_Microbotryales
Cphaeopunctatus_Agaricales
Fpenicillatus_Tremellales
Tdeformans_Taphrinales
Plactucaedebilis_Taphrinales
Binositovora_Saccharomycetales
Mmelibiosi_Saccharomycetales
Cfulgens_Pezizales
Kpfeilii_Pezizales
Aoligospora_Orbiliales
Mhaptotylum_Orbiliales
Tguianense_Xylonales
Skochii_Symbiotaphrinales
Uflorida_Lecanorales
Xparietina_Teloschistales
Abenhamiae_Onygenales
Aaculeatus_Eurotiales
Aterreus_Eurotiales
Aarachidicola_Eurotiales
Acaelatus_Eurotiales
Psteckii_Eurotiales
Arestrictus_Eurotiales
Mmicroscopicum_Microthyriales
Bcircinans_Pleosporales
Lmytilinum_Mytilinidiales
Lpalustris_Mytilinidiales
Dexigua_Pleosporales
Lmaculans_Pleosporales
Ffenestrata_Pleosporales
Patrata_Patellariales
Sproteae_Botryosphaeriales
Pcitrichinaensis_Botryosphaeriales
Mphaseolina_Botryosphaeriales
Teluteriae_Trypetheliales
Arichmondensis_Capnodiales
Czeae-maydis_Capnodiales
Zpseudotritici_Capnodiales
Rfirma_Helotiales
Bmedusae_Helotiales
Belliptica_Helotiales
Bcalthae_Helotiales
Bpseudocinerea_Helotiales
Bcinerea_Helotiales
Btulipae_Helotiales
Bconvoluta_Helotiales
Bgalanthina_Helotiales
Bnarcissicola_Helotiales
Bpaeoniae_Helotiales
Bporri_Helotiales
Bhyacinthi_Helotiales
Asarcoides_Helotiales
Tstercoreus_Thelebolales
Tmicrosporus_Thelebolales
Enecator_Erysiphales
Rericae_Helotiales
Clongipes_Helotiales
Mbicolor_Helotiales
Glozoyensis_Helotiales
Zvarium_Lulworthiales
Ljuncicola_Helotiales
Lmacrosporus_Helotiales
Pscopiformis_Helotiales
Amacrosclerotiorum_Helotiales
Binquinans_Helotiales
Cstrobi_Rhytismatales
Sflavida_Rhytismatales
Pelatina_Triblidiales
Edeformans_Rhytismatales
Lnitens_Rhytismatales
Lthalassiae_Lulworthiales
Cligniaria_Coniochaetales
Cpulchella_Calosphaeriales
Paleophilum_Togniniales
Lminuta_Diaporthales
Clustricola_Diaporthales
Dampelina_Diaporthales
Cchrysosperma_Diaporthales
Cpopulea_Diaporthales
Cinnumera_Chaetosphaeriales
Gtetrasperma_Sordariales
Cbulbillosum_Sordariales
Czoysiae_Glomerellales
Gacutata_Glomerellales
Vdahliae_Hypocreales
Salkalinus_Glomerellales
Pcucumerina_Glomerellales
Moryzae_Magnaporthales
Ggraminis_Magnaporthales
Mpoae_Magnaporthales
Mtrigonosporus_Microascales
Cmaritima_Microascales
Mtiffanyae_Melanosporales
Tradiata_Torpedosporales
Opiliferum_Ophiostomatales
Gclavigera_Ophiostomatales
Crosea_Hypocreales
Minundatum_Hypocreales
Colidum_Hypocreales
Bbassiana_Hypocreales
Cmilitaris_Hypocreales
Pvexata_Xylariales
Koryzae_Trichosphaeriales
Amontagnei_Xylariales
Deschscholzii_Xylariales
Bnummularia_Xylariales
Aavocetta_Xylariales

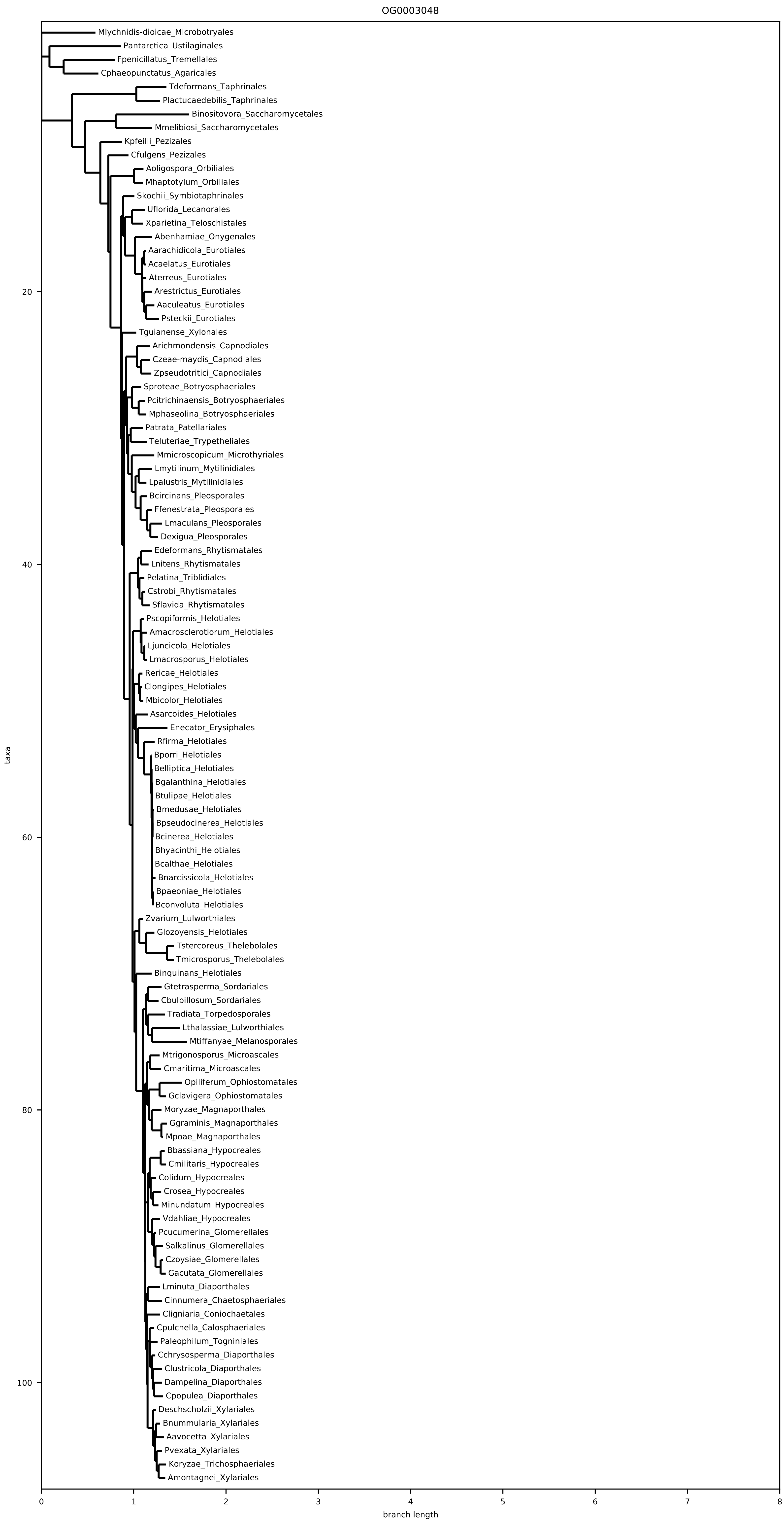
OG0003048
Mlychnidis-dioicae_Microbotryales
Pantarctica_Ustilaginales
Fpenicillatus_Tremellales
Cphaeopunctatus_Agaricales
Tdeformans_Taphrinales
Plactucaedebilis_Taphrinales
Binositovora_Saccharomycetales
Mmelibiosi_Saccharomycetales
Kpfeilii_Pezizales
Cfulgens_Pezizales
Aoligospora_Orbiliales
Mhaptotylum_Orbiliales
Skochii_Symbiotaphrinales
Uflorida_Lecanorales
Xparietina_Teloschistales
Abenhamiae_Onygenales
Aarachidicola_Eurotiales
Acaelatus_Eurotiales
Aterreus_Eurotiales
Arestrictus_Eurotiales
Aaculeatus_Eurotiales
Psteckii_Eurotiales
Tguianense_Xylonales
Arichmondensis_Capnodiales
Czeae-maydis_Capnodiales
Zpseudotritici_Capnodiales
Sproteae_Botryosphaeriales
Pcitrichinaensis_Botryosphaeriales
Mphaseolina_Botryosphaeriales
Patrata_Patellariales
Teluteriae_Trypetheliales
Mmicroscopicum_Microthyriales
Lmytilinum_Mytilinidiales
Lpalustris_Mytilinidiales
Bcircinans_Pleosporales
Ffenestrata_Pleosporales
Lmaculans_Pleosporales
Dexigua_Pleosporales
Edeformans_Rhytismatales
Lnitens_Rhytismatales
Pelatina_Triblidiales
Cstrobi_Rhytismatales
Sflavida_Rhytismatales
Pscopiformis_Helotiales
Amacrosclerotiorum_Helotiales
Ljuncicola_Helotiales
Lmacrosporus_Helotiales
Rericae_Helotiales
Clongipes_Helotiales
Mbicolor_Helotiales
Asarcoides_Helotiales
Enecator_Erysiphales
Rfirma_Helotiales
Bporri_Helotiales
Belliptica_Helotiales
Bgalanthina_Helotiales
Btulipae_Helotiales
Bmedusae_Helotiales
Bpseudocinerea_Helotiales
Bcinerea_Helotiales
Bhyacinthi_Helotiales
Bcalthae_Helotiales
Bnarcissicola_Helotiales
Bpaeoniae_Helotiales
Bconvoluta_Helotiales
Zvarium_Lulworthiales
Glozoyensis_Helotiales
Tstercoreus_Thelebolales
Tmicrosporus_Thelebolales
Binquinans_Helotiales
Gtetrasperma_Sordariales
Cbulbillosum_Sordariales
Tradiata_Torpedosporales
Lthalassiae_Lulworthiales
Mtiffanyae_Melanosporales
Mtrigonosporus_Microascales
Cmaritima_Microascales
Opiliferum_Ophiostomatales
Gclavigera_Ophiostomatales
Moryzae_Magnaporthales
Ggraminis_Magnaporthales
Mpoae_Magnaporthales
Bbassiana_Hypocreales
Cmilitaris_Hypocreales
Colidum_Hypocreales
Crosea_Hypocreales
Minundatum_Hypocreales
Vdahliae_Hypocreales
Pcucumerina_Glomerellales
Salkalinus_Glomerellales
Czoysiae_Glomerellales
Gacutata_Glomerellales
Lminuta_Diaporthales
Cinnumera_Chaetosphaeriales
Cligniaria_Coniochaetales
Cpulchella_Calosphaeriales
Paleophilum_Togniniales
Cchrysosperma_Diaporthales
Clustricola_Diaporthales
Dampelina_Diaporthales
Cpopulea_Diaporthales
Deschscholzii_Xylariales
Bnummularia_Xylariales
Aavocetta_Xylariales
Pvexata_Xylariales
Koryzae_Trichosphaeriales
Amontagnei_Xylariales
taxa
20
40
60
80
100
0
1
2
3
4
5
6
7
8
branch length

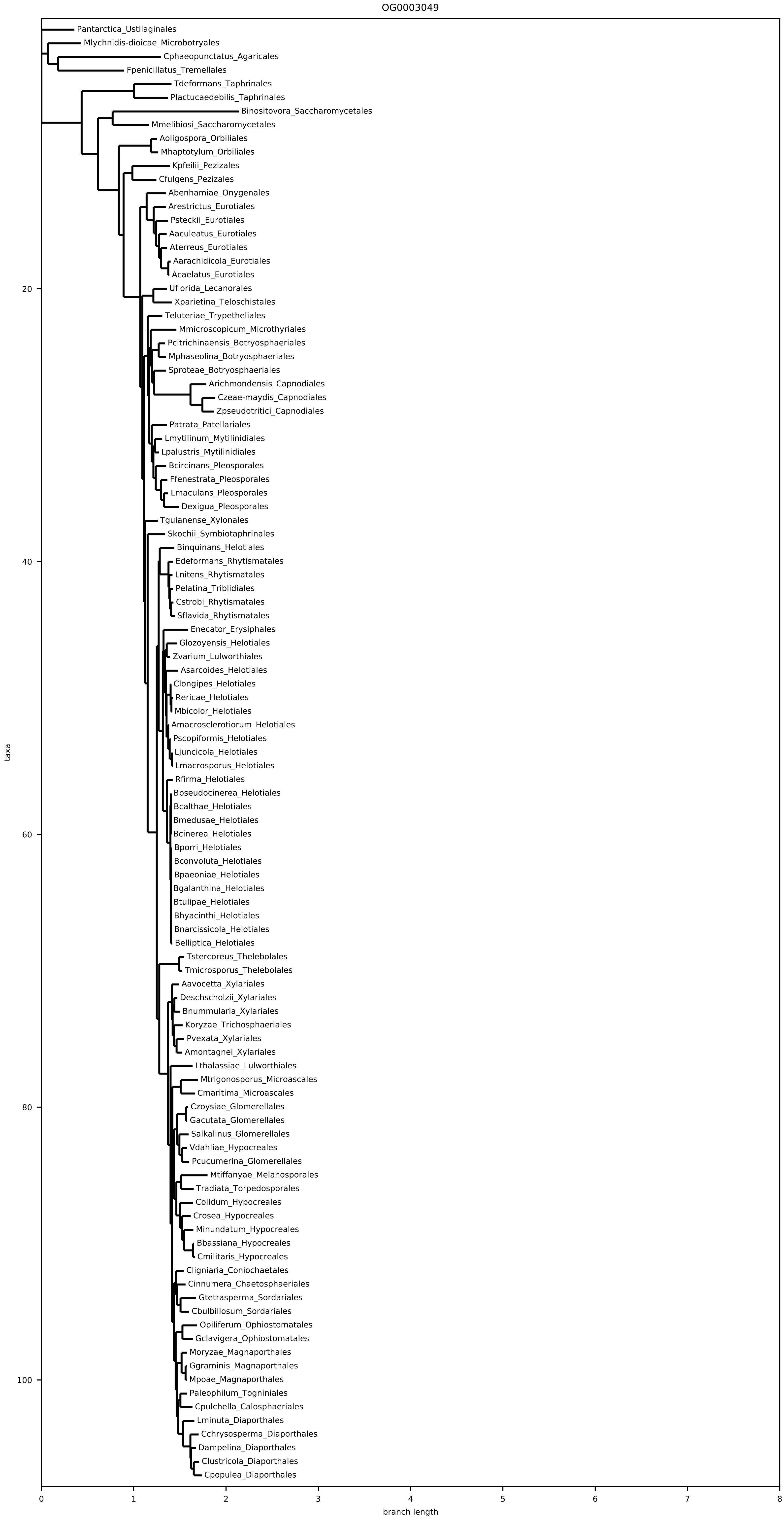
OG0003049
Pantarctica_Ustilaginales
Mlychnidis-dioicae_Microbotryales
Cphaeopunctatus_Agaricales
Fpenicillatus_Tremellales
Tdeformans_Taphrinales
Plactucaedebilis_Taphrinales
Binositovora_Saccharomycetales
Mmelibiosi_Saccharomycetales
Aoligospora_Orbiliales
Mhaptotylum_Orbiliales
Kpfeilii_Pezizales
Cfulgens_Pezizales
Abenhamiae_Onygenales
Arestrictus_Eurotiales
Psteckii_Eurotiales
Aaculeatus_Eurotiales
Aterreus_Eurotiales
Aarachidicola_Eurotiales
Acaelatus_Eurotiales
Uflorida_Lecanorales
Xparietina_Teloschistales
Teluteriae_Trypetheliales
Mmicroscopicum_Microthyriales
Pcitrichinaensis_Botryosphaeriales
Mphaseolina_Botryosphaeriales
Sproteae_Botryosphaeriales
Arichmondensis_Capnodiales
Czeae-maydis_Capnodiales
Zpseudotritici_Capnodiales
Patrata_Patellariales
Lmytilinum_Mytilinidiales
Lpalustris_Mytilinidiales
Bcircinans_Pleosporales
Ffenestrata_Pleosporales
Lmaculans_Pleosporales
Dexigua_Pleosporales
Tguianense_Xylonales
Skochii_Symbiotaphrinales
Binquinans_Helotiales
Edeformans_Rhytismatales
Lnitens_Rhytismatales
Pelatina_Triblidiales
Cstrobi_Rhytismatales
Sflavida_Rhytismatales
Enecator_Erysiphales
Glozoyensis_Helotiales
Zvarium_Lulworthiales
Asarcoides_Helotiales
Clongipes_Helotiales
Rericae_Helotiales
Mbicolor_Helotiales
Amacrosclerotiorum_Helotiales
Pscopiformis_Helotiales
Ljuncicola_Helotiales
Lmacrosporus_Helotiales
Rfirma_Helotiales
Bpseudocinerea_Helotiales
Bcalthae_Helotiales
Bmedusae_Helotiales
Bcinerea_Helotiales
Bporri_Helotiales
Bconvoluta_Helotiales
Bpaeoniae_Helotiales
Bgalanthina_Helotiales
Btulipae_Helotiales
Bhyacinthi_Helotiales
Bnarcissicola_Helotiales
Belliptica_Helotiales
Tstercoreus_Thelebolales
Tmicrosporus_Thelebolales
Aavocetta_Xylariales
Deschscholzii_Xylariales
Bnummularia_Xylariales
Koryzae_Trichosphaeriales
Pvexata_Xylariales
Amontagnei_Xylariales
Lthalassiae_Lulworthiales
Mtrigonosporus_Microascales
Cmaritima_Microascales
Czoysiae_Glomerellales
Gacutata_Glomerellales
Salkalinus_Glomerellales
Vdahliae_Hypocreales
Pcucumerina_Glomerellales
Mtiffanyae_Melanosporales
Tradiata_Torpedosporales
Colidum_Hypocreales
Crosea_Hypocreales
Minundatum_Hypocreales
Bbassiana_Hypocreales
Cmilitaris_Hypocreales
Cligniaria_Coniochaetales
Cinnumera_Chaetosphaeriales
Gtetrasperma_Sordariales
Cbulbillosum_Sordariales
Opiliferum_Ophiostomatales
Gclavigera_Ophiostomatales
Moryzae_Magnaporthales
Ggraminis_Magnaporthales
Mpoae_Magnaporthales
Paleophilum_Togniniales
Cpulchella_Calosphaeriales
Lminuta_Diaporthales
Cchrysosperma_Diaporthales
Dampelina_Diaporthales
Clustricola_Diaporthales
Cpopulea_Diaporthales
taxa
20
40
60
80
100
0
1
2
3
4
5
6
7
8
branch length

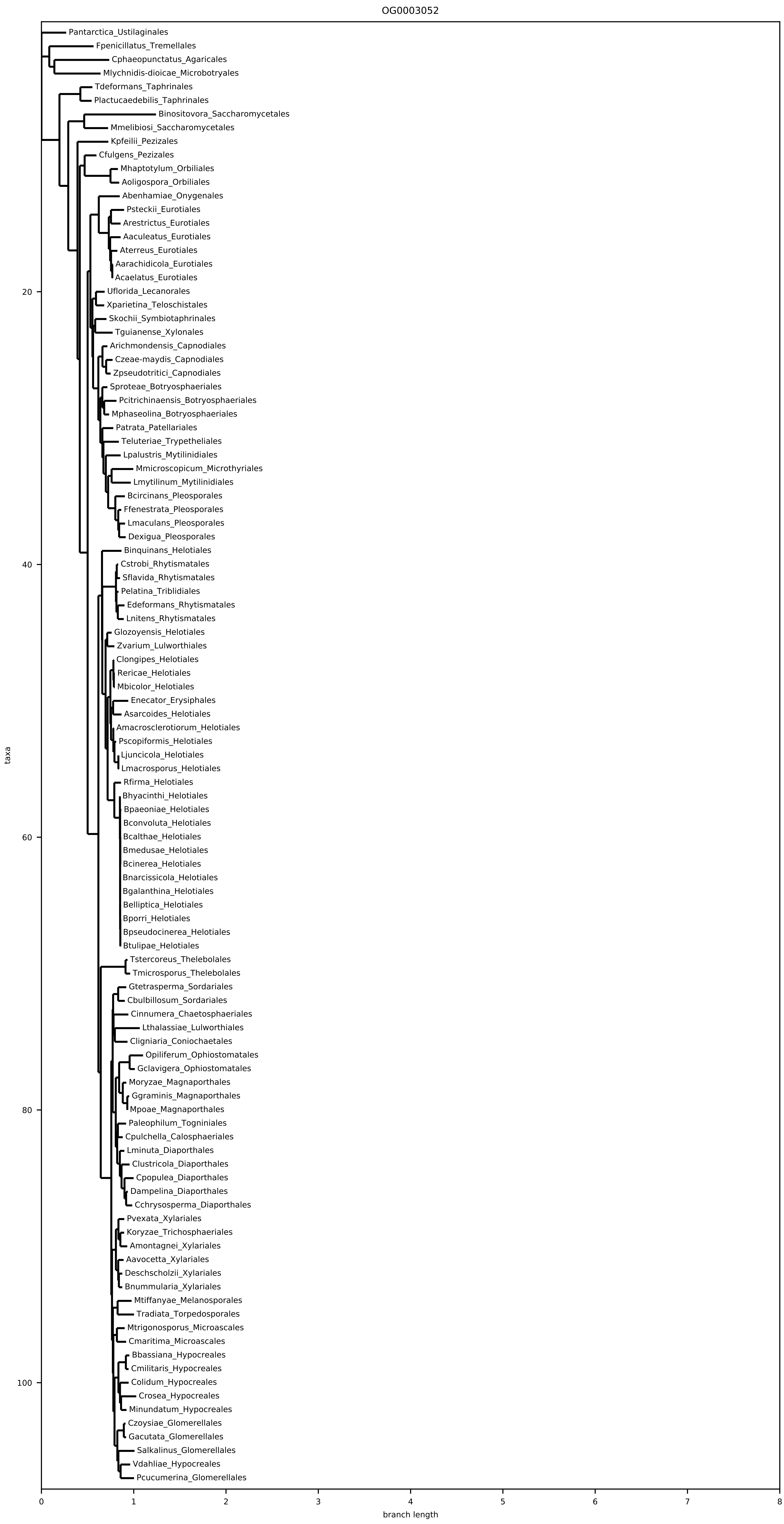
OG0003052
Pantarctica_Ustilaginales
Fpenicillatus_Tremellales
Cphaeopunctatus_Agaricales
Mlychnidis-dioicae_Microbotryales
Tdeformans_Taphrinales
Plactucaedebilis_Taphrinales
Binositovora_Saccharomycetales
Mmelibiosi_Saccharomycetales
Kpfeilii_Pezizales
Cfulgens_Pezizales
Mhaptotylum_Orbiliales
Aoligospora_Orbiliales
Abenhamiae_Onygenales
Psteckii_Eurotiales
Arestrictus_Eurotiales
Aaculeatus_Eurotiales
Aterreus_Eurotiales
Aarachidicola_Eurotiales
Acaelatus_Eurotiales
Uflorida_Lecanorales
Xparietina_Teloschistales
Skochii_Symbiotaphrinales
Tguianense_Xylonales
Arichmondensis_Capnodiales
Czeae-maydis_Capnodiales
Zpseudotritici_Capnodiales
Sproteae_Botryosphaeriales
Pcitrichinaensis_Botryosphaeriales
Mphaseolina_Botryosphaeriales
Patrata_Patellariales
Teluteriae_Trypetheliales
Lpalustris_Mytilinidiales
Mmicroscopicum_Microthyriales
Lmytilinum_Mytilinidiales
Bcircinans_Pleosporales
Ffenestrata_Pleosporales
Lmaculans_Pleosporales
Dexigua_Pleosporales
Binquinans_Helotiales
Cstrobi_Rhytismatales
Sflavida_Rhytismatales
Pelatina_Triblidiales
Edeformans_Rhytismatales
Lnitens_Rhytismatales
Glozoyensis_Helotiales
Zvarium_Lulworthiales
Clongipes_Helotiales
Rericae_Helotiales
Mbicolor_Helotiales
Enecator_Erysiphales
Asarcoides_Helotiales
Amacrosclerotiorum_Helotiales
Pscopiformis_Helotiales
Ljuncicola_Helotiales
Lmacrosporus_Helotiales
Rfirma_Helotiales
Bhyacinthi_Helotiales
Bpaeoniae_Helotiales
Bconvoluta_Helotiales
Bcalthae_Helotiales
Bmedusae_Helotiales
Bcinerea_Helotiales
Bnarcissicola_Helotiales
Bgalanthina_Helotiales
Belliptica_Helotiales
Bporri_Helotiales
Bpseudocinerea_Helotiales
Btulipae_Helotiales
Tstercoreus_Thelebolales
Tmicrosporus_Thelebolales
Gtetrasperma_Sordariales
Cbulbillosum_Sordariales
Cinnumera_Chaetosphaeriales
Lthalassiae_Lulworthiales
Cligniaria_Coniochaetales
Opiliferum_Ophiostomatales
Gclavigera_Ophiostomatales
Moryzae_Magnaporthales
Ggraminis_Magnaporthales
Mpoae_Magnaporthales
Paleophilum_Togniniales
Cpulchella_Calosphaeriales
Lminuta_Diaporthales
Clustricola_Diaporthales
Cpopulea_Diaporthales
Dampelina_Diaporthales
Cchrysosperma_Diaporthales
Pvexata_Xylariales
Koryzae_Trichosphaeriales
Amontagnei_Xylariales
Aavocetta_Xylariales
Deschscholzii_Xylariales
Bnummularia_Xylariales
Mtiffanyae_Melanosporales
Tradiata_Torpedosporales
Mtrigonosporus_Microascales
Cmaritima_Microascales
Bbassiana_Hypocreales
Cmilitaris_Hypocreales
Colidum_Hypocreales
Crosea_Hypocreales
Minundatum_Hypocreales
Czoysiae_Glomerellales
Gacutata_Glomerellales
Salkalinus_Glomerellales
Vdahliae_Hypocreales
Pcucumerina_Glomerellales
taxa
20
40
60
80
100
0
1
2
3
4
5
6
7
8
branch length

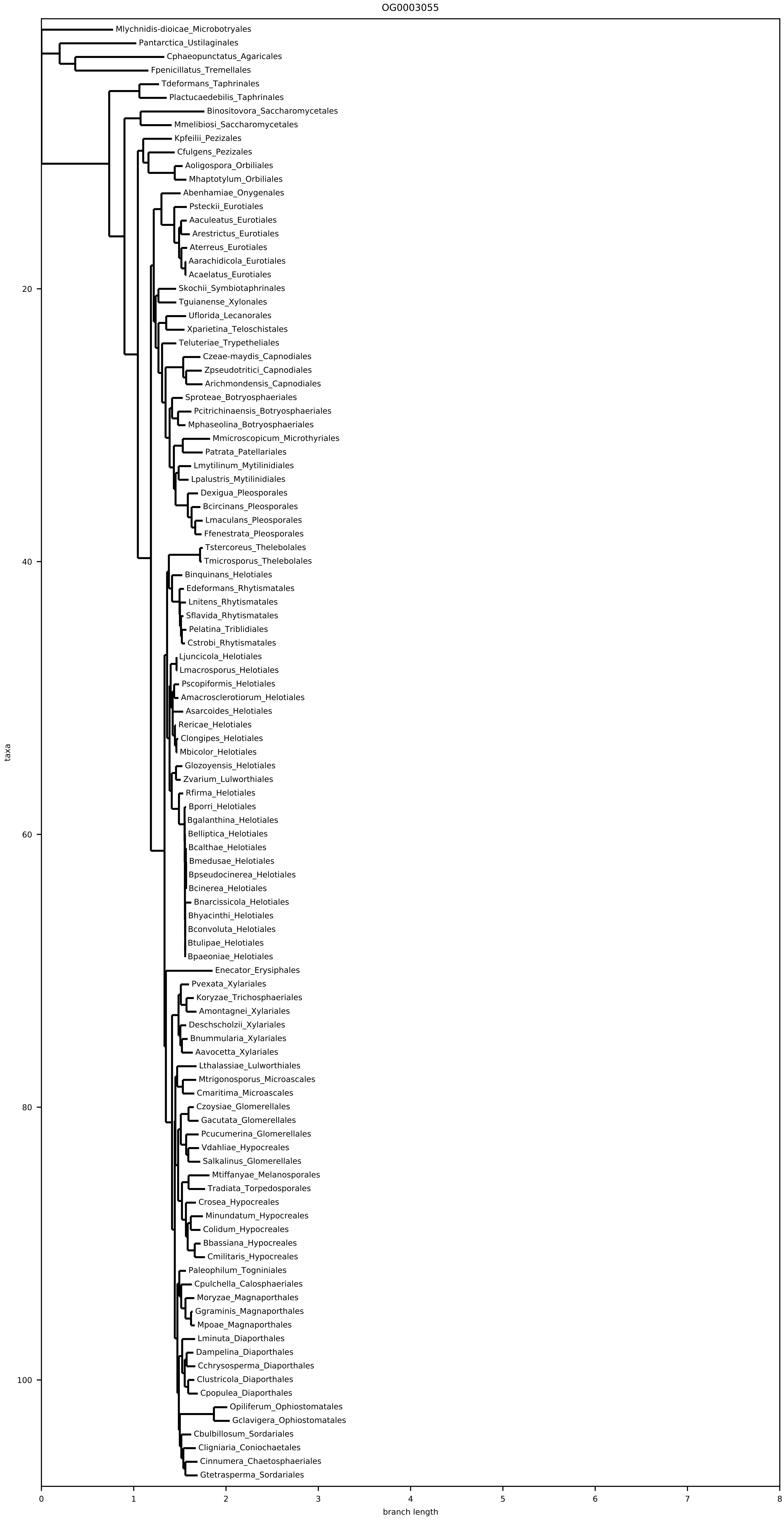
OG0003055
Mlychnidis-dioicae_Microbotryales
Pantarctica_Ustilaginales
Cphaeopunctatus_Agaricales
Fpenicillatus_Tremellales
Tdeformans_Taphrinales
Plactucaedebilis_Taphrinales
Binositovora_Saccharomycetales
Mmelibiosi_Saccharomycetales
Kpfeilii_Pezizales
Cfulgens_Pezizales
Aoligospora_Orbiliales
Mhaptotylum_Orbiliales
Abenhamiae_Onygenales
Psteckii_Eurotiales
Aaculeatus_Eurotiales
Arestrictus_Eurotiales
Aterreus_Eurotiales
Aarachidicola_Eurotiales
Acaelatus_Eurotiales
Skochii_Symbiotaphrinales
Tguianense_Xylonales
Uflorida_Lecanorales
Xparietina_Teloschistales
Teluteriae_Trypetheliales
Czeae-maydis_Capnodiales
Zpseudotritici_Capnodiales
Arichmondensis_Capnodiales
Sproteae_Botryosphaeriales
Pcitrichinaensis_Botryosphaeriales
Mphaseolina_Botryosphaeriales
Mmicroscopicum_Microthyriales
Patrata_Patellariales
Lmytilinum_Mytilinidiales
Lpalustris_Mytilinidiales
Dexigua_Pleosporales
Bcircinans_Pleosporales
Lmaculans_Pleosporales
Ffenestrata_Pleosporales
Tstercoreus_Thelebolales
Tmicrosporus_Thelebolales
Binquinans_Helotiales
Edeformans_Rhytismatales
Lnitens_Rhytismatales
Sflavida_Rhytismatales
Pelatina_Triblidiales
Cstrobi_Rhytismatales
Ljuncicola_Helotiales
Lmacrosporus_Helotiales
Pscopiformis_Helotiales
Amacrosclerotiorum_Helotiales
Asarcoides_Helotiales
Rericae_Helotiales
Clongipes_Helotiales
Mbicolor_Helotiales
Glozoyensis_Helotiales
Zvarium_Lulworthiales
Rfirma_Helotiales
Bporri_Helotiales
Bgalanthina_Helotiales
Belliptica_Helotiales
Bcalthae_Helotiales
Bmedusae_Helotiales
Bpseudocinerea_Helotiales
Bcinerea_Helotiales
Bnarcissicola_Helotiales
Bhyacinthi_Helotiales
Bconvoluta_Helotiales
Btulipae_Helotiales
Bpaeoniae_Helotiales
Enecator_Erysiphales
Pvexata_Xylariales
Koryzae_Trichosphaeriales
Amontagnei_Xylariales
Deschscholzii_Xylariales
Bnummularia_Xylariales
Aavocetta_Xylariales
Lthalassiae_Lulworthiales
Mtrigonosporus_Microascales
Cmaritima_Microascales
Czoysiae_Glomerellales
Gacutata_Glomerellales
Pcucumerina_Glomerellales
Vdahliae_Hypocreales
Salkalinus_Glomerellales
Mtiffanyae_Melanosporales
Tradiata_Torpedosporales
Crosea_Hypocreales
Minundatum_Hypocreales
Colidum_Hypocreales
Bbassiana_Hypocreales
Cmilitaris_Hypocreales
Paleophilum_Togniniales
Cpulchella_Calosphaeriales
Moryzae_Magnaporthales
Ggraminis_Magnaporthales
Mpoae_Magnaporthales
Lminuta_Diaporthales
Dampelina_Diaporthales
Cchrysosperma_Diaporthales
Clustricola_Diaporthales
Cpopulea_Diaporthales
Opiliferum_Ophiostomatales
Gclavigera_Ophiostomatales
Cbulbillosum_Sordariales
Cligniaria_Coniochaetales
Cinnumera_Chaetosphaeriales
Gtetrasperma_Sordariales
taxa
20
40
60
80
100
0
1
2
3
4
5
6
7
8
branch length

OG0003059

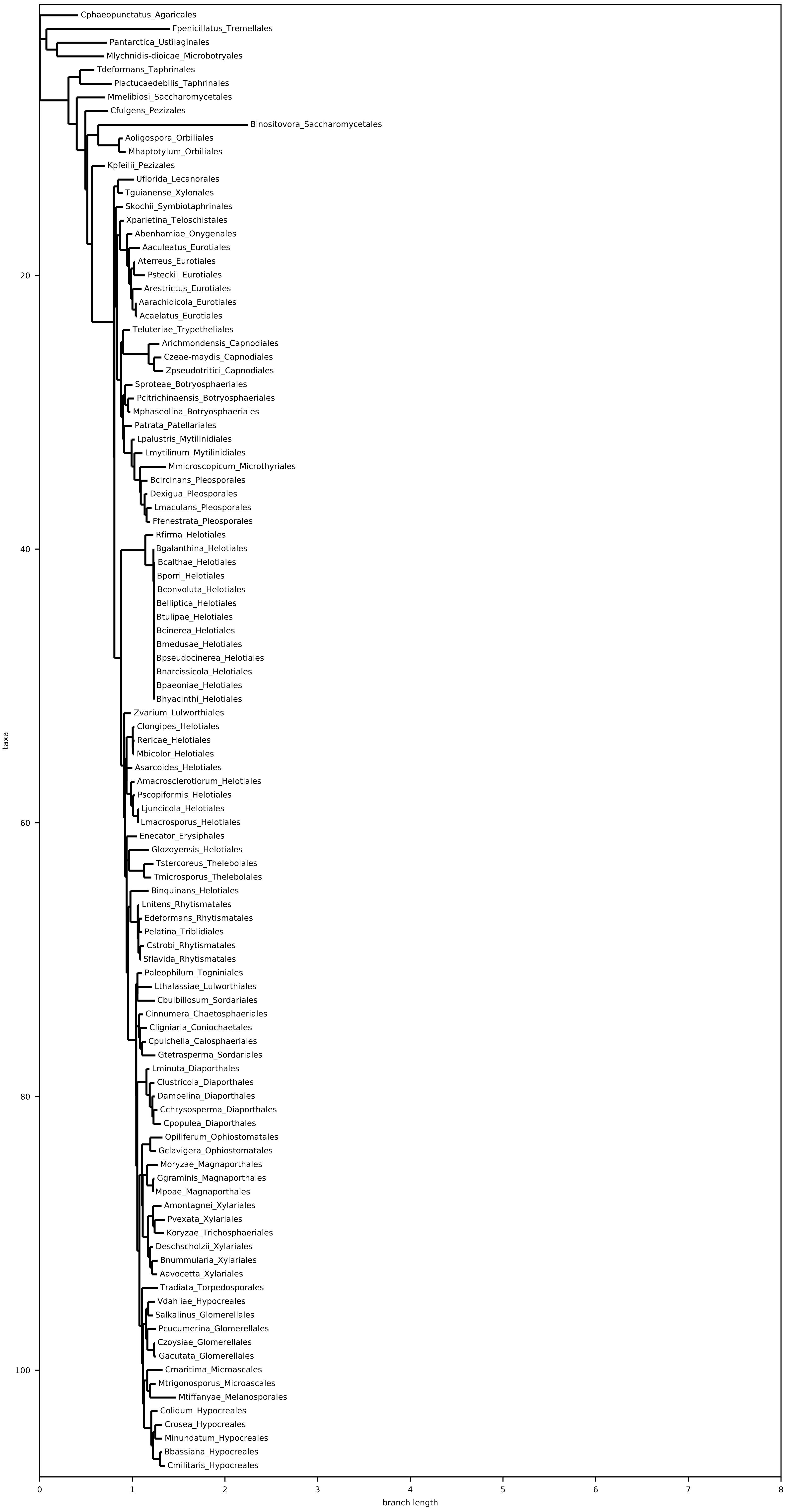
Cphaeopunctatus_Agaricales
Fpenicillatus_Tremellales
Pantarctica_Ustilaginales
Mlychnidis-dioicae_Microbotryales
Tdeformans_Taphrinales
Plactucaedebilis_Taphrinales
Mmelibiosi_Saccharomycetales
Cfulgens_Pezizales
Binositovora_Saccharomycetales
Aoligospora_Orbiliales
Mhaptotylum_Orbiliales
Kpfeilii_Pezizales
Uflorida_Lecanorales
Tguianense_Xylonales
Skochii_Symbiotaphrinales
Xparietina_Teloschistales
Abenhamiae_Onygenales
Aaculeatus_Eurotiales
Aterreus_Eurotiales
Psteckii_Eurotiales
Arestrictus_Eurotiales
Aarachidicola_Eurotiales
Acaelatus_Eurotiales
Teluteriae_Trypetheliales
Arichmondensis_Capnodiales
Czeae-maydis_Capnodiales
Zpseudotritici_Capnodiales
Sproteae_Botryosphaeriales
Pcitrichinaensis_Botryosphaeriales
Mphaseolina_Botryosphaeriales
Patrata_Patellariales
Lpalustris_Mytilinidiales
Lmytilinum_Mytilinidiales
Mmicroscopicum_Microthyriales
Bcircinans_Pleosporales
Dexigua_Pleosporales
Lmaculans_Pleosporales
Ffenestrata_Pleosporales
Rfirma_Helotiales
Bgalanthina_Helotiales
Bcalthae_Helotiales
Bporri_Helotiales
Bconvoluta_Helotiales
Belliptica_Helotiales
Btulipae_Helotiales
Bcinerea_Helotiales
Bmedusae_Helotiales
Bpseudocinerea_Helotiales
Bnarcissicola_Helotiales
Bpaeoniae_Helotiales
Bhyacinthi_Helotiales
Zvarium_Lulworthiales
Clongipes_Helotiales
Rericae_Helotiales
Mbicolor_Helotiales
Asarcoides_Helotiales
Amacrosclerotiorum_Helotiales
Pscopiformis_Helotiales
Ljuncicola_Helotiales
Lmacrosporus_Helotiales
Enecator_Erysiphales
Glozoyensis_Helotiales
Tstercoreus_Thelebolales
Tmicrosporus_Thelebolales
Binquinans_Helotiales
Lnitens_Rhytismatales
Edeformans_Rhytismatales
Pelatina_Triblidiales
Cstrobi_Rhytismatales
Sflavida_Rhytismatales
Paleophilum_Togniniales
Lthalassiae_Lulworthiales
Cbulbillosum_Sordariales
Cinnumera_Chaetosphaeriales
Cligniaria_Coniochaetales
Cpulchella_Calosphaeriales
Gtetrasperma_Sordariales
Lminuta_Diaporthales
Clustricola_Diaporthales
Dampelina_Diaporthales
Cchrysosperma_Diaporthales
Cpopulea_Diaporthales
Opiliferum_Ophiostomatales
Gclavigera_Ophiostomatales
Moryzae_Magnaporthales
Ggraminis_Magnaporthales
Mpoae_Magnaporthales
Amontagnei_Xylariales
Pvexata_Xylariales
Koryzae_Trichosphaeriales
Deschscholzii_Xylariales
Bnummularia_Xylariales
Aavocetta_Xylariales
Tradiata_Torpedosporales
Vdahliae_Hypocreales
Salkalinus_Glomerellales
Pcucumerina_Glomerellales
Czoysiae_Glomerellales
Gacutata_Glomerellales
Cmaritima_Microascales
Mtrigonosporus_Microascales
Mtiffanyae_Melanosporales
Colidum_Hypocreales
Crosea_Hypocreales
Minundatum_Hypocreales
Bbassiana_Hypocreales
Cmilitaris_Hypocreales
taxa
20
40
60
80
100
0
1
2
3
4
5
6
7
8
branch length

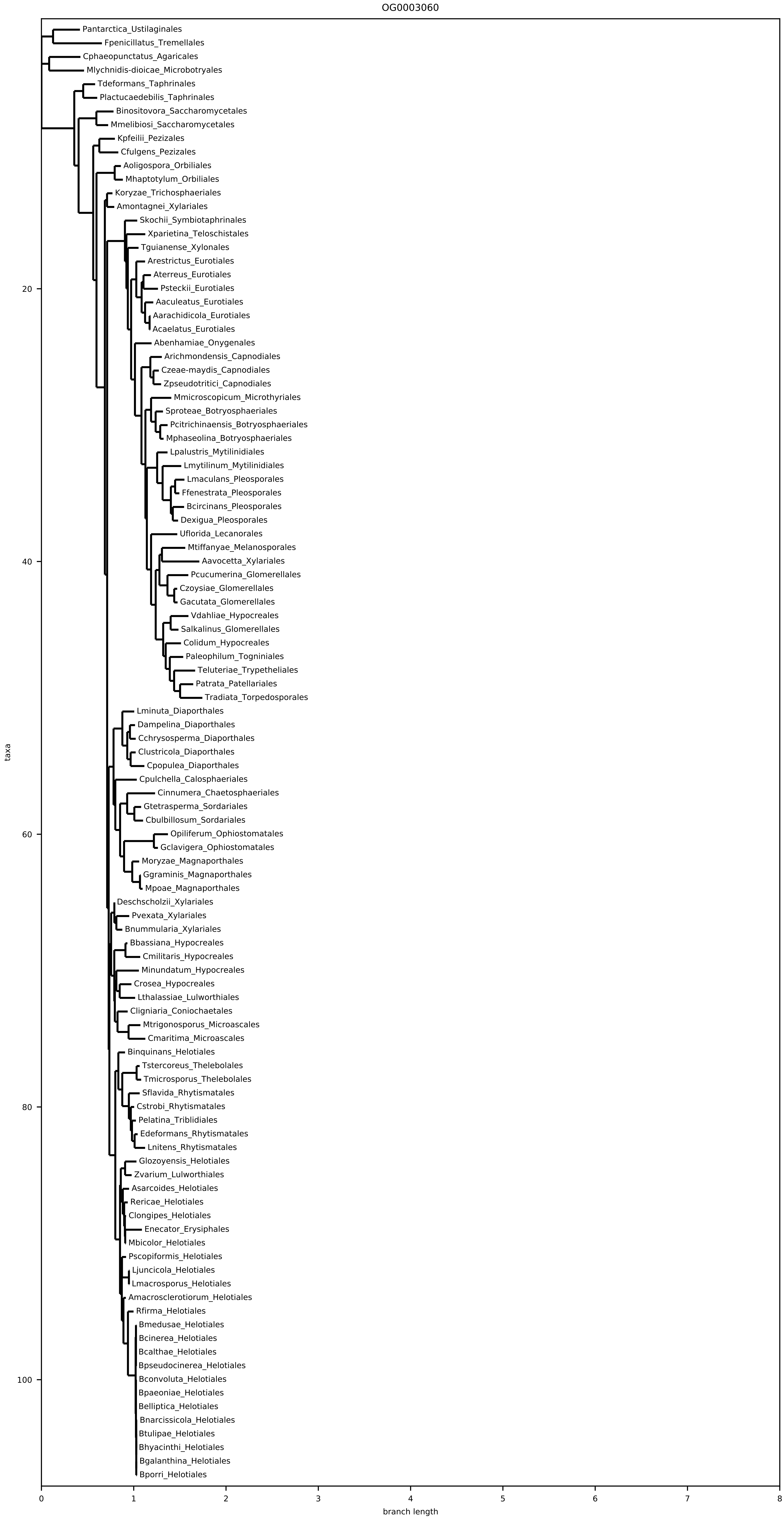
OG0003060
Pantarctica_Ustilaginales
Fpenicillatus_Tremellales
Cphaeopunctatus_Agaricales
Mlychnidis-dioicae_Microbotryales
Tdeformans_Taphrinales
Plactucaedebilis_Taphrinales
Binositovora_Saccharomycetales
Mmelibiosi_Saccharomycetales
Kpfeilii_Pezizales
Cfulgens_Pezizales
Aoligospora_Orbiliales
Mhaptotylum_Orbiliales
Koryzae_Trichosphaeriales
Amontagnei_Xylariales
Skochii_Symbiotaphrinales
Xparietina_Teloschistales
Tguianense_Xylonales
Arestrictus_Eurotiales
Aterreus_Eurotiales
Psteckii_Eurotiales
Aaculeatus_Eurotiales
Aarachidicola_Eurotiales
Acaelatus_Eurotiales
Abenhamiae_Onygenales
Arichmondensis_Capnodiales
Czeae-maydis_Capnodiales
Zpseudotritici_Capnodiales
Mmicroscopicum_Microthyriales
Sproteae_Botryosphaeriales
Pcitrichinaensis_Botryosphaeriales
Mphaseolina_Botryosphaeriales
Lpalustris_Mytilinidiales
Lmytilinum_Mytilinidiales
Lmaculans_Pleosporales
Ffenestrata_Pleosporales
Bcircinans_Pleosporales
Dexigua_Pleosporales
Uflorida_Lecanorales
Mtiffanyae_Melanosporales
Aavocetta_Xylariales
Pcucumerina_Glomerellales
Czoysiae_Glomerellales
Gacutata_Glomerellales
Vdahliae_Hypocreales
Salkalinus_Glomerellales
Colidum_Hypocreales
Paleophilum_Togniniales
Teluteriae_Trypetheliales
Patrata_Patellariales
Tradiata_Torpedosporales
Lminuta_Diaporthales
Dampelina_Diaporthales
Cchrysosperma_Diaporthales
Clustricola_Diaporthales
Cpopulea_Diaporthales
Cpulchella_Calosphaeriales
Cinnumera_Chaetosphaeriales
Gtetrasperma_Sordariales
Cbulbillosum_Sordariales
Opiliferum_Ophiostomatales
Gclavigera_Ophiostomatales
Moryzae_Magnaporthales
Ggraminis_Magnaporthales
Mpoae_Magnaporthales
Deschscholzii_Xylariales
Pvexata_Xylariales
Bnummularia_Xylariales
Bbassiana_Hypocreales
Cmilitaris_Hypocreales
Minundatum_Hypocreales
Crosea_Hypocreales
Lthalassiae_Lulworthiales
Cligniaria_Coniochaetales
Mtrigonosporus_Microascales
Cmaritima_Microascales
Binquinans_Helotiales
Tstercoreus_Thelebolales
Tmicrosporus_Thelebolales
Sflavida_Rhytismatales
Cstrobi_Rhytismatales
Pelatina_Triblidiales
Edeformans_Rhytismatales
Lnitens_Rhytismatales
Glozoyensis_Helotiales
Zvarium_Lulworthiales
Asarcoides_Helotiales
Rericae_Helotiales
Clongipes_Helotiales
Enecator_Erysiphales
Mbicolor_Helotiales
Pscopiformis_Helotiales
Ljuncicola_Helotiales
Lmacrosporus_Helotiales
Amacrosclerotiorum_Helotiales
Rfirma_Helotiales
Bmedusae_Helotiales
Bcinerea_Helotiales
Bcalthae_Helotiales
Bpseudocinerea_Helotiales
Bconvoluta_Helotiales
Bpaeoniae_Helotiales
Belliptica_Helotiales
Bnarcissicola_Helotiales
Btulipae_Helotiales
Bhyacinthi_Helotiales
Bgalanthina_Helotiales
Bporri_Helotiales
taxa
20
40
60
80
100
0
1
2
3
4
5
6
7
8
branch length

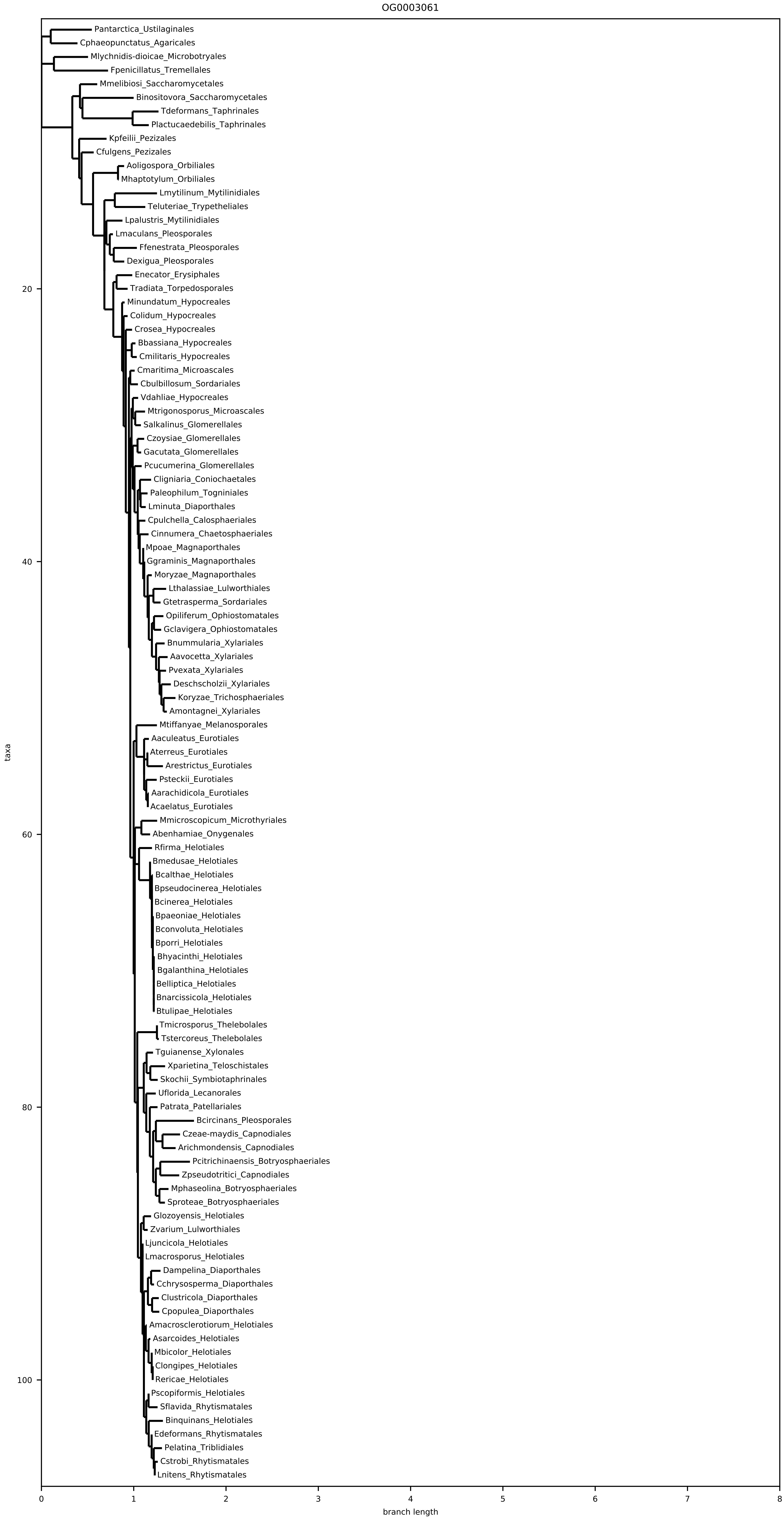

OG0003061
taxa
branch length
0
1
2
3
4
5
6
7
8
20
40
60
80
100
Pantarctica_Ustilaginales
Cphaeopunctatus_Agaricales
Mlychnidis-dioicae_Microbotryales
Fpenicillatus_Tremellales
Mmelibiosi_Saccharomycetales
Binositovora_Saccharomycetales
Tdeformans_Taphrinales
Plactucaedebilis_Taphrinales
Kpfeilii_Pezizales
Cfulgens_Pezizales
Aoligospora_Orbiliales
Mhaptotylum_Orbiliales
Lmytilinum_Mytilinidiales
Teluteriae_Trypetheliales
Lpalustris_Mytilinidiales
Lmaculans_Pleosporales
Ffenestrata_Pleosporales
Dexigua_Pleosporales
Enecator_Erysiphales
Tradiata_Torpedosporales
Minundatum_Hypocreales
Colidum_Hypocreales
Crosea_Hypocreales
Bbassiana_Hypocreales
Cmilitaris_Hypocreales
Cmaritima_Microascales
Cbulbillosum_Sordariales
Vdahliae_Hypocreales
Mtrigonosporus_Microascales
Salkalinus_Glomerellales
Czoysiae_Glomerellales
Gacutata_Glomerellales
Pcucumerina_Glomerellales
Cligniaria_Coniochaetales
Paleophilum_Togniniales
Lminuta_Diaporthales
Cpulchella_Calosphaeriales
Cinnumera_Chaetosphaeriales
Mpoae_Magnaporthales
Ggraminis_Magnaporthales
Moryzae_Magnaporthales
Lthalassiae_Lulworthiales
Gtetrasperma_Sordariales
Opiliferum_Ophiostomatales
Gclavigera_Ophiostomatales
Bnummularia_Xylariales
Aavocetta_Xylariales
Pvexata_Xylariales
Deschscholzii_Xylariales
Koryzae_Trichosphaeriales
Amontagnei_Xylariales
Mtiffanyae_Melanosporales
Aaculeatus_Eurotiales
Aterreus_Eurotiales
Arestrictus_Eurotiales
Psteckii_Eurotiales
Aarachidicola_Eurotiales
Acaelatus_Eurotiales
Mmicroscopicum_Microthyriales
Abenhamiae_Onygenales
Rfirma_Helotiales
Bmedusae_Helotiales
Bcalthae_Helotiales
Bpseudocinerea_Helotiales
Bcinerea_Helotiales
Bpaeoniae_Helotiales
Bconvoluta_Helotiales
Bporri_Helotiales
Bhyacinthi_Helotiales
Bgalanthina_Helotiales
Belliptica_Helotiales
Bnarcissicola_Helotiales
Btulipae_Helotiales
Tmicrosporus_Thelebolales
Tstercoreus_Thelebolales
Tguianense_Xylonales
Xparietina_Teloschistales
Skochii_Symbiotaphrinales
Uflorida_Lecanorales
Patrata_Patellariales
Bcircinans_Pleosporales
Czeae-maydis_Capnodiales
Arichmondensis_Capnodiales
Pcitrichinaensis_Botryosphaeriales
Zpseudotritici_Capnodiales
Mphaseolina_Botryosphaeriales
Sproteae_Botryosphaeriales
Glozoyensis_Helotiales
Zvarium_Lulworthiales
Ljuncicola_Helotiales
Lmacrosporus_Helotiales
Dampelina_Diaporthales
Cchrysosperma_Diaporthales
Clustricola_Diaporthales
Cpopulea_Diaporthales
Amacrosclerotiorum_Helotiales
Asarcoides_Helotiales
Mbicolor_Helotiales
Clongipes_Helotiales
Rericae_Helotiales
Pscopiformis_Helotiales
Sflavida_Rhytismatales
Binquinans_Helotiales
Edeformans_Rhytismatales
Pelatina_Triblidiales
Cstrobi_Rhytismatales
Lnitens_Rhytismatales

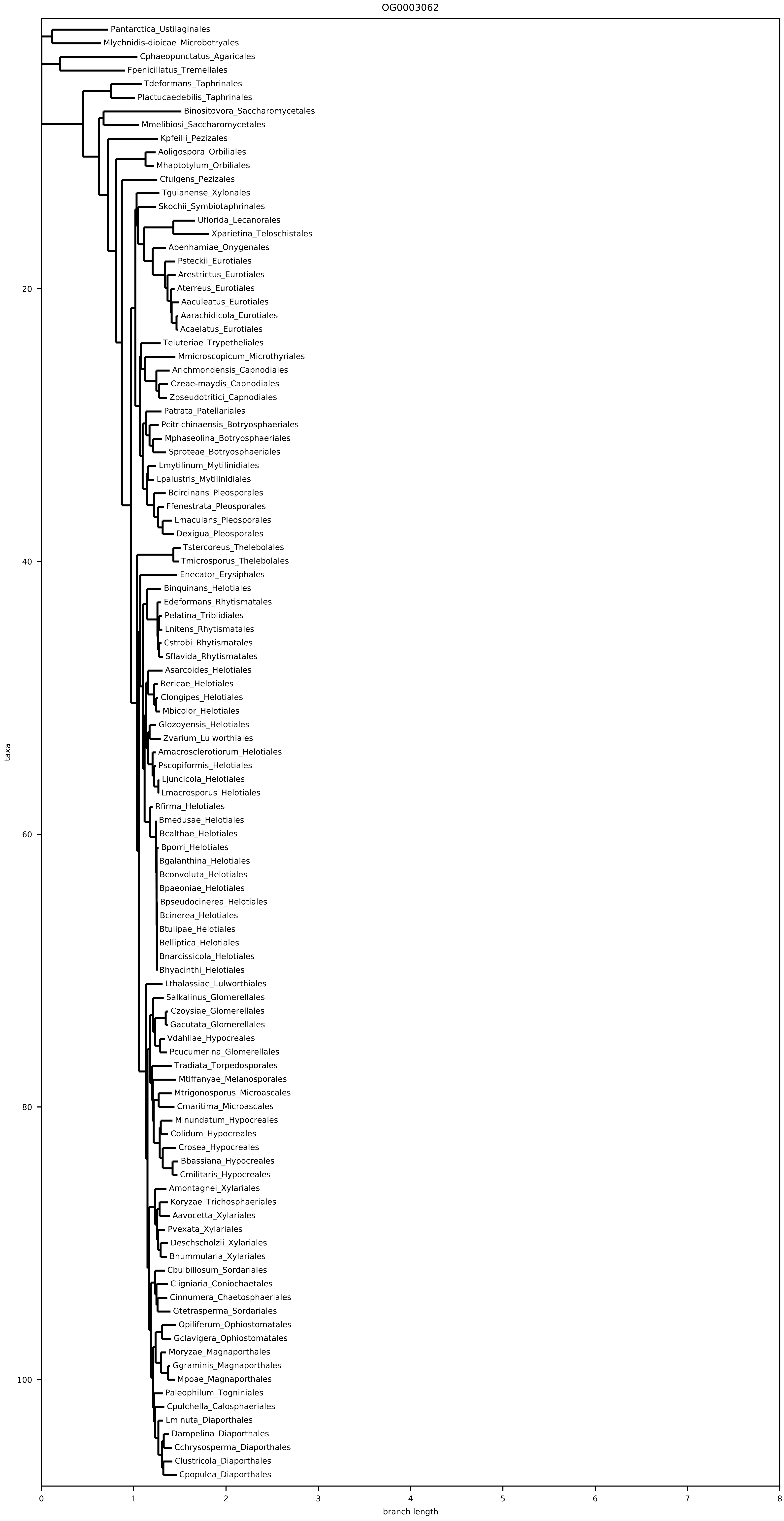

OG0003062
Pantarctica_Ustilaginales
Mlychnidis-dioicae_Microbotryales
Cphaeopunctatus_Agaricales
Fpenicillatus_Tremellales
Tdeformans_Taphrinales
Plactucaedebilis_Taphrinales
Binositovora_Saccharomycetales
Mmelibiosi_Saccharomycetales
Kpfeilii_Pezizales
Aoligospora_Orbiliales
Mhaptotylum_Orbiliales
Cfulgens_Pezizales
Tguianense_Xylonales
Skochii_Symbiotaphrinales
Uflorida_Lecanorales
Xparietina_Teloschistales
Abenhamiae_Onygenales
Psteckii_Eurotiales
Arestrictus_Eurotiales
Aterreus_Eurotiales
Aaculeatus_Eurotiales
Aarachidicola_Eurotiales
Acaelatus_Eurotiales
Teluteriae_Trypetheliales
Mmicroscopicum_Microthyriales
Arichmondensis_Capnodiales
Czeae-maydis_Capnodiales
Zpseudotritici_Capnodiales
Patrata_Patellariales
Pcitrichinaensis_Botryosphaeriales
Mphaseolina_Botryosphaeriales
Sproteae_Botryosphaeriales
Lmytilinum_Mytilinidiales
Lpalustris_Mytilinidiales
Bcircinans_Pleosporales
Ffenestrata_Pleosporales
Lmaculans_Pleosporales
Dexigua_Pleosporales
Tstercoreus_Thelebolales
Tmicrosporus_Thelebolales
Enecator_Erysiphales
Binquinans_Helotiales
Edeformans_Rhytismatales
Pelatina_Triblidiales
Lnitens_Rhytismatales
Cstrobi_Rhytismatales
Sflavida_Rhytismatales
Asarcoides_Helotiales
Rericae_Helotiales
Clongipes_Helotiales
Mbicolor_Helotiales
Glozoyensis_Helotiales
Zvarium_Lulworthiales
Amacrosclerotiorum_Helotiales
Pscopiformis_Helotiales
Ljuncicola_Helotiales
Lmacrosporus_Helotiales
Rfirma_Helotiales
Bmedusae_Helotiales
Bcalthae_Helotiales
Bporri_Helotiales
Bgalanthina_Helotiales
Bconvoluta_Helotiales
Bpaeoniae_Helotiales
Bpseudocinerea_Helotiales
Bcinerea_Helotiales
Btulipae_Helotiales
Belliptica_Helotiales
Bnarcissicola_Helotiales
Bhyacinthi_Helotiales
Lthalassiae_Lulworthiales
Salkalinus_Glomerellales
Czoysiae_Glomerellales
Gacutata_Glomerellales
Vdahliae_Hypocreales
Pcucumerina_Glomerellales
Tradiata_Torpedosporales
Mtiffanyae_Melanosporales
Mtrigonosporus_Microascales
Cmaritima_Microascales
Minundatum_Hypocreales
Colidum_Hypocreales
Crosea_Hypocreales
Bbassiana_Hypocreales
Cmilitaris_Hypocreales
Amontagnei_Xylariales
Koryzae_Trichosphaeriales
Aavocetta_Xylariales
Pvexata_Xylariales
Deschscholzii_Xylariales
Bnummularia_Xylariales
Cbulbillosum_Sordariales
Cligniaria_Coniochaetales
Cinnumera_Chaetosphaeriales
Gtetrasperma_Sordariales
Opiliferum_Ophiostomatales
Gclavigera_Ophiostomatales
Moryzae_Magnaporthales
Ggraminis_Magnaporthales
Mpoae_Magnaporthales
Paleophilum_Togniniales
Cpulchella_Calosphaeriales
Lminuta_Diaporthales
Dampelina_Diaporthales
Cchrysosperma_Diaporthales
Clustricola_Diaporthales
Cpopulea_Diaporthales
taxa
20
40
60
80
100
branch length
0
1
2
3
4
5
6
7
8

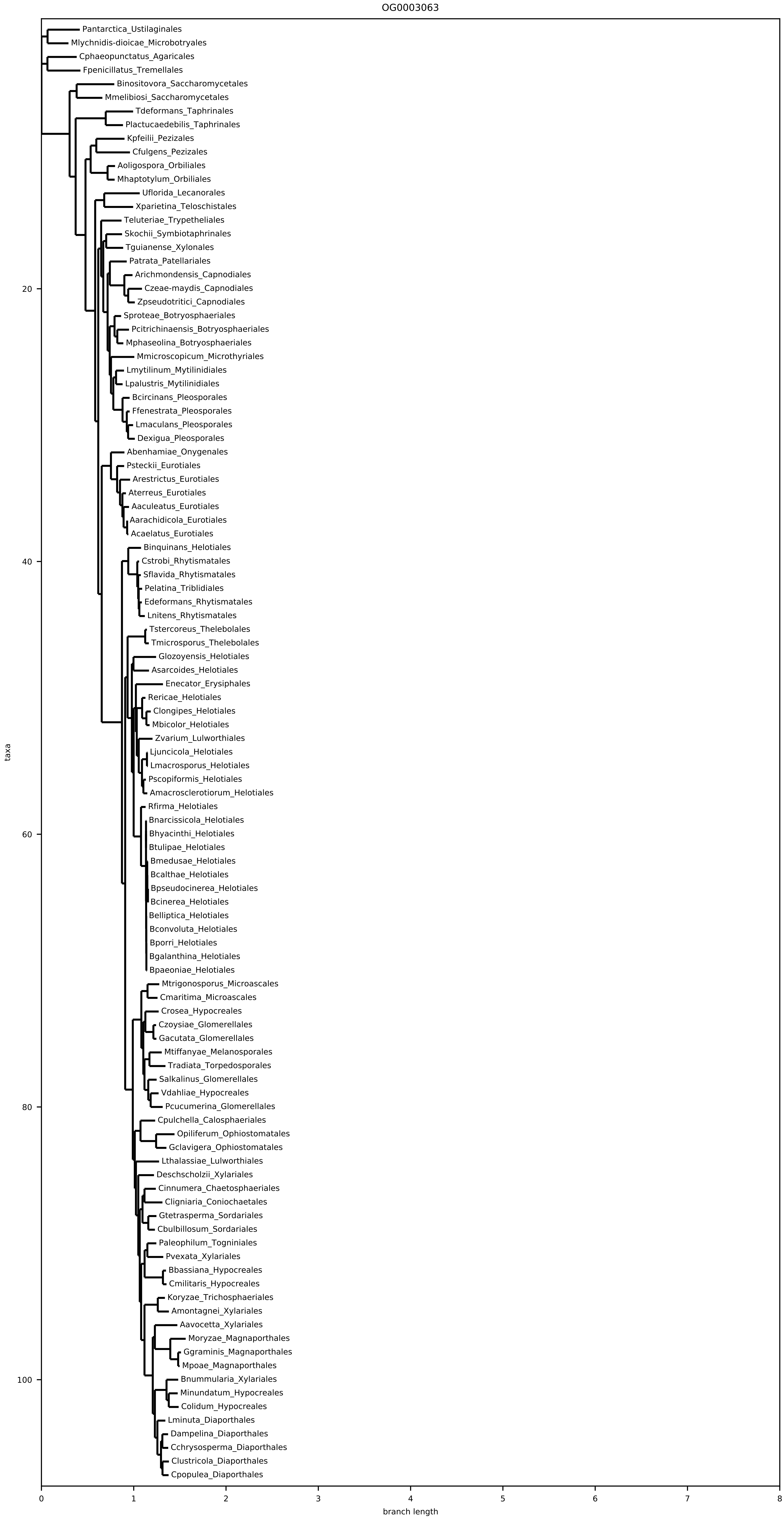

OG0003063
taxa
branch length
Pantarctica_Ustilaginales
Mlychnidis-dioicae_Microbotryales
Cphaeopunctatus_Agaricales
Fpenicillatus_Tremellales
Binositovora_Saccharomycetales
Mmelibiosi_Saccharomycetales
Tdeformans_Taphrinales
Plactucaedebilis_Taphrinales
Kpfeilii_Pezizales
Cfulgens_Pezizales
Aoligospora_Orbiliales
Mhaptotylum_Orbiliales
Uflorida_Lecanorales
Xparietina_Teloschistales
Teluteriae_Trypetheliales
Skochii_Symbiotaphrinales
Tguianense_Xylonales
Patrata_Patellariales
Arichmondensis_Capnodiales
Czeae-maydis_Capnodiales
Zpseudotritici_Capnodiales
Sproteae_Botryosphaeriales
Pcitrichinaensis_Botryosphaeriales
Mphaseolina_Botryosphaeriales
Mmicroscopicum_Microthyriales
Lmytilinum_Mytilinidiales
Lpalustris_Mytilinidiales
Bcircinans_Pleosporales
Ffenestrata_Pleosporales
Lmaculans_Pleosporales
Dexigua_Pleosporales
Abenhamiae_Onygenales
Psteckii_Eurotiales
Arestrictus_Eurotiales
Aterreus_Eurotiales
Aaculeatus_Eurotiales
Aarachidicola_Eurotiales
Acaelatus_Eurotiales
Binquinans_Helotiales
Cstrobi_Rhytismatales
Sflavida_Rhytismatales
Pelatina_Triblidiales
Edeformans_Rhytismatales
Lnitens_Rhytismatales
Tstercoreus_Thelebolales
Tmicrosporus_Thelebolales
Glozoyensis_Helotiales
Asarcoides_Helotiales
Enecator_Erysiphales
Rericae_Helotiales
Clongipes_Helotiales
Mbicolor_Helotiales
Zvarium_Lulworthiales
Ljuncicola_Helotiales
Lmacrosporus_Helotiales
Pscopiformis_Helotiales
Amacrosclerotiorum_Helotiales
Rfirma_Helotiales
Bnarcissicola_Helotiales
Bhyacinthi_Helotiales
Btulipae_Helotiales
Bmedusae_Helotiales
Bcalthae_Helotiales
Bpseudocinerea_Helotiales
Bcinerea_Helotiales
Belliptica_Helotiales
Bconvoluta_Helotiales
Bporri_Helotiales
Bgalanthina_Helotiales
Bpaeoniae_Helotiales
Mtrigonosporus_Microascales
Cmaritima_Microascales
Crosea_Hypocreales
Czoysiae_Glomerellales
Gacutata_Glomerellales
Mtiffanyae_Melanosporales
Tradiata_Torpedosporales
Salkalinus_Glomerellales
Vdahliae_Hypocreales
Pcucumerina_Glomerellales
Cpulchella_Calosphaeriales
Opiliferum_Ophiostomatales
Gclavigera_Ophiostomatales
Lthalassiae_Lulworthiales
Deschscholzii_Xylariales
Cinnumera_Chaetosphaeriales
Cligniaria_Coniochaetales
Gtetrasperma_Sordariales
Cbulbillosum_Sordariales
Paleophilum_Togniniales
Pvexata_Xylariales
Bbassiana_Hypocreales
Cmilitaris_Hypocreales
Koryzae_Trichosphaeriales
Amontagnei_Xylariales
Aavocetta_Xylariales
Moryzae_Magnaporthales
Ggraminis_Magnaporthales
Mpoae_Magnaporthales
Bnummularia_Xylariales
Minundatum_Hypocreales
Colidum_Hypocreales
Lminuta_Diaporthales
Dampelina_Diaporthales
Cchrysosperma_Diaporthales
Clustricola_Diaporthales
Cpopulea_Diaporthales
20
40
60
80
100
0
1
2
3
4
5
6
7
8

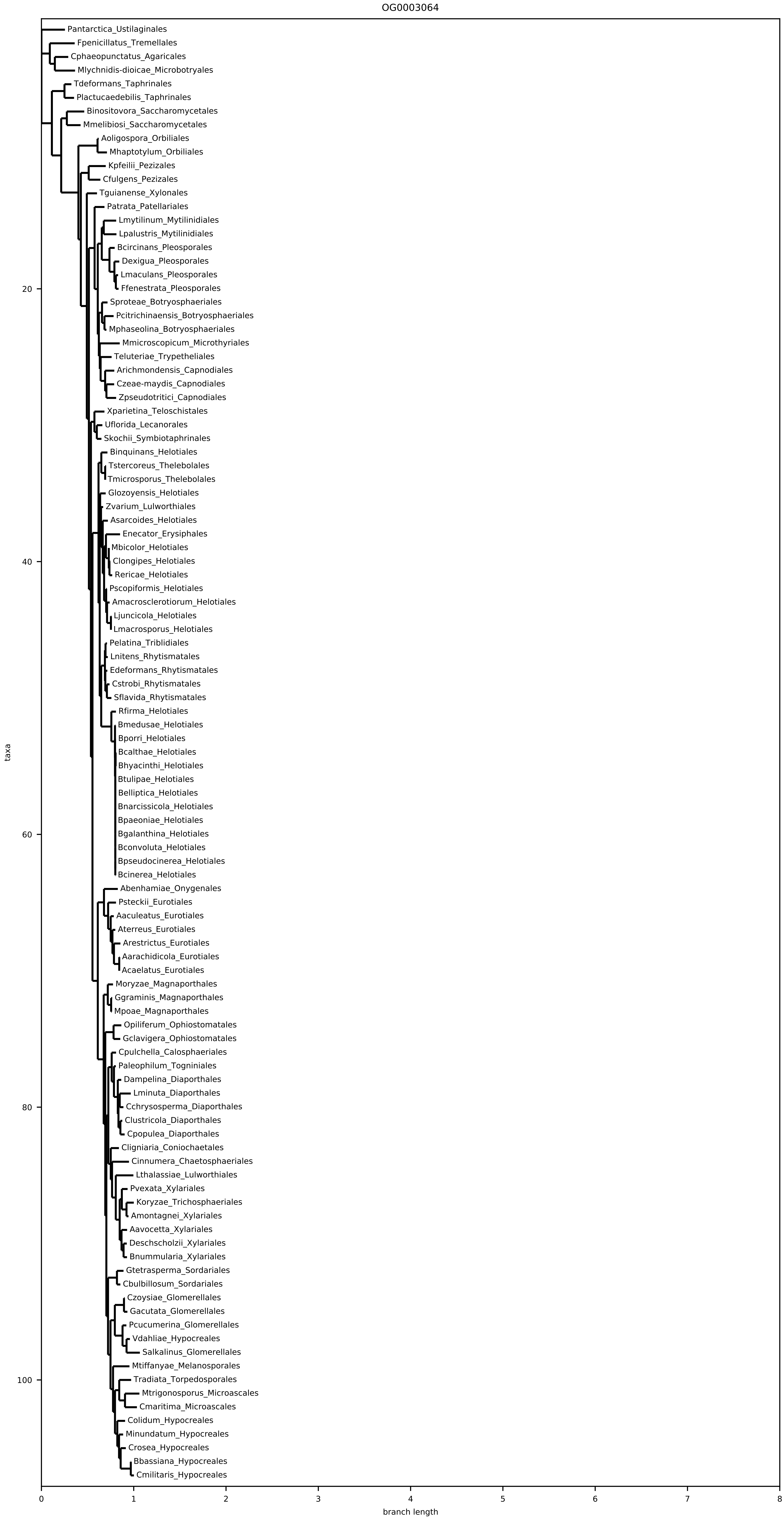
OG0003064
Pantarctica_Ustilaginales
Fpenicillatus_Tremellales
Cphaeopunctatus_Agaricales
Mlychnidis-dioicae_Microbotryales
Tdeformans_Taphrinales
Plactucaedebilis_Taphrinales
Binositovora_Saccharomycetales
Mmelibiosi_Saccharomycetales
Aoligospora_Orbiliales
Mhaptotylum_Orbiliales
Kpfeilii_Pezizales
Cfulgens_Pezizales
Tguianense_Xylonales
Patrata_Patellariales
Lmytilinum_Mytilinidiales
Lpalustris_Mytilinidiales
Bcircinans_Pleosporales
Dexigua_Pleosporales
Lmaculans_Pleosporales
Ffenestrata_Pleosporales
Sproteae_Botryosphaeriales
Pcitrichinaensis_Botryosphaeriales
Mphaseolina_Botryosphaeriales
Mmicroscopicum_Microthyriales
Teluteriae_Trypetheliales
Arichmondensis_Capnodiales
Czeae-maydis_Capnodiales
Zpseudotritici_Capnodiales
Xparietina_Teloschistales
Uflorida_Lecanorales
Skochii_Symbiotaphrinales
Binquinans_Helotiales
Tstercoreus_Thelebolales
Tmicrosporus_Thelebolales
Glozoyensis_Helotiales
Zvarium_Lulworthiales
Asarcoides_Helotiales
Enecator_Erysiphales
Mbicolor_Helotiales
Clongipes_Helotiales
Rericae_Helotiales
Pscopiformis_Helotiales
Amacrosclerotiorum_Helotiales
Ljuncicola_Helotiales
Lmacrosporus_Helotiales
Pelatina_Triblidiales
Lnitens_Rhytismatales
Edeformans_Rhytismatales
Cstrobi_Rhytismatales
Sflavida_Rhytismatales
Rfirma_Helotiales
Bmedusae_Helotiales
Bporri_Helotiales
Bcalthae_Helotiales
Bhyacinthi_Helotiales
Btulipae_Helotiales
Belliptica_Helotiales
Bnarcissicola_Helotiales
Bpaeoniae_Helotiales
Bgalanthina_Helotiales
Bconvoluta_Helotiales
Bpseudocinerea_Helotiales
Bcinerea_Helotiales
Abenhamiae_Onygenales
Psteckii_Eurotiales
Aaculeatus_Eurotiales
Aterreus_Eurotiales
Arestrictus_Eurotiales
Aarachidicola_Eurotiales
Acaelatus_Eurotiales
Moryzae_Magnaporthales
Ggraminis_Magnaporthales
Mpoae_Magnaporthales
Opiliferum_Ophiostomatales
Gclavigera_Ophiostomatales
Cpulchella_Calosphaeriales
Paleophilum_Togniniales
Dampelina_Diaporthales
Lminuta_Diaporthales
Cchrysosperma_Diaporthales
Clustricola_Diaporthales
Cpopulea_Diaporthales
Cligniaria_Coniochaetales
Cinnumera_Chaetosphaeriales
Lthalassiae_Lulworthiales
Pvexata_Xylariales
Koryzae_Trichosphaeriales
Amontagnei_Xylariales
Aavocetta_Xylariales
Deschscholzii_Xylariales
Bnummularia_Xylariales
Gtetrasperma_Sordariales
Cbulbillosum_Sordariales
Czoysiae_Glomerellales
Gacutata_Glomerellales
Pcucumerina_Glomerellales
Vdahliae_Hypocreales
Salkalinus_Glomerellales
Mtiffanyae_Melanosporales
Tradiata_Torpedosporales
Mtrigonosporus_Microascales
Cmaritima_Microascales
Colidum_Hypocreales
Minundatum_Hypocreales
Crosea_Hypocreales
Bbassiana_Hypocreales
Cmilitaris_Hypocreales
taxa
20
40
60
80
100
0
1
2
3
4
5
6
7
8
branch length

OG0003066

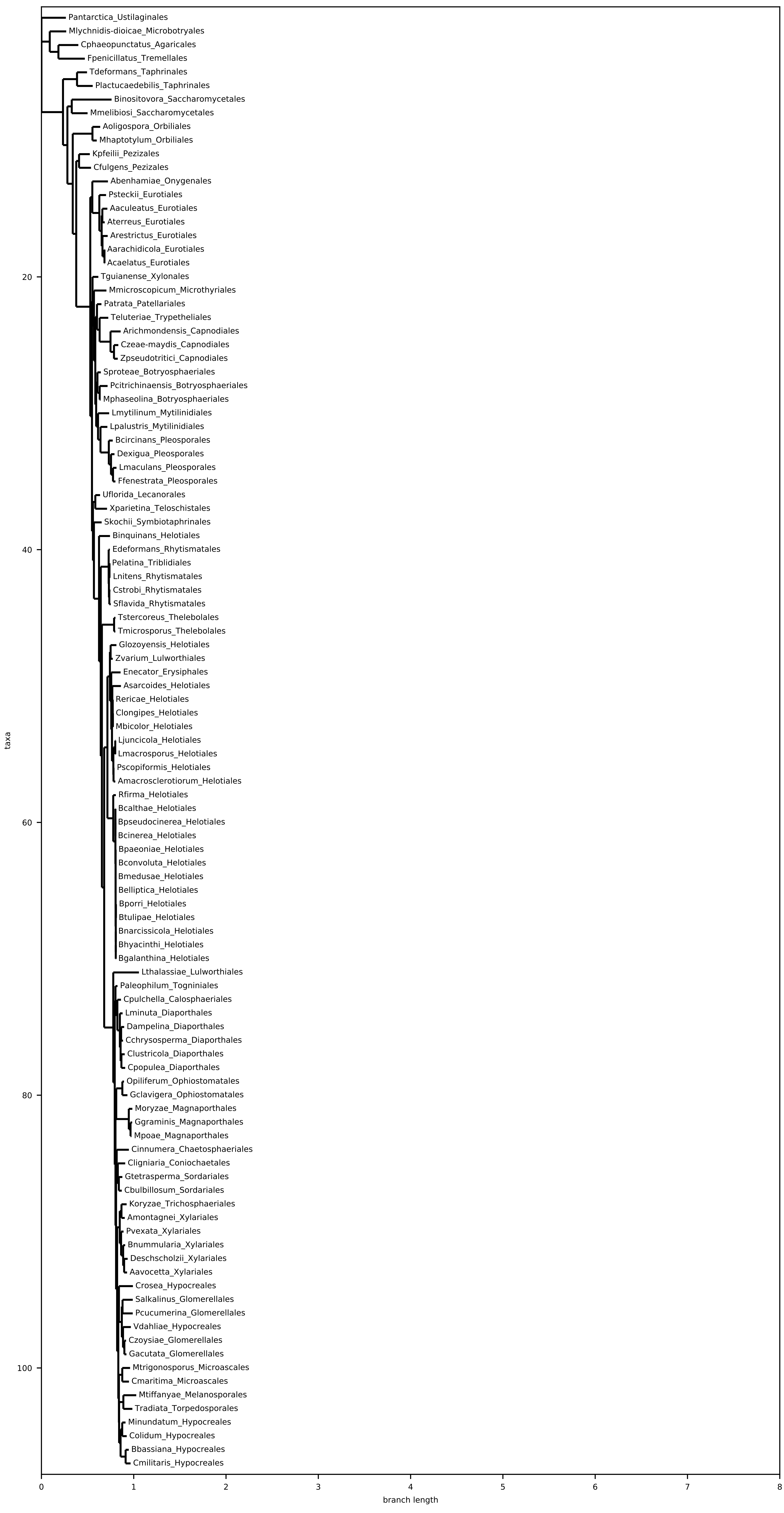

OG0003066
Pantarctica_Ustilaginales
Mlychnidis-dioicae_Microbotryales
Cphaeopunctatus_Agaricales
Fpenicillatus_Tremellales
Tdeformans_Taphrinales
Plactucaedebilis_Taphrinales
Binositovora_Saccharomycetales
Mmelibiosi_Saccharomycetales
Aoligospora_Orbiliales
Mhaptotylum_Orbiliales
Kpfeilii_Pezizales
Cfulgens_Pezizales
Abenhamiae_Onygenales
Psteckii_Eurotiales
Aaculeatus_Eurotiales
Aterreus_Eurotiales
Arestrictus_Eurotiales
Aarachidicola_Eurotiales
Acaelatus_Eurotiales
Tguianense_Xylonales
Mmicroscopicum_Microthyriales
Patrata_Patellariales
Teluteriae_Trypetheliales
Arichmondensis_Capnodiales
Czeae-maydis_Capnodiales
Zpseudotritici_Capnodiales
Sproteae_Botryosphaeriales
Pcitrichinaensis_Botryosphaeriales
Mphaseolina_Botryosphaeriales
Lmytilinum_Mytilinidiales
Lpalustris_Mytilinidiales
Bcircinans_Pleosporales
Dexigua_Pleosporales
Lmaculans_Pleosporales
Ffenestrata_Pleosporales
Uflorida_Lecanorales
Xparietina_Teloschistales
Skochii_Symbiotaphrinales
Binquinans_Helotiales
Edeformans_Rhytismatales
Pelatina_Triblidiales
Lnitens_Rhytismatales
Cstrobi_Rhytismatales
Sflavida_Rhytismatales
Tstercoreus_Thelebolales
Tmicrosporus_Thelebolales
Glozoyensis_Helotiales
Zvarium_Lulworthiales
Enecator_Erysiphales
Asarcoides_Helotiales
Rericae_Helotiales
Clongipes_Helotiales
Mbicolor_Helotiales
Ljuncicola_Helotiales
Lmacrosporus_Helotiales
Pscopiformis_Helotiales
Amacrosclerotiorum_Helotiales
Rfirma_Helotiales
Bcalthae_Helotiales
Bpseudocinerea_Helotiales
Bcinerea_Helotiales
Bpaeoniae_Helotiales
Bconvoluta_Helotiales
Bmedusae_Helotiales
Belliptica_Helotiales
Bporri_Helotiales
Btulipae_Helotiales
Bnarcissicola_Helotiales
Bhyacinthi_Helotiales
Bgalanthina_Helotiales
Lthalassiae_Lulworthiales
Paleophilum_Togniniales
Cpulchella_Calosphaeriales
Lminuta_Diaporthales
Dampelina_Diaporthales
Cchrysosperma_Diaporthales
Clustricola_Diaporthales
Cpopulea_Diaporthales
Opiliferum_Ophiostomatales
Gclavigera_Ophiostomatales
Moryzae_Magnaporthales
Ggraminis_Magnaporthales
Mpoae_Magnaporthales
Cinnumera_Chaetosphaeriales
Cligniaria_Coniochaetales
Gtetrasperma_Sordariales
Cbulbillosum_Sordariales
Koryzae_Trichosphaeriales
Amontagnei_Xylariales
Pvexata_Xylariales
Bnummularia_Xylariales
Deschscholzii_Xylariales
Aavocetta_Xylariales
Crosea_Hypocreales
Salkalinus_Glomerellales
Pcucumerina_Glomerellales
Vdahliae_Hypocreales
Czoysiae_Glomerellales
Gacutata_Glomerellales
Mtrigonosporus_Microascales
Cmaritima_Microascales
Mtiffanyae_Melanosporales
Tradiata_Torpedosporales
Minundatum_Hypocreales
Colidum_Hypocreales
Bbassiana_Hypocreales
Cmilitaris_Hypocreales
taxa
20
40
60
80
100
0
1
2
3
4
5
6
7
8
branch length

OG0003067

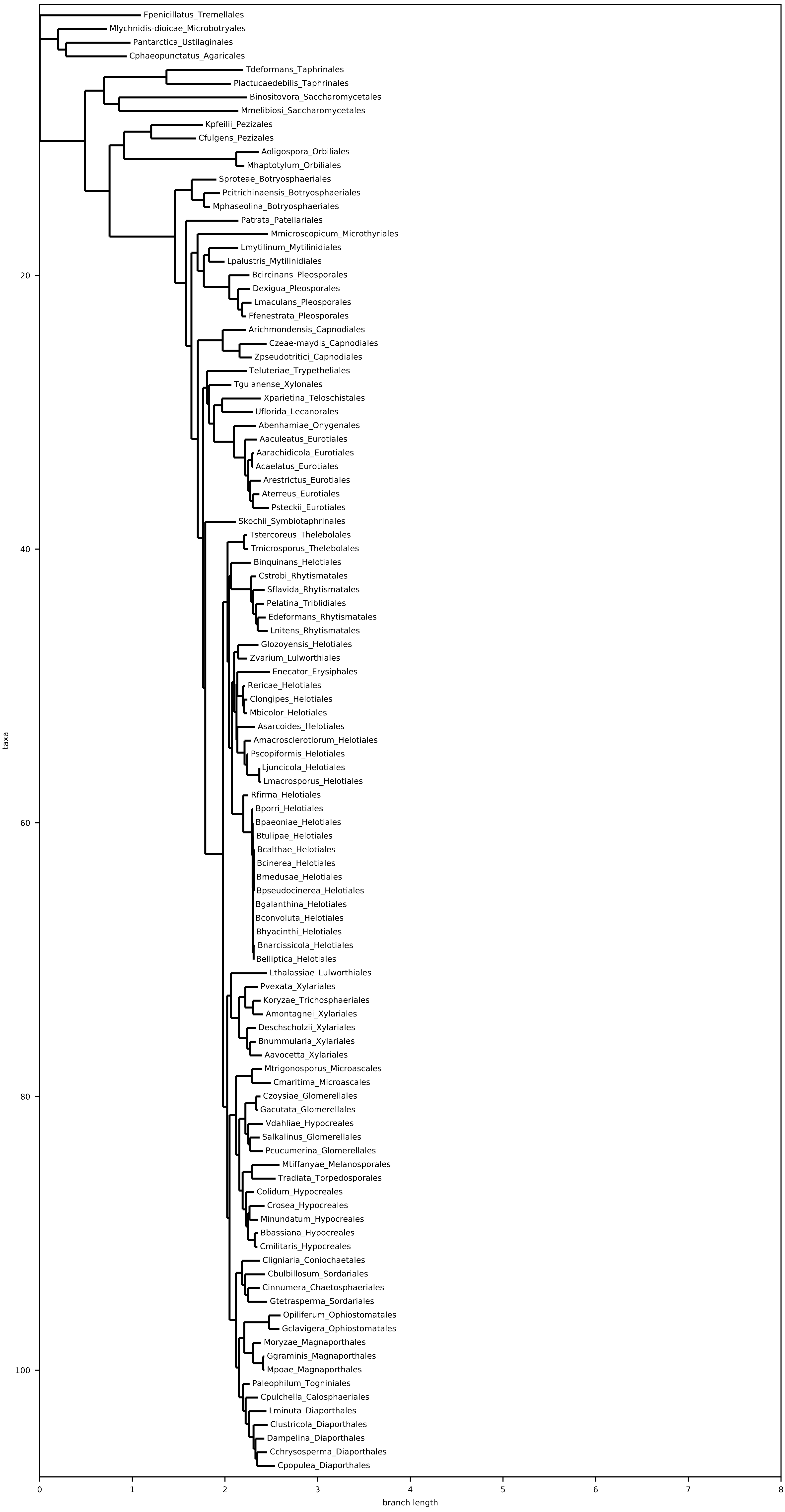

Fpenicillatus_Tremellales
Mlychnidis-dioicae_Microbotryales
Pantarctica_Ustilaginales
Cphaeopunctatus_Agaricales
Tdeformans_Taphrinales
Plactucaedebilis_Taphrinales
Binositovora_Saccharomycetales
Mmelibiosi_Saccharomycetales
Kpfeilii_Pezizales
Cfulgens_Pezizales
Aoligospora_Orbiliales
Mhaptotylum_Orbiliales
Sproteae_Botryosphaeriales
Pcitrichinaensis_Botryosphaeriales
Mphaseolina_Botryosphaeriales
Patrata_Patellariales
Mmicroscopicum_Microthyriales
Lmytilinum_Mytilinidiales
Lpalustris_Mytilinidiales
Bcircinans_Pleosporales
Dexigua_Pleosporales
Lmaculans_Pleosporales
Ffenestrata_Pleosporales
Arichmondensis_Capnodiales
Czeae-maydis_Capnodiales
Zpseudotritici_Capnodiales
Teluteriae_Trypetheliales
Tguianense_Xylonales
Xparietina_Teloschistales
Uflorida_Lecanorales
Abenhamiae_Onygenales
Aaculeatus_Eurotiales
Aarachidicola_Eurotiales
Acaelatus_Eurotiales
Arestrictus_Eurotiales
Aterreus_Eurotiales
Psteckii_Eurotiales
Skochii_Symbiotaphrinales
Tstercoreus_Thelebolales
Tmicrosporus_Thelebolales
Binquinans_Helotiales
Cstrobi_Rhytismatales
Sflavida_Rhytismatales
Pelatina_Triblidiales
Edeformans_Rhytismatales
Lnitens_Rhytismatales
Glozoyensis_Helotiales
Zvarium_Lulworthiales
Enecator_Erysiphales
Rericae_Helotiales
Clongipes_Helotiales
Mbicolor_Helotiales
Asarcoides_Helotiales
Amacrosclerotiorum_Helotiales
Pscopiformis_Helotiales
Ljuncicola_Helotiales
Lmacrosporus_Helotiales
Rfirma_Helotiales
Bporri_Helotiales
Bpaeoniae_Helotiales
Btulipae_Helotiales
Bcalthae_Helotiales
Bcinerea_Helotiales
Bmedusae_Helotiales
Bpseudocinerea_Helotiales
Bgalanthina_Helotiales
Bconvoluta_Helotiales
Bhyacinthi_Helotiales
Bnarcissicola_Helotiales
Belliptica_Helotiales
Lthalassiae_Lulworthiales
Pvexata_Xylariales
Koryzae_Trichosphaeriales
Amontagnei_Xylariales
Deschscholzii_Xylariales
Bnummularia_Xylariales
Aavocetta_Xylariales
Mtrigonosporus_Microascales
Cmaritima_Microascales
Czoysiae_Glomerellales
Gacutata_Glomerellales
Vdahliae_Hypocreales
Salkalinus_Glomerellales
Pcucumerina_Glomerellales
Mtiffanyae_Melanosporales
Tradiata_Torpedosporales
Colidum_Hypocreales
Crosea_Hypocreales
Minundatum_Hypocreales
Bbassiana_Hypocreales
Cmilitaris_Hypocreales
Cligniaria_Coniochaetales
Cbulbillosum_Sordariales
Cinnumera_Chaetosphaeriales
Gtetrasperma_Sordariales
Opiliferum_Ophiostomatales
Gclavigera_Ophiostomatales
Moryzae_Magnaporthales
Ggraminis_Magnaporthales
Mpoae_Magnaporthales
Paleophilum_Togniniales
Cpulchella_Calosphaeriales
Lminuta_Diaporthales
Clustricola_Diaporthales
Dampelina_Diaporthales
Cchrysosperma_Diaporthales
Cpopulea_Diaporthales
taxa
20
40
60
80
100
0
1
2
3
4
5
6
7
8
branch length

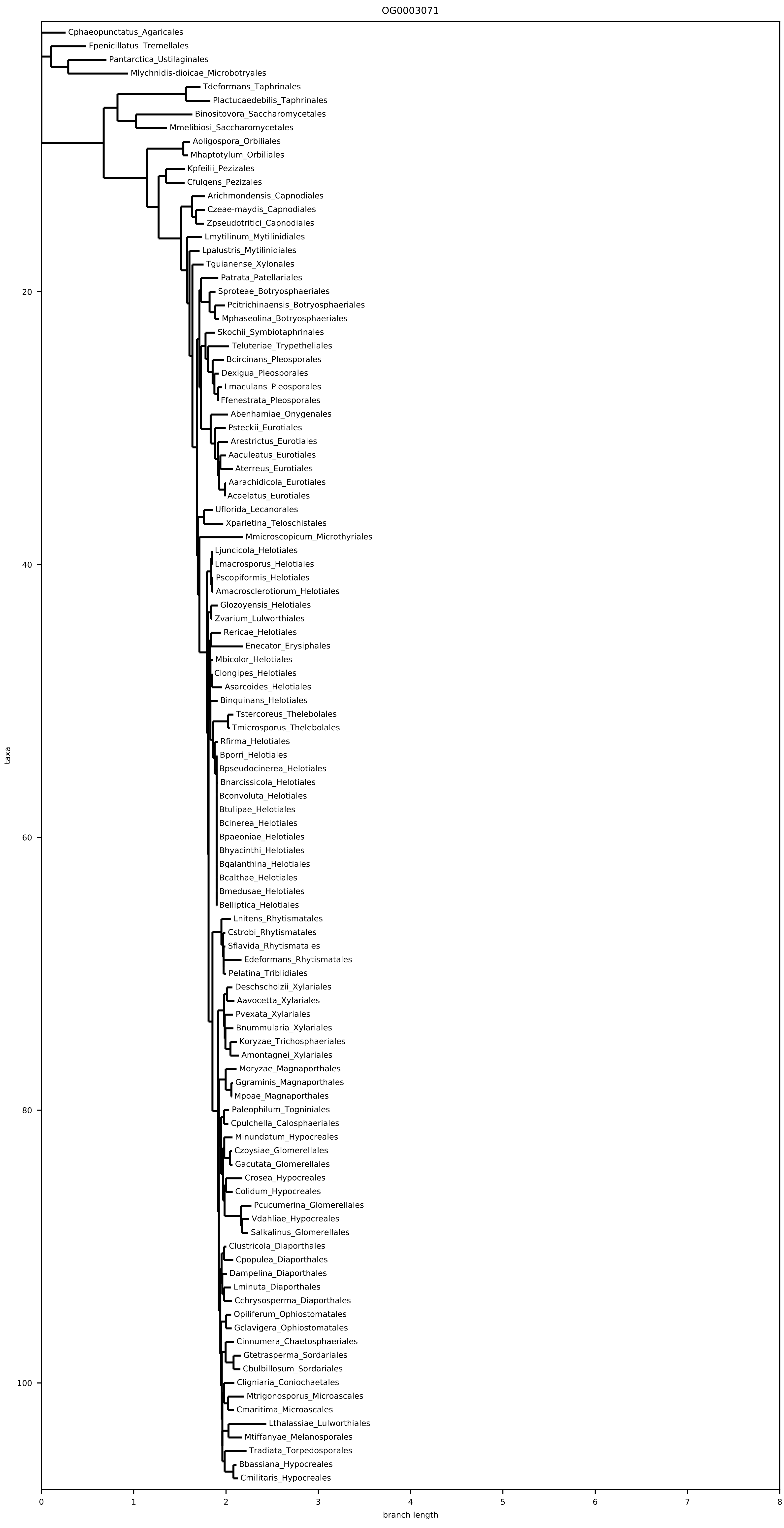
OG0003071
Cphaeopunctatus_Agaricales
Fpenicillatus_Tremellales
Pantarctica_Ustilaginales
Mlychnidis-dioicae_Microbotryales
Tdeformans_Taphrinales
Plactucaedebilis_Taphrinales
Binositovora_Saccharomycetales
Mmelibiosi_Saccharomycetales
Aoligospora_Orbiliales
Mhaptotylum_Orbiliales
Kpfeilii_Pezizales
Cfulgens_Pezizales
Arichmondensis_Capnodiales
Czeae-maydis_Capnodiales
Zpseudotritici_Capnodiales
Lmytilinum_Mytilinidiales
Lpalustris_Mytilinidiales
Tguianense_Xylonales
Patrata_Patellariales
Sproteae_Botryosphaeriales
Pcitrichinaensis_Botryosphaeriales
Mphaseolina_Botryosphaeriales
Skochii_Symbiotaphrinales
Teluteriae_Trypetheliales
Bcircinans_Pleosporales
Dexigua_Pleosporales
Lmaculans_Pleosporales
Ffenestrata_Pleosporales
Abenhamiae_Onygenales
Psteckii_Eurotiales
Arestrictus_Eurotiales
Aaculeatus_Eurotiales
Aterreus_Eurotiales
Aarachidicola_Eurotiales
Acaelatus_Eurotiales
Uflorida_Lecanorales
Xparietina_Teloschistales
Mmicroscopicum_Microthyriales
Ljuncicola_Helotiales
Lmacrosporus_Helotiales
Pscopiformis_Helotiales
Amacrosclerotiorum_Helotiales
Glozoyensis_Helotiales
Zvarium_Lulworthiales
Rericae_Helotiales
Enecator_Erysiphales
Mbicolor_Helotiales
Clongipes_Helotiales
Asarcoides_Helotiales
Binquinans_Helotiales
Tstercoreus_Thelebolales
Tmicrosporus_Thelebolales
Rfirma_Helotiales
Bporri_Helotiales
Bpseudocinerea_Helotiales
Bnarcissicola_Helotiales
Bconvoluta_Helotiales
Btulipae_Helotiales
Bcinerea_Helotiales
Bpaeoniae_Helotiales
Bhyacinthi_Helotiales
Bgalanthina_Helotiales
Bcalthae_Helotiales
Bmedusae_Helotiales
Belliptica_Helotiales
Lnitens_Rhytismatales
Cstrobi_Rhytismatales
Sflavida_Rhytismatales
Edeformans_Rhytismatales
Pelatina_Triblidiales
Deschscholzii_Xylariales
Aavocetta_Xylariales
Pvexata_Xylariales
Bnummularia_Xylariales
Koryzae_Trichosphaeriales
Amontagnei_Xylariales
Moryzae_Magnaporthales
Ggraminis_Magnaporthales
Mpoae_Magnaporthales
Paleophilum_Togniniales
Cpulchella_Calosphaeriales
Minundatum_Hypocreales
Czoysiae_Glomerellales
Gacutata_Glomerellales
Crosea_Hypocreales
Colidum_Hypocreales
Pcucumerina_Glomerellales
Vdahliae_Hypocreales
Salkalinus_Glomerellales
Clustricola_Diaporthales
Cpopulea_Diaporthales
Dampelina_Diaporthales
Lminuta_Diaporthales
Cchrysosperma_Diaporthales
Opiliferum_Ophiostomatales
Gclavigera_Ophiostomatales
Cinnumera_Chaetosphaeriales
Gtetrasperma_Sordariales
Cbulbillosum_Sordariales
Cligniaria_Coniochaetales
Mtrigonosporus_Microascales
Cmaritima_Microascales
Lthalassiae_Lulworthiales
Mtiffanyae_Melanosporales
Tradiata_Torpedosporales
Bbassiana_Hypocreales
Cmilitaris_Hypocreales
taxa
20
40
60
80
100
0
1
2
3
4
5
6
7
8
branch length

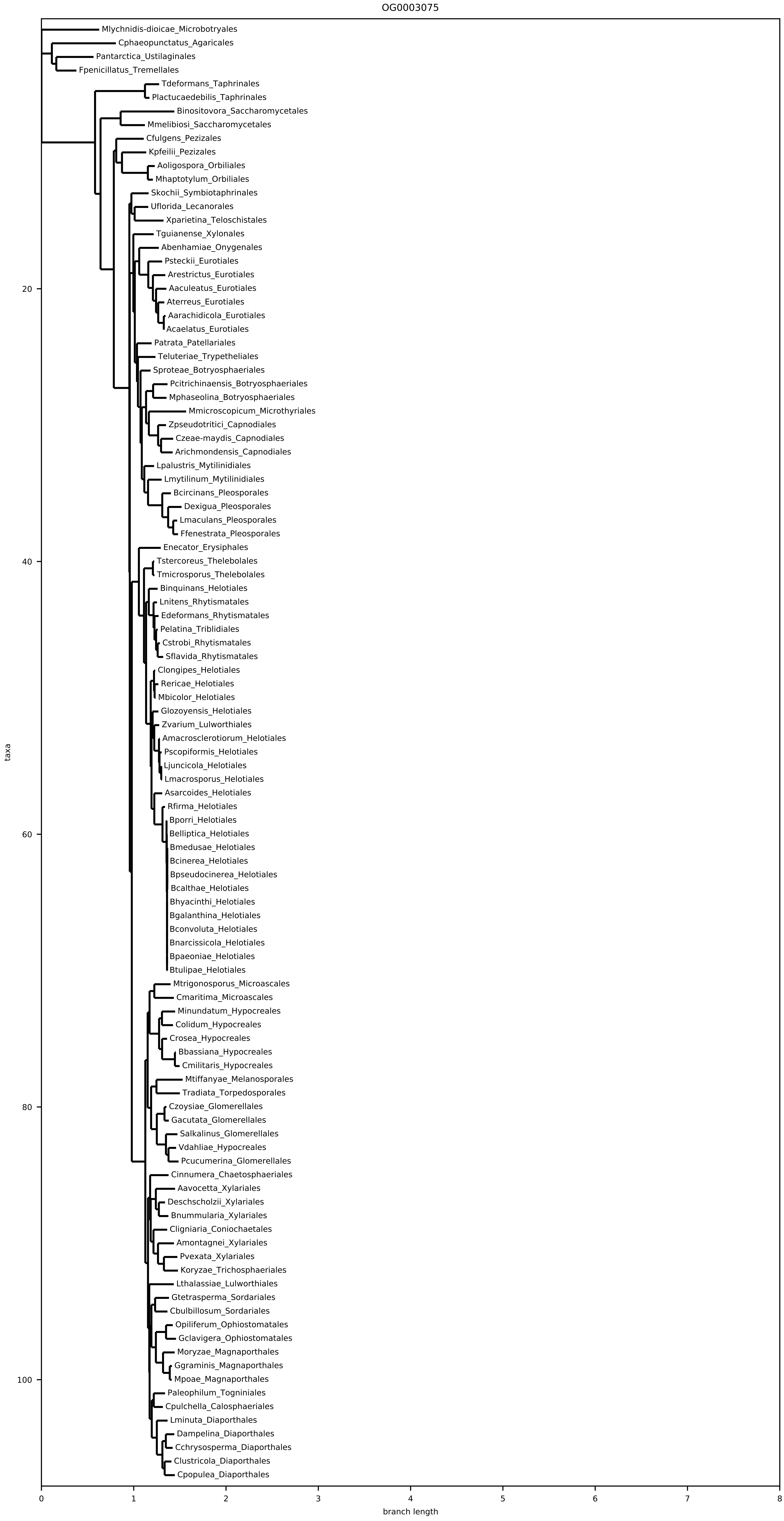
OG0003075
Mlychnidis-dioicae_Microbotryales
Cphaeopunctatus_Agaricales
Pantarctica_Ustilaginales
Fpenicillatus_Tremellales
Tdeformans_Taphrinales
Plactucaedebilis_Taphrinales
Binositovora_Saccharomycetales
Mmelibiosi_Saccharomycetales
Cfulgens_Pezizales
Kpfeilii_Pezizales
Aoligospora_Orbiliales
Mhaptotylum_Orbiliales
Skochii_Symbiotaphrinales
Uflorida_Lecanorales
Xparietina_Teloschistales
Tguianense_Xylonales
Abenhamiae_Onygenales
Psteckii_Eurotiales
Arestrictus_Eurotiales
Aaculeatus_Eurotiales
Aterreus_Eurotiales
Aarachidicola_Eurotiales
Acaelatus_Eurotiales
Patrata_Patellariales
Teluteriae_Trypetheliales
Sproteae_Botryosphaeriales
Pcitrichinaensis_Botryosphaeriales
Mphaseolina_Botryosphaeriales
Mmicroscopicum_Microthyriales
Zpseudotritici_Capnodiales
Czeae-maydis_Capnodiales
Arichmondensis_Capnodiales
Lpalustris_Mytilinidiales
Lmytilinum_Mytilinidiales
Bcircinans_Pleosporales
Dexigua_Pleosporales
Lmaculans_Pleosporales
Ffenestrata_Pleosporales
Enecator_Erysiphales
Tstercoreus_Thelebolales
Tmicrosporus_Thelebolales
Binquinans_Helotiales
Lnitens_Rhytismatales
Edeformans_Rhytismatales
Pelatina_Triblidiales
Cstrobi_Rhytismatales
Sflavida_Rhytismatales
Clongipes_Helotiales
Rericae_Helotiales
Mbicolor_Helotiales
Glozoyensis_Helotiales
Zvarium_Lulworthiales
Amacrosclerotiorum_Helotiales
Pscopiformis_Helotiales
Ljuncicola_Helotiales
Lmacrosporus_Helotiales
Asarcoides_Helotiales
Rfirma_Helotiales
Bporri_Helotiales
Belliptica_Helotiales
Bmedusae_Helotiales
Bcinerea_Helotiales
Bpseudocinerea_Helotiales
Bcalthae_Helotiales
Bhyacinthi_Helotiales
Bgalanthina_Helotiales
Bconvoluta_Helotiales
Bnarcissicola_Helotiales
Bpaeoniae_Helotiales
Btulipae_Helotiales
Mtrigonosporus_Microascales
Cmaritima_Microascales
Minundatum_Hypocreales
Colidum_Hypocreales
Crosea_Hypocreales
Bbassiana_Hypocreales
Cmilitaris_Hypocreales
Mtiffanyae_Melanosporales
Tradiata_Torpedosporales
Czoysiae_Glomerellales
Gacutata_Glomerellales
Salkalinus_Glomerellales
Vdahliae_Hypocreales
Pcucumerina_Glomerellales
Cinnumera_Chaetosphaeriales
Aavocetta_Xylariales
Deschscholzii_Xylariales
Bnummularia_Xylariales
Cligniaria_Coniochaetales
Amontagnei_Xylariales
Pvexata_Xylariales
Koryzae_Trichosphaeriales
Lthalassiae_Lulworthiales
Gtetrasperma_Sordariales
Cbulbillosum_Sordariales
Opiliferum_Ophiostomatales
Gclavigera_Ophiostomatales
Moryzae_Magnaporthales
Ggraminis_Magnaporthales
Mpoae_Magnaporthales
Paleophilum_Togniniales
Cpulchella_Calosphaeriales
Lminuta_Diaporthales
Dampelina_Diaporthales
Cchrysosperma_Diaporthales
Clustricola_Diaporthales
Cpopulea_Diaporthales
taxa
20
40
60
80
100
0
1
2
3
4
5
6
7
8
branch length

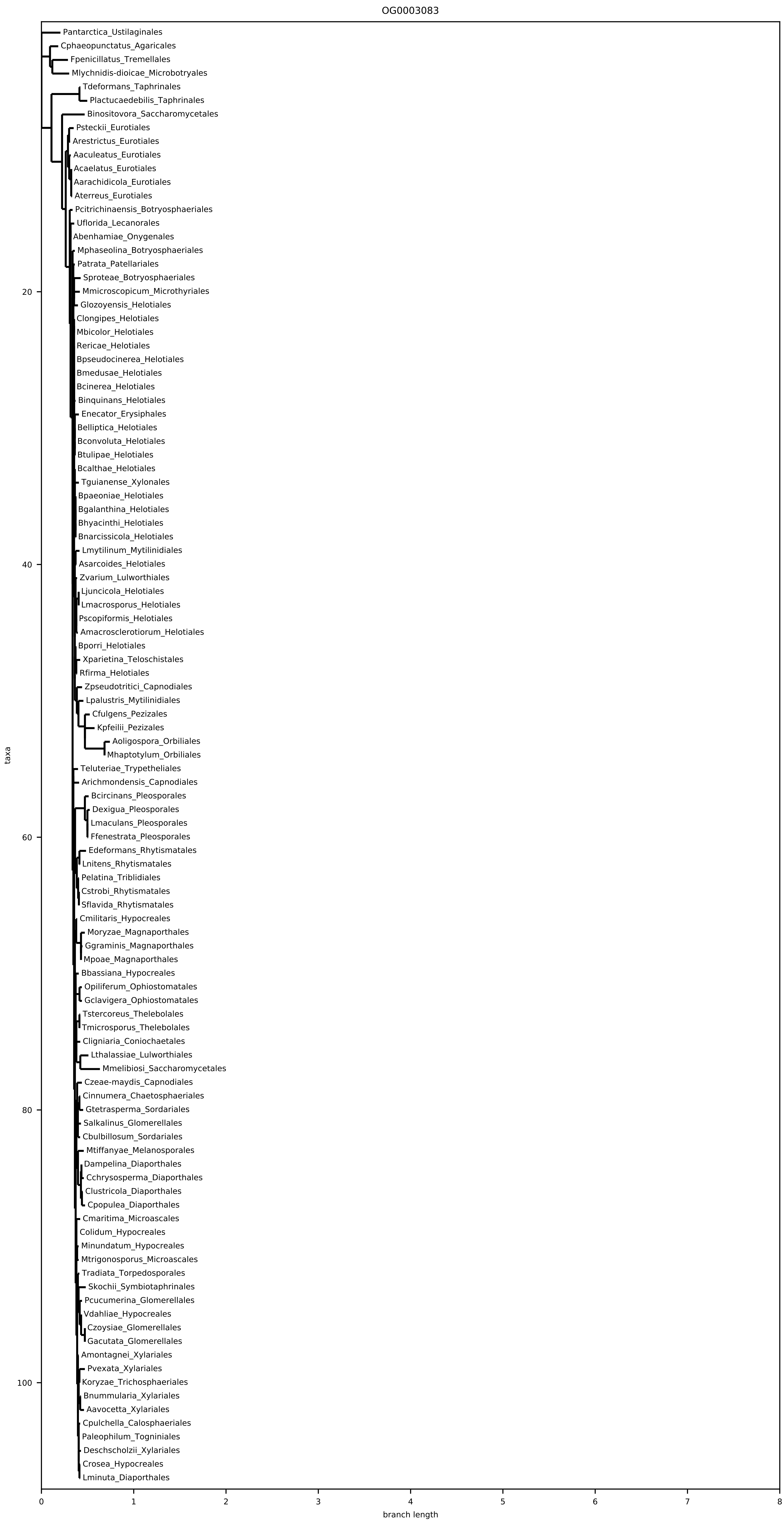
OG0003083
Pantarctica_Ustilaginales
Cphaeopunctatus_Agaricales
Fpenicillatus_Tremellales
Mlychnidis-dioicae_Microbotryales
Tdeformans_Taphrinales
Plactucaedebilis_Taphrinales
Binositovora_Saccharomycetales
Psteckii_Eurotiales
Arestrictus_Eurotiales
Aaculeatus_Eurotiales
Acaelatus_Eurotiales
Aarachidicola_Eurotiales
Aterreus_Eurotiales
Pcitrichinaensis_Botryosphaeriales
Uflorida_Lecanorales
Abenhamiae_Onygenales
Mphaseolina_Botryosphaeriales
Patrata_Patellariales
Sproteae_Botryosphaeriales
Mmicroscopicum_Microthyriales
Glozoyensis_Helotiales
Clongipes_Helotiales
Mbicolor_Helotiales
Rericae_Helotiales
Bpseudocinerea_Helotiales
Bmedusae_Helotiales
Bcinerea_Helotiales
Binquinans_Helotiales
Enecator_Erysiphales
Belliptica_Helotiales
Bconvoluta_Helotiales
Btulipae_Helotiales
Bcalthae_Helotiales
Tguianense_Xylonales
Bpaeoniae_Helotiales
Bgalanthina_Helotiales
Bhyacinthi_Helotiales
Bnarcissicola_Helotiales
Lmytilinum_Mytilinidiales
Asarcoides_Helotiales
Zvarium_Lulworthiales
Ljuncicola_Helotiales
Lmacrosporus_Helotiales
Pscopiformis_Helotiales
Amacrosclerotiorum_Helotiales
Bporri_Helotiales
Xparietina_Teloschistales
Rfirma_Helotiales
Zpseudotritici_Capnodiales
Lpalustris_Mytilinidiales
Cfulgens_Pezizales
Kpfeilii_Pezizales
Aoligospora_Orbiliales
Mhaptotylum_Orbiliales
Teluteriae_Trypetheliales
Arichmondensis_Capnodiales
Bcircinans_Pleosporales
Dexigua_Pleosporales
Lmaculans_Pleosporales
Ffenestrata_Pleosporales
Edeformans_Rhytismatales
Lnitens_Rhytismatales
Pelatina_Triblidiales
Cstrobi_Rhytismatales
Sflavida_Rhytismatales
Cmilitaris_Hypocreales
Moryzae_Magnaporthales
Ggraminis_Magnaporthales
Mpoae_Magnaporthales
Bbassiana_Hypocreales
Opiliferum_Ophiostomatales
Gclavigera_Ophiostomatales
Tstercoreus_Thelebolales
Tmicrosporus_Thelebolales
Cligniaria_Coniochaetales
Lthalassiae_Lulworthiales
Mmelibiosi_Saccharomycetales
Czeae-maydis_Capnodiales
Cinnumera_Chaetosphaeriales
Gtetrasperma_Sordariales
Salkalinus_Glomerellales
Cbulbillosum_Sordariales
Mtiffanyae_Melanosporales
Dampelina_Diaporthales
Cchrysosperma_Diaporthales
Clustricola_Diaporthales
Cpopulea_Diaporthales
Cmaritima_Microascales
Colidum_Hypocreales
Minundatum_Hypocreales
Mtrigonosporus_Microascales
Tradiata_Torpedosporales
Skochii_Symbiotaphrinales
Pcucumerina_Glomerellales
Vdahliae_Hypocreales
Czoysiae_Glomerellales
Gacutata_Glomerellales
Amontagnei_Xylariales
Pvexata_Xylariales
Koryzae_Trichosphaeriales
Bnummularia_Xylariales
Aavocetta_Xylariales
Cpulchella_Calosphaeriales
Paleophilum_Togniniales
Deschscholzii_Xylariales
Crosea_Hypocreales
Lminuta_Diaporthales
taxa
20
40
60
80
100
0
1
2
3
4
5
6
7
8
branch length

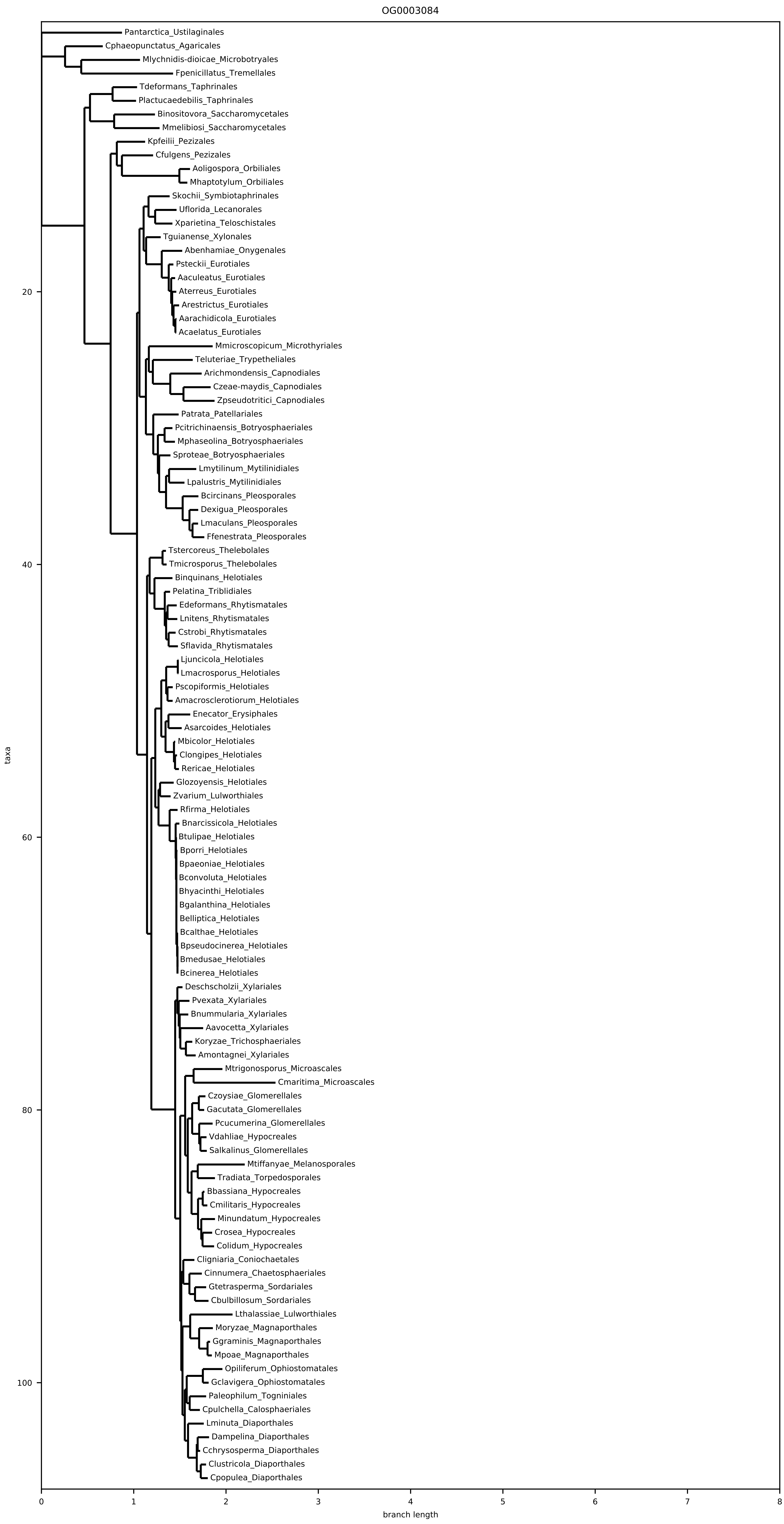
OG0003084
Pantarctica_Ustilaginales
Cphaeopunctatus_Agaricales
Mlychnidis-dioicae_Microbotryales
Fpenicillatus_Tremellales
Tdeformans_Taphrinales
Plactucaedebilis_Taphrinales
Binositovora_Saccharomycetales
Mmelibiosi_Saccharomycetales
Kpfeilii_Pezizales
Cfulgens_Pezizales
Aoligospora_Orbiliales
Mhaptotylum_Orbiliales
Skochii_Symbiotaphrinales
Uflorida_Lecanorales
Xparietina_Teloschistales
Tguianense_Xylonales
Abenhamiae_Onygenales
Psteckii_Eurotiales
Aaculeatus_Eurotiales
Aterreus_Eurotiales
Arestrictus_Eurotiales
Aarachidicola_Eurotiales
Acaelatus_Eurotiales
Mmicroscopicum_Microthyriales
Teluteriae_Trypetheliales
Arichmondensis_Capnodiales
Czeae-maydis_Capnodiales
Zpseudotritici_Capnodiales
Patrata_Patellariales
Pcitrichinaensis_Botryosphaeriales
Mphaseolina_Botryosphaeriales
Sproteae_Botryosphaeriales
Lmytilinum_Mytilinidiales
Lpalustris_Mytilinidiales
Bcircinans_Pleosporales
Dexigua_Pleosporales
Lmaculans_Pleosporales
Ffenestrata_Pleosporales
Tstercoreus_Thelebolales
Tmicrosporus_Thelebolales
Binquinans_Helotiales
Pelatina_Triblidiales
Edeformans_Rhytismatales
Lnitens_Rhytismatales
Cstrobi_Rhytismatales
Sflavida_Rhytismatales
Ljuncicola_Helotiales
Lmacrosporus_Helotiales
Pscopiformis_Helotiales
Amacrosclerotiorum_Helotiales
Enecator_Erysiphales
Asarcoides_Helotiales
Mbicolor_Helotiales
Clongipes_Helotiales
Rericae_Helotiales
Glozoyensis_Helotiales
Zvarium_Lulworthiales
Rfirma_Helotiales
Bnarcissicola_Helotiales
Btulipae_Helotiales
Bporri_Helotiales
Bpaeoniae_Helotiales
Bconvoluta_Helotiales
Bhyacinthi_Helotiales
Bgalanthina_Helotiales
Belliptica_Helotiales
Bcalthae_Helotiales
Bpseudocinerea_Helotiales
Bmedusae_Helotiales
Bcinerea_Helotiales
Deschscholzii_Xylariales
Pvexata_Xylariales
Bnummularia_Xylariales
Aavocetta_Xylariales
Koryzae_Trichosphaeriales
Amontagnei_Xylariales
Mtrigonosporus_Microascales
Cmaritima_Microascales
Czoysiae_Glomerellales
Gacutata_Glomerellales
Pcucumerina_Glomerellales
Vdahliae_Hypocreales
Salkalinus_Glomerellales
Mtiffanyae_Melanosporales
Tradiata_Torpedosporales
Bbassiana_Hypocreales
Cmilitaris_Hypocreales
Minundatum_Hypocreales
Crosea_Hypocreales
Colidum_Hypocreales
Cligniaria_Coniochaetales
Cinnumera_Chaetosphaeriales
Gtetrasperma_Sordariales
Cbulbillosum_Sordariales
Lthalassiae_Lulworthiales
Moryzae_Magnaporthales
Ggraminis_Magnaporthales
Mpoae_Magnaporthales
Opiliferum_Ophiostomatales
Gclavigera_Ophiostomatales
Paleophilum_Togniniales
Cpulchella_Calosphaeriales
Lminuta_Diaporthales
Dampelina_Diaporthales
Cchrysosperma_Diaporthales
Clustricola_Diaporthales
Cpopulea_Diaporthales
taxa
20
40
60
80
100
0
1
2
3
4
5
6
7
8
branch length

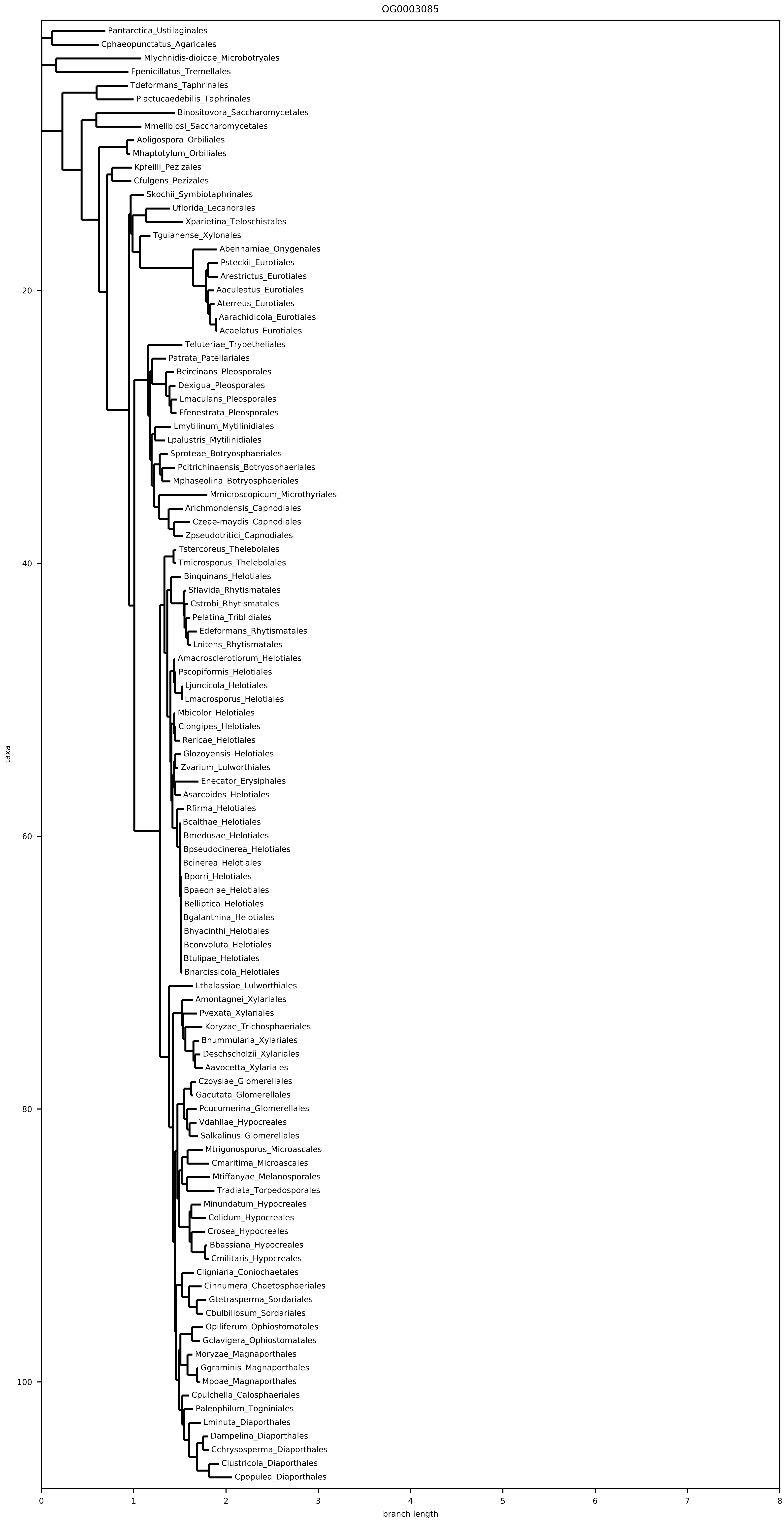
OG0003085
Pantarctica_Ustilaginales
Cphaeopunctatus_Agaricales
Mlychnidis-dioicae_Microbotryales
Fpenicillatus_Tremellales
Tdeformans_Taphrinales
Plactucaedebilis_Taphrinales
Binositovora_Saccharomycetales
Mmelibiosi_Saccharomycetales
Aoligospora_Orbiliales
Mhaptotylum_Orbiliales
Kpfeilii_Pezizales
Cfulgens_Pezizales
Skochii_Symbiotaphrinales
Uflorida_Lecanorales
Xparietina_Teloschistales
Tguianense_Xylonales
Abenhamiae_Onygenales
Psteckii_Eurotiales
Arestrictus_Eurotiales
Aaculeatus_Eurotiales
Aterreus_Eurotiales
Aarachidicola_Eurotiales
Acaelatus_Eurotiales
Teluteriae_Trypetheliales
Patrata_Patellariales
Bcircinans_Pleosporales
Dexigua_Pleosporales
Lmaculans_Pleosporales
Ffenestrata_Pleosporales
Lmytilinum_Mytilinidiales
Lpalustris_Mytilinidiales
Sproteae_Botryosphaeriales
Pcitrichinaensis_Botryosphaeriales
Mphaseolina_Botryosphaeriales
Mmicroscopicum_Microthyriales
Arichmondensis_Capnodiales
Czeae-maydis_Capnodiales
Zpseudotritici_Capnodiales
Tstercoreus_Thelebolales
Tmicrosporus_Thelebolales
Binquinans_Helotiales
Sflavida_Rhytismatales
Cstrobi_Rhytismatales
Pelatina_Triblidiales
Edeformans_Rhytismatales
Lnitens_Rhytismatales
Amacrosclerotiorum_Helotiales
Pscopiformis_Helotiales
Ljuncicola_Helotiales
Lmacrosporus_Helotiales
Mbicolor_Helotiales
Clongipes_Helotiales
Rericae_Helotiales
Glozoyensis_Helotiales
Zvarium_Lulworthiales
Enecator_Erysiphales
Asarcoides_Helotiales
Rfirma_Helotiales
Bcalthae_Helotiales
Bmedusae_Helotiales
Bpseudocinerea_Helotiales
Bcinerea_Helotiales
Bporri_Helotiales
Bpaeoniae_Helotiales
Belliptica_Helotiales
Bgalanthina_Helotiales
Bhyacinthi_Helotiales
Bconvoluta_Helotiales
Btulipae_Helotiales
Bnarcissicola_Helotiales
Lthalassiae_Lulworthiales
Amontagnei_Xylariales
Pvexata_Xylariales
Koryzae_Trichosphaeriales
Bnummularia_Xylariales
Deschscholzii_Xylariales
Aavocetta_Xylariales
Czoysiae_Glomerellales
Gacutata_Glomerellales
Pcucumerina_Glomerellales
Vdahliae_Hypocreales
Salkalinus_Glomerellales
Mtrigonosporus_Microascales
Cmaritima_Microascales
Mtiffanyae_Melanosporales
Tradiata_Torpedosporales
Minundatum_Hypocreales
Colidum_Hypocreales
Crosea_Hypocreales
Bbassiana_Hypocreales
Cmilitaris_Hypocreales
Cligniaria_Coniochaetales
Cinnumera_Chaetosphaeriales
Gtetrasperma_Sordariales
Cbulbillosum_Sordariales
Opiliferum_Ophiostomatales
Gclavigera_Ophiostomatales
Moryzae_Magnaporthales
Ggraminis_Magnaporthales
Mpoae_Magnaporthales
Cpulchella_Calosphaeriales
Paleophilum_Togniniales
Lminuta_Diaporthales
Dampelina_Diaporthales
Cchrysosperma_Diaporthales
Clustricola_Diaporthales
Cpopulea_Diaporthales
taxa
20
40
60
80
100
0
1
2
3
4
5
6
7
8
branch length

OG0003087

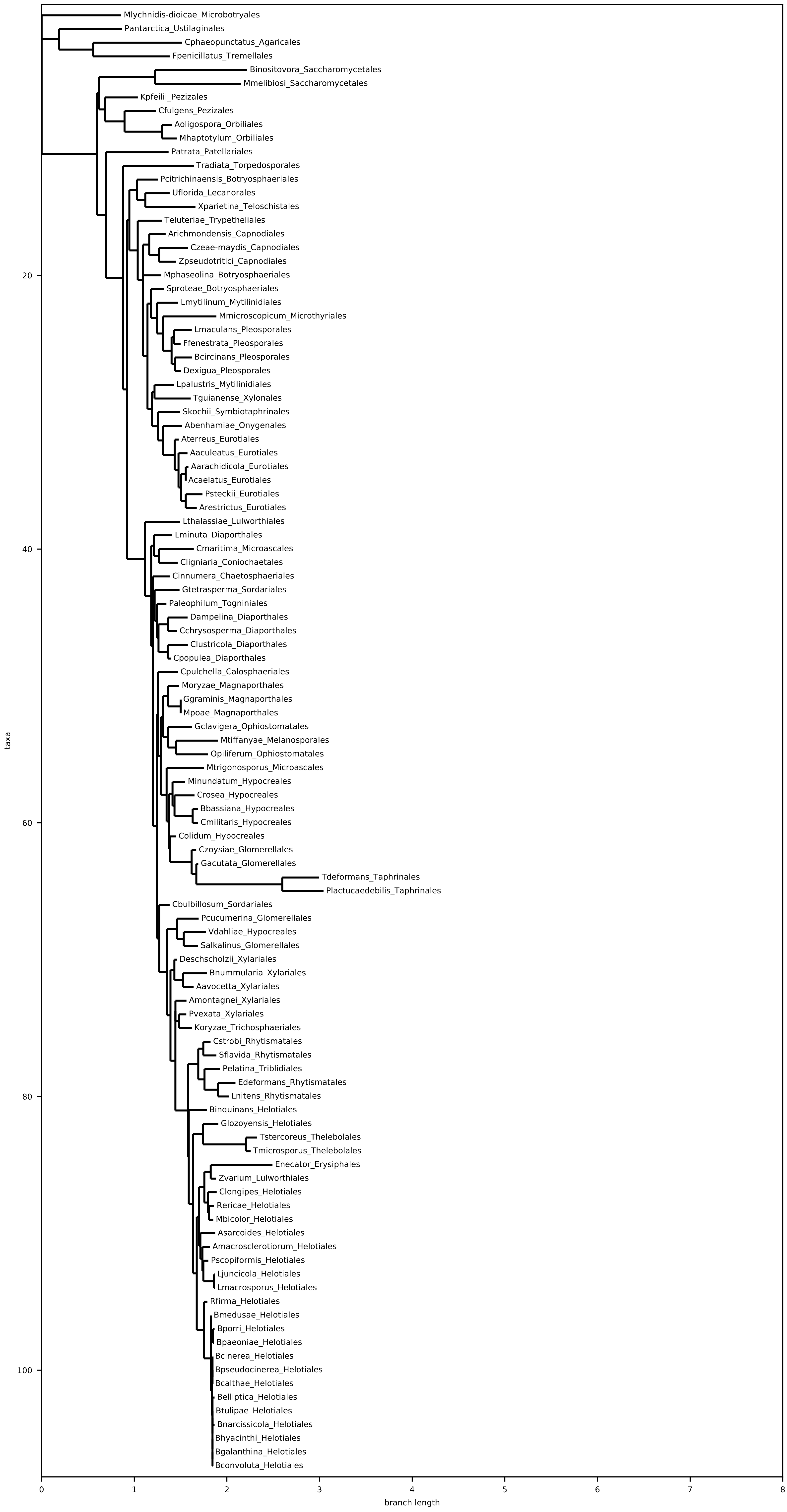
Mlychnidis-dioicae_Microbotryales
Pantarctica_Ustilaginales
Cphaeopunctatus_Agaricales
Fpenicillatus_Tremellales
Binositovora_Saccharomycetales
Mmelibiosi_Saccharomycetales
Kpfeilii_Pezizales
Cfulgens_Pezizales
Aoligospora_Orbiliales
Mhaptotylum_Orbiliales
Patrata_Patellariales
Tradiata_Torpedosporales
Pcitrichinaensis_Botryosphaeriales
Uflorida_Lecanorales
Xparietina_Teloschistales
Teluteriae_Trypetheliales
Arichmondensis_Capnodiales
Czeae-maydis_Capnodiales
Zpseudotritici_Capnodiales
Mphaseolina_Botryosphaeriales
Sproteae_Botryosphaeriales
Lmytilinum_Mytilinidiales
Mmicroscopicum_Microthyriales
Lmaculans_Pleosporales
Ffenestrata_Pleosporales
Bcircinans_Pleosporales
Dexigua_Pleosporales
Lpalustris_Mytilinidiales
Tguianense_Xylonales
Skochii_Symbiotaphrinales
Abenhamiae_Onygenales
Aterreus_Eurotiales
Aaculeatus_Eurotiales
Aarachidicola_Eurotiales
Acaelatus_Eurotiales
Psteckii_Eurotiales
Arestrictus_Eurotiales
Lthalassiae_Lulworthiales
Lminuta_Diaporthales
Cmaritima_Microascales
Cligniaria_Coniochaetales
Cinnumera_Chaetosphaeriales
Gtetrasperma_Sordariales
Paleophilum_Togniniales
Dampelina_Diaporthales
Cchrysosperma_Diaporthales
Clustricola_Diaporthales
Cpopulea_Diaporthales
Cpulchella_Calosphaeriales
Moryzae_Magnaporthales
Ggraminis_Magnaporthales
Mpoae_Magnaporthales
Gclavigera_Ophiostomatales
Mtiffanyae_Melanosporales
Opiliferum_Ophiostomatales
Mtrigonosporus_Microascales
Minundatum_Hypocreales
Crosea_Hypocreales
Bbassiana_Hypocreales
Cmilitaris_Hypocreales
Colidum_Hypocreales
Czoysiae_Glomerellales
Gacutata_Glomerellales
Tdeformans_Taphrinales
Plactucaedebilis_Taphrinales
Cbulbillosum_Sordariales
Pcucumerina_Glomerellales
Vdahliae_Hypocreales
Salkalinus_Glomerellales
Deschscholzii_Xylariales
Bnummularia_Xylariales
Aavocetta_Xylariales
Amontagnei_Xylariales
Pvexata_Xylariales
Koryzae_Trichosphaeriales
Cstrobi_Rhytismatales
Sflavida_Rhytismatales
Pelatina_Triblidiales
Edeformans_Rhytismatales
Lnitens_Rhytismatales
Binquinans_Helotiales
Glozoyensis_Helotiales
Tstercoreus_Thelebolales
Tmicrosporus_Thelebolales
Enecator_Erysiphales
Zvarium_Lulworthiales
Clongipes_Helotiales
Rericae_Helotiales
Mbicolor_Helotiales
Asarcoides_Helotiales
Amacrosclerotiorum_Helotiales
Pscopiformis_Helotiales
Ljuncicola_Helotiales
Lmacrosporus_Helotiales
Rfirma_Helotiales
Bmedusae_Helotiales
Bporri_Helotiales
Bpaeoniae_Helotiales
Bcinerea_Helotiales
Bpseudocinerea_Helotiales
Bcalthae_Helotiales
Belliptica_Helotiales
Btulipae_Helotiales
Bnarcissicola_Helotiales
Bhyacinthi_Helotiales
Bgalanthina_Helotiales
Bconvoluta_Helotiales
taxa
20
40
60
80
100
0
1
2
3
4
5
6
7
8
branch length

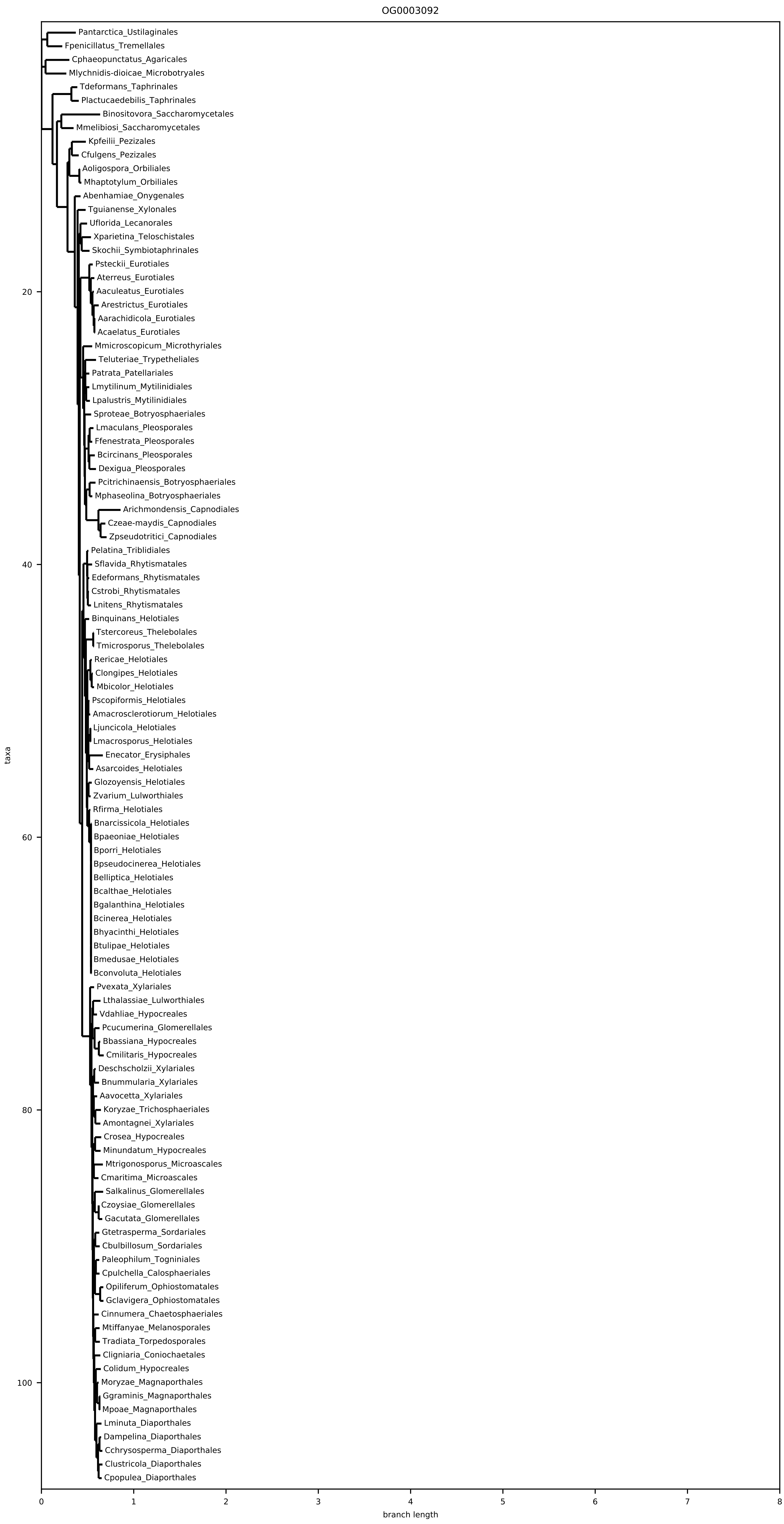

OG0003092
Pantarctica_Ustilaginales
Fpenicillatus_Tremellales
Cphaeopunctatus_Agaricales
Mlychnidis-dioicae_Microbotryales
Tdeformans_Taphrinales
Plactucaedebilis_Taphrinales
Binositovora_Saccharomycetales
Mmelibiosi_Saccharomycetales
Kpfeilii_Pezizales
Cfulgens_Pezizales
Aoligospora_Orbiliales
Mhaptotylum_Orbiliales
Abenhamiae_Onygenales
Tguianense_Xylonales
Uflorida_Lecanorales
Xparietina_Teloschistales
Skochii_Symbiotaphrinales
Psteckii_Eurotiales
Aterreus_Eurotiales
Aaculeatus_Eurotiales
Arestrictus_Eurotiales
Aarachidicola_Eurotiales
Acaelatus_Eurotiales
Mmicroscopicum_Microthyriales
Teluteriae_Trypetheliales
Patrata_Patellariales
Lmytilinum_Mytilinidiales
Lpalustris_Mytilinidiales
Sproteae_Botryosphaeriales
Lmaculans_Pleosporales
Ffenestrata_Pleosporales
Bcircinans_Pleosporales
Dexigua_Pleosporales
Pcitrichinaensis_Botryosphaeriales
Mphaseolina_Botryosphaeriales
Arichmondensis_Capnodiales
Czeae-maydis_Capnodiales
Zpseudotritici_Capnodiales
Pelatina_Triblidiales
Sflavida_Rhytismatales
Edeformans_Rhytismatales
Cstrobi_Rhytismatales
Lnitens_Rhytismatales
Binquinans_Helotiales
Tstercoreus_Thelebolales
Tmicrosporus_Thelebolales
Rericae_Helotiales
Clongipes_Helotiales
Mbicolor_Helotiales
Pscopiformis_Helotiales
Amacrosclerotiorum_Helotiales
Ljuncicola_Helotiales
Lmacrosporus_Helotiales
Enecator_Erysiphales
Asarcoides_Helotiales
Glozoyensis_Helotiales
Zvarium_Lulworthiales
Rfirma_Helotiales
Bnarcissicola_Helotiales
Bpaeoniae_Helotiales
Bporri_Helotiales
Bpseudocinerea_Helotiales
Belliptica_Helotiales
Bcalthae_Helotiales
Bgalanthina_Helotiales
Bcinerea_Helotiales
Bhyacinthi_Helotiales
Btulipae_Helotiales
Bmedusae_Helotiales
Bconvoluta_Helotiales
Pvexata_Xylariales
Lthalassiae_Lulworthiales
Vdahliae_Hypocreales
Pcucumerina_Glomerellales
Bbassiana_Hypocreales
Cmilitaris_Hypocreales
Deschscholzii_Xylariales
Bnummularia_Xylariales
Aavocetta_Xylariales
Koryzae_Trichosphaeriales
Amontagnei_Xylariales
Crosea_Hypocreales
Minundatum_Hypocreales
Mtrigonosporus_Microascales
Cmaritima_Microascales
Salkalinus_Glomerellales
Czoysiae_Glomerellales
Gacutata_Glomerellales
Gtetrasperma_Sordariales
Cbulbillosum_Sordariales
Paleophilum_Togniniales
Cpulchella_Calosphaeriales
Opiliferum_Ophiostomatales
Gclavigera_Ophiostomatales
Cinnumera_Chaetosphaeriales
Mtiffanyae_Melanosporales
Tradiata_Torpedosporales
Cligniaria_Coniochaetales
Colidum_Hypocreales
Moryzae_Magnaporthales
Ggraminis_Magnaporthales
Mpoae_Magnaporthales
Lminuta_Diaporthales
Dampelina_Diaporthales
Cchrysosperma_Diaporthales
Clustricola_Diaporthales
Cpopulea_Diaporthales
taxa
20
40
60
80
100
0
1
2
3
4
5
6
7
8
branch length

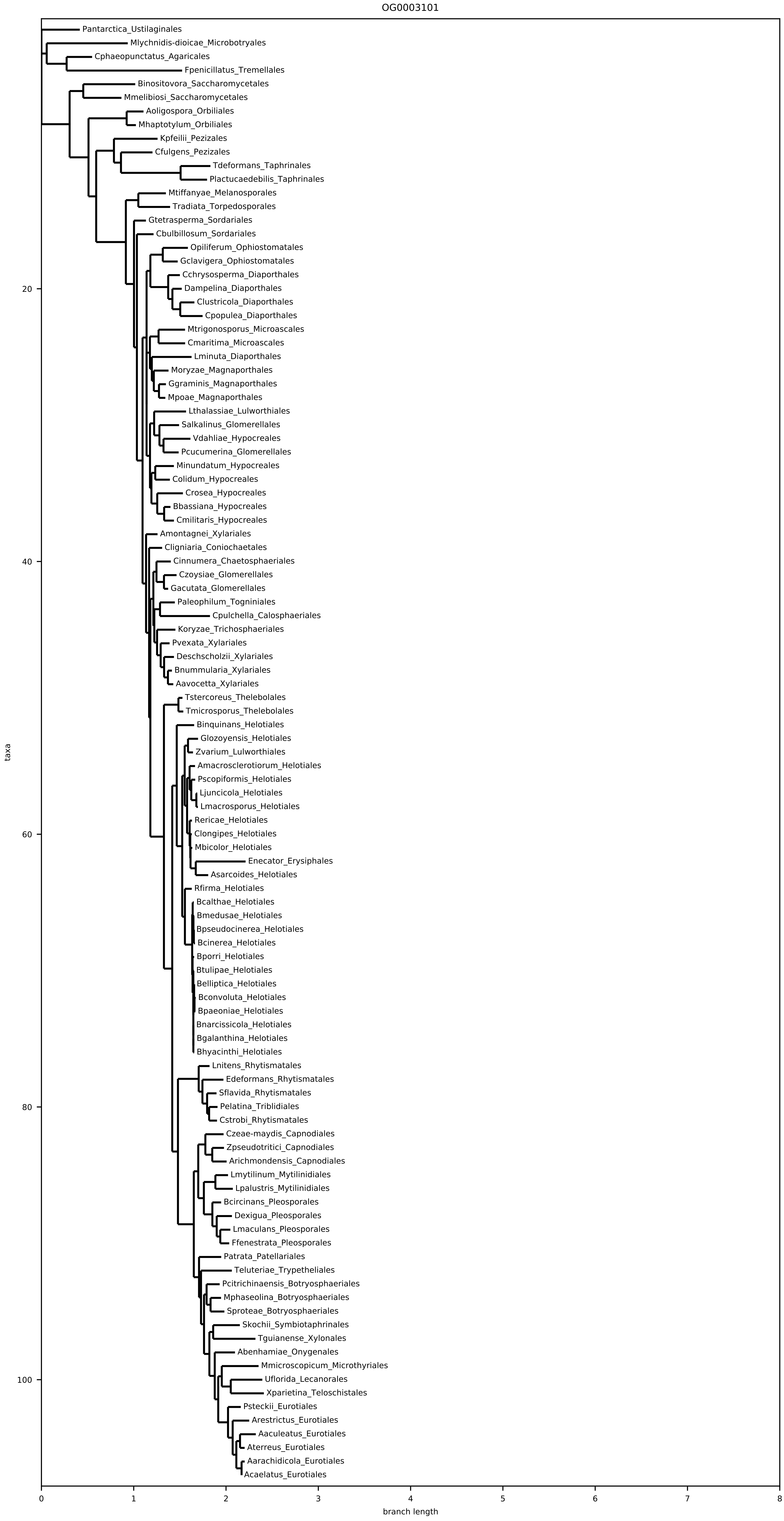
OG0003101
Pantarctica_Ustilaginales
Mlychnidis-dioicae_Microbotryales
Cphaeopunctatus_Agaricales
Fpenicillatus_Tremellales
Binositovora_Saccharomycetales
Mmelibiosi_Saccharomycetales
Aoligospora_Orbiliales
Mhaptotylum_Orbiliales
Kpfeilii_Pezizales
Cfulgens_Pezizales
Tdeformans_Taphrinales
Plactucaedebilis_Taphrinales
Mtiffanyae_Melanosporales
Tradiata_Torpedosporales
Gtetrasperma_Sordariales
Cbulbillosum_Sordariales
Opiliferum_Ophiostomatales
Gclavigera_Ophiostomatales
Cchrysosperma_Diaporthales
Dampelina_Diaporthales
Clustricola_Diaporthales
Cpopulea_Diaporthales
Mtrigonosporus_Microascales
Cmaritima_Microascales
Lminuta_Diaporthales
Moryzae_Magnaporthales
Ggraminis_Magnaporthales
Mpoae_Magnaporthales
Lthalassiae_Lulworthiales
Salkalinus_Glomerellales
Vdahliae_Hypocreales
Pcucumerina_Glomerellales
Minundatum_Hypocreales
Colidum_Hypocreales
Crosea_Hypocreales
Bbassiana_Hypocreales
Cmilitaris_Hypocreales
Amontagnei_Xylariales
Cligniaria_Coniochaetales
Cinnumera_Chaetosphaeriales
Czoysiae_Glomerellales
Gacutata_Glomerellales
Paleophilum_Togniniales
Cpulchella_Calosphaeriales
Koryzae_Trichosphaeriales
Pvexata_Xylariales
Deschscholzii_Xylariales
Bnummularia_Xylariales
Aavocetta_Xylariales
Tstercoreus_Thelebolales
Tmicrosporus_Thelebolales
Binquinans_Helotiales
Glozoyensis_Helotiales
Zvarium_Lulworthiales
Amacrosclerotiorum_Helotiales
Pscopiformis_Helotiales
Ljuncicola_Helotiales
Lmacrosporus_Helotiales
Rericae_Helotiales
Clongipes_Helotiales
Mbicolor_Helotiales
Enecator_Erysiphales
Asarcoides_Helotiales
Rfirma_Helotiales
Bcalthae_Helotiales
Bmedusae_Helotiales
Bpseudocinerea_Helotiales
Bcinerea_Helotiales
Bporri_Helotiales
Btulipae_Helotiales
Belliptica_Helotiales
Bconvoluta_Helotiales
Bpaeoniae_Helotiales
Bnarcissicola_Helotiales
Bgalanthina_Helotiales
Bhyacinthi_Helotiales
Lnitens_Rhytismatales
Edeformans_Rhytismatales
Sflavida_Rhytismatales
Pelatina_Triblidiales
Cstrobi_Rhytismatales
Czeae-maydis_Capnodiales
Zpseudotritici_Capnodiales
Arichmondensis_Capnodiales
Lmytilinum_Mytilinidiales
Lpalustris_Mytilinidiales
Bcircinans_Pleosporales
Dexigua_Pleosporales
Lmaculans_Pleosporales
Ffenestrata_Pleosporales
Patrata_Patellariales
Teluteriae_Trypetheliales
Pcitrichinaensis_Botryosphaeriales
Mphaseolina_Botryosphaeriales
Sproteae_Botryosphaeriales
Skochii_Symbiotaphrinales
Tguianense_Xylonales
Abenhamiae_Onygenales
Mmicroscopicum_Microthyriales
Uflorida_Lecanorales
Xparietina_Teloschistales
Psteckii_Eurotiales
Arestrictus_Eurotiales
Aaculeatus_Eurotiales
Aterreus_Eurotiales
Aarachidicola_Eurotiales
Acaelatus_Eurotiales
taxa
20
40
60
80
100
0
1
2
3
4
5
6
7
8
branch length

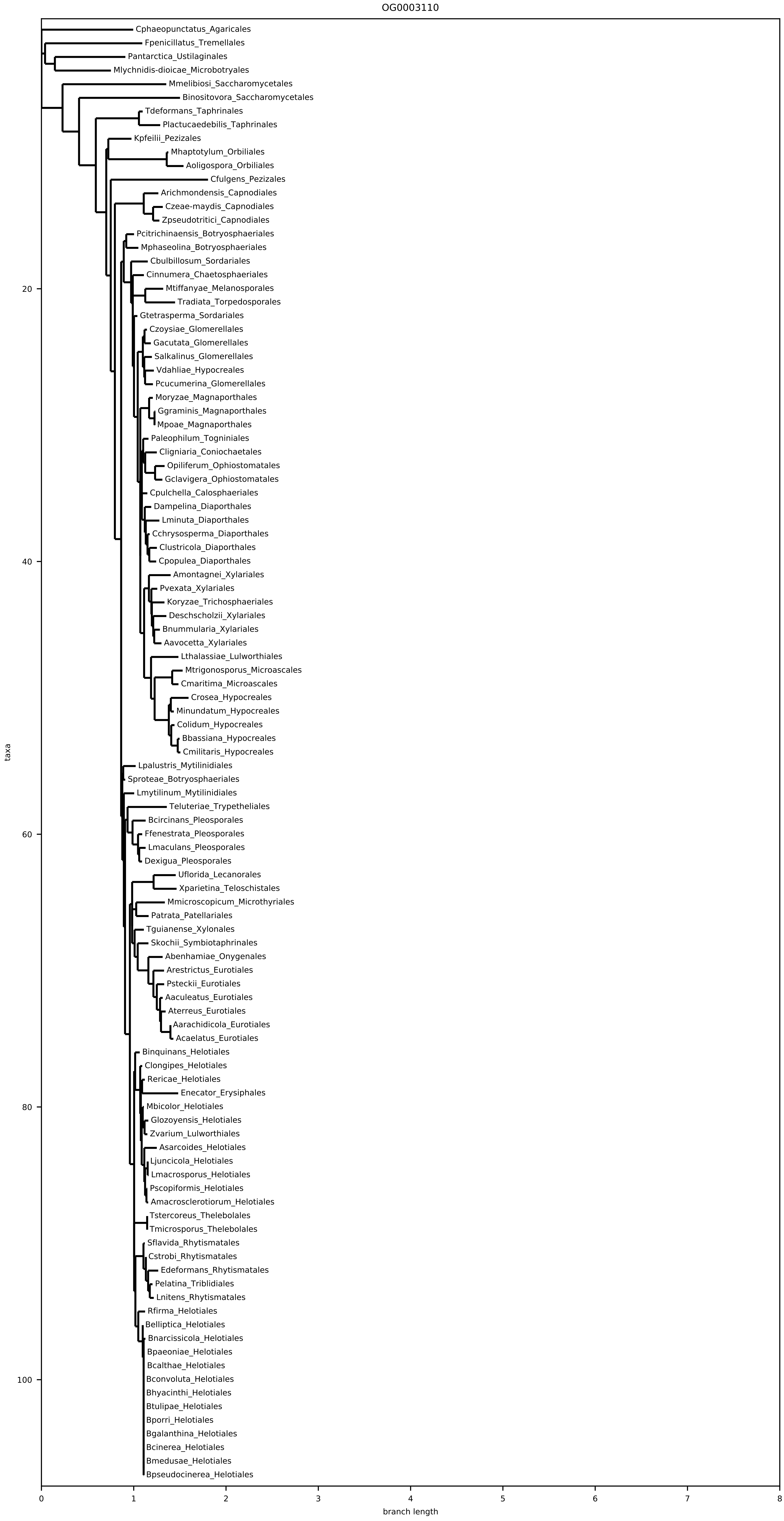
OG0003110
Cphaeopunctatus_Agaricales
Fpenicillatus_Tremellales
Pantarctica_Ustilaginales
Mlychnidis-dioicae_Microbotryales
Mmelibiosi_Saccharomycetales
Binositovora_Saccharomycetales
Tdeformans_Taphrinales
Plactucaedebilis_Taphrinales
Kpfeilii_Pezizales
Mhaptotylum_Orbiliales
Aoligospora_Orbiliales
Cfulgens_Pezizales
Arichmondensis_Capnodiales
Czeae-maydis_Capnodiales
Zpseudotritici_Capnodiales
Pcitrichinaensis_Botryosphaeriales
Mphaseolina_Botryosphaeriales
Cbulbillosum_Sordariales
Cinnumera_Chaetosphaeriales
Mtiffanyae_Melanosporales
Tradiata_Torpedosporales
Gtetrasperma_Sordariales
Czoysiae_Glomerellales
Gacutata_Glomerellales
Salkalinus_Glomerellales
Vdahliae_Hypocreales
Pcucumerina_Glomerellales
Moryzae_Magnaporthales
Ggraminis_Magnaporthales
Mpoae_Magnaporthales
Paleophilum_Togniniales
Cligniaria_Coniochaetales
Opiliferum_Ophiostomatales
Gclavigera_Ophiostomatales
Cpulchella_Calosphaeriales
Dampelina_Diaporthales
Lminuta_Diaporthales
Cchrysosperma_Diaporthales
Clustricola_Diaporthales
Cpopulea_Diaporthales
Amontagnei_Xylariales
Pvexata_Xylariales
Koryzae_Trichosphaeriales
Deschscholzii_Xylariales
Bnummularia_Xylariales
Aavocetta_Xylariales
Lthalassiae_Lulworthiales
Mtrigonosporus_Microascales
Cmaritima_Microascales
Crosea_Hypocreales
Minundatum_Hypocreales
Colidum_Hypocreales
Bbassiana_Hypocreales
Cmilitaris_Hypocreales
Lpalustris_Mytilinidiales
Sproteae_Botryosphaeriales
Lmytilinum_Mytilinidiales
Teluteriae_Trypetheliales
Bcircinans_Pleosporales
Ffenestrata_Pleosporales
Lmaculans_Pleosporales
Dexigua_Pleosporales
Uflorida_Lecanorales
Xparietina_Teloschistales
Mmicroscopicum_Microthyriales
Patrata_Patellariales
Tguianense_Xylonales
Skochii_Symbiotaphrinales
Abenhamiae_Onygenales
Arestrictus_Eurotiales
Psteckii_Eurotiales
Aaculeatus_Eurotiales
Aterreus_Eurotiales
Aarachidicola_Eurotiales
Acaelatus_Eurotiales
Binquinans_Helotiales
Clongipes_Helotiales
Rericae_Helotiales
Enecator_Erysiphales
Mbicolor_Helotiales
Glozoyensis_Helotiales
Zvarium_Lulworthiales
Asarcoides_Helotiales
Ljuncicola_Helotiales
Lmacrosporus_Helotiales
Pscopiformis_Helotiales
Amacrosclerotiorum_Helotiales
Tstercoreus_Thelebolales
Tmicrosporus_Thelebolales
Sflavida_Rhytismatales
Cstrobi_Rhytismatales
Edeformans_Rhytismatales
Pelatina_Triblidiales
Lnitens_Rhytismatales
Rfirma_Helotiales
Belliptica_Helotiales
Bnarcissicola_Helotiales
Bpaeoniae_Helotiales
Bcalthae_Helotiales
Bconvoluta_Helotiales
Bhyacinthi_Helotiales
Btulipae_Helotiales
Bporri_Helotiales
Bgalanthina_Helotiales
Bcinerea_Helotiales
Bmedusae_Helotiales
Bpseudocinerea_Helotiales
taxa
20
40
60
80
100
0
1
2
3
4
5
6
7
8
branch length

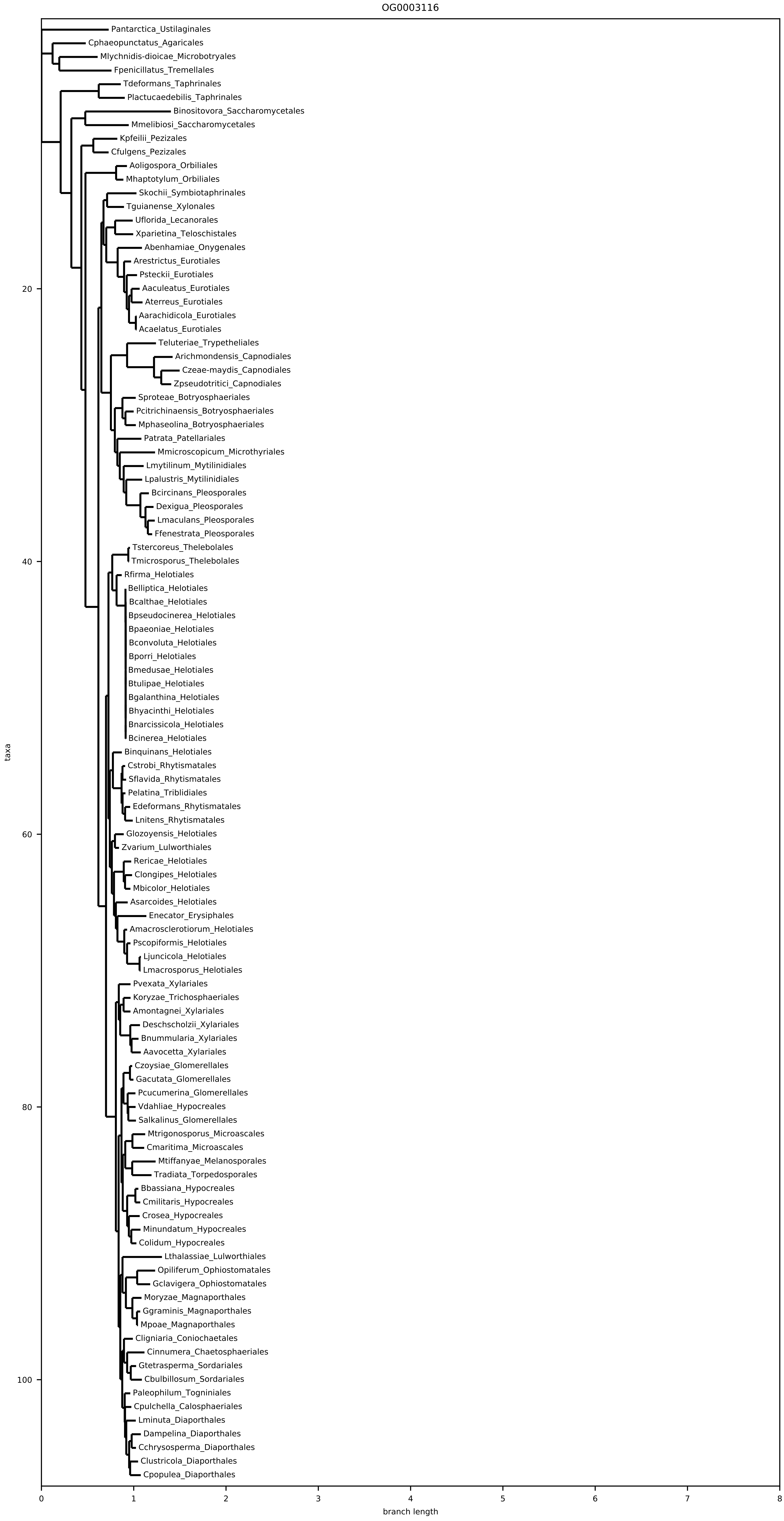
OG0003116
Pantarctica_Ustilaginales
Cphaeopunctatus_Agaricales
Mlychnidis-dioicae_Microbotryales
Fpenicillatus_Tremellales
Tdeformans_Taphrinales
Plactucaedebilis_Taphrinales
Binositovora_Saccharomycetales
Mmelibiosi_Saccharomycetales
Kpfeilii_Pezizales
Cfulgens_Pezizales
Aoligospora_Orbiliales
Mhaptotylum_Orbiliales
Skochii_Symbiotaphrinales
Tguianense_Xylonales
Uflorida_Lecanorales
Xparietina_Teloschistales
Abenhamiae_Onygenales
Arestrictus_Eurotiales
Psteckii_Eurotiales
Aaculeatus_Eurotiales
Aterreus_Eurotiales
Aarachidicola_Eurotiales
Acaelatus_Eurotiales
Teluteriae_Trypetheliales
Arichmondensis_Capnodiales
Czeae-maydis_Capnodiales
Zpseudotritici_Capnodiales
Sproteae_Botryosphaeriales
Pcitrichinaensis_Botryosphaeriales
Mphaseolina_Botryosphaeriales
Patrata_Patellariales
Mmicroscopicum_Microthyriales
Lmytilinum_Mytilinidiales
Lpalustris_Mytilinidiales
Bcircinans_Pleosporales
Dexigua_Pleosporales
Lmaculans_Pleosporales
Ffenestrata_Pleosporales
Tstercoreus_Thelebolales
Tmicrosporus_Thelebolales
Rfirma_Helotiales
Belliptica_Helotiales
Bcalthae_Helotiales
Bpseudocinerea_Helotiales
Bpaeoniae_Helotiales
Bconvoluta_Helotiales
Bporri_Helotiales
Bmedusae_Helotiales
Btulipae_Helotiales
Bgalanthina_Helotiales
Bhyacinthi_Helotiales
Bnarcissicola_Helotiales
Bcinerea_Helotiales
Binquinans_Helotiales
Cstrobi_Rhytismatales
Sflavida_Rhytismatales
Pelatina_Triblidiales
Edeformans_Rhytismatales
Lnitens_Rhytismatales
Glozoyensis_Helotiales
Zvarium_Lulworthiales
Rericae_Helotiales
Clongipes_Helotiales
Mbicolor_Helotiales
Asarcoides_Helotiales
Enecator_Erysiphales
Amacrosclerotiorum_Helotiales
Pscopiformis_Helotiales
Ljuncicola_Helotiales
Lmacrosporus_Helotiales
Pvexata_Xylariales
Koryzae_Trichosphaeriales
Amontagnei_Xylariales
Deschscholzii_Xylariales
Bnummularia_Xylariales
Aavocetta_Xylariales
Czoysiae_Glomerellales
Gacutata_Glomerellales
Pcucumerina_Glomerellales
Vdahliae_Hypocreales
Salkalinus_Glomerellales
Mtrigonosporus_Microascales
Cmaritima_Microascales
Mtiffanyae_Melanosporales
Tradiata_Torpedosporales
Bbassiana_Hypocreales
Cmilitaris_Hypocreales
Crosea_Hypocreales
Minundatum_Hypocreales
Colidum_Hypocreales
Lthalassiae_Lulworthiales
Opiliferum_Ophiostomatales
Gclavigera_Ophiostomatales
Moryzae_Magnaporthales
Ggraminis_Magnaporthales
Mpoae_Magnaporthales
Cligniaria_Coniochaetales
Cinnumera_Chaetosphaeriales
Gtetrasperma_Sordariales
Cbulbillosum_Sordariales
Paleophilum_Togniniales
Cpulchella_Calosphaeriales
Lminuta_Diaporthales
Dampelina_Diaporthales
Cchrysosperma_Diaporthales
Clustricola_Diaporthales
Cpopulea_Diaporthales
taxa
20
40
60
80
100
0
1
2
3
4
5
6
7
8
branch length

OG0003123

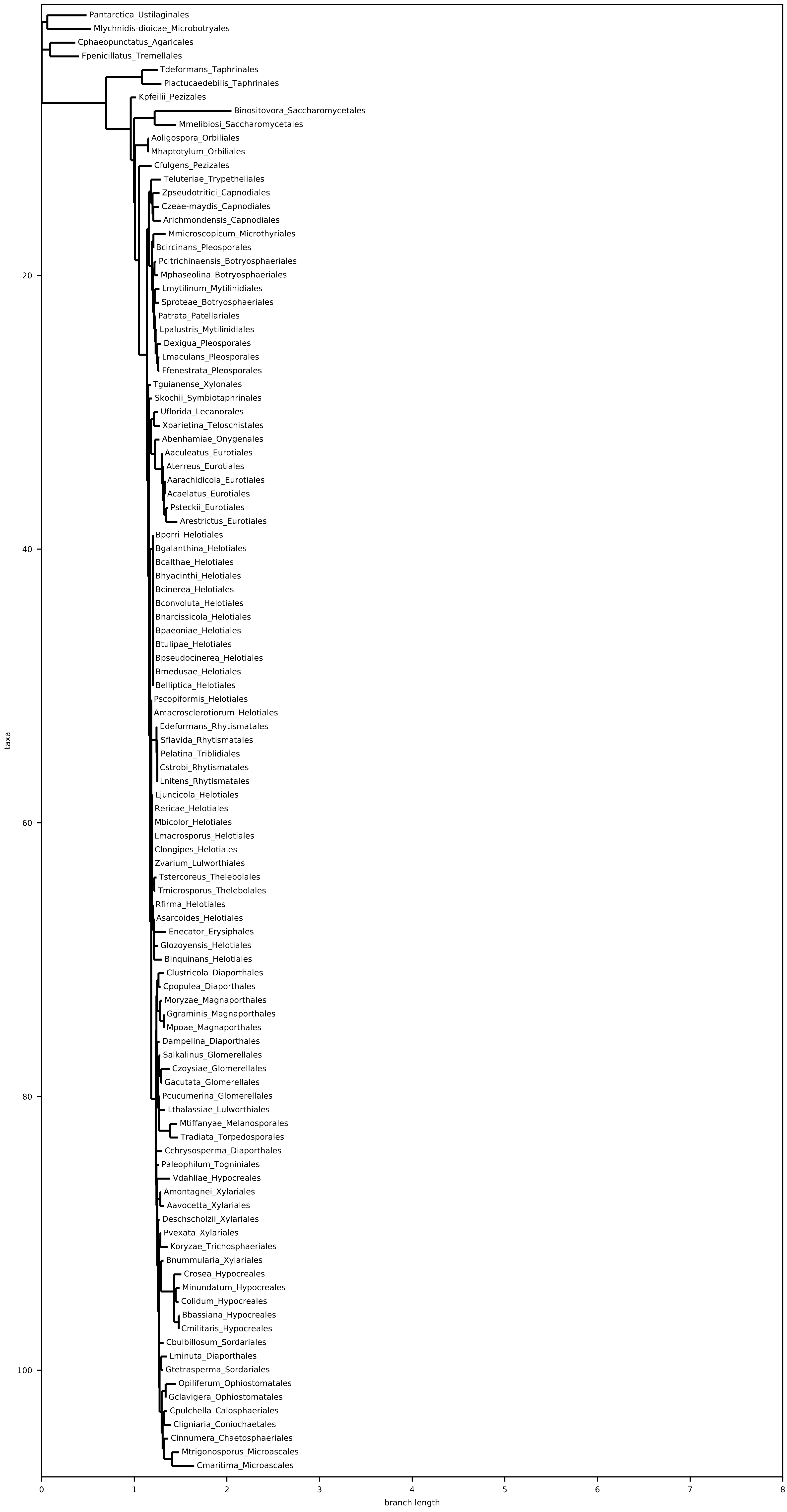
Pantarctica_Ustilaginales
Mlychnidis-dioicae_Microbotryales
Cphaeopunctatus_Agaricales
Fpenicillatus_Tremellales
Tdeformans_Taphrinales
Plactucaedebilis_Taphrinales
Kpfeilii_Pezizales
Binositovora_Saccharomycetales
Mmelibiosi_Saccharomycetales
Aoligospora_Orbiliales
Mhaptotylum_Orbiliales
Cfulgens_Pezizales
Teluteriae_Trypetheliales
Zpseudotritici_Capnodiales
Czeae-maydis_Capnodiales
Arichmondensis_Capnodiales
Mmicroscopicum_Microthyriales
Bcircinans_Pleosporales
Pcitrichinaensis_Botryosphaeriales
Mphaseolina_Botryosphaeriales
Lmytilinum_Mytilinidiales
Sproteae_Botryosphaeriales
Patrata_Patellariales
Lpalustris_Mytilinidiales
Dexigua_Pleosporales
Lmaculans_Pleosporales
Ffenestrata_Pleosporales
Tguianense_Xylonales
Skochii_Symbiotaphrinales
Uflorida_Lecanorales
Xparietina_Teloschistales
Abenhamiae_Onygenales
Aaculeatus_Eurotiales
Aterreus_Eurotiales
Aarachidicola_Eurotiales
Acaelatus_Eurotiales
Psteckii_Eurotiales
Arestrictus_Eurotiales
Bporri_Helotiales
Bgalanthina_Helotiales
Bcalthae_Helotiales
Bhyacinthi_Helotiales
Bcinerea_Helotiales
Bconvoluta_Helotiales
Bnarcissicola_Helotiales
Bpaeoniae_Helotiales
Btulipae_Helotiales
Bpseudocinerea_Helotiales
Bmedusae_Helotiales
Belliptica_Helotiales
Pscopiformis_Helotiales
Amacrosclerotiorum_Helotiales
Edeformans_Rhytismatales
Sflavida_Rhytismatales
Pelatina_Triblidiales
Cstrobi_Rhytismatales
Lnitens_Rhytismatales
Ljuncicola_Helotiales
Rericae_Helotiales
Mbicolor_Helotiales
Lmacrosporus_Helotiales
Clongipes_Helotiales
Zvarium_Lulworthiales
Tstercoreus_Thelebolales
Tmicrosporus_Thelebolales
Rfirma_Helotiales
Asarcoides_Helotiales
Enecator_Erysiphales
Glozoyensis_Helotiales
Binquinans_Helotiales
Clustricola_Diaporthales
Cpopulea_Diaporthales
Moryzae_Magnaporthales
Ggraminis_Magnaporthales
Mpoae_Magnaporthales
Dampelina_Diaporthales
Salkalinus_Glomerellales
Czoysiae_Glomerellales
Gacutata_Glomerellales
Pcucumerina_Glomerellales
Lthalassiae_Lulworthiales
Mtiffanyae_Melanosporales
Tradiata_Torpedosporales
Cchrysosperma_Diaporthales
Paleophilum_Togniniales
Vdahliae_Hypocreales
Amontagnei_Xylariales
Aavocetta_Xylariales
Deschscholzii_Xylariales
Pvexata_Xylariales
Koryzae_Trichosphaeriales
Bnummularia_Xylariales
Crosea_Hypocreales
Minundatum_Hypocreales
Colidum_Hypocreales
Bbassiana_Hypocreales
Cmilitaris_Hypocreales
Cbulbillosum_Sordariales
Lminuta_Diaporthales
Gtetrasperma_Sordariales
Opiliferum_Ophiostomatales
Gclavigera_Ophiostomatales
Cpulchella_Calosphaeriales
Cligniaria_Coniochaetales
Cinnumera_Chaetosphaeriales
Mtrigonosporus_Microascales
Cmaritima_Microascales
taxa
20
40
60
80
100
0
1
2
3
4
5
6
7
8
branch length

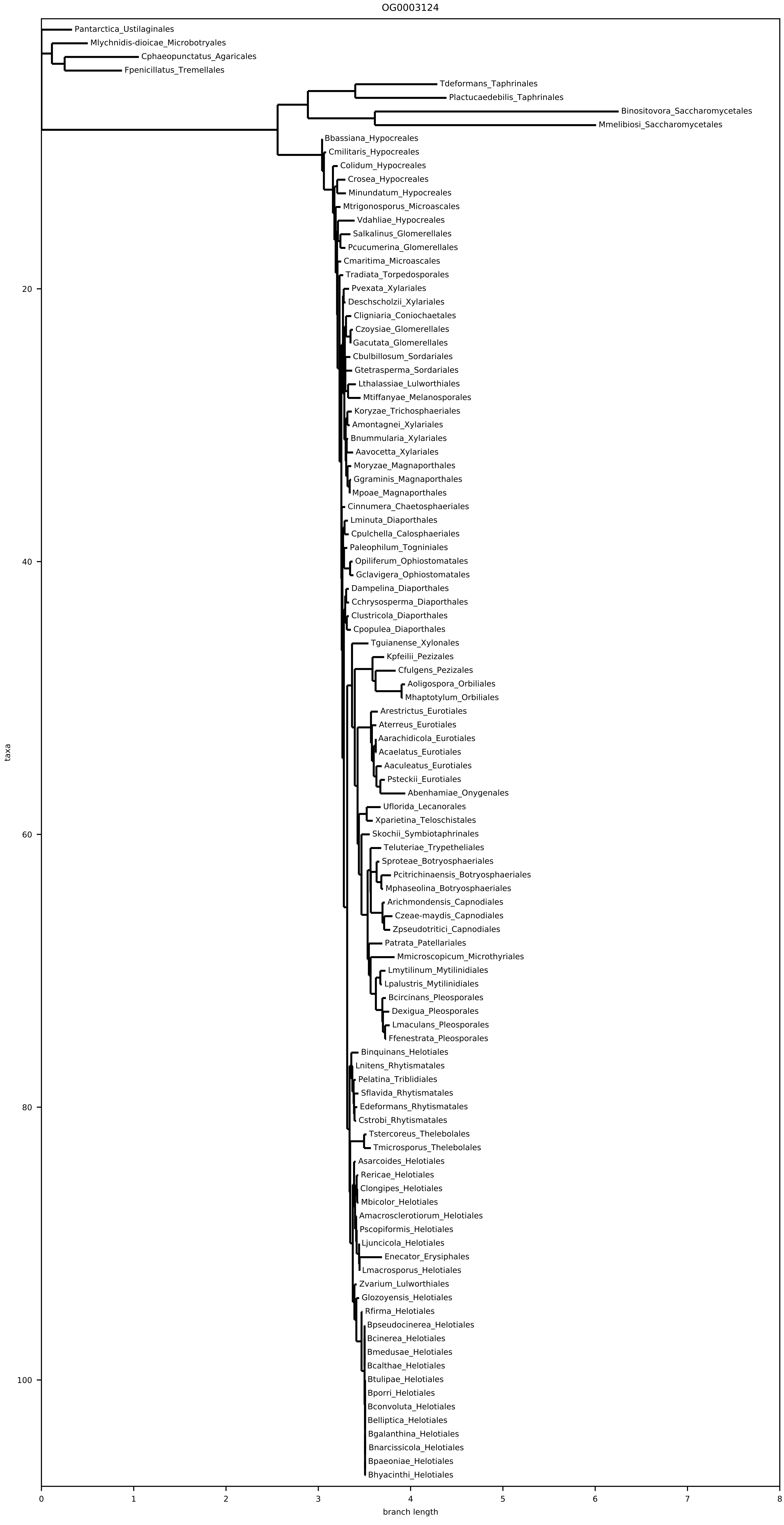
OG0003124
Pantarctica_Ustilaginales
Mlychnidis-dioicae_Microbotryales
Cphaeopunctatus_Agaricales
Fpenicillatus_Tremellales
Tdeformans_Taphrinales
Plactucaedebilis_Taphrinales
Binositovora_Saccharomycetales
Mmelibiosi_Saccharomycetales
Bbassiana_Hypocreales
Cmilitaris_Hypocreales
Colidum_Hypocreales
Crosea_Hypocreales
Minundatum_Hypocreales
Mtrigonosporus_Microascales
Vdahliae_Hypocreales
Salkalinus_Glomerellales
Pcucumerina_Glomerellales
Cmaritima_Microascales
Tradiata_Torpedosporales
Pvexata_Xylariales
Deschscholzii_Xylariales
Cligniaria_Coniochaetales
Czoysiae_Glomerellales
Gacutata_Glomerellales
Cbulbillosum_Sordariales
Gtetrasperma_Sordariales
Lthalassiae_Lulworthiales
Mtiffanyae_Melanosporales
Koryzae_Trichosphaeriales
Amontagnei_Xylariales
Bnummularia_Xylariales
Aavocetta_Xylariales
Moryzae_Magnaporthales
Ggraminis_Magnaporthales
Mpoae_Magnaporthales
Cinnumera_Chaetosphaeriales
Lminuta_Diaporthales
Cpulchella_Calosphaeriales
Paleophilum_Togniniales
Opiliferum_Ophiostomatales
Gclavigera_Ophiostomatales
Dampelina_Diaporthales
Cchrysosperma_Diaporthales
Clustricola_Diaporthales
Cpopulea_Diaporthales
Tguianense_Xylonales
Kpfeilii_Pezizales
Cfulgens_Pezizales
Aoligospora_Orbiliales
Mhaptotylum_Orbiliales
Arestrictus_Eurotiales
Aterreus_Eurotiales
Aarachidicola_Eurotiales
Acaelatus_Eurotiales
Aaculeatus_Eurotiales
Psteckii_Eurotiales
Abenhamiae_Onygenales
Uflorida_Lecanorales
Xparietina_Teloschistales
Skochii_Symbiotaphrinales
Teluteriae_Trypetheliales
Sproteae_Botryosphaeriales
Pcitrichinaensis_Botryosphaeriales
Mphaseolina_Botryosphaeriales
Arichmondensis_Capnodiales
Czeae-maydis_Capnodiales
Zpseudotritici_Capnodiales
Patrata_Patellariales
Mmicroscopicum_Microthyriales
Lmytilinum_Mytilinidiales
Lpalustris_Mytilinidiales
Bcircinans_Pleosporales
Dexigua_Pleosporales
Lmaculans_Pleosporales
Ffenestrata_Pleosporales
Binquinans_Helotiales
Lnitens_Rhytismatales
Pelatina_Triblidiales
Sflavida_Rhytismatales
Edeformans_Rhytismatales
Cstrobi_Rhytismatales
Tstercoreus_Thelebolales
Tmicrosporus_Thelebolales
Asarcoides_Helotiales
Rericae_Helotiales
Clongipes_Helotiales
Mbicolor_Helotiales
Amacrosclerotiorum_Helotiales
Pscopiformis_Helotiales
Ljuncicola_Helotiales
Enecator_Erysiphales
Lmacrosporus_Helotiales
Zvarium_Lulworthiales
Glozoyensis_Helotiales
Rfirma_Helotiales
Bpseudocinerea_Helotiales
Bcinerea_Helotiales
Bmedusae_Helotiales
Bcalthae_Helotiales
Btulipae_Helotiales
Bporri_Helotiales
Bconvoluta_Helotiales
Belliptica_Helotiales
Bgalanthina_Helotiales
Bnarcissicola_Helotiales
Bpaeoniae_Helotiales
Bhyacinthi_Helotiales
taxa
20
40
60
80
100
0
1
2
3
4
5
6
7
8
branch length

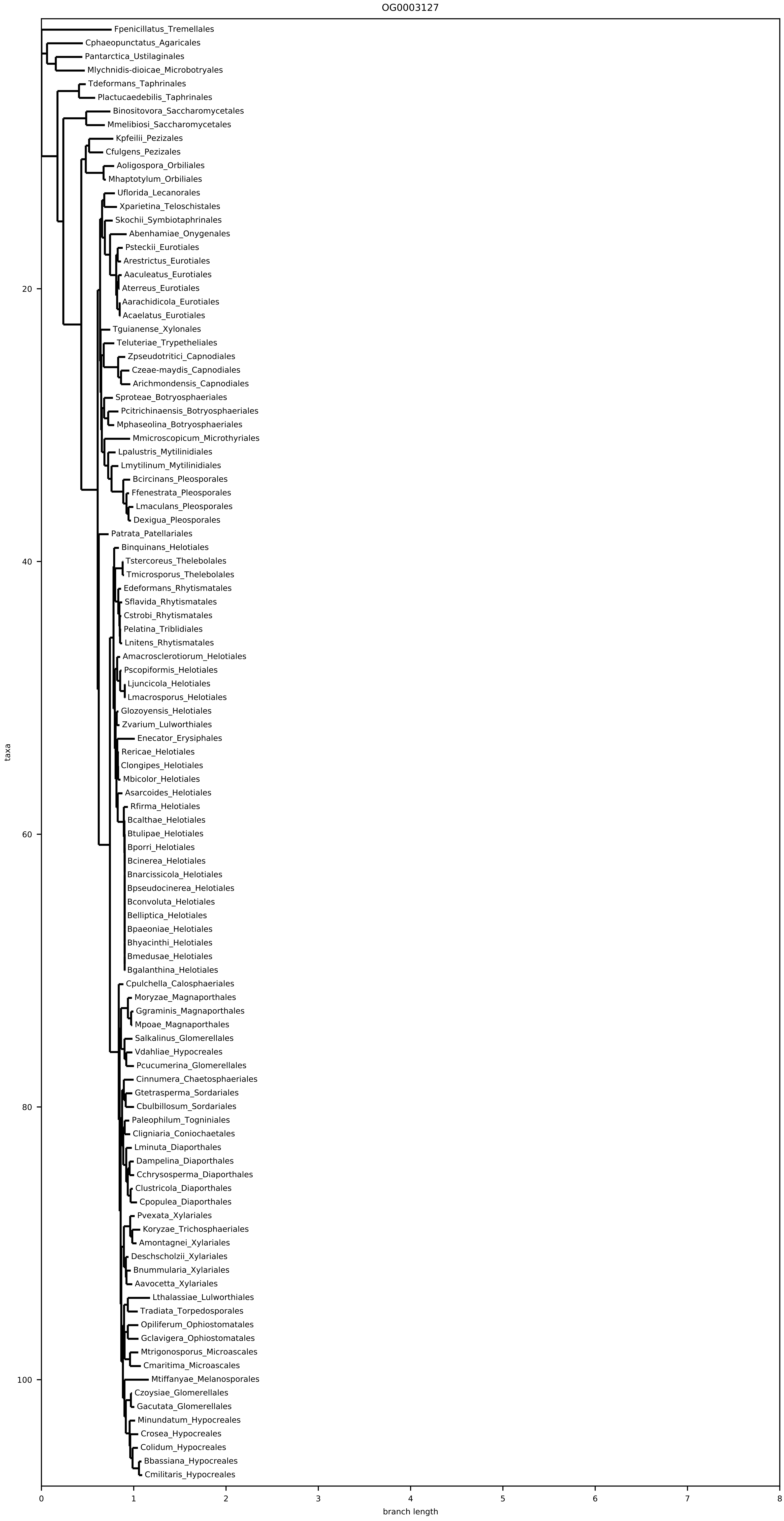

OG0003127
Fpenicillatus_Tremellales
Cphaeopunctatus_Agaricales
Pantarctica_Ustilaginales
Mlychnidis-dioicae_Microbotryales
Tdeformans_Taphrinales
Plactucaedebilis_Taphrinales
Binositovora_Saccharomycetales
Mmelibiosi_Saccharomycetales
Kpfeilii_Pezizales
Cfulgens_Pezizales
Aoligospora_Orbiliales
Mhaptotylum_Orbiliales
Uflorida_Lecanorales
Xparietina_Teloschistales
Skochii_Symbiotaphrinales
Abenhamiae_Onygenales
Psteckii_Eurotiales
Arestrictus_Eurotiales
Aaculeatus_Eurotiales
Aterreus_Eurotiales
Aarachidicola_Eurotiales
Acaelatus_Eurotiales
Tguianense_Xylonales
Teluteriae_Trypetheliales
Zpseudotritici_Capnodiales
Czeae-maydis_Capnodiales
Arichmondensis_Capnodiales
Sproteae_Botryosphaeriales
Pcitrichinaensis_Botryosphaeriales
Mphaseolina_Botryosphaeriales
Mmicroscopicum_Microthyriales
Lpalustris_Mytilinidiales
Lmytilinum_Mytilinidiales
Bcircinans_Pleosporales
Ffenestrata_Pleosporales
Lmaculans_Pleosporales
Dexigua_Pleosporales
Patrata_Patellariales
Binquinans_Helotiales
Tstercoreus_Thelebolales
Tmicrosporus_Thelebolales
Edeformans_Rhytismatales
Sflavida_Rhytismatales
Cstrobi_Rhytismatales
Pelatina_Triblidiales
Lnitens_Rhytismatales
Amacrosclerotiorum_Helotiales
Pscopiformis_Helotiales
Ljuncicola_Helotiales
Lmacrosporus_Helotiales
Glozoyensis_Helotiales
Zvarium_Lulworthiales
Enecator_Erysiphales
Rericae_Helotiales
Clongipes_Helotiales
Mbicolor_Helotiales
Asarcoides_Helotiales
Rfirma_Helotiales
Bcalthae_Helotiales
Btulipae_Helotiales
Bporri_Helotiales
Bcinerea_Helotiales
Bnarcissicola_Helotiales
Bpseudocinerea_Helotiales
Bconvoluta_Helotiales
Belliptica_Helotiales
Bpaeoniae_Helotiales
Bhyacinthi_Helotiales
Bmedusae_Helotiales
Bgalanthina_Helotiales
Cpulchella_Calosphaeriales
Moryzae_Magnaporthales
Ggraminis_Magnaporthales
Mpoae_Magnaporthales
Salkalinus_Glomerellales
Vdahliae_Hypocreales
Pcucumerina_Glomerellales
Cinnumera_Chaetosphaeriales
Gtetrasperma_Sordariales
Cbulbillosum_Sordariales
Paleophilum_Togniniales
Cligniaria_Coniochaetales
Lminuta_Diaporthales
Dampelina_Diaporthales
Cchrysosperma_Diaporthales
Clustricola_Diaporthales
Cpopulea_Diaporthales
Pvexata_Xylariales
Koryzae_Trichosphaeriales
Amontagnei_Xylariales
Deschscholzii_Xylariales
Bnummularia_Xylariales
Aavocetta_Xylariales
Lthalassiae_Lulworthiales
Tradiata_Torpedosporales
Opiliferum_Ophiostomatales
Gclavigera_Ophiostomatales
Mtrigonosporus_Microascales
Cmaritima_Microascales
Mtiffanyae_Melanosporales
Czoysiae_Glomerellales
Gacutata_Glomerellales
Minundatum_Hypocreales
Crosea_Hypocreales
Colidum_Hypocreales
Bbassiana_Hypocreales
Cmilitaris_Hypocreales
taxa
20
40
60
80
100
branch length
0
1
2
3
4
5
6
7
8

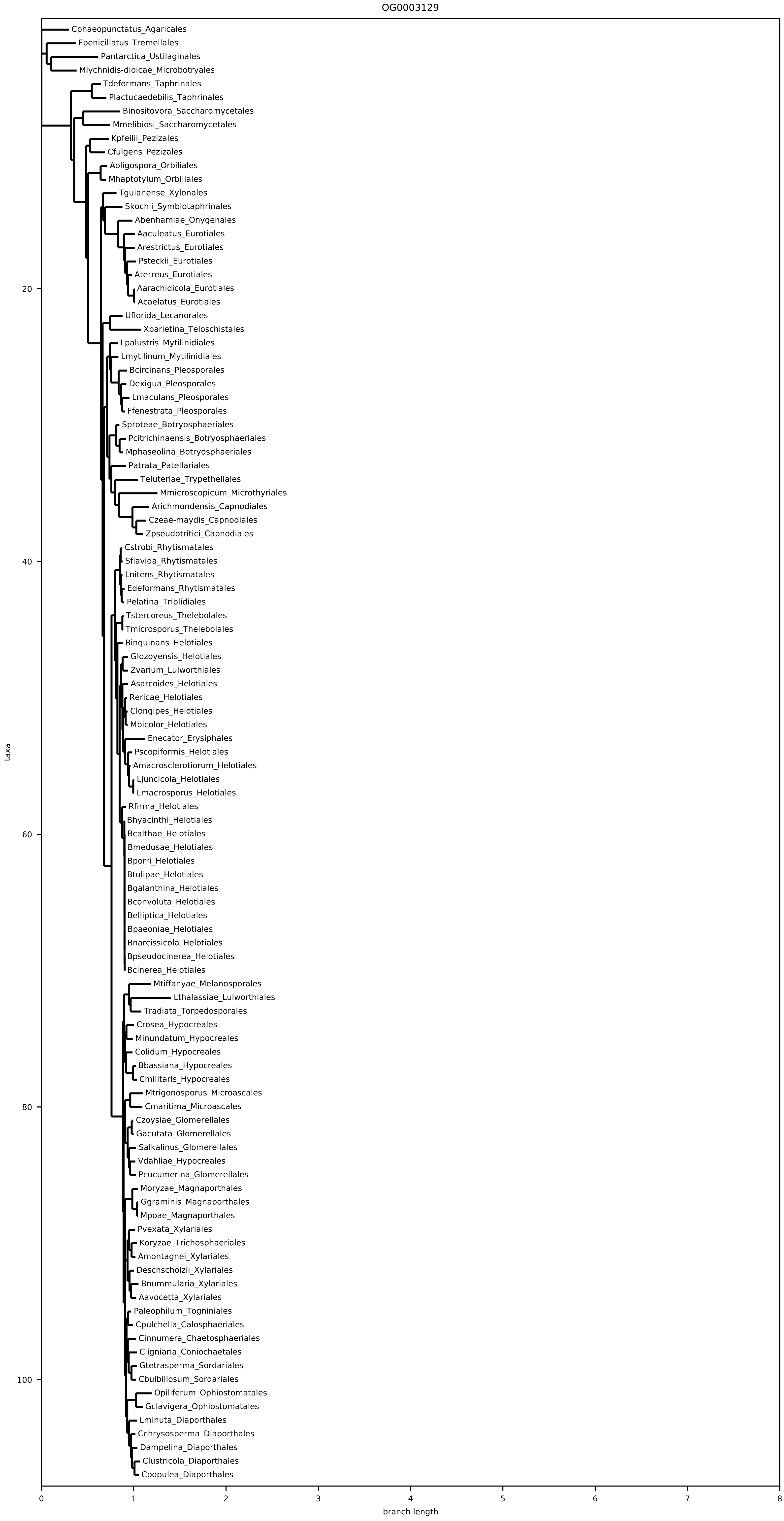
OG0003129
Cphaeopunctatus_Agaricales
Fpenicillatus_Tremellales
Pantarctica_Ustilaginales
Mlychnidis-dioicae_Microbotryales
Tdeformans_Taphrinales
Plactucaedebilis_Taphrinales
Binositovora_Saccharomycetales
Mmelibiosi_Saccharomycetales
Kpfeilii_Pezizales
Cfulgens_Pezizales
Aoligospora_Orbiliales
Mhaptotylum_Orbiliales
Tguianense_Xylonales
Skochii_Symbiotaphrinales
Abenhamiae_Onygenales
Aaculeatus_Eurotiales
Arestrictus_Eurotiales
Psteckii_Eurotiales
Aterreus_Eurotiales
Aarachidicola_Eurotiales
Acaelatus_Eurotiales
Uflorida_Lecanorales
Xparietina_Teloschistales
Lpalustris_Mytilinidiales
Lmytilinum_Mytilinidiales
Bcircinans_Pleosporales
Dexigua_Pleosporales
Lmaculans_Pleosporales
Ffenestrata_Pleosporales
Sproteae_Botryosphaeriales
Pcitrichinaensis_Botryosphaeriales
Mphaseolina_Botryosphaeriales
Patrata_Patellariales
Teluteriae_Trypetheliales
Mmicroscopicum_Microthyriales
Arichmondensis_Capnodiales
Czeae-maydis_Capnodiales
Zpseudotritici_Capnodiales
Cstrobi_Rhytismatales
Sflavida_Rhytismatales
Lnitens_Rhytismatales
Edeformans_Rhytismatales
Pelatina_Triblidiales
Tstercoreus_Thelebolales
Tmicrosporus_Thelebolales
Binquinans_Helotiales
Glozoyensis_Helotiales
Zvarium_Lulworthiales
Asarcoides_Helotiales
Rericae_Helotiales
Clongipes_Helotiales
Mbicolor_Helotiales
Enecator_Erysiphales
Pscopiformis_Helotiales
Amacrosclerotiorum_Helotiales
Ljuncicola_Helotiales
Lmacrosporus_Helotiales
Rfirma_Helotiales
Bhyacinthi_Helotiales
Bcalthae_Helotiales
Bmedusae_Helotiales
Bporri_Helotiales
Btulipae_Helotiales
Bgalanthina_Helotiales
Bconvoluta_Helotiales
Belliptica_Helotiales
Bpaeoniae_Helotiales
Bnarcissicola_Helotiales
Bpseudocinerea_Helotiales
Bcinerea_Helotiales
Mtiffanyae_Melanosporales
Lthalassiae_Lulworthiales
Tradiata_Torpedosporales
Crosea_Hypocreales
Minundatum_Hypocreales
Colidum_Hypocreales
Bbassiana_Hypocreales
Cmilitaris_Hypocreales
Mtrigonosporus_Microascales
Cmaritima_Microascales
Czoysiae_Glomerellales
Gacutata_Glomerellales
Salkalinus_Glomerellales
Vdahliae_Hypocreales
Pcucumerina_Glomerellales
Moryzae_Magnaporthales
Ggraminis_Magnaporthales
Mpoae_Magnaporthales
Pvexata_Xylariales
Koryzae_Trichosphaeriales
Amontagnei_Xylariales
Deschscholzii_Xylariales
Bnummularia_Xylariales
Aavocetta_Xylariales
Paleophilum_Togniniales
Cpulchella_Calosphaeriales
Cinnumera_Chaetosphaeriales
Cligniaria_Coniochaetales
Gtetrasperma_Sordariales
Cbulbillosum_Sordariales
Opiliferum_Ophiostomatales
Gclavigera_Ophiostomatales
Lminuta_Diaporthales
Cchrysosperma_Diaporthales
Dampelina_Diaporthales
Clustricola_Diaporthales
Cpopulea_Diaporthales
taxa
20
40
60
80
100
0
1
2
3
4
5
6
7
8
branch length

OG0003132

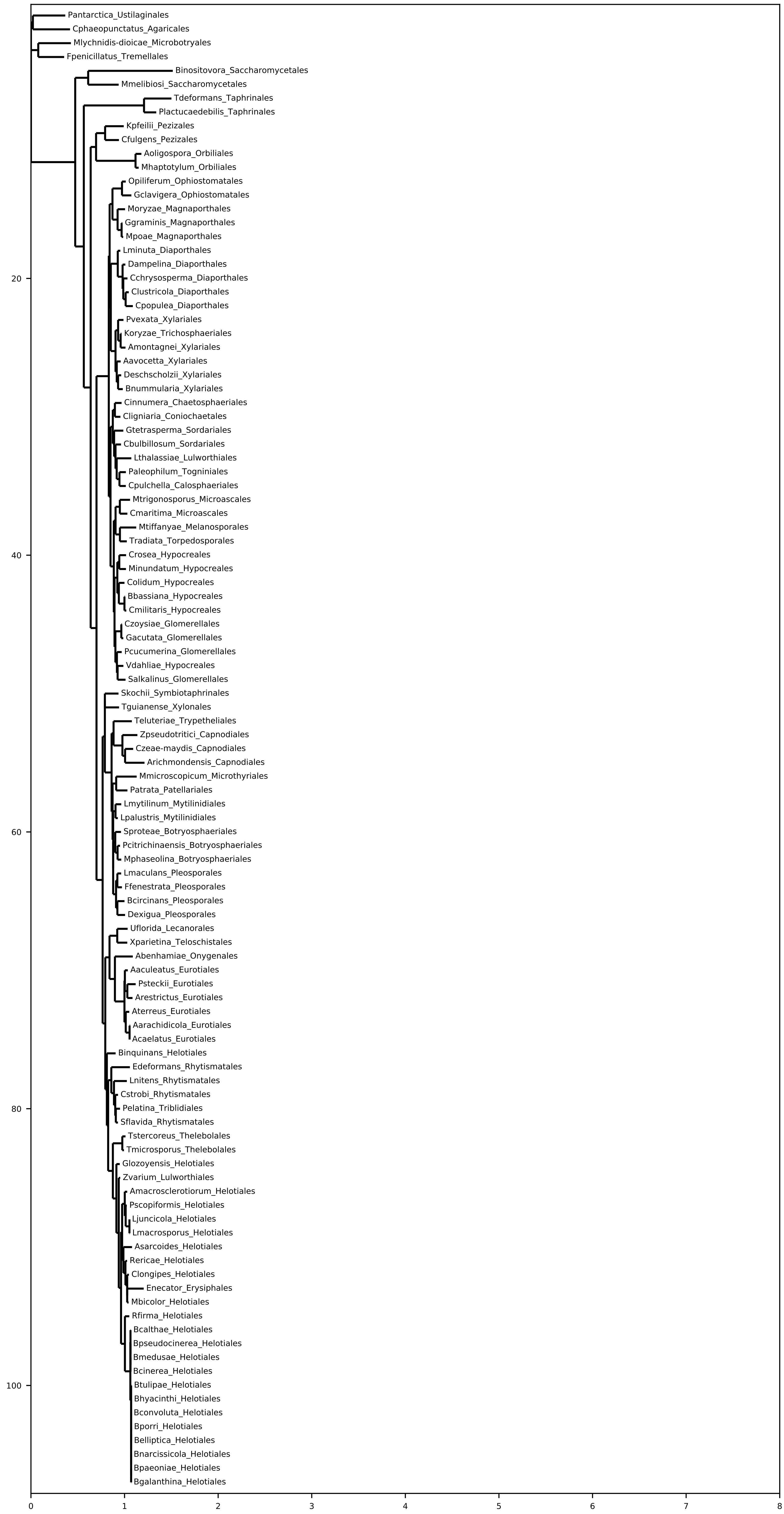

taxa

branch length

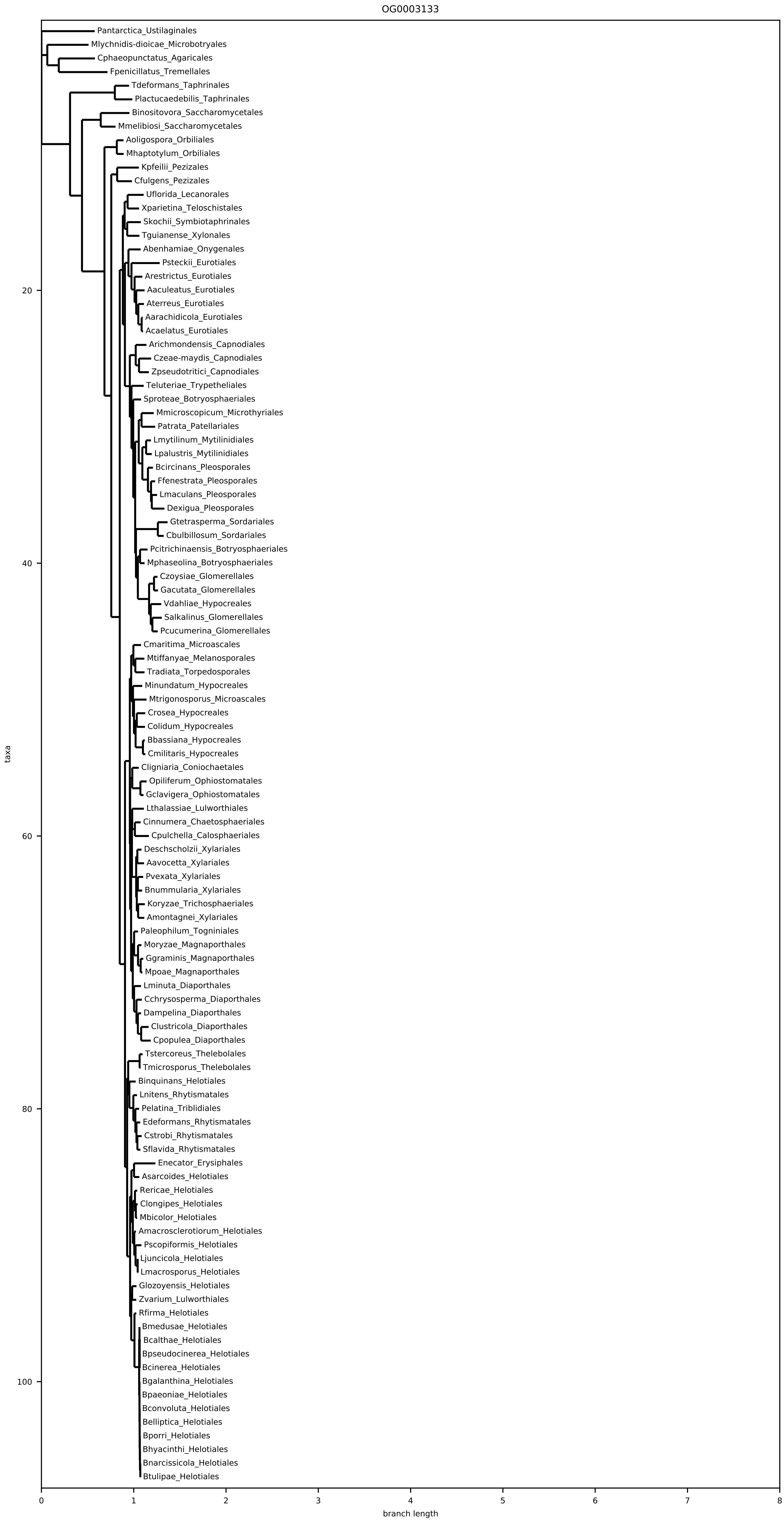
OG0003133
Pantarctica_Ustilaginales
Mlychnidis-dioicae_Microbotryales
Cphaeopunctatus_Agaricales
Fpenicillatus_Tremellales
Tdeformans_Taphrinales
Plactucaedebilis_Taphrinales
Binositovora_Saccharomycetales
Mmelibiosi_Saccharomycetales
Aoligospora_Orbiliales
Mhaptotylum_Orbiliales
Kpfeilii_Pezizales
Cfulgens_Pezizales
Uflorida_Lecanorales
Xparietina_Teloschistales
Skochii_Symbiotaphrinales
Tguianense_Xylonales
Abenhamiae_Onygenales
Psteckii_Eurotiales
Arestrictus_Eurotiales
Aaculeatus_Eurotiales
Aterreus_Eurotiales
Aarachidicola_Eurotiales
Acaelatus_Eurotiales
Arichmondensis_Capnodiales
Czeae-maydis_Capnodiales
Zpseudotritici_Capnodiales
Teluteriae_Trypetheliales
Sproteae_Botryosphaeriales
Mmicroscopicum_Microthyriales
Patrata_Patellariales
Lmytilinum_Mytilinidiales
Lpalustris_Mytilinidiales
Bcircinans_Pleosporales
Ffenestrata_Pleosporales
Lmaculans_Pleosporales
Dexigua_Pleosporales
Gtetrasperma_Sordariales
Cbulbillosum_Sordariales
Pcitrichinaensis_Botryosphaeriales
Mphaseolina_Botryosphaeriales
Czoysiae_Glomerellales
Gacutata_Glomerellales
Vdahliae_Hypocreales
Salkalinus_Glomerellales
Pcucumerina_Glomerellales
Cmaritima_Microascales
Mtiffanyae_Melanosporales
Tradiata_Torpedosporales
Minundatum_Hypocreales
Mtrigonosporus_Microascales
Crosea_Hypocreales
Colidum_Hypocreales
Bbassiana_Hypocreales
Cmilitaris_Hypocreales
Cligniaria_Coniochaetales
Opiliferum_Ophiostomatales
Gclavigera_Ophiostomatales
Lthalassiae_Lulworthiales
Cinnumera_Chaetosphaeriales
Cpulchella_Calosphaeriales
Deschscholzii_Xylariales
Aavocetta_Xylariales
Pvexata_Xylariales
Bnummularia_Xylariales
Koryzae_Trichosphaeriales
Amontagnei_Xylariales
Paleophilum_Togniniales
Moryzae_Magnaporthales
Ggraminis_Magnaporthales
Mpoae_Magnaporthales
Lminuta_Diaporthales
Cchrysosperma_Diaporthales
Dampelina_Diaporthales
Clustricola_Diaporthales
Cpopulea_Diaporthales
Tstercoreus_Thelebolales
Tmicrosporus_Thelebolales
Binquinans_Helotiales
Lnitens_Rhytismatales
Pelatina_Triblidiales
Edeformans_Rhytismatales
Cstrobi_Rhytismatales
Sflavida_Rhytismatales
Enecator_Erysiphales
Asarcoides_Helotiales
Rericae_Helotiales
Clongipes_Helotiales
Mbicolor_Helotiales
Amacrosclerotiorum_Helotiales
Pscopiformis_Helotiales
Ljuncicola_Helotiales
Lmacrosporus_Helotiales
Glozoyensis_Helotiales
Zvarium_Lulworthiales
Rfirma_Helotiales
Bmedusae_Helotiales
Bcalthae_Helotiales
Bpseudocinerea_Helotiales
Bcinerea_Helotiales
Bgalanthina_Helotiales
Bpaeoniae_Helotiales
Bconvoluta_Helotiales
Belliptica_Helotiales
Bporri_Helotiales
Bhyacinthi_Helotiales
Bnarcissicola_Helotiales
Btulipae_Helotiales
taxa
20
40
60
80
100
0
1
2
3
4
5
6
7
8
branch length

OG0003142

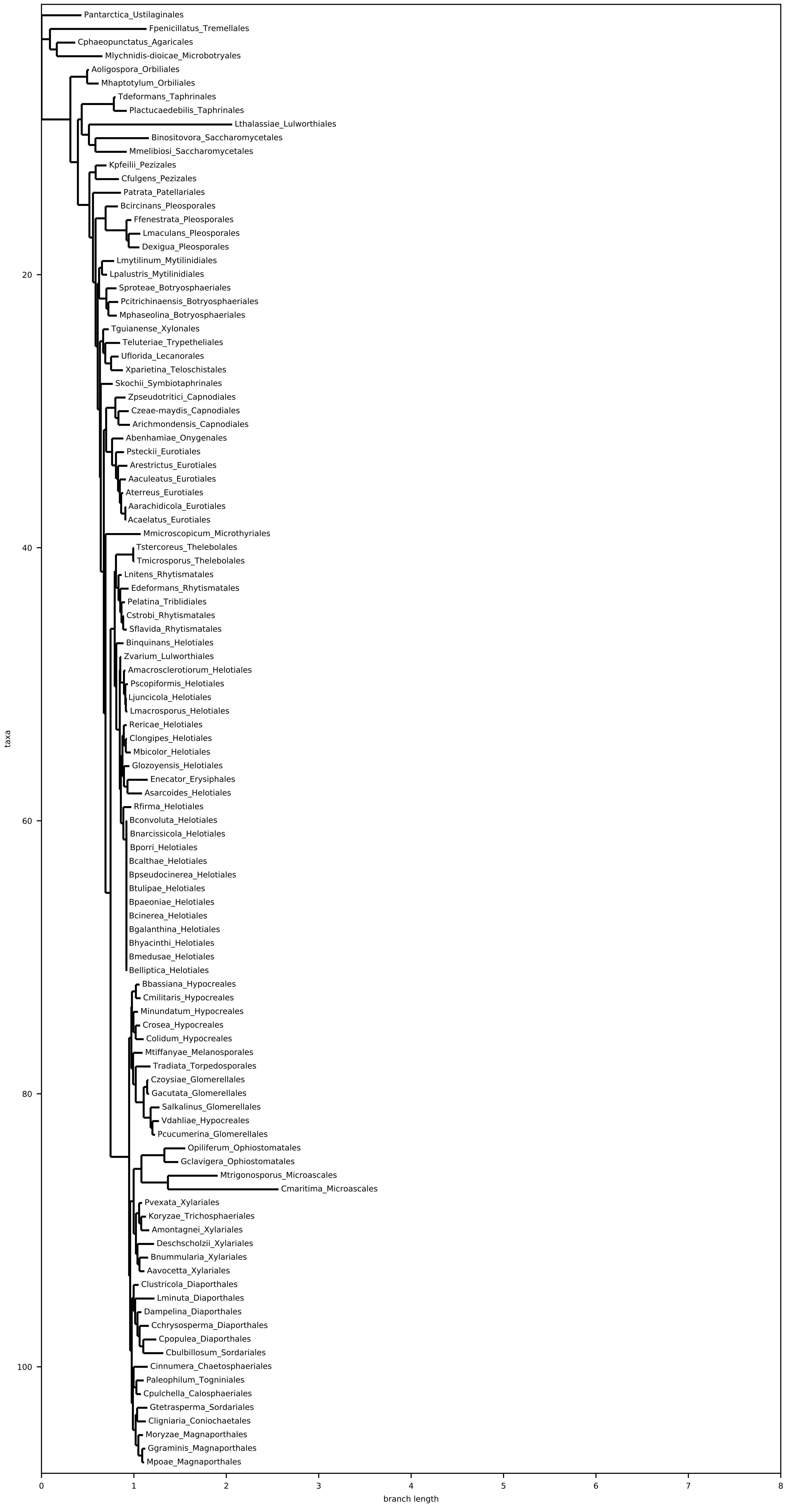

Pantarctica_Ustilaginales
Fpenicillatus_Tremellales
Cphaeopunctatus_Agaricales
Mlychnidis-dioicae_Microbotryales
Aoligospora_Orbiliales
Mhaptotylum_Orbiliales
Tdeformans_Taphrinales
Plactucaedebilis_Taphrinales
Lthalassiae_Lulworthiales
Binositovora_Saccharomycetales
Mmelibiosi_Saccharomycetales
Kpfeilii_Pezizales
Cfulgens_Pezizales
Patrata_Patellariales
Bcircinans_Pleosporales
Ffenestrata_Pleosporales
Lmaculans_Pleosporales
Dexigua_Pleosporales
Lmytilinum_Mytilinidiales
Lpalustris_Mytilinidiales
Sproteae_Botryosphaeriales
Pcitrichinaensis_Botryosphaeriales
Mphaseolina_Botryosphaeriales
Tguianense_Xylonales
Teluteriae_Trypetheliales
Uflorida_Lecanorales
Xparietina_Teloschistales
Skochii_Symbiotaphrinales
Zpseudotritici_Capnodiales
Czeae-maydis_Capnodiales
Arichmondensis_Capnodiales
Abenhamiae_Onygenales
Psteckii_Eurotiales
Arestrictus_Eurotiales
Aaculeatus_Eurotiales
Aterreus_Eurotiales
Aarachidicola_Eurotiales
Acaelatus_Eurotiales
Mmicroscopicum_Microthyriales
Tstercoreus_Thelebolales
Tmicrosporus_Thelebolales
Lnitens_Rhytismatales
Edeformans_Rhytismatales
Pelatina_Triblidiales
Cstrobi_Rhytismatales
Sflavida_Rhytismatales
Binquinans_Helotiales
Zvarium_Lulworthiales
Amacrosclerotiorum_Helotiales
Pscopiformis_Helotiales
Ljuncicola_Helotiales
Lmacrosporus_Helotiales
Rericae_Helotiales
Clongipes_Helotiales
Mbicolor_Helotiales
Glozoyensis_Helotiales
Enecator_Erysiphales
Asarcoides_Helotiales
Rfirma_Helotiales
Bconvoluta_Helotiales
Bnarcissicola_Helotiales
Bporri_Helotiales
Bcalthae_Helotiales
Bpseudocinerea_Helotiales
Btulipae_Helotiales
Bpaeoniae_Helotiales
Bcinerea_Helotiales
Bgalanthina_Helotiales
Bhyacinthi_Helotiales
Bmedusae_Helotiales
Belliptica_Helotiales
Bbassiana_Hypocreales
Cmilitaris_Hypocreales
Minundatum_Hypocreales
Crosea_Hypocreales
Colidum_Hypocreales
Mtiffanyae_Melanosporales
Tradiata_Torpedosporales
Czoysiae_Glomerellales
Gacutata_Glomerellales
Salkalinus_Glomerellales
Vdahliae_Hypocreales
Pcucumerina_Glomerellales
Opiliferum_Ophiostomatales
Gclavigera_Ophiostomatales
Mtrigonosporus_Microascales
Cmaritima_Microascales
Pvexata_Xylariales
Koryzae_Trichosphaeriales
Amontagnei_Xylariales
Deschscholzii_Xylariales
Bnummularia_Xylariales
Aavocetta_Xylariales
Clustricola_Diaporthales
Lminuta_Diaporthales
Dampelina_Diaporthales
Cchrysosperma_Diaporthales
Cpopulea_Diaporthales
Cbulbillosum_Sordariales
Cinnumera_Chaetosphaeriales
Paleophilum_Togniniales
Cpulchella_Calosphaeriales
Gtetrasperma_Sordariales
Cligniaria_Coniochaetales
Moryzae_Magnaporthales
Ggraminis_Magnaporthales
Mpoae_Magnaporthales
20
40
60
80
100
taxa
0
1
2
3
4
5
6
7
8
branch length

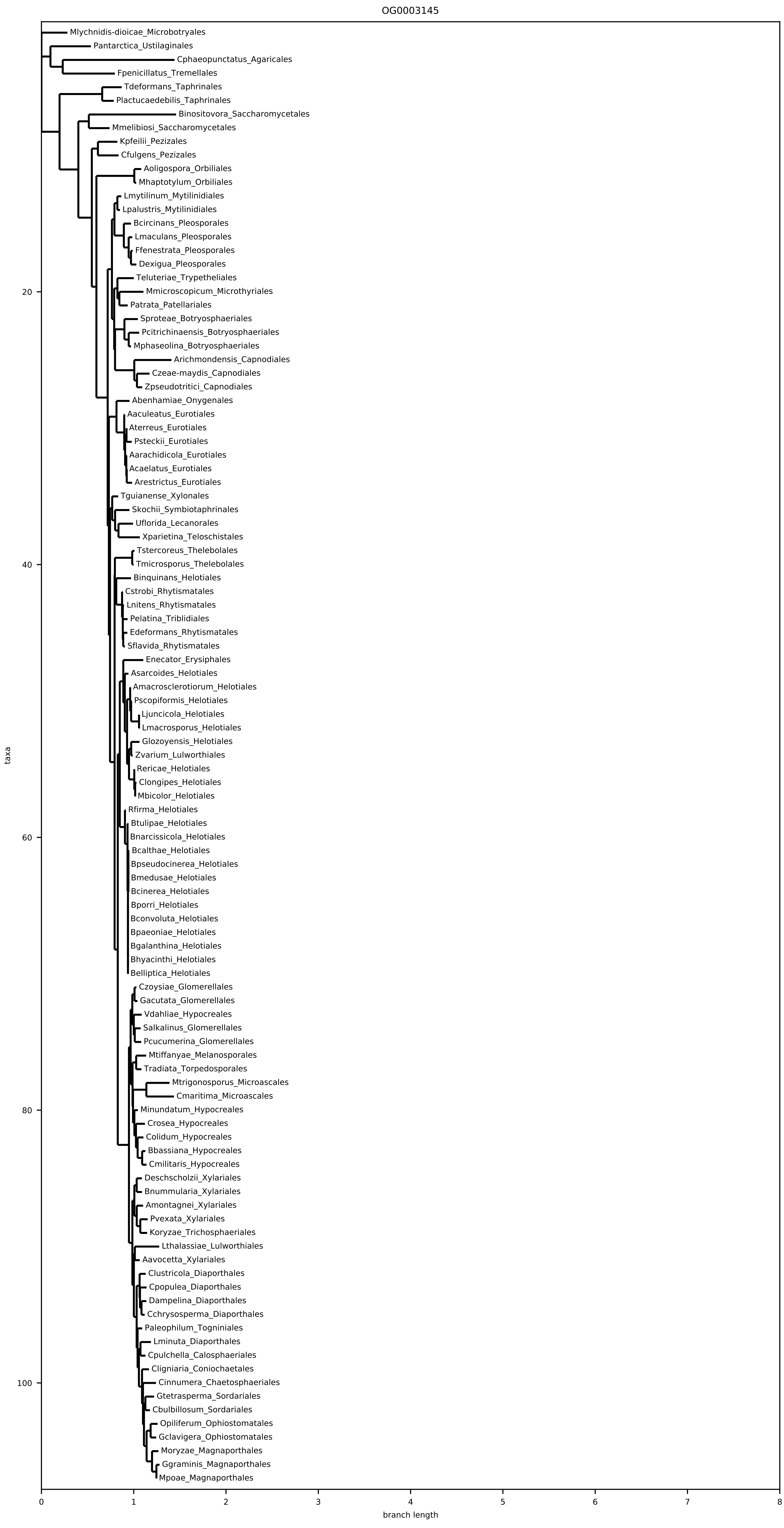

OG0003145
Mlychnidis-dioicae_Microbotryales
Pantarctica_Ustilaginales
Cphaeopunctatus_Agaricales
Fpenicillatus_Tremellales
Tdeformans_Taphrinales
Plactucaedebilis_Taphrinales
Binositovora_Saccharomycetales
Mmelibiosi_Saccharomycetales
Kpfeilii_Pezizales
Cfulgens_Pezizales
Aoligospora_Orbiliales
Mhaptotylum_Orbiliales
Lmytilinum_Mytilinidiales
Lpalustris_Mytilinidiales
Bcircinans_Pleosporales
Lmaculans_Pleosporales
Ffenestrata_Pleosporales
Dexigua_Pleosporales
Teluteriae_Trypetheliales
Mmicroscopicum_Microthyriales
Patrata_Patellariales
Sproteae_Botryosphaeriales
Pcitrichinaensis_Botryosphaeriales
Mphaseolina_Botryosphaeriales
Arichmondensis_Capnodiales
Czeae-maydis_Capnodiales
Zpseudotritici_Capnodiales
Abenhamiae_Onygenales
Aaculeatus_Eurotiales
Aterreus_Eurotiales
Psteckii_Eurotiales
Aarachidicola_Eurotiales
Acaelatus_Eurotiales
Arestrictus_Eurotiales
Tguianense_Xylonales
Skochii_Symbiotaphrinales
Uflorida_Lecanorales
Xparietina_Teloschistales
Tstercoreus_Thelebolales
Tmicrosporus_Thelebolales
Binquinans_Helotiales
Cstrobi_Rhytismatales
Lnitens_Rhytismatales
Pelatina_Triblidiales
Edeformans_Rhytismatales
Sflavida_Rhytismatales
Enecator_Erysiphales
Asarcoides_Helotiales
Amacrosclerotiorum_Helotiales
Pscopiformis_Helotiales
Ljuncicola_Helotiales
Lmacrosporus_Helotiales
Glozoyensis_Helotiales
Zvarium_Lulworthiales
Rericae_Helotiales
Clongipes_Helotiales
Mbicolor_Helotiales
Rfirma_Helotiales
Btulipae_Helotiales
Bnarcissicola_Helotiales
Bcalthae_Helotiales
Bpseudocinerea_Helotiales
Bmedusae_Helotiales
Bcinerea_Helotiales
Bporri_Helotiales
Bconvoluta_Helotiales
Bpaeoniae_Helotiales
Bgalanthina_Helotiales
Bhyacinthi_Helotiales
Belliptica_Helotiales
Czoysiae_Glomerellales
Gacutata_Glomerellales
Vdahliae_Hypocreales
Salkalinus_Glomerellales
Pcucumerina_Glomerellales
Mtiffanyae_Melanosporales
Tradiata_Torpedosporales
Mtrigonosporus_Microascales
Cmaritima_Microascales
Minundatum_Hypocreales
Crosea_Hypocreales
Colidum_Hypocreales
Bbassiana_Hypocreales
Cmilitaris_Hypocreales
Deschscholzii_Xylariales
Bnummularia_Xylariales
Amontagnei_Xylariales
Pvexata_Xylariales
Koryzae_Trichosphaeriales
Lthalassiae_Lulworthiales
Aavocetta_Xylariales
Clustricola_Diaporthales
Cpopulea_Diaporthales
Dampelina_Diaporthales
Cchrysosperma_Diaporthales
Paleophilum_Togniniales
Lminuta_Diaporthales
Cpulchella_Calosphaeriales
Cligniaria_Coniochaetales
Cinnumera_Chaetosphaeriales
Gtetrasperma_Sordariales
Cbulbillosum_Sordariales
Opiliferum_Ophiostomatales
Gclavigera_Ophiostomatales
Moryzae_Magnaporthales
Ggraminis_Magnaporthales
Mpoae_Magnaporthales
taxa
20
40
60
80
100
0
1
2
3
4
5
6
7
8
branch length

OG0003146

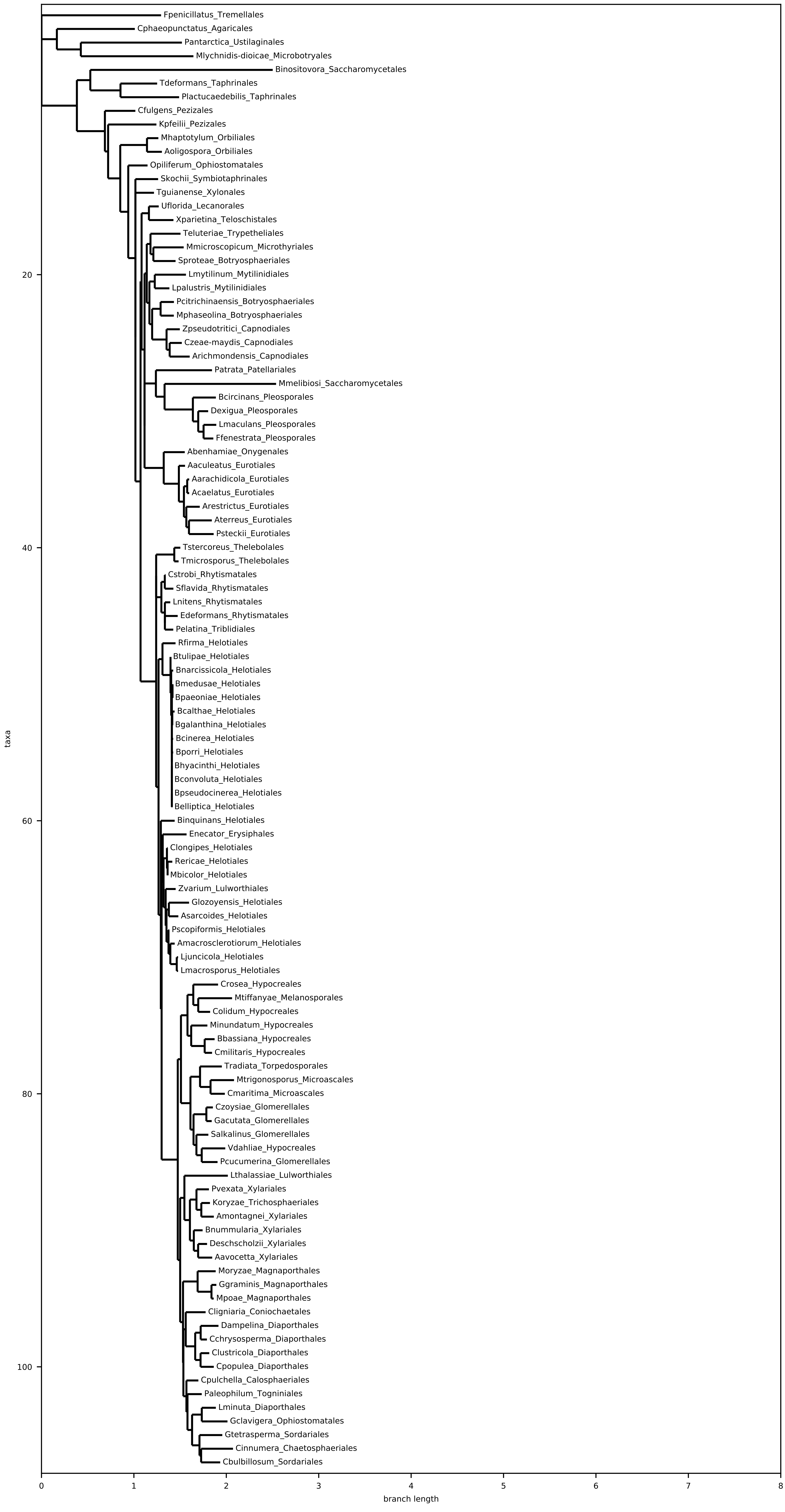

Fpenicillatus_Tremellales
Cphaeopunctatus_Agaricales
Pantarctica_Ustilaginales
Mlychnidis-dioicae_Microbotryales
Binositovora_Saccharomycetales
Tdeformans_Taphrinales
Plactucaedebilis_Taphrinales
Cfulgens_Pezizales
Kpfeilii_Pezizales
Mhaptotylum_Orbiliales
Aoligospora_Orbiliales
Opiliferum_Ophiostomatales
Skochii_Symbiotaphrinales
Tguianense_Xylonales
Uflorida_Lecanorales
Xparietina_Teloschistales
Teluteriae_Trypetheliales
Mmicroscopicum_Microthyriales
Sproteae_Botryosphaeriales
Lmytilinum_Mytilinidiales
Lpalustris_Mytilinidiales
Pcitrichinaensis_Botryosphaeriales
Mphaseolina_Botryosphaeriales
Zpseudotritici_Capnodiales
Czeae-maydis_Capnodiales
Arichmondensis_Capnodiales
Patrata_Patellariales
Mmelibiosi_Saccharomycetales
Bcircinans_Pleosporales
Dexigua_Pleosporales
Lmaculans_Pleosporales
Ffenestrata_Pleosporales
Abenhamiae_Onygenales
Aaculeatus_Eurotiales
Aarachidicola_Eurotiales
Acaelatus_Eurotiales
Arestrictus_Eurotiales
Aterreus_Eurotiales
Psteckii_Eurotiales
Tstercoreus_Thelebolales
Tmicrosporus_Thelebolales
Cstrobi_Rhytismatales
Sflavida_Rhytismatales
Lnitens_Rhytismatales
Edeformans_Rhytismatales
Pelatina_Triblidiales
Rfirma_Helotiales
Btulipae_Helotiales
Bnarcissicola_Helotiales
Bmedusae_Helotiales
Bpaeoniae_Helotiales
Bcalthae_Helotiales
Bgalanthina_Helotiales
Bcinerea_Helotiales
Bporri_Helotiales
Bhyacinthi_Helotiales
Bconvoluta_Helotiales
Bpseudocinerea_Helotiales
Belliptica_Helotiales
Binquinans_Helotiales
Enecator_Erysiphales
Clongipes_Helotiales
Rericae_Helotiales
Mbicolor_Helotiales
Zvarium_Lulworthiales
Glozoyensis_Helotiales
Asarcoides_Helotiales
Pscopiformis_Helotiales
Amacrosclerotiorum_Helotiales
Ljuncicola_Helotiales
Lmacrosporus_Helotiales
Crosea_Hypocreales
Mtiffanyae_Melanosporales
Colidum_Hypocreales
Minundatum_Hypocreales
Bbassiana_Hypocreales
Cmilitaris_Hypocreales
Tradiata_Torpedosporales
Mtrigonosporus_Microascales
Cmaritima_Microascales
Czoysiae_Glomerellales
Gacutata_Glomerellales
Salkalinus_Glomerellales
Vdahliae_Hypocreales
Pcucumerina_Glomerellales
Lthalassiae_Lulworthiales
Pvexata_Xylariales
Koryzae_Trichosphaeriales
Amontagnei_Xylariales
Bnummularia_Xylariales
Deschscholzii_Xylariales
Aavocetta_Xylariales
Moryzae_Magnaporthales
Ggraminis_Magnaporthales
Mpoae_Magnaporthales
Cligniaria_Coniochaetales
Dampelina_Diaporthales
Cchrysosperma_Diaporthales
Clustricola_Diaporthales
Cpopulea_Diaporthales
Cpulchella_Calosphaeriales
Paleophilum_Togniniales
Lminuta_Diaporthales
Gclavigera_Ophiostomatales
Gtetrasperma_Sordariales
Cinnumera_Chaetosphaeriales
Cbulbillosum_Sordariales
taxa
20
40
60
80
100
0
1
2
3
4
5
6
7
8
branch length

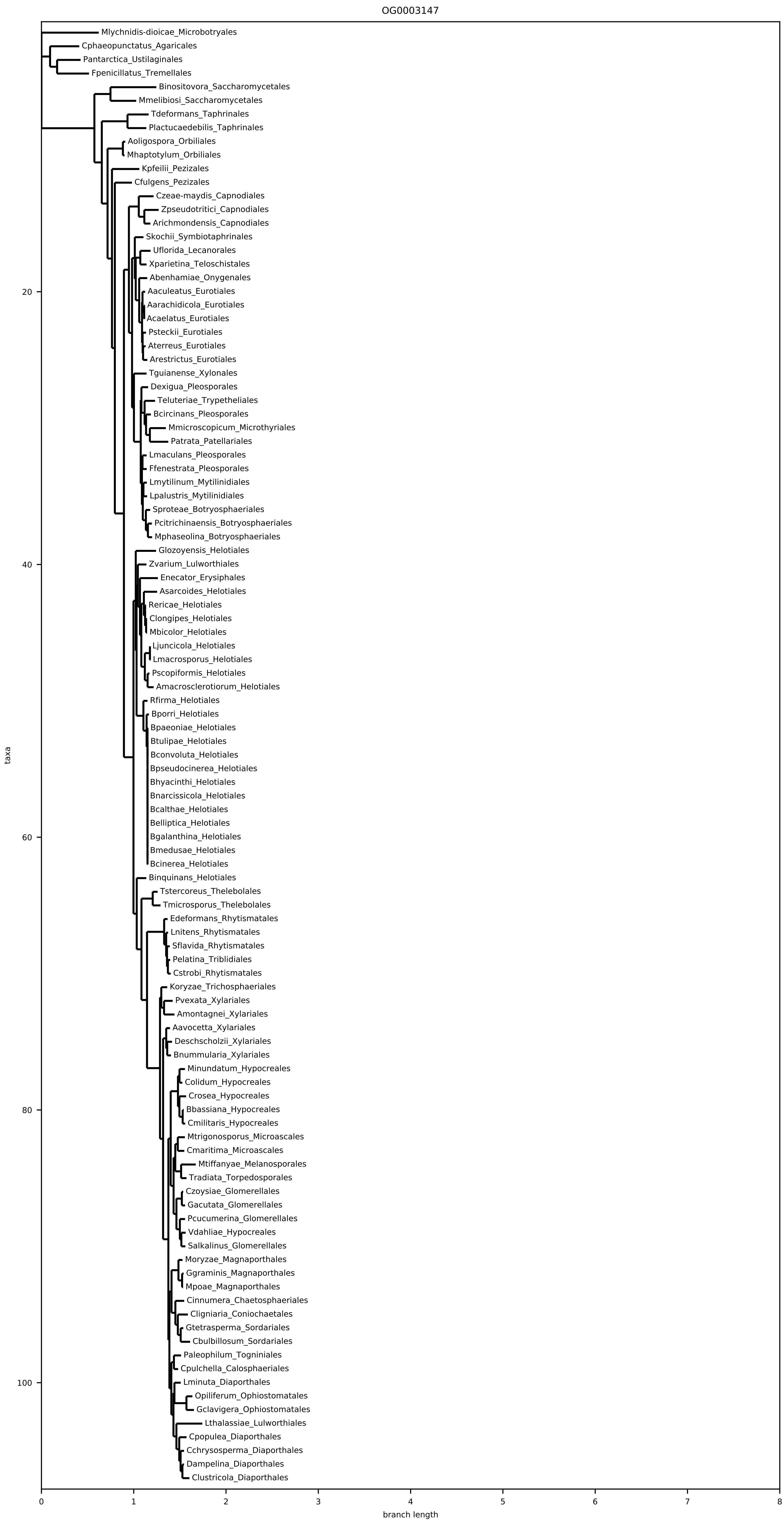
OG0003147
Mlychnidis-dioicae_Microbotryales
Cphaeopunctatus_Agaricales
Pantarctica_Ustilaginales
Fpenicillatus_Tremellales
Binositovora_Saccharomycetales
Mmelibiosi_Saccharomycetales
Tdeformans_Taphrinales
Plactucaedebilis_Taphrinales
Aoligospora_Orbiliales
Mhaptotylum_Orbiliales
Kpfeilii_Pezizales
Cfulgens_Pezizales
Czeae-maydis_Capnodiales
Zpseudotritici_Capnodiales
Arichmondensis_Capnodiales
Skochii_Symbiotaphrinales
Uflorida_Lecanorales
Xparietina_Teloschistales
Abenhamiae_Onygenales
Aaculeatus_Eurotiales
Aarachidicola_Eurotiales
Acaelatus_Eurotiales
Psteckii_Eurotiales
Aterreus_Eurotiales
Arestrictus_Eurotiales
Tguianense_Xylonales
Dexigua_Pleosporales
Teluteriae_Trypetheliales
Bcircinans_Pleosporales
Mmicroscopicum_Microthyriales
Patrata_Patellariales
Lmaculans_Pleosporales
Ffenestrata_Pleosporales
Lmytilinum_Mytilinidiales
Lpalustris_Mytilinidiales
Sproteae_Botryosphaeriales
Pcitrichinaensis_Botryosphaeriales
Mphaseolina_Botryosphaeriales
Glozoyensis_Helotiales
Zvarium_Lulworthiales
Enecator_Erysiphales
Asarcoides_Helotiales
Rericae_Helotiales
Clongipes_Helotiales
Mbicolor_Helotiales
Ljuncicola_Helotiales
Lmacrosporus_Helotiales
Pscopiformis_Helotiales
Amacrosclerotiorum_Helotiales
Rfirma_Helotiales
Bporri_Helotiales
Bpaeoniae_Helotiales
Btulipae_Helotiales
Bconvoluta_Helotiales
Bpseudocinerea_Helotiales
Bhyacinthi_Helotiales
Bnarcissicola_Helotiales
Bcalthae_Helotiales
Belliptica_Helotiales
Bgalanthina_Helotiales
Bmedusae_Helotiales
Bcinerea_Helotiales
Binquinans_Helotiales
Tstercoreus_Thelebolales
Tmicrosporus_Thelebolales
Edeformans_Rhytismatales
Lnitens_Rhytismatales
Sflavida_Rhytismatales
Pelatina_Triblidiales
Cstrobi_Rhytismatales
Koryzae_Trichosphaeriales
Pvexata_Xylariales
Amontagnei_Xylariales
Aavocetta_Xylariales
Deschscholzii_Xylariales
Bnummularia_Xylariales
Minundatum_Hypocreales
Colidum_Hypocreales
Crosea_Hypocreales
Bbassiana_Hypocreales
Cmilitaris_Hypocreales
Mtrigonosporus_Microascales
Cmaritima_Microascales
Mtiffanyae_Melanosporales
Tradiata_Torpedosporales
Czoysiae_Glomerellales
Gacutata_Glomerellales
Pcucumerina_Glomerellales
Vdahliae_Hypocreales
Salkalinus_Glomerellales
Moryzae_Magnaporthales
Ggraminis_Magnaporthales
Mpoae_Magnaporthales
Cinnumera_Chaetosphaeriales
Cligniaria_Coniochaetales
Gtetrasperma_Sordariales
Cbulbillosum_Sordariales
Paleophilum_Togniniales
Cpulchella_Calosphaeriales
Lminuta_Diaporthales
Opiliferum_Ophiostomatales
Gclavigera_Ophiostomatales
Lthalassiae_Lulworthiales
Cpopulea_Diaporthales
Cchrysosperma_Diaporthales
Dampelina_Diaporthales
Clustricola_Diaporthales
taxa
20
40
60
80
100
0
1
2
3
4
5
6
7
8
branch length

OG0003155

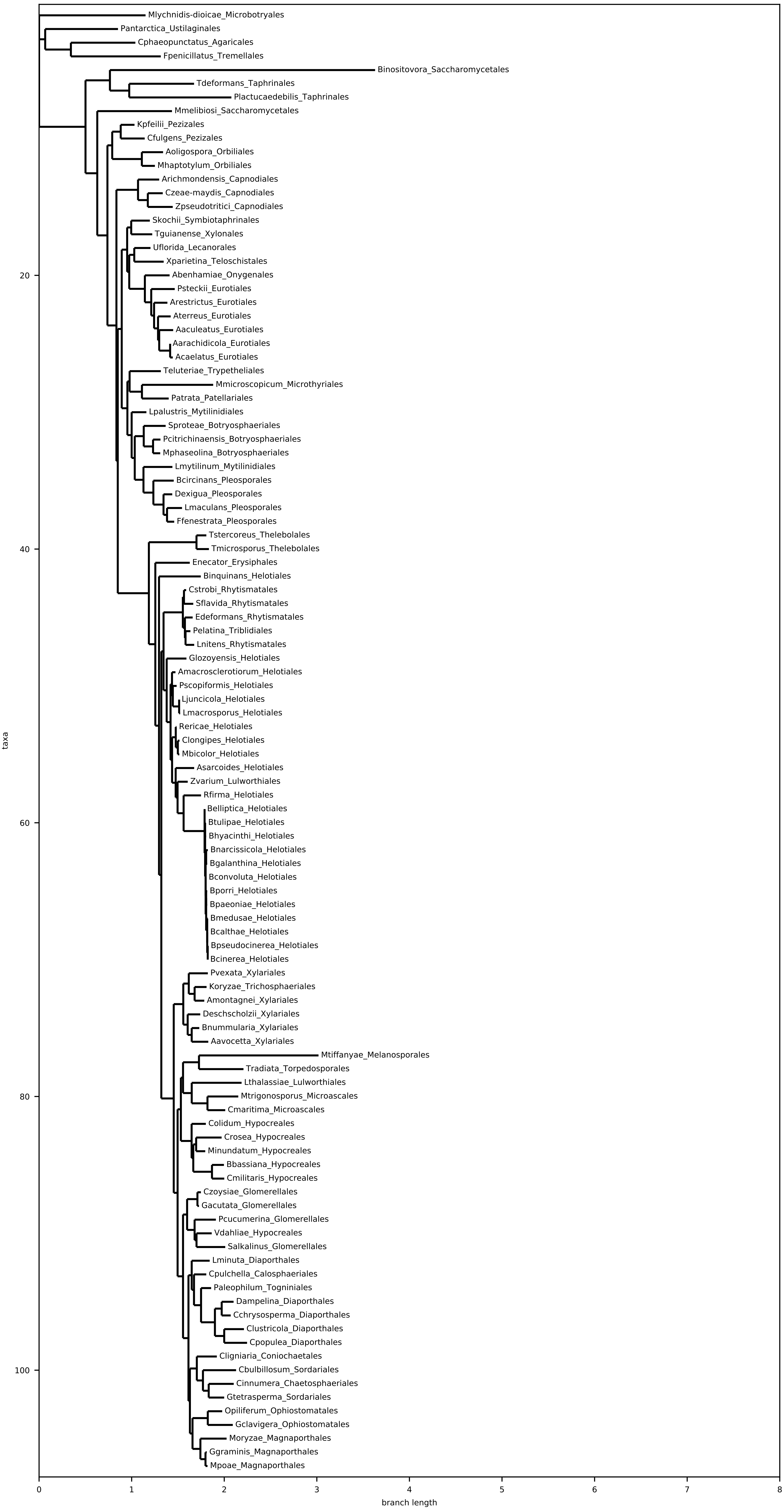

Mlychnidis-dioicae_Microbotryales
Pantarctica_Ustilaginales
Cphaeopunctatus_Agaricales
Fpenicillatus_Tremellales
Binositovora_Saccharomycetales
Tdeformans_Taphrinales
Plactucaedebilis_Taphrinales
Mmelibiosi_Saccharomycetales
Kpfeilii_Pezizales
Cfulgens_Pezizales
Aoligospora_Orbiliales
Mhaptotylum_Orbiliales
Arichmondensis_Capnodiales
Czeae-maydis_Capnodiales
Zpseudotritici_Capnodiales
Skochii_Symbiotaphrinales
Tguianense_Xylonales
Uflorida_Lecanorales
Xparietina_Teloschistales
Abenhamiae_Onygenales
Psteckii_Eurotiales
Arestrictus_Eurotiales
Aterreus_Eurotiales
Aaculeatus_Eurotiales
Aarachidicola_Eurotiales
Acaelatus_Eurotiales
Teluteriae_Trypetheliales
Mmicroscopicum_Microthyriales
Patrata_Patellariales
Lpalustris_Mytilinidiales
Sproteae_Botryosphaeriales
Pcitrichinaensis_Botryosphaeriales
Mphaseolina_Botryosphaeriales
Lmytilinum_Mytilinidiales
Bcircinans_Pleosporales
Dexigua_Pleosporales
Lmaculans_Pleosporales
Ffenestrata_Pleosporales
Tstercoreus_Thelebolales
Tmicrosporus_Thelebolales
Enecator_Erysiphales
Binquinans_Helotiales
Cstrobi_Rhytismatales
Sflavida_Rhytismatales
Edeformans_Rhytismatales
Pelatina_Triblidiales
Lnitens_Rhytismatales
Glozoyensis_Helotiales
Amacrosclerotiorum_Helotiales
Pscopiformis_Helotiales
Ljuncicola_Helotiales
Lmacrosporus_Helotiales
Rericae_Helotiales
Clongipes_Helotiales
Mbicolor_Helotiales
Asarcoides_Helotiales
Zvarium_Lulworthiales
Rfirma_Helotiales
Belliptica_Helotiales
Btulipae_Helotiales
Bhyacinthi_Helotiales
Bnarcissicola_Helotiales
Bgalanthina_Helotiales
Bconvoluta_Helotiales
Bporri_Helotiales
Bpaeoniae_Helotiales
Bmedusae_Helotiales
Bcalthae_Helotiales
Bpseudocinerea_Helotiales
Bcinerea_Helotiales
Pvexata_Xylariales
Koryzae_Trichosphaeriales
Amontagnei_Xylariales
Deschscholzii_Xylariales
Bnummularia_Xylariales
Aavocetta_Xylariales
Mtiffanyae_Melanosporales
Tradiata_Torpedosporales
Lthalassiae_Lulworthiales
Mtrigonosporus_Microascales
Cmaritima_Microascales
Colidum_Hypocreales
Crosea_Hypocreales
Minundatum_Hypocreales
Bbassiana_Hypocreales
Cmilitaris_Hypocreales
Czoysiae_Glomerellales
Gacutata_Glomerellales
Pcucumerina_Glomerellales
Vdahliae_Hypocreales
Salkalinus_Glomerellales
Lminuta_Diaporthales
Cpulchella_Calosphaeriales
Paleophilum_Togniniales
Dampelina_Diaporthales
Cchrysosperma_Diaporthales
Clustricola_Diaporthales
Cpopulea_Diaporthales
Cligniaria_Coniochaetales
Cbulbillosum_Sordariales
Cinnumera_Chaetosphaeriales
Gtetrasperma_Sordariales
Opiliferum_Ophiostomatales
Gclavigera_Ophiostomatales
Moryzae_Magnaporthales
Ggraminis_Magnaporthales
Mpoae_Magnaporthales
taxa
20
40
60
80
100
0
1
2
3
4
5
6
7
8
branch length

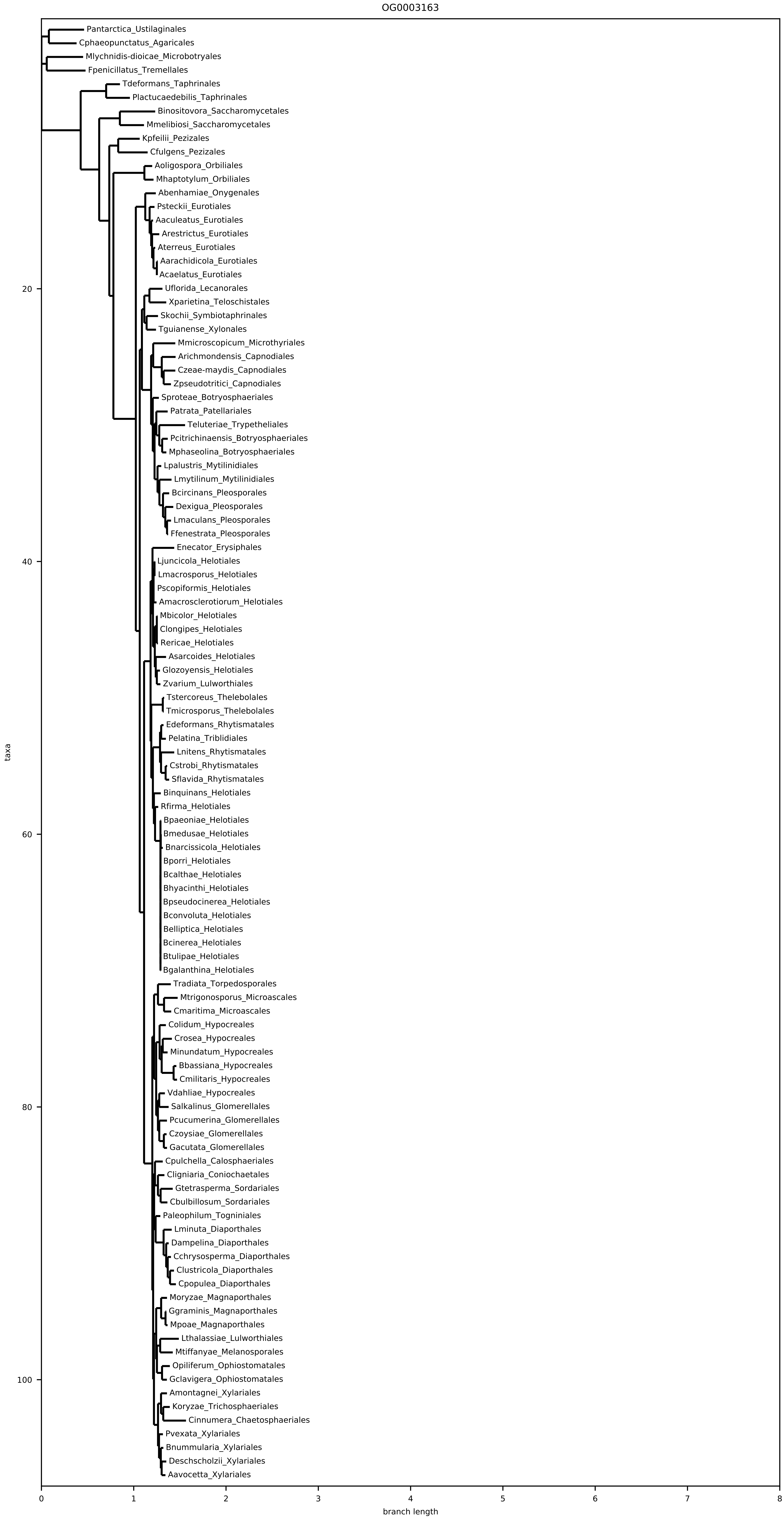
OG0003163
Pantarctica_Ustilaginales
Cphaeopunctatus_Agaricales
Mlychnidis-dioicae_Microbotryales
Fpenicillatus_Tremellales
Tdeformans_Taphrinales
Plactucaedebilis_Taphrinales
Binositovora_Saccharomycetales
Mmelibiosi_Saccharomycetales
Kpfeilii_Pezizales
Cfulgens_Pezizales
Aoligospora_Orbiliales
Mhaptotylum_Orbiliales
Abenhamiae_Onygenales
Psteckii_Eurotiales
Aaculeatus_Eurotiales
Arestrictus_Eurotiales
Aterreus_Eurotiales
Aarachidicola_Eurotiales
Acaelatus_Eurotiales
Uflorida_Lecanorales
Xparietina_Teloschistales
Skochii_Symbiotaphrinales
Tguianense_Xylonales
Mmicroscopicum_Microthyriales
Arichmondensis_Capnodiales
Czeae-maydis_Capnodiales
Zpseudotritici_Capnodiales
Sproteae_Botryosphaeriales
Patrata_Patellariales
Teluteriae_Trypetheliales
Pcitrichinaensis_Botryosphaeriales
Mphaseolina_Botryosphaeriales
Lpalustris_Mytilinidiales
Lmytilinum_Mytilinidiales
Bcircinans_Pleosporales
Dexigua_Pleosporales
Lmaculans_Pleosporales
Ffenestrata_Pleosporales
Enecator_Erysiphales
Ljuncicola_Helotiales
Lmacrosporus_Helotiales
Pscopiformis_Helotiales
Amacrosclerotiorum_Helotiales
Mbicolor_Helotiales
Clongipes_Helotiales
Rericae_Helotiales
Asarcoides_Helotiales
Glozoyensis_Helotiales
Zvarium_Lulworthiales
Tstercoreus_Thelebolales
Tmicrosporus_Thelebolales
Edeformans_Rhytismatales
Pelatina_Triblidiales
Lnitens_Rhytismatales
Cstrobi_Rhytismatales
Sflavida_Rhytismatales
Binquinans_Helotiales
Rfirma_Helotiales
Bpaeoniae_Helotiales
Bmedusae_Helotiales
Bnarcissicola_Helotiales
Bporri_Helotiales
Bcalthae_Helotiales
Bhyacinthi_Helotiales
Bpseudocinerea_Helotiales
Bconvoluta_Helotiales
Belliptica_Helotiales
Bcinerea_Helotiales
Btulipae_Helotiales
Bgalanthina_Helotiales
Tradiata_Torpedosporales
Mtrigonosporus_Microascales
Cmaritima_Microascales
Colidum_Hypocreales
Crosea_Hypocreales
Minundatum_Hypocreales
Bbassiana_Hypocreales
Cmilitaris_Hypocreales
Vdahliae_Hypocreales
Salkalinus_Glomerellales
Pcucumerina_Glomerellales
Czoysiae_Glomerellales
Gacutata_Glomerellales
Cpulchella_Calosphaeriales
Cligniaria_Coniochaetales
Gtetrasperma_Sordariales
Cbulbillosum_Sordariales
Paleophilum_Togniniales
Lminuta_Diaporthales
Dampelina_Diaporthales
Cchrysosperma_Diaporthales
Clustricola_Diaporthales
Cpopulea_Diaporthales
Moryzae_Magnaporthales
Ggraminis_Magnaporthales
Mpoae_Magnaporthales
Lthalassiae_Lulworthiales
Mtiffanyae_Melanosporales
Opiliferum_Ophiostomatales
Gclavigera_Ophiostomatales
Amontagnei_Xylariales
Koryzae_Trichosphaeriales
Cinnumera_Chaetosphaeriales
Pvexata_Xylariales
Bnummularia_Xylariales
Deschscholzii_Xylariales
Aavocetta_Xylariales
taxa
20
40
60
80
100
0
1
2
3
4
5
6
7
8
branch length

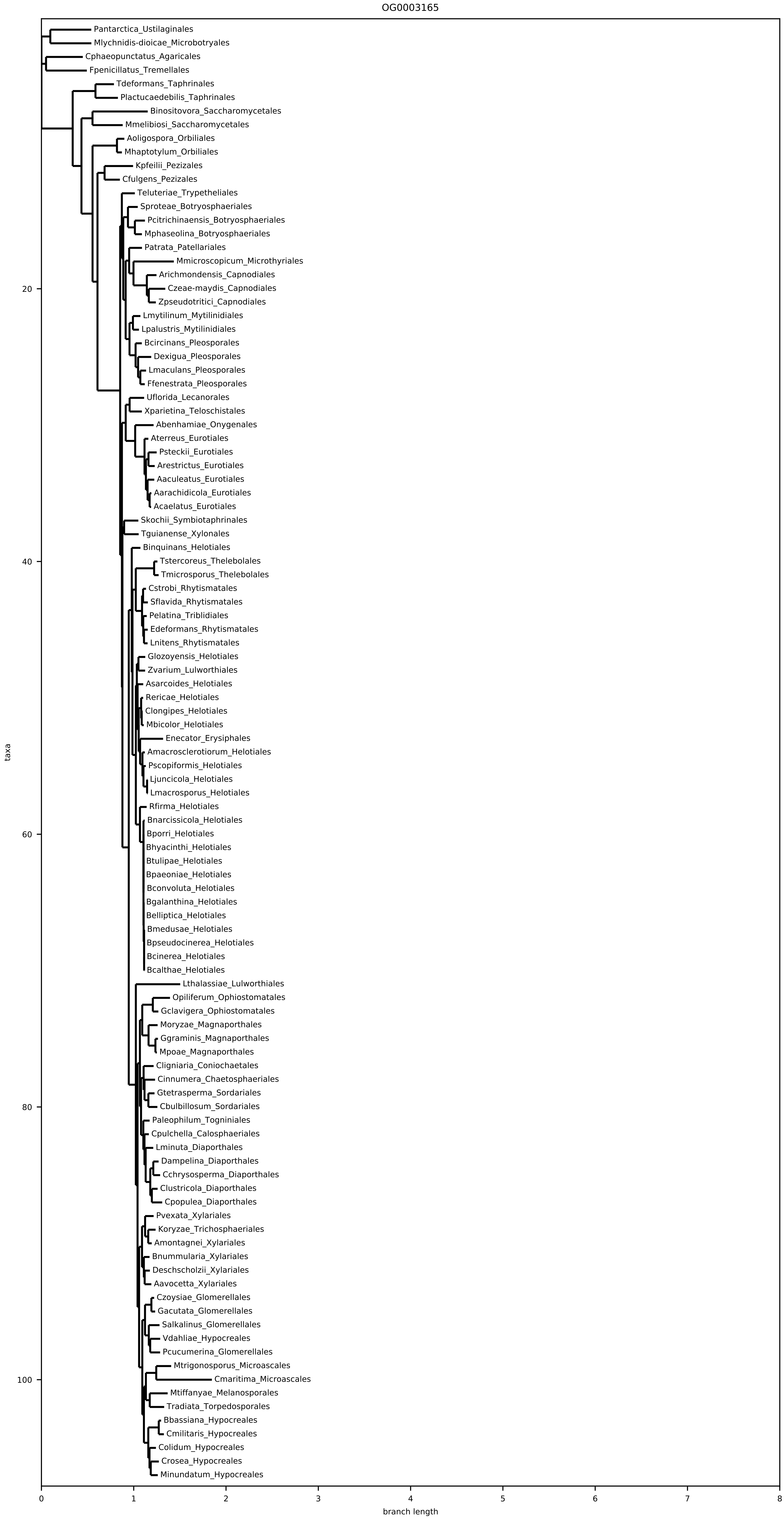

OG0003165
Pantarctica_Ustilaginales
Mlychnidis-dioicae_Microbotryales
Cphaeopunctatus_Agaricales
Fpenicillatus_Tremellales
Tdeformans_Taphrinales
Plactucaedebilis_Taphrinales
Binositovora_Saccharomycetales
Mmelibiosi_Saccharomycetales
Aoligospora_Orbiliales
Mhaptotylum_Orbiliales
Kpfeilii_Pezizales
Cfulgens_Pezizales
Teluteriae_Trypetheliales
Sproteae_Botryosphaeriales
Pcitrichinaensis_Botryosphaeriales
Mphaseolina_Botryosphaeriales
Patrata_Patellariales
Mmicroscopicum_Microthyriales
Arichmondensis_Capnodiales
Czeae-maydis_Capnodiales
Zpseudotritici_Capnodiales
Lmytilinum_Mytilinidiales
Lpalustris_Mytilinidiales
Bcircinans_Pleosporales
Dexigua_Pleosporales
Lmaculans_Pleosporales
Ffenestrata_Pleosporales
Uflorida_Lecanorales
Xparietina_Teloschistales
Abenhamiae_Onygenales
Aterreus_Eurotiales
Psteckii_Eurotiales
Arestrictus_Eurotiales
Aaculeatus_Eurotiales
Aarachidicola_Eurotiales
Acaelatus_Eurotiales
Skochii_Symbiotaphrinales
Tguianense_Xylonales
Binquinans_Helotiales
Tstercoreus_Thelebolales
Tmicrosporus_Thelebolales
Cstrobi_Rhytismatales
Sflavida_Rhytismatales
Pelatina_Triblidiales
Edeformans_Rhytismatales
Lnitens_Rhytismatales
Glozoyensis_Helotiales
Zvarium_Lulworthiales
Asarcoides_Helotiales
Rericae_Helotiales
Clongipes_Helotiales
Mbicolor_Helotiales
Enecator_Erysiphales
Amacrosclerotiorum_Helotiales
Pscopiformis_Helotiales
Ljuncicola_Helotiales
Lmacrosporus_Helotiales
Rfirma_Helotiales
Bnarcissicola_Helotiales
Bporri_Helotiales
Bhyacinthi_Helotiales
Btulipae_Helotiales
Bpaeoniae_Helotiales
Bconvoluta_Helotiales
Bgalanthina_Helotiales
Belliptica_Helotiales
Bmedusae_Helotiales
Bpseudocinerea_Helotiales
Bcinerea_Helotiales
Bcalthae_Helotiales
Lthalassiae_Lulworthiales
Opiliferum_Ophiostomatales
Gclavigera_Ophiostomatales
Moryzae_Magnaporthales
Ggraminis_Magnaporthales
Mpoae_Magnaporthales
Cligniaria_Coniochaetales
Cinnumera_Chaetosphaeriales
Gtetrasperma_Sordariales
Cbulbillosum_Sordariales
Paleophilum_Togniniales
Cpulchella_Calosphaeriales
Lminuta_Diaporthales
Dampelina_Diaporthales
Cchrysosperma_Diaporthales
Clustricola_Diaporthales
Cpopulea_Diaporthales
Pvexata_Xylariales
Koryzae_Trichosphaeriales
Amontagnei_Xylariales
Bnummularia_Xylariales
Deschscholzii_Xylariales
Aavocetta_Xylariales
Czoysiae_Glomerellales
Gacutata_Glomerellales
Salkalinus_Glomerellales
Vdahliae_Hypocreales
Pcucumerina_Glomerellales
Mtrigonosporus_Microascales
Cmaritima_Microascales
Mtiffanyae_Melanosporales
Tradiata_Torpedosporales
Bbassiana_Hypocreales
Cmilitaris_Hypocreales
Colidum_Hypocreales
Crosea_Hypocreales
Minundatum_Hypocreales
taxa
20
40
60
80
100
0
1
2
3
4
5
6
7
8
branch length

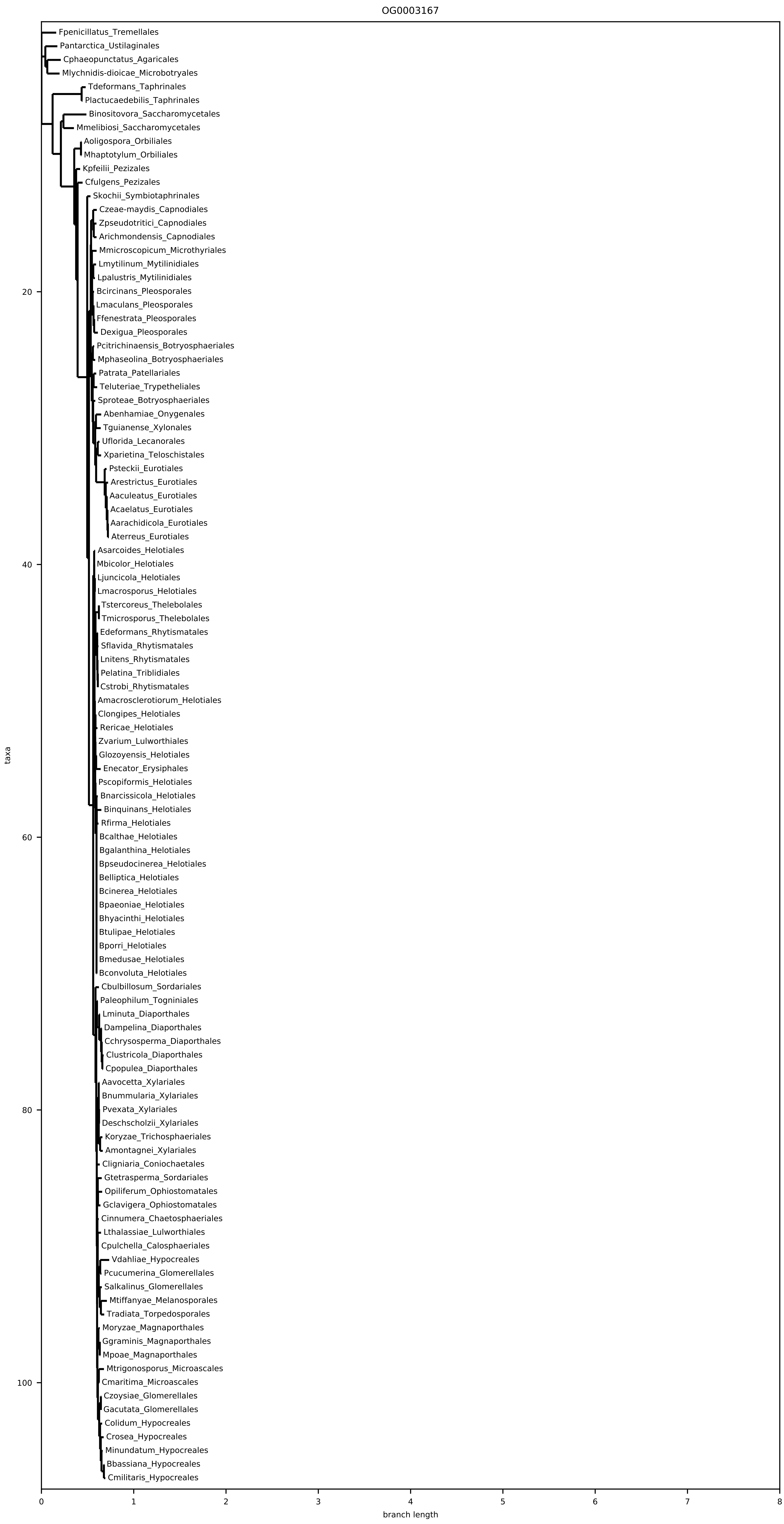
OG0003167
Fpenicillatus_Tremellales
Pantarctica_Ustilaginales
Cphaeopunctatus_Agaricales
Mlychnidis-dioicae_Microbotryales
Tdeformans_Taphrinales
Plactucaedebilis_Taphrinales
Binositovora_Saccharomycetales
Mmelibiosi_Saccharomycetales
Aoligospora_Orbiliales
Mhaptotylum_Orbiliales
Kpfeilii_Pezizales
Cfulgens_Pezizales
Skochii_Symbiotaphrinales
Czeae-maydis_Capnodiales
Zpseudotritici_Capnodiales
Arichmondensis_Capnodiales
Mmicroscopicum_Microthyriales
Lmytilinum_Mytilinidiales
Lpalustris_Mytilinidiales
Bcircinans_Pleosporales
Lmaculans_Pleosporales
Ffenestrata_Pleosporales
Dexigua_Pleosporales
Pcitrichinaensis_Botryosphaeriales
Mphaseolina_Botryosphaeriales
Patrata_Patellariales
Teluteriae_Trypetheliales
Sproteae_Botryosphaeriales
Abenhamiae_Onygenales
Tguianense_Xylonales
Uflorida_Lecanorales
Xparietina_Teloschistales
Psteckii_Eurotiales
Arestrictus_Eurotiales
Aaculeatus_Eurotiales
Acaelatus_Eurotiales
Aarachidicola_Eurotiales
Aterreus_Eurotiales
Asarcoides_Helotiales
Mbicolor_Helotiales
Ljuncicola_Helotiales
Lmacrosporus_Helotiales
Tstercoreus_Thelebolales
Tmicrosporus_Thelebolales
Edeformans_Rhytismatales
Sflavida_Rhytismatales
Lnitens_Rhytismatales
Pelatina_Triblidiales
Cstrobi_Rhytismatales
Amacrosclerotiorum_Helotiales
Clongipes_Helotiales
Rericae_Helotiales
Zvarium_Lulworthiales
Glozoyensis_Helotiales
Enecator_Erysiphales
Pscopiformis_Helotiales
Bnarcissicola_Helotiales
Binquinans_Helotiales
Rfirma_Helotiales
Bcalthae_Helotiales
Bgalanthina_Helotiales
Bpseudocinerea_Helotiales
Belliptica_Helotiales
Bcinerea_Helotiales
Bpaeoniae_Helotiales
Bhyacinthi_Helotiales
Btulipae_Helotiales
Bporri_Helotiales
Bmedusae_Helotiales
Bconvoluta_Helotiales
Cbulbillosum_Sordariales
Paleophilum_Togniniales
Lminuta_Diaporthales
Dampelina_Diaporthales
Cchrysosperma_Diaporthales
Clustricola_Diaporthales
Cpopulea_Diaporthales
Aavocetta_Xylariales
Bnummularia_Xylariales
Pvexata_Xylariales
Deschscholzii_Xylariales
Koryzae_Trichosphaeriales
Amontagnei_Xylariales
Cligniaria_Coniochaetales
Gtetrasperma_Sordariales
Opiliferum_Ophiostomatales
Gclavigera_Ophiostomatales
Cinnumera_Chaetosphaeriales
Lthalassiae_Lulworthiales
Cpulchella_Calosphaeriales
Vdahliae_Hypocreales
Pcucumerina_Glomerellales
Salkalinus_Glomerellales
Mtiffanyae_Melanosporales
Tradiata_Torpedosporales
Moryzae_Magnaporthales
Ggraminis_Magnaporthales
Mpoae_Magnaporthales
Mtrigonosporus_Microascales
Cmaritima_Microascales
Czoysiae_Glomerellales
Gacutata_Glomerellales
Colidum_Hypocreales
Crosea_Hypocreales
Minundatum_Hypocreales
Bbassiana_Hypocreales
Cmilitaris_Hypocreales
taxa
20
40
60
80
100
0
1
2
3
4
5
6
7
8
branch length

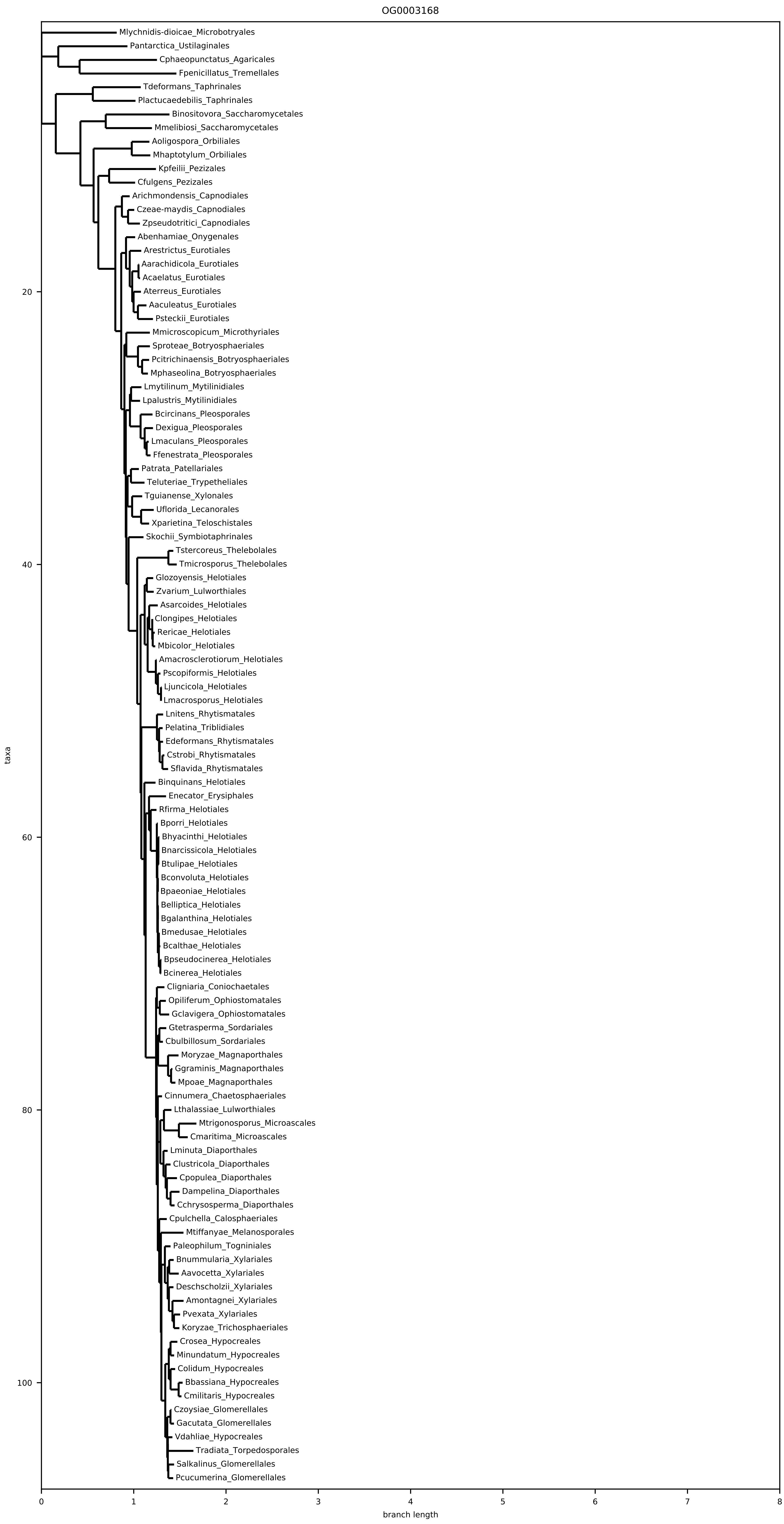
OG0003168
Mlychnidis-dioicae_Microbotryales
Pantarctica_Ustilaginales
Cphaeopunctatus_Agaricales
Fpenicillatus_Tremellales
Tdeformans_Taphrinales
Plactucaedebilis_Taphrinales
Binositovora_Saccharomycetales
Mmelibiosi_Saccharomycetales
Aoligospora_Orbiliales
Mhaptotylum_Orbiliales
Kpfeilii_Pezizales
Cfulgens_Pezizales
Arichmondensis_Capnodiales
Czeae-maydis_Capnodiales
Zpseudotritici_Capnodiales
Abenhamiae_Onygenales
Arestrictus_Eurotiales
Aarachidicola_Eurotiales
Acaelatus_Eurotiales
Aterreus_Eurotiales
Aaculeatus_Eurotiales
Psteckii_Eurotiales
Mmicroscopicum_Microthyriales
Sproteae_Botryosphaeriales
Pcitrichinaensis_Botryosphaeriales
Mphaseolina_Botryosphaeriales
Lmytilinum_Mytilinidiales
Lpalustris_Mytilinidiales
Bcircinans_Pleosporales
Dexigua_Pleosporales
Lmaculans_Pleosporales
Ffenestrata_Pleosporales
Patrata_Patellariales
Teluteriae_Trypetheliales
Tguianense_Xylonales
Uflorida_Lecanorales
Xparietina_Teloschistales
Skochii_Symbiotaphrinales
Tstercoreus_Thelebolales
Tmicrosporus_Thelebolales
Glozoyensis_Helotiales
Zvarium_Lulworthiales
Asarcoides_Helotiales
Clongipes_Helotiales
Rericae_Helotiales
Mbicolor_Helotiales
Amacrosclerotiorum_Helotiales
Pscopiformis_Helotiales
Ljuncicola_Helotiales
Lmacrosporus_Helotiales
Lnitens_Rhytismatales
Pelatina_Triblidiales
Edeformans_Rhytismatales
Cstrobi_Rhytismatales
Sflavida_Rhytismatales
Binquinans_Helotiales
Enecator_Erysiphales
Rfirma_Helotiales
Bporri_Helotiales
Bhyacinthi_Helotiales
Bnarcissicola_Helotiales
Btulipae_Helotiales
Bconvoluta_Helotiales
Bpaeoniae_Helotiales
Belliptica_Helotiales
Bgalanthina_Helotiales
Bmedusae_Helotiales
Bcalthae_Helotiales
Bpseudocinerea_Helotiales
Bcinerea_Helotiales
Cligniaria_Coniochaetales
Opiliferum_Ophiostomatales
Gclavigera_Ophiostomatales
Gtetrasperma_Sordariales
Cbulbillosum_Sordariales
Moryzae_Magnaporthales
Ggraminis_Magnaporthales
Mpoae_Magnaporthales
Cinnumera_Chaetosphaeriales
Lthalassiae_Lulworthiales
Mtrigonosporus_Microascales
Cmaritima_Microascales
Lminuta_Diaporthales
Clustricola_Diaporthales
Cpopulea_Diaporthales
Dampelina_Diaporthales
Cchrysosperma_Diaporthales
Cpulchella_Calosphaeriales
Mtiffanyae_Melanosporales
Paleophilum_Togniniales
Bnummularia_Xylariales
Aavocetta_Xylariales
Deschscholzii_Xylariales
Amontagnei_Xylariales
Pvexata_Xylariales
Koryzae_Trichosphaeriales
Crosea_Hypocreales
Minundatum_Hypocreales
Colidum_Hypocreales
Bbassiana_Hypocreales
Cmilitaris_Hypocreales
Czoysiae_Glomerellales
Gacutata_Glomerellales
Vdahliae_Hypocreales
Tradiata_Torpedosporales
Salkalinus_Glomerellales
Pcucumerina_Glomerellales
taxa
20
40
60
80
100
branch length
0
1
2
3
4
5
6
7
8

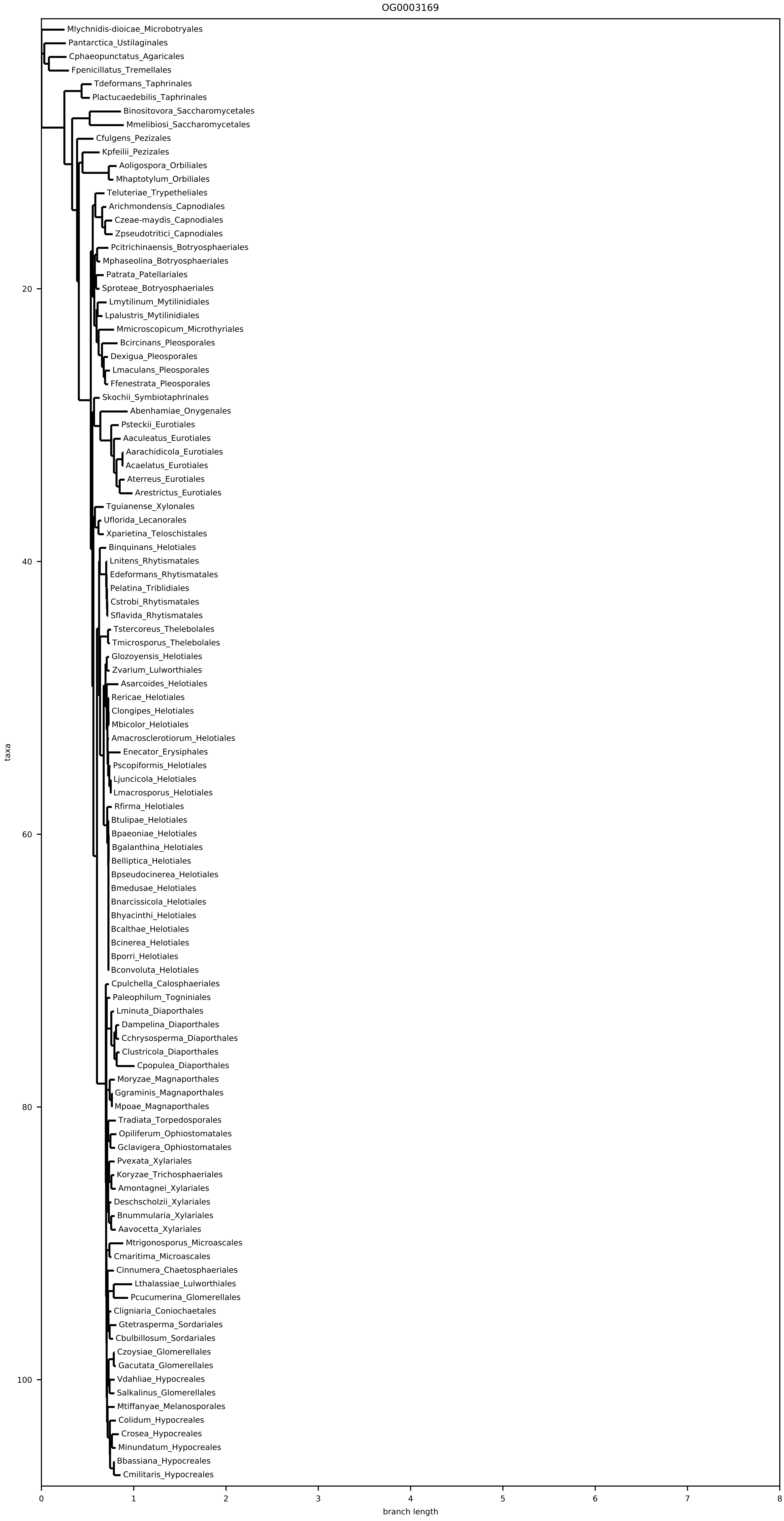
OG0003169
Mlychnidis-dioicae_Microbotryales
Pantarctica_Ustilaginales
Cphaeopunctatus_Agaricales
Fpenicillatus_Tremellales
Tdeformans_Taphrinales
Plactucaedebilis_Taphrinales
Binositovora_Saccharomycetales
Mmelibiosi_Saccharomycetales
Cfulgens_Pezizales
Kpfeilii_Pezizales
Aoligospora_Orbiliales
Mhaptotylum_Orbiliales
Teluteriae_Trypetheliales
Arichmondensis_Capnodiales
Czeae-maydis_Capnodiales
Zpseudotritici_Capnodiales
Pcitrichinaensis_Botryosphaeriales
Mphaseolina_Botryosphaeriales
Patrata_Patellariales
Sproteae_Botryosphaeriales
Lmytilinum_Mytilinidiales
Lpalustris_Mytilinidiales
Mmicroscopicum_Microthyriales
Bcircinans_Pleosporales
Dexigua_Pleosporales
Lmaculans_Pleosporales
Ffenestrata_Pleosporales
Skochii_Symbiotaphrinales
Abenhamiae_Onygenales
Psteckii_Eurotiales
Aaculeatus_Eurotiales
Aarachidicola_Eurotiales
Acaelatus_Eurotiales
Aterreus_Eurotiales
Arestrictus_Eurotiales
Tguianense_Xylonales
Uflorida_Lecanorales
Xparietina_Teloschistales
Binquinans_Helotiales
Lnitens_Rhytismatales
Edeformans_Rhytismatales
Pelatina_Triblidiales
Cstrobi_Rhytismatales
Sflavida_Rhytismatales
Tstercoreus_Thelebolales
Tmicrosporus_Thelebolales
Glozoyensis_Helotiales
Zvarium_Lulworthiales
Asarcoides_Helotiales
Rericae_Helotiales
Clongipes_Helotiales
Mbicolor_Helotiales
Amacrosclerotiorum_Helotiales
Enecator_Erysiphales
Pscopiformis_Helotiales
Ljuncicola_Helotiales
Lmacrosporus_Helotiales
Rfirma_Helotiales
Btulipae_Helotiales
Bpaeoniae_Helotiales
Bgalanthina_Helotiales
Belliptica_Helotiales
Bpseudocinerea_Helotiales
Bmedusae_Helotiales
Bnarcissicola_Helotiales
Bhyacinthi_Helotiales
Bcalthae_Helotiales
Bcinerea_Helotiales
Bporri_Helotiales
Bconvoluta_Helotiales
Cpulchella_Calosphaeriales
Paleophilum_Togniniales
Lminuta_Diaporthales
Dampelina_Diaporthales
Cchrysosperma_Diaporthales
Clustricola_Diaporthales
Cpopulea_Diaporthales
Moryzae_Magnaporthales
Ggraminis_Magnaporthales
Mpoae_Magnaporthales
Tradiata_Torpedosporales
Opiliferum_Ophiostomatales
Gclavigera_Ophiostomatales
Pvexata_Xylariales
Koryzae_Trichosphaeriales
Amontagnei_Xylariales
Deschscholzii_Xylariales
Bnummularia_Xylariales
Aavocetta_Xylariales
Mtrigonosporus_Microascales
Cmaritima_Microascales
Cinnumera_Chaetosphaeriales
Lthalassiae_Lulworthiales
Pcucumerina_Glomerellales
Cligniaria_Coniochaetales
Gtetrasperma_Sordariales
Cbulbillosum_Sordariales
Czoysiae_Glomerellales
Gacutata_Glomerellales
Vdahliae_Hypocreales
Salkalinus_Glomerellales
Mtiffanyae_Melanosporales
Colidum_Hypocreales
Crosea_Hypocreales
Minundatum_Hypocreales
Bbassiana_Hypocreales
Cmilitaris_Hypocreales
taxa
20
40
60
80
100
0
1
2
3
4
5
6
7
8
branch length

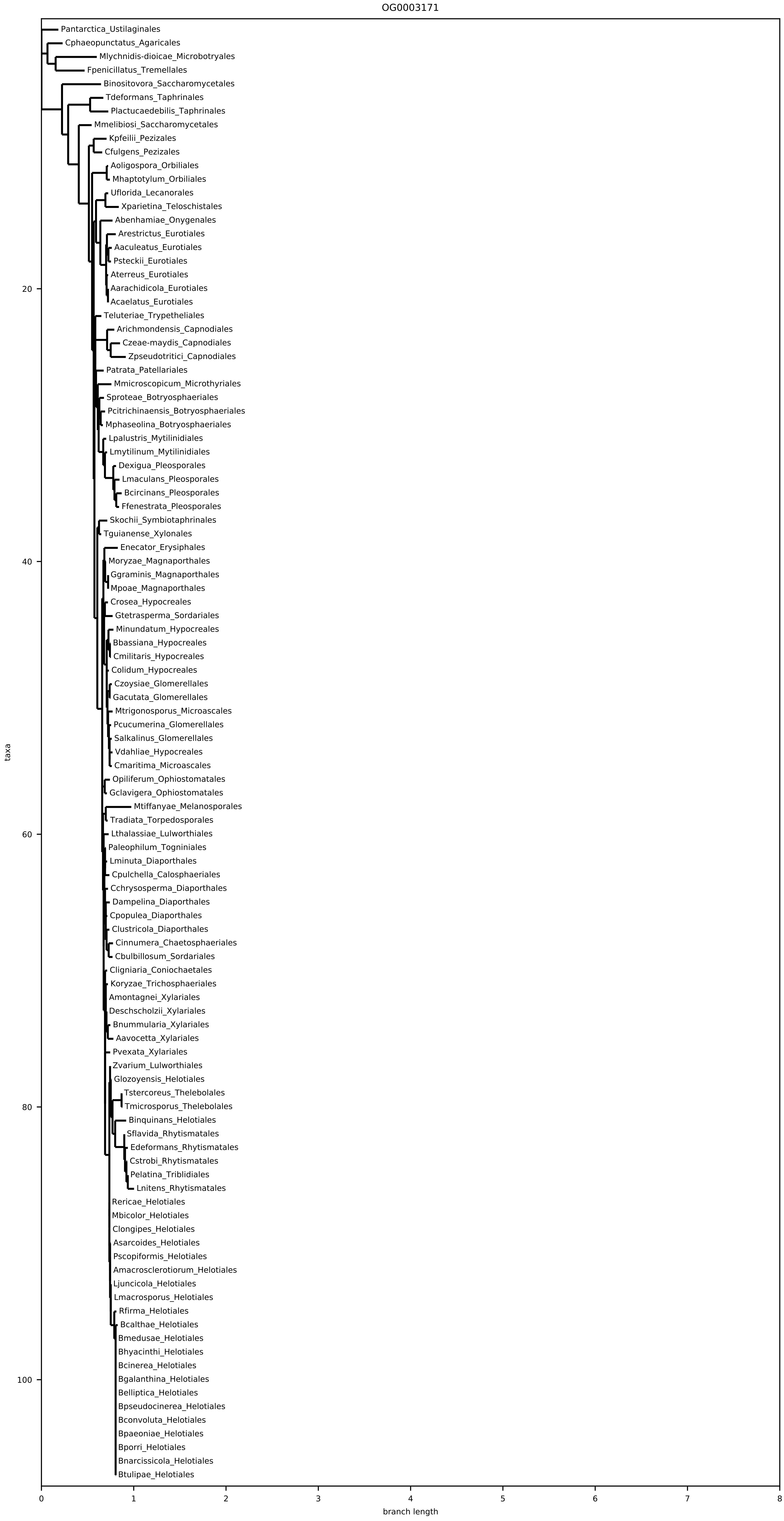
OG0003171
Pantarctica_Ustilaginales
Cphaeopunctatus_Agaricales
Mlychnidis-dioicae_Microbotryales
Fpenicillatus_Tremellales
Binositovora_Saccharomycetales
Tdeformans_Taphrinales
Plactucaedebilis_Taphrinales
Mmelibiosi_Saccharomycetales
Kpfeilii_Pezizales
Cfulgens_Pezizales
Aoligospora_Orbiliales
Mhaptotylum_Orbiliales
Uflorida_Lecanorales
Xparietina_Teloschistales
Abenhamiae_Onygenales
Arestrictus_Eurotiales
Aaculeatus_Eurotiales
Psteckii_Eurotiales
Aterreus_Eurotiales
Aarachidicola_Eurotiales
Acaelatus_Eurotiales
Teluteriae_Trypetheliales
Arichmondensis_Capnodiales
Czeae-maydis_Capnodiales
Zpseudotritici_Capnodiales
Patrata_Patellariales
Mmicroscopicum_Microthyriales
Sproteae_Botryosphaeriales
Pcitrichinaensis_Botryosphaeriales
Mphaseolina_Botryosphaeriales
Lpalustris_Mytilinidiales
Lmytilinum_Mytilinidiales
Dexigua_Pleosporales
Lmaculans_Pleosporales
Bcircinans_Pleosporales
Ffenestrata_Pleosporales
Skochii_Symbiotaphrinales
Tguianense_Xylonales
Enecator_Erysiphales
Moryzae_Magnaporthales
Ggraminis_Magnaporthales
Mpoae_Magnaporthales
Crosea_Hypocreales
Gtetrasperma_Sordariales
Minundatum_Hypocreales
Bbassiana_Hypocreales
Cmilitaris_Hypocreales
Colidum_Hypocreales
Czoysiae_Glomerellales
Gacutata_Glomerellales
Mtrigonosporus_Microascales
Pcucumerina_Glomerellales
Salkalinus_Glomerellales
Vdahliae_Hypocreales
Cmaritima_Microascales
Opiliferum_Ophiostomatales
Gclavigera_Ophiostomatales
Mtiffanyae_Melanosporales
Tradiata_Torpedosporales
Lthalassiae_Lulworthiales
Paleophilum_Togniniales
Lminuta_Diaporthales
Cpulchella_Calosphaeriales
Cchrysosperma_Diaporthales
Dampelina_Diaporthales
Cpopulea_Diaporthales
Clustricola_Diaporthales
Cinnumera_Chaetosphaeriales
Cbulbillosum_Sordariales
Cligniaria_Coniochaetales
Koryzae_Trichosphaeriales
Amontagnei_Xylariales
Deschscholzii_Xylariales
Bnummularia_Xylariales
Aavocetta_Xylariales
Pvexata_Xylariales
Zvarium_Lulworthiales
Glozoyensis_Helotiales
Tstercoreus_Thelebolales
Tmicrosporus_Thelebolales
Binquinans_Helotiales
Sflavida_Rhytismatales
Edeformans_Rhytismatales
Cstrobi_Rhytismatales
Pelatina_Triblidiales
Lnitens_Rhytismatales
Rericae_Helotiales
Mbicolor_Helotiales
Clongipes_Helotiales
Asarcoides_Helotiales
Pscopiformis_Helotiales
Amacrosclerotiorum_Helotiales
Ljuncicola_Helotiales
Lmacrosporus_Helotiales
Rfirma_Helotiales
Bcalthae_Helotiales
Bmedusae_Helotiales
Bhyacinthi_Helotiales
Bcinerea_Helotiales
Bgalanthina_Helotiales
Belliptica_Helotiales
Bpseudocinerea_Helotiales
Bconvoluta_Helotiales
Bpaeoniae_Helotiales
Bporri_Helotiales
Bnarcissicola_Helotiales
Btulipae_Helotiales
taxa
20
40
60
80
100
0
1
2
3
4
5
6
7
8
branch length

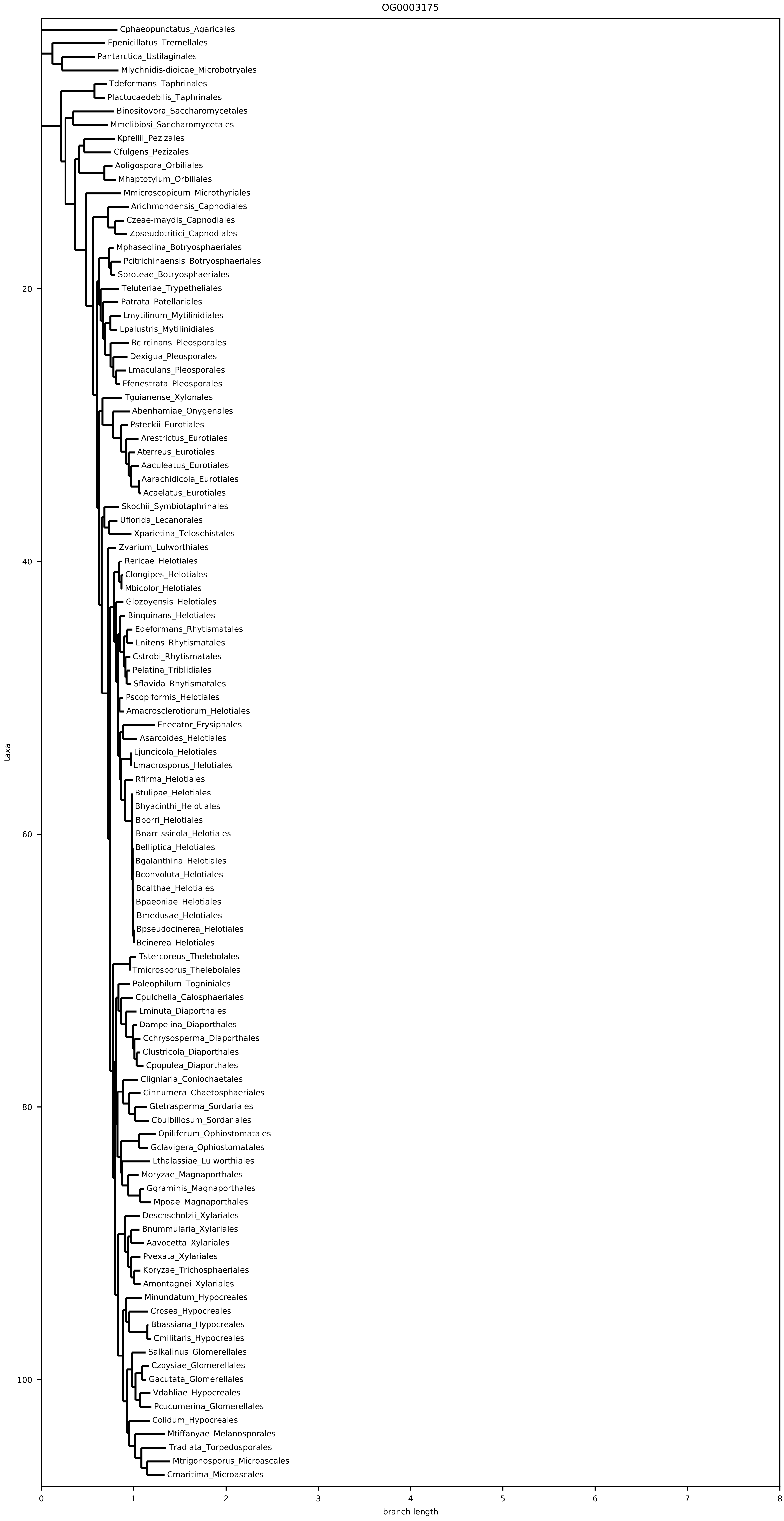
OG0003175
Cphaeopunctatus_Agaricales
Fpenicillatus_Tremellales
Pantarctica_Ustilaginales
Mlychnidis-dioicae_Microbotryales
Tdeformans_Taphrinales
Plactucaedebilis_Taphrinales
Binositovora_Saccharomycetales
Mmelibiosi_Saccharomycetales
Kpfeilii_Pezizales
Cfulgens_Pezizales
Aoligospora_Orbiliales
Mhaptotylum_Orbiliales
Mmicroscopicum_Microthyriales
Arichmondensis_Capnodiales
Czeae-maydis_Capnodiales
Zpseudotritici_Capnodiales
Mphaseolina_Botryosphaeriales
Pcitrichinaensis_Botryosphaeriales
Sproteae_Botryosphaeriales
Teluteriae_Trypetheliales
Patrata_Patellariales
Lmytilinum_Mytilinidiales
Lpalustris_Mytilinidiales
Bcircinans_Pleosporales
Dexigua_Pleosporales
Lmaculans_Pleosporales
Ffenestrata_Pleosporales
Tguianense_Xylonales
Abenhamiae_Onygenales
Psteckii_Eurotiales
Arestrictus_Eurotiales
Aterreus_Eurotiales
Aaculeatus_Eurotiales
Aarachidicola_Eurotiales
Acaelatus_Eurotiales
Skochii_Symbiotaphrinales
Uflorida_Lecanorales
Xparietina_Teloschistales
Zvarium_Lulworthiales
Rericae_Helotiales
Clongipes_Helotiales
Mbicolor_Helotiales
Glozoyensis_Helotiales
Binquinans_Helotiales
Edeformans_Rhytismatales
Lnitens_Rhytismatales
Cstrobi_Rhytismatales
Pelatina_Triblidiales
Sflavida_Rhytismatales
Pscopiformis_Helotiales
Amacrosclerotiorum_Helotiales
Enecator_Erysiphales
Asarcoides_Helotiales
Ljuncicola_Helotiales
Lmacrosporus_Helotiales
Rfirma_Helotiales
Btulipae_Helotiales
Bhyacinthi_Helotiales
Bporri_Helotiales
Bnarcissicola_Helotiales
Belliptica_Helotiales
Bgalanthina_Helotiales
Bconvoluta_Helotiales
Bcalthae_Helotiales
Bpaeoniae_Helotiales
Bmedusae_Helotiales
Bpseudocinerea_Helotiales
Bcinerea_Helotiales
Tstercoreus_Thelebolales
Tmicrosporus_Thelebolales
Paleophilum_Togniniales
Cpulchella_Calosphaeriales
Lminuta_Diaporthales
Dampelina_Diaporthales
Cchrysosperma_Diaporthales
Clustricola_Diaporthales
Cpopulea_Diaporthales
Cligniaria_Coniochaetales
Cinnumera_Chaetosphaeriales
Gtetrasperma_Sordariales
Cbulbillosum_Sordariales
Opiliferum_Ophiostomatales
Gclavigera_Ophiostomatales
Lthalassiae_Lulworthiales
Moryzae_Magnaporthales
Ggraminis_Magnaporthales
Mpoae_Magnaporthales
Deschscholzii_Xylariales
Bnummularia_Xylariales
Aavocetta_Xylariales
Pvexata_Xylariales
Koryzae_Trichosphaeriales
Amontagnei_Xylariales
Minundatum_Hypocreales
Crosea_Hypocreales
Bbassiana_Hypocreales
Cmilitaris_Hypocreales
Salkalinus_Glomerellales
Czoysiae_Glomerellales
Gacutata_Glomerellales
Vdahliae_Hypocreales
Pcucumerina_Glomerellales
Colidum_Hypocreales
Mtiffanyae_Melanosporales
Tradiata_Torpedosporales
Mtrigonosporus_Microascales
Cmaritima_Microascales
taxa
20
40
60
80
100
0
1
2
3
4
5
6
7
8
branch length

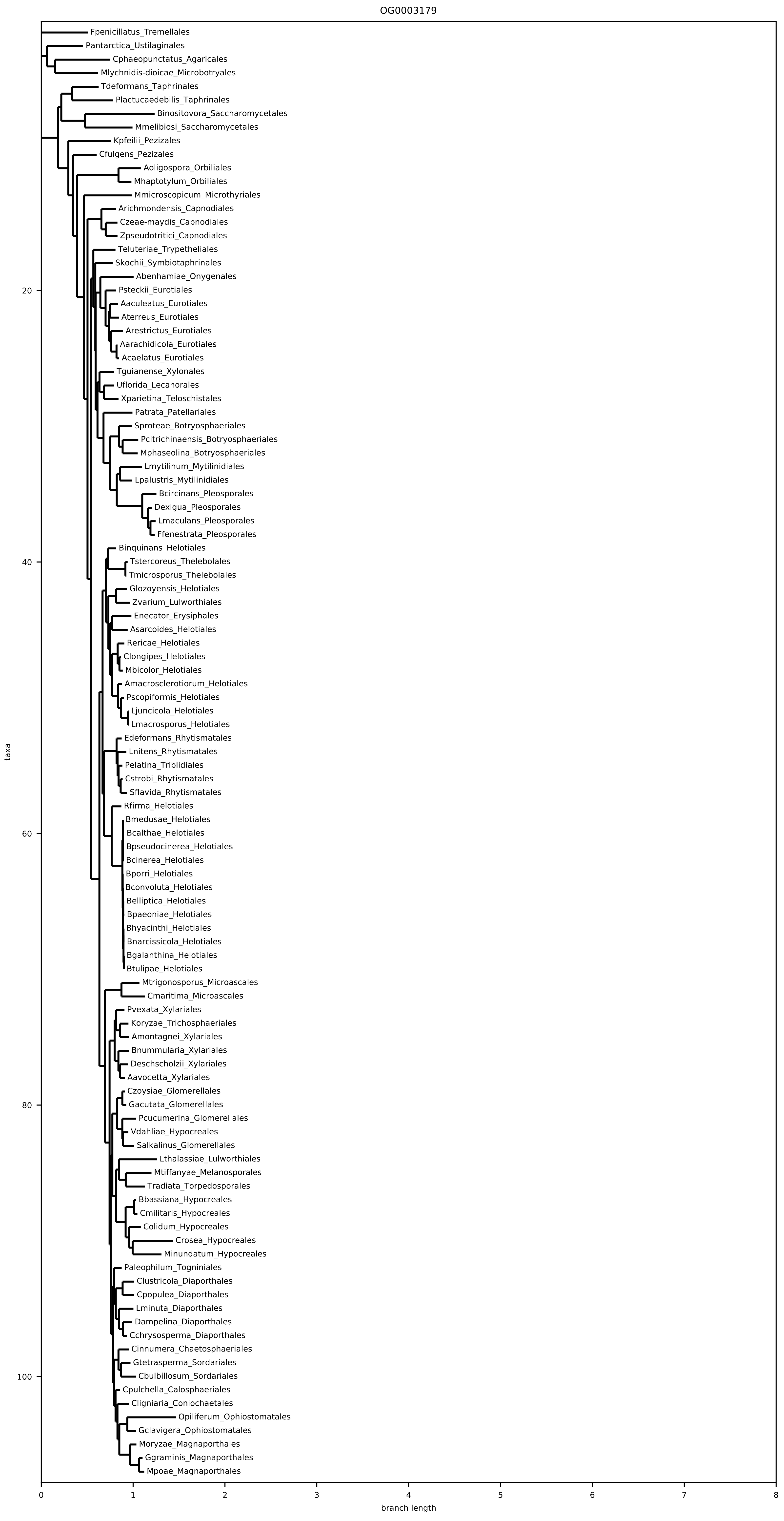
OG0003179
Fpenicillatus_Tremellales
Pantarctica_Ustilaginales
Cphaeopunctatus_Agaricales
Mlychnidis-dioicae_Microbotryales
Tdeformans_Taphrinales
Plactucaedebilis_Taphrinales
Binositovora_Saccharomycetales
Mmelibiosi_Saccharomycetales
Kpfeilii_Pezizales
Cfulgens_Pezizales
Aoligospora_Orbiliales
Mhaptotylum_Orbiliales
Mmicroscopicum_Microthyriales
Arichmondensis_Capnodiales
Czeae-maydis_Capnodiales
Zpseudotritici_Capnodiales
Teluteriae_Trypetheliales
Skochii_Symbiotaphrinales
Abenhamiae_Onygenales
Psteckii_Eurotiales
Aaculeatus_Eurotiales
Aterreus_Eurotiales
Arestrictus_Eurotiales
Aarachidicola_Eurotiales
Acaelatus_Eurotiales
Tguianense_Xylonales
Uflorida_Lecanorales
Xparietina_Teloschistales
Patrata_Patellariales
Sproteae_Botryosphaeriales
Pcitrichinaensis_Botryosphaeriales
Mphaseolina_Botryosphaeriales
Lmytilinum_Mytilinidiales
Lpalustris_Mytilinidiales
Bcircinans_Pleosporales
Dexigua_Pleosporales
Lmaculans_Pleosporales
Ffenestrata_Pleosporales
Binquinans_Helotiales
Tstercoreus_Thelebolales
Tmicrosporus_Thelebolales
Glozoyensis_Helotiales
Zvarium_Lulworthiales
Enecator_Erysiphales
Asarcoides_Helotiales
Rericae_Helotiales
Clongipes_Helotiales
Mbicolor_Helotiales
Amacrosclerotiorum_Helotiales
Pscopiformis_Helotiales
Ljuncicola_Helotiales
Lmacrosporus_Helotiales
Edeformans_Rhytismatales
Lnitens_Rhytismatales
Pelatina_Triblidiales
Cstrobi_Rhytismatales
Sflavida_Rhytismatales
Rfirma_Helotiales
Bmedusae_Helotiales
Bcalthae_Helotiales
Bpseudocinerea_Helotiales
Bcinerea_Helotiales
Bporri_Helotiales
Bconvoluta_Helotiales
Belliptica_Helotiales
Bpaeoniae_Helotiales
Bhyacinthi_Helotiales
Bnarcissicola_Helotiales
Bgalanthina_Helotiales
Btulipae_Helotiales
Mtrigonosporus_Microascales
Cmaritima_Microascales
Pvexata_Xylariales
Koryzae_Trichosphaeriales
Amontagnei_Xylariales
Bnummularia_Xylariales
Deschscholzii_Xylariales
Aavocetta_Xylariales
Czoysiae_Glomerellales
Gacutata_Glomerellales
Pcucumerina_Glomerellales
Vdahliae_Hypocreales
Salkalinus_Glomerellales
Lthalassiae_Lulworthiales
Mtiffanyae_Melanosporales
Tradiata_Torpedosporales
Bbassiana_Hypocreales
Cmilitaris_Hypocreales
Colidum_Hypocreales
Crosea_Hypocreales
Minundatum_Hypocreales
Paleophilum_Togniniales
Clustricola_Diaporthales
Cpopulea_Diaporthales
Lminuta_Diaporthales
Dampelina_Diaporthales
Cchrysosperma_Diaporthales
Cinnumera_Chaetosphaeriales
Gtetrasperma_Sordariales
Cbulbillosum_Sordariales
Cpulchella_Calosphaeriales
Cligniaria_Coniochaetales
Opiliferum_Ophiostomatales
Gclavigera_Ophiostomatales
Moryzae_Magnaporthales
Ggraminis_Magnaporthales
Mpoae_Magnaporthales
taxa
20
40
60
80
100
0
1
2
3
4
5
6
7
8
branch length

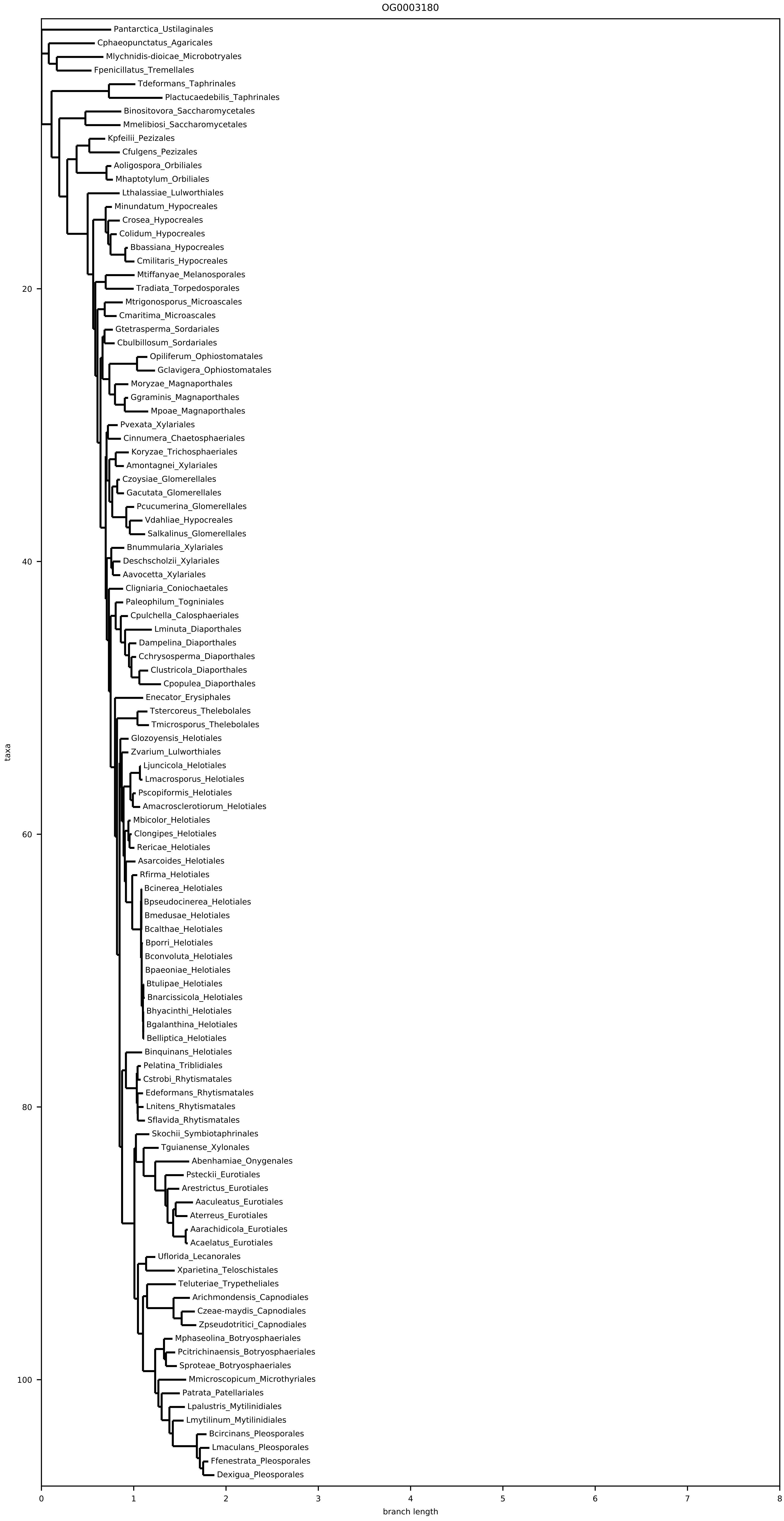
OG0003180
Pantarctica_Ustilaginales
Cphaeopunctatus_Agaricales
Mlychnidis-dioicae_Microbotryales
Fpenicillatus_Tremellales
Tdeformans_Taphrinales
Plactucaedebilis_Taphrinales
Binositovora_Saccharomycetales
Mmelibiosi_Saccharomycetales
Kpfeilii_Pezizales
Cfulgens_Pezizales
Aoligospora_Orbiliales
Mhaptotylum_Orbiliales
Lthalassiae_Lulworthiales
Minundatum_Hypocreales
Crosea_Hypocreales
Colidum_Hypocreales
Bbassiana_Hypocreales
Cmilitaris_Hypocreales
Mtiffanyae_Melanosporales
Tradiata_Torpedosporales
Mtrigonosporus_Microascales
Cmaritima_Microascales
Gtetrasperma_Sordariales
Cbulbillosum_Sordariales
Opiliferum_Ophiostomatales
Gclavigera_Ophiostomatales
Moryzae_Magnaporthales
Ggraminis_Magnaporthales
Mpoae_Magnaporthales
Pvexata_Xylariales
Cinnumera_Chaetosphaeriales
Koryzae_Trichosphaeriales
Amontagnei_Xylariales
Czoysiae_Glomerellales
Gacutata_Glomerellales
Pcucumerina_Glomerellales
Vdahliae_Hypocreales
Salkalinus_Glomerellales
Bnummularia_Xylariales
Deschscholzii_Xylariales
Aavocetta_Xylariales
Cligniaria_Coniochaetales
Paleophilum_Togniniales
Cpulchella_Calosphaeriales
Lminuta_Diaporthales
Dampelina_Diaporthales
Cchrysosperma_Diaporthales
Clustricola_Diaporthales
Cpopulea_Diaporthales
Enecator_Erysiphales
Tstercoreus_Thelebolales
Tmicrosporus_Thelebolales
Glozoyensis_Helotiales
Zvarium_Lulworthiales
Ljuncicola_Helotiales
Lmacrosporus_Helotiales
Pscopiformis_Helotiales
Amacrosclerotiorum_Helotiales
Mbicolor_Helotiales
Clongipes_Helotiales
Rericae_Helotiales
Asarcoides_Helotiales
Rfirma_Helotiales
Bcinerea_Helotiales
Bpseudocinerea_Helotiales
Bmedusae_Helotiales
Bcalthae_Helotiales
Bporri_Helotiales
Bconvoluta_Helotiales
Bpaeoniae_Helotiales
Btulipae_Helotiales
Bnarcissicola_Helotiales
Bhyacinthi_Helotiales
Bgalanthina_Helotiales
Belliptica_Helotiales
Binquinans_Helotiales
Pelatina_Triblidiales
Cstrobi_Rhytismatales
Edeformans_Rhytismatales
Lnitens_Rhytismatales
Sflavida_Rhytismatales
Skochii_Symbiotaphrinales
Tguianense_Xylonales
Abenhamiae_Onygenales
Psteckii_Eurotiales
Arestrictus_Eurotiales
Aaculeatus_Eurotiales
Aterreus_Eurotiales
Aarachidicola_Eurotiales
Acaelatus_Eurotiales
Uflorida_Lecanorales
Xparietina_Teloschistales
Teluteriae_Trypetheliales
Arichmondensis_Capnodiales
Czeae-maydis_Capnodiales
Zpseudotritici_Capnodiales
Mphaseolina_Botryosphaeriales
Pcitrichinaensis_Botryosphaeriales
Sproteae_Botryosphaeriales
Mmicroscopicum_Microthyriales
Patrata_Patellariales
Lpalustris_Mytilinidiales
Lmytilinum_Mytilinidiales
Bcircinans_Pleosporales
Lmaculans_Pleosporales
Ffenestrata_Pleosporales
Dexigua_Pleosporales
taxa
20
40
60
80
100
0
1
2
3
4
5
6
7
8
branch length

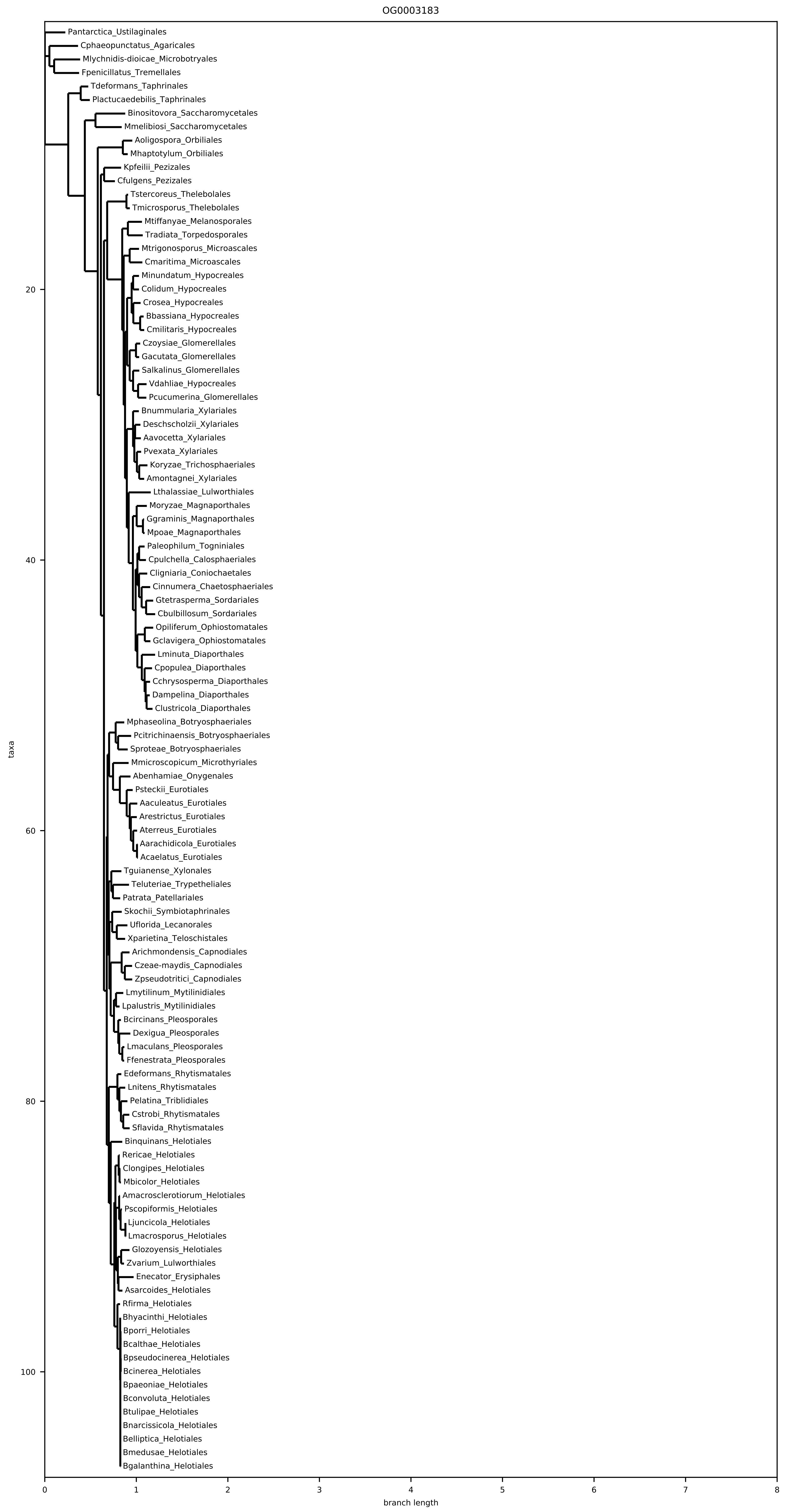
OG0003183
Pantarctica_Ustilaginales
Cphaeopunctatus_Agaricales
Mlychnidis-dioicae_Microbotryales
Fpenicillatus_Tremellales
Tdeformans_Taphrinales
Plactucaedebilis_Taphrinales
Binositovora_Saccharomycetales
Mmelibiosi_Saccharomycetales
Aoligospora_Orbiliales
Mhaptotylum_Orbiliales
Kpfeilii_Pezizales
Cfulgens_Pezizales
Tstercoreus_Thelebolales
Tmicrosporus_Thelebolales
Mtiffanyae_Melanosporales
Tradiata_Torpedosporales
Mtrigonosporus_Microascales
Cmaritima_Microascales
Minundatum_Hypocreales
Colidum_Hypocreales
Crosea_Hypocreales
Bbassiana_Hypocreales
Cmilitaris_Hypocreales
Czoysiae_Glomerellales
Gacutata_Glomerellales
Salkalinus_Glomerellales
Vdahliae_Hypocreales
Pcucumerina_Glomerellales
Bnummularia_Xylariales
Deschscholzii_Xylariales
Aavocetta_Xylariales
Pvexata_Xylariales
Koryzae_Trichosphaeriales
Amontagnei_Xylariales
Lthalassiae_Lulworthiales
Moryzae_Magnaporthales
Ggraminis_Magnaporthales
Mpoae_Magnaporthales
Paleophilum_Togniniales
Cpulchella_Calosphaeriales
Cligniaria_Coniochaetales
Cinnumera_Chaetosphaeriales
Gtetrasperma_Sordariales
Cbulbillosum_Sordariales
Opiliferum_Ophiostomatales
Gclavigera_Ophiostomatales
Lminuta_Diaporthales
Cpopulea_Diaporthales
Cchrysosperma_Diaporthales
Dampelina_Diaporthales
Clustricola_Diaporthales
Mphaseolina_Botryosphaeriales
Pcitrichinaensis_Botryosphaeriales
Sproteae_Botryosphaeriales
Mmicroscopicum_Microthyriales
Abenhamiae_Onygenales
Psteckii_Eurotiales
Aaculeatus_Eurotiales
Arestrictus_Eurotiales
Aterreus_Eurotiales
Aarachidicola_Eurotiales
Acaelatus_Eurotiales
Tguianense_Xylonales
Teluteriae_Trypetheliales
Patrata_Patellariales
Skochii_Symbiotaphrinales
Uflorida_Lecanorales
Xparietina_Teloschistales
Arichmondensis_Capnodiales
Czeae-maydis_Capnodiales
Zpseudotritici_Capnodiales
Lmytilinum_Mytilinidiales
Lpalustris_Mytilinidiales
Bcircinans_Pleosporales
Dexigua_Pleosporales
Lmaculans_Pleosporales
Ffenestrata_Pleosporales
Edeformans_Rhytismatales
Lnitens_Rhytismatales
Pelatina_Triblidiales
Cstrobi_Rhytismatales
Sflavida_Rhytismatales
Binquinans_Helotiales
Rericae_Helotiales
Clongipes_Helotiales
Mbicolor_Helotiales
Amacrosclerotiorum_Helotiales
Pscopiformis_Helotiales
Ljuncicola_Helotiales
Lmacrosporus_Helotiales
Glozoyensis_Helotiales
Zvarium_Lulworthiales
Enecator_Erysiphales
Asarcoides_Helotiales
Rfirma_Helotiales
Bhyacinthi_Helotiales
Bporri_Helotiales
Bcalthae_Helotiales
Bpseudocinerea_Helotiales
Bcinerea_Helotiales
Bpaeoniae_Helotiales
Bconvoluta_Helotiales
Btulipae_Helotiales
Bnarcissicola_Helotiales
Belliptica_Helotiales
Bmedusae_Helotiales
Bgalanthina_Helotiales
taxa
20
40
60
80
100
0
1
2
3
4
5
6
7
8
branch length

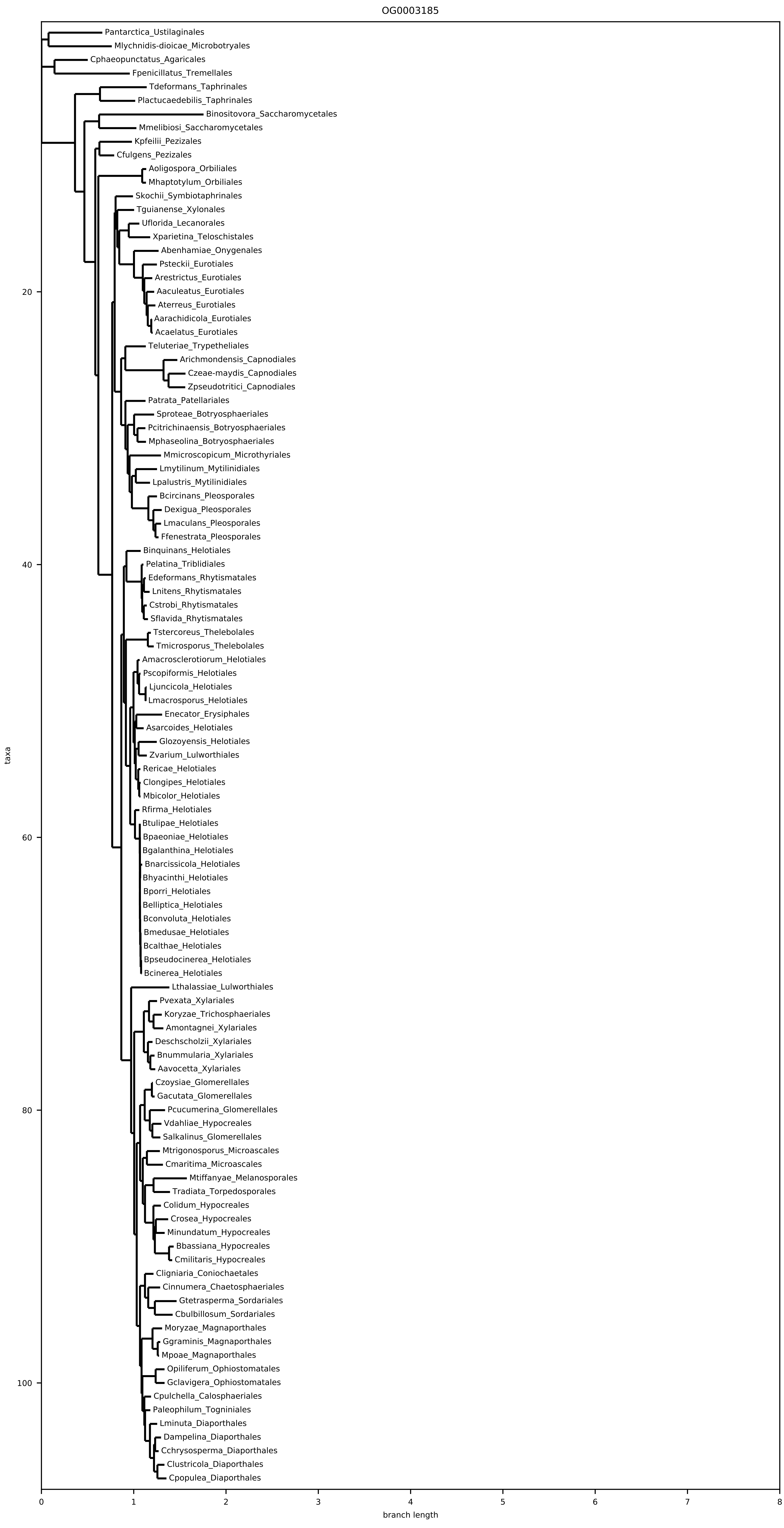

OG0003185
Pantarctica_Ustilaginales
Mlychnidis-dioicae_Microbotryales
Cphaeopunctatus_Agaricales
Fpenicillatus_Tremellales
Tdeformans_Taphrinales
Plactucaedebilis_Taphrinales
Binositovora_Saccharomycetales
Mmelibiosi_Saccharomycetales
Kpfeilii_Pezizales
Cfulgens_Pezizales
Aoligospora_Orbiliales
Mhaptotylum_Orbiliales
Skochii_Symbiotaphrinales
Tguianense_Xylonales
Uflorida_Lecanorales
Xparietina_Teloschistales
Abenhamiae_Onygenales
Psteckii_Eurotiales
Arestrictus_Eurotiales
Aaculeatus_Eurotiales
Aterreus_Eurotiales
Aarachidicola_Eurotiales
Acaelatus_Eurotiales
Teluteriae_Trypetheliales
Arichmondensis_Capnodiales
Czeae-maydis_Capnodiales
Zpseudotritici_Capnodiales
Patrata_Patellariales
Sproteae_Botryosphaeriales
Pcitrichinaensis_Botryosphaeriales
Mphaseolina_Botryosphaeriales
Mmicroscopicum_Microthyriales
Lmytilinum_Mytilinidiales
Lpalustris_Mytilinidiales
Bcircinans_Pleosporales
Dexigua_Pleosporales
Lmaculans_Pleosporales
Ffenestrata_Pleosporales
Binquinans_Helotiales
Pelatina_Triblidiales
Edeformans_Rhytismatales
Lnitens_Rhytismatales
Cstrobi_Rhytismatales
Sflavida_Rhytismatales
Tstercoreus_Thelebolales
Tmicrosporus_Thelebolales
Amacrosclerotiorum_Helotiales
Pscopiformis_Helotiales
Ljuncicola_Helotiales
Lmacrosporus_Helotiales
Enecator_Erysiphales
Asarcoides_Helotiales
Glozoyensis_Helotiales
Zvarium_Lulworthiales
Rericae_Helotiales
Clongipes_Helotiales
Mbicolor_Helotiales
Rfirma_Helotiales
Btulipae_Helotiales
Bpaeoniae_Helotiales
Bgalanthina_Helotiales
Bnarcissicola_Helotiales
Bhyacinthi_Helotiales
Bporri_Helotiales
Belliptica_Helotiales
Bconvoluta_Helotiales
Bmedusae_Helotiales
Bcalthae_Helotiales
Bpseudocinerea_Helotiales
Bcinerea_Helotiales
Lthalassiae_Lulworthiales
Pvexata_Xylariales
Koryzae_Trichosphaeriales
Amontagnei_Xylariales
Deschscholzii_Xylariales
Bnummularia_Xylariales
Aavocetta_Xylariales
Czoysiae_Glomerellales
Gacutata_Glomerellales
Pcucumerina_Glomerellales
Vdahliae_Hypocreales
Salkalinus_Glomerellales
Mtrigonosporus_Microascales
Cmaritima_Microascales
Mtiffanyae_Melanosporales
Tradiata_Torpedosporales
Colidum_Hypocreales
Crosea_Hypocreales
Minundatum_Hypocreales
Bbassiana_Hypocreales
Cmilitaris_Hypocreales
Cligniaria_Coniochaetales
Cinnumera_Chaetosphaeriales
Gtetrasperma_Sordariales
Cbulbillosum_Sordariales
Moryzae_Magnaporthales
Ggraminis_Magnaporthales
Mpoae_Magnaporthales
Opiliferum_Ophiostomatales
Gclavigera_Ophiostomatales
Cpulchella_Calosphaeriales
Paleophilum_Togniniales
Lminuta_Diaporthales
Dampelina_Diaporthales
Cchrysosperma_Diaporthales
Clustricola_Diaporthales
Cpopulea_Diaporthales
taxa
20
40
60
80
100
0
1
2
3
4
5
6
7
8
branch length

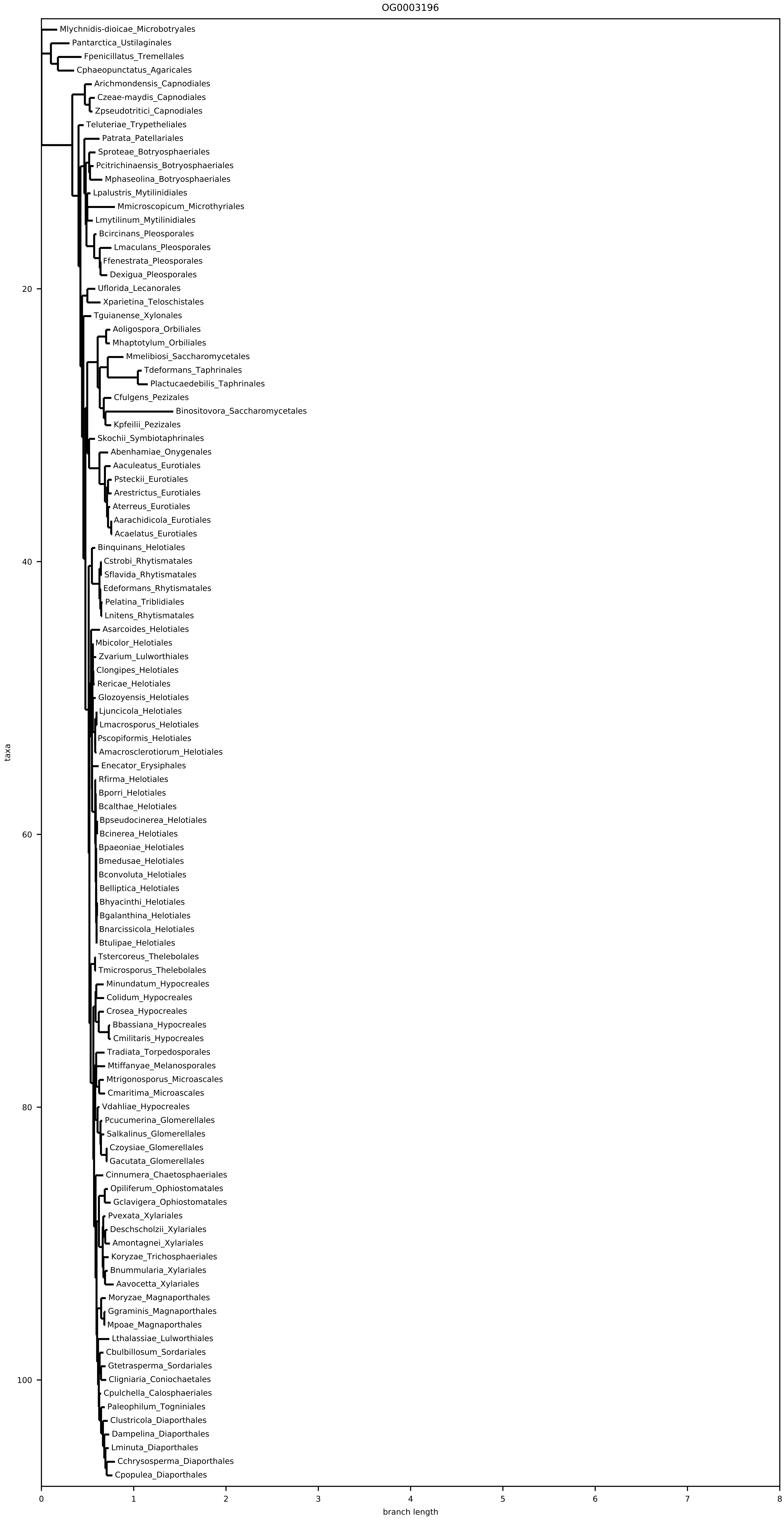
OG0003196
Mlychnidis-dioicae_Microbotryales
Pantarctica_Ustilaginales
Fpenicillatus_Tremellales
Cphaeopunctatus_Agaricales
Arichmondensis_Capnodiales
Czeae-maydis_Capnodiales
Zpseudotritici_Capnodiales
Teluteriae_Trypetheliales
Patrata_Patellariales
Sproteae_Botryosphaeriales
Pcitrichinaensis_Botryosphaeriales
Mphaseolina_Botryosphaeriales
Lpalustris_Mytilinidiales
Mmicroscopicum_Microthyriales
Lmytilinum_Mytilinidiales
Bcircinans_Pleosporales
Lmaculans_Pleosporales
Ffenestrata_Pleosporales
Dexigua_Pleosporales
Uflorida_Lecanorales
Xparietina_Teloschistales
Tguianense_Xylonales
Aoligospora_Orbiliales
Mhaptotylum_Orbiliales
Mmelibiosi_Saccharomycetales
Tdeformans_Taphrinales
Plactucaedebilis_Taphrinales
Cfulgens_Pezizales
Binositovora_Saccharomycetales
Kpfeilii_Pezizales
Skochii_Symbiotaphrinales
Abenhamiae_Onygenales
Aaculeatus_Eurotiales
Psteckii_Eurotiales
Arestrictus_Eurotiales
Aterreus_Eurotiales
Aarachidicola_Eurotiales
Acaelatus_Eurotiales
Binquinans_Helotiales
Cstrobi_Rhytismatales
Sflavida_Rhytismatales
Edeformans_Rhytismatales
Pelatina_Triblidiales
Lnitens_Rhytismatales
Asarcoides_Helotiales
Mbicolor_Helotiales
Zvarium_Lulworthiales
Clongipes_Helotiales
Rericae_Helotiales
Glozoyensis_Helotiales
Ljuncicola_Helotiales
Lmacrosporus_Helotiales
Pscopiformis_Helotiales
Amacrosclerotiorum_Helotiales
Enecator_Erysiphales
Rfirma_Helotiales
Bporri_Helotiales
Bcalthae_Helotiales
Bpseudocinerea_Helotiales
Bcinerea_Helotiales
Bpaeoniae_Helotiales
Bmedusae_Helotiales
Bconvoluta_Helotiales
Belliptica_Helotiales
Bhyacinthi_Helotiales
Bgalanthina_Helotiales
Bnarcissicola_Helotiales
Btulipae_Helotiales
Tstercoreus_Thelebolales
Tmicrosporus_Thelebolales
Minundatum_Hypocreales
Colidum_Hypocreales
Crosea_Hypocreales
Bbassiana_Hypocreales
Cmilitaris_Hypocreales
Tradiata_Torpedosporales
Mtiffanyae_Melanosporales
Mtrigonosporus_Microascales
Cmaritima_Microascales
Vdahliae_Hypocreales
Pcucumerina_Glomerellales
Salkalinus_Glomerellales
Czoysiae_Glomerellales
Gacutata_Glomerellales
Cinnumera_Chaetosphaeriales
Opiliferum_Ophiostomatales
Gclavigera_Ophiostomatales
Pvexata_Xylariales
Deschscholzii_Xylariales
Amontagnei_Xylariales
Koryzae_Trichosphaeriales
Bnummularia_Xylariales
Aavocetta_Xylariales
Moryzae_Magnaporthales
Ggraminis_Magnaporthales
Mpoae_Magnaporthales
Lthalassiae_Lulworthiales
Cbulbillosum_Sordariales
Gtetrasperma_Sordariales
Cligniaria_Coniochaetales
Cpulchella_Calosphaeriales
Paleophilum_Togniniales
Clustricola_Diaporthales
Dampelina_Diaporthales
Lminuta_Diaporthales
Cchrysosperma_Diaporthales
Cpopulea_Diaporthales
taxa
20
40
60
80
100
0
1
2
3
4
5
6
7
8
branch length

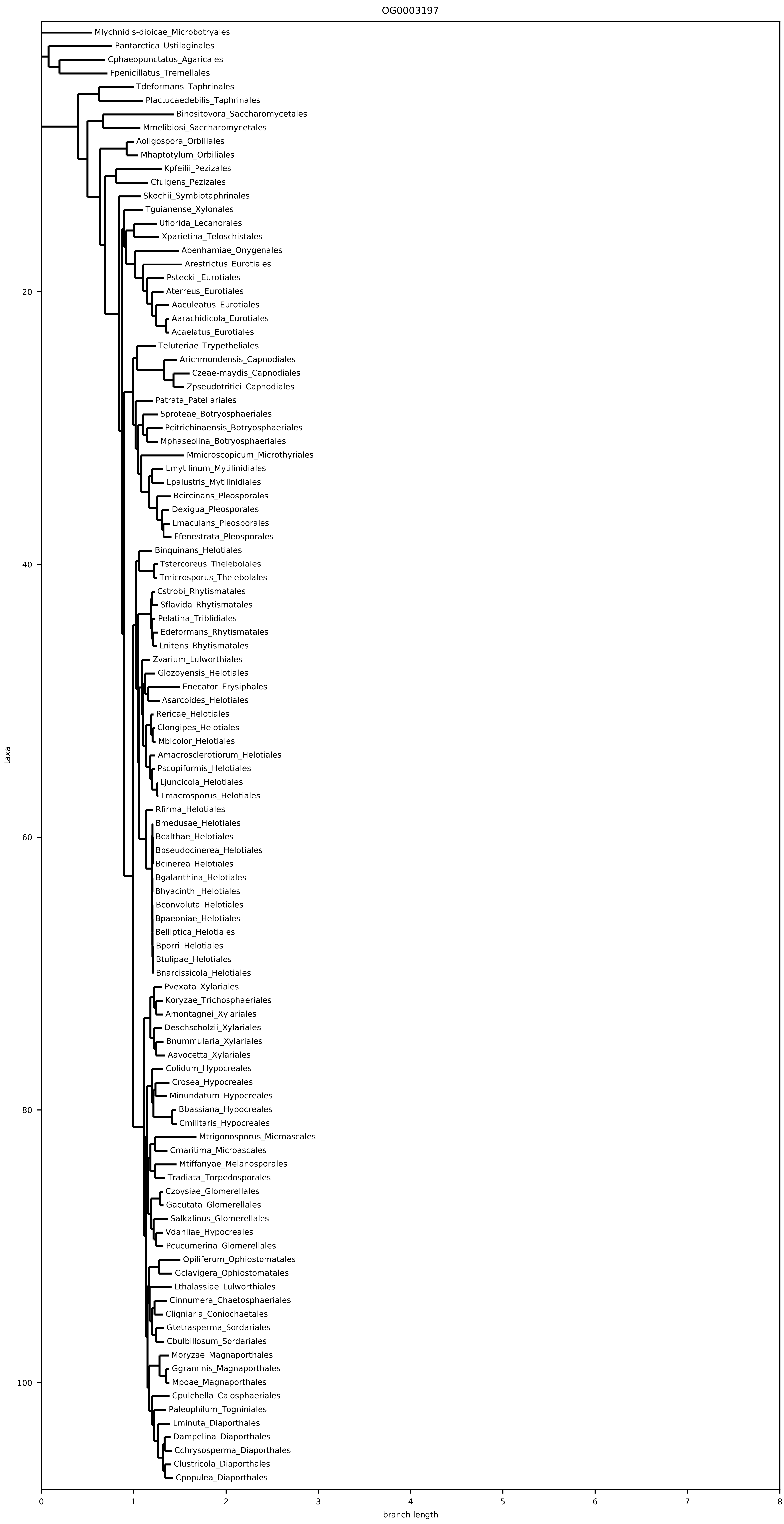

OG0003197
Mlychnidis-dioicae_Microbotryales
Pantarctica_Ustilaginales
Cphaeopunctatus_Agaricales
Fpenicillatus_Tremellales
Tdeformans_Taphrinales
Plactucaedebilis_Taphrinales
Binositovora_Saccharomycetales
Mmelibiosi_Saccharomycetales
Aoligospora_Orbiliales
Mhaptotylum_Orbiliales
Kpfeilii_Pezizales
Cfulgens_Pezizales
Skochii_Symbiotaphrinales
Tguianense_Xylonales
Uflorida_Lecanorales
Xparietina_Teloschistales
Abenhamiae_Onygenales
Arestrictus_Eurotiales
Psteckii_Eurotiales
Aterreus_Eurotiales
Aaculeatus_Eurotiales
Aarachidicola_Eurotiales
Acaelatus_Eurotiales
Teluteriae_Trypetheliales
Arichmondensis_Capnodiales
Czeae-maydis_Capnodiales
Zpseudotritici_Capnodiales
Patrata_Patellariales
Sproteae_Botryosphaeriales
Pcitrichinaensis_Botryosphaeriales
Mphaseolina_Botryosphaeriales
Mmicroscopicum_Microthyriales
Lmytilinum_Mytilinidiales
Lpalustris_Mytilinidiales
Bcircinans_Pleosporales
Dexigua_Pleosporales
Lmaculans_Pleosporales
Ffenestrata_Pleosporales
Binquinans_Helotiales
Tstercoreus_Thelebolales
Tmicrosporus_Thelebolales
Cstrobi_Rhytismatales
Sflavida_Rhytismatales
Pelatina_Triblidiales
Edeformans_Rhytismatales
Lnitens_Rhytismatales
Zvarium_Lulworthiales
Glozoyensis_Helotiales
Enecator_Erysiphales
Asarcoides_Helotiales
Rericae_Helotiales
Clongipes_Helotiales
Mbicolor_Helotiales
Amacrosclerotiorum_Helotiales
Pscopiformis_Helotiales
Ljuncicola_Helotiales
Lmacrosporus_Helotiales
Rfirma_Helotiales
Bmedusae_Helotiales
Bcalthae_Helotiales
Bpseudocinerea_Helotiales
Bcinerea_Helotiales
Bgalanthina_Helotiales
Bhyacinthi_Helotiales
Bconvoluta_Helotiales
Bpaeoniae_Helotiales
Belliptica_Helotiales
Bporri_Helotiales
Btulipae_Helotiales
Bnarcissicola_Helotiales
Pvexata_Xylariales
Koryzae_Trichosphaeriales
Amontagnei_Xylariales
Deschscholzii_Xylariales
Bnummularia_Xylariales
Aavocetta_Xylariales
Colidum_Hypocreales
Crosea_Hypocreales
Minundatum_Hypocreales
Bbassiana_Hypocreales
Cmilitaris_Hypocreales
Mtrigonosporus_Microascales
Cmaritima_Microascales
Mtiffanyae_Melanosporales
Tradiata_Torpedosporales
Czoysiae_Glomerellales
Gacutata_Glomerellales
Salkalinus_Glomerellales
Vdahliae_Hypocreales
Pcucumerina_Glomerellales
Opiliferum_Ophiostomatales
Gclavigera_Ophiostomatales
Lthalassiae_Lulworthiales
Cinnumera_Chaetosphaeriales
Cligniaria_Coniochaetales
Gtetrasperma_Sordariales
Cbulbillosum_Sordariales
Moryzae_Magnaporthales
Ggraminis_Magnaporthales
Mpoae_Magnaporthales
Cpulchella_Calosphaeriales
Paleophilum_Togniniales
Lminuta_Diaporthales
Dampelina_Diaporthales
Cchrysosperma_Diaporthales
Clustricola_Diaporthales
Cpopulea_Diaporthales
taxa
20
40
60
80
100
0
1
2
3
4
5
6
7
8
branch length

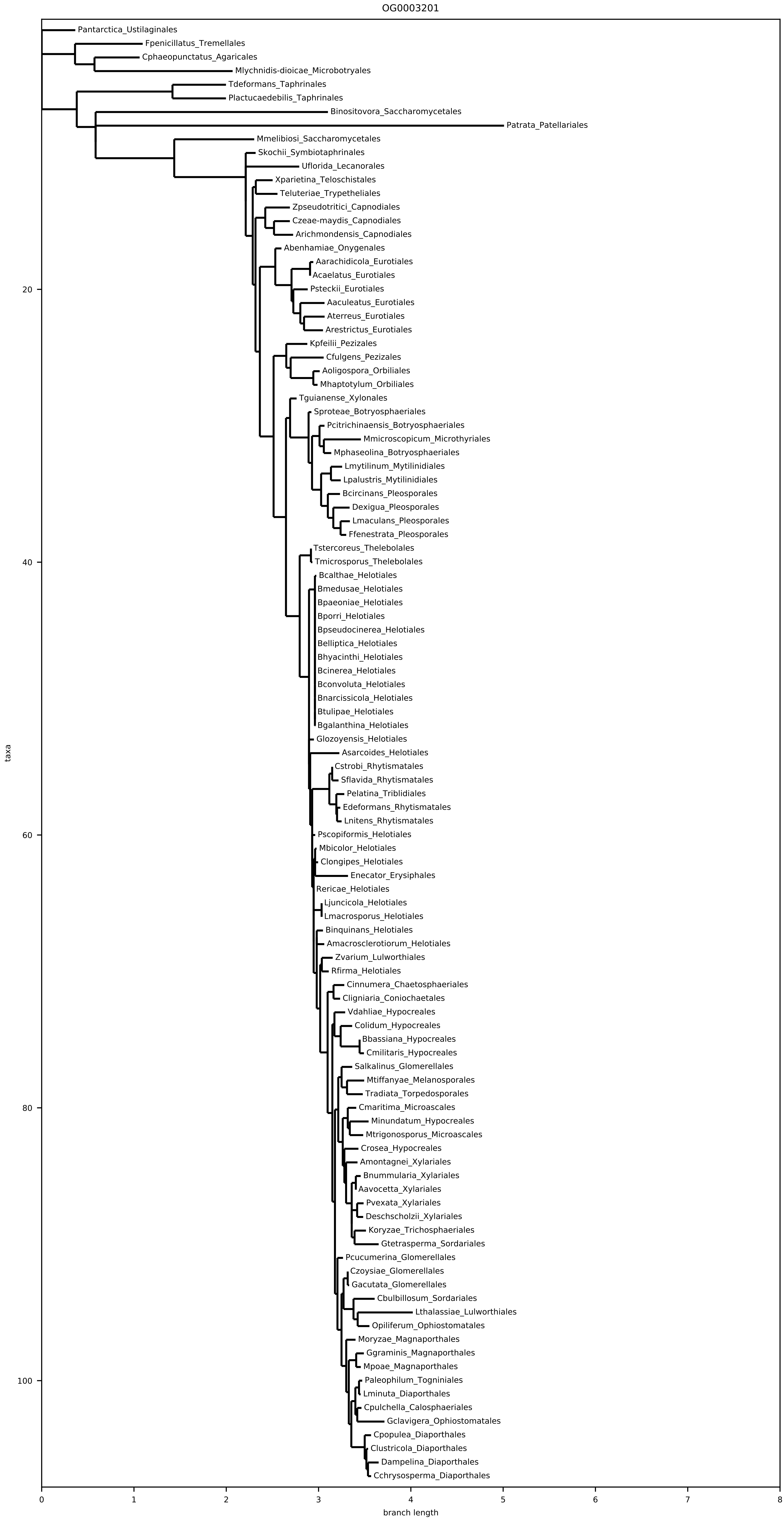

OG0003201
Pantarctica_Ustilaginales
Fpenicillatus_Tremellales
Cphaeopunctatus_Agaricales
Mlychnidis-dioicae_Microbotryales
Tdeformans_Taphrinales
Plactucaedebilis_Taphrinales
Binositovora_Saccharomycetales
Patrata_Patellariales
Mmelibiosi_Saccharomycetales
Skochii_Symbiotaphrinales
Uflorida_Lecanorales
Xparietina_Teloschistales
Teluteriae_Trypetheliales
Zpseudotritici_Capnodiales
Czeae-maydis_Capnodiales
Arichmondensis_Capnodiales
Abenhamiae_Onygenales
Aarachidicola_Eurotiales
Acaelatus_Eurotiales
Psteckii_Eurotiales
Aaculeatus_Eurotiales
Aterreus_Eurotiales
Arestrictus_Eurotiales
Kpfeilii_Pezizales
Cfulgens_Pezizales
Aoligospora_Orbiliales
Mhaptotylum_Orbiliales
Tguianense_Xylonales
Sproteae_Botryosphaeriales
Pcitrichinaensis_Botryosphaeriales
Mmicroscopicum_Microthyriales
Mphaseolina_Botryosphaeriales
Lmytilinum_Mytilinidiales
Lpalustris_Mytilinidiales
Bcircinans_Pleosporales
Dexigua_Pleosporales
Lmaculans_Pleosporales
Ffenestrata_Pleosporales
Tstercoreus_Thelebolales
Tmicrosporus_Thelebolales
Bcalthae_Helotiales
Bmedusae_Helotiales
Bpaeoniae_Helotiales
Bporri_Helotiales
Bpseudocinerea_Helotiales
Belliptica_Helotiales
Bhyacinthi_Helotiales
Bcinerea_Helotiales
Bconvoluta_Helotiales
Bnarcissicola_Helotiales
Btulipae_Helotiales
Bgalanthina_Helotiales
Glozoyensis_Helotiales
Asarcoides_Helotiales
Cstrobi_Rhytismatales
Sflavida_Rhytismatales
Pelatina_Triblidiales
Edeformans_Rhytismatales
Lnitens_Rhytismatales
Pscopiformis_Helotiales
Mbicolor_Helotiales
Clongipes_Helotiales
Enecator_Erysiphales
Rericae_Helotiales
Ljuncicola_Helotiales
Lmacrosporus_Helotiales
Binquinans_Helotiales
Amacrosclerotiorum_Helotiales
Zvarium_Lulworthiales
Rfirma_Helotiales
Cinnumera_Chaetosphaeriales
Cligniaria_Coniochaetales
Vdahliae_Hypocreales
Colidum_Hypocreales
Bbassiana_Hypocreales
Cmilitaris_Hypocreales
Salkalinus_Glomerellales
Mtiffanyae_Melanosporales
Tradiata_Torpedosporales
Cmaritima_Microascales
Minundatum_Hypocreales
Mtrigonosporus_Microascales
Crosea_Hypocreales
Amontagnei_Xylariales
Bnummularia_Xylariales
Aavocetta_Xylariales
Pvexata_Xylariales
Deschscholzii_Xylariales
Koryzae_Trichosphaeriales
Gtetrasperma_Sordariales
Pcucumerina_Glomerellales
Czoysiae_Glomerellales
Gacutata_Glomerellales
Cbulbillosum_Sordariales
Lthalassiae_Lulworthiales
Opiliferum_Ophiostomatales
Moryzae_Magnaporthales
Ggraminis_Magnaporthales
Mpoae_Magnaporthales
Paleophilum_Togniniales
Lminuta_Diaporthales
Cpulchella_Calosphaeriales
Gclavigera_Ophiostomatales
Cpopulea_Diaporthales
Clustricola_Diaporthales
Dampelina_Diaporthales
Cchrysosperma_Diaporthales
taxa
20
40
60
80
100
0
1
2
3
4
5
6
7
8
branch length

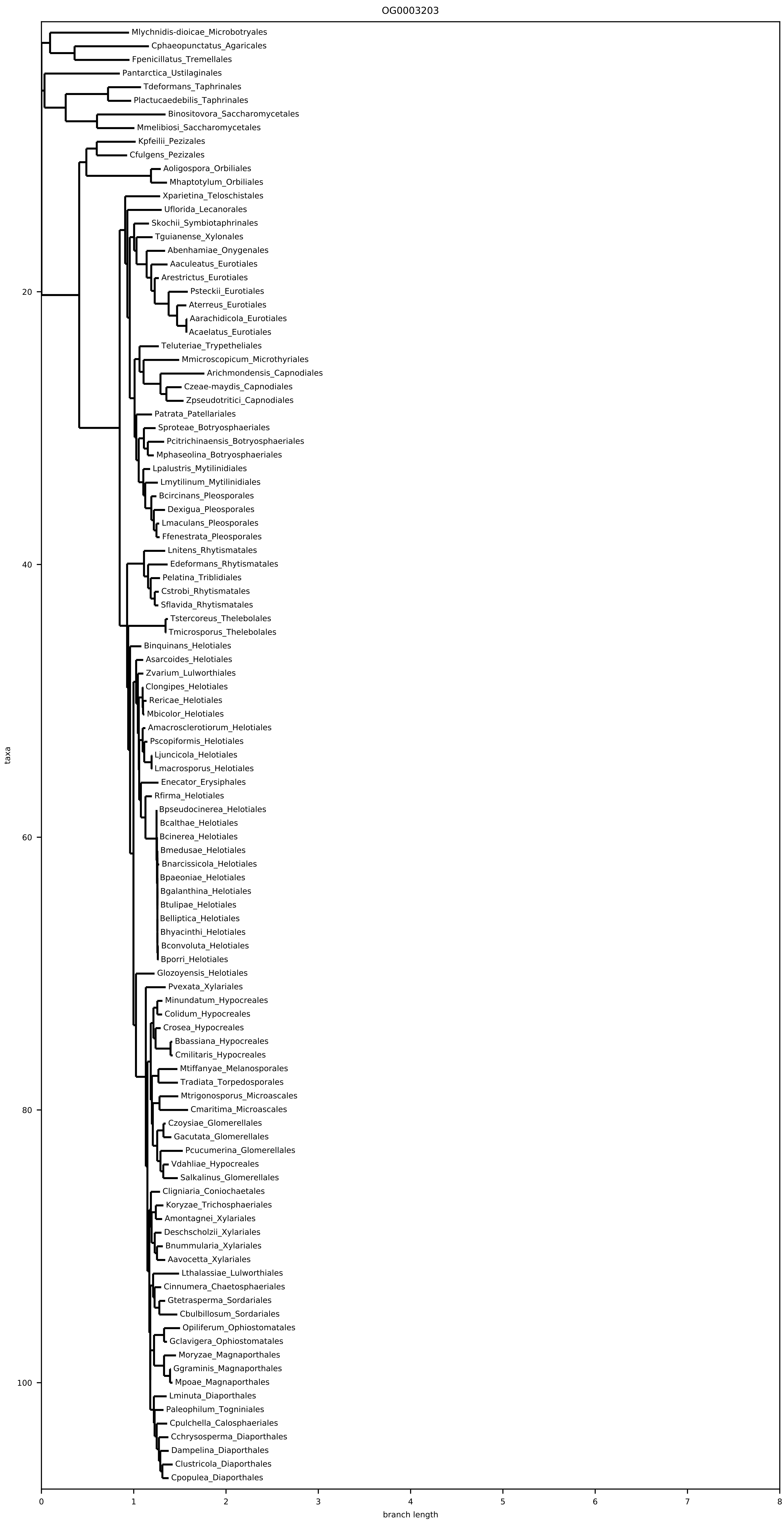
OG0003203
Mlychnidis-dioicae_Microbotryales
Cphaeopunctatus_Agaricales
Fpenicillatus_Tremellales
Pantarctica_Ustilaginales
Tdeformans_Taphrinales
Plactucaedebilis_Taphrinales
Binositovora_Saccharomycetales
Mmelibiosi_Saccharomycetales
Kpfeilii_Pezizales
Cfulgens_Pezizales
Aoligospora_Orbiliales
Mhaptotylum_Orbiliales
Xparietina_Teloschistales
Uflorida_Lecanorales
Skochii_Symbiotaphrinales
Tguianense_Xylonales
Abenhamiae_Onygenales
Aaculeatus_Eurotiales
Arestrictus_Eurotiales
Psteckii_Eurotiales
Aterreus_Eurotiales
Aarachidicola_Eurotiales
Acaelatus_Eurotiales
Teluteriae_Trypetheliales
Mmicroscopicum_Microthyriales
Arichmondensis_Capnodiales
Czeae-maydis_Capnodiales
Zpseudotritici_Capnodiales
Patrata_Patellariales
Sproteae_Botryosphaeriales
Pcitrichinaensis_Botryosphaeriales
Mphaseolina_Botryosphaeriales
Lpalustris_Mytilinidiales
Lmytilinum_Mytilinidiales
Bcircinans_Pleosporales
Dexigua_Pleosporales
Lmaculans_Pleosporales
Ffenestrata_Pleosporales
Lnitens_Rhytismatales
Edeformans_Rhytismatales
Pelatina_Triblidiales
Cstrobi_Rhytismatales
Sflavida_Rhytismatales
Tstercoreus_Thelebolales
Tmicrosporus_Thelebolales
Binquinans_Helotiales
Asarcoides_Helotiales
Zvarium_Lulworthiales
Clongipes_Helotiales
Rericae_Helotiales
Mbicolor_Helotiales
Amacrosclerotiorum_Helotiales
Pscopiformis_Helotiales
Ljuncicola_Helotiales
Lmacrosporus_Helotiales
Enecator_Erysiphales
Rfirma_Helotiales
Bpseudocinerea_Helotiales
Bcalthae_Helotiales
Bcinerea_Helotiales
Bmedusae_Helotiales
Bnarcissicola_Helotiales
Bpaeoniae_Helotiales
Bgalanthina_Helotiales
Btulipae_Helotiales
Belliptica_Helotiales
Bhyacinthi_Helotiales
Bconvoluta_Helotiales
Bporri_Helotiales
Glozoyensis_Helotiales
Pvexata_Xylariales
Minundatum_Hypocreales
Colidum_Hypocreales
Crosea_Hypocreales
Bbassiana_Hypocreales
Cmilitaris_Hypocreales
Mtiffanyae_Melanosporales
Tradiata_Torpedosporales
Mtrigonosporus_Microascales
Cmaritima_Microascales
Czoysiae_Glomerellales
Gacutata_Glomerellales
Pcucumerina_Glomerellales
Vdahliae_Hypocreales
Salkalinus_Glomerellales
Cligniaria_Coniochaetales
Koryzae_Trichosphaeriales
Amontagnei_Xylariales
Deschscholzii_Xylariales
Bnummularia_Xylariales
Aavocetta_Xylariales
Lthalassiae_Lulworthiales
Cinnumera_Chaetosphaeriales
Gtetrasperma_Sordariales
Cbulbillosum_Sordariales
Opiliferum_Ophiostomatales
Gclavigera_Ophiostomatales
Moryzae_Magnaporthales
Ggraminis_Magnaporthales
Mpoae_Magnaporthales
Lminuta_Diaporthales
Paleophilum_Togniniales
Cpulchella_Calosphaeriales
Cchrysosperma_Diaporthales
Dampelina_Diaporthales
Clustricola_Diaporthales
Cpopulea_Diaporthales
taxa
20
40
60
80
100
branch length
0
1
2
3
4
5
6
7
8

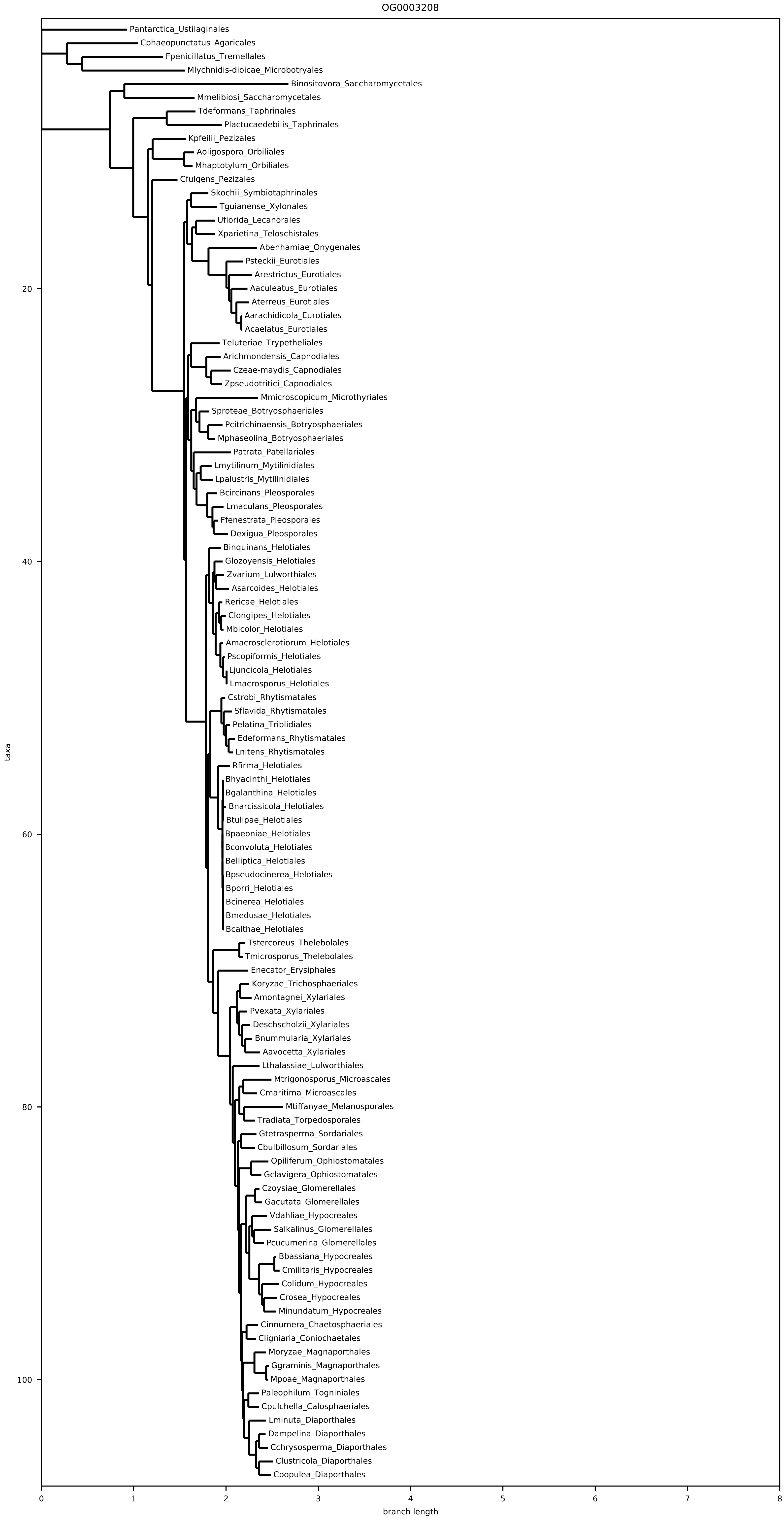
OG0003208
Pantarctica_Ustilaginales
Cphaeopunctatus_Agaricales
Fpenicillatus_Tremellales
Mlychnidis-dioicae_Microbotryales
Binositovora_Saccharomycetales
Mmelibiosi_Saccharomycetales
Tdeformans_Taphrinales
Plactucaedebilis_Taphrinales
Kpfeilii_Pezizales
Aoligospora_Orbiliales
Mhaptotylum_Orbiliales
Cfulgens_Pezizales
Skochii_Symbiotaphrinales
Tguianense_Xylonales
Uflorida_Lecanorales
Xparietina_Teloschistales
Abenhamiae_Onygenales
Psteckii_Eurotiales
Arestrictus_Eurotiales
Aaculeatus_Eurotiales
Aterreus_Eurotiales
Aarachidicola_Eurotiales
Acaelatus_Eurotiales
Teluteriae_Trypetheliales
Arichmondensis_Capnodiales
Czeae-maydis_Capnodiales
Zpseudotritici_Capnodiales
Mmicroscopicum_Microthyriales
Sproteae_Botryosphaeriales
Pcitrichinaensis_Botryosphaeriales
Mphaseolina_Botryosphaeriales
Patrata_Patellariales
Lmytilinum_Mytilinidiales
Lpalustris_Mytilinidiales
Bcircinans_Pleosporales
Lmaculans_Pleosporales
Ffenestrata_Pleosporales
Dexigua_Pleosporales
Binquinans_Helotiales
Glozoyensis_Helotiales
Zvarium_Lulworthiales
Asarcoides_Helotiales
Rericae_Helotiales
Clongipes_Helotiales
Mbicolor_Helotiales
Amacrosclerotiorum_Helotiales
Pscopiformis_Helotiales
Ljuncicola_Helotiales
Lmacrosporus_Helotiales
Cstrobi_Rhytismatales
Sflavida_Rhytismatales
Pelatina_Triblidiales
Edeformans_Rhytismatales
Lnitens_Rhytismatales
Rfirma_Helotiales
Bhyacinthi_Helotiales
Bgalanthina_Helotiales
Bnarcissicola_Helotiales
Btulipae_Helotiales
Bpaeoniae_Helotiales
Bconvoluta_Helotiales
Belliptica_Helotiales
Bpseudocinerea_Helotiales
Bporri_Helotiales
Bcinerea_Helotiales
Bmedusae_Helotiales
Bcalthae_Helotiales
Tstercoreus_Thelebolales
Tmicrosporus_Thelebolales
Enecator_Erysiphales
Koryzae_Trichosphaeriales
Amontagnei_Xylariales
Pvexata_Xylariales
Deschscholzii_Xylariales
Bnummularia_Xylariales
Aavocetta_Xylariales
Lthalassiae_Lulworthiales
Mtrigonosporus_Microascales
Cmaritima_Microascales
Mtiffanyae_Melanosporales
Tradiata_Torpedosporales
Gtetrasperma_Sordariales
Cbulbillosum_Sordariales
Opiliferum_Ophiostomatales
Gclavigera_Ophiostomatales
Czoysiae_Glomerellales
Gacutata_Glomerellales
Vdahliae_Hypocreales
Salkalinus_Glomerellales
Pcucumerina_Glomerellales
Bbassiana_Hypocreales
Cmilitaris_Hypocreales
Colidum_Hypocreales
Crosea_Hypocreales
Minundatum_Hypocreales
Cinnumera_Chaetosphaeriales
Cligniaria_Coniochaetales
Moryzae_Magnaporthales
Ggraminis_Magnaporthales
Mpoae_Magnaporthales
Paleophilum_Togniniales
Cpulchella_Calosphaeriales
Lminuta_Diaporthales
Dampelina_Diaporthales
Cchrysosperma_Diaporthales
Clustricola_Diaporthales
Cpopulea_Diaporthales
taxa
20
40
60
80
100
0
1
2
3
4
5
6
7
8
branch length

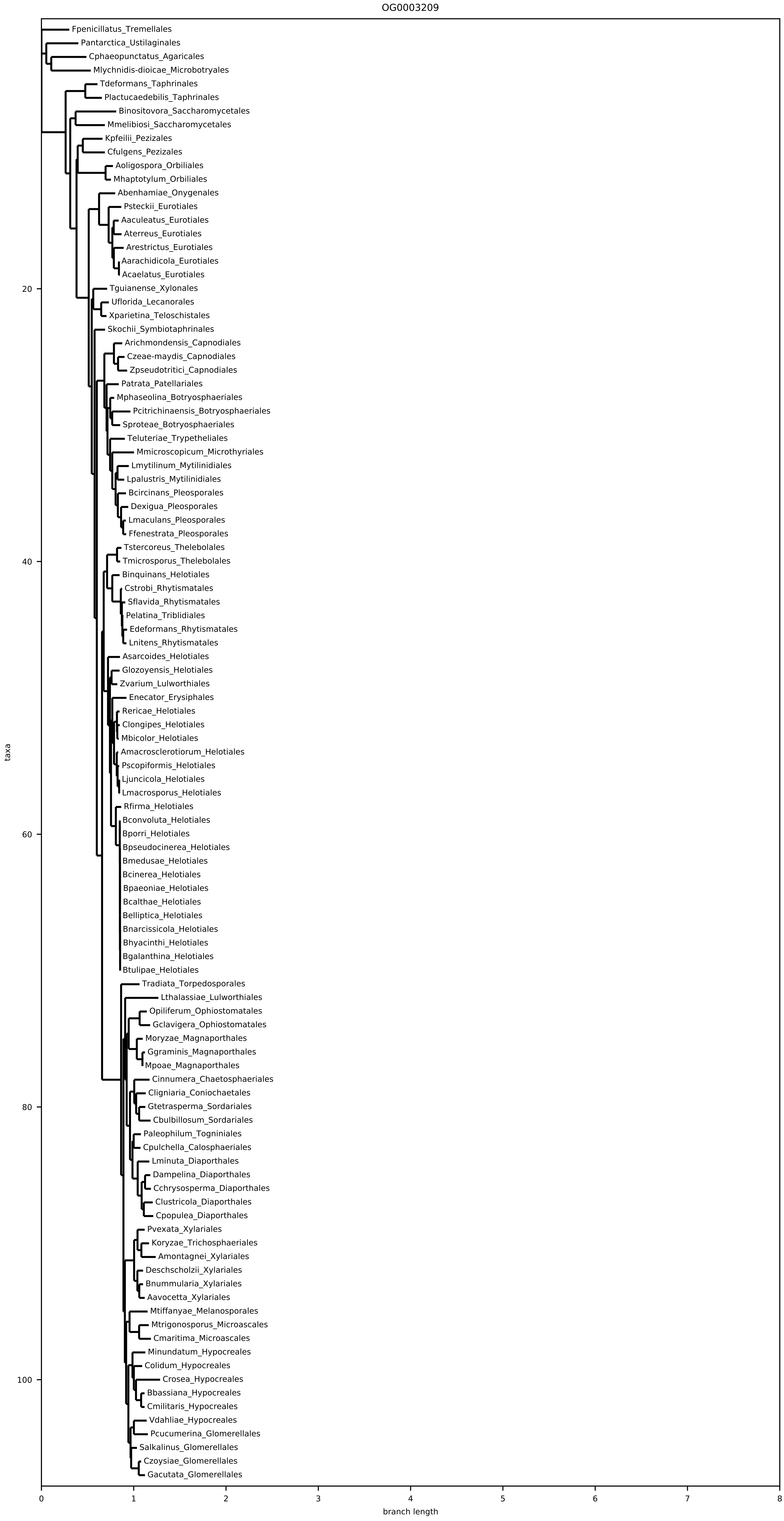

OG0003209
Fpenicillatus_Tremellales
Pantarctica_Ustilaginales
Cphaeopunctatus_Agaricales
Mlychnidis-dioicae_Microbotryales
Tdeformans_Taphrinales
Plactucaedebilis_Taphrinales
Binositovora_Saccharomycetales
Mmelibiosi_Saccharomycetales
Kpfeilii_Pezizales
Cfulgens_Pezizales
Aoligospora_Orbiliales
Mhaptotylum_Orbiliales
Abenhamiae_Onygenales
Psteckii_Eurotiales
Aaculeatus_Eurotiales
Aterreus_Eurotiales
Arestrictus_Eurotiales
Aarachidicola_Eurotiales
Acaelatus_Eurotiales
Tguianense_Xylonales
Uflorida_Lecanorales
Xparietina_Teloschistales
Skochii_Symbiotaphrinales
Arichmondensis_Capnodiales
Czeae-maydis_Capnodiales
Zpseudotritici_Capnodiales
Patrata_Patellariales
Mphaseolina_Botryosphaeriales
Pcitrichinaensis_Botryosphaeriales
Sproteae_Botryosphaeriales
Teluteriae_Trypetheliales
Mmicroscopicum_Microthyriales
Lmytilinum_Mytilinidiales
Lpalustris_Mytilinidiales
Bcircinans_Pleosporales
Dexigua_Pleosporales
Lmaculans_Pleosporales
Ffenestrata_Pleosporales
Tstercoreus_Thelebolales
Tmicrosporus_Thelebolales
Binquinans_Helotiales
Cstrobi_Rhytismatales
Sflavida_Rhytismatales
Pelatina_Triblidiales
Edeformans_Rhytismatales
Lnitens_Rhytismatales
Asarcoides_Helotiales
Glozoyensis_Helotiales
Zvarium_Lulworthiales
Enecator_Erysiphales
Rericae_Helotiales
Clongipes_Helotiales
Mbicolor_Helotiales
Amacrosclerotiorum_Helotiales
Pscopiformis_Helotiales
Ljuncicola_Helotiales
Lmacrosporus_Helotiales
Rfirma_Helotiales
Bconvoluta_Helotiales
Bporri_Helotiales
Bpseudocinerea_Helotiales
Bmedusae_Helotiales
Bcinerea_Helotiales
Bpaeoniae_Helotiales
Bcalthae_Helotiales
Belliptica_Helotiales
Bnarcissicola_Helotiales
Bhyacinthi_Helotiales
Bgalanthina_Helotiales
Btulipae_Helotiales
Tradiata_Torpedosporales
Lthalassiae_Lulworthiales
Opiliferum_Ophiostomatales
Gclavigera_Ophiostomatales
Moryzae_Magnaporthales
Ggraminis_Magnaporthales
Mpoae_Magnaporthales
Cinnumera_Chaetosphaeriales
Cligniaria_Coniochaetales
Gtetrasperma_Sordariales
Cbulbillosum_Sordariales
Paleophilum_Togniniales
Cpulchella_Calosphaeriales
Lminuta_Diaporthales
Dampelina_Diaporthales
Cchrysosperma_Diaporthales
Clustricola_Diaporthales
Cpopulea_Diaporthales
Pvexata_Xylariales
Koryzae_Trichosphaeriales
Amontagnei_Xylariales
Deschscholzii_Xylariales
Bnummularia_Xylariales
Aavocetta_Xylariales
Mtiffanyae_Melanosporales
Mtrigonosporus_Microascales
Cmaritima_Microascales
Minundatum_Hypocreales
Colidum_Hypocreales
Crosea_Hypocreales
Bbassiana_Hypocreales
Cmilitaris_Hypocreales
Vdahliae_Hypocreales
Pcucumerina_Glomerellales
Salkalinus_Glomerellales
Czoysiae_Glomerellales
Gacutata_Glomerellales
taxa
20
40
60
80
100
0
1
2
3
4
5
6
7
8
branch length

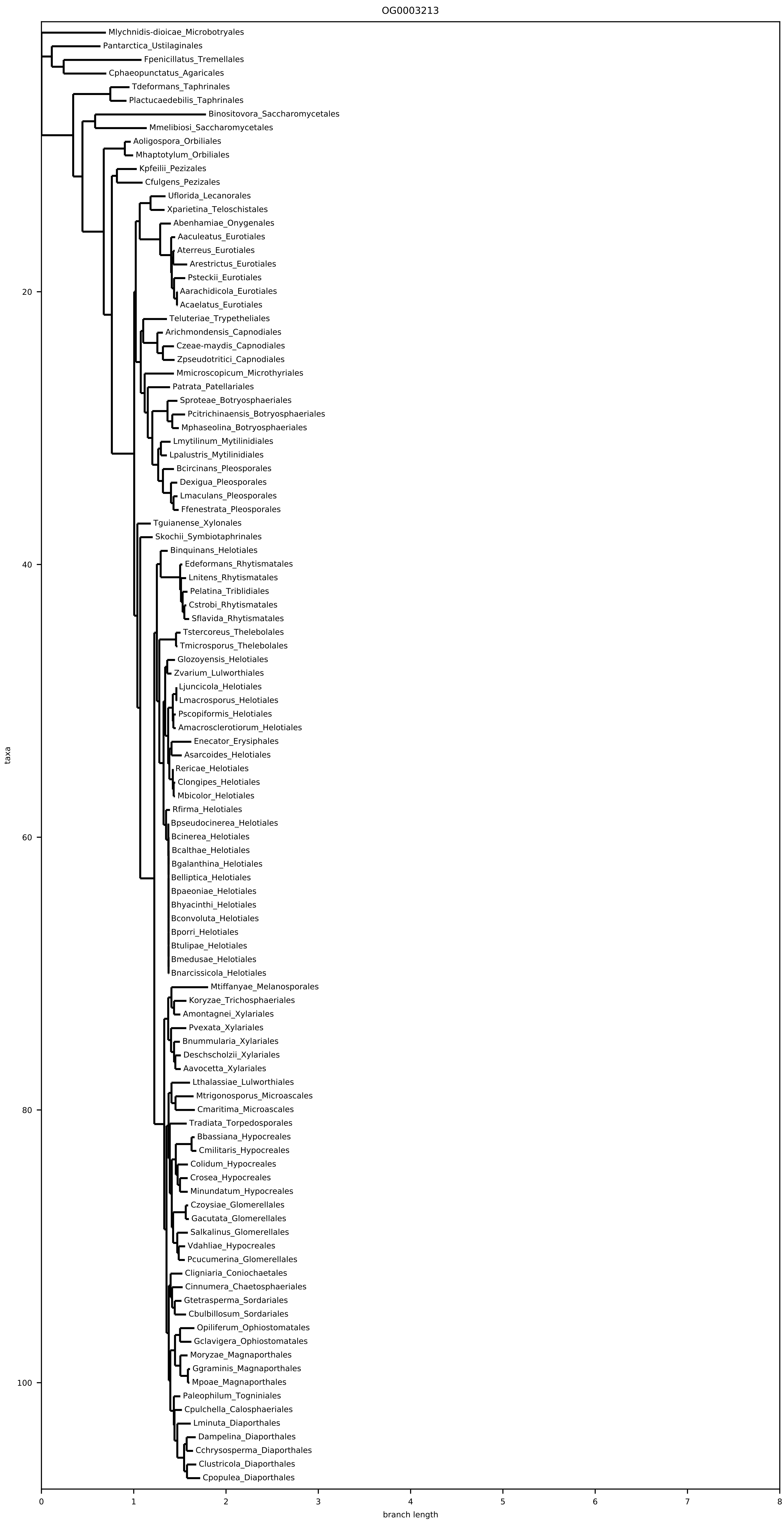

OG0003213
Mlychnidis-dioicae_Microbotryales
Pantarctica_Ustilaginales
Fpenicillatus_Tremellales
Cphaeopunctatus_Agaricales
Tdeformans_Taphrinales
Plactucaedebilis_Taphrinales
Binositovora_Saccharomycetales
Mmelibiosi_Saccharomycetales
Aoligospora_Orbiliales
Mhaptotylum_Orbiliales
Kpfeilii_Pezizales
Cfulgens_Pezizales
Uflorida_Lecanorales
Xparietina_Teloschistales
Abenhamiae_Onygenales
Aaculeatus_Eurotiales
Aterreus_Eurotiales
Arestrictus_Eurotiales
Psteckii_Eurotiales
Aarachidicola_Eurotiales
Acaelatus_Eurotiales
Teluteriae_Trypetheliales
Arichmondensis_Capnodiales
Czeae-maydis_Capnodiales
Zpseudotritici_Capnodiales
Mmicroscopicum_Microthyriales
Patrata_Patellariales
Sproteae_Botryosphaeriales
Pcitrichinaensis_Botryosphaeriales
Mphaseolina_Botryosphaeriales
Lmytilinum_Mytilinidiales
Lpalustris_Mytilinidiales
Bcircinans_Pleosporales
Dexigua_Pleosporales
Lmaculans_Pleosporales
Ffenestrata_Pleosporales
Tguianense_Xylonales
Skochii_Symbiotaphrinales
Binquinans_Helotiales
Edeformans_Rhytismatales
Lnitens_Rhytismatales
Pelatina_Triblidiales
Cstrobi_Rhytismatales
Sflavida_Rhytismatales
Tstercoreus_Thelebolales
Tmicrosporus_Thelebolales
Glozoyensis_Helotiales
Zvarium_Lulworthiales
Ljuncicola_Helotiales
Lmacrosporus_Helotiales
Pscopiformis_Helotiales
Amacrosclerotiorum_Helotiales
Enecator_Erysiphales
Asarcoides_Helotiales
Rericae_Helotiales
Clongipes_Helotiales
Mbicolor_Helotiales
Rfirma_Helotiales
Bpseudocinerea_Helotiales
Bcinerea_Helotiales
Bcalthae_Helotiales
Bgalanthina_Helotiales
Belliptica_Helotiales
Bpaeoniae_Helotiales
Bhyacinthi_Helotiales
Bconvoluta_Helotiales
Bporri_Helotiales
Btulipae_Helotiales
Bmedusae_Helotiales
Bnarcissicola_Helotiales
Mtiffanyae_Melanosporales
Koryzae_Trichosphaeriales
Amontagnei_Xylariales
Pvexata_Xylariales
Bnummularia_Xylariales
Deschscholzii_Xylariales
Aavocetta_Xylariales
Lthalassiae_Lulworthiales
Mtrigonosporus_Microascales
Cmaritima_Microascales
Tradiata_Torpedosporales
Bbassiana_Hypocreales
Cmilitaris_Hypocreales
Colidum_Hypocreales
Crosea_Hypocreales
Minundatum_Hypocreales
Czoysiae_Glomerellales
Gacutata_Glomerellales
Salkalinus_Glomerellales
Vdahliae_Hypocreales
Pcucumerina_Glomerellales
Cligniaria_Coniochaetales
Cinnumera_Chaetosphaeriales
Gtetrasperma_Sordariales
Cbulbillosum_Sordariales
Opiliferum_Ophiostomatales
Gclavigera_Ophiostomatales
Moryzae_Magnaporthales
Ggraminis_Magnaporthales
Mpoae_Magnaporthales
Paleophilum_Togniniales
Cpulchella_Calosphaeriales
Lminuta_Diaporthales
Dampelina_Diaporthales
Cchrysosperma_Diaporthales
Clustricola_Diaporthales
Cpopulea_Diaporthales
taxa
20
40
60
80
100
0
1
2
3
4
5
6
7
8
branch length

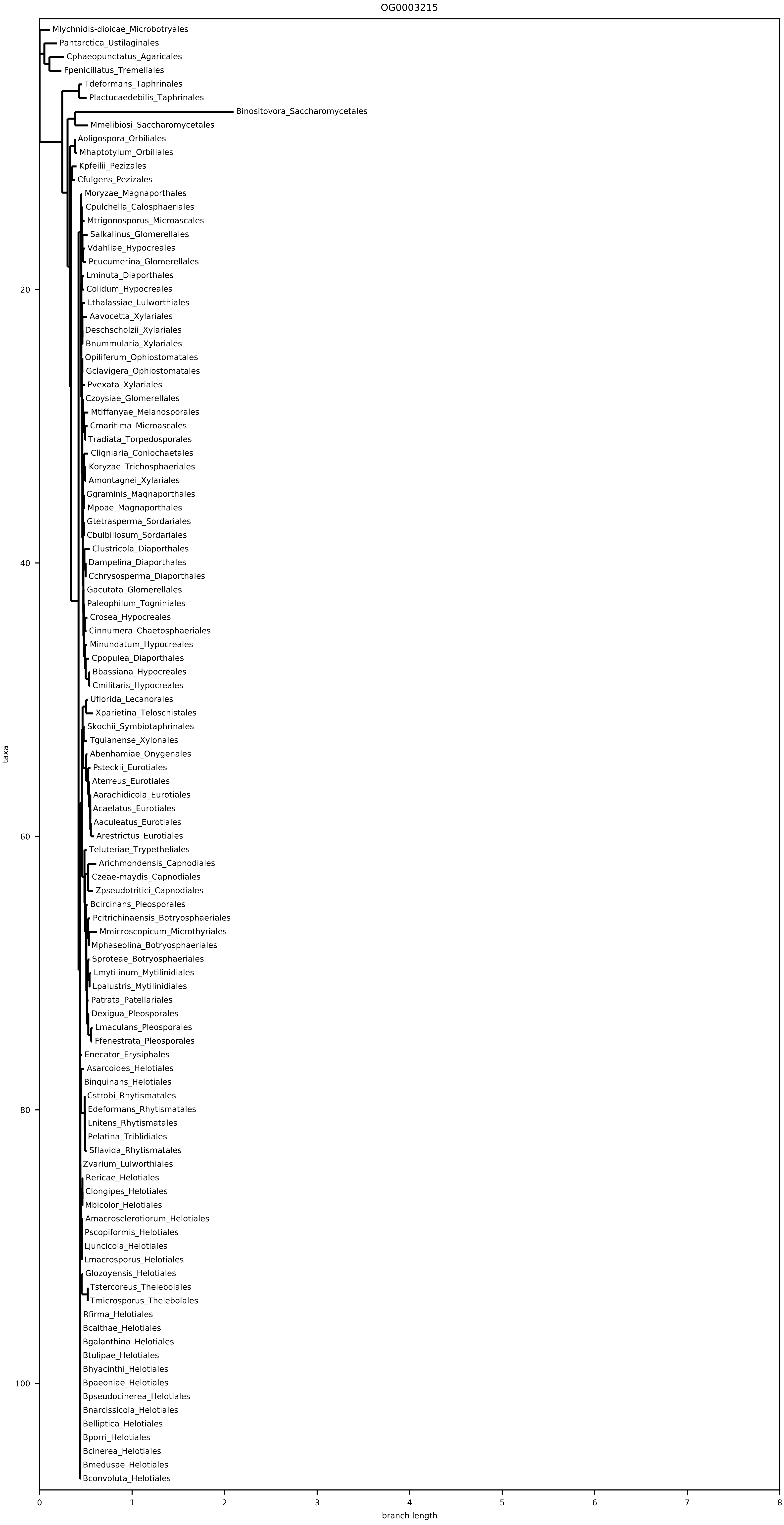
OG0003215
Mlychnidis-dioicae_Microbotryales
Pantarctica_Ustilaginales
Cphaeopunctatus_Agaricales
Fpenicillatus_Tremellales
Tdeformans_Taphrinales
Plactucaedebilis_Taphrinales
Binositovora_Saccharomycetales
Mmelibiosi_Saccharomycetales
Aoligospora_Orbiliales
Mhaptotylum_Orbiliales
Kpfeilii_Pezizales
Cfulgens_Pezizales
Moryzae_Magnaporthales
Cpulchella_Calosphaeriales
Mtrigonosporus_Microascales
Salkalinus_Glomerellales
Vdahliae_Hypocreales
Pcucumerina_Glomerellales
Lminuta_Diaporthales
Colidum_Hypocreales
Lthalassiae_Lulworthiales
Aavocetta_Xylariales
Deschscholzii_Xylariales
Bnummularia_Xylariales
Opiliferum_Ophiostomatales
Gclavigera_Ophiostomatales
Pvexata_Xylariales
Czoysiae_Glomerellales
Mtiffanyae_Melanosporales
Cmaritima_Microascales
Tradiata_Torpedosporales
Cligniaria_Coniochaetales
Koryzae_Trichosphaeriales
Amontagnei_Xylariales
Ggraminis_Magnaporthales
Mpoae_Magnaporthales
Gtetrasperma_Sordariales
Cbulbillosum_Sordariales
Clustricola_Diaporthales
Dampelina_Diaporthales
Cchrysosperma_Diaporthales
Gacutata_Glomerellales
Paleophilum_Togniniales
Crosea_Hypocreales
Cinnumera_Chaetosphaeriales
Minundatum_Hypocreales
Cpopulea_Diaporthales
Bbassiana_Hypocreales
Cmilitaris_Hypocreales
Uflorida_Lecanorales
Xparietina_Teloschistales
Skochii_Symbiotaphrinales
Tguianense_Xylonales
Abenhamiae_Onygenales
Psteckii_Eurotiales
Aterreus_Eurotiales
Aarachidicola_Eurotiales
Acaelatus_Eurotiales
Aaculeatus_Eurotiales
Arestrictus_Eurotiales
Teluteriae_Trypetheliales
Arichmondensis_Capnodiales
Czeae-maydis_Capnodiales
Zpseudotritici_Capnodiales
Bcircinans_Pleosporales
Pcitrichinaensis_Botryosphaeriales
Mmicroscopicum_Microthyriales
Mphaseolina_Botryosphaeriales
Sproteae_Botryosphaeriales
Lmytilinum_Mytilinidiales
Lpalustris_Mytilinidiales
Patrata_Patellariales
Dexigua_Pleosporales
Lmaculans_Pleosporales
Ffenestrata_Pleosporales
Enecator_Erysiphales
Asarcoides_Helotiales
Binquinans_Helotiales
Cstrobi_Rhytismatales
Edeformans_Rhytismatales
Lnitens_Rhytismatales
Pelatina_Triblidiales
Sflavida_Rhytismatales
Zvarium_Lulworthiales
Rericae_Helotiales
Clongipes_Helotiales
Mbicolor_Helotiales
Amacrosclerotiorum_Helotiales
Pscopiformis_Helotiales
Ljuncicola_Helotiales
Lmacrosporus_Helotiales
Glozoyensis_Helotiales
Tstercoreus_Thelebolales
Tmicrosporus_Thelebolales
Rfirma_Helotiales
Bcalthae_Helotiales
Bgalanthina_Helotiales
Btulipae_Helotiales
Bhyacinthi_Helotiales
Bpaeoniae_Helotiales
Bpseudocinerea_Helotiales
Bnarcissicola_Helotiales
Belliptica_Helotiales
Bporri_Helotiales
Bcinerea_Helotiales
Bmedusae_Helotiales
Bconvoluta_Helotiales
taxa
20
40
60
80
100
0
1
2
3
4
5
6
7
8
branch length

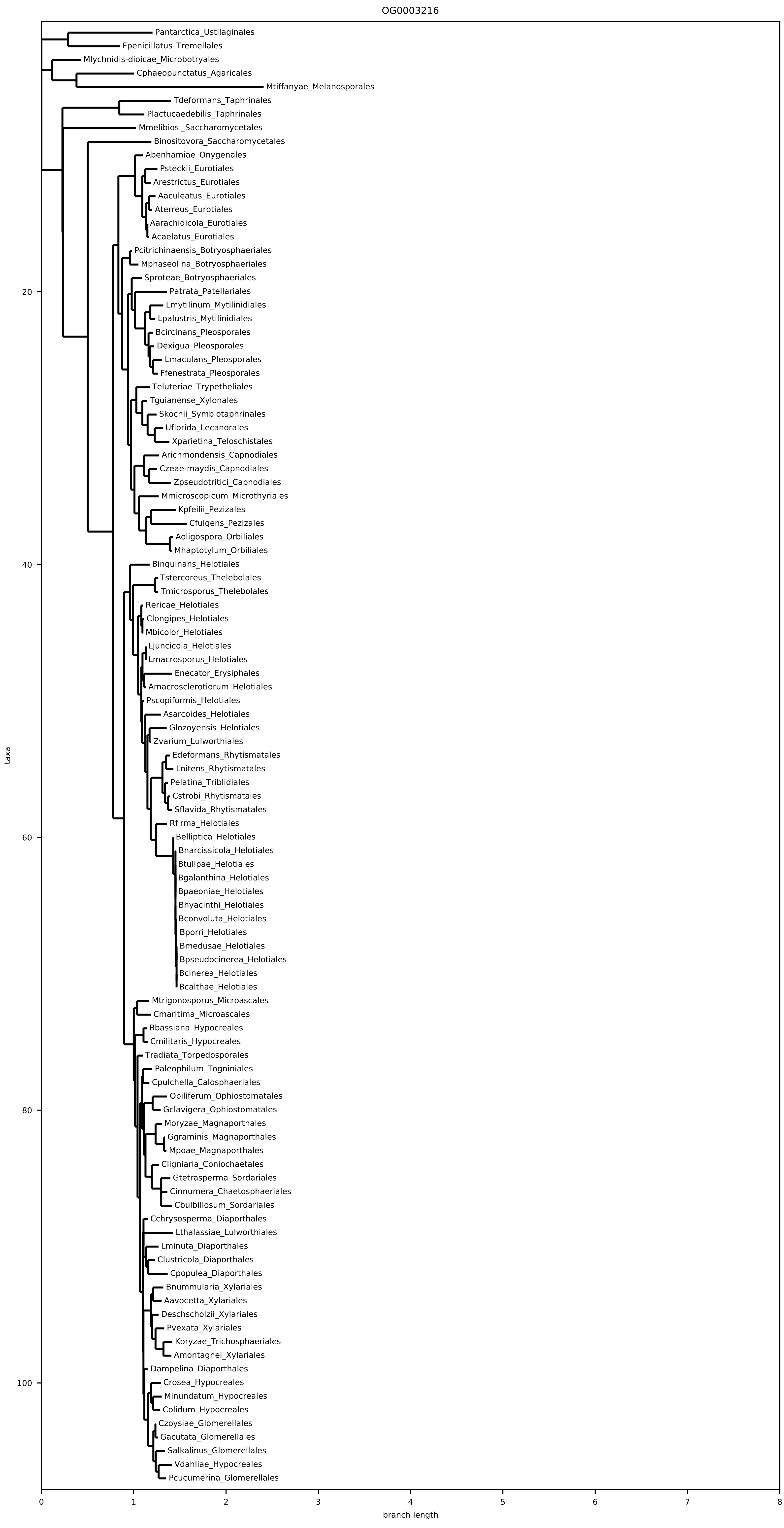
OG0003216
Pantarctica_Ustilaginales
Fpenicillatus_Tremellales
Mlychnidis-dioicae_Microbotryales
Cphaeopunctatus_Agaricales
Mtiffanyae_Melanosporales
Tdeformans_Taphrinales
Plactucaedebilis_Taphrinales
Mmelibiosi_Saccharomycetales
Binositovora_Saccharomycetales
Abenhamiae_Onygenales
Psteckii_Eurotiales
Arestrictus_Eurotiales
Aaculeatus_Eurotiales
Aterreus_Eurotiales
Aarachidicola_Eurotiales
Acaelatus_Eurotiales
Pcitrichinaensis_Botryosphaeriales
Mphaseolina_Botryosphaeriales
Sproteae_Botryosphaeriales
Patrata_Patellariales
Lmytilinum_Mytilinidiales
Lpalustris_Mytilinidiales
Bcircinans_Pleosporales
Dexigua_Pleosporales
Lmaculans_Pleosporales
Ffenestrata_Pleosporales
Teluteriae_Trypetheliales
Tguianense_Xylonales
Skochii_Symbiotaphrinales
Uflorida_Lecanorales
Xparietina_Teloschistales
Arichmondensis_Capnodiales
Czeae-maydis_Capnodiales
Zpseudotritici_Capnodiales
Mmicroscopicum_Microthyriales
Kpfeilii_Pezizales
Cfulgens_Pezizales
Aoligospora_Orbiliales
Mhaptotylum_Orbiliales
Binquinans_Helotiales
Tstercoreus_Thelebolales
Tmicrosporus_Thelebolales
Rericae_Helotiales
Clongipes_Helotiales
Mbicolor_Helotiales
Ljuncicola_Helotiales
Lmacrosporus_Helotiales
Enecator_Erysiphales
Amacrosclerotiorum_Helotiales
Pscopiformis_Helotiales
Asarcoides_Helotiales
Glozoyensis_Helotiales
Zvarium_Lulworthiales
Edeformans_Rhytismatales
Lnitens_Rhytismatales
Pelatina_Triblidiales
Cstrobi_Rhytismatales
Sflavida_Rhytismatales
Rfirma_Helotiales
Belliptica_Helotiales
Bnarcissicola_Helotiales
Btulipae_Helotiales
Bgalanthina_Helotiales
Bpaeoniae_Helotiales
Bhyacinthi_Helotiales
Bconvoluta_Helotiales
Bporri_Helotiales
Bmedusae_Helotiales
Bpseudocinerea_Helotiales
Bcinerea_Helotiales
Bcalthae_Helotiales
Mtrigonosporus_Microascales
Cmaritima_Microascales
Bbassiana_Hypocreales
Cmilitaris_Hypocreales
Tradiata_Torpedosporales
Paleophilum_Togniniales
Cpulchella_Calosphaeriales
Opiliferum_Ophiostomatales
Gclavigera_Ophiostomatales
Moryzae_Magnaporthales
Ggraminis_Magnaporthales
Mpoae_Magnaporthales
Cligniaria_Coniochaetales
Gtetrasperma_Sordariales
Cinnumera_Chaetosphaeriales
Cbulbillosum_Sordariales
Cchrysosperma_Diaporthales
Lthalassiae_Lulworthiales
Lminuta_Diaporthales
Clustricola_Diaporthales
Cpopulea_Diaporthales
Bnummularia_Xylariales
Aavocetta_Xylariales
Deschscholzii_Xylariales
Pvexata_Xylariales
Koryzae_Trichosphaeriales
Amontagnei_Xylariales
Dampelina_Diaporthales
Crosea_Hypocreales
Minundatum_Hypocreales
Colidum_Hypocreales
Czoysiae_Glomerellales
Gacutata_Glomerellales
Salkalinus_Glomerellales
Vdahliae_Hypocreales
Pcucumerina_Glomerellales
taxa
20
40
60
80
100
0
1
2
3
4
5
6
7
8
branch length

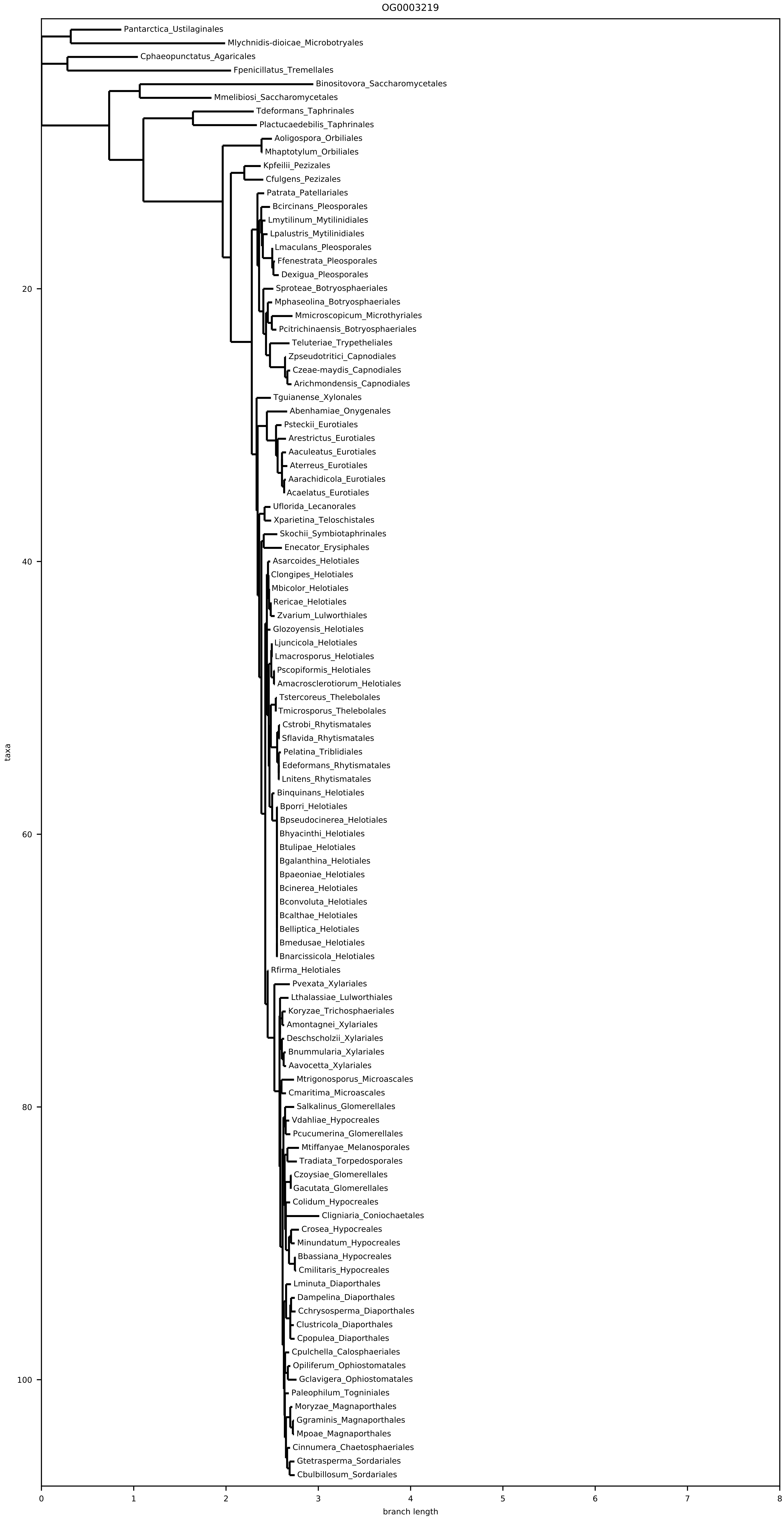
OG0003219
Pantarctica_Ustilaginales
Mlychnidis-dioicae_Microbotryales
Cphaeopunctatus_Agaricales
Fpenicillatus_Tremellales
Binositovora_Saccharomycetales
Mmelibiosi_Saccharomycetales
Tdeformans_Taphrinales
Plactucaedebilis_Taphrinales
Aoligospora_Orbiliales
Mhaptotylum_Orbiliales
Kpfeilii_Pezizales
Cfulgens_Pezizales
Patrata_Patellariales
Bcircinans_Pleosporales
Lmytilinum_Mytilinidiales
Lpalustris_Mytilinidiales
Lmaculans_Pleosporales
Ffenestrata_Pleosporales
Dexigua_Pleosporales
Sproteae_Botryosphaeriales
Mphaseolina_Botryosphaeriales
Mmicroscopicum_Microthyriales
Pcitrichinaensis_Botryosphaeriales
Teluteriae_Trypetheliales
Zpseudotritici_Capnodiales
Czeae-maydis_Capnodiales
Arichmondensis_Capnodiales
Tguianense_Xylonales
Abenhamiae_Onygenales
Psteckii_Eurotiales
Arestrictus_Eurotiales
Aaculeatus_Eurotiales
Aterreus_Eurotiales
Aarachidicola_Eurotiales
Acaelatus_Eurotiales
Uflorida_Lecanorales
Xparietina_Teloschistales
Skochii_Symbiotaphrinales
Enecator_Erysiphales
Asarcoides_Helotiales
Clongipes_Helotiales
Mbicolor_Helotiales
Rericae_Helotiales
Zvarium_Lulworthiales
Glozoyensis_Helotiales
Ljuncicola_Helotiales
Lmacrosporus_Helotiales
Pscopiformis_Helotiales
Amacrosclerotiorum_Helotiales
Tstercoreus_Thelebolales
Tmicrosporus_Thelebolales
Cstrobi_Rhytismatales
Sflavida_Rhytismatales
Pelatina_Triblidiales
Edeformans_Rhytismatales
Lnitens_Rhytismatales
Binquinans_Helotiales
Bporri_Helotiales
Bpseudocinerea_Helotiales
Bhyacinthi_Helotiales
Btulipae_Helotiales
Bgalanthina_Helotiales
Bpaeoniae_Helotiales
Bcinerea_Helotiales
Bconvoluta_Helotiales
Bcalthae_Helotiales
Belliptica_Helotiales
Bmedusae_Helotiales
Bnarcissicola_Helotiales
Rfirma_Helotiales
Pvexata_Xylariales
Lthalassiae_Lulworthiales
Koryzae_Trichosphaeriales
Amontagnei_Xylariales
Deschscholzii_Xylariales
Bnummularia_Xylariales
Aavocetta_Xylariales
Mtrigonosporus_Microascales
Cmaritima_Microascales
Salkalinus_Glomerellales
Vdahliae_Hypocreales
Pcucumerina_Glomerellales
Mtiffanyae_Melanosporales
Tradiata_Torpedosporales
Czoysiae_Glomerellales
Gacutata_Glomerellales
Colidum_Hypocreales
Cligniaria_Coniochaetales
Crosea_Hypocreales
Minundatum_Hypocreales
Bbassiana_Hypocreales
Cmilitaris_Hypocreales
Lminuta_Diaporthales
Dampelina_Diaporthales
Cchrysosperma_Diaporthales
Clustricola_Diaporthales
Cpopulea_Diaporthales
Cpulchella_Calosphaeriales
Opiliferum_Ophiostomatales
Gclavigera_Ophiostomatales
Paleophilum_Togniniales
Moryzae_Magnaporthales
Ggraminis_Magnaporthales
Mpoae_Magnaporthales
Cinnumera_Chaetosphaeriales
Gtetrasperma_Sordariales
Cbulbillosum_Sordariales
taxa
20
40
60
80
100
0
1
2
3
4
5
6
7
8
branch length

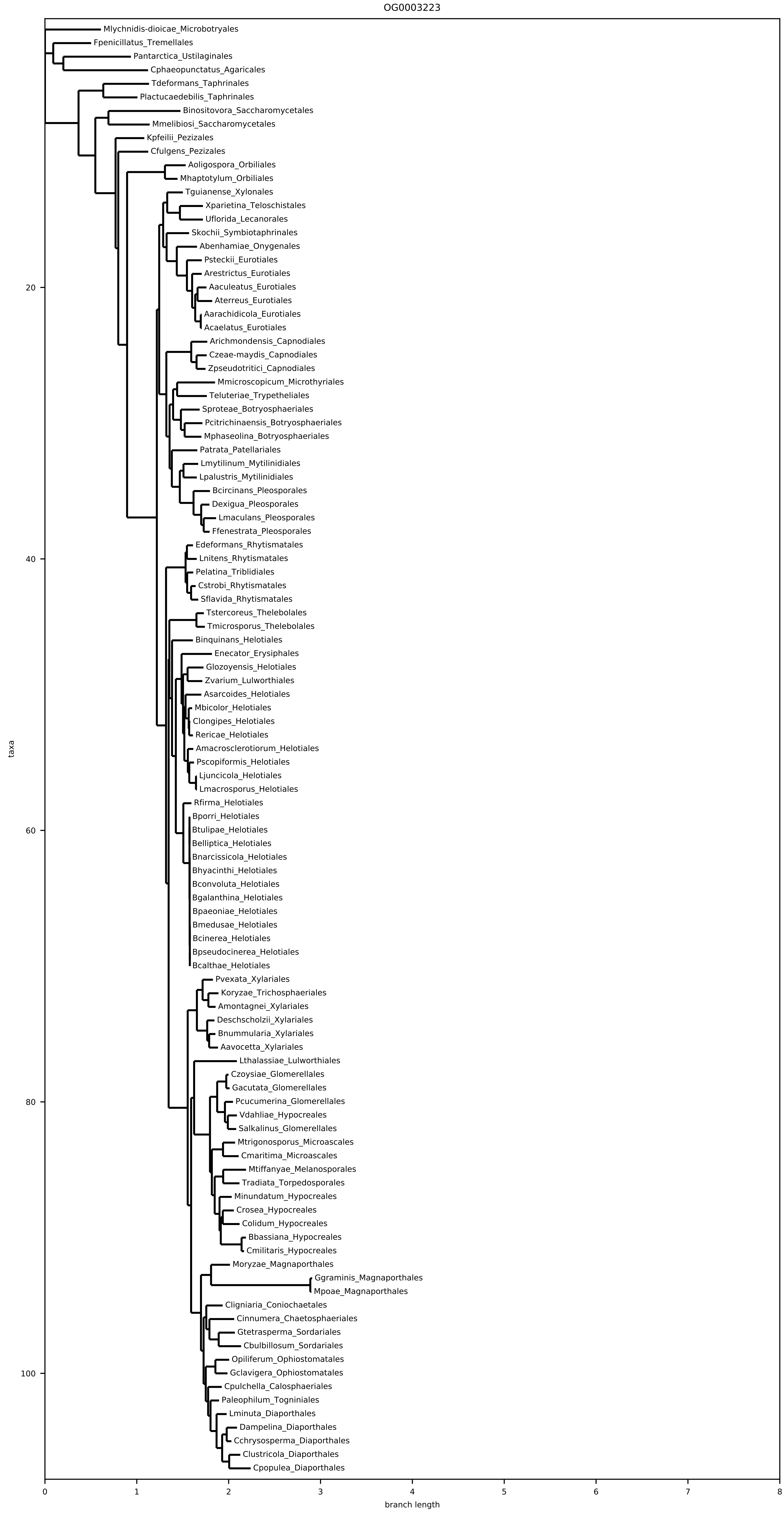

OG0003223
Mlychnidis-dioicae_Microbotryales
Fpenicillatus_Tremellales
Pantarctica_Ustilaginales
Cphaeopunctatus_Agaricales
Tdeformans_Taphrinales
Plactucaedebilis_Taphrinales
Binositovora_Saccharomycetales
Mmelibiosi_Saccharomycetales
Kpfeilii_Pezizales
Cfulgens_Pezizales
Aoligospora_Orbiliales
Mhaptotylum_Orbiliales
Tguianense_Xylonales
Xparietina_Teloschistales
Uflorida_Lecanorales
Skochii_Symbiotaphrinales
Abenhamiae_Onygenales
Psteckii_Eurotiales
Arestrictus_Eurotiales
Aaculeatus_Eurotiales
Aterreus_Eurotiales
Aarachidicola_Eurotiales
Acaelatus_Eurotiales
Arichmondensis_Capnodiales
Czeae-maydis_Capnodiales
Zpseudotritici_Capnodiales
Mmicroscopicum_Microthyriales
Teluteriae_Trypetheliales
Sproteae_Botryosphaeriales
Pcitrichinaensis_Botryosphaeriales
Mphaseolina_Botryosphaeriales
Patrata_Patellariales
Lmytilinum_Mytilinidiales
Lpalustris_Mytilinidiales
Bcircinans_Pleosporales
Dexigua_Pleosporales
Lmaculans_Pleosporales
Ffenestrata_Pleosporales
Edeformans_Rhytismatales
Lnitens_Rhytismatales
Pelatina_Triblidiales
Cstrobi_Rhytismatales
Sflavida_Rhytismatales
Tstercoreus_Thelebolales
Tmicrosporus_Thelebolales
Binquinans_Helotiales
Enecator_Erysiphales
Glozoyensis_Helotiales
Zvarium_Lulworthiales
Asarcoides_Helotiales
Mbicolor_Helotiales
Clongipes_Helotiales
Rericae_Helotiales
Amacrosclerotiorum_Helotiales
Pscopiformis_Helotiales
Ljuncicola_Helotiales
Lmacrosporus_Helotiales
Rfirma_Helotiales
Bporri_Helotiales
Btulipae_Helotiales
Belliptica_Helotiales
Bnarcissicola_Helotiales
Bhyacinthi_Helotiales
Bconvoluta_Helotiales
Bgalanthina_Helotiales
Bpaeoniae_Helotiales
Bmedusae_Helotiales
Bcinerea_Helotiales
Bpseudocinerea_Helotiales
Bcalthae_Helotiales
Pvexata_Xylariales
Koryzae_Trichosphaeriales
Amontagnei_Xylariales
Deschscholzii_Xylariales
Bnummularia_Xylariales
Aavocetta_Xylariales
Lthalassiae_Lulworthiales
Czoysiae_Glomerellales
Gacutata_Glomerellales
Pcucumerina_Glomerellales
Vdahliae_Hypocreales
Salkalinus_Glomerellales
Mtrigonosporus_Microascales
Cmaritima_Microascales
Mtiffanyae_Melanosporales
Tradiata_Torpedosporales
Minundatum_Hypocreales
Crosea_Hypocreales
Colidum_Hypocreales
Bbassiana_Hypocreales
Cmilitaris_Hypocreales
Moryzae_Magnaporthales
Ggraminis_Magnaporthales
Mpoae_Magnaporthales
Cligniaria_Coniochaetales
Cinnumera_Chaetosphaeriales
Gtetrasperma_Sordariales
Cbulbillosum_Sordariales
Opiliferum_Ophiostomatales
Gclavigera_Ophiostomatales
Cpulchella_Calosphaeriales
Paleophilum_Togniniales
Lminuta_Diaporthales
Dampelina_Diaporthales
Cchrysosperma_Diaporthales
Clustricola_Diaporthales
Cpopulea_Diaporthales
taxa
20
40
60
80
100
0
1
2
3
4
5
6
7
8
branch length

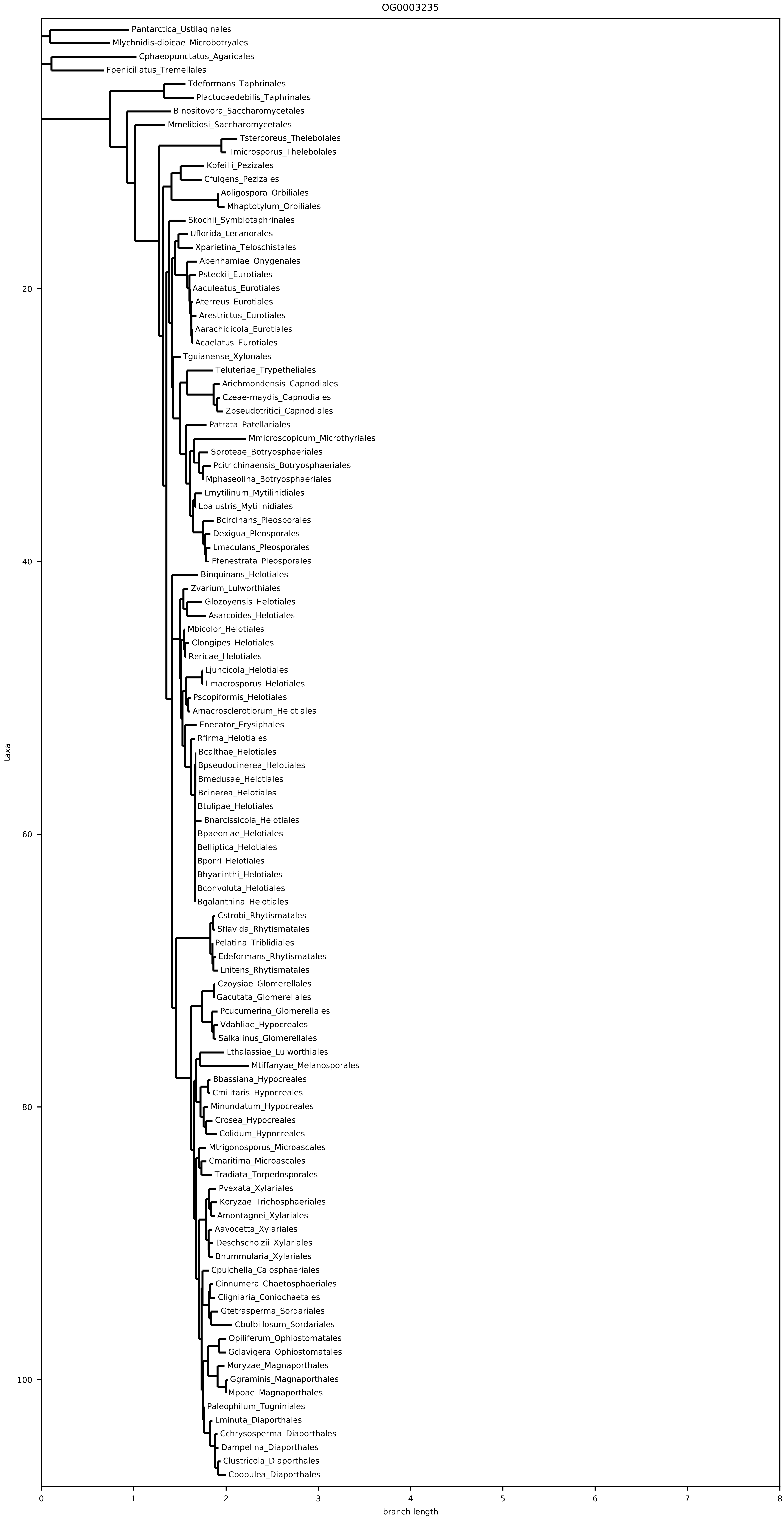
OG0003235
Pantarctica_Ustilaginales
Mlychnidis-dioicae_Microbotryales
Cphaeopunctatus_Agaricales
Fpenicillatus_Tremellales
Tdeformans_Taphrinales
Plactucaedebilis_Taphrinales
Binositovora_Saccharomycetales
Mmelibiosi_Saccharomycetales
Tstercoreus_Thelebolales
Tmicrosporus_Thelebolales
Kpfeilii_Pezizales
Cfulgens_Pezizales
Aoligospora_Orbiliales
Mhaptotylum_Orbiliales
Skochii_Symbiotaphrinales
Uflorida_Lecanorales
Xparietina_Teloschistales
Abenhamiae_Onygenales
Psteckii_Eurotiales
Aaculeatus_Eurotiales
Aterreus_Eurotiales
Arestrictus_Eurotiales
Aarachidicola_Eurotiales
Acaelatus_Eurotiales
Tguianense_Xylonales
Teluteriae_Trypetheliales
Arichmondensis_Capnodiales
Czeae-maydis_Capnodiales
Zpseudotritici_Capnodiales
Patrata_Patellariales
Mmicroscopicum_Microthyriales
Sproteae_Botryosphaeriales
Pcitrichinaensis_Botryosphaeriales
Mphaseolina_Botryosphaeriales
Lmytilinum_Mytilinidiales
Lpalustris_Mytilinidiales
Bcircinans_Pleosporales
Dexigua_Pleosporales
Lmaculans_Pleosporales
Ffenestrata_Pleosporales
Binquinans_Helotiales
Zvarium_Lulworthiales
Glozoyensis_Helotiales
Asarcoides_Helotiales
Mbicolor_Helotiales
Clongipes_Helotiales
Rericae_Helotiales
Ljuncicola_Helotiales
Lmacrosporus_Helotiales
Pscopiformis_Helotiales
Amacrosclerotiorum_Helotiales
Enecator_Erysiphales
Rfirma_Helotiales
Bcalthae_Helotiales
Bpseudocinerea_Helotiales
Bmedusae_Helotiales
Bcinerea_Helotiales
Btulipae_Helotiales
Bnarcissicola_Helotiales
Bpaeoniae_Helotiales
Belliptica_Helotiales
Bporri_Helotiales
Bhyacinthi_Helotiales
Bconvoluta_Helotiales
Bgalanthina_Helotiales
Cstrobi_Rhytismatales
Sflavida_Rhytismatales
Pelatina_Triblidiales
Edeformans_Rhytismatales
Lnitens_Rhytismatales
Czoysiae_Glomerellales
Gacutata_Glomerellales
Pcucumerina_Glomerellales
Vdahliae_Hypocreales
Salkalinus_Glomerellales
Lthalassiae_Lulworthiales
Mtiffanyae_Melanosporales
Bbassiana_Hypocreales
Cmilitaris_Hypocreales
Minundatum_Hypocreales
Crosea_Hypocreales
Colidum_Hypocreales
Mtrigonosporus_Microascales
Cmaritima_Microascales
Tradiata_Torpedosporales
Pvexata_Xylariales
Koryzae_Trichosphaeriales
Amontagnei_Xylariales
Aavocetta_Xylariales
Deschscholzii_Xylariales
Bnummularia_Xylariales
Cpulchella_Calosphaeriales
Cinnumera_Chaetosphaeriales
Cligniaria_Coniochaetales
Gtetrasperma_Sordariales
Cbulbillosum_Sordariales
Opiliferum_Ophiostomatales
Gclavigera_Ophiostomatales
Moryzae_Magnaporthales
Ggraminis_Magnaporthales
Mpoae_Magnaporthales
Paleophilum_Togniniales
Lminuta_Diaporthales
Cchrysosperma_Diaporthales
Dampelina_Diaporthales
Clustricola_Diaporthales
Cpopulea_Diaporthales
taxa
20
40
60
80
100
0
1
2
3
4
5
6
7
8
branch length

OG0003236

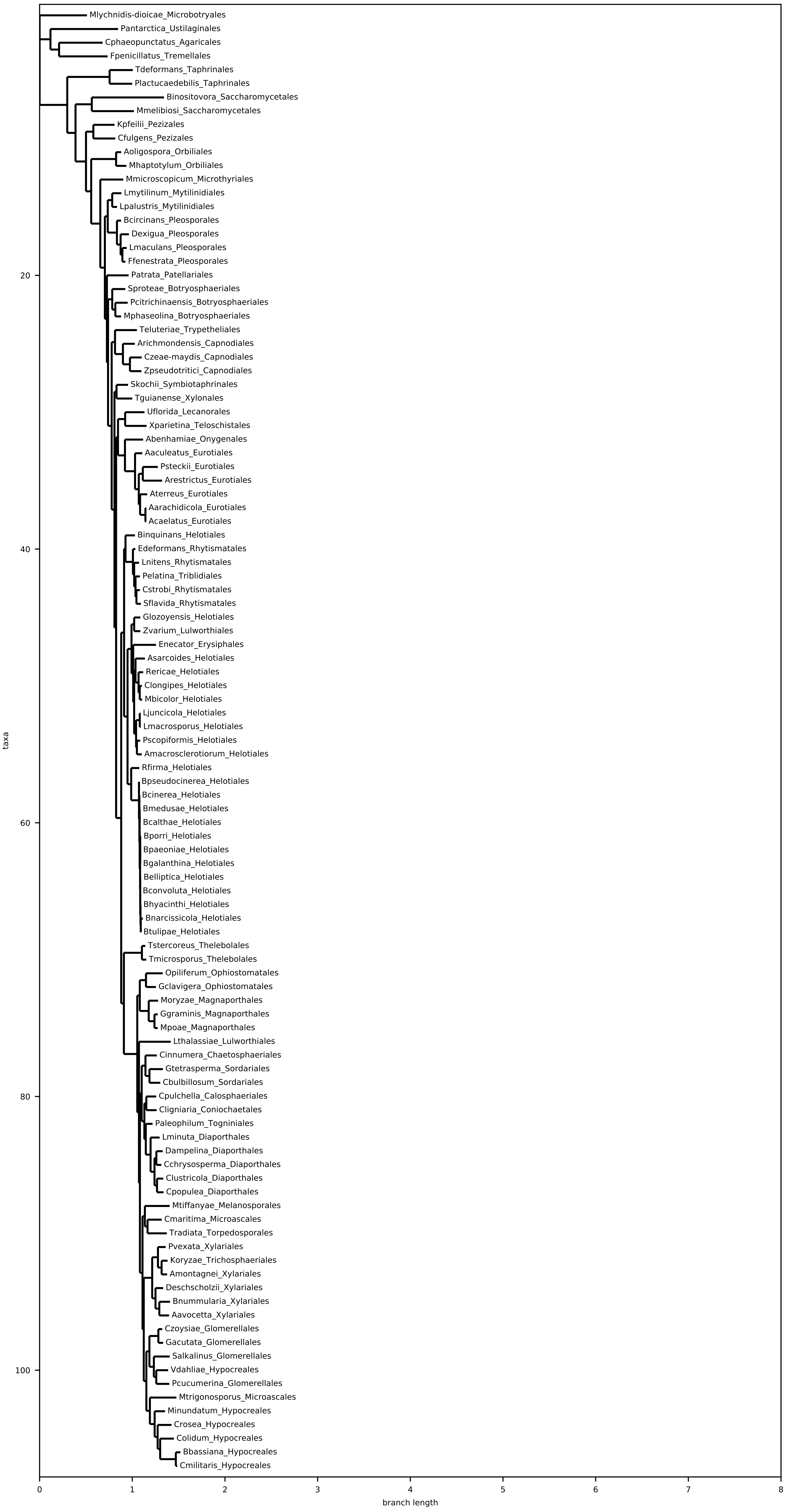
Mlychnidis-dioicae_Microbotryales
Pantarctica_Ustilaginales
Cphaeopunctatus_Agaricales
Fpenicillatus_Tremellales
Tdeformans_Taphrinales
Plactucaedebilis_Taphrinales
Binositovora_Saccharomycetales
Mmelibiosi_Saccharomycetales
Kpfeilii_Pezizales
Cfulgens_Pezizales
Aoligospora_Orbiliales
Mhaptotylum_Orbiliales
Mmicroscopicum_Microthyriales
Lmytilinum_Mytilinidiales
Lpalustris_Mytilinidiales
Bcircinans_Pleosporales
Dexigua_Pleosporales
Lmaculans_Pleosporales
Ffenestrata_Pleosporales
Patrata_Patellariales
Sproteae_Botryosphaeriales
Pcitrichinaensis_Botryosphaeriales
Mphaseolina_Botryosphaeriales
Teluteriae_Trypetheliales
Arichmondensis_Capnodiales
Czeae-maydis_Capnodiales
Zpseudotritici_Capnodiales
Skochii_Symbiotaphrinales
Tguianense_Xylonales
Uflorida_Lecanorales
Xparietina_Teloschistales
Abenhamiae_Onygenales
Aaculeatus_Eurotiales
Psteckii_Eurotiales
Arestrictus_Eurotiales
Aterreus_Eurotiales
Aarachidicola_Eurotiales
Acaelatus_Eurotiales
Binquinans_Helotiales
Edeformans_Rhytismatales
Lnitens_Rhytismatales
Pelatina_Triblidiales
Cstrobi_Rhytismatales
Sflavida_Rhytismatales
Glozoyensis_Helotiales
Zvarium_Lulworthiales
Enecator_Erysiphales
Asarcoides_Helotiales
Rericae_Helotiales
Clongipes_Helotiales
Mbicolor_Helotiales
Ljuncicola_Helotiales
Lmacrosporus_Helotiales
Pscopiformis_Helotiales
Amacrosclerotiorum_Helotiales
Rfirma_Helotiales
Bpseudocinerea_Helotiales
Bcinerea_Helotiales
Bmedusae_Helotiales
Bcalthae_Helotiales
Bporri_Helotiales
Bpaeoniae_Helotiales
Bgalanthina_Helotiales
Belliptica_Helotiales
Bconvoluta_Helotiales
Bhyacinthi_Helotiales
Bnarcissicola_Helotiales
Btulipae_Helotiales
Tstercoreus_Thelebolales
Tmicrosporus_Thelebolales
Opiliferum_Ophiostomatales
Gclavigera_Ophiostomatales
Moryzae_Magnaporthales
Ggraminis_Magnaporthales
Mpoae_Magnaporthales
Lthalassiae_Lulworthiales
Cinnumera_Chaetosphaeriales
Gtetrasperma_Sordariales
Cbulbillosum_Sordariales
Cpulchella_Calosphaeriales
Cligniaria_Coniochaetales
Paleophilum_Togniniales
Lminuta_Diaporthales
Dampelina_Diaporthales
Cchrysosperma_Diaporthales
Clustricola_Diaporthales
Cpopulea_Diaporthales
Mtiffanyae_Melanosporales
Cmaritima_Microascales
Tradiata_Torpedosporales
Pvexata_Xylariales
Koryzae_Trichosphaeriales
Amontagnei_Xylariales
Deschscholzii_Xylariales
Bnummularia_Xylariales
Aavocetta_Xylariales
Czoysiae_Glomerellales
Gacutata_Glomerellales
Salkalinus_Glomerellales
Vdahliae_Hypocreales
Pcucumerina_Glomerellales
Mtrigonosporus_Microascales
Minundatum_Hypocreales
Crosea_Hypocreales
Colidum_Hypocreales
Bbassiana_Hypocreales
Cmilitaris_Hypocreales
taxa
20
40
60
80
100
0
1
2
3
4
5
6
7
8
branch length

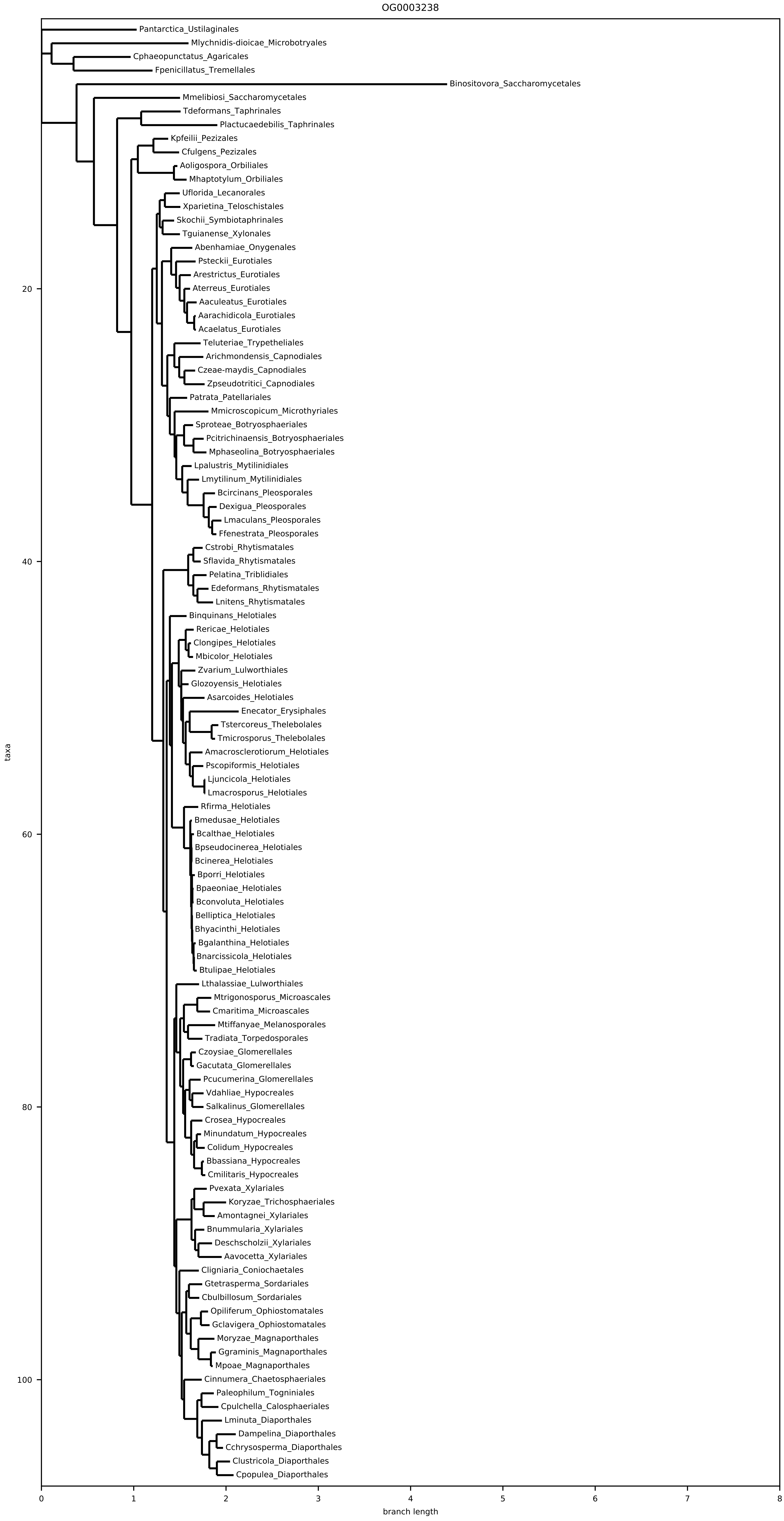

OG0003238
Pantarctica_Ustilaginales
Mlychnidis-dioicae_Microbotryales
Cphaeopunctatus_Agaricales
Fpenicillatus_Tremellales
Binositovora_Saccharomycetales
Mmelibiosi_Saccharomycetales
Tdeformans_Taphrinales
Plactucaedebilis_Taphrinales
Kpfeilii_Pezizales
Cfulgens_Pezizales
Aoligospora_Orbiliales
Mhaptotylum_Orbiliales
Uflorida_Lecanorales
Xparietina_Teloschistales
Skochii_Symbiotaphrinales
Tguianense_Xylonales
Abenhamiae_Onygenales
Psteckii_Eurotiales
Arestrictus_Eurotiales
Aterreus_Eurotiales
Aaculeatus_Eurotiales
Aarachidicola_Eurotiales
Acaelatus_Eurotiales
Teluteriae_Trypetheliales
Arichmondensis_Capnodiales
Czeae-maydis_Capnodiales
Zpseudotritici_Capnodiales
Patrata_Patellariales
Mmicroscopicum_Microthyriales
Sproteae_Botryosphaeriales
Pcitrichinaensis_Botryosphaeriales
Mphaseolina_Botryosphaeriales
Lpalustris_Mytilinidiales
Lmytilinum_Mytilinidiales
Bcircinans_Pleosporales
Dexigua_Pleosporales
Lmaculans_Pleosporales
Ffenestrata_Pleosporales
Cstrobi_Rhytismatales
Sflavida_Rhytismatales
Pelatina_Triblidiales
Edeformans_Rhytismatales
Lnitens_Rhytismatales
Binquinans_Helotiales
Rericae_Helotiales
Clongipes_Helotiales
Mbicolor_Helotiales
Zvarium_Lulworthiales
Glozoyensis_Helotiales
Asarcoides_Helotiales
Enecator_Erysiphales
Tstercoreus_Thelebolales
Tmicrosporus_Thelebolales
Amacrosclerotiorum_Helotiales
Pscopiformis_Helotiales
Ljuncicola_Helotiales
Lmacrosporus_Helotiales
Rfirma_Helotiales
Bmedusae_Helotiales
Bcalthae_Helotiales
Bpseudocinerea_Helotiales
Bcinerea_Helotiales
Bporri_Helotiales
Bpaeoniae_Helotiales
Bconvoluta_Helotiales
Belliptica_Helotiales
Bhyacinthi_Helotiales
Bgalanthina_Helotiales
Bnarcissicola_Helotiales
Btulipae_Helotiales
Lthalassiae_Lulworthiales
Mtrigonosporus_Microascales
Cmaritima_Microascales
Mtiffanyae_Melanosporales
Tradiata_Torpedosporales
Czoysiae_Glomerellales
Gacutata_Glomerellales
Pcucumerina_Glomerellales
Vdahliae_Hypocreales
Salkalinus_Glomerellales
Crosea_Hypocreales
Minundatum_Hypocreales
Colidum_Hypocreales
Bbassiana_Hypocreales
Cmilitaris_Hypocreales
Pvexata_Xylariales
Koryzae_Trichosphaeriales
Amontagnei_Xylariales
Bnummularia_Xylariales
Deschscholzii_Xylariales
Aavocetta_Xylariales
Cligniaria_Coniochaetales
Gtetrasperma_Sordariales
Cbulbillosum_Sordariales
Opiliferum_Ophiostomatales
Gclavigera_Ophiostomatales
Moryzae_Magnaporthales
Ggraminis_Magnaporthales
Mpoae_Magnaporthales
Cinnumera_Chaetosphaeriales
Paleophilum_Togniniales
Cpulchella_Calosphaeriales
Lminuta_Diaporthales
Dampelina_Diaporthales
Cchrysosperma_Diaporthales
Clustricola_Diaporthales
Cpopulea_Diaporthales
taxa
20
40
60
80
100
0
1
2
3
4
5
6
7
8
branch length

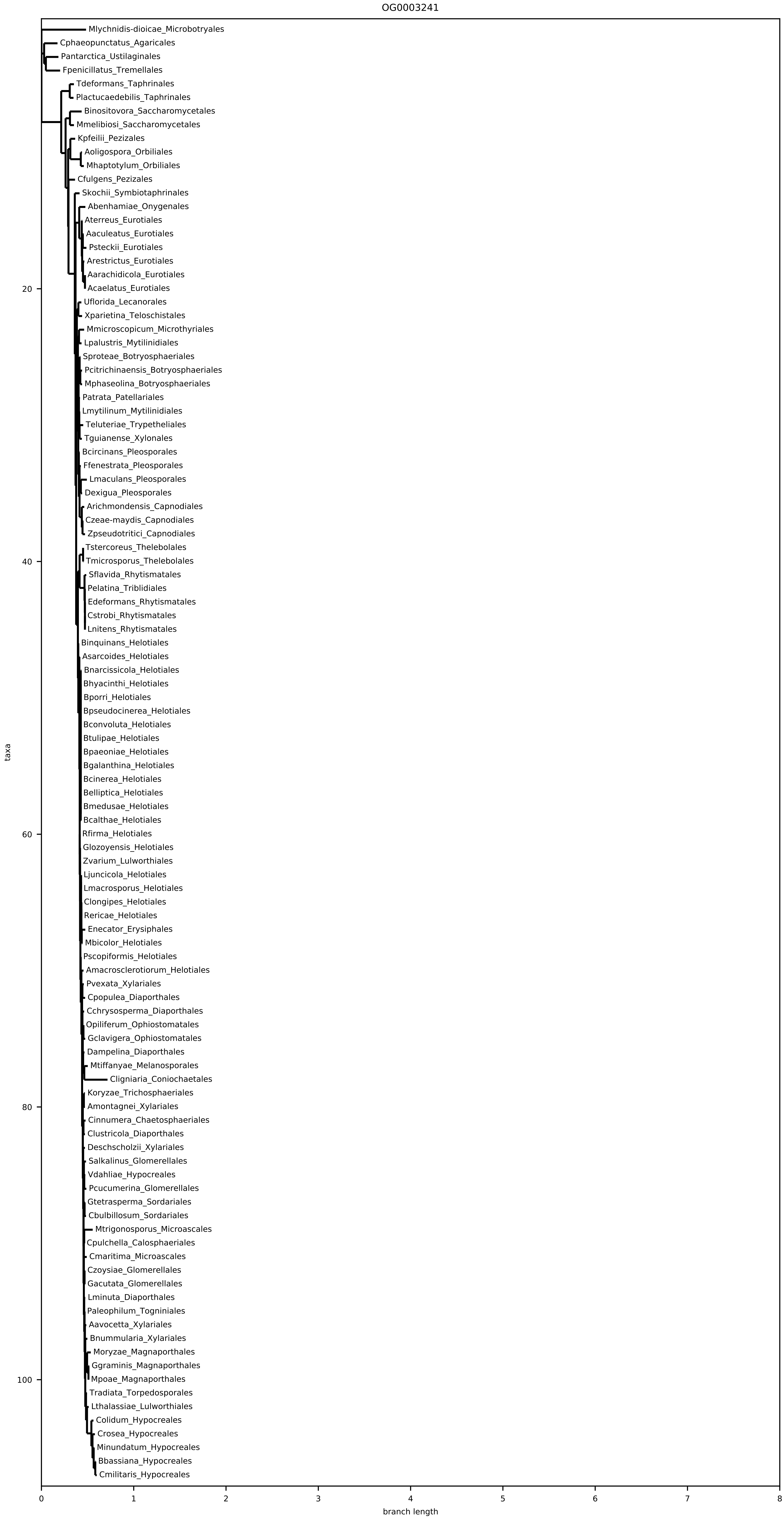
OG0003241
Mlychnidis-dioicae_Microbotryales
Cphaeopunctatus_Agaricales
Pantarctica_Ustilaginales
Fpenicillatus_Tremellales
Tdeformans_Taphrinales
Plactucaedebilis_Taphrinales
Binositovora_Saccharomycetales
Mmelibiosi_Saccharomycetales
Kpfeilii_Pezizales
Aoligospora_Orbiliales
Mhaptotylum_Orbiliales
Cfulgens_Pezizales
Skochii_Symbiotaphrinales
Abenhamiae_Onygenales
Aterreus_Eurotiales
Aaculeatus_Eurotiales
Psteckii_Eurotiales
Arestrictus_Eurotiales
Aarachidicola_Eurotiales
Acaelatus_Eurotiales
Uflorida_Lecanorales
Xparietina_Teloschistales
Mmicroscopicum_Microthyriales
Lpalustris_Mytilinidiales
Sproteae_Botryosphaeriales
Pcitrichinaensis_Botryosphaeriales
Mphaseolina_Botryosphaeriales
Patrata_Patellariales
Lmytilinum_Mytilinidiales
Teluteriae_Trypetheliales
Tguianense_Xylonales
Bcircinans_Pleosporales
Ffenestrata_Pleosporales
Lmaculans_Pleosporales
Dexigua_Pleosporales
Arichmondensis_Capnodiales
Czeae-maydis_Capnodiales
Zpseudotritici_Capnodiales
Tstercoreus_Thelebolales
Tmicrosporus_Thelebolales
Sflavida_Rhytismatales
Pelatina_Triblidiales
Edeformans_Rhytismatales
Cstrobi_Rhytismatales
Lnitens_Rhytismatales
Binquinans_Helotiales
Asarcoides_Helotiales
Bnarcissicola_Helotiales
Bhyacinthi_Helotiales
Bporri_Helotiales
Bpseudocinerea_Helotiales
Bconvoluta_Helotiales
Btulipae_Helotiales
Bpaeoniae_Helotiales
Bgalanthina_Helotiales
Bcinerea_Helotiales
Belliptica_Helotiales
Bmedusae_Helotiales
Bcalthae_Helotiales
Rfirma_Helotiales
Glozoyensis_Helotiales
Zvarium_Lulworthiales
Ljuncicola_Helotiales
Lmacrosporus_Helotiales
Clongipes_Helotiales
Rericae_Helotiales
Enecator_Erysiphales
Mbicolor_Helotiales
Pscopiformis_Helotiales
Amacrosclerotiorum_Helotiales
Pvexata_Xylariales
Cpopulea_Diaporthales
Cchrysosperma_Diaporthales
Opiliferum_Ophiostomatales
Gclavigera_Ophiostomatales
Dampelina_Diaporthales
Mtiffanyae_Melanosporales
Cligniaria_Coniochaetales
Koryzae_Trichosphaeriales
Amontagnei_Xylariales
Cinnumera_Chaetosphaeriales
Clustricola_Diaporthales
Deschscholzii_Xylariales
Salkalinus_Glomerellales
Vdahliae_Hypocreales
Pcucumerina_Glomerellales
Gtetrasperma_Sordariales
Cbulbillosum_Sordariales
Mtrigonosporus_Microascales
Cpulchella_Calosphaeriales
Cmaritima_Microascales
Czoysiae_Glomerellales
Gacutata_Glomerellales
Lminuta_Diaporthales
Paleophilum_Togniniales
Aavocetta_Xylariales
Bnummularia_Xylariales
Moryzae_Magnaporthales
Ggraminis_Magnaporthales
Mpoae_Magnaporthales
Tradiata_Torpedosporales
Lthalassiae_Lulworthiales
Colidum_Hypocreales
Crosea_Hypocreales
Minundatum_Hypocreales
Bbassiana_Hypocreales
Cmilitaris_Hypocreales
taxa
20
40
60
80
100
0
1
2
3
4
5
6
7
8
branch length

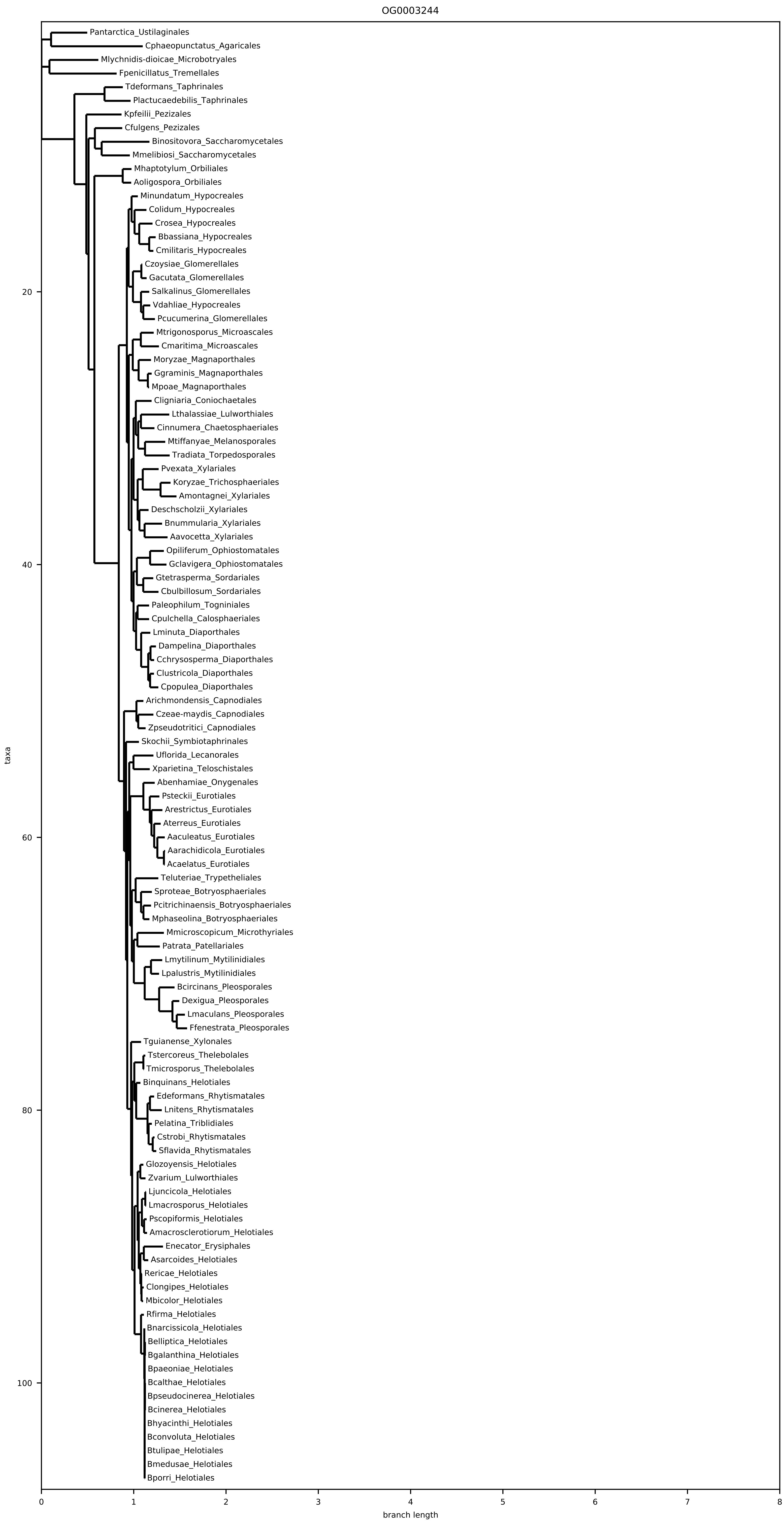
OG0003244
Pantarctica_Ustilaginales
Cphaeopunctatus_Agaricales
Mlychnidis-dioicae_Microbotryales
Fpenicillatus_Tremellales
Tdeformans_Taphrinales
Plactucaedebilis_Taphrinales
Kpfeilii_Pezizales
Cfulgens_Pezizales
Binositovora_Saccharomycetales
Mmelibiosi_Saccharomycetales
Mhaptotylum_Orbiliales
Aoligospora_Orbiliales
Minundatum_Hypocreales
Colidum_Hypocreales
Crosea_Hypocreales
Bbassiana_Hypocreales
Cmilitaris_Hypocreales
Czoysiae_Glomerellales
Gacutata_Glomerellales
Salkalinus_Glomerellales
Vdahliae_Hypocreales
Pcucumerina_Glomerellales
Mtrigonosporus_Microascales
Cmaritima_Microascales
Moryzae_Magnaporthales
Ggraminis_Magnaporthales
Mpoae_Magnaporthales
Cligniaria_Coniochaetales
Lthalassiae_Lulworthiales
Cinnumera_Chaetosphaeriales
Mtiffanyae_Melanosporales
Tradiata_Torpedosporales
Pvexata_Xylariales
Koryzae_Trichosphaeriales
Amontagnei_Xylariales
Deschscholzii_Xylariales
Bnummularia_Xylariales
Aavocetta_Xylariales
Opiliferum_Ophiostomatales
Gclavigera_Ophiostomatales
Gtetrasperma_Sordariales
Cbulbillosum_Sordariales
Paleophilum_Togniniales
Cpulchella_Calosphaeriales
Lminuta_Diaporthales
Dampelina_Diaporthales
Cchrysosperma_Diaporthales
Clustricola_Diaporthales
Cpopulea_Diaporthales
Arichmondensis_Capnodiales
Czeae-maydis_Capnodiales
Zpseudotritici_Capnodiales
Skochii_Symbiotaphrinales
Uflorida_Lecanorales
Xparietina_Teloschistales
Abenhamiae_Onygenales
Psteckii_Eurotiales
Arestrictus_Eurotiales
Aterreus_Eurotiales
Aaculeatus_Eurotiales
Aarachidicola_Eurotiales
Acaelatus_Eurotiales
Teluteriae_Trypetheliales
Sproteae_Botryosphaeriales
Pcitrichinaensis_Botryosphaeriales
Mphaseolina_Botryosphaeriales
Mmicroscopicum_Microthyriales
Patrata_Patellariales
Lmytilinum_Mytilinidiales
Lpalustris_Mytilinidiales
Bcircinans_Pleosporales
Dexigua_Pleosporales
Lmaculans_Pleosporales
Ffenestrata_Pleosporales
Tguianense_Xylonales
Tstercoreus_Thelebolales
Tmicrosporus_Thelebolales
Binquinans_Helotiales
Edeformans_Rhytismatales
Lnitens_Rhytismatales
Pelatina_Triblidiales
Cstrobi_Rhytismatales
Sflavida_Rhytismatales
Glozoyensis_Helotiales
Zvarium_Lulworthiales
Ljuncicola_Helotiales
Lmacrosporus_Helotiales
Pscopiformis_Helotiales
Amacrosclerotiorum_Helotiales
Enecator_Erysiphales
Asarcoides_Helotiales
Rericae_Helotiales
Clongipes_Helotiales
Mbicolor_Helotiales
Rfirma_Helotiales
Bnarcissicola_Helotiales
Belliptica_Helotiales
Bgalanthina_Helotiales
Bpaeoniae_Helotiales
Bcalthae_Helotiales
Bpseudocinerea_Helotiales
Bcinerea_Helotiales
Bhyacinthi_Helotiales
Bconvoluta_Helotiales
Btulipae_Helotiales
Bmedusae_Helotiales
Bporri_Helotiales
taxa
20
40
60
80
100
0
1
2
3
4
5
6
7
8
branch length

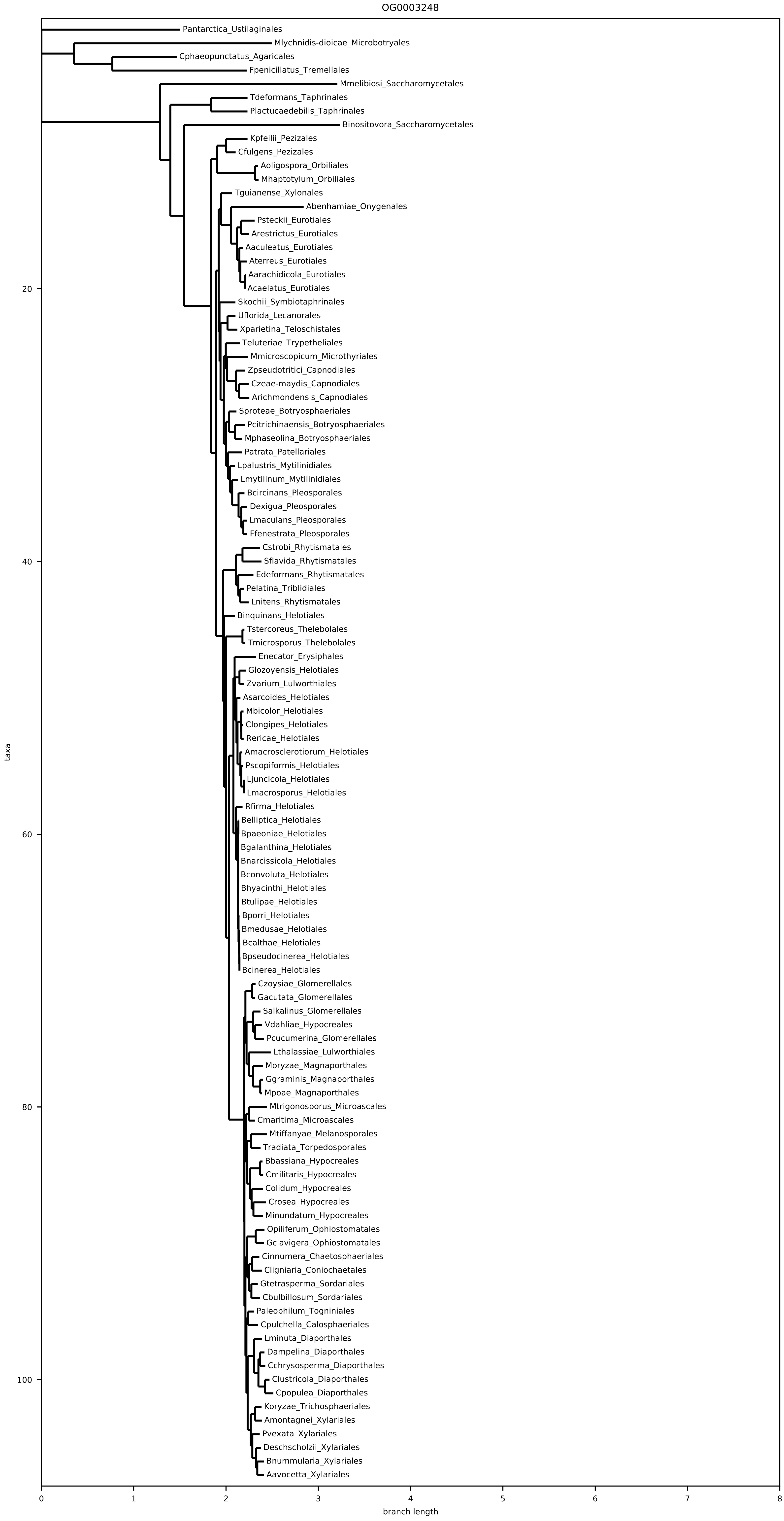

OG0003248
taxa
branch length
0
1
2
3
4
5
6
7
8
20
40
60
80
100
Pantarctica_Ustilaginales
Mlychnidis-dioicae_Microbotryales
Cphaeopunctatus_Agaricales
Fpenicillatus_Tremellales
Mmelibiosi_Saccharomycetales
Tdeformans_Taphrinales
Plactucaedebilis_Taphrinales
Binositovora_Saccharomycetales
Kpfeilii_Pezizales
Cfulgens_Pezizales
Aoligospora_Orbiliales
Mhaptotylum_Orbiliales
Tguianense_Xylonales
Abenhamiae_Onygenales
Psteckii_Eurotiales
Arestrictus_Eurotiales
Aaculeatus_Eurotiales
Aterreus_Eurotiales
Aarachidicola_Eurotiales
Acaelatus_Eurotiales
Skochii_Symbiotaphrinales
Uflorida_Lecanorales
Xparietina_Teloschistales
Teluteriae_Trypetheliales
Mmicroscopicum_Microthyriales
Zpseudotritici_Capnodiales
Czeae-maydis_Capnodiales
Arichmondensis_Capnodiales
Sproteae_Botryosphaeriales
Pcitrichinaensis_Botryosphaeriales
Mphaseolina_Botryosphaeriales
Patrata_Patellariales
Lpalustris_Mytilinidiales
Lmytilinum_Mytilinidiales
Bcircinans_Pleosporales
Dexigua_Pleosporales
Lmaculans_Pleosporales
Ffenestrata_Pleosporales
Cstrobi_Rhytismatales
Sflavida_Rhytismatales
Edeformans_Rhytismatales
Pelatina_Triblidiales
Lnitens_Rhytismatales
Binquinans_Helotiales
Tstercoreus_Thelebolales
Tmicrosporus_Thelebolales
Enecator_Erysiphales
Glozoyensis_Helotiales
Zvarium_Lulworthiales
Asarcoides_Helotiales
Mbicolor_Helotiales
Clongipes_Helotiales
Rericae_Helotiales
Amacrosclerotiorum_Helotiales
Pscopiformis_Helotiales
Ljuncicola_Helotiales
Lmacrosporus_Helotiales
Rfirma_Helotiales
Belliptica_Helotiales
Bpaeoniae_Helotiales
Bgalanthina_Helotiales
Bnarcissicola_Helotiales
Bconvoluta_Helotiales
Bhyacinthi_Helotiales
Btulipae_Helotiales
Bporri_Helotiales
Bmedusae_Helotiales
Bcalthae_Helotiales
Bpseudocinerea_Helotiales
Bcinerea_Helotiales
Czoysiae_Glomerellales
Gacutata_Glomerellales
Salkalinus_Glomerellales
Vdahliae_Hypocreales
Pcucumerina_Glomerellales
Lthalassiae_Lulworthiales
Moryzae_Magnaporthales
Ggraminis_Magnaporthales
Mpoae_Magnaporthales
Mtrigonosporus_Microascales
Cmaritima_Microascales
Mtiffanyae_Melanosporales
Tradiata_Torpedosporales
Bbassiana_Hypocreales
Cmilitaris_Hypocreales
Colidum_Hypocreales
Crosea_Hypocreales
Minundatum_Hypocreales
Opiliferum_Ophiostomatales
Gclavigera_Ophiostomatales
Cinnumera_Chaetosphaeriales
Cligniaria_Coniochaetales
Gtetrasperma_Sordariales
Cbulbillosum_Sordariales
Paleophilum_Togniniales
Cpulchella_Calosphaeriales
Lminuta_Diaporthales
Dampelina_Diaporthales
Cchrysosperma_Diaporthales
Clustricola_Diaporthales
Cpopulea_Diaporthales
Koryzae_Trichosphaeriales
Amontagnei_Xylariales
Pvexata_Xylariales
Deschscholzii_Xylariales
Bnummularia_Xylariales
Aavocetta_Xylariales

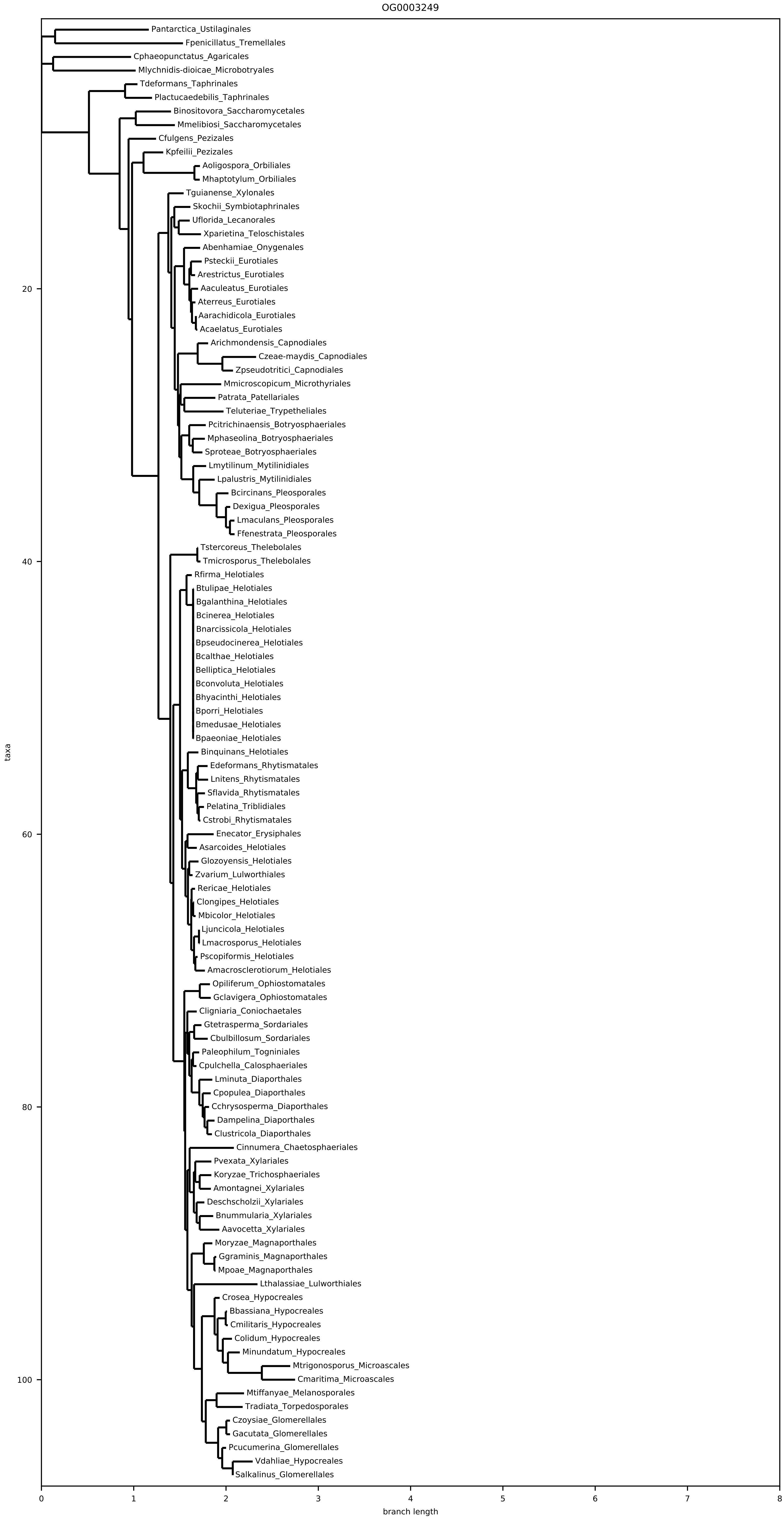

OG0003249
Pantarctica_Ustilaginales
Fpenicillatus_Tremellales
Cphaeopunctatus_Agaricales
Mlychnidis-dioicae_Microbotryales
Tdeformans_Taphrinales
Plactucaedebilis_Taphrinales
Binositovora_Saccharomycetales
Mmelibiosi_Saccharomycetales
Cfulgens_Pezizales
Kpfeilii_Pezizales
Aoligospora_Orbiliales
Mhaptotylum_Orbiliales
Tguianense_Xylonales
Skochii_Symbiotaphrinales
Uflorida_Lecanorales
Xparietina_Teloschistales
Abenhamiae_Onygenales
Psteckii_Eurotiales
Arestrictus_Eurotiales
Aaculeatus_Eurotiales
Aterreus_Eurotiales
Aarachidicola_Eurotiales
Acaelatus_Eurotiales
Arichmondensis_Capnodiales
Czeae-maydis_Capnodiales
Zpseudotritici_Capnodiales
Mmicroscopicum_Microthyriales
Patrata_Patellariales
Teluteriae_Trypetheliales
Pcitrichinaensis_Botryosphaeriales
Mphaseolina_Botryosphaeriales
Sproteae_Botryosphaeriales
Lmytilinum_Mytilinidiales
Lpalustris_Mytilinidiales
Bcircinans_Pleosporales
Dexigua_Pleosporales
Lmaculans_Pleosporales
Ffenestrata_Pleosporales
Tstercoreus_Thelebolales
Tmicrosporus_Thelebolales
Rfirma_Helotiales
Btulipae_Helotiales
Bgalanthina_Helotiales
Bcinerea_Helotiales
Bnarcissicola_Helotiales
Bpseudocinerea_Helotiales
Bcalthae_Helotiales
Belliptica_Helotiales
Bconvoluta_Helotiales
Bhyacinthi_Helotiales
Bporri_Helotiales
Bmedusae_Helotiales
Bpaeoniae_Helotiales
Binquinans_Helotiales
Edeformans_Rhytismatales
Lnitens_Rhytismatales
Sflavida_Rhytismatales
Pelatina_Triblidiales
Cstrobi_Rhytismatales
Enecator_Erysiphales
Asarcoides_Helotiales
Glozoyensis_Helotiales
Zvarium_Lulworthiales
Rericae_Helotiales
Clongipes_Helotiales
Mbicolor_Helotiales
Ljuncicola_Helotiales
Lmacrosporus_Helotiales
Pscopiformis_Helotiales
Amacrosclerotiorum_Helotiales
Opiliferum_Ophiostomatales
Gclavigera_Ophiostomatales
Cligniaria_Coniochaetales
Gtetrasperma_Sordariales
Cbulbillosum_Sordariales
Paleophilum_Togniniales
Cpulchella_Calosphaeriales
Lminuta_Diaporthales
Cpopulea_Diaporthales
Cchrysosperma_Diaporthales
Dampelina_Diaporthales
Clustricola_Diaporthales
Cinnumera_Chaetosphaeriales
Pvexata_Xylariales
Koryzae_Trichosphaeriales
Amontagnei_Xylariales
Deschscholzii_Xylariales
Bnummularia_Xylariales
Aavocetta_Xylariales
Moryzae_Magnaporthales
Ggraminis_Magnaporthales
Mpoae_Magnaporthales
Lthalassiae_Lulworthiales
Crosea_Hypocreales
Bbassiana_Hypocreales
Cmilitaris_Hypocreales
Colidum_Hypocreales
Minundatum_Hypocreales
Mtrigonosporus_Microascales
Cmaritima_Microascales
Mtiffanyae_Melanosporales
Tradiata_Torpedosporales
Czoysiae_Glomerellales
Gacutata_Glomerellales
Pcucumerina_Glomerellales
Vdahliae_Hypocreales
Salkalinus_Glomerellales
taxa
20
40
60
80
100
0
1
2
3
4
5
6
7
8
branch length

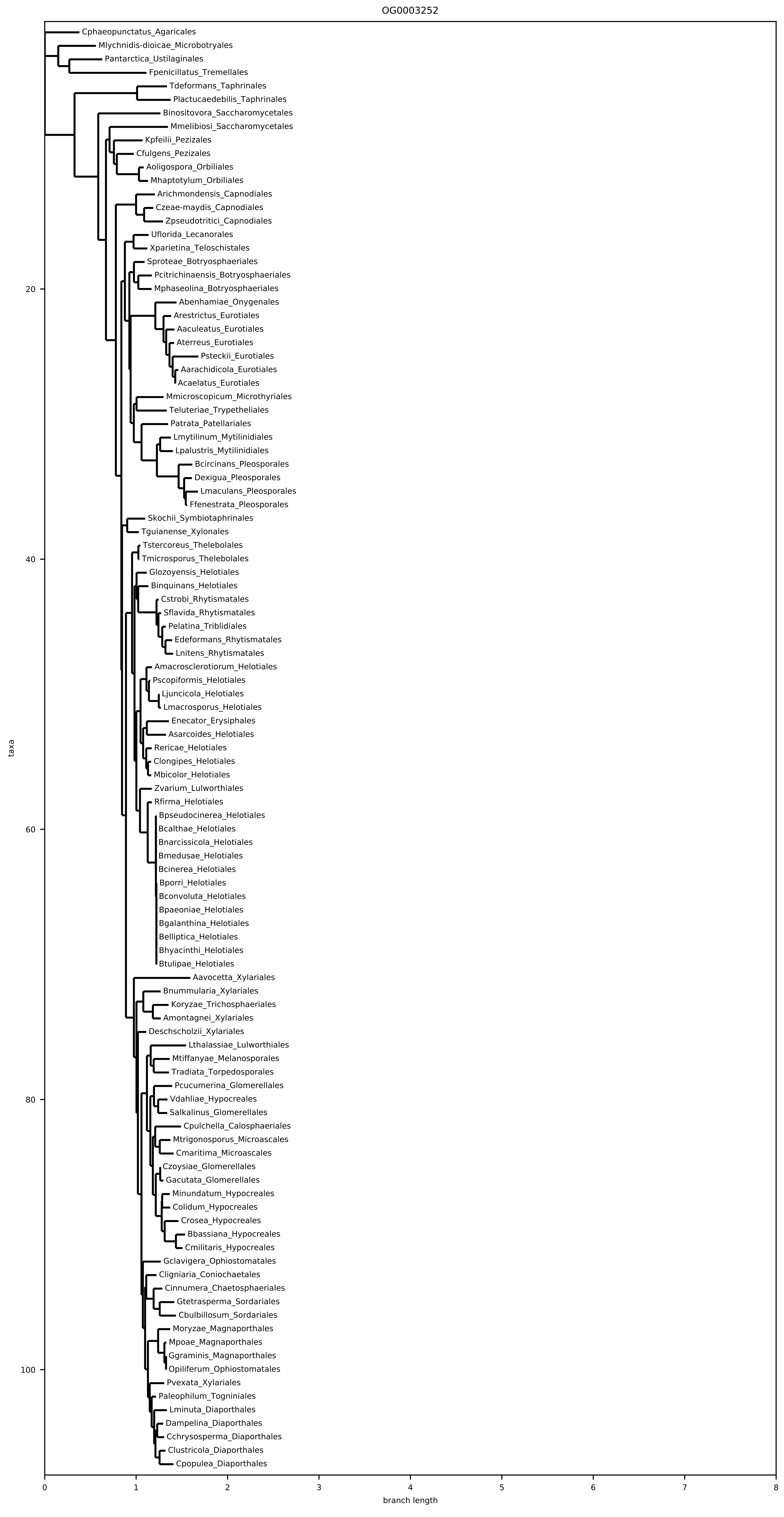

OG0003252
Cphaeopunctatus_Agaricales
Mlychnidis-dioicae_Microbotryales
Pantarctica_Ustilaginales
Fpenicillatus_Tremellales
Tdeformans_Taphrinales
Plactucaedebilis_Taphrinales
Binositovora_Saccharomycetales
Mmelibiosi_Saccharomycetales
Kpfeilii_Pezizales
Cfulgens_Pezizales
Aoligospora_Orbiliales
Mhaptotylum_Orbiliales
Arichmondensis_Capnodiales
Czeae-maydis_Capnodiales
Zpseudotritici_Capnodiales
Uflorida_Lecanorales
Xparietina_Teloschistales
Sproteae_Botryosphaeriales
Pcitrichinaensis_Botryosphaeriales
Mphaseolina_Botryosphaeriales
Abenhamiae_Onygenales
Arestrictus_Eurotiales
Aaculeatus_Eurotiales
Aterreus_Eurotiales
Psteckii_Eurotiales
Aarachidicola_Eurotiales
Acaelatus_Eurotiales
Mmicroscopicum_Microthyriales
Teluteriae_Trypetheliales
Patrata_Patellariales
Lmytilinum_Mytilinidiales
Lpalustris_Mytilinidiales
Bcircinans_Pleosporales
Dexigua_Pleosporales
Lmaculans_Pleosporales
Ffenestrata_Pleosporales
Skochii_Symbiotaphrinales
Tguianense_Xylonales
Tstercoreus_Thelebolales
Tmicrosporus_Thelebolales
Glozoyensis_Helotiales
Binquinans_Helotiales
Cstrobi_Rhytismatales
Sflavida_Rhytismatales
Pelatina_Triblidiales
Edeformans_Rhytismatales
Lnitens_Rhytismatales
Amacrosclerotiorum_Helotiales
Pscopiformis_Helotiales
Ljuncicola_Helotiales
Lmacrosporus_Helotiales
Enecator_Erysiphales
Asarcoides_Helotiales
Rericae_Helotiales
Clongipes_Helotiales
Mbicolor_Helotiales
Zvarium_Lulworthiales
Rfirma_Helotiales
Bpseudocinerea_Helotiales
Bcalthae_Helotiales
Bnarcissicola_Helotiales
Bmedusae_Helotiales
Bcinerea_Helotiales
Bporri_Helotiales
Bconvoluta_Helotiales
Bpaeoniae_Helotiales
Bgalanthina_Helotiales
Belliptica_Helotiales
Bhyacinthi_Helotiales
Btulipae_Helotiales
Aavocetta_Xylariales
Bnummularia_Xylariales
Koryzae_Trichosphaeriales
Amontagnei_Xylariales
Deschscholzii_Xylariales
Lthalassiae_Lulworthiales
Mtiffanyae_Melanosporales
Tradiata_Torpedosporales
Pcucumerina_Glomerellales
Vdahliae_Hypocreales
Salkalinus_Glomerellales
Cpulchella_Calosphaeriales
Mtrigonosporus_Microascales
Cmaritima_Microascales
Czoysiae_Glomerellales
Gacutata_Glomerellales
Minundatum_Hypocreales
Colidum_Hypocreales
Crosea_Hypocreales
Bbassiana_Hypocreales
Cmilitaris_Hypocreales
Gclavigera_Ophiostomatales
Cligniaria_Coniochaetales
Cinnumera_Chaetosphaeriales
Gtetrasperma_Sordariales
Cbulbillosum_Sordariales
Moryzae_Magnaporthales
Mpoae_Magnaporthales
Ggraminis_Magnaporthales
Opiliferum_Ophiostomatales
Pvexata_Xylariales
Paleophilum_Togniniales
Lminuta_Diaporthales
Dampelina_Diaporthales
Cchrysosperma_Diaporthales
Clustricola_Diaporthales
Cpopulea_Diaporthales
taxa
20
40
60
80
100
0
1
2
3
4
5
6
7
8
branch length

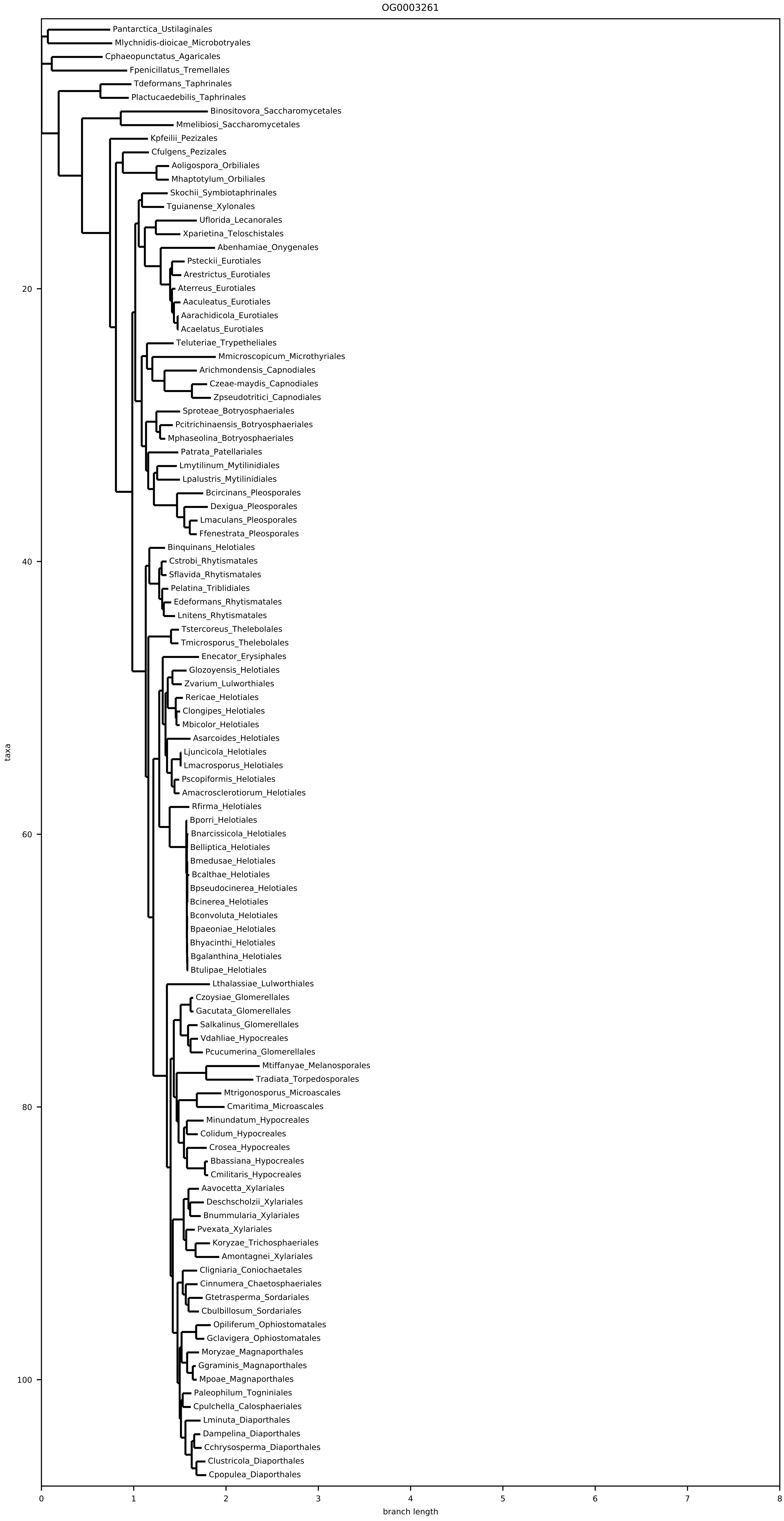

OG0003261
taxa
branch length
Pantarctica_Ustilaginales
Mlychnidis-dioicae_Microbotryales
Cphaeopunctatus_Agaricales
Fpenicillatus_Tremellales
Tdeformans_Taphrinales
Plactucaedebilis_Taphrinales
Binositovora_Saccharomycetales
Mmelibiosi_Saccharomycetales
Kpfeilii_Pezizales
Cfulgens_Pezizales
Aoligospora_Orbiliales
Mhaptotylum_Orbiliales
Skochii_Symbiotaphrinales
Tguianense_Xylonales
Uflorida_Lecanorales
Xparietina_Teloschistales
Abenhamiae_Onygenales
Psteckii_Eurotiales
Arestrictus_Eurotiales
Aterreus_Eurotiales
Aaculeatus_Eurotiales
Aarachidicola_Eurotiales
Acaelatus_Eurotiales
Teluteriae_Trypetheliales
Mmicroscopicum_Microthyriales
Arichmondensis_Capnodiales
Czeae-maydis_Capnodiales
Zpseudotritici_Capnodiales
Sproteae_Botryosphaeriales
Pcitrichinaensis_Botryosphaeriales
Mphaseolina_Botryosphaeriales
Patrata_Patellariales
Lmytilinum_Mytilinidiales
Lpalustris_Mytilinidiales
Bcircinans_Pleosporales
Dexigua_Pleosporales
Lmaculans_Pleosporales
Ffenestrata_Pleosporales
Binquinans_Helotiales
Cstrobi_Rhytismatales
Sflavida_Rhytismatales
Pelatina_Triblidiales
Edeformans_Rhytismatales
Lnitens_Rhytismatales
Tstercoreus_Thelebolales
Tmicrosporus_Thelebolales
Enecator_Erysiphales
Glozoyensis_Helotiales
Zvarium_Lulworthiales
Rericae_Helotiales
Clongipes_Helotiales
Mbicolor_Helotiales
Asarcoides_Helotiales
Ljuncicola_Helotiales
Lmacrosporus_Helotiales
Pscopiformis_Helotiales
Amacrosclerotiorum_Helotiales
Rfirma_Helotiales
Bporri_Helotiales
Bnarcissicola_Helotiales
Belliptica_Helotiales
Bmedusae_Helotiales
Bcalthae_Helotiales
Bpseudocinerea_Helotiales
Bcinerea_Helotiales
Bconvoluta_Helotiales
Bpaeoniae_Helotiales
Bhyacinthi_Helotiales
Bgalanthina_Helotiales
Btulipae_Helotiales
Lthalassiae_Lulworthiales
Czoysiae_Glomerellales
Gacutata_Glomerellales
Salkalinus_Glomerellales
Vdahliae_Hypocreales
Pcucumerina_Glomerellales
Mtiffanyae_Melanosporales
Tradiata_Torpedosporales
Mtrigonosporus_Microascales
Cmaritima_Microascales
Minundatum_Hypocreales
Colidum_Hypocreales
Crosea_Hypocreales
Bbassiana_Hypocreales
Cmilitaris_Hypocreales
Aavocetta_Xylariales
Deschscholzii_Xylariales
Bnummularia_Xylariales
Pvexata_Xylariales
Koryzae_Trichosphaeriales
Amontagnei_Xylariales
Cligniaria_Coniochaetales
Cinnumera_Chaetosphaeriales
Gtetrasperma_Sordariales
Cbulbillosum_Sordariales
Opiliferum_Ophiostomatales
Gclavigera_Ophiostomatales
Moryzae_Magnaporthales
Ggraminis_Magnaporthales
Mpoae_Magnaporthales
Paleophilum_Togniniales
Cpulchella_Calosphaeriales
Lminuta_Diaporthales
Dampelina_Diaporthales
Cchrysosperma_Diaporthales
Clustricola_Diaporthales
Cpopulea_Diaporthales
20
40
60
80
100
0
1
2
3
4
5
6
7
8

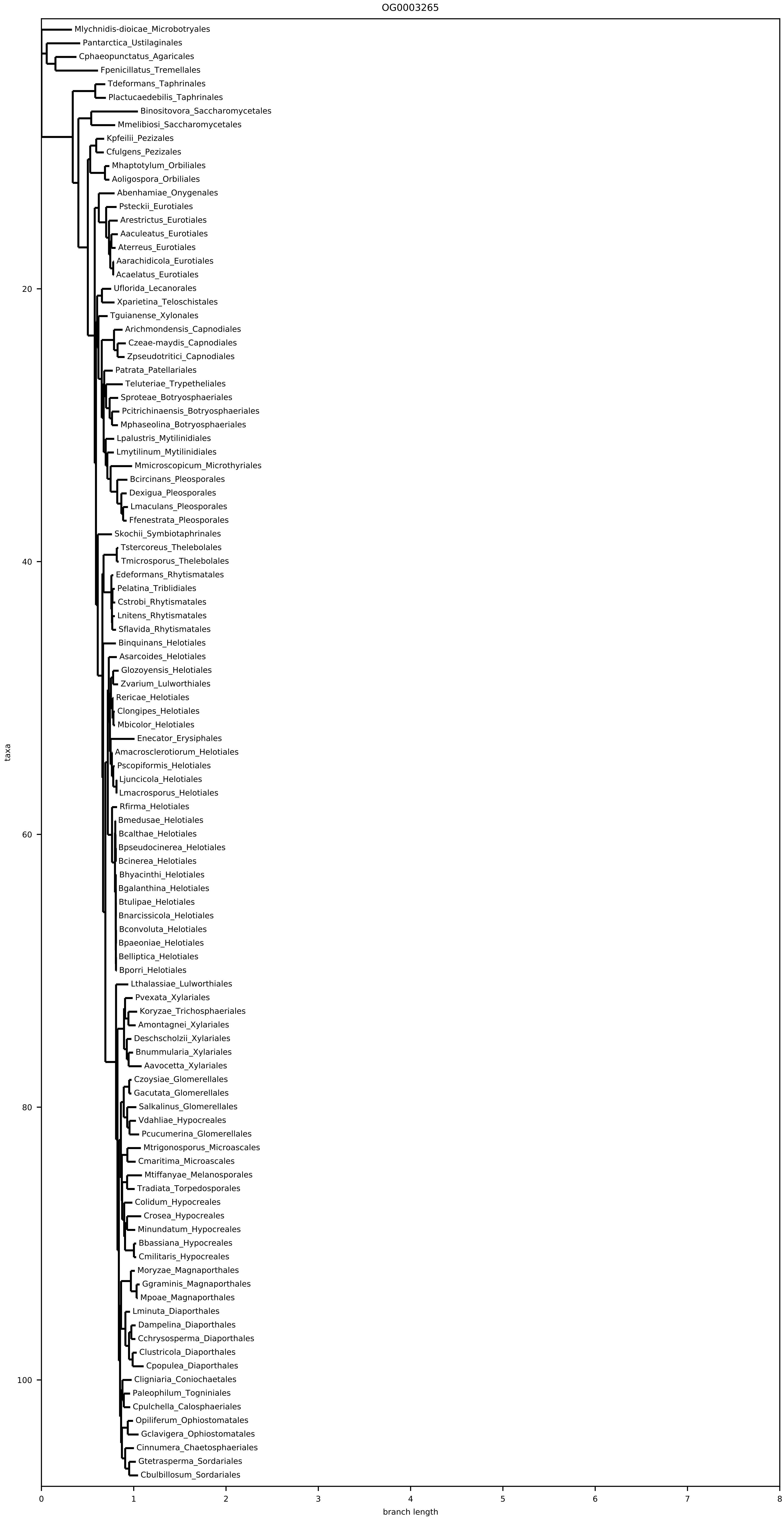
OG0003265
Mlychnidis-dioicae_Microbotryales
Pantarctica_Ustilaginales
Cphaeopunctatus_Agaricales
Fpenicillatus_Tremellales
Tdeformans_Taphrinales
Plactucaedebilis_Taphrinales
Binositovora_Saccharomycetales
Mmelibiosi_Saccharomycetales
Kpfeilii_Pezizales
Cfulgens_Pezizales
Mhaptotylum_Orbiliales
Aoligospora_Orbiliales
Abenhamiae_Onygenales
Psteckii_Eurotiales
Arestrictus_Eurotiales
Aaculeatus_Eurotiales
Aterreus_Eurotiales
Aarachidicola_Eurotiales
Acaelatus_Eurotiales
Uflorida_Lecanorales
Xparietina_Teloschistales
Tguianense_Xylonales
Arichmondensis_Capnodiales
Czeae-maydis_Capnodiales
Zpseudotritici_Capnodiales
Patrata_Patellariales
Teluteriae_Trypetheliales
Sproteae_Botryosphaeriales
Pcitrichinaensis_Botryosphaeriales
Mphaseolina_Botryosphaeriales
Lpalustris_Mytilinidiales
Lmytilinum_Mytilinidiales
Mmicroscopicum_Microthyriales
Bcircinans_Pleosporales
Dexigua_Pleosporales
Lmaculans_Pleosporales
Ffenestrata_Pleosporales
Skochii_Symbiotaphrinales
Tstercoreus_Thelebolales
Tmicrosporus_Thelebolales
Edeformans_Rhytismatales
Pelatina_Triblidiales
Cstrobi_Rhytismatales
Lnitens_Rhytismatales
Sflavida_Rhytismatales
Binquinans_Helotiales
Asarcoides_Helotiales
Glozoyensis_Helotiales
Zvarium_Lulworthiales
Rericae_Helotiales
Clongipes_Helotiales
Mbicolor_Helotiales
Enecator_Erysiphales
Amacrosclerotiorum_Helotiales
Pscopiformis_Helotiales
Ljuncicola_Helotiales
Lmacrosporus_Helotiales
Rfirma_Helotiales
Bmedusae_Helotiales
Bcalthae_Helotiales
Bpseudocinerea_Helotiales
Bcinerea_Helotiales
Bhyacinthi_Helotiales
Bgalanthina_Helotiales
Btulipae_Helotiales
Bnarcissicola_Helotiales
Bconvoluta_Helotiales
Bpaeoniae_Helotiales
Belliptica_Helotiales
Bporri_Helotiales
Lthalassiae_Lulworthiales
Pvexata_Xylariales
Koryzae_Trichosphaeriales
Amontagnei_Xylariales
Deschscholzii_Xylariales
Bnummularia_Xylariales
Aavocetta_Xylariales
Czoysiae_Glomerellales
Gacutata_Glomerellales
Salkalinus_Glomerellales
Vdahliae_Hypocreales
Pcucumerina_Glomerellales
Mtrigonosporus_Microascales
Cmaritima_Microascales
Mtiffanyae_Melanosporales
Tradiata_Torpedosporales
Colidum_Hypocreales
Crosea_Hypocreales
Minundatum_Hypocreales
Bbassiana_Hypocreales
Cmilitaris_Hypocreales
Moryzae_Magnaporthales
Ggraminis_Magnaporthales
Mpoae_Magnaporthales
Lminuta_Diaporthales
Dampelina_Diaporthales
Cchrysosperma_Diaporthales
Clustricola_Diaporthales
Cpopulea_Diaporthales
Cligniaria_Coniochaetales
Paleophilum_Togniniales
Cpulchella_Calosphaeriales
Opiliferum_Ophiostomatales
Gclavigera_Ophiostomatales
Cinnumera_Chaetosphaeriales
Gtetrasperma_Sordariales
Cbulbillosum_Sordariales
taxa
20
40
60
80
100
0
1
2
3
4
5
6
7
8
branch length

OG0003266

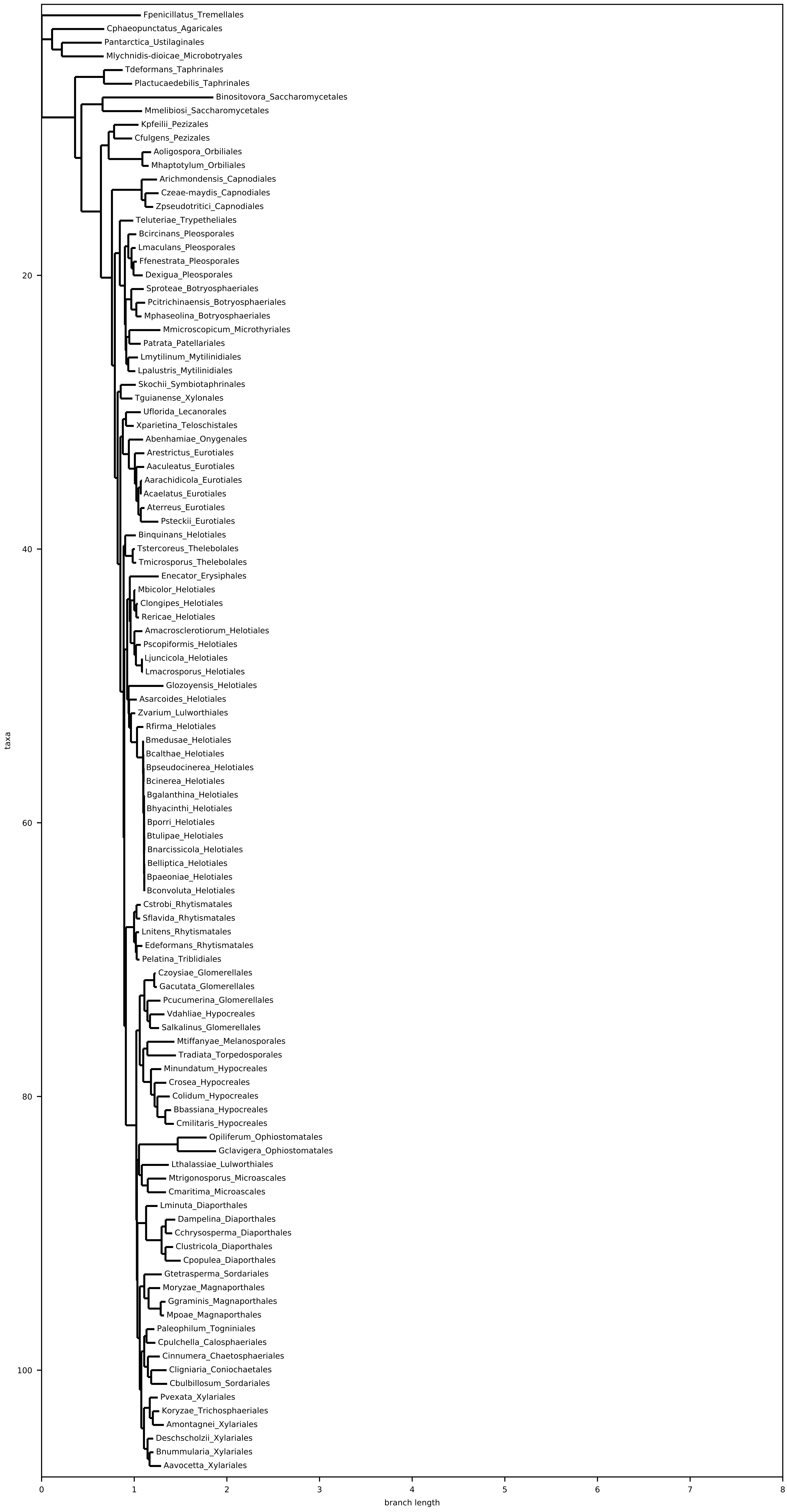
Fpenicillatus_Tremellales
Cphaeopunctatus_Agaricales
Pantarctica_Ustilaginales
Mlychnidis-dioicae_Microbotryales
Tdeformans_Taphrinales
Plactucaedebilis_Taphrinales
Binositovora_Saccharomycetales
Mmelibiosi_Saccharomycetales
Kpfeilii_Pezizales
Cfulgens_Pezizales
Aoligospora_Orbiliales
Mhaptotylum_Orbiliales
Arichmondensis_Capnodiales
Czeae-maydis_Capnodiales
Zpseudotritici_Capnodiales
Teluteriae_Trypetheliales
Bcircinans_Pleosporales
Lmaculans_Pleosporales
Ffenestrata_Pleosporales
Dexigua_Pleosporales
Sproteae_Botryosphaeriales
Pcitrichinaensis_Botryosphaeriales
Mphaseolina_Botryosphaeriales
Mmicroscopicum_Microthyriales
Patrata_Patellariales
Lmytilinum_Mytilinidiales
Lpalustris_Mytilinidiales
Skochii_Symbiotaphrinales
Tguianense_Xylonales
Uflorida_Lecanorales
Xparietina_Teloschistales
Abenhamiae_Onygenales
Arestrictus_Eurotiales
Aaculeatus_Eurotiales
Aarachidicola_Eurotiales
Acaelatus_Eurotiales
Aterreus_Eurotiales
Psteckii_Eurotiales
Binquinans_Helotiales
Tstercoreus_Thelebolales
Tmicrosporus_Thelebolales
Enecator_Erysiphales
Mbicolor_Helotiales
Clongipes_Helotiales
Rericae_Helotiales
Amacrosclerotiorum_Helotiales
Pscopiformis_Helotiales
Ljuncicola_Helotiales
Lmacrosporus_Helotiales
Glozoyensis_Helotiales
Asarcoides_Helotiales
Zvarium_Lulworthiales
Rfirma_Helotiales
Bmedusae_Helotiales
Bcalthae_Helotiales
Bpseudocinerea_Helotiales
Bcinerea_Helotiales
Bgalanthina_Helotiales
Bhyacinthi_Helotiales
Bporri_Helotiales
Btulipae_Helotiales
Bnarcissicola_Helotiales
Belliptica_Helotiales
Bpaeoniae_Helotiales
Bconvoluta_Helotiales
Cstrobi_Rhytismatales
Sflavida_Rhytismatales
Lnitens_Rhytismatales
Edeformans_Rhytismatales
Pelatina_Triblidiales
Czoysiae_Glomerellales
Gacutata_Glomerellales
Pcucumerina_Glomerellales
Vdahliae_Hypocreales
Salkalinus_Glomerellales
Mtiffanyae_Melanosporales
Tradiata_Torpedosporales
Minundatum_Hypocreales
Crosea_Hypocreales
Colidum_Hypocreales
Bbassiana_Hypocreales
Cmilitaris_Hypocreales
Opiliferum_Ophiostomatales
Gclavigera_Ophiostomatales
Lthalassiae_Lulworthiales
Mtrigonosporus_Microascales
Cmaritima_Microascales
Lminuta_Diaporthales
Dampelina_Diaporthales
Cchrysosperma_Diaporthales
Clustricola_Diaporthales
Cpopulea_Diaporthales
Gtetrasperma_Sordariales
Moryzae_Magnaporthales
Ggraminis_Magnaporthales
Mpoae_Magnaporthales
Paleophilum_Togniniales
Cpulchella_Calosphaeriales
Cinnumera_Chaetosphaeriales
Cligniaria_Coniochaetales
Cbulbillosum_Sordariales
Pvexata_Xylariales
Koryzae_Trichosphaeriales
Amontagnei_Xylariales
Deschscholzii_Xylariales
Bnummularia_Xylariales
Aavocetta_Xylariales
taxa
20
40
60
80
100
0
1
2
3
4
5
6
7
8
branch length

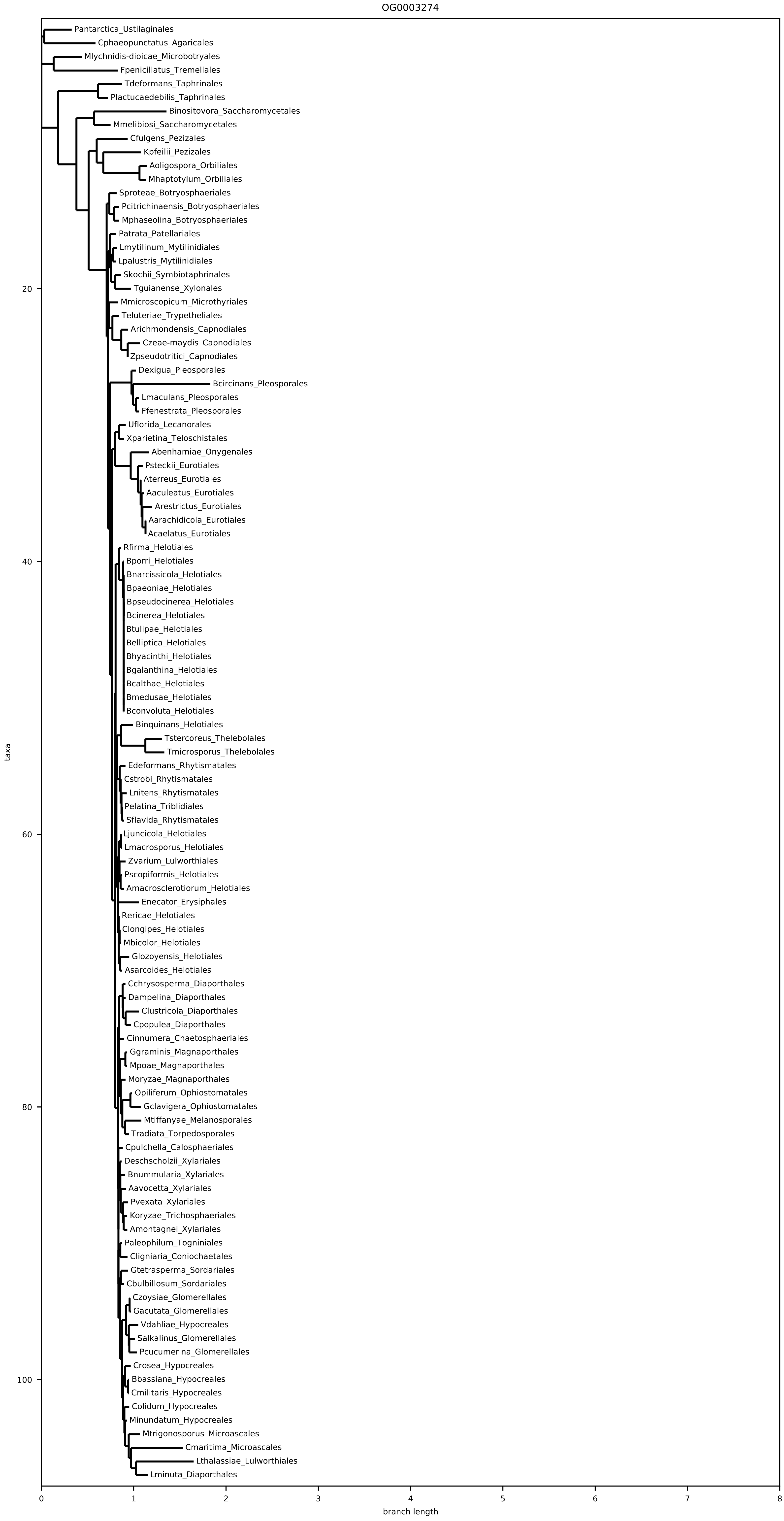
OG0003274
Pantarctica_Ustilaginales
Cphaeopunctatus_Agaricales
Mlychnidis-dioicae_Microbotryales
Fpenicillatus_Tremellales
Tdeformans_Taphrinales
Plactucaedebilis_Taphrinales
Binositovora_Saccharomycetales
Mmelibiosi_Saccharomycetales
Cfulgens_Pezizales
Kpfeilii_Pezizales
Aoligospora_Orbiliales
Mhaptotylum_Orbiliales
Sproteae_Botryosphaeriales
Pcitrichinaensis_Botryosphaeriales
Mphaseolina_Botryosphaeriales
Patrata_Patellariales
Lmytilinum_Mytilinidiales
Lpalustris_Mytilinidiales
Skochii_Symbiotaphrinales
Tguianense_Xylonales
Mmicroscopicum_Microthyriales
Teluteriae_Trypetheliales
Arichmondensis_Capnodiales
Czeae-maydis_Capnodiales
Zpseudotritici_Capnodiales
Dexigua_Pleosporales
Bcircinans_Pleosporales
Lmaculans_Pleosporales
Ffenestrata_Pleosporales
Uflorida_Lecanorales
Xparietina_Teloschistales
Abenhamiae_Onygenales
Psteckii_Eurotiales
Aterreus_Eurotiales
Aaculeatus_Eurotiales
Arestrictus_Eurotiales
Aarachidicola_Eurotiales
Acaelatus_Eurotiales
Rfirma_Helotiales
Bporri_Helotiales
Bnarcissicola_Helotiales
Bpaeoniae_Helotiales
Bpseudocinerea_Helotiales
Bcinerea_Helotiales
Btulipae_Helotiales
Belliptica_Helotiales
Bhyacinthi_Helotiales
Bgalanthina_Helotiales
Bcalthae_Helotiales
Bmedusae_Helotiales
Bconvoluta_Helotiales
Binquinans_Helotiales
Tstercoreus_Thelebolales
Tmicrosporus_Thelebolales
Edeformans_Rhytismatales
Cstrobi_Rhytismatales
Lnitens_Rhytismatales
Pelatina_Triblidiales
Sflavida_Rhytismatales
Ljuncicola_Helotiales
Lmacrosporus_Helotiales
Zvarium_Lulworthiales
Pscopiformis_Helotiales
Amacrosclerotiorum_Helotiales
Enecator_Erysiphales
Rericae_Helotiales
Clongipes_Helotiales
Mbicolor_Helotiales
Glozoyensis_Helotiales
Asarcoides_Helotiales
Cchrysosperma_Diaporthales
Dampelina_Diaporthales
Clustricola_Diaporthales
Cpopulea_Diaporthales
Cinnumera_Chaetosphaeriales
Ggraminis_Magnaporthales
Mpoae_Magnaporthales
Moryzae_Magnaporthales
Opiliferum_Ophiostomatales
Gclavigera_Ophiostomatales
Mtiffanyae_Melanosporales
Tradiata_Torpedosporales
Cpulchella_Calosphaeriales
Deschscholzii_Xylariales
Bnummularia_Xylariales
Aavocetta_Xylariales
Pvexata_Xylariales
Koryzae_Trichosphaeriales
Amontagnei_Xylariales
Paleophilum_Togniniales
Cligniaria_Coniochaetales
Gtetrasperma_Sordariales
Cbulbillosum_Sordariales
Czoysiae_Glomerellales
Gacutata_Glomerellales
Vdahliae_Hypocreales
Salkalinus_Glomerellales
Pcucumerina_Glomerellales
Crosea_Hypocreales
Bbassiana_Hypocreales
Cmilitaris_Hypocreales
Colidum_Hypocreales
Minundatum_Hypocreales
Mtrigonosporus_Microascales
Cmaritima_Microascales
Lthalassiae_Lulworthiales
Lminuta_Diaporthales
taxa
20
40
60
80
100
0
1
2
3
4
5
6
7
8
branch length

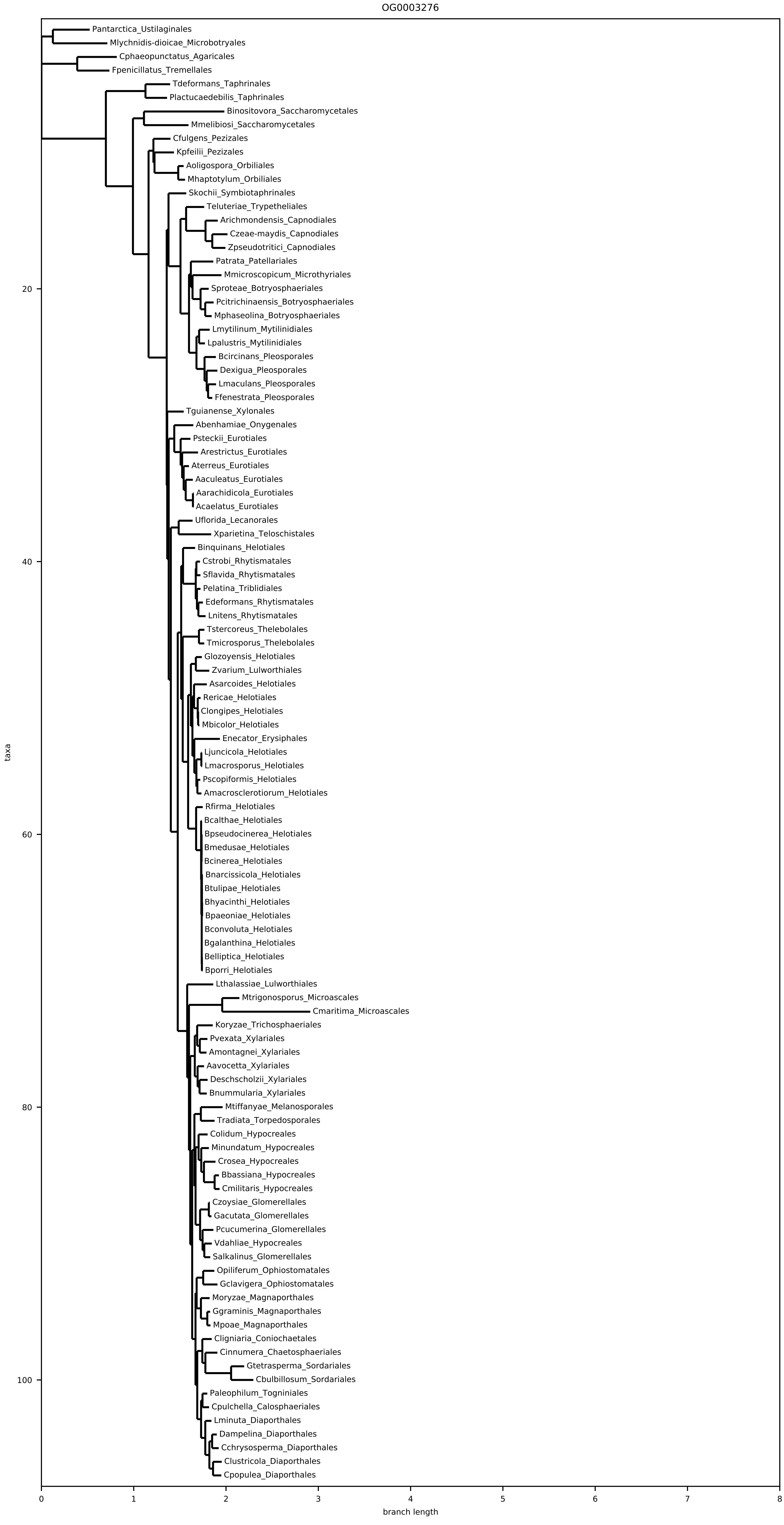
OG0003276
Pantarctica_Ustilaginales
Mlychnidis-dioicae_Microbotryales
Cphaeopunctatus_Agaricales
Fpenicillatus_Tremellales
Tdeformans_Taphrinales
Plactucaedebilis_Taphrinales
Binositovora_Saccharomycetales
Mmelibiosi_Saccharomycetales
Cfulgens_Pezizales
Kpfeilii_Pezizales
Aoligospora_Orbiliales
Mhaptotylum_Orbiliales
Skochii_Symbiotaphrinales
Teluteriae_Trypetheliales
Arichmondensis_Capnodiales
Czeae-maydis_Capnodiales
Zpseudotritici_Capnodiales
Patrata_Patellariales
Mmicroscopicum_Microthyriales
Sproteae_Botryosphaeriales
Pcitrichinaensis_Botryosphaeriales
Mphaseolina_Botryosphaeriales
Lmytilinum_Mytilinidiales
Lpalustris_Mytilinidiales
Bcircinans_Pleosporales
Dexigua_Pleosporales
Lmaculans_Pleosporales
Ffenestrata_Pleosporales
Tguianense_Xylonales
Abenhamiae_Onygenales
Psteckii_Eurotiales
Arestrictus_Eurotiales
Aterreus_Eurotiales
Aaculeatus_Eurotiales
Aarachidicola_Eurotiales
Acaelatus_Eurotiales
Uflorida_Lecanorales
Xparietina_Teloschistales
Binquinans_Helotiales
Cstrobi_Rhytismatales
Sflavida_Rhytismatales
Pelatina_Triblidiales
Edeformans_Rhytismatales
Lnitens_Rhytismatales
Tstercoreus_Thelebolales
Tmicrosporus_Thelebolales
Glozoyensis_Helotiales
Zvarium_Lulworthiales
Asarcoides_Helotiales
Rericae_Helotiales
Clongipes_Helotiales
Mbicolor_Helotiales
Enecator_Erysiphales
Ljuncicola_Helotiales
Lmacrosporus_Helotiales
Pscopiformis_Helotiales
Amacrosclerotiorum_Helotiales
Rfirma_Helotiales
Bcalthae_Helotiales
Bpseudocinerea_Helotiales
Bmedusae_Helotiales
Bcinerea_Helotiales
Bnarcissicola_Helotiales
Btulipae_Helotiales
Bhyacinthi_Helotiales
Bpaeoniae_Helotiales
Bconvoluta_Helotiales
Bgalanthina_Helotiales
Belliptica_Helotiales
Bporri_Helotiales
Lthalassiae_Lulworthiales
Mtrigonosporus_Microascales
Cmaritima_Microascales
Koryzae_Trichosphaeriales
Pvexata_Xylariales
Amontagnei_Xylariales
Aavocetta_Xylariales
Deschscholzii_Xylariales
Bnummularia_Xylariales
Mtiffanyae_Melanosporales
Tradiata_Torpedosporales
Colidum_Hypocreales
Minundatum_Hypocreales
Crosea_Hypocreales
Bbassiana_Hypocreales
Cmilitaris_Hypocreales
Czoysiae_Glomerellales
Gacutata_Glomerellales
Pcucumerina_Glomerellales
Vdahliae_Hypocreales
Salkalinus_Glomerellales
Opiliferum_Ophiostomatales
Gclavigera_Ophiostomatales
Moryzae_Magnaporthales
Ggraminis_Magnaporthales
Mpoae_Magnaporthales
Cligniaria_Coniochaetales
Cinnumera_Chaetosphaeriales
Gtetrasperma_Sordariales
Cbulbillosum_Sordariales
Paleophilum_Togniniales
Cpulchella_Calosphaeriales
Lminuta_Diaporthales
Dampelina_Diaporthales
Cchrysosperma_Diaporthales
Clustricola_Diaporthales
Cpopulea_Diaporthales
taxa
20
40
60
80
100
0
1
2
3
4
5
6
7
8
branch length

OG0003281

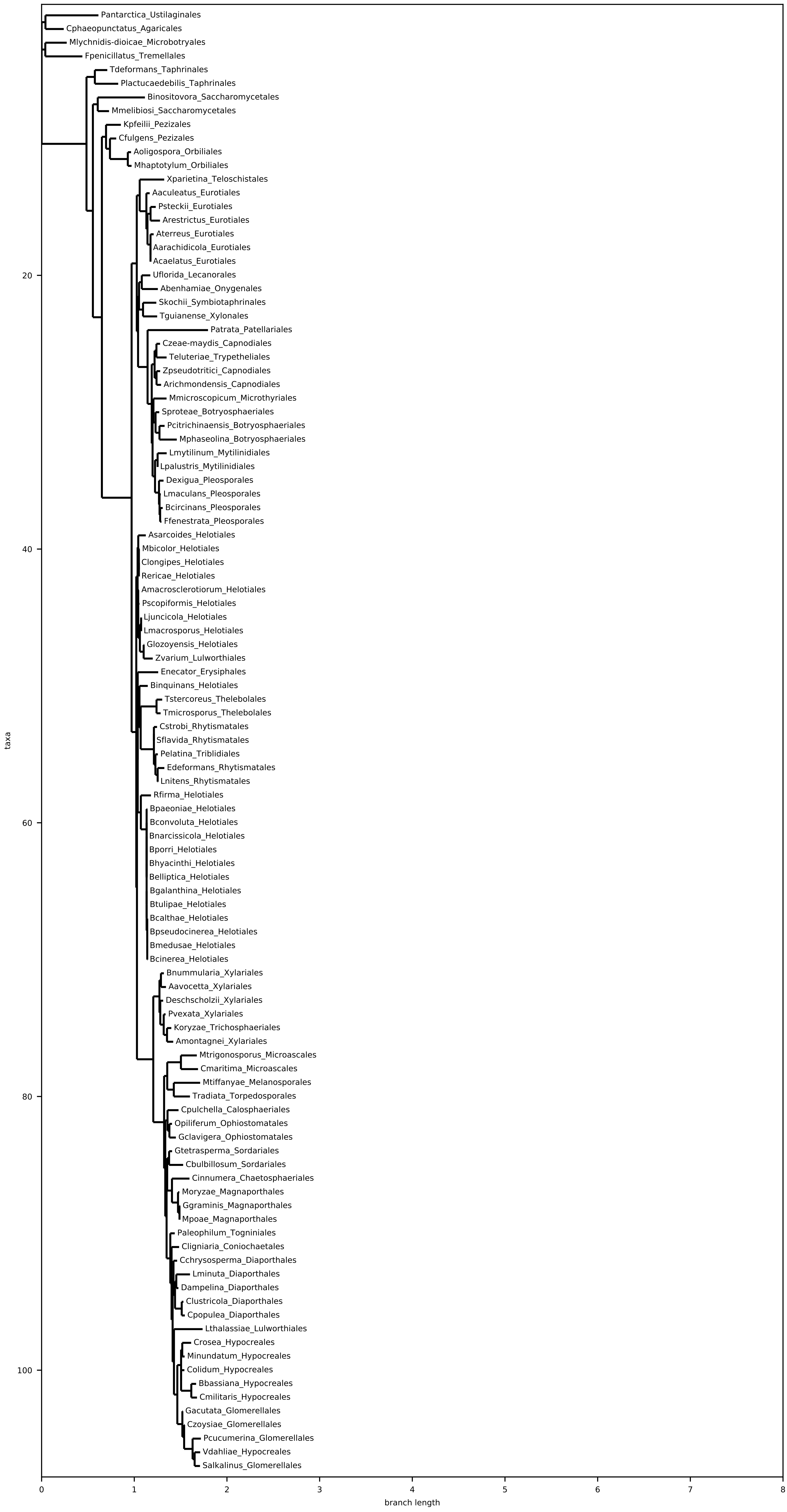
Pantarctica_Ustilaginales
Cphaeopunctatus_Agaricales
Mlychnidis-dioicae_Microbotryales
Fpenicillatus_Tremellales
Tdeformans_Taphrinales
Plactucaedebilis_Taphrinales
Binositovora_Saccharomycetales
Mmelibiosi_Saccharomycetales
Kpfeilii_Pezizales
Cfulgens_Pezizales
Aoligospora_Orbiliales
Mhaptotylum_Orbiliales
Xparietina_Teloschistales
Aaculeatus_Eurotiales
Psteckii_Eurotiales
Arestrictus_Eurotiales
Aterreus_Eurotiales
Aarachidicola_Eurotiales
Acaelatus_Eurotiales
Uflorida_Lecanorales
Abenhamiae_Onygenales
Skochii_Symbiotaphrinales
Tguianense_Xylonales
Patrata_Patellariales
Czeae-maydis_Capnodiales
Teluteriae_Trypetheliales
Zpseudotritici_Capnodiales
Arichmondensis_Capnodiales
Mmicroscopicum_Microthyriales
Sproteae_Botryosphaeriales
Pcitrichinaensis_Botryosphaeriales
Mphaseolina_Botryosphaeriales
Lmytilinum_Mytilinidiales
Lpalustris_Mytilinidiales
Dexigua_Pleosporales
Lmaculans_Pleosporales
Bcircinans_Pleosporales
Ffenestrata_Pleosporales
Asarcoides_Helotiales
Mbicolor_Helotiales
Clongipes_Helotiales
Rericae_Helotiales
Amacrosclerotiorum_Helotiales
Pscopiformis_Helotiales
Ljuncicola_Helotiales
Lmacrosporus_Helotiales
Glozoyensis_Helotiales
Zvarium_Lulworthiales
Enecator_Erysiphales
Binquinans_Helotiales
Tstercoreus_Thelebolales
Tmicrosporus_Thelebolales
Cstrobi_Rhytismatales
Sflavida_Rhytismatales
Pelatina_Triblidiales
Edeformans_Rhytismatales
Lnitens_Rhytismatales
Rfirma_Helotiales
Bpaeoniae_Helotiales
Bconvoluta_Helotiales
Bnarcissicola_Helotiales
Bporri_Helotiales
Bhyacinthi_Helotiales
Belliptica_Helotiales
Bgalanthina_Helotiales
Btulipae_Helotiales
Bcalthae_Helotiales
Bpseudocinerea_Helotiales
Bmedusae_Helotiales
Bcinerea_Helotiales
Bnummularia_Xylariales
Aavocetta_Xylariales
Deschscholzii_Xylariales
Pvexata_Xylariales
Koryzae_Trichosphaeriales
Amontagnei_Xylariales
Mtrigonosporus_Microascales
Cmaritima_Microascales
Mtiffanyae_Melanosporales
Tradiata_Torpedosporales
Cpulchella_Calosphaeriales
Opiliferum_Ophiostomatales
Gclavigera_Ophiostomatales
Gtetrasperma_Sordariales
Cbulbillosum_Sordariales
Cinnumera_Chaetosphaeriales
Moryzae_Magnaporthales
Ggraminis_Magnaporthales
Mpoae_Magnaporthales
Paleophilum_Togniniales
Cligniaria_Coniochaetales
Cchrysosperma_Diaporthales
Lminuta_Diaporthales
Dampelina_Diaporthales
Clustricola_Diaporthales
Cpopulea_Diaporthales
Lthalassiae_Lulworthiales
Crosea_Hypocreales
Minundatum_Hypocreales
Colidum_Hypocreales
Bbassiana_Hypocreales
Cmilitaris_Hypocreales
Gacutata_Glomerellales
Czoysiae_Glomerellales
Pcucumerina_Glomerellales
Vdahliae_Hypocreales
Salkalinus_Glomerellales
taxa
20
40
60
80
100
0
1
2
3
4
5
6
7
8
branch length

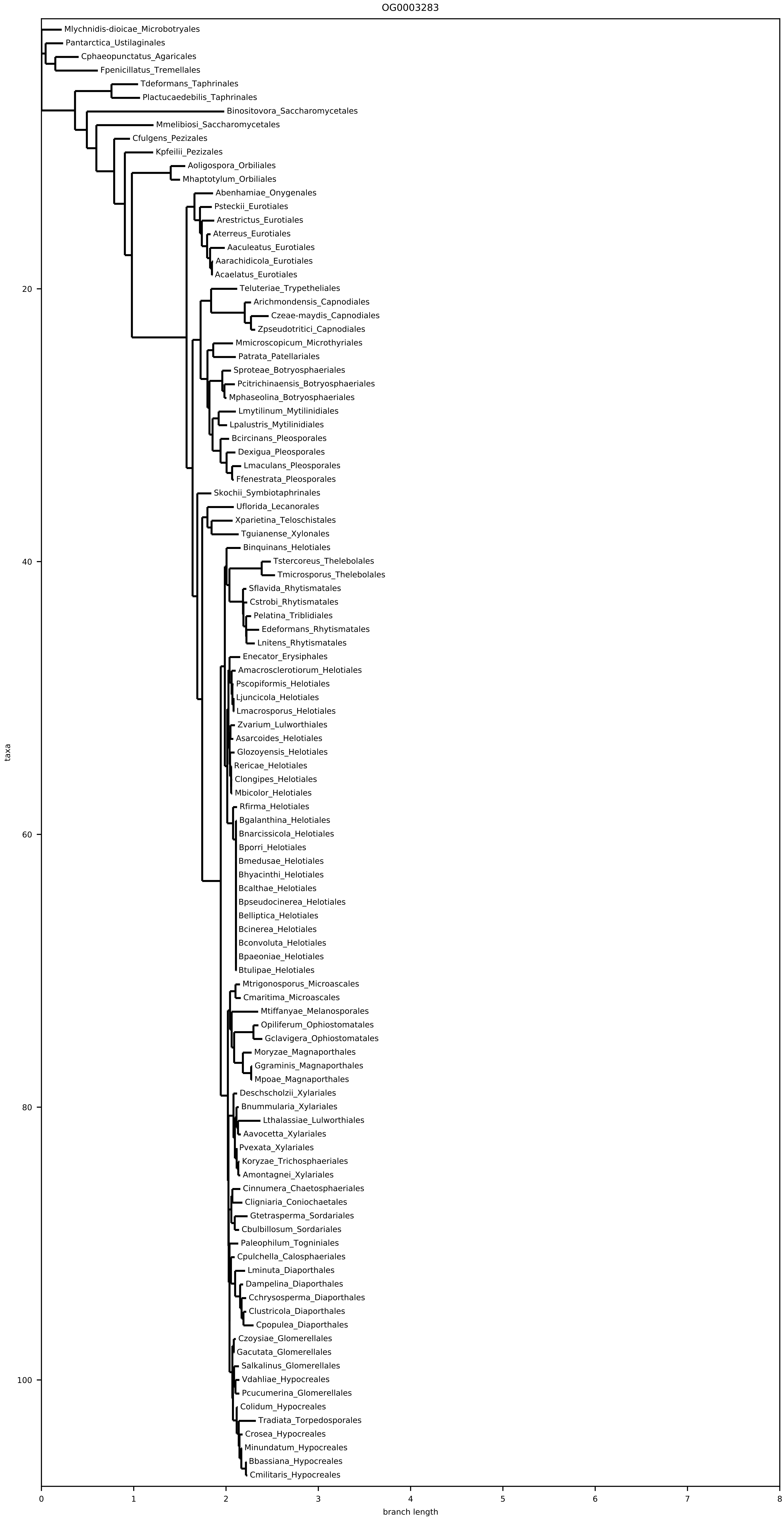
OG0003283
Mlychnidis-dioicae_Microbotryales
Pantarctica_Ustilaginales
Cphaeopunctatus_Agaricales
Fpenicillatus_Tremellales
Tdeformans_Taphrinales
Plactucaedebilis_Taphrinales
Binositovora_Saccharomycetales
Mmelibiosi_Saccharomycetales
Cfulgens_Pezizales
Kpfeilii_Pezizales
Aoligospora_Orbiliales
Mhaptotylum_Orbiliales
Abenhamiae_Onygenales
Psteckii_Eurotiales
Arestrictus_Eurotiales
Aterreus_Eurotiales
Aaculeatus_Eurotiales
Aarachidicola_Eurotiales
Acaelatus_Eurotiales
Teluteriae_Trypetheliales
Arichmondensis_Capnodiales
Czeae-maydis_Capnodiales
Zpseudotritici_Capnodiales
Mmicroscopicum_Microthyriales
Patrata_Patellariales
Sproteae_Botryosphaeriales
Pcitrichinaensis_Botryosphaeriales
Mphaseolina_Botryosphaeriales
Lmytilinum_Mytilinidiales
Lpalustris_Mytilinidiales
Bcircinans_Pleosporales
Dexigua_Pleosporales
Lmaculans_Pleosporales
Ffenestrata_Pleosporales
Skochii_Symbiotaphrinales
Uflorida_Lecanorales
Xparietina_Teloschistales
Tguianense_Xylonales
Binquinans_Helotiales
Tstercoreus_Thelebolales
Tmicrosporus_Thelebolales
Sflavida_Rhytismatales
Cstrobi_Rhytismatales
Pelatina_Triblidiales
Edeformans_Rhytismatales
Lnitens_Rhytismatales
Enecator_Erysiphales
Amacrosclerotiorum_Helotiales
Pscopiformis_Helotiales
Ljuncicola_Helotiales
Lmacrosporus_Helotiales
Zvarium_Lulworthiales
Asarcoides_Helotiales
Glozoyensis_Helotiales
Rericae_Helotiales
Clongipes_Helotiales
Mbicolor_Helotiales
Rfirma_Helotiales
Bgalanthina_Helotiales
Bnarcissicola_Helotiales
Bporri_Helotiales
Bmedusae_Helotiales
Bhyacinthi_Helotiales
Bcalthae_Helotiales
Bpseudocinerea_Helotiales
Belliptica_Helotiales
Bcinerea_Helotiales
Bconvoluta_Helotiales
Bpaeoniae_Helotiales
Btulipae_Helotiales
Mtrigonosporus_Microascales
Cmaritima_Microascales
Mtiffanyae_Melanosporales
Opiliferum_Ophiostomatales
Gclavigera_Ophiostomatales
Moryzae_Magnaporthales
Ggraminis_Magnaporthales
Mpoae_Magnaporthales
Deschscholzii_Xylariales
Bnummularia_Xylariales
Lthalassiae_Lulworthiales
Aavocetta_Xylariales
Pvexata_Xylariales
Koryzae_Trichosphaeriales
Amontagnei_Xylariales
Cinnumera_Chaetosphaeriales
Cligniaria_Coniochaetales
Gtetrasperma_Sordariales
Cbulbillosum_Sordariales
Paleophilum_Togniniales
Cpulchella_Calosphaeriales
Lminuta_Diaporthales
Dampelina_Diaporthales
Cchrysosperma_Diaporthales
Clustricola_Diaporthales
Cpopulea_Diaporthales
Czoysiae_Glomerellales
Gacutata_Glomerellales
Salkalinus_Glomerellales
Vdahliae_Hypocreales
Pcucumerina_Glomerellales
Colidum_Hypocreales
Tradiata_Torpedosporales
Crosea_Hypocreales
Minundatum_Hypocreales
Bbassiana_Hypocreales
Cmilitaris_Hypocreales
taxa
20
40
60
80
100
0
1
2
3
4
5
6
7
8
branch length

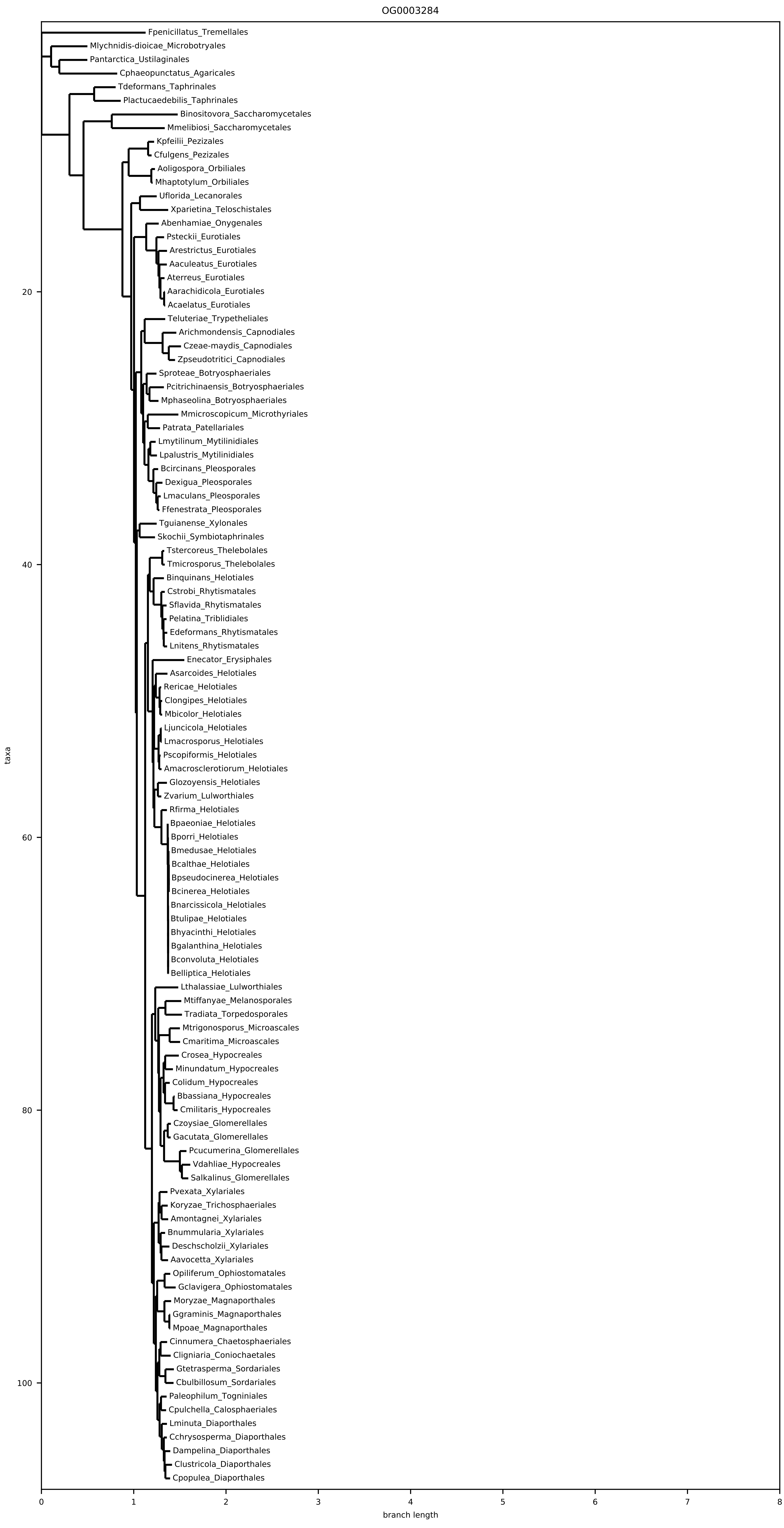
OG0003284
Fpenicillatus_Tremellales
Mlychnidis-dioicae_Microbotryales
Pantarctica_Ustilaginales
Cphaeopunctatus_Agaricales
Tdeformans_Taphrinales
Plactucaedebilis_Taphrinales
Binositovora_Saccharomycetales
Mmelibiosi_Saccharomycetales
Kpfeilii_Pezizales
Cfulgens_Pezizales
Aoligospora_Orbiliales
Mhaptotylum_Orbiliales
Uflorida_Lecanorales
Xparietina_Teloschistales
Abenhamiae_Onygenales
Psteckii_Eurotiales
Arestrictus_Eurotiales
Aaculeatus_Eurotiales
Aterreus_Eurotiales
Aarachidicola_Eurotiales
Acaelatus_Eurotiales
Teluteriae_Trypetheliales
Arichmondensis_Capnodiales
Czeae-maydis_Capnodiales
Zpseudotritici_Capnodiales
Sproteae_Botryosphaeriales
Pcitrichinaensis_Botryosphaeriales
Mphaseolina_Botryosphaeriales
Mmicroscopicum_Microthyriales
Patrata_Patellariales
Lmytilinum_Mytilinidiales
Lpalustris_Mytilinidiales
Bcircinans_Pleosporales
Dexigua_Pleosporales
Lmaculans_Pleosporales
Ffenestrata_Pleosporales
Tguianense_Xylonales
Skochii_Symbiotaphrinales
Tstercoreus_Thelebolales
Tmicrosporus_Thelebolales
Binquinans_Helotiales
Cstrobi_Rhytismatales
Sflavida_Rhytismatales
Pelatina_Triblidiales
Edeformans_Rhytismatales
Lnitens_Rhytismatales
Enecator_Erysiphales
Asarcoides_Helotiales
Rericae_Helotiales
Clongipes_Helotiales
Mbicolor_Helotiales
Ljuncicola_Helotiales
Lmacrosporus_Helotiales
Pscopiformis_Helotiales
Amacrosclerotiorum_Helotiales
Glozoyensis_Helotiales
Zvarium_Lulworthiales
Rfirma_Helotiales
Bpaeoniae_Helotiales
Bporri_Helotiales
Bmedusae_Helotiales
Bcalthae_Helotiales
Bpseudocinerea_Helotiales
Bcinerea_Helotiales
Bnarcissicola_Helotiales
Btulipae_Helotiales
Bhyacinthi_Helotiales
Bgalanthina_Helotiales
Bconvoluta_Helotiales
Belliptica_Helotiales
Lthalassiae_Lulworthiales
Mtiffanyae_Melanosporales
Tradiata_Torpedosporales
Mtrigonosporus_Microascales
Cmaritima_Microascales
Crosea_Hypocreales
Minundatum_Hypocreales
Colidum_Hypocreales
Bbassiana_Hypocreales
Cmilitaris_Hypocreales
Czoysiae_Glomerellales
Gacutata_Glomerellales
Pcucumerina_Glomerellales
Vdahliae_Hypocreales
Salkalinus_Glomerellales
Pvexata_Xylariales
Koryzae_Trichosphaeriales
Amontagnei_Xylariales
Bnummularia_Xylariales
Deschscholzii_Xylariales
Aavocetta_Xylariales
Opiliferum_Ophiostomatales
Gclavigera_Ophiostomatales
Moryzae_Magnaporthales
Ggraminis_Magnaporthales
Mpoae_Magnaporthales
Cinnumera_Chaetosphaeriales
Cligniaria_Coniochaetales
Gtetrasperma_Sordariales
Cbulbillosum_Sordariales
Paleophilum_Togniniales
Cpulchella_Calosphaeriales
Lminuta_Diaporthales
Cchrysosperma_Diaporthales
Dampelina_Diaporthales
Clustricola_Diaporthales
Cpopulea_Diaporthales
taxa
20
40
60
80
100
0
1
2
3
4
5
6
7
8
branch length

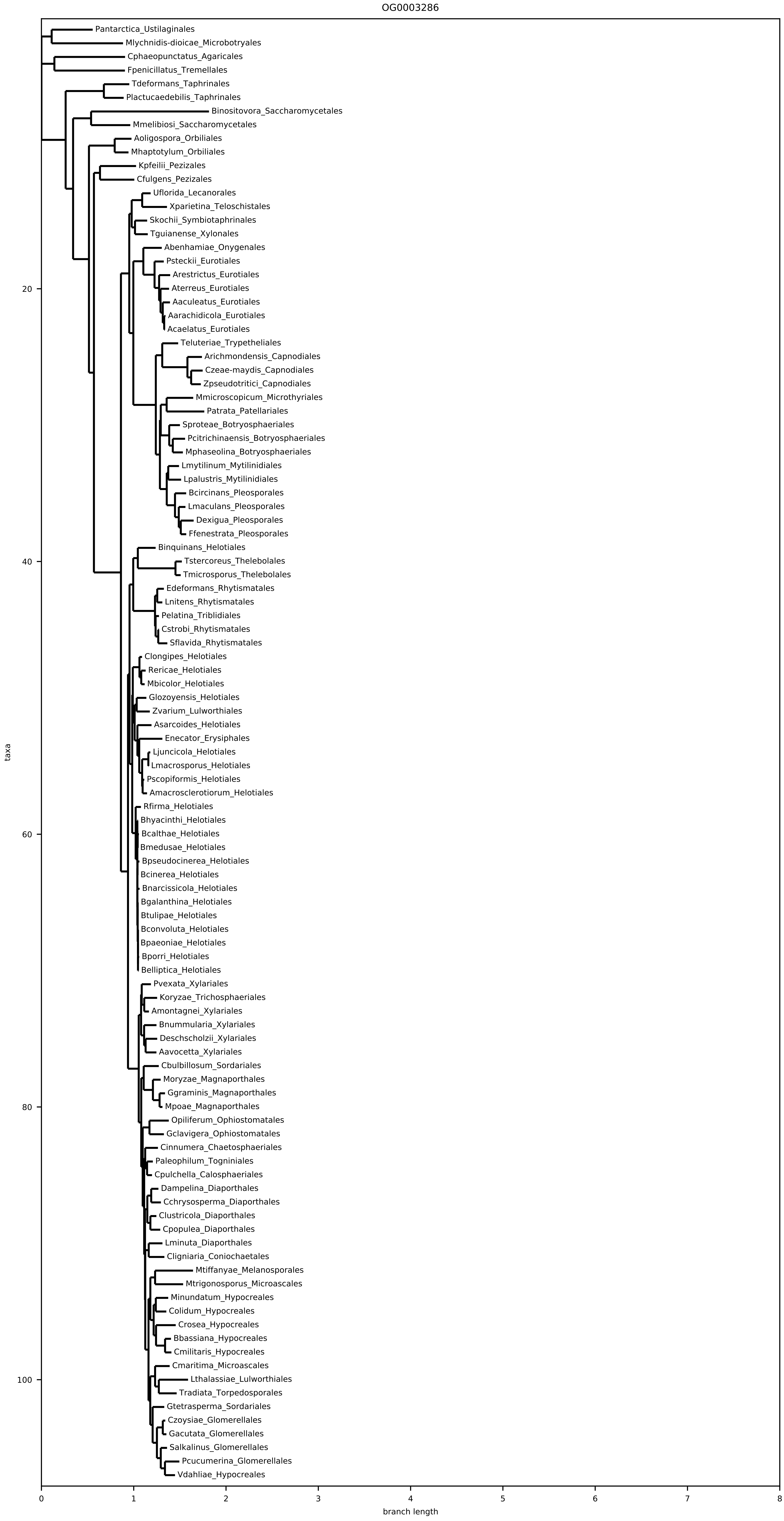

OG0003286
Pantarctica_Ustilaginales
Mlychnidis-dioicae_Microbotryales
Cphaeopunctatus_Agaricales
Fpenicillatus_Tremellales
Tdeformans_Taphrinales
Plactucaedebilis_Taphrinales
Binositovora_Saccharomycetales
Mmelibiosi_Saccharomycetales
Aoligospora_Orbiliales
Mhaptotylum_Orbiliales
Kpfeilii_Pezizales
Cfulgens_Pezizales
Uflorida_Lecanorales
Xparietina_Teloschistales
Skochii_Symbiotaphrinales
Tguianense_Xylonales
Abenhamiae_Onygenales
Psteckii_Eurotiales
Arestrictus_Eurotiales
Aterreus_Eurotiales
Aaculeatus_Eurotiales
Aarachidicola_Eurotiales
Acaelatus_Eurotiales
Teluteriae_Trypetheliales
Arichmondensis_Capnodiales
Czeae-maydis_Capnodiales
Zpseudotritici_Capnodiales
Mmicroscopicum_Microthyriales
Patrata_Patellariales
Sproteae_Botryosphaeriales
Pcitrichinaensis_Botryosphaeriales
Mphaseolina_Botryosphaeriales
Lmytilinum_Mytilinidiales
Lpalustris_Mytilinidiales
Bcircinans_Pleosporales
Lmaculans_Pleosporales
Dexigua_Pleosporales
Ffenestrata_Pleosporales
Binquinans_Helotiales
Tstercoreus_Thelebolales
Tmicrosporus_Thelebolales
Edeformans_Rhytismatales
Lnitens_Rhytismatales
Pelatina_Triblidiales
Cstrobi_Rhytismatales
Sflavida_Rhytismatales
Clongipes_Helotiales
Rericae_Helotiales
Mbicolor_Helotiales
Glozoyensis_Helotiales
Zvarium_Lulworthiales
Asarcoides_Helotiales
Enecator_Erysiphales
Ljuncicola_Helotiales
Lmacrosporus_Helotiales
Pscopiformis_Helotiales
Amacrosclerotiorum_Helotiales
Rfirma_Helotiales
Bhyacinthi_Helotiales
Bcalthae_Helotiales
Bmedusae_Helotiales
Bpseudocinerea_Helotiales
Bcinerea_Helotiales
Bnarcissicola_Helotiales
Bgalanthina_Helotiales
Btulipae_Helotiales
Bconvoluta_Helotiales
Bpaeoniae_Helotiales
Bporri_Helotiales
Belliptica_Helotiales
Pvexata_Xylariales
Koryzae_Trichosphaeriales
Amontagnei_Xylariales
Bnummularia_Xylariales
Deschscholzii_Xylariales
Aavocetta_Xylariales
Cbulbillosum_Sordariales
Moryzae_Magnaporthales
Ggraminis_Magnaporthales
Mpoae_Magnaporthales
Opiliferum_Ophiostomatales
Gclavigera_Ophiostomatales
Cinnumera_Chaetosphaeriales
Paleophilum_Togniniales
Cpulchella_Calosphaeriales
Dampelina_Diaporthales
Cchrysosperma_Diaporthales
Clustricola_Diaporthales
Cpopulea_Diaporthales
Lminuta_Diaporthales
Cligniaria_Coniochaetales
Mtiffanyae_Melanosporales
Mtrigonosporus_Microascales
Minundatum_Hypocreales
Colidum_Hypocreales
Crosea_Hypocreales
Bbassiana_Hypocreales
Cmilitaris_Hypocreales
Cmaritima_Microascales
Lthalassiae_Lulworthiales
Tradiata_Torpedosporales
Gtetrasperma_Sordariales
Czoysiae_Glomerellales
Gacutata_Glomerellales
Salkalinus_Glomerellales
Pcucumerina_Glomerellales
Vdahliae_Hypocreales
taxa
20
40
60
80
100
0
1
2
3
4
5
6
7
8
branch length

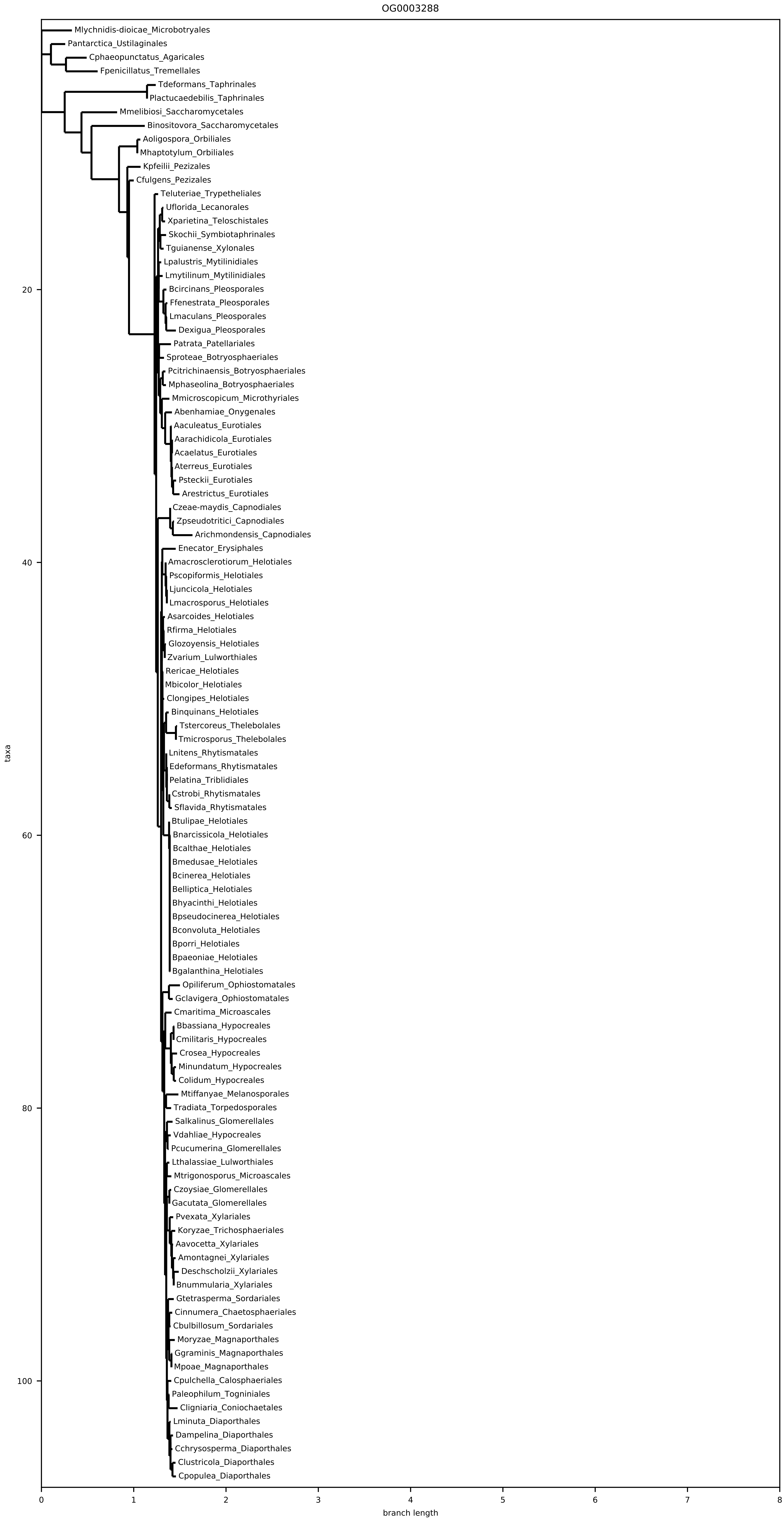
OG0003288
Mlychnidis-dioicae_Microbotryales
Pantarctica_Ustilaginales
Cphaeopunctatus_Agaricales
Fpenicillatus_Tremellales
Tdeformans_Taphrinales
Plactucaedebilis_Taphrinales
Mmelibiosi_Saccharomycetales
Binositovora_Saccharomycetales
Aoligospora_Orbiliales
Mhaptotylum_Orbiliales
Kpfeilii_Pezizales
Cfulgens_Pezizales
Teluteriae_Trypetheliales
Uflorida_Lecanorales
Xparietina_Teloschistales
Skochii_Symbiotaphrinales
Tguianense_Xylonales
Lpalustris_Mytilinidiales
Lmytilinum_Mytilinidiales
Bcircinans_Pleosporales
Ffenestrata_Pleosporales
Lmaculans_Pleosporales
Dexigua_Pleosporales
Patrata_Patellariales
Sproteae_Botryosphaeriales
Pcitrichinaensis_Botryosphaeriales
Mphaseolina_Botryosphaeriales
Mmicroscopicum_Microthyriales
Abenhamiae_Onygenales
Aaculeatus_Eurotiales
Aarachidicola_Eurotiales
Acaelatus_Eurotiales
Aterreus_Eurotiales
Psteckii_Eurotiales
Arestrictus_Eurotiales
Czeae-maydis_Capnodiales
Zpseudotritici_Capnodiales
Arichmondensis_Capnodiales
Enecator_Erysiphales
Amacrosclerotiorum_Helotiales
Pscopiformis_Helotiales
Ljuncicola_Helotiales
Lmacrosporus_Helotiales
Asarcoides_Helotiales
Rfirma_Helotiales
Glozoyensis_Helotiales
Zvarium_Lulworthiales
Rericae_Helotiales
Mbicolor_Helotiales
Clongipes_Helotiales
Binquinans_Helotiales
Tstercoreus_Thelebolales
Tmicrosporus_Thelebolales
Lnitens_Rhytismatales
Edeformans_Rhytismatales
Pelatina_Triblidiales
Cstrobi_Rhytismatales
Sflavida_Rhytismatales
Btulipae_Helotiales
Bnarcissicola_Helotiales
Bcalthae_Helotiales
Bmedusae_Helotiales
Bcinerea_Helotiales
Belliptica_Helotiales
Bhyacinthi_Helotiales
Bpseudocinerea_Helotiales
Bconvoluta_Helotiales
Bporri_Helotiales
Bpaeoniae_Helotiales
Bgalanthina_Helotiales
Opiliferum_Ophiostomatales
Gclavigera_Ophiostomatales
Cmaritima_Microascales
Bbassiana_Hypocreales
Cmilitaris_Hypocreales
Crosea_Hypocreales
Minundatum_Hypocreales
Colidum_Hypocreales
Mtiffanyae_Melanosporales
Tradiata_Torpedosporales
Salkalinus_Glomerellales
Vdahliae_Hypocreales
Pcucumerina_Glomerellales
Lthalassiae_Lulworthiales
Mtrigonosporus_Microascales
Czoysiae_Glomerellales
Gacutata_Glomerellales
Pvexata_Xylariales
Koryzae_Trichosphaeriales
Aavocetta_Xylariales
Amontagnei_Xylariales
Deschscholzii_Xylariales
Bnummularia_Xylariales
Gtetrasperma_Sordariales
Cinnumera_Chaetosphaeriales
Cbulbillosum_Sordariales
Moryzae_Magnaporthales
Ggraminis_Magnaporthales
Mpoae_Magnaporthales
Cpulchella_Calosphaeriales
Paleophilum_Togniniales
Cligniaria_Coniochaetales
Lminuta_Diaporthales
Dampelina_Diaporthales
Cchrysosperma_Diaporthales
Clustricola_Diaporthales
Cpopulea_Diaporthales
taxa
20
40
60
80
100
0
1
2
3
4
5
6
7
8
branch length

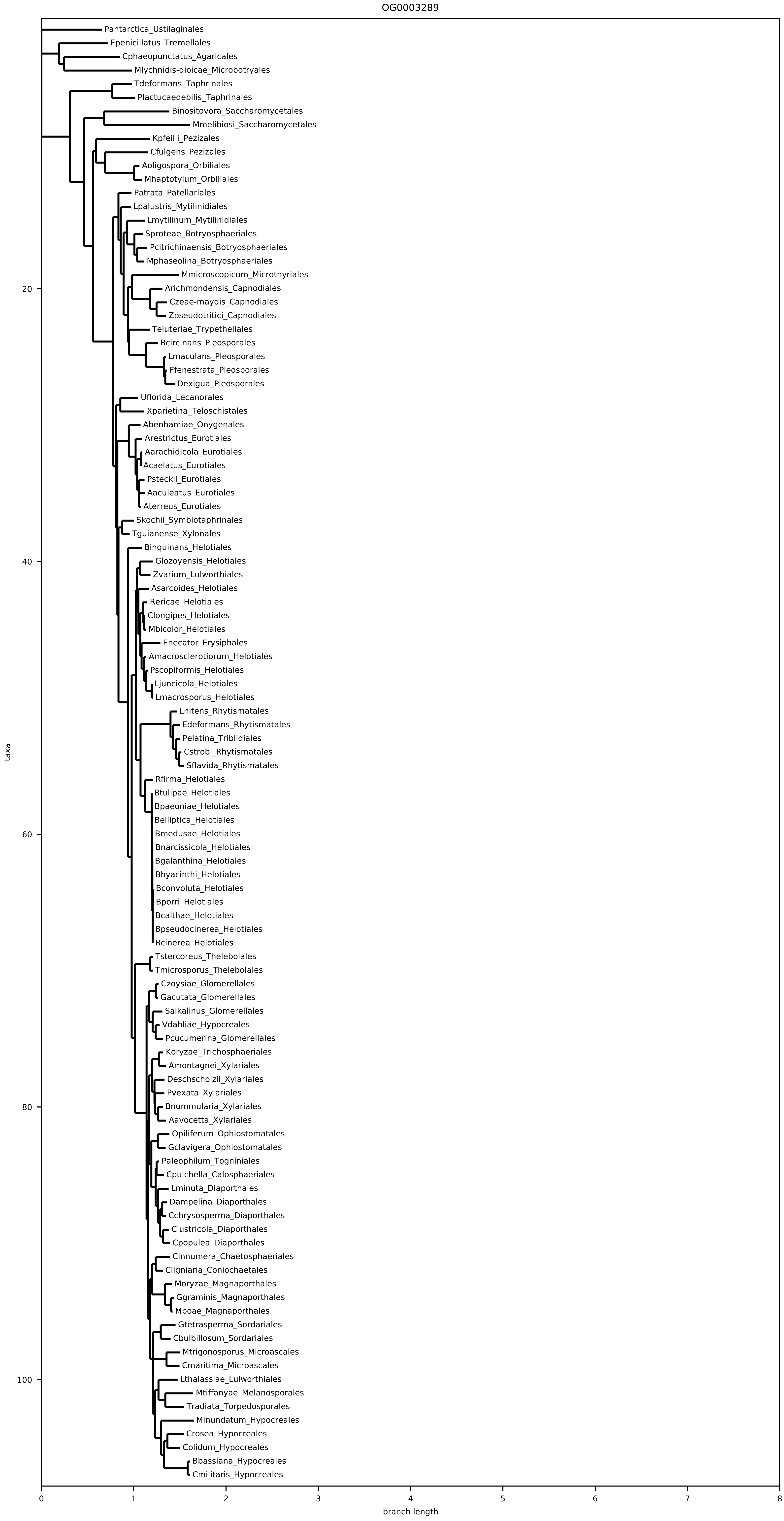
OG0003289
Pantarctica_Ustilaginales
Fpenicillatus_Tremellales
Cphaeopunctatus_Agaricales
Mlychnidis-dioicae_Microbotryales
Tdeformans_Taphrinales
Plactucaedebilis_Taphrinales
Binositovora_Saccharomycetales
Mmelibiosi_Saccharomycetales
Kpfeilii_Pezizales
Cfulgens_Pezizales
Aoligospora_Orbiliales
Mhaptotylum_Orbiliales
Patrata_Patellariales
Lpalustris_Mytilinidiales
Lmytilinum_Mytilinidiales
Sproteae_Botryosphaeriales
Pcitrichinaensis_Botryosphaeriales
Mphaseolina_Botryosphaeriales
Mmicroscopicum_Microthyriales
Arichmondensis_Capnodiales
Czeae-maydis_Capnodiales
Zpseudotritici_Capnodiales
Teluteriae_Trypetheliales
Bcircinans_Pleosporales
Lmaculans_Pleosporales
Ffenestrata_Pleosporales
Dexigua_Pleosporales
Uflorida_Lecanorales
Xparietina_Teloschistales
Abenhamiae_Onygenales
Arestrictus_Eurotiales
Aarachidicola_Eurotiales
Acaelatus_Eurotiales
Psteckii_Eurotiales
Aaculeatus_Eurotiales
Aterreus_Eurotiales
Skochii_Symbiotaphrinales
Tguianense_Xylonales
Binquinans_Helotiales
Glozoyensis_Helotiales
Zvarium_Lulworthiales
Asarcoides_Helotiales
Rericae_Helotiales
Clongipes_Helotiales
Mbicolor_Helotiales
Enecator_Erysiphales
Amacrosclerotiorum_Helotiales
Pscopiformis_Helotiales
Ljuncicola_Helotiales
Lmacrosporus_Helotiales
Lnitens_Rhytismatales
Edeformans_Rhytismatales
Pelatina_Triblidiales
Cstrobi_Rhytismatales
Sflavida_Rhytismatales
Rfirma_Helotiales
Btulipae_Helotiales
Bpaeoniae_Helotiales
Belliptica_Helotiales
Bmedusae_Helotiales
Bnarcissicola_Helotiales
Bgalanthina_Helotiales
Bhyacinthi_Helotiales
Bconvoluta_Helotiales
Bporri_Helotiales
Bcalthae_Helotiales
Bpseudocinerea_Helotiales
Bcinerea_Helotiales
Tstercoreus_Thelebolales
Tmicrosporus_Thelebolales
Czoysiae_Glomerellales
Gacutata_Glomerellales
Salkalinus_Glomerellales
Vdahliae_Hypocreales
Pcucumerina_Glomerellales
Koryzae_Trichosphaeriales
Amontagnei_Xylariales
Deschscholzii_Xylariales
Pvexata_Xylariales
Bnummularia_Xylariales
Aavocetta_Xylariales
Opiliferum_Ophiostomatales
Gclavigera_Ophiostomatales
Paleophilum_Togniniales
Cpulchella_Calosphaeriales
Lminuta_Diaporthales
Dampelina_Diaporthales
Cchrysosperma_Diaporthales
Clustricola_Diaporthales
Cpopulea_Diaporthales
Cinnumera_Chaetosphaeriales
Cligniaria_Coniochaetales
Moryzae_Magnaporthales
Ggraminis_Magnaporthales
Mpoae_Magnaporthales
Gtetrasperma_Sordariales
Cbulbillosum_Sordariales
Mtrigonosporus_Microascales
Cmaritima_Microascales
Lthalassiae_Lulworthiales
Mtiffanyae_Melanosporales
Tradiata_Torpedosporales
Minundatum_Hypocreales
Crosea_Hypocreales
Colidum_Hypocreales
Bbassiana_Hypocreales
Cmilitaris_Hypocreales
taxa
20
40
60
80
100
0
1
2
3
4
5
6
7
8
branch length

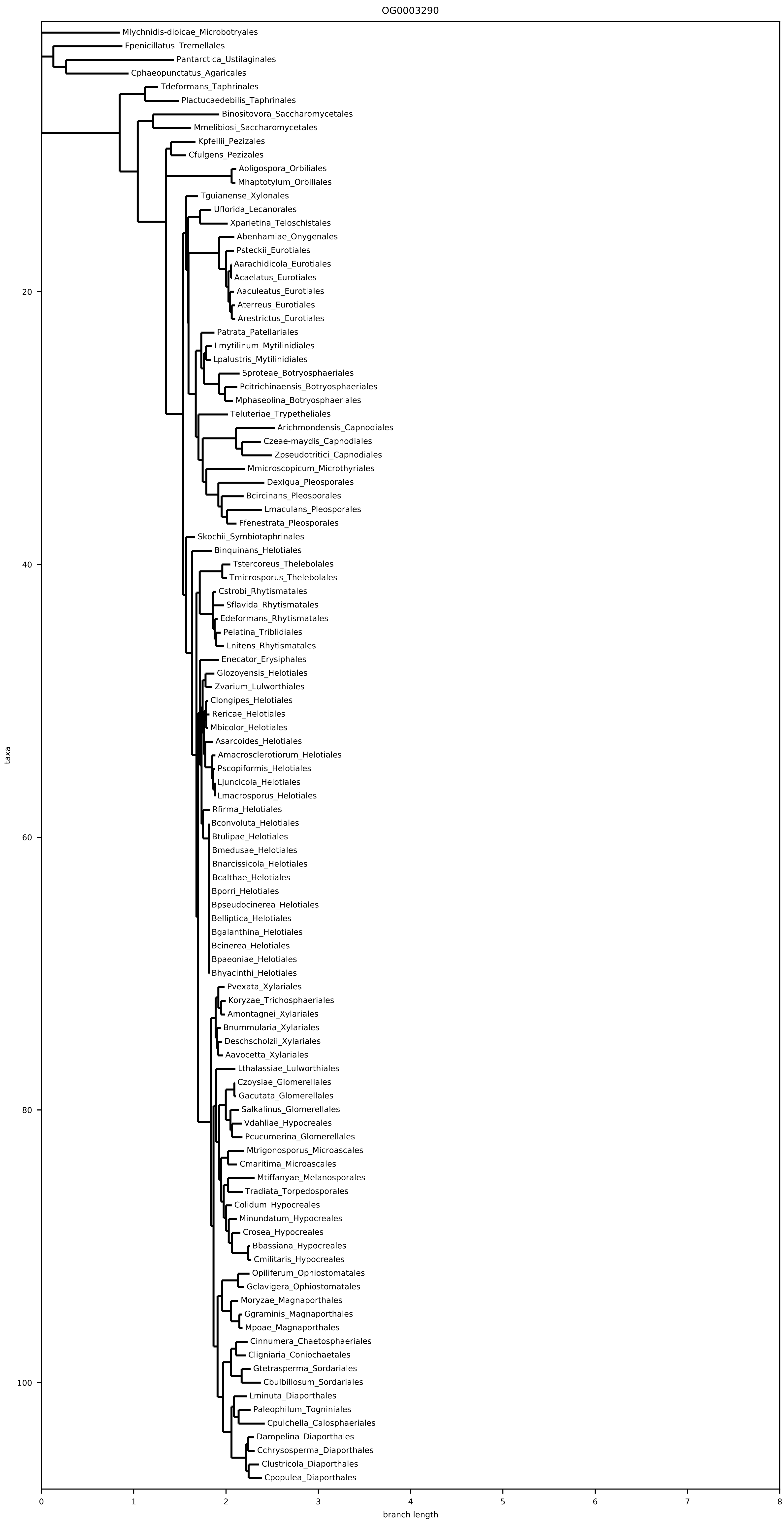
OG0003290
Mlychnidis-dioicae_Microbotryales
Fpenicillatus_Tremellales
Pantarctica_Ustilaginales
Cphaeopunctatus_Agaricales
Tdeformans_Taphrinales
Plactucaedebilis_Taphrinales
Binositovora_Saccharomycetales
Mmelibiosi_Saccharomycetales
Kpfeilii_Pezizales
Cfulgens_Pezizales
Aoligospora_Orbiliales
Mhaptotylum_Orbiliales
Tguianense_Xylonales
Uflorida_Lecanorales
Xparietina_Teloschistales
Abenhamiae_Onygenales
Psteckii_Eurotiales
Aarachidicola_Eurotiales
Acaelatus_Eurotiales
Aaculeatus_Eurotiales
Aterreus_Eurotiales
Arestrictus_Eurotiales
Patrata_Patellariales
Lmytilinum_Mytilinidiales
Lpalustris_Mytilinidiales
Sproteae_Botryosphaeriales
Pcitrichinaensis_Botryosphaeriales
Mphaseolina_Botryosphaeriales
Teluteriae_Trypetheliales
Arichmondensis_Capnodiales
Czeae-maydis_Capnodiales
Zpseudotritici_Capnodiales
Mmicroscopicum_Microthyriales
Dexigua_Pleosporales
Bcircinans_Pleosporales
Lmaculans_Pleosporales
Ffenestrata_Pleosporales
Skochii_Symbiotaphrinales
Binquinans_Helotiales
Tstercoreus_Thelebolales
Tmicrosporus_Thelebolales
Cstrobi_Rhytismatales
Sflavida_Rhytismatales
Edeformans_Rhytismatales
Pelatina_Triblidiales
Lnitens_Rhytismatales
Enecator_Erysiphales
Glozoyensis_Helotiales
Zvarium_Lulworthiales
Clongipes_Helotiales
Rericae_Helotiales
Mbicolor_Helotiales
Asarcoides_Helotiales
Amacrosclerotiorum_Helotiales
Pscopiformis_Helotiales
Ljuncicola_Helotiales
Lmacrosporus_Helotiales
Rfirma_Helotiales
Bconvoluta_Helotiales
Btulipae_Helotiales
Bmedusae_Helotiales
Bnarcissicola_Helotiales
Bcalthae_Helotiales
Bporri_Helotiales
Bpseudocinerea_Helotiales
Belliptica_Helotiales
Bgalanthina_Helotiales
Bcinerea_Helotiales
Bpaeoniae_Helotiales
Bhyacinthi_Helotiales
Pvexata_Xylariales
Koryzae_Trichosphaeriales
Amontagnei_Xylariales
Bnummularia_Xylariales
Deschscholzii_Xylariales
Aavocetta_Xylariales
Lthalassiae_Lulworthiales
Czoysiae_Glomerellales
Gacutata_Glomerellales
Salkalinus_Glomerellales
Vdahliae_Hypocreales
Pcucumerina_Glomerellales
Mtrigonosporus_Microascales
Cmaritima_Microascales
Mtiffanyae_Melanosporales
Tradiata_Torpedosporales
Colidum_Hypocreales
Minundatum_Hypocreales
Crosea_Hypocreales
Bbassiana_Hypocreales
Cmilitaris_Hypocreales
Opiliferum_Ophiostomatales
Gclavigera_Ophiostomatales
Moryzae_Magnaporthales
Ggraminis_Magnaporthales
Mpoae_Magnaporthales
Cinnumera_Chaetosphaeriales
Cligniaria_Coniochaetales
Gtetrasperma_Sordariales
Cbulbillosum_Sordariales
Lminuta_Diaporthales
Paleophilum_Togniniales
Cpulchella_Calosphaeriales
Dampelina_Diaporthales
Cchrysosperma_Diaporthales
Clustricola_Diaporthales
Cpopulea_Diaporthales
taxa
20
40
60
80
100
branch length
0
1
2
3
4
5
6
7
8

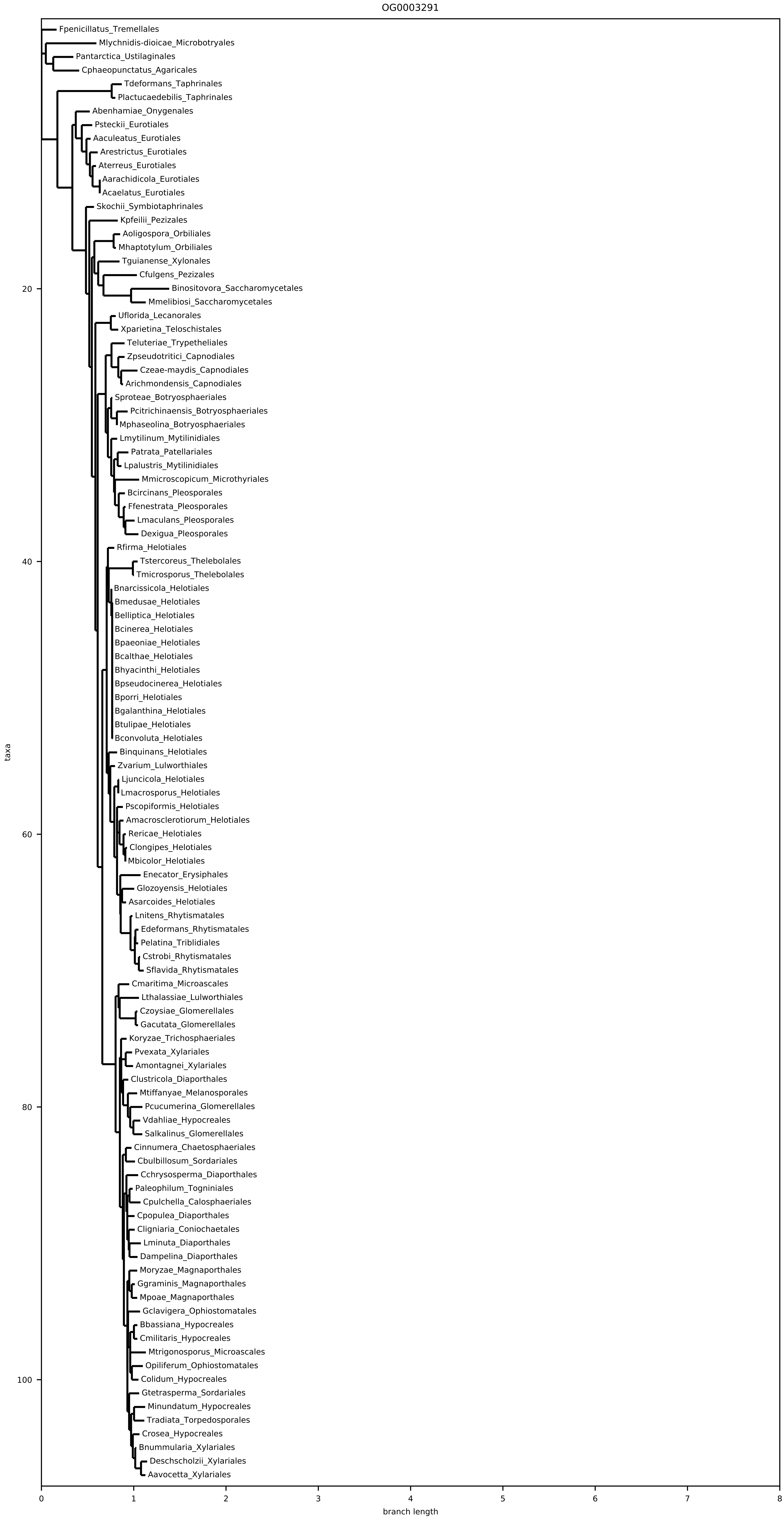
OG0003291
Fpenicillatus_Tremellales
Mlychnidis-dioicae_Microbotryales
Pantarctica_Ustilaginales
Cphaeopunctatus_Agaricales
Tdeformans_Taphrinales
Plactucaedebilis_Taphrinales
Abenhamiae_Onygenales
Psteckii_Eurotiales
Aaculeatus_Eurotiales
Arestrictus_Eurotiales
Aterreus_Eurotiales
Aarachidicola_Eurotiales
Acaelatus_Eurotiales
Skochii_Symbiotaphrinales
Kpfeilii_Pezizales
Aoligospora_Orbiliales
Mhaptotylum_Orbiliales
Tguianense_Xylonales
Cfulgens_Pezizales
Binositovora_Saccharomycetales
Mmelibiosi_Saccharomycetales
Uflorida_Lecanorales
Xparietina_Teloschistales
Teluteriae_Trypetheliales
Zpseudotritici_Capnodiales
Czeae-maydis_Capnodiales
Arichmondensis_Capnodiales
Sproteae_Botryosphaeriales
Pcitrichinaensis_Botryosphaeriales
Mphaseolina_Botryosphaeriales
Lmytilinum_Mytilinidiales
Patrata_Patellariales
Lpalustris_Mytilinidiales
Mmicroscopicum_Microthyriales
Bcircinans_Pleosporales
Ffenestrata_Pleosporales
Lmaculans_Pleosporales
Dexigua_Pleosporales
Rfirma_Helotiales
Tstercoreus_Thelebolales
Tmicrosporus_Thelebolales
Bnarcissicola_Helotiales
Bmedusae_Helotiales
Belliptica_Helotiales
Bcinerea_Helotiales
Bpaeoniae_Helotiales
Bcalthae_Helotiales
Bhyacinthi_Helotiales
Bpseudocinerea_Helotiales
Bporri_Helotiales
Bgalanthina_Helotiales
Btulipae_Helotiales
Bconvoluta_Helotiales
Binquinans_Helotiales
Zvarium_Lulworthiales
Ljuncicola_Helotiales
Lmacrosporus_Helotiales
Pscopiformis_Helotiales
Amacrosclerotiorum_Helotiales
Rericae_Helotiales
Clongipes_Helotiales
Mbicolor_Helotiales
Enecator_Erysiphales
Glozoyensis_Helotiales
Asarcoides_Helotiales
Lnitens_Rhytismatales
Edeformans_Rhytismatales
Pelatina_Triblidiales
Cstrobi_Rhytismatales
Sflavida_Rhytismatales
Cmaritima_Microascales
Lthalassiae_Lulworthiales
Czoysiae_Glomerellales
Gacutata_Glomerellales
Koryzae_Trichosphaeriales
Pvexata_Xylariales
Amontagnei_Xylariales
Clustricola_Diaporthales
Mtiffanyae_Melanosporales
Pcucumerina_Glomerellales
Vdahliae_Hypocreales
Salkalinus_Glomerellales
Cinnumera_Chaetosphaeriales
Cbulbillosum_Sordariales
Cchrysosperma_Diaporthales
Paleophilum_Togniniales
Cpulchella_Calosphaeriales
Cpopulea_Diaporthales
Cligniaria_Coniochaetales
Lminuta_Diaporthales
Dampelina_Diaporthales
Moryzae_Magnaporthales
Ggraminis_Magnaporthales
Mpoae_Magnaporthales
Gclavigera_Ophiostomatales
Bbassiana_Hypocreales
Cmilitaris_Hypocreales
Mtrigonosporus_Microascales
Opiliferum_Ophiostomatales
Colidum_Hypocreales
Gtetrasperma_Sordariales
Minundatum_Hypocreales
Tradiata_Torpedosporales
Crosea_Hypocreales
Bnummularia_Xylariales
Deschscholzii_Xylariales
Aavocetta_Xylariales
taxa
20
40
60
80
100
0
1
2
3
4
5
6
7
8
branch length

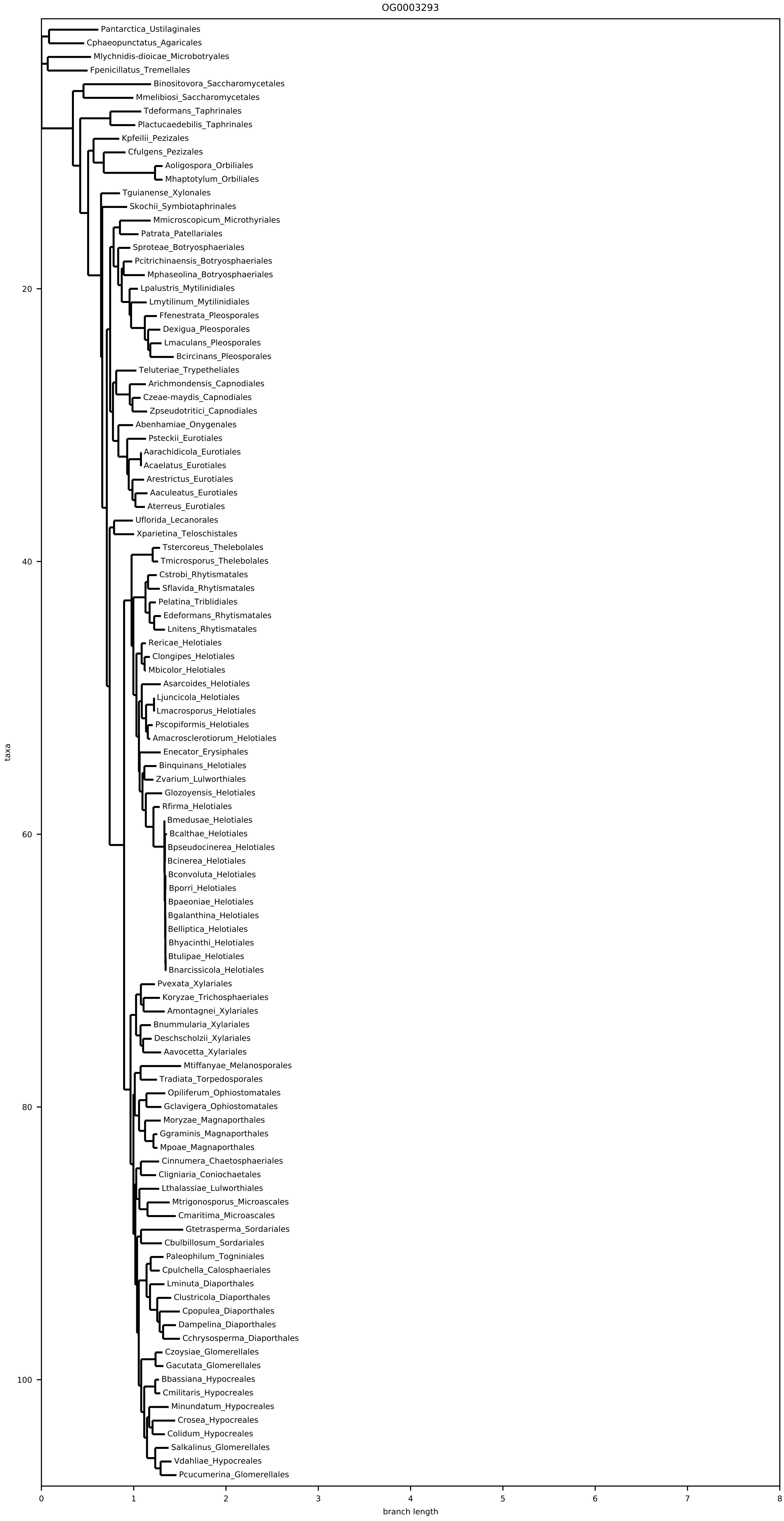

OG0003293
Pantarctica_Ustilaginales
Cphaeopunctatus_Agaricales
Mlychnidis-dioicae_Microbotryales
Fpenicillatus_Tremellales
Binositovora_Saccharomycetales
Mmelibiosi_Saccharomycetales
Tdeformans_Taphrinales
Plactucaedebilis_Taphrinales
Kpfeilii_Pezizales
Cfulgens_Pezizales
Aoligospora_Orbiliales
Mhaptotylum_Orbiliales
Tguianense_Xylonales
Skochii_Symbiotaphrinales
Mmicroscopicum_Microthyriales
Patrata_Patellariales
Sproteae_Botryosphaeriales
Pcitrichinaensis_Botryosphaeriales
Mphaseolina_Botryosphaeriales
Lpalustris_Mytilinidiales
Lmytilinum_Mytilinidiales
Ffenestrata_Pleosporales
Dexigua_Pleosporales
Lmaculans_Pleosporales
Bcircinans_Pleosporales
Teluteriae_Trypetheliales
Arichmondensis_Capnodiales
Czeae-maydis_Capnodiales
Zpseudotritici_Capnodiales
Abenhamiae_Onygenales
Psteckii_Eurotiales
Aarachidicola_Eurotiales
Acaelatus_Eurotiales
Arestrictus_Eurotiales
Aaculeatus_Eurotiales
Aterreus_Eurotiales
Uflorida_Lecanorales
Xparietina_Teloschistales
Tstercoreus_Thelebolales
Tmicrosporus_Thelebolales
Cstrobi_Rhytismatales
Sflavida_Rhytismatales
Pelatina_Triblidiales
Edeformans_Rhytismatales
Lnitens_Rhytismatales
Rericae_Helotiales
Clongipes_Helotiales
Mbicolor_Helotiales
Asarcoides_Helotiales
Ljuncicola_Helotiales
Lmacrosporus_Helotiales
Pscopiformis_Helotiales
Amacrosclerotiorum_Helotiales
Enecator_Erysiphales
Binquinans_Helotiales
Zvarium_Lulworthiales
Glozoyensis_Helotiales
Rfirma_Helotiales
Bmedusae_Helotiales
Bcalthae_Helotiales
Bpseudocinerea_Helotiales
Bcinerea_Helotiales
Bconvoluta_Helotiales
Bporri_Helotiales
Bpaeoniae_Helotiales
Bgalanthina_Helotiales
Belliptica_Helotiales
Bhyacinthi_Helotiales
Btulipae_Helotiales
Bnarcissicola_Helotiales
Pvexata_Xylariales
Koryzae_Trichosphaeriales
Amontagnei_Xylariales
Bnummularia_Xylariales
Deschscholzii_Xylariales
Aavocetta_Xylariales
Mtiffanyae_Melanosporales
Tradiata_Torpedosporales
Opiliferum_Ophiostomatales
Gclavigera_Ophiostomatales
Moryzae_Magnaporthales
Ggraminis_Magnaporthales
Mpoae_Magnaporthales
Cinnumera_Chaetosphaeriales
Cligniaria_Coniochaetales
Lthalassiae_Lulworthiales
Mtrigonosporus_Microascales
Cmaritima_Microascales
Gtetrasperma_Sordariales
Cbulbillosum_Sordariales
Paleophilum_Togniniales
Cpulchella_Calosphaeriales
Lminuta_Diaporthales
Clustricola_Diaporthales
Cpopulea_Diaporthales
Dampelina_Diaporthales
Cchrysosperma_Diaporthales
Czoysiae_Glomerellales
Gacutata_Glomerellales
Bbassiana_Hypocreales
Cmilitaris_Hypocreales
Minundatum_Hypocreales
Crosea_Hypocreales
Colidum_Hypocreales
Salkalinus_Glomerellales
Vdahliae_Hypocreales
Pcucumerina_Glomerellales
taxa
20
40
60
80
100
0
1
2
3
4
5
6
7
8
branch length

OG0003301

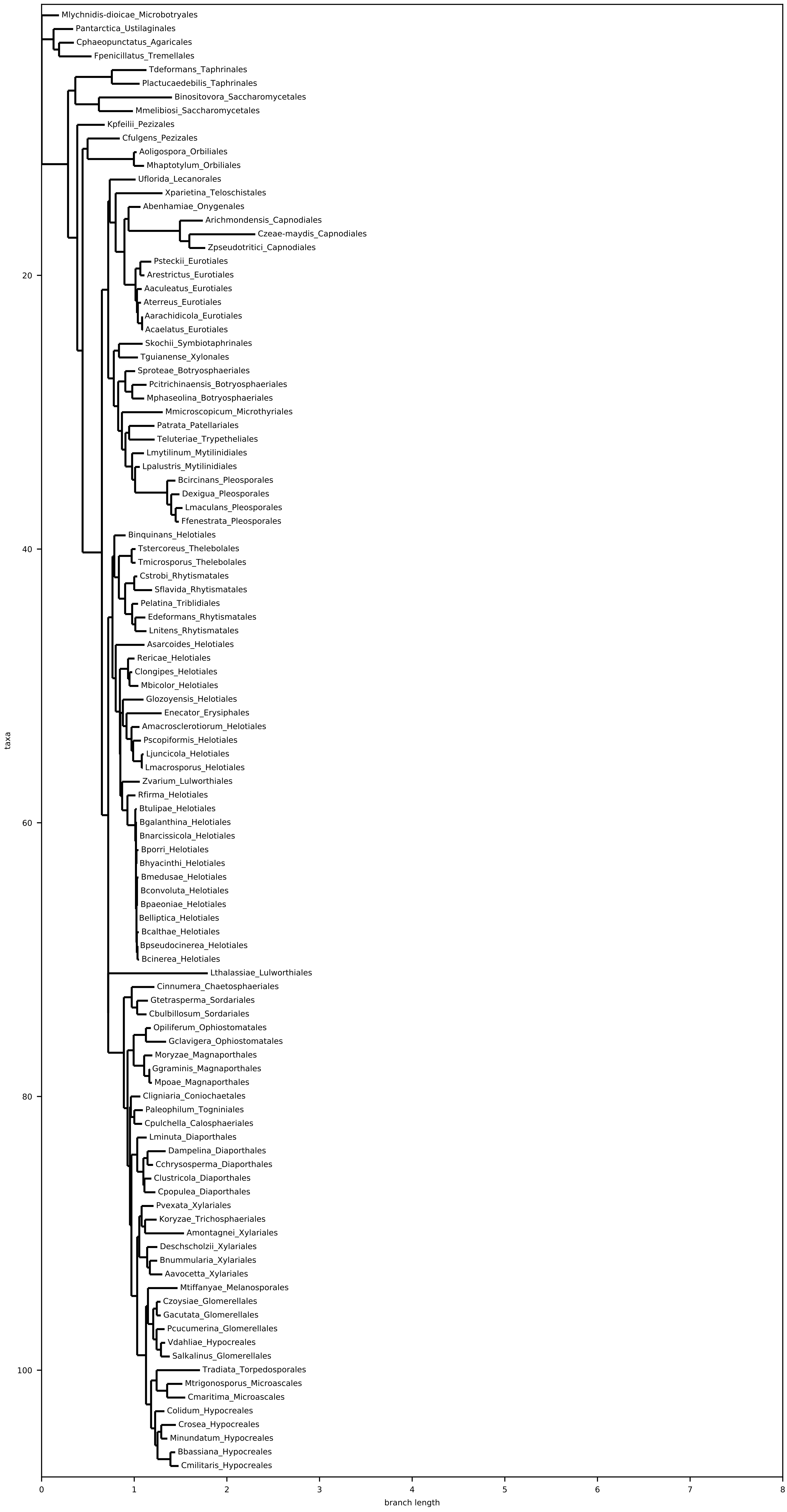
Mlychnidis-dioicae_Microbotryales
Pantarctica_Ustilaginales
Cphaeopunctatus_Agaricales
Fpenicillatus_Tremellales
Tdeformans_Taphrinales
Plactucaedebilis_Taphrinales
Binositovora_Saccharomycetales
Mmelibiosi_Saccharomycetales
Kpfeilii_Pezizales
Cfulgens_Pezizales
Aoligospora_Orbiliales
Mhaptotylum_Orbiliales
Uflorida_Lecanorales
Xparietina_Teloschistales
Abenhamiae_Onygenales
Arichmondensis_Capnodiales
Czeae-maydis_Capnodiales
Zpseudotritici_Capnodiales
Psteckii_Eurotiales
Arestrictus_Eurotiales
Aaculeatus_Eurotiales
Aterreus_Eurotiales
Aarachidicola_Eurotiales
Acaelatus_Eurotiales
Skochii_Symbiotaphrinales
Tguianense_Xylonales
Sproteae_Botryosphaeriales
Pcitrichinaensis_Botryosphaeriales
Mphaseolina_Botryosphaeriales
Mmicroscopicum_Microthyriales
Patrata_Patellariales
Teluteriae_Trypetheliales
Lmytilinum_Mytilinidiales
Lpalustris_Mytilinidiales
Bcircinans_Pleosporales
Dexigua_Pleosporales
Lmaculans_Pleosporales
Ffenestrata_Pleosporales
Binquinans_Helotiales
Tstercoreus_Thelebolales
Tmicrosporus_Thelebolales
Cstrobi_Rhytismatales
Sflavida_Rhytismatales
Pelatina_Triblidiales
Edeformans_Rhytismatales
Lnitens_Rhytismatales
Asarcoides_Helotiales
Rericae_Helotiales
Clongipes_Helotiales
Mbicolor_Helotiales
Glozoyensis_Helotiales
Enecator_Erysiphales
Amacrosclerotiorum_Helotiales
Pscopiformis_Helotiales
Ljuncicola_Helotiales
Lmacrosporus_Helotiales
Zvarium_Lulworthiales
Rfirma_Helotiales
Btulipae_Helotiales
Bgalanthina_Helotiales
Bnarcissicola_Helotiales
Bporri_Helotiales
Bhyacinthi_Helotiales
Bmedusae_Helotiales
Bconvoluta_Helotiales
Bpaeoniae_Helotiales
Belliptica_Helotiales
Bcalthae_Helotiales
Bpseudocinerea_Helotiales
Bcinerea_Helotiales
Lthalassiae_Lulworthiales
Cinnumera_Chaetosphaeriales
Gtetrasperma_Sordariales
Cbulbillosum_Sordariales
Opiliferum_Ophiostomatales
Gclavigera_Ophiostomatales
Moryzae_Magnaporthales
Ggraminis_Magnaporthales
Mpoae_Magnaporthales
Cligniaria_Coniochaetales
Paleophilum_Togniniales
Cpulchella_Calosphaeriales
Lminuta_Diaporthales
Dampelina_Diaporthales
Cchrysosperma_Diaporthales
Clustricola_Diaporthales
Cpopulea_Diaporthales
Pvexata_Xylariales
Koryzae_Trichosphaeriales
Amontagnei_Xylariales
Deschscholzii_Xylariales
Bnummularia_Xylariales
Aavocetta_Xylariales
Mtiffanyae_Melanosporales
Czoysiae_Glomerellales
Gacutata_Glomerellales
Pcucumerina_Glomerellales
Vdahliae_Hypocreales
Salkalinus_Glomerellales
Tradiata_Torpedosporales
Mtrigonosporus_Microascales
Cmaritima_Microascales
Colidum_Hypocreales
Crosea_Hypocreales
Minundatum_Hypocreales
Bbassiana_Hypocreales
Cmilitaris_Hypocreales
taxa
20
40
60
80
100
0
1
2
3
4
5
6
7
8
branch length

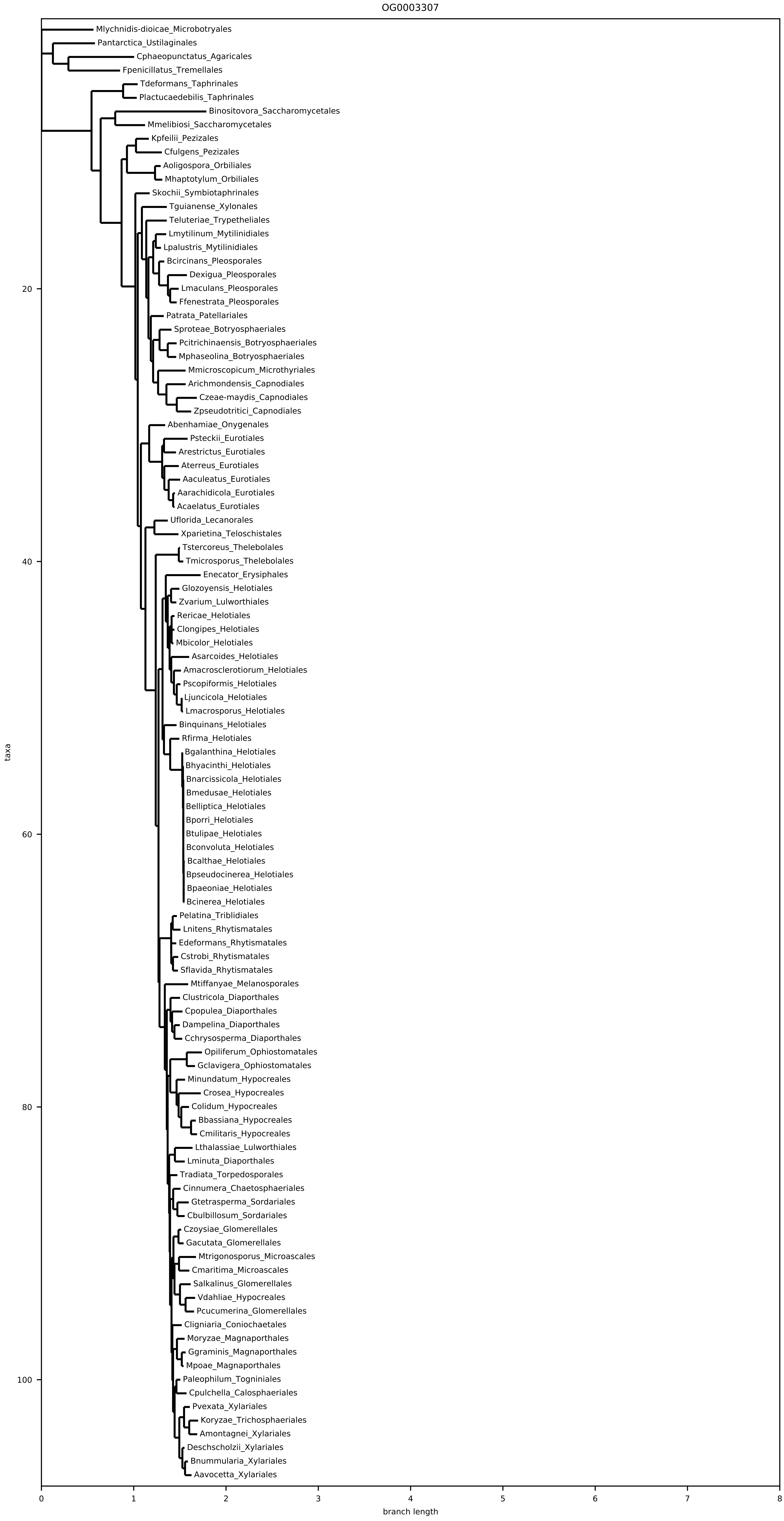
OG0003307
Mlychnidis-dioicae_Microbotryales
Pantarctica_Ustilaginales
Cphaeopunctatus_Agaricales
Fpenicillatus_Tremellales
Tdeformans_Taphrinales
Plactucaedebilis_Taphrinales
Binositovora_Saccharomycetales
Mmelibiosi_Saccharomycetales
Kpfeilii_Pezizales
Cfulgens_Pezizales
Aoligospora_Orbiliales
Mhaptotylum_Orbiliales
Skochii_Symbiotaphrinales
Tguianense_Xylonales
Teluteriae_Trypetheliales
Lmytilinum_Mytilinidiales
Lpalustris_Mytilinidiales
Bcircinans_Pleosporales
Dexigua_Pleosporales
Lmaculans_Pleosporales
Ffenestrata_Pleosporales
Patrata_Patellariales
Sproteae_Botryosphaeriales
Pcitrichinaensis_Botryosphaeriales
Mphaseolina_Botryosphaeriales
Mmicroscopicum_Microthyriales
Arichmondensis_Capnodiales
Czeae-maydis_Capnodiales
Zpseudotritici_Capnodiales
Abenhamiae_Onygenales
Psteckii_Eurotiales
Arestrictus_Eurotiales
Aterreus_Eurotiales
Aaculeatus_Eurotiales
Aarachidicola_Eurotiales
Acaelatus_Eurotiales
Uflorida_Lecanorales
Xparietina_Teloschistales
Tstercoreus_Thelebolales
Tmicrosporus_Thelebolales
Enecator_Erysiphales
Glozoyensis_Helotiales
Zvarium_Lulworthiales
Rericae_Helotiales
Clongipes_Helotiales
Mbicolor_Helotiales
Asarcoides_Helotiales
Amacrosclerotiorum_Helotiales
Pscopiformis_Helotiales
Ljuncicola_Helotiales
Lmacrosporus_Helotiales
Binquinans_Helotiales
Rfirma_Helotiales
Bgalanthina_Helotiales
Bhyacinthi_Helotiales
Bnarcissicola_Helotiales
Bmedusae_Helotiales
Belliptica_Helotiales
Bporri_Helotiales
Btulipae_Helotiales
Bconvoluta_Helotiales
Bcalthae_Helotiales
Bpseudocinerea_Helotiales
Bpaeoniae_Helotiales
Bcinerea_Helotiales
Pelatina_Triblidiales
Lnitens_Rhytismatales
Edeformans_Rhytismatales
Cstrobi_Rhytismatales
Sflavida_Rhytismatales
Mtiffanyae_Melanosporales
Clustricola_Diaporthales
Cpopulea_Diaporthales
Dampelina_Diaporthales
Cchrysosperma_Diaporthales
Opiliferum_Ophiostomatales
Gclavigera_Ophiostomatales
Minundatum_Hypocreales
Crosea_Hypocreales
Colidum_Hypocreales
Bbassiana_Hypocreales
Cmilitaris_Hypocreales
Lthalassiae_Lulworthiales
Lminuta_Diaporthales
Tradiata_Torpedosporales
Cinnumera_Chaetosphaeriales
Gtetrasperma_Sordariales
Cbulbillosum_Sordariales
Czoysiae_Glomerellales
Gacutata_Glomerellales
Mtrigonosporus_Microascales
Cmaritima_Microascales
Salkalinus_Glomerellales
Vdahliae_Hypocreales
Pcucumerina_Glomerellales
Cligniaria_Coniochaetales
Moryzae_Magnaporthales
Ggraminis_Magnaporthales
Mpoae_Magnaporthales
Paleophilum_Togniniales
Cpulchella_Calosphaeriales
Pvexata_Xylariales
Koryzae_Trichosphaeriales
Amontagnei_Xylariales
Deschscholzii_Xylariales
Bnummularia_Xylariales
Aavocetta_Xylariales
taxa
20
40
60
80
100
0
1
2
3
4
5
6
7
8
branch length

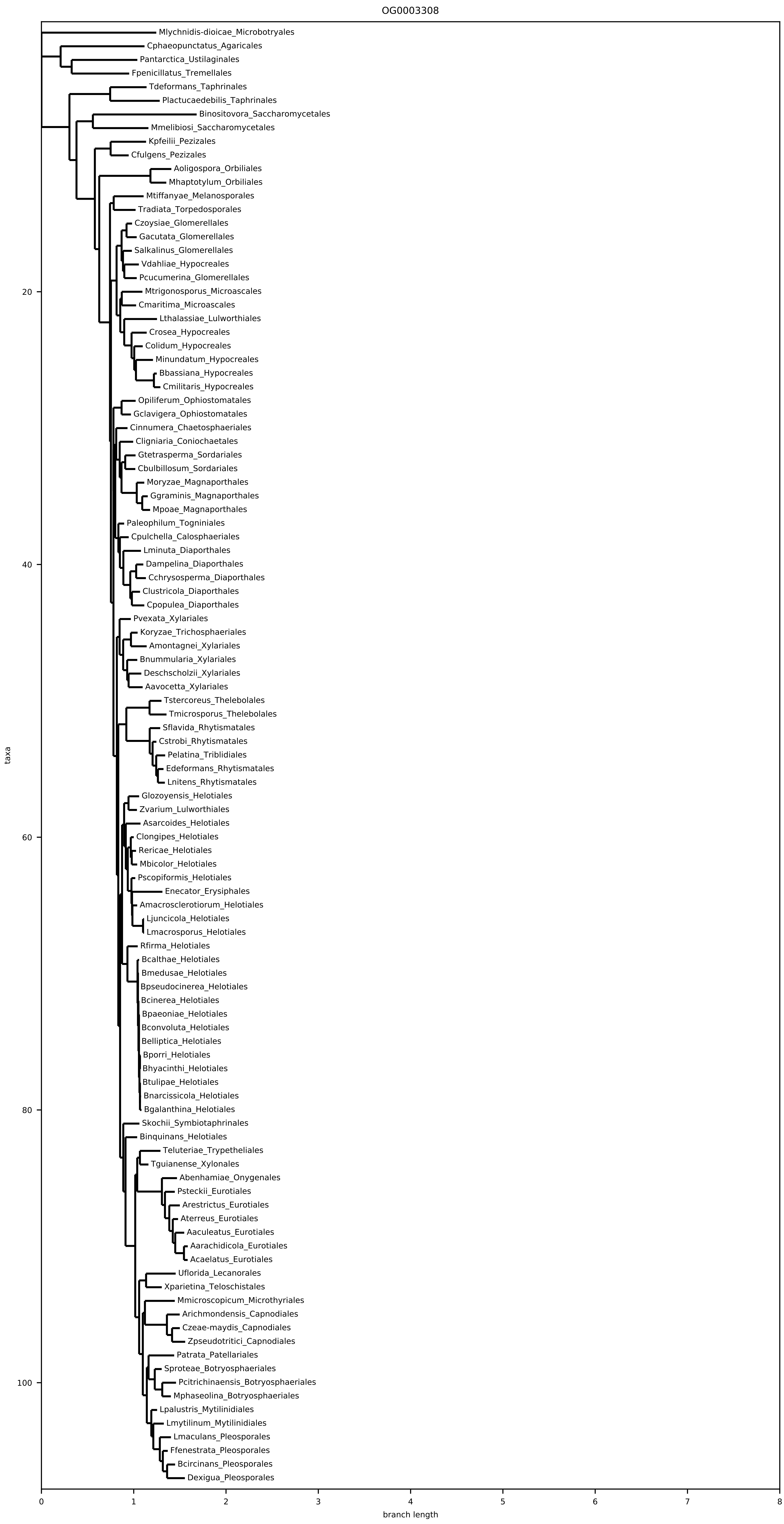
OG0003308
Mlychnidis-dioicae_Microbotryales
Cphaeopunctatus_Agaricales
Pantarctica_Ustilaginales
Fpenicillatus_Tremellales
Tdeformans_Taphrinales
Plactucaedebilis_Taphrinales
Binositovora_Saccharomycetales
Mmelibiosi_Saccharomycetales
Kpfeilii_Pezizales
Cfulgens_Pezizales
Aoligospora_Orbiliales
Mhaptotylum_Orbiliales
Mtiffanyae_Melanosporales
Tradiata_Torpedosporales
Czoysiae_Glomerellales
Gacutata_Glomerellales
Salkalinus_Glomerellales
Vdahliae_Hypocreales
Pcucumerina_Glomerellales
Mtrigonosporus_Microascales
Cmaritima_Microascales
Lthalassiae_Lulworthiales
Crosea_Hypocreales
Colidum_Hypocreales
Minundatum_Hypocreales
Bbassiana_Hypocreales
Cmilitaris_Hypocreales
Opiliferum_Ophiostomatales
Gclavigera_Ophiostomatales
Cinnumera_Chaetosphaeriales
Cligniaria_Coniochaetales
Gtetrasperma_Sordariales
Cbulbillosum_Sordariales
Moryzae_Magnaporthales
Ggraminis_Magnaporthales
Mpoae_Magnaporthales
Paleophilum_Togniniales
Cpulchella_Calosphaeriales
Lminuta_Diaporthales
Dampelina_Diaporthales
Cchrysosperma_Diaporthales
Clustricola_Diaporthales
Cpopulea_Diaporthales
Pvexata_Xylariales
Koryzae_Trichosphaeriales
Amontagnei_Xylariales
Bnummularia_Xylariales
Deschscholzii_Xylariales
Aavocetta_Xylariales
Tstercoreus_Thelebolales
Tmicrosporus_Thelebolales
Sflavida_Rhytismatales
Cstrobi_Rhytismatales
Pelatina_Triblidiales
Edeformans_Rhytismatales
Lnitens_Rhytismatales
Glozoyensis_Helotiales
Zvarium_Lulworthiales
Asarcoides_Helotiales
Clongipes_Helotiales
Rericae_Helotiales
Mbicolor_Helotiales
Pscopiformis_Helotiales
Enecator_Erysiphales
Amacrosclerotiorum_Helotiales
Ljuncicola_Helotiales
Lmacrosporus_Helotiales
Rfirma_Helotiales
Bcalthae_Helotiales
Bmedusae_Helotiales
Bpseudocinerea_Helotiales
Bcinerea_Helotiales
Bpaeoniae_Helotiales
Bconvoluta_Helotiales
Belliptica_Helotiales
Bporri_Helotiales
Bhyacinthi_Helotiales
Btulipae_Helotiales
Bnarcissicola_Helotiales
Bgalanthina_Helotiales
Skochii_Symbiotaphrinales
Binquinans_Helotiales
Teluteriae_Trypetheliales
Tguianense_Xylonales
Abenhamiae_Onygenales
Psteckii_Eurotiales
Arestrictus_Eurotiales
Aterreus_Eurotiales
Aaculeatus_Eurotiales
Aarachidicola_Eurotiales
Acaelatus_Eurotiales
Uflorida_Lecanorales
Xparietina_Teloschistales
Mmicroscopicum_Microthyriales
Arichmondensis_Capnodiales
Czeae-maydis_Capnodiales
Zpseudotritici_Capnodiales
Patrata_Patellariales
Sproteae_Botryosphaeriales
Pcitrichinaensis_Botryosphaeriales
Mphaseolina_Botryosphaeriales
Lpalustris_Mytilinidiales
Lmytilinum_Mytilinidiales
Lmaculans_Pleosporales
Ffenestrata_Pleosporales
Bcircinans_Pleosporales
Dexigua_Pleosporales
taxa
20
40
60
80
100
0
1
2
3
4
5
6
7
8
branch length

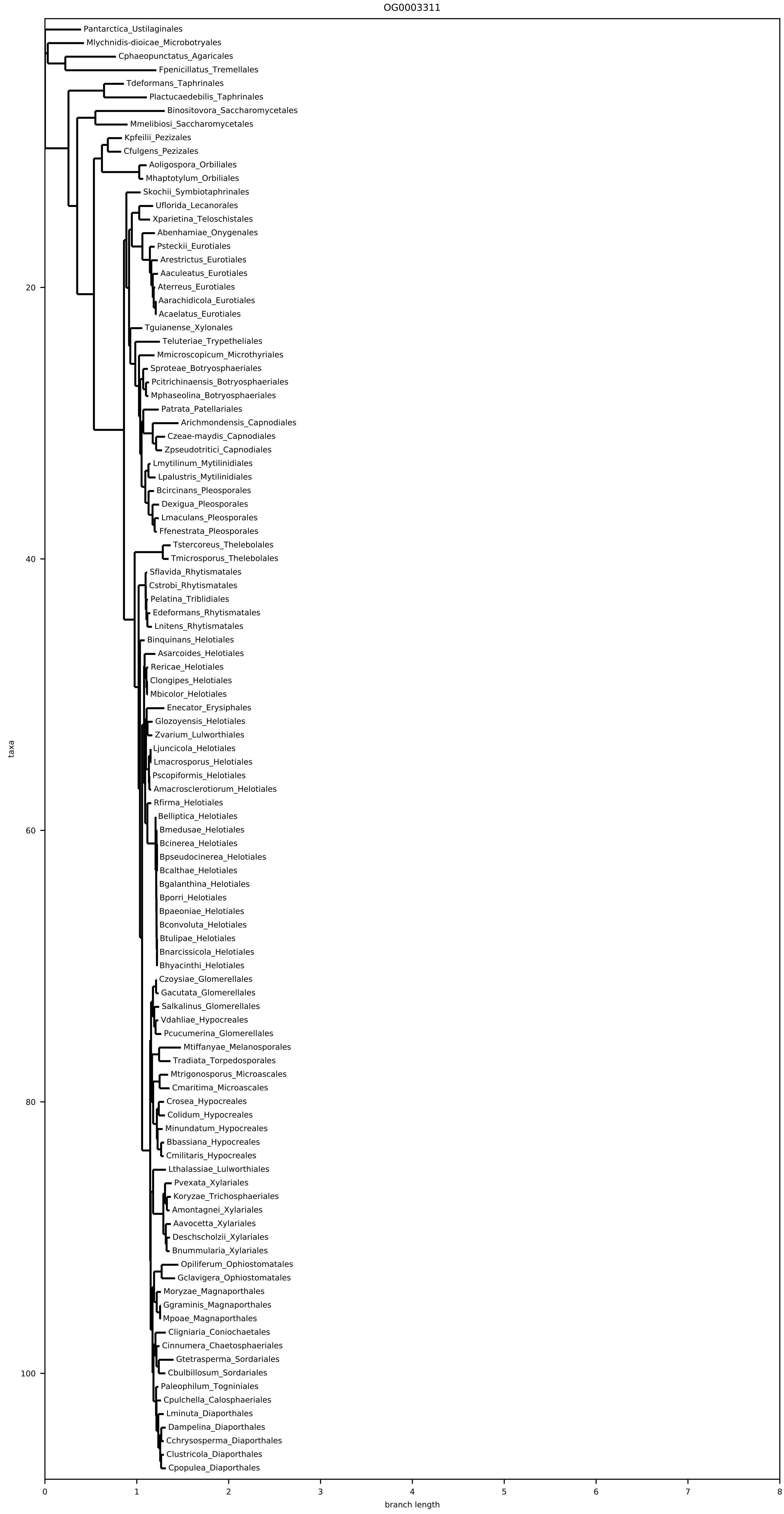
OG0003311
Pantarctica_Ustilaginales
Mlychnidis-dioicae_Microbotryales
Cphaeopunctatus_Agaricales
Fpenicillatus_Tremellales
Tdeformans_Taphrinales
Plactucaedebilis_Taphrinales
Binositovora_Saccharomycetales
Mmelibiosi_Saccharomycetales
Kpfeilii_Pezizales
Cfulgens_Pezizales
Aoligospora_Orbiliales
Mhaptotylum_Orbiliales
Skochii_Symbiotaphrinales
Uflorida_Lecanorales
Xparietina_Teloschistales
Abenhamiae_Onygenales
Psteckii_Eurotiales
Arestrictus_Eurotiales
Aaculeatus_Eurotiales
Aterreus_Eurotiales
Aarachidicola_Eurotiales
Acaelatus_Eurotiales
Tguianense_Xylonales
Teluteriae_Trypetheliales
Mmicroscopicum_Microthyriales
Sproteae_Botryosphaeriales
Pcitrichinaensis_Botryosphaeriales
Mphaseolina_Botryosphaeriales
Patrata_Patellariales
Arichmondensis_Capnodiales
Czeae-maydis_Capnodiales
Zpseudotritici_Capnodiales
Lmytilinum_Mytilinidiales
Lpalustris_Mytilinidiales
Bcircinans_Pleosporales
Dexigua_Pleosporales
Lmaculans_Pleosporales
Ffenestrata_Pleosporales
Tstercoreus_Thelebolales
Tmicrosporus_Thelebolales
Sflavida_Rhytismatales
Cstrobi_Rhytismatales
Pelatina_Triblidiales
Edeformans_Rhytismatales
Lnitens_Rhytismatales
Binquinans_Helotiales
Asarcoides_Helotiales
Rericae_Helotiales
Clongipes_Helotiales
Mbicolor_Helotiales
Enecator_Erysiphales
Glozoyensis_Helotiales
Zvarium_Lulworthiales
Ljuncicola_Helotiales
Lmacrosporus_Helotiales
Pscopiformis_Helotiales
Amacrosclerotiorum_Helotiales
Rfirma_Helotiales
Belliptica_Helotiales
Bmedusae_Helotiales
Bcinerea_Helotiales
Bpseudocinerea_Helotiales
Bcalthae_Helotiales
Bgalanthina_Helotiales
Bporri_Helotiales
Bpaeoniae_Helotiales
Bconvoluta_Helotiales
Btulipae_Helotiales
Bnarcissicola_Helotiales
Bhyacinthi_Helotiales
Czoysiae_Glomerellales
Gacutata_Glomerellales
Salkalinus_Glomerellales
Vdahliae_Hypocreales
Pcucumerina_Glomerellales
Mtiffanyae_Melanosporales
Tradiata_Torpedosporales
Mtrigonosporus_Microascales
Cmaritima_Microascales
Crosea_Hypocreales
Colidum_Hypocreales
Minundatum_Hypocreales
Bbassiana_Hypocreales
Cmilitaris_Hypocreales
Lthalassiae_Lulworthiales
Pvexata_Xylariales
Koryzae_Trichosphaeriales
Amontagnei_Xylariales
Aavocetta_Xylariales
Deschscholzii_Xylariales
Bnummularia_Xylariales
Opiliferum_Ophiostomatales
Gclavigera_Ophiostomatales
Moryzae_Magnaporthales
Ggraminis_Magnaporthales
Mpoae_Magnaporthales
Cligniaria_Coniochaetales
Cinnumera_Chaetosphaeriales
Gtetrasperma_Sordariales
Cbulbillosum_Sordariales
Paleophilum_Togniniales
Cpulchella_Calosphaeriales
Lminuta_Diaporthales
Dampelina_Diaporthales
Cchrysosperma_Diaporthales
Clustricola_Diaporthales
Cpopulea_Diaporthales
taxa
20
40
60
80
100
0
1
2
3
4
5
6
7
8
branch length
